# Practical and Scalable Kinetic Resolution of BINOLs Mediated by a Chiral Counterion

**DOI:** 10.1002/anie.201814381

**Published:** 2019-03-05

**Authors:** Benjamin A. Jones, Tudor Balan, John D. Jolliffe, Craig D. Campbell, Martin D. Smith

**Affiliations:** ^1^ Chemistry Research Laboratory University of Oxford 12 Mansfield Road Oxford OX1 3TA UK

**Keywords:** ammonium, axial chirality, BINOL, counterion, phase-transfer

## Abstract

BINOLs are valuable and widely used building blocks, chiral ligands, and catalysts that are effective across a remarkable range of different chemical transformations. Here we demonstrate that an ammonium salt catalyzed kinetic resolution of racemic BINOLs with benzyl tosylate proceeds with s up to 46. This is a scalable and practical process that can be applied across >30 different *C*_2_‐ and non‐*C*_2_‐symmetric BINOLs. Implementation of this method enables the enantioselective synthesis of a wide range of BINOL derivatives with over 99:1 e.r.

Atropisomeric biaryls, exemplified by 1,1′‐binapthyl‐2,2′‐diol (BINOL)^1^ are valuable scaffolds for catalysis, materials, and medicine. BINOL itself is a building block for numerous catalyst scaffolds. *C*_2_‐symmetric BINOLs are generally accessible through derivatization of enantiopure BINOL, which can be accessed by resolution^2^ and also from commercial sources.^3^ However, this strategy is not always feasible for non‐*C*_2_‐symmetric derivatives.^4^ One approach to this problem that may extend across a wide range of substrates is kinetic resolution (KR)—the achievement of resolution by virtue of unequal rates of reaction.^5^, ^6^ This is a valuable technique for obtaining extremely high levels of enantioenrichment in unreacted substrate through high selectivity factors (*s*) and control of conversion. Previous approaches to the KR^7^ of BINOLs include Tsuji's palladium catalyzed alcoholysis of vinyl ether derivatives using a chiral diamine ligand, which enabled the recovery of enantioenriched unreacted substrate with *s* up to 36 (Figure 1 A).^8^ An alternative approach from the Sibi group (Figure 1 B) involves employing a chiral 4‐dimethylaminopyridine equivalent to enable enantioselective *O*‐acylation of a mono‐functionalized BINOL derivative with *s* up to 38.^9^ A related acylative approach from Zhao (Figure 1 C) employs an *N*‐heterocyclic carbene in the presence of an α‐alkoxyaldehyde. Through internal redox, this generates an *N*‐acyl azolium salt that leads to kinetic resolution of BINOLs with *s* up to >100.^10^ There are a number of enzymatic kinetic resolutions of BINOL that can deliver enantioenriched materials;^11^ of particular note is the KR and dynamic KR from Akai that employs immobilized *Pseudomonas* sp. lipoprotein lipase (Toyobo LIP‐301), in the presence of isopropenyl acetate (Figure 1 D).^12^

**Figure 1:**
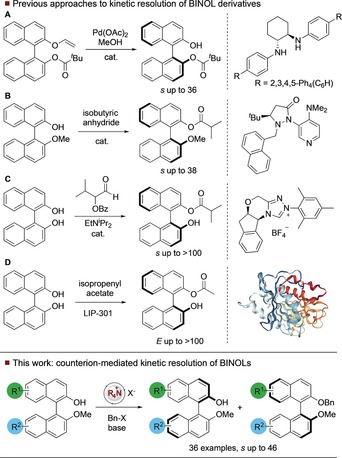
Previous approaches to catalytic KR of BINOLS, and our strategy.

We reasoned that an enantioselective *O*‐functionalization, mediated by a chiral counterion^13^ in the presence of an alkylating agent, could effect a simple and functional group tolerant KR of BINOLs. We have previously demonstrated that racemic 2‐tetralones can be *O*‐alkylated with high levels of atropselectivity.^14^ Here we build on this approach but extend our strategy to the *O*‐functionalization of naptholates. This could enable access to non‐*C*_2_‐symmetric BINOLs with the high levels of enantioenrichment required for the construction of catalysts, whilst also being a scalable and operationally simple procedure.

Our investigation started with the kinetic resolution of monomethyl BINOL **1** (Table 1). *O*‐Alkylation of **1** with benzyl bromide in the presence of *N*‐benzyl quinidinium chloride **3** and potassium phosphate gave poor conversion (13 %) and *s* of 3.1 (entry 1). This could be significantly augmented through the selection of catalysts bearing electron withdrawing groups on the *N*‐benzyl moiety: catalyst **4** gave better conversion and a much‐improved selectivity (*s*=13, entry 2). The use of benzyl iodide as the electrophile gave a broadly similar selectivity (*s*=12, entry 3), but higher conversion (53 %) was achieved when using benzene as the solvent (entry 4). This is a valuable observation, as temporal control of conversion is important for attaining high levels of enantioselectivity in kinetic resolutions. We explored this further with a change of electrophile to benzyl tosylate;^15^ this gave lower selectivity (*s*=8.5, entry 5) but enabled greater control of conversion. Consequently, we reasoned that through optimization of catalyst structure we would be able to build upon and modify this set of reaction conditions to attain sufficiently high selectivity in the kinetic resolution.

**Table 1:** Optimization: kinetic resolution of BINOLs.^[a]^ 
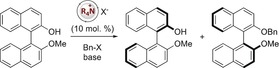

Entry	Cat.	Base^[b]^	Solvent	Bn‐X	*s* ^[c]^
1	3	K_3_PO_4_	PhMe	BnBr	3.1
2	4	K_3_PO_4_	PhMe	BnBr	13
3	4	K_3_PO_4_	PhMe	BnI	12
4	4	K_3_PO_4_	PhH	BnI	14
5	4	K_2_CO_3_	PhH	BnOTs	8.5
6	5	K_2_CO_3_	PhH	BnOTs	21
7	6	K_2_CO_3_	PhH	BnOTs	21
8	7	K_2_CO_3_	PhH	BnOTs	21
9	8	K_2_CO_3_	PhH	BnOTs	33^[d]^
10	8	K_2_CO_3_	PhH:Et_2_O^[e]^	BnOTs	35^[d]^
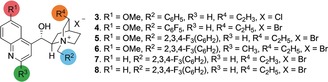					

[a] Conditions: substrate **1** (0.02 mmol), cat. (0.1 equiv), base (5.0 equiv), solvent ([substrate]=0.1 m), 298 K, 48 h. [b] base: 50 % aq., w/w. [c] *s* determined from enantiomeric ratio (e.r.) values of unreacted starting material and *O*‐alkylated product; e.r. values were determined by chiral stationary phase HPLC. [d] [substrate]=0.025 M. [e] 9:1 PhH:Et_2_O, v/v.

We were able to increase selectivity through the application of catalyst **5**, which bears a trisubstituted arene on the pendant *N*‐substituent (*s*=21, entry 6).^16^ We next examined the effect of small changes around this catalyst structure. Catalyst **6** bears a 2‐methyl group on the quinoline and was designed to test if quinoline *N*‐functionalization was affecting performance; this gave identical selectivity (*s*=21, entry 7). We also examined whether the quinidine backbone was more effective than the cinchonine backbone through evaluation of catalyst **7**; this ultimately gave identical selectivity (*s*=21, entry 8) though a shorter reaction time to reach viable conversion was deemed advantageous. Examination of catalyst **8**, which bears a 3‐ethyl group gave significantly better performance when operated at a lower concentration (*s*=33, entry 9) which was further augmented (to *s*=35, entry 10) through the application of diethyl ether as a co‐solvent; furthermore, this reduced reaction time to 24 h.

With a working process in hand, we examined the scope of our counterion‐mediated kinetic resolution across both *C*_2_‐ and non *C*_2_‐symmetric substrates (Table 2). Monomethyl BINOL can be alkylated with *s*=35, enabling recovery of unreacted substrate **1** in 47 % yield (98:2 e.r.); ^[17]^ the absolute configuration of the major enantiomer of **1** is (*R*)‐, through comparison with literature values and by preparation of an authentic sample from (*R*)‐BINOL.^18^ Removal of the *O*‐alkyl groups in **1** or its *O*‐benzylated analogue is easily achieved.^14^ The alkyl group can be varied to encompass both *O*‐isopropyl **9** and benzyl ethers **10**. 6′‐Nitro‐BINOL derivative **11** can be recovered from the alkylation reaction in 39 % yield and >99:1 e.r. (*s*=46). Alkyl substituents such as methyl are also tolerated in the 6′ position as in **12**. Non‐symmetric 7‐substituted systems are broadly well tolerated with both controllable conversion and high selectivity. Thus substrates **13**–**17** are all effective in the KR with good selectivity factors, enabling isolation of substrate in usefully high levels of enantioenrichment. Substitution in the 6‐position is possible without deleterious effects on the reaction: **18**–**21** are all successfully *O*‐alkylated with good selectivity. Variation in the substituent at the 5‐position is also well tolerated: **22**–**24** can be isolated with high levels of selectivity. Phenanthrene biaryl **25** is also selectively alkylated (*s*=30). There is relatively little sensitivity to changes in substituents in the 4‐position: **26**–**29** are all viable substrates that proceed to afford recovered substrate in good yields and enantioselectivities. *C*_2_‐symmetric substrates are also tolerated under this protocol. 7,7′‐dibromo derivative **30** functions well under the optimized reaction conditions (*s*=33). However, these positions appear to be somewhat sensitive to substitution: **31** and **32** are both less selective substrates than most others. In contrast, 6,6′‐dibromo BINOL substrate **33** (*s*=34) is a significantly better substrate for the reaction, as is the 6,6′‐diphenyl substrate **34** (*s*=39). Changing the aryl group as in **35**–**37** is also well tolerated with little effect on selectivity 5,5′‐substituted BINOLS are also good substrates for the kinetic resolution: **38** and **39** are successful and selective substrates but 5,5′‐diphenyl substrate **40** is significantly less selective. We are also able to resolve substrates bearing substitution in the 4,4′ positions, but this appears to be relatively sensitive to the size of the substituent: 4,4′‐dibromo **41** (*s*=21) is relatively selective, but 4,4′‐diphenyl substrate **42** (*s*=16) is significantly less successful. In contrast, a smaller substituent (4,4′‐dimethyl substrate **43**) enables isolation of unreacted starting substrate in good selectivity (*s*=34).

**Table 2:** Scope of counterion‐mediated kinetic resolution of C_2_‐ and non‐C_2_‐symmetric BINOLs.

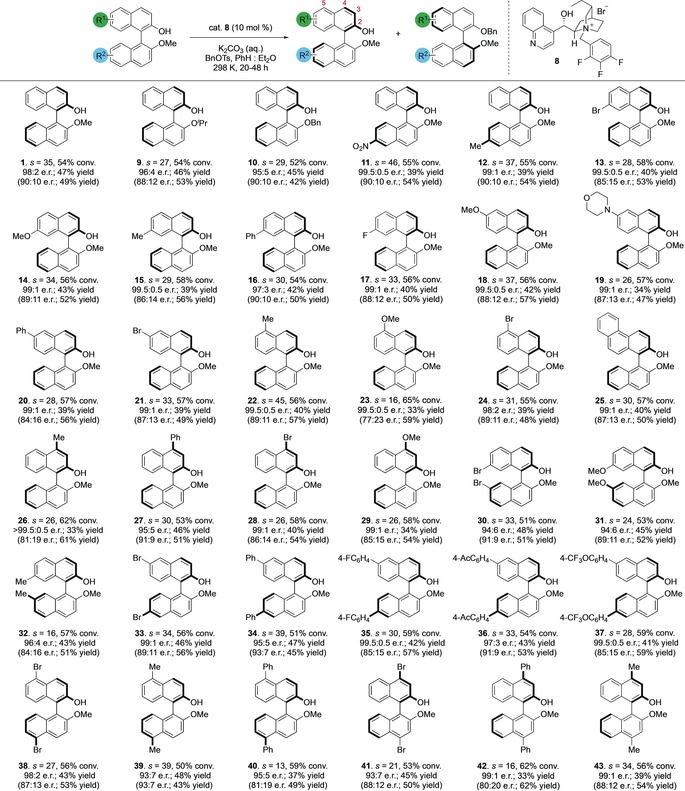

Reaction conditions: BINOL (0.15 mmol), cat. **8** (0.1 equiv), K_2_CO_3_ (5.0 equiv, sat. aq.), BnOTs (1.5 equiv), 9:1 PhH:Et_2_O (v/v, [BINOL]=0.025 m); reaction time 20–48 h, 298 K.^19^ Conversions and *s*electivity factors were determined from e.r. values of unreacted starting material and *O*‐alkylated product^20^ (see Supporting Information for full details). e.r. and yield are given first for unreacted starting material; figures in parentheses that follow are for *O*‐benzylated product. Enantiomeric ratios were determined by chiral stationary phase HPLC. Yields are for isolated and purified material. Positions around the BINOL core are indicated in red numerals.

The scalability and practicability of this process is demonstrated by a 22 mmol scale reaction (Scheme 1).

**Scheme 1:**
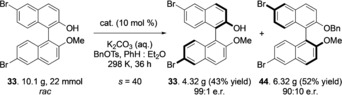
Reaction conditions: BINOL **33** (22 mmol), cat. **8** (0.1 equiv), K_2_CO_3_ (5.0 equiv, sat. aq.), BnOTs (1.5 equiv), 9:1 PhH:Et_2_O (v/v, [BINOL]=0.025 m); reaction time 36 h, 298 K. Enantiomeric ratios were determined by chiral stationary phase HPLC. Yields are for isolated and purified material.

Treatment of *rac*‐BINOL derivative **33** (10.1 g) with catalyst **8** (synthesized in 2 steps from commercial material) and benzyl tosylate enabled isolation of enantioenriched unreacted BINOL **33** in 43 % yield and 99:1 e.r. (*s*=40 at 54 % conversion). This result demonstrates that the KR process can be applied on a practical multi‐gram scale for those interested in the synthesis of highly enantioenriched BINOLs.

From a mechanistic perspective, we propose that rapid and reversible deprotonation of **1** affords diastereoisomeric BINOLate ammonium salts **45** and **46**, and that these react at different rates in the alkylation step in the presence of benzyl tosylate (Figure 2).

**Figure 2:**
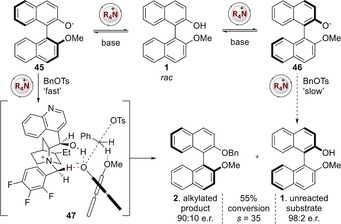
Proposed mechanism of observed kinetic resolution.

A proposed transition structure **47** involves hydrogen bonding from the α‐C−H of the ammonium salt and the secondary alcohol to the BINOLate anion, with an additional C−H⋅⋅⋅O hydrogen bond from the benzylic electrophile C−H to the methyl ether. This is consistent with a recently proposed model from Fan and Hong and reflects the observed absolute configuration of the *O*‐benzylated product and unreacted substrate.^21^

In conclusion, we have demonstrated that a readily available chiral ammonium salt can discriminate between the enantiomeric forms of a wide range of BINOLs in an alkylative KR process. This is a selective, viable and practical process for the synthesis of useful quantities of both *C*_2_ and non‐*C*_2_‐symmetric BINOLs at the high levels of enantioselectivity required for catalysis and related applications.

## Conflict of interest

The authors declare no conflict of interest.

## Supporting information

As a service to our authors and readers, this journal provides supporting information supplied by the authors. Such materials are peer reviewed and may be re‐organized for online delivery, but are not copy‐edited or typeset. Technical support issues arising from supporting information (other than missing files) should be addressed to the authors.

Supplementary

## References

[1] J. M. Brunel , Chem. Rev. 2005, 105, 857–897. 15755079 10.1021/cr040079g

[2] The formation of inclusion complexes is the most efficient method for the resolution of BINOL on a preparative scale. For complexation with *N*-benzylcinchoninium chloride see

[2a] K. Tanaka , T. Okada , F. Toda , Angew. Chem. Int. Ed. Engl. 1993, 32, 1147–1148;

[2b] D. Cai , D. L. Hughes , T. R. Verhoeven , P. J. Reider , Tetrahedron Lett. 1995, 36, 7991–7994;

[2c] D. Cai , D. L. Hughes , T. R. Verhoeven , P. J. Reider , Org. Synth. 1999, 76, 1–5; For complexation with proline see

[2d] X. Hu , Z. Shan , Q. Chang , Tetrahedron: Asymmetry 2012, 23, 1327–1131.

[3] Enantiopure BINOL can be purchased for less than 1GBP per gram from many sources.

[4a] P. Kočovský , Š. Vyskočil , M. Smrčina , Chem. Rev. 2003, 103, 3213–3245; 12914496 10.1021/cr9900230

[4b] H. Gao , Q.-L. Xu , M. Yousufuddin , D. H. Ess , L. Kürti , Angew. Chem. Int. Ed. 2016, 55, 566–571; 10.1002/anie.201508419 PMC4824624 26592491

[5] H. B. Kagan , J. C. Fiaud , Top. Stereochem. 1988, 18, 249–330.

[6] M. D. Greenhalgh , J. E. Taylor , A. D. Smith , Tetrahedron 2018, 74, 5554–5560.

[7] G. Ma , M. P. Sibi , Chem. Eur. J. 2015, 21, 11644–11657. 26237330 10.1002/chem.201500869

[8] H. Aoyama , M. Tokunaga , J. Kiyosu , T. Iwasawa , Y. Obora , Y. Tsuji , J. Am. Chem. Soc. 2005, 127, 10474–10475. 16045319 10.1021/ja051750h

[9a] G. Ma , J. Deng , M. P. Sibi , Angew. Chem. Int. Ed. 2014, 53, 11818–11821; 10.1002/anie.201406684 25124842

[9b] S. Qu , M. D. Greenhalgh , A. D. Smith , Chem. Eur. J. 2019, 10.1002/chem.201805631.

[10] S. Liu , S. B. Poh , Y. Zhao , Angew. Chem. Int. Ed. 2014, 53, 11041–11045; 10.1002/anie.201406192 25145856

[11a] Y. Fujimoto , H. Iwadate , N. Ikekawa , J. Chem. Soc. Chem. Commun. 1985, 1333–1334;

[11b] S. Miyano , K. Kawahara , Y. Inoue , H. Hashimoto , Chem. Lett. 1987, 16, 355–356;

[11c] M. Inagaki , J. Hiratake , T. Nishioka , J. Oda , Agric. Biol. Chem. 1989, 53, 1879–1884;

[11d] R. J. Kazlauskas , J. Am. Chem. Soc. 1989, 111, 4953–4959;

[11e] T. Furutani , M. Hatsuda , R. Imashiro , M. Seki , Tetrahedron: Asymmetry 1999, 10, 4763–4768;

[11f] M. Juárez-Hernandez , D. V. Johnson , H. L. Holland , J. McNulty , A. Capretta , Tetrahedron: Asymmetry 2003, 14, 289–291.

[12] G. A. I. Moustafa , Y. Oki , S. Akai , Angew. Chem. Int. Ed. 2018, 57, 10278–10282; 10.1002/anie.201804161 29704286

[13a] S. Shirakawa , K. Liu , K. Maruoka , J. Am. Chem. Soc. 2012, 134, 916–919; 22208662 10.1021/ja211069f

[13b] S. Shirakawa , X. Wu , K. Maruoka , Angew. Chem. Int. Ed. 2013, 52, 14200–14203; 10.1002/anie.201308237 24222438

[14] J. D. Jolliffe , R. J. Armstrong , M. D. Smith , Nat. Chem. 2017, 9, 558–562. 28537599 10.1038/nchem.2710

[15] F. Kazemi , A. R. Massah , M. Javaherian , Tetrahedron 2007, 63, 5083–5087.

[16] For full details of optimization see supplementary information.

[17] Our selectivity factors are invariant with substrate concentration, consistent with a first order reaction.

[18] See supplementary information for full details of absolute configuration confirmation. For optical rotation and other data see reference [13] and: F. Ishiwari , K.-I. Fukasawa , T. Sato , K. Nakazono , Y. Koyama , T. Takata , Chem. Eur. J. 2011, 17, 12067–12075. 21922578 10.1002/chem.201101727

[19] It is important to quench excess benzyl tosylate to stop the reaction at the required conversion. This is performed by the addition of piperidine prior to workup and product isolation.

[20] J. M. Keith , J. F. Larrow , E. N. Jacobsen , Adv. Synth. Catal. 2001, 343, 5.

[21] H. Li , W. Fan , X. Hong , Org. Biomol. Chem. 2019, 17, 1916. 30280168 10.1039/c8ob02173b

