# Supplementary material for: Practical and Scalable Kinetic Resolution of BINOLs Mediated by a Chiral Counterion
Source: Angew Chem Int Ed Engl. 2019 Mar 5;58(14):4596–600. doi: 10.1002/anie.201814381 (PMC6492193; doi:10.1002/anie.201814381)
Supplement: Supplementary file 1 — Supplementary [file ANIE-58-4596-s001.pdf]

Angewandte Chemie
Eine Zeitschrift der Gesellschaft Deutscher Chemiker

Supporting Information

Practical and Scalable Kinetic Resolution of BINOLs Mediated by a Chiral Counterion

*Benjamin A. Jones[+], Tudor Balan[+], John D. Jolliffe, Craig D. Campbell, and Martin D. Smith**

anie_201814381_sm_miscellaneous_information.pdf

1. **General Information…………………………………………………………………………………………… S2**
2. **Catalyst Synthesis…………………………………………………………………………………………… S3**
3. **Extended Optimization Table……………………………………………………………………………… S4**
4. **Synthesis of Racemic BINOLs………………………………………………………………………… S11**
   4.1. 7-Substituted BINOLs……………………………………………………………………………… S12
   4.2. 6-Substituted BINOLs……………………………………………………………………………… S20
   4.3. 5-Substituted BINOLs……………………………………………………………………………… S30
   4.4. 4 -Substituted BINOLs………………………………………………………………………………S39
   4.5. BINOLs Prepared by Other Synthetic Routes………………………………………………S49
5. **General Procedure for the Counterion Mediated Kinetic Resolution of BINOLs………S61**
6. **Counterion Mediated Kinetic Resolution of BINOLs…………………………………………… S63**
7. **10 g Scale Kinetic Resolution…………………………………………………………………………. S99**
8. **Determination of Absolute Stereochemistry…………………………………………………….S100**
9. **References………………………………………………………………………………………………….S103**
10. **NMR Spectra and HPLC Traces………………………………………………………………………S104**

1. General Information

Reactions requiring moisture-sensitive reagents were carried out in flame-dried glassware, under an atmosphere of argon (balloon pressure). Dichloromethane, tetrahydrofuran and toluene were purified by filtration through activated alumina columns employing the method of Grubbs et al.[1] Water was purified by an Elix® UV-10 system. Reagents were used directly as supplied by major chemical suppliers, or following purification procedures described by Perrin and Armarego.[2] Petrol 40-60 refers to the fraction of petroleum ether which boils in the range 40-60 °C. Brine refers to a saturated aqueous solution of sodium chloride.

Silica gel chromatography was carried out using Merck Geduran® Silica gel (40-63 µm particle size). Thin layer chromatography (TLC) was carried out using pre-coated, aluminium backed plates (Merck Kieselgel 60 F254). Visualisation was achieved with ultraviolet irradiation (254 nm) and staining with permanganate. NMR spectroscopy was carried out using Bruker Avance spectrometers in the deuterated solvent stated, using the residual non-deuterated solvent signal as an internal reference (^{1}H NMR: $CDCl_3$ (7.26), $(CD_3)_2SO$ (2.50); ^{13}C NMR: $CDCl_3$ (77.16), $(CD_3)_2SO$ (39.52); ^{19}F NMR: $CFCl_3$ (0.00)). Chemical shifts are quoted in ppm, based on appearance rather than interpretation. Signal patterns are indicated as: br s, broad singlet; s, singlet; d, doublet; t, triplet; q, quartet; m, multiplet. Coupling constants, *J*, are quoted to the nearest 0.1 Hz and are presented as observed. All ^{19}F NMR spectra are reported as proton/fluorine decoupled unless otherwise stated.

Infrared spectra were prepared as a neat film and were recorded using a Bruker Tensor 27 FTIR spectrometer using an ATR module.

Low resolution mass spectrometry was carried out using ESI and was performed on a Micromass LCT Premier Spectrometer. HRMS was carried out using Bruker MicroTDF and Micromass GCT spectrometers under electrospray ionization (ESI) or ammonia chemical ionization (ACI)/electron ionization (EI) conditions respectively. Analytical chiral HPLC was carried out on a Dionex UltiMate 3000 HPLC system comprising a Dionex LPG-3400A pump, WPS-3000SL autosampler and TCC-3000SD column S2 compartment, and a Daicel Chiralpak column (0.46 cm × 25 cm), equipped with an appropriate guard column (0.4 cm × 1 cm). For compound **Bn-15** (2-(benzyloxy)-2'-methoxy-7-methyl-1,1'-binaphthalene), chiral analytical HPLC was performed on a SHIMADZU Prominence-*i* LC2030-LT instrument. Melting points were determined using a Reichert melting point apparatus and are uncorrected.

Optical rotations were recorded on a Schmidt-Haensch Unipol L2000 polarimeter and values are quoted in deg·mL·g^{-1}·dm^{-1}. Concentrations are quoted in g/100 mL.

2. Catalyst synthesis

Representative procedure:

N-(2,3,4-trifluorobenzyl)hydrocinchoninium bromide (8)

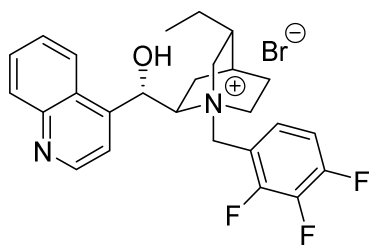

To a suspension of hyndrocinchonine (1.00 g, 3.37 mmol, 1.00 equiv) in tetrahydrofuran (34.0 mL) was added 2,3,4-trifluorobenzyl bromide (759 mg, 0.44 mL, 3.37 mmol, 1.00 equiv). The mixture was heated at 75 °C for 16 h and cooled to room temperature to give a white precipitate. The solids were collected in a funnel and washed successively with diethyl ether (200 mL), tetrahydrofuran (200 mL), ethyl acetate (100 mL) and petroleum ether 40-60 (200 mL). The resulting powder was dried under vacuum to give N-(2,3,4-trifluorobenzyl)hydrocinchoninium bromide (**8**) (1.57 g, 89% yield) as a white solid.

m.p. > 250 °C (CH_2Cl_2/MeOH)

IR (film) ν_{max}/cm^{-1}: 3164, 2959, 1516, 1493, 1457, 1307, 1126, 1072, 1044, 1013, 995, 963, 931, 899, 813, 777, 762, 753, 684, 636, 616.

^{1}H NMR (500 MHz, $CDCl_3$) δ 8.83 (d, *J* = 3.6 Hz, 1H), 8.28 (d, *J* = 8.3 Hz, 1H), 8.25 (br. s, 1H), 7.86 (d, *J* = 3.4 Hz, 1H), 7.51 (d, *J* = 8.1 Hz, 1H), 7.05 (q, *J* = 7.3 Hz, 1H), 6.96 (t, *J* = 7.1 Hz, 1H), 6.45 (app d, *J* = 19.0 Hz, 2H), 6.21 (d, *J* = 11.9 Hz, 1H), 5.52 (d, *J* = 12.1 Hz, 1H), 4.33-4.17 (m, 3H), 2.99 (dd, , *J* = 11.2, 8.4 Hz, 1H), 2.67 (q, *J* = 9.9 Hz, 1H), 2.00 (app t, *J* = 12.1 Hz, 1H), 1.89-1.58 (m, 4H), 1.59-1.37 (m, 3H), 0.90-0.76 (m, 3H), 0.70 (br s, 1H).

^{13}C NMR (126 MHz, $CDCl_3$, ^{19}F decoupled) δ 149.5, 147.1, 130.3, 129.6, 128.3, 127.0, 123.6, 123.5, 119.7, 113.1, 113.0, 67.3, 65.8, 57.4, 56.5, 53.6, 36.2, 24.6, 24.3, 24.2, 21.8, 11.4.

HRMS (ESI$^+$) $C_{26}H_{28}ON_2F_3^+$ [M-Br]$^+$: requires 441.21482, found: 441.21426, Δ -1.3 ppm.

3. Extended optimisation table

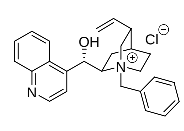
CN1

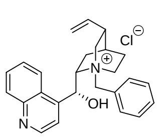
CD2

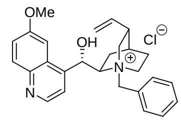
3

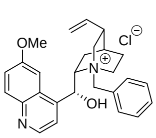
QN3

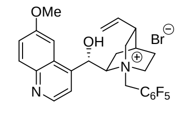
QD4

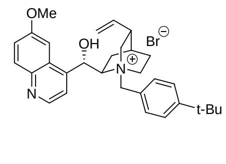
QD5

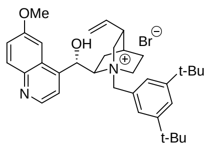
QD6

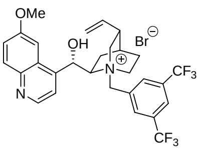
QD7

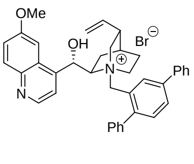
QD8

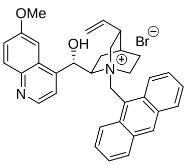
QD9

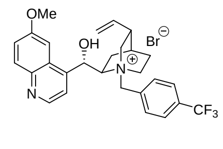
QD10

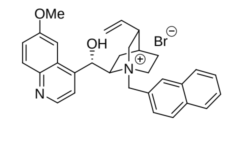
QD11

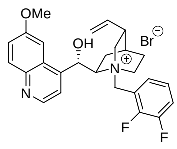
QD12

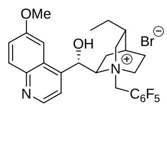
4

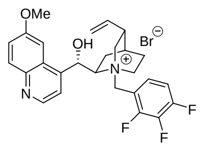
5

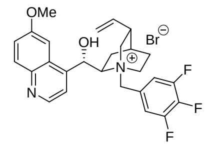
QD13

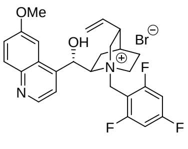
QD14

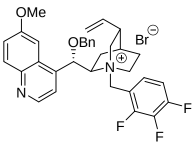
QD15

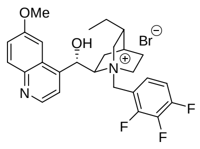
QD16

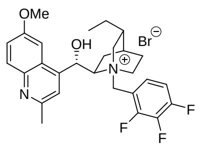
6

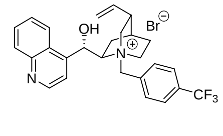
CN17

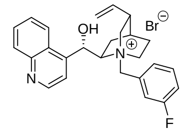
CN18

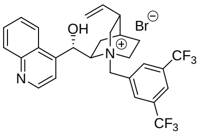
CN19

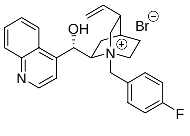
CN20

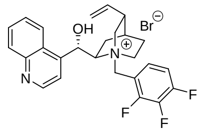
7

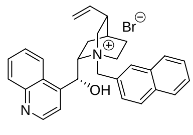
CN21

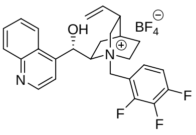
CN22

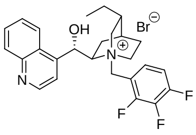
8

Figure 1. Catalysts used during optimisation studies

Entry	Cat	Base	Solvent	R-X (eq)	Time	*s*	Conv	Starting material e.r.	Product e.r.
1	**CN1**	KOH (25% aq, 5.0 eq)	$PhCH_3$ (0.1 M)	BnBr (0.6)	18h	1.1	55%	52.5:47.5	52:48
2	**CD2**	KOH (25% aq, 5.0 eq)	$PhCH_3$ (0.1 M)	BnBr (0.6)	18h	1.1	50%	51.5:48.5	51.5:48.5
3	**3**	KOH (25% aq, 5.0 eq)	$PhCH_3$ (0.1 M)	BnBr (0.6)	18h	1.2	42%	52.5:47.5	52.5:47.5
4	**QN3**	KOH (25% aq, 5.0 eq)	$PhCH_3$ (0.1 M)	BnBr (0.6)	18h	1.1	50%	51:49	51:49
5	**3**	NaOH (25% aq, 5.0 eq)	$PhCH_3$ (0.1 M)	BnBr (0.6)	18h	1.2	36%	52:48	53.5:46.5
6	**3**	RbOH (25% aq, 5.0 eq)	$PhCH_3$ (0.1 M)	BnBr (0.6)	18h	1.3	35%	55.5:44.5	51.5:48.5
7	**3**	CsOH (25% aq, 5.0 eq)	$PhCH_3$ (0.1 M)	BnBr (0.6)	18h	1.2	42%	52.5:47.5	52.5:46.5
8	**3**	K_2CO_3 (50% aq, 5.0 eq)	$PhCH_3$ (0.1 M)	BnBr (0.6)	18h	2.7	12%	53:47	72:28
9	**3**	Cs_2CO_3 (50% aq, 5.0 eq)	$PhCH_3$ (0.1 M)	BnBr (0.6)	18h	2.5	13%	53:47	71:29
10	**3**	K_3PO_4 (50% aq, 5.0 eq)	$PhCH_3$ (0.1 M)	BnBr (0.6)	18h	3.1	13%	53:47	73.5:26.5
11	**QD4**	K_3PO_4 (50% aq, 5.0 eq)	$PhCH_3$ (0.1 M)	BnBr (0.6)	48h	9.3	24%	62:38	89:11
12	**QD5**	K_3PO_4 (50% aq, 5.0 eq)	$PhCH_3$ (0.1 M)	BnBr (0.6)	48h	2.3	16%	53.5:46.5	68.5:31.5
13	**QD6**	K_3PO_4 (50% aq, 5.0 eq)	$PhCH_3$ (0.1 M)	BnBr (0.6)	48h	1.9	18%	53:47	64:36
14	**QD7**	K_3PO_4 (50% aq, 5.0 eq)	$PhCH_3$ (0.1 M)	BnBr (0.6)	48h	4.8	3%	51:49	79.5:20.5
15	**QD8**	K_3PO_4 (50% aq, 5.0 eq)	$PhCH_3$ (0.1 M)	BnBr (0.6)	48h	2.8	10%	52.5:47.5	73.5:26.5
16	**QD9**	K_3PO_4 (50% aq, 5.0 eq)	$PhCH_3$ (0.1 M)	BnBr (0.6)	48h	3.0	2%	51:49	74.5:25.5
17	**QD10**	K_3PO_4 (50% aq, 5.0 eq)	$PhCH_3$ (0.1 M)	BnBr (0.6)	48h	2.9	4%	51:49	76:24
18	**QD11**	K_3PO_4 (50% aq, 5.0 eq)	$PhCH_3$ (0.1 M)	BnBr (0.6)	48h	5.3	29%	62.5:37.5	80:20
19	**QD12**	K_3PO_4 (50% aq, 5.0 eq)	$PhCH_3$ (0.1 M)	BnBr (0.6)	48h	7.3	13%	55.5:45.5	88:12
20	**4**	K_3PO_4 (50% aq, 5.0 eq)	$PhCH_3$ (0.1 M)	BnBr (0.6)	48h	13	27%	65:35	90:10
21	**5**	K_3PO_4 (50% aq, 5.0 eq)	$PhCH_3$ (0.1 M)	BnBr (0.6)	48h	7.6	5%	52:48	91:9
22	**QD13**	K_3PO_4 (50% aq, 5.0 eq)	$PhCH_3$ (0.1 M)	BnBr (0.6)	48h	5.6	13%	55:45	83:17

Entry	Cat	Base	Solvent	R-X (eq)	Time	*s*	Conv	Starting material e.r.	Product e.r.
23	**QD14**	K_3PO_4 (50% aq, 5.0 eq)	$PhCH_3$ (0.1 M)	BnBr (0.6)	48h	7.4	14%	56:44	87:13
24	**QD15**	K_3PO_4 (50% aq, 5.0 eq)	$PhCH_3$ (0.1 M)	BnBr (0.6)	48h	N/A	N/A	50:50	51:49
25	**QD16**	K_3PO_4 (50% aq, 5.0 eq)	$PhCH_3$ (0.1 M)	BnBr (0.6)	48h	8.9	6%	52.5:47.5	90.5:9.5
26	**4**	K_3PO_4 (50% aq, 5.0 eq)	$PhCH_3$ (0.1 M)	Allyl bromide (0.6)	48h	7.2	13%	55:45	85:15
27	**4**	K_3PO_4 (50% aq, 5.0 eq)	$PhCH_3$ (0.1 M)	Allyl iodide (0.6)	48h	7.1	35%	68:32	83:17
28	**4**	K_3PO_4 (50% aq, 5.0 eq)	$PhCH_3$ (0.1 M)	MeI (0.6)	48h	3.5	19%	56:44	76:24
29	**4**	K_3PO_4 (50% aq, 5.0 eq)	$PhCH_3$ (0.1 M)	BnI (0.6)	48h	12	35%	71:29	89:11
30	**4**	K_3PO_4 (s, 2.0 eq)	$PhCH_3$ (0.1 M)	BnI (0.6)	48h	6.4	45%	74.5:25.5	80:20
31	**4**	K_3PO_4 (s, 5.0 eq)	$PhCH_3$ (0.1 M)	BnI (0.6)	48h	4.4	44%	70:30	75:25
32	**4**	K_3PO_4 (s, 10.0 eq)	$PhCH_3$ (0.1 M)	BnI (0.6)	48h	5.5	55%	81:29	75:25
33	**4**	Na_3PO_4 (s, 5.0 eq)	$PhCH_3$ (0.1 M)	BnI (0.6)	48h	8.5	12%	55:45	88.5:11.5
34	**4**	Na_3PO_4 (15% aq.)	$PhCH_3$ (0.1 M)	BnI (0.6)	48h	6.1	42%	71.5:28.5	80:20
35	**4**	Na_2CO_3 (s, 5.0 eq)	$PhCH_3$ (0.1 M)	BnI (0.6)	48h	N/A	N/A	50:50	75:25
36	**4**	Na_2CO_3 (25% aq, 5.0 eq)	$PhCH_3$ (0.1 M)	BnI (0.6)	48h	9.7	9%	54:46	90:10
37	**4**	K_2CO_3 (s, 5.0 eq)	$PhCH_3$ (0.1 M)	BnI (0.6)	48h	8.8	11%	55:45	89:11
38	**4**	K_2CO_3 (50% aq, 5.0 eq)	$PhCH_3$ (0.1 M)	BnI (0.6)	48h	12	12%	55.5:45.5	91.5:8.5
39	**4**	Cs_2CO_3 (s, 2.0 eq)	$PhCH_3$ (0.1 M)	BnI (0.6)	48h	1.7	39%	56.5:43.5	60:40
40	**4**	Cs_2CO_3 (50% aq, 5.0 eq)	$PhCH_3$ (0.1 M)	BnI (0.6)	48h	8.2	26%	62.5:37.5	86.5:13.5
41	**4**	NaOH (25% aq, 5.0 eq)	$PhCH_3$ (0.1 M)	BnI (0.6)	48h	2.7	57%	70:30	65:35
42	**4**	KOH (25% aq, 5.0 eq)	$PhCH_3$ (0.1 M)	BnI (0.6)	48h	3.0	63%	76:24	65.5:34.5
43	**4**	CsOH (25% aq, 5.0 eq)	$PhCH_3$ (0.1 M)	BnI (0.6)	48h	3.8	60%	78.5:21.5	69:31
44	**4**	RbOH (25% aq, 5.0 eq)	$PhCH_3$ (0.1 M)	BnI (0.6)	48h	6.1	56%	83.5:16.5	76:24

Entry	Cat	Base	Solvent	R-X (eq)	Time	*s*	Conv	Starting material e.r.	Product e.r.
46	**4**	K_3PO_4 (50% aq, 5.0 eq)	Et_2O (0.1 M)	BnI (0.6)	48h	7.7	34%	68:32	84.5:15.5
47	**4**	K_3PO_4 (50% aq, 5.0 eq)	^{t}BuOMe (0.1 M)	BnI (0.6)	48h	4.7	42%	69.5:30.5	76.5:23.5
48	**4**	K_3PO_4 (50% aq, 5.0 eq)	Mesityl oxide (0.1 M)	BnI (0.6)	48h	2.4	73%	76.5:23.5	60:40
49	**4**	K_3PO_4 (50% aq, 5.0 eq)	CH_2Cl_2 (0.1 M)	BnI (0.6)	48h	8.2	68%	97:3	72:28
50	**4**	K_3PO_4 (50% aq, 5.0 eq)	$CHCl_3$ (0.1 M)	BnI (0.6)	48h	10	57%	91:9	79.5:20.5
51	**4**	K_3PO_4 (50% aq, 5.0 eq)	PhH (0.1 M)	BnI (0.6)	48h	14	53%	90:10	85:15
52	**4**	K_3PO_4 (50% aq, 5.0 eq)	$PhCF_3$ (0.1 M)	BnI (0.6)	48h	2.4	43%	62:38	66:34
53	**4**	K_3PO_4 (50% aq, 5.0 eq)	m-Xylene (0.1 M)	BnI (0.6)	48h	15	10%	55:45	93:17
54	**4**	K_3PO_4 (50% aq, 5.0 eq)	p-Xylene (0.1 M)	BnI (0.6)	48h	13	7%	53:47	92.5:7.5
55	**4**	K_3PO_4 (50% aq, 5.0 eq)	1,2-Dichloro-ethane (0.1 M)	BnI (0.6)	48h	7.6	72%	98:2	69:31
56	**4**	K_3PO_4 (50% aq, 5.0 eq)	CCl_4 (0.1 M)	BnI (0.6)	48h	8.6	29%	65:35	86.5:13.5
57	**4**	K_3PO_4 (50% aq, 5.0 eq)	2-MeTHF (0.1 M)	BnI (0.6)	48h	9.7	34%	69:31	87:13
58	**4**	K_3PO_4 (50% aq, 5.0 eq)	$^i Pr_2O$ (0.1 M)	BnI (0.6)	48h	2.1	8%	51.5:48.5	67.5:32.5
59	**4**	K_3PO_4 (50% aq, 5.0 eq)	THF (0.1 M)	BnI (0.6)	48h	2.5	67%	74.5:25.5	62:38
60	**4**	K_3PO_4 (50% aq, 5.0 eq)	1,4-Dioxane (0.1 M)	BnI (0.6)	48h	6.6	60%	88:12	75:25
61	**4**	K_3PO_4 (s, 5.0 eq)	9:1 PhH/ $CHCl_3$ (0.1 M)	BnOTs (0.6)	72h	2.5	56%	68:32	64:36
62	**4**	K_2CO_3 (50% aq, 5.0 eq)	PhH (0.1 M)	BnOTs (0.6)	72h	8.5	48%	80:20	81:19
63	**4**	Cs_2CO_3 (50% aq, 5.0 eq)	PhH (0.1 M)	BnOTs (0.6)	72h	6.0	41%	80:20	71:29
64	**QD15**	K_2CO_3 (50% aq, 5.0 eq)	PhH (0.1 M)	BnOTs (0.6)	72h	15	55%	92:8	85:15
65	**QD12**	K_2CO_3 (50% aq, 5.0 eq)	PhH (0.1 M)	BnOTs (0.6)	72h	13.9	57%	94:6	83:17
66	**QD4**	K_2CO_3 (50% aq, 5.0 eq)	PhH (0.1 M)	BnOTs (0.6)	72h	5.8	43%	72:28	79:21

Entry	Cat	Base	Solvent	R-X (eq)	Time	*s*	Conv	Starting material e.r.	Product e.r.
67	**QD13**	K_2CO_3 (50% aq, 5.0 eq)	PhH (0.1 M)	BnOTs (0.6)	72h	3.4	50%	70.5:29.5	70:30
68	**QD14**	K_2CO_3 (50% aq, 5.0 eq)	PhH (0.1 M)	BnOTs (0.6)	72h	6.5	57%	85:15	76:24
69	**6**	K_2CO_3 (50% aq, 5.0 eq)	PhH (0.1 M)	BnOTs (0.6)	72h	21	54%	94.5:5.5	88:12
70	**CN17**	K_2CO_3 (50% aq, 5.0 eq)	PhH (0.1 M)	BnOTs (0.6)	48h	1.8	50%	60:40	60:40
71	**CN18**	K_2CO_3 (50% aq, 5.0 eq)	PhH (0.1 M)	BnOTs (0.6)	48h	3.9	44%	68.5:31.5	73.5:26.5
72	**CN19**	K_2CO_3 (50% aq, 5.0 eq)	PhH (0.1 M)	BnOTs (0.6)	48h	2.7	37%	61:39	68.5:31.5
73	**CN20**	K_2CO_3 (50% aq, 5.0 eq)	PhH (0.1 M)	BnOTs (0.6)	48h	2.4	47%	63.5:36.5	65.5:34.5
74	**7**	K_2CO_3 (50% aq, 5.0 eq)	PhH (0.1 M)	BnOTs (0.6)	48h	21	48%	87:13	91.5:9.5
75	**CN1**	K_2CO_3 (50% aq, 5.0 eq)	PhH (0.1 M)	BnOTs (0.6)	48h	2.9	51%	68:32	67:33
76	**CN21**	K_2CO_3 (50% aq, 5.0 eq)	PhH (0.1 M)	BnOTs (0.6)	48h	4.3	37%	65.5:34.5	76:24
77	**CN22**	K_2CO_3 (50% aq, 5.0 eq)	PhH (0.1 M)	BnOTs (0.6)	48h	17	44%	82:18	90:10
78	**QD16**	K_2CO_3 (50% aq, 5.0 eq)	PhH (0.1 M)	BnOTs (0.6)	72h	21	55%	94:6	88:12
79	**QD16**	K_2CO_3 (50% aq, 5.0 eq)	PhMe (0.1 M)	BnOTs (0.6)	72h	19	43%	81:19	90.5:9.5
80	**QD16**	K_2CO_3 (50% aq, 5.0 eq)	CH_2Cl_2 (0.1 M)	BnOTs (0.6)	72h	9	31%	66.5:33.5	86:14
81	**QD16**	K_2CO_3 (50% aq, 5.0 eq)	$CHCl_3$ (0.1 M)	BnOTs (0.6)	72h	10	45%	79:21	85:15
82	**QD16**	K_2CO_3 (50% aq, 5.0 eq)	m-Xylene (0.1 M)	BnOTs (0.6)	72h	19	44%	82:18	90.5:9.5
83	**QD16**	K_2CO_3 (50% aq, 5.0 eq)	p-Xylene (0.1 M)	BnOTs (0.6)	72h	22	47%	84:16	89:11
84	**QD16**	K_2CO_3 (50% aq, 5.0 eq)	$PhCF_3$ (0.1 M)	BnOTs (0.6)	72h	7.5	22%	60:40	86:14
85	**QD16**	K_2CO_3 (50% aq, 5.0 eq)	Et_2O (0.1 M)	BnOTs (0.6)	72h	12	49%	84:16	85:15
86	**QD16**	K_2CO_3 (50% aq, 5.0 eq)	$^{i}Pr_2O$ (0.1 M)	BnOTs (0.6)	72h	5.0	31%	64:36	80.5:19.5
87	**QD16**	K_2CO_3 (50% aq, 5.0 eq)	^{t}BuOMe (0.1 M)	BnOTs (0.6)	72h	11	47%	81:19	85:15
88	**QD16**	K_2CO_3 (50% aq, 5.0 eq)	PhH (0.2 M)	BnOTs (0.6)	72h	12	41%	79.5:20.5	87:13

Entry	Cat	Base	Solvent	R-X (eq)	Time	*s*	Conv	Starting material e.r.	Product e.r.
89	**QD16**	K_2CO_3 (50% aq, 5.0 eq)	PhH (0.05 M)	BnOTs (0.6)	72h	19	43%	81:19	90.5:9.5
90	**QD16**	K_2CO_3 (50% aq, 5.0 eq)	PhH (0.025 M)	BnOTs (0.6)	72h	25	44%	83.5:16.5	92:8
91	**CN22**	K_2CO_3 (50% aq, 5.0 eq)	PhH (0.025 M)	BnOTs (0.6)	72h	26	43%	83:17	93:7
92	**8**	K_2CO_3 (50% aq, 5.0 eq)	PhH (0.025 M)	BnOTs (0.6)	72h	32	46%	87:13	93.5:6.5
93	**8**	NaOH (25% aq, 2.0 eq)	PhH (0.025 M)	BnOTs (0.6)	24h	9.2	51%	83.5:16.5	82.5:17.5
94	**8**	KOH (25% aq, 2.0 eq)	PhH (0.025 M)	BnOTs (0.6)	24h	7.8	50%	81:19	81:19
95	**8**	CsOH (25% aq, 2.0 eq)	PhH (0.025 M)	BnOTs (0.6)	24h	19	49%	88:12	89:11
96	**8**	$KHCO_3$ (25% aq, 5.0 eq)	PhH (0.025 M)	BnOTs (0.6)	24h	33	4%	51.5:48.5	94:6
97	**8**	KF (25% aq, 5.0 eq)	PhH (0.025 M)	BnOTs (0.6)	24h	20	8%	54:46	95:5
98	**8**	CsF (25% aq, 5.0 eq)	PhH (0.025 M)	BnOTs (0.6)	24h	40	2%	51:49	97.5:2.5
99	**8**	K_2CO_3 (50% aq, 5.0 eq)	PhH (0.025 M)	BnOTs (1.0)	24h	32	53%	96.5:3.5	90.5:9.5
100	**8**	K_2CO_3 (50% aq, 5.0 eq)	PhH (0.025 M)	BnOTs (1.0)	24h	32	44%	84.5:15.5	94:6
101	**8**	Na_3PO_4 (s, 5.0 eq)	PhH (0.025 M)	BnOTs (0.6)	24h	30	28%	67.5:32.5	95.5:4.5
102	**8**	K_3PO_4 (50% aq, 5.0 eq)	PhH (0.025 M)	BnOTs (0.6)	24h	34	48%	90.5:9.5	93:7
103	**8**	Rb_2CO_3 (50% aq, 5.0 eq)	PhH (0.025 M)	BnOTs (0.6)	24h	29	40%	79.5:20.5	94:6
104	**8**	NaOH (25% aq, 2.0 eq)	PhH (0.025 M)	BnOTs (0.6)	48h	10	54%	87.5:12.5	82:18
105	**8**	KOH (25% aq, 2.0 eq)	PhH (0.025 M)	BnOTs (0.6)	48h	8.2	8%	85.5:14.5	80:20
106	**8**	CsOH (25% aq, 2.0 eq)	PhH (0.025 M)	BnOTs (0.6)	48h	20	55%	95:5	87:13
107	**8**	$KHCO_3$ (25% aq, 5.0 eq)	PhH (0.025 M)	BnOTs (0.6)	48h	27	4%	53:47	96.5:3.5
108	**8**	KF (25% aq, 5.0 eq)	PhH (0.025 M)	BnOTs (0.6)	48h	53	10%	55:45	98:2
109	**8**	CsF (25% aq, 5.0 eq)	PhH (0.025 M)	BnOTs (0.6)	48h	25	4%	52:48	96:4
110	**8**	K_2CO_3 (50% aq, 5.0 eq)	PhH (0.025 M)	BnOTs (1.0)	48h	25	58%	99:1	85.5:14.5

Entry	Cat	Base	Solvent	R-X (eq)	Time	*s*	Conv	Starting material e.r.	Product e.r.
111	**8**	K_2CO_3 (50% aq, 5.0 eq)	PhH (0.025 M)	BnOTs (1.0)	48h	32	51%	93:7	92:8
112	**8**	Na_3PO_4 (s, 5.0 eq)	PhH (0.025 M)	BnOTs (0.6)	48h	29	35%	74:26	94.5:5.5
113	**8**	K_3PO_4 (50% aq, 5.0 eq)	PhH (0.025 M)	BnOTs (0.6)	48h	31	54%	96.5:3.5	90.5:9.5
114	**8**	Rb_2CO_3 (50% aq, 5.0 eq)	PhH (0.025 M)	BnOTs (0.6)	48h	31	47%	88:12	93:7
115	**8**	K_2CO_3 (sat. aq, 5.0 eq)	PhH (0.025 M)	BnOTs (0.6)	48h	35	50%	93:7	93:7
116	**8**	K_2CO_3 (sat. aq, 5.0 eq)	PhH (0.025 M)	BnOTs (0.7)	48h	35	55%	98.5:1.5	89.5:10.5
117	**8**	K_2CO_3 (sat. aq, 5.0 eq)	PhH (0.025 M)	BnOTs (0.8)	48h	34	57%	99.5:0.5	87.5:12.5
118	**8**	K_2CO_3 (sat. aq, 5.0 eq)	PhH (0.025 M)	BnOTs (0.9)	48h	25	60%	99.5:0.5	83.5:16.5
119	**8**	K_2CO_3 (sat. aq, 5.0 eq)	PhH (0.025 M)	BnOTs (1.0)	48h	26	60%	>99.5:0.5	83:17
120	**8**	K_2CO_3 (sat. aq, 5.0 eq)	PhH (0.025 M)	BnOTs (1.5)	48h	13	69%	>99.5:0.5	72:28
121	**8**	K_2CO_3 (sat. aq, 5.0 eq)	9:1 PhH /CH_2Cl_2 (0.025 M)	BnOTs (0.6)	48h	31	45%	85.5:14.5	93.5:6.5
122	**8**	K_2CO_3 (sat. aq, 5.0 eq)	9:1 PhH /$CHCl_3$ (0.025 M)	BnOTs (0.6)	48h	31	46%	87:13	93:7
123	**8**	K_2CO_3 (sat. aq, 5.0 eq)	9:1 PhH /Et_2O (0.025 M)	BnOTs (0.6)	48h	34	48%	90:10	93.5:6.5
124	**8**	K_2CO_3 (s, 5.0 eq)	9:1 PhH /Et_2O (0.025 M)	BnOTs (0.6)	48h	34	46%	87.5:12.5	94:6
125	**8**	K_3PO_4 (sat. aq, 5.0 eq)	9:1 PhH /Et_2O (0.025 M)	BnOTs (0.6)	48h	32	46%	87.5:12.5	93.5:6.5
126	**8**	K_2CO_3 (sat. aq, 5.0 eq)	9:1 PhH /Et_2O (0.025 M)	BnOTs (1.0)	24h	36	49%	91.5:8.5	93.5:6.5
127	**8**	K_2CO_3 (sat. aq, 5.0 eq)	9:1 PhH /Et_2O (0.025 M)	BnOTs (1.5)	24h	35	54%	98:2	90:10

4. Synthesis of Racemic BINOLs

Racemic BINOLs were synthesised as shown below. Brominated 2-naphthols (see sub-sections for synthetic routes) were dimerised following a literature procedure.[3] 6,6'-dibromo BINOL (**54**) was prepared by bromination of racemic BINOL. Monomethylation gave the C_2 symmetric brominated methyl BINOLs. Debromination with ^{n}BuLi followed by aqueous quenching gave the non-C_2 symmetric bromination BINOLs. The C-Br bonds were then further functionalised through a variety of reactions.

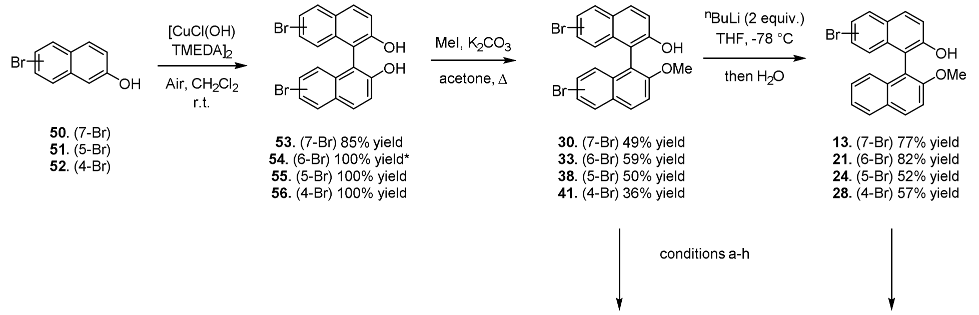

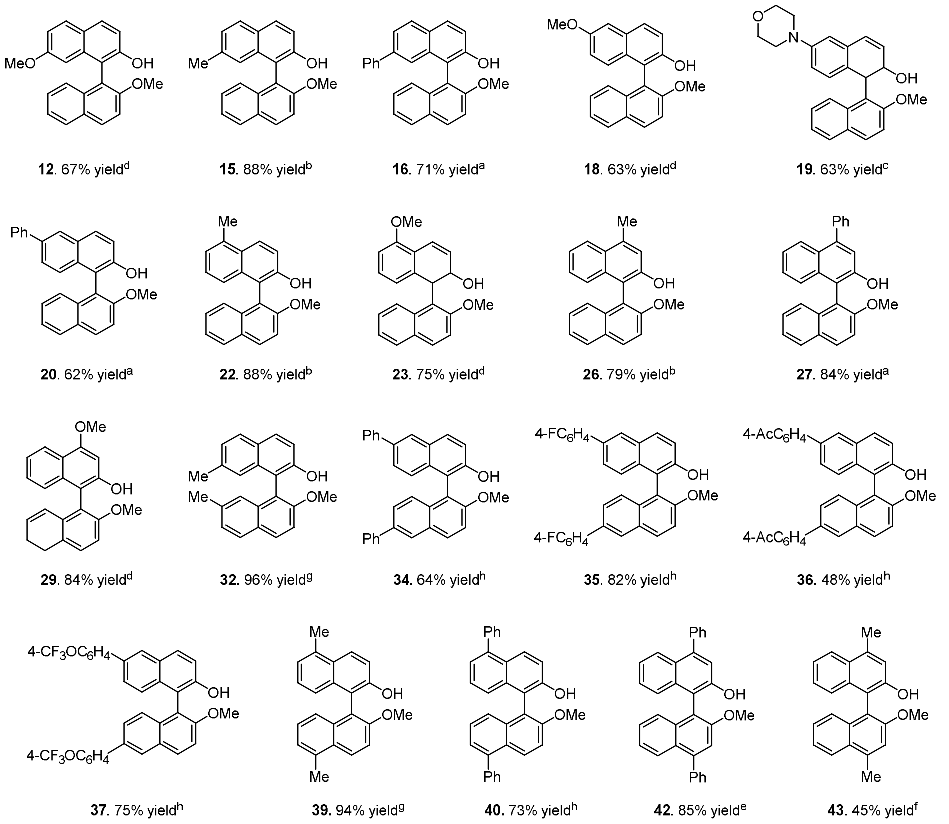

Synthesis of substituted BINOLs by C-Br bond transformation. Conditions: A: $PhB(OH)_2$ (1.20 equiv.), $Pd(PPh_3)_4$ (5 mol%), Na_2CO_3 (2.50 equiv.), dioxane-H_2O, 90 °C, 16 h. Conditions B: MeMgI (3.00 equiv.), $Pd(dppf)Cl_2$ (10 mol%), THF, Δ. Conditions C: morpholine, $Pd_2(dba)_3$ (1 mol%), DavePhos (1.2 mol%), LiHMDS (2.20 equiv.), THF. Conditions D: NaOMe (12.0 equiv.), CuCl (3.00 equiv.), DMF, 100 °C. Conditions E: $PhB(OH)_2$ (2.40 equiv.), $Pd(PPh_3)_4$ (5 mol%), Na_2CO_3 (5.00 equiv.), dioxane-H_2O, 90 °C, 16 h. Conditions F: MeMgI (10.0 equiv.), $Pd(dppf)Cl_2$ (10 mol%), THF, Δ. Conditions G: $MeB(OH)_2$ (4.0 equiv), $Pd(dppf)_2Cl_2 \cdot CH_2Cl_2$ (10 mol%), Cs_2CO_3 (4.0 equiv), 1,4-dioxane, Δ. Conditions H: $ArB(OH)_2$ (2.5 equiv), $Pd(PPh_3)_4$ (0.05 equiv), Na_2CO_2 (2 M aq, 5.0 equiv), DME, Δ.

4.1. 7-Substituted BINOLs

7-Bromonaphthalen-2-ol (50)

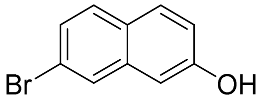

Following a modified literature procedure.[4] Bromine (6.14 mL, 19.1 g, 120 mmol, 1.20 equiv.) was added dropwise over 30 minutes to a suspension of PPh_3 (31.4 g, 120 mmol, 1.20 equiv.) in acetonitrile (50 mL) at 0 °C. The reaction was warmed to room temperature, 2,7-dihydroxynaphthalene (16.0 g, 100 mmol, 1.00 equiv.) was added and the reaction was heated at 90 °C for 1 hour. Acetonitrile was removed *in vacuo,* the flask was fitted with a gas trap filled with saturated Na_2SO_3 solution and the reaction was heated at 250 °C for 1 hour. The resulting mixture was dissolved in CH_2Cl_2 and filtered through a plug of silica and concentrated. Purification *via* flash column chromatography (50% CH_2Cl_2 – petroleum ether to 100% CH_2Cl_2) afforded 7-Bromonaphthalen-2-ol (**50**) (7.48 g, 30%) as a brown solid.

m.p. = 119-120 °C (CH_2Cl_2/petroleum ether 40-60).

IR (film) ν_{max}/cm^{-1}: 3366, 2923, 1652, 1577, 1558, 1506, 1473, 1457, 1253, 1209, 921, 626.

^{1}H NMR (500 MHz, $CDCl_3$) δ =7.87 (d, *J* = 1.68 Hz, 1H), 7.74 (d, *J* = 8.82 Hz, 1H), 7.65 (d, *J* = 8.66 Hz, 1H), 7.42 (dd, *J* = 8.71, 1.92 Hz), 7.13 (dd, *J* = 8.81, 2.49 Hz, 1H), 7.08 (d, *J* = 2.35 Hz), 5.05 (s, 1H).

^{13}C NMR (126 MHz, $CDCl_3$) δ = 154.1, 135.8, 129.9, 129.4, 128.3, 127.3, 127.0, 120.8, 118.2, 108.7.

HRMS (ESI^-) $C_{10}H_7{}^{79}BrO$ $[M-H]^-$ requires 220.9608; found 220.9607, Δ -0.5 ppm.

Data in agreement with literature reported[4]

7,7'-Dibromo-[1,1'-binaphthalene]-2,2'-diol (53)

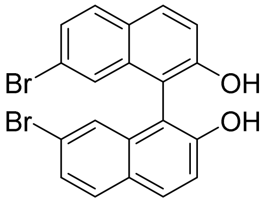

7-Bromonaphthalen-2-ol (**50**) (7.48 g, 33.5 mmol, 1.00 equiv.) was dissolved in CH_2Cl_2 (220 mL) and $[Cu(TMEDA)OHCl)]_2$ (155 mg, 0.0350 mmol, 1 mol%) was added. The reaction was stirred for 16 hours at room temperature open to air. The reaction mixture was filtered through a short silica plug eluting with CH_2Cl_2. Evaporation of the solvent gave 7,7'-dibromo-[1,1'-binaphthalene]-2,2'-diol (**53**) (6.28 g, 85%) as an off-white solid.

m.p. = 115-117 °C (CH_2Cl_2).

IR (film) ν_{max}/cm^{-1}: 3466, 1611, 1500, 1418, 1378, 1349, 1317, 1213, 1195, 1171, 1128, 836, 739.

^{1}H NMR (400 MHz, $CDCl_3$) δ = 7.94 (d, J = 8.9 Hz, 2H), 7.76 (d, J = 8.6 Hz, 2H), 7.47 (d, J = 8.7 Hz, 2H), 7.37 (d, J = 8.9 Hz, 2H), 7.23 (s, 2H), 5.04 (s, 2H).

^{13}C NMR (101 MHz, $CDCl_3$) δ = 153.6, 134.6, 131.7, 130.2, 127.9, 127.8, 125.9, 122.4, 118.3, 109.5.

HRMS (ESI$^-$) $C_{20}H_{12}Br_2O_2$ [M-H]$^-$ requires 440.91310, found: 440.91330, Δ +0.5 ppm.

Data in agreement with literature reported[4]

7,7'-Dibromo-2'-methoxy-[1,1'-binaphthalen]-2-ol (30)

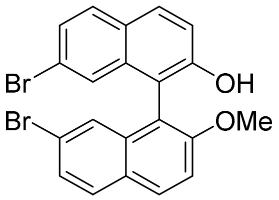

7,7'-dibromo-[1,1'-binaphthalene]-2,2'-diol (**53**) (6.38 g, 14.4 mmol, 1.00 equiv.) was dissolved in acetone (115 mL) and K_2CO_3 (2.58 g, 18.7 mmol, 1.30 equiv.) followed by iodomethane (984 µL, 2.24 g, 15.8 mmol, 1.10 equiv.) were added. The reaction was heated under reflux for 16 hours. Upon cooling to room temperature, the solvent was removed *in vacuo*. The residue was partitioned between water and CH_2Cl_2 and the phases were separated. The aqueous layer was extracted twice with CH_2Cl_2 and the organic layers were combined, dried over anhydrous sodium sulfate, filtered and concentrated to give the crude product. Purification *via* flash column chromatography (1:1 CH_2Cl_2 – petroleum ether 40-60) gave 7,7'-dibromo-2'-methoxy-[1,1'-binaphthalen]-2-ol (**30**) (3.21 g, 49%) as an off-white solid.

m.p. = 179-181 °C (CH_2Cl_2/petroleum ether 40-60).

IR (film) ν_{max}/cm^{-1}: 3503, 1612, 1497, 1255, 1088, 830, 754.

^{1}H NMR (500 MHz, $CDCl_3$) δ = 8.02 (d, J = 9.1 Hz, 1H), 7.87 (d, J = 8.9 Hz, 1H), 7.76 (d, J = 8.7 Hz, 1H), 7.73 (d, J = 8.7 Hz, 1H), 7.47 (d, J = 9.1 Hz, 1H), 7.46 (dd, J = 8.7, 1.9 Hz, 1H), 7.40 (dd, J = 8.6, 1.9 Hz, 1H), 7.35 (d, J = 8.9 Hz, 1H), 7.28 (d, J = 1.9 Hz, 1H), 7.15 (d, J = 1.9 Hz, 1H), 4.88 (s, 1H), 3.81 (s, 3H).

^{13}C NMR (127 MHz, $CDCl_3$) δ = 156.9, 152.1, 135.3, 135.1, 131.6, 130.2, 130.1, 130.1, 128.0, 127.9, 127.8, 127.0, 126.7, 126.5, 122.5, 121.4, 118.1, 114.0, 113.8, 113.7, 56.7

HRMS (ESI$^-$) $C_{21}H_{14}{}^{79}Br_2O_2$ $[M-H]^-$: requires 454.92878, found: 454.92905 Δ +0.6 ppm.

2'-methoxy-7,7'-dimethyl-[1,1'-binaphthalen]-2-ol (32)

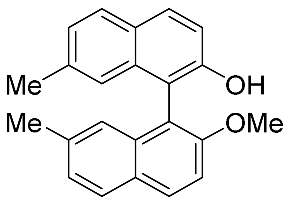

To a flask containing 7,7'-dibromo-2'-methoxy-[1,1'-binaphthalen]-2-ol (**30**) (200 mg, 0.437 mmol, 1.00 equiv.), Pd(dppf)Cl_2·CH_2Cl_2 (35.6 mg, 0.044 mmol, 0.10 equiv.), cesium carbonate (569 mg, 1.75 mmol, 4.00 equiv.) and methylboronic acid (104 mg, 1.75 mmol, 4.00 equiv.), was added 1,4-dioxane (4.50 mL, N_2 sparged). The resulting suspension was heated at 105 °C for 48 h. The mixture was cooled to room temperature, diluted with EtOAc (10.0 mL) and filtered through a short plug of silica (eluting with EtOAc). The resulting solution was concentrated *in vacuo* and purified by flash column chromatography (2:1 petroleum ether 40-60 – diethyl ether) to give 2'-methoxy-7,7'-dimethyl-[1,1'-binaphthalen]-2-ol (**32**) as an off-white powder (138 mg, 96%)

m.p. = 75-78 °C (CH_2Cl_2/petroleum ether 40-60)

IR (film) ν_{max}/cm^{-1}: 3489, 3431, 3046, 3012, 2917, 2848, 1624, 1510, 1458, 1360, 1322, 1263, 1249, 1216, 1182, 1170, 1152, 1085, 831, 756.

^{1}H NMR (500 MHz, $CDCl_3$) δ_H: 8.01 (d, *J* = 9.1 Hz, 1H), 7.85 (d, *J* = 8.9 Hz, 1H), 7.80 (d, *J* = 8.3 Hz, 1H), 7.76 (d, *J* = 8.3 Hz, 1H), 7.41 (d, *J* = 9.1 Hz), 7.28 (d, *J* = 8.8 Hz, 1H), 7.21 (dd, *J* = 8.3, 1.49 Hz, 1H), 7.15 (dd, *J* = 8.3, 1.5 Hz, 1H), 6.95 (d, *J* = 0.4 Hz, 1H), 6.82 (d, *J* = 0.5 Hz, 1H), 4.81 (s, 1H), 3.78 (s, 3H), 2.27 (s, 3H), 2.25 (s, 3H)

^{13}C NMR (126 MHz, $CDCl_3$) δ_C: 156.4, 151.3, 137.4, 136.2, 134.4, 134.1, 130.8, 129.5, 128.1, 128.1, 127.9, 127.5, 126.7, 125.7, 124.0, 123.8, 116.5, 114.9, 114.8, 113.0, 56.8, 22.1, 22.1

HRMS (ESI$^+$) $C_{23}H_{21}O_2$ [M+H]$^+$ requires 329.15361; found 329.15366, Δ +0.2 ppm.

7-Bromo-2'-methoxy-[1,1'-binaphthalen]-2-ol (13)

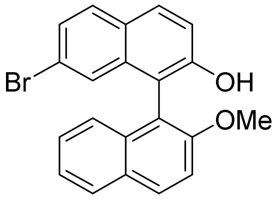

7,7'-Dibromo-2'-methoxy-[1,1'-binaphthalen]-2-ol (**30**) (2.52 g, 5.50 mmol, 1.00 equiv.) was dissolved in THF (55 mL) and cooled to −78 °C. ^{n}BuLi (1.6 M in hexane, 6.87 mL, 11.0 mmol, 2.00 equiv.) was added by syringe pump over 1 hour after which time the reaction was stirred for a further 1 hour at −78 °C. The reaction was quenched by dropwise addition of water. After warming to room temperature, saturated ammonium chloride solution was added. EtOAc was added and the phases were separated. The aqueous phase was extracted twice with EtOAc and the combined organic layers were washed with brine, dried over anhydrous sodium sulfate, filtered and concentrated to give the crude product which was purified by column chromatography (20% EtOAc-petroleum ether 40-60) to give 7-bromo-2'-methoxy-[1,1'-binaphthalen]-2-ol (**13**) (1.54 g, 77%)

m.p. = 171-172 °C. (EtOAc/petroleum ether 40-60)

IR (film) ν_{max}/cm^{-1}: 3496, 1612, 1501, 1429, 1352, 1250, 1168, 828, 809, 754.

^{1}H NMR ($CDCl_3$, 400 MHz) δ = 8.08 (d, *J* = 9.1 Hz, 1H), 7.91 (d, *J* = 8.1 Hz, 1H), 7.86 (d, *J* = 9.0 Hz, 1H), 7.72 (d, *J* = 8.6 Hz, 1H), 7.49 (d, *J* = 9.1 Hz, 1H), 7.42–7.37 (m, 2H, 1), 7.35 (d, *J* = 8.9 Hz, 1H), 7.32 (ddd, *J* = 8.2, 6.8, 1.4 Hz, 1H), 7.19 (d, *J* = 1.8 Hz, 1H), 7.15 (dd, *J* = 8.4, 1.1 Hz, 1H), 4.93 (s, 1H), 3.82 (s, 4H).

^{13}C NMR ($CDCl_3$, 101 MHz) δ = 156.2, 152.2, 135.2, 133.9, 131.6, 129.9, 129.9, 129.6, 128.4, 127.7, 127.0, 126.8, 124.6, 124.4, 121.2, 118.0, 114.6, 114.3, 113.8, 56.7.

HRMS (ESI^{-}) $C_{21}H_{15}O_2{}^{79}Br$ [M−H]$^{-}$ requires 377.01827; found 377.01807, Δ −0.5 ppm.

2',7-dimethoxy-[1,1'-binaphthalen]-2-ol (14)

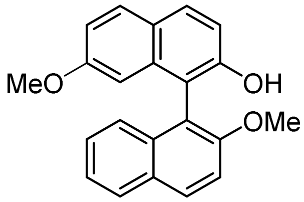

7-Bromo-2'-methoxy-[1,1'-binaphthalen]-2-ol (**13**) (170 mg, 0.448 mmol, 1.00 equiv.) was dissolved in DMF (4.5 mL) then CuCl (133 mg, 1.34 mmol 3.00 equiv.) and NaOMe (4.4 M in MeOH, 1.22 mL, 5.38 mmol, 12.0 equiv.) were added sequentially. The reaction was heated at 100 °C for 16 h then cooled to room temperature. Ice water was added followed by hydrochloric acid (3 M). The resulting suspension was extracted 3 times with EtOAc and the combined organic layers were washed with brine, dried over anhydrous sodium sulfate, filtered and concentrated to give the crude product. Purification *via* flash column chromatography (3:1 CH_2Cl_2 – petroleum ether 40-60 to 9:1 CH_2Cl_2 – petroleum ether 40-60) afforded 2',7-dimethoxy-[1,1'-binaphthalen]-2-ol (**14**) as an off-white solid (99.7 mg, 67%).

m.p. = 137-139 °C (CH_2Cl_2/ petroleum ether 40-60).

IR (film) ν_{max}/cm^{-1}: 3495, 3427, 3058, 3007, 2936, 28238, 1621, 1592, 1511, 1464, 1430, 1368, 1331, 1264, 1248, 1221, 1199, 1166, 1148, 1082, 833, 815, 752.

^{1}H NMR (500 MHz, $CDCl_3$) *δ* = 8.05 (d, *J* = 9.1 Hz, 1H), 7.90 (d, *J* = 8.1 Hz, 1H), 7.83 (d, *J* = 8.8 Hz, 1H), 7.77 (d, *J* = 8.9 Hz, 1H), 7.48 (d, *J* = 9.1 Hz, 1H), 7.38 (ddd, *J* = 1.2, 6.8, 8.1 Hz, 1H), 7.30 (ddd, *J* = 1.3, 6.8, 8.4 Hz, 1H), 7.25-7.20 (m, 2H), 6.99 (dd, *J* = 2.5, 8.9 Hz, 1H), 6.37 (d, *J* = 2.5 Hz, 1H), 4.92 (s, 1H), 3.82 (s, 3H), 3.51 (s, 3H).

^{13}C NMR (126 MHz, $CDCl_3$) *δ* = 158.3, 156.0, 151.9, 135.2, 133.9, 131.2, 129.8, 129.6, 129.6, 128.2, 127.4, 125.0, 124.7, 124.3, 115.5, 115.2, 115.1, 114.3, 113.9, 104.3, 56.8, 55.1.

HRMS (ESI^{+}) $C_{22}H_{19}O_3^{+}$ $[M+H]^{+}$: requires: 331.13287, found: 331.13297, Δ +0.3 ppm.

2'-Methoxy-7-methyl-[1,1'-binaphthalen]-2-ol (15)

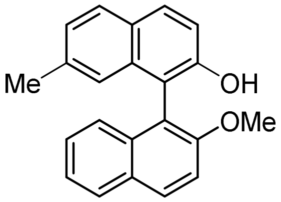

7-Bromo-2'-methoxy-[1,1'-binaphthalen]-2-ol (**13**) (443 mg, 1.20 mmol, 1.00 equiv.) and Pd(dppf)Cl_2·CH_2Cl_2 (98 mg, 0.12 mmol, 10 mol%) were dissolved in THF (15 mL) and MeMgCl solution (3 M in Et_2O, 2 mL, 6.00 mmol, 5.00 equiv.) was added. The resulting solution was heated under reflux for 16 hours. On cooling to room temperature, saturated ammonium chloride solution was added and phases were separated. The aqueous phase was extracted twice with EtOAc and the combined organic layers were washed with brine, dried over anhydrous sodium sulfate, filtered and concentrated to give the crude product which was purified by column chromatography (9:1 petroleum ether 40-60 – EtOAc) to give 2'-methoxy-7-methyl-[1,1'-binaphthalen]-2-ol (**15**) (334 mg, 88%) as an off-white solid.

m.p. = 87-88 °C (EtOAc/petroleum ether 40-60).

IR (film) ν_{max}/cm^{-1}: 3481, 3396, 1704, 1621, 1591, 1508, 1473, 1459, 1431, 1378, 1362, 1332, 1302, 1265, 1248, 1220, 1184, 1172, 1146, 1129, 1083, 1053, 1041, 1020, 977, 908, 859, 833, 811, 775, 747, 676, 629.

^{1}H NMR (400 MHz, $CDCl_3$) δ = 8.06 (1H, d, *J* = 9.0 Hz), 7.92 (1H, d, *J* = 8.1 Hz), 7.87 (1H, d, *J* = 8.9 Hz), 7.78 (1H, d, *J* = 8.3 Hz), 7.50 (1H, d, *J* = 9.1 Hz), 7.40 (1H, ddd, *J* = 8.1, 6.6, 1.3 Hz), 7.35–7.28 (2H, m), 7.24–7.15 (2H, m), 6.84 (1H, s), 4.90 (1H, s), 3.82 (3H, s), 2.27 (3H, s).

^{13}C NMR (101 MHz, $CDCl_3$) δ = 156.0, 151.3, 136.2, 134.1, 134.0, 131.0, 129.5, 129.5, 128.1, 128.0, 127.4, 127.3, 125.6, 125.0, 124.2, 123.8, 116.5, 115.6, 114.5, 113.9, 56.7, 29.8.

HRMS (ESI^-) $C_{22}H_{18}O_2$ $[M-H]^-$ requires 313.12340; found 313.12326, Δ -0.5 ppm.

2'-Methoxy-7-phenyl-[1,1'-binaphthalen]-2-ol (16)

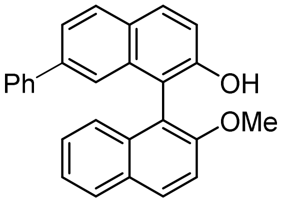

7-Bromo-2'-methoxy-[1,1'-binaphthalen]-2-ol (**13**) (548 mg, 1.50 mmol, 1.00 equiv.), phenyl boronic acid (220 mg, 1.80 mmol, 1.20 equiv.) and $Pd(PPh_3)_4$ (86.7 mg, 0.0750 mmol, 5 mol%) were charged to a flask fitted with a water-cooled condenser. The flask was evacuated and backfilled with argon three times. 1,2-Dimethoxyethane (Argon sparged, 15 mL) was added followed by aqueous Na_2CO_3 solution (Argon sparged, 2 M, 1.87 mL, 2.50 equiv.). The flask was fitted with an argon balloon and heated at 90 °C for 16 hours. After cooling to room temperature, 1 M HCl solution was added and the solution was extracted three times with CH_2Cl_2, the combined organic layers were dried over anhydrous sodium sulfate, filtered and concentrated to give the crude product which was purified by column chromatography (9:1 petroleum ether 40-60 – EtOAc) to give 2'-methoxy-7-phenyl-[1,1'-binaphthalen]-2-ol 16) (410 mg, 71%) as a white solid.

m.p. = 86-87 °C (EtOAc/petroleum ether 40-60).

IR (film) ν_{max}/cm^{-1}: 3489, 3055, 2935, 2837, 2361, 1619, 1591, 1507, 1456, 1377, 1264, 1247, 1166, 1131, 1055, 839, 811, 752, 697.

^{1}H NMR (400 MHz, $CDCl_3$) δ_H =7.93 (d, J = 9.0 Hz, 1H), 7.86–7.82 (m, 2H), 7.79 (dt, J = 8.3, 1.0 Hz, 2H), 7.48 (dd, J = 8.5, 1.8 Hz, 2H), 7.37 (d, J = 9.1 Hz, 2H), 7.31–7.24 (m, 4H), 7.23–7.10 (m, 6H), 3.70 (s, 3H).

^{13}C NMR (101 MHz, $CDCl_3$) δ_C = 156.0, 151.7, 141.6, 139.2, 134.1, 134.0, 131.2, 129.6, 129.5, 128.7, 128.6, 128.4, 128.3, 127.5, 127.4, 127.1, 124.9, 124.3, 123.2, 123.0, 117.6, 115.4, 115.2, 113.8, 56.7.

HRMS (ESI$^+$) $C_{27}H_{20}O_2$ $[M+H]^+$ requires 377.15361; found 377.15363, Δ +0.1 ppm.

4.2. 6-Substituted BINOLs

6,6'-Dibromo-[1,1'-binaphthalene]-2,2'-diol (54)

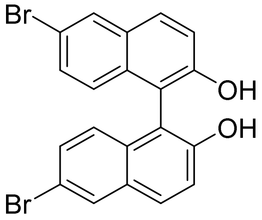

(±)-BINOL (14.3 g, 50.0 mmol, 1.00 equiv.) was dissolved in acetonitrile (250 mL) and the resulting solution cooled to 0 °C. Bromine (7.70 mL, 24.0 g, 150 mmol, 3.00 equiv.) was added dropwise over 10 minutes. The reaction was stirred for 3 hours at 0 °C after which time saturated Na_2SO_3 solution was added. The solution was extracted three times with CH_2Cl_2 and the combined organic layers were dried over sodium sulfate, filtered and concentrated to give 6,6'-dibromo-[1,1'-binaphthalene]-2,2'-diol (**54**) (22.2 g, 100%) as an off-white solid.

m.p. = 115-117 °C (CH_2Cl_2).

^{1}H NMR (500 MHz, $CDCl_3$) δ = 8.05 (d, J = 2.0 Hz, 2H), 7.90 (d, J = 9.0 Hz, 2H), 7.40 (d, J = 9.0 Hz, 2H), 7.37 (dd, J = 2.0, 9.0 Hz, 2H), 6.96 (d, J = 9.0 Hz, 2H), 5.04 (s, 2H).

^{13}C NMR (127 MHz, $CDCl_3$) δ = 153.1, 132.0, 131.0, 130.9, 130.7, 130.6, 126.0, 119.1, 118.2, 110.8.

Data in agreement with literature reported.[5]

6,6'-Dibromo-2'-methoxy-[1,1'-binaphthalen]-2-ol (33)

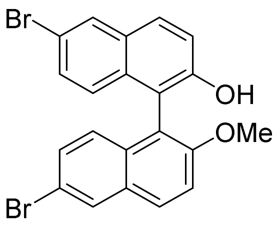

6,6'-Dibromo-[1,1'-binaphthalene]-2,2'-diol (**54**) (22.20 g, 50.0 mmol, 1.00 equiv.) and K_2CO_3 (8.97 g, 55.0 mmol, 1.10 equiv.) were suspended in acetone (400 mL) and iodomethane (7.80 g, 3.43 mL, 65.0 mmol, 1.30 equiv) was added. The resulting suspension was heated under reflux for 16 hours after which time the reaction was cooled to room temperature. The solvent was removed by evaporation and the residue was partitioned between water and CH_2Cl_2. The phases were separated and the aqueous layer was extracted twice with CH_2Cl_2. The combined organic layers were dried over anhydrous sodium sulfate, filtered and concentrated to give the crude product. Purification *via* flash column chromatography (1:1 CH_2Cl_2 – petroleum ether) afforded 6,6'-dibromo-2'-methoxy-[1,1'-binaphthalen]-2-ol (**30**) (13.5 g, 59%) as a white solid.

m.p. = 115-117 °C (CH_2Cl_2/petroleum ether 40-60).

IR (film) ν_{max}/cm^{-1}: 3480, 3059, 2935, 2837, 2361, 2341, 1614, 1583, 1493, 1459, 1440, 1418, 1381 1347, 1325, 1303, 1262, 1246, 1210, 1195, 1171, 1146, 1133, 1087, 1068, 1046, 976, 939, 904, 876, 808, 770, 738, 703, 672, 611.

^{1}H NMR (400 MHz, CDCl3) δ 8.05 (s, 1H), 8.01 (s, 1H), 7.96 (d, J = 9.1 Hz, 1H), 7.81 (d, J = 8.9 Hz, 1H), 7.49 (d, J = 9.1 Hz, 1H), 7.39-7.24 (m, 2H), 7.00 (d, J = 9.0 Hz, 1H), 6.86 (d, J = 8.9 Hz, 1H), 4.90 (s, 1H), 3.81 (s, 3H).

^{13}C NMR (101 MHz, $CDCl_3$) δ = 156.2, 151.7, 132.5, 132.3, 130.9, 130.5, 130.4, 130.3, 130.2, 129.9, 129.2, 126.6, 126.6, 118.8, 118.2, 117.3, 115.0, 114.8, 114.7, 56.7.

Data in agreement with literature reported[6]

2'-Methoxy-6,6'-diphenyl-[1,1'-binaphthalen]-2-ol (4)

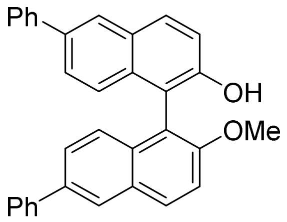

To a flask containing **33** (500 mg, 1.09 mmol, 1.00 equiv), phenylboronic acid (333 mg, 2.73 mmol, 2.50 equiv) and $Pd(PPh_3)_4$ (63.1 mg, 54.6 µmol, 0.05 equiv) was added 1,2-dimethoxyethane (11.50 mL, Ar sparged) and sodium carbonate(2.0 M aq, 2.72 mL, Ar sparged). The resulting mixture was heated to 90 °C for 16 hours and cooled to room temperature before addition of water (10 mL) and EtOAc (10 mL). The layers were separated and the aqueous layer was extracted with EtOAc (10 mL × 2). The combined organic layers were washed with brine, dried over anhydrous $MgSO_4$, filtered and the solvent removed under reduced pressure. The resulting solid was purified by flash column chromatography (2:1 CH_2Cl_2 – petroleum ether 40-60) to give the title compound **34** as a white solid (316 mg, 0.70 mmol, 64% yield).

m.p. = 192-194 °C (CH_2Cl_2/petroleum ether 40-60).

IR (film) ν_{max}/cm^{-1}: 3531, 1595, 1493, 1383, 1357, 1264, 1191, 1087, 810, 756, 699.

^{1}H NMR (500 MHz, $CDCl_3$) δ_H: 8.15-8.11 (m, 2H), 8.10 (d, J = 1.7 Hz, 1H), 7.99 (d, J = 8.9 Hz, 1H), 7.71-7.67 (m, 4H), 7.59 (dd, J = 1.8, 8.8 Hz, 1H), 7.53 (d, J = 9.1 Hz, 1H), 7.53 (dd, J = 1.9, 8.7 Hz, 1H), 7.50-7.44 (m, 4H), 7.42 (d, J = 8.9 Hz, 1H), 7.40-7.33 (m, 1H), 7.31 (d, J = 8.8 Hz, 1H), 7.18 (d, J = 8.7 Hz, 1H), 5.01 (s, 1H), 3.85 (s, 3H).

^{13}C NMR (126 MHz, $CDCl_3$) δ_C: 156.2, 151.6, 141.3, 140.9, 137.1, 136.2, 133.3, 133.1, 131.6, 130.3, 129.8, 129.5, 129.0, 128.9, 127.4, 127.3, 127.2, 127.1, 127.0, 126.3, 126.2, 125.6, 125.5, 118.1, 115.3, 115.0, 114.3, 56.8.

HRMS (ESI^-) $C_{33}H_{23}O_2^-$ $[M-H]^-$: requires 451.17035, found: 451.17053, Δ +0.4 ppm.

6,6'-Bis(4-fluorophenyl)-2'-methoxy-[1,1'-binaphthalen]-2-ol (6)

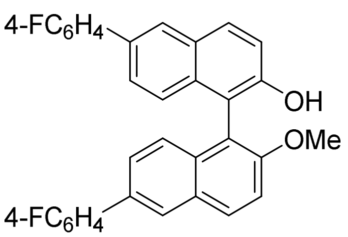

To a flask containing **3** (500 mg, 1.09 mmol, 1.00 equiv), (4-fluorophenyl)boronic acid (382 mg, 2.73 mmol, 2.50 equiv) and $Pd(PPh_3)_4$ (63.1 mg, 54.6 µmol, 0.05 equiv) was added 1,2-dimethoxyethane (11.50 mL, Ar sparged) and Na_2CO_3 (2.0 M aq, 2.72 mL, Ar sparged). The resulting mixture was heated to 90 °C for 16 hours and cooled to room temperature before addition of water (10 mL) and EtOAc (10 mL). The layers were separated and the aqueous layer was extracted with EtOAc (10 mL × 2). The combined organic layers were washed with brine, dried over anhydrous $MgSO_4$, filtered and the solvent removed under reduced pressure. The resulting solid was purified by flash column chromatography (2:1 CH_2Cl_2 – petroleum ether 40-60) to give the title compound **6** as a white solid (436 mg, 0.89 mmol, 82% yield).

m.p. = 145-147 °C (CH_2Cl_2/petroleum ether 40-60).

IR (film) ν_{max}/cm^{-1}: 3525, 1604, 1515, 1473, 1265, 1159, 1087, 820.

^{1}H NMR (500 MHz, $CDCl_3$) δ_H: 8.12 (d, J = 9.1 Hz, 1H), 8.06 (d, J = 1.8 Hz, 1H), 8.02 (d, J = 1.8 Hz, 1H), 7.97 (d, J = 8.9 Hz, 1H), 7.65-7.59 (m, 4H), 7.54 (d, J = 9.1 Hz, 1H), 7.51 (dd, J = 1.9, 8.8 Hz, 1H), 7.45 (dd, J = 1.9, 8.7 Hz, 1H), 7.40 (d, J = 8.9 Hz, 1H), 7.28 (d, J = 8.8 Hz, 1H), 7.18-7.10 (m, 5H), 4.97 (s, 1H), 3.85 (s, 3H).

^{13}C NMR (126 MHz, $CDCl_3$) δ_C: 162.6 (d, J = 246.7 Hz), 162.5 (d, J = 246.0 Hz), 156.3, 151.6, 137.4 (d, J = 3.4 Hz), 137.0 (d, J = 3.4 Hz), 136.1, 135.2, 133.3, 133.0, 131.5, 130.3, 129.8, 129.5, 128.9 (app. t, J = 7.6 Hz), 127.1, 126.1, 126.0, 125.7, 125.6, 118.2, 116.0, 115.9, 115.8, 115.7, 115.2, 115.0, 114.4, 56.9.

^{19}F NMR (470 MHz, $CDCl_3$, ^{1}H decoupled) δ_F: -115.6 (s), -116.1 (s).

HRMS (ESI$^-$) $C_{33}H_{21}F_2O_4^-$ [M-H]$^-$: requires 487.15151, found: 487.15134, Δ -0.4 ppm.

1,1'-((2-Hydroxy-2'-methoxy-[1,1'-binaphthalene]-6,6'-diyl)bis(4,1-phenylene))bis(ethan-1-one) (36)

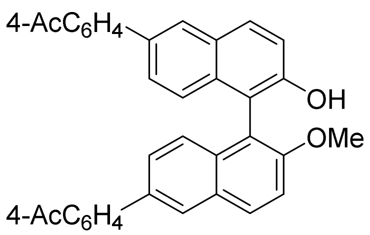

To a flask containing **33** (500 mg, 1.09 mmol, 1.00 equiv), (4-acetylphenyl)boronic acid (447 mg, 2.73 mmol, 2.50 equiv) and $Pd(PPh_3)_4$ (63.1 mg, 54.6 µmol, 0.05 equiv) was added 1,2-dimethoxyethane (11.50 mL, Ar sparged) and Na_2CO_3 (2.0 M aq, 2.72 mL, Ar sparged). The resulting mixture was heated to 90 °C for 16 hours and cooled to room temperature before addition of water (10 mL) and EtOAc (10 mL). The layers were separated and the aqueous layer was extracted with EtOAc (10 mL × 2). The combined organic layers were washed with brine, dried over anhydrous $MgSO_4$, filtered and the solvent removed under reduced pressure. The resulting solid was purified by flash column chromatography (19:1 CH_2Cl_2 – diethyl ether to 9:1 CH_2Cl_2 – diethyl ether) to give the title compound **36** as a pale yellow solid (280 mg, 0.52 mmol, 48% yield).

m.p. = 161-163 °C (CH_2Cl_2/diethyl ether).

IR (film) ν_{max}/cm^{-1}: 3340, 1674, 1598, 1497, 1358, 1268, 1249, 1057, 818, 751.

^{1}H NMR (500 MHz, $CDCl_3$) δ_H: 8.15 (app. d, J = 8.8 Hz, 3H), 8.03 (app. d, J = 8.2 Hz, 4H), 8.00 (d, J = 8.9 Hz, 1H), 7.76 (app. dd, J = 1.8, 8.4 Hz, 4H), 7.58 (dd, J = 1.8, 8.9 Hz, 1H), 7.55 (d, J = 9.3 Hz, 1H), 7.52 (dd, J = 1.9, 8.8 Hz, 1H), 7.43 (d, J = 8.9 Hz, 1H), 7.31 (d, J = 8.8 Hz, 1H), 7.17 (d, J = 8.8 Hz, 1H), 5.18 (s, 1H), 3.86 (s, 3H), 2.63 (s, 6H).

^{13}C NMR (126 MHz, $CDCl_3$) δ_C: 197.9, 197.9, 156.6, 152.1, 145.9, 145.4, 135.9, 135.7, 135.6, 134.7, 133.8, 133.6, 131.8, 130.6, 129.6, 129.4, 129.2, 129.1, 127.3, 127.3, 126.9, 126.8, 126.8, 125.9, 125.8, 125.7, 118.5, 115.2, 115.0, 114.5, 56.8, 26.8.

HRMS (ESI^+) $C_{37}H_{29}O_4^+$ $[M+H]^+$: requires: 537.20604, found: 537.20612, Δ +0.2 ppm.

2'-Methoxy-6,6'-bis(4-(trifluoromethoxy)phenyl)-[1,1'-binaphthalen]-2-ol (37)

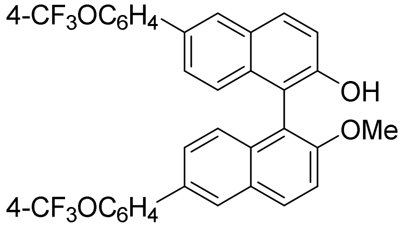

To a flask containing **33** (500 mg, 1.09 mmol, 1.00 equiv), (4-(trifluoromethoxy)phenyl)boronic acid (562 mg, 2.73 mmol, 2.50 equiv) and $Pd(PPh_3)_4$ (63.1 mg, 54.6 μmol, 0.05 equiv) was added 1,2-dimethoxyethane (11.50 mL, Ar sparged) and Na_2CO_3 (2.0 M aq, 2.72 mL, Ar sparged). The resulting mixture was heated to 90 °C for 16 hours and cooled to room temperature before addition of water (10 mL) and EtOAc (10 mL). The layers were separated and the aqueous layer was extracted with EtOAc (10 mL × 2). The combined organic layers were washed with brine, dried over anhydrous $MgSO_4$, filtered and the solvent removed under reduced pressure. The resulting solid was purified by flash column chromatography (2:1 CH_2Cl_2 – petroleum ether 40-60) to give the title compound **37** as a white solid (511 mg, 0.82 mmol, 75% yield).

m.p. = 110-112 °C (CH_2Cl_2/petroleum ether 40-60).

IR (film) ν_{max}/cm^{-1}: 3521, 1598, 1497, 1251, 1211, 1164, 1089, 1059, 854.

^{1}H NMR (500 MHz, $CDCl_3$) δ_H: 8.14 (d, J = 9.1 Hz, 1H), 8.08 (d, J = 1.8 Hz, 1H), 8.05 (d, J = 1.8 Hz, 1H), 7.98 (d, J = 8.9 Hz, 1H), 7.70-7.65 (m, 4H), 7.55 (d, J = 9.1 Hz, 1H), 7.52 (dd, J = 1.9, 8.8 Hz, 1H), 7.46 (dd, J = 1.9, 8.8 Hz, 1H), 7.41 (d, J = 8.9 Hz, 1H), 7.34-7.27 (m, 5H), 7.16 (d, J = 8.8 Hz, 1H), 4.98 (s, 1H), 3.86 (s, 3H)

^{13}C NMR (126 MHz, $CDCl_3$) δ_C: 156.4, 151.8, 148.8, 148.8, 148.6, 140.1, 139.7, 135.7, 134.8, 133.5, 133.2, 131.7, 130.4, 129.7, 129.5, 128.6, 128.6, 1270, 126.5, 126.4, 126.0, 125.8, 125.7, 121.5, 121.4, 120.7 (q, J = 257.4 Hz), 118.4, 115.1, 115.0, 114.4, 56.8.

^{19}F NMR (470 MHz, $CDCl_3$) δ_F: -57.8 (s).

HRMS (ESI$^-$) $C_{35}H_{21}F_6O_4^-$ [M-H]$^-$: requires: 619.13495, found: 619.13458, Δ -0.6 ppm.

6-Bromo-2'-methoxy-[1,1'-binaphthalen]-2-ol (21)

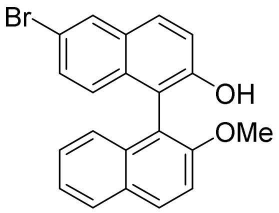

6,6'-Dibromo-2'-methoxy-[1,1'-binaphthalen]-2-ol (**33**) (1.95 g, 4.26 mmol, 1.00 equiv.) was dissolved in THF (43 mL) and cooled to −78 °C. ^{n}BuLi (1.6 M in hexane, 5.33 mL, 8.52 mmol, 2.00 equiv.) was added by syringe pump over 1 hour after which time the reaction was stirred for a further 1 hour at −78 °C. The reaction was quenched by dropwise addition of water. After warming to room temperature, saturated ammonium chloride solution was added. EtOAc was added and the phases were separated. The aqueous phase was extracted twice with EtOAc and the combined organic layers were washed with brine, dried over anhydrous sodium sulfate, filtered and concentrated to give the crude product. Purification *via* flash column chromatography (4:1 petroleum ether 40-60 – EtOAc) afforded 6-bromo-2'-methoxy-[1,1'-binaphthalen]-2-ol (**21**) (1.27 g, 82%)

m.p. = 132-133 °C (EtOAc/petroleum ether 40-60).

IR (film) ν_{max}/cm^{-1}: 3514, 3059, 2936, 2839, 1588, 1504, 1498, 1474, 1459, 1432, 1379, 1350, 1331, 1307, 1262, 1245, 1205, 1182, 1167, 1144, 1129, 1085, 1053, 974, 936, 903, 879, 866, 809, 782, 747, 673.

^{1}H NMR (400 MHz, $CDCl_3$) δ = 7.97 (d, *J* = 9.1 Hz, 1H), 8.02 (d, *J* = 2.1 Hz, 1H), 7.91 (d, *J* = 8.1 Hz, 1H), 7.81 (d, *J* = 8.9 Hz, 1H), 7.48 (d, *J* = 9.1 Hz, 1H), 7.41 (obs. m, 1H), 7.37 (d, *J* = 8.8 Hz, 1H), 7.33–7.26 (m, 2H), 7.14 (d, *J* = 8.5 Hz, 1H), 6.93 (d, *J* = 9.0 Hz, 1H), 4.96 (s, 1H, OH), 3.81 (s, 3H).

^{13}C NMR (101 MHz, $CDCl_3$) δ = 156.0, 151.6, 133.9, 132.3, 131.4, 130.3, 130.1, 129.6, 129.4, 128.9, 128.3, 127.5, 126.7, 124.6, 124.3, 118.6, 117.0, 115.3, 114.5, 113.7, 56.0

HRMS (ESI^-) $C_{21}H_{15}O_2{}^{79}Br$ $[M-H]^-$ requires 377.01827; found 377.01807, Δ −0.5 ppm.

Data in agreement with literature[7]

2',6-dimethoxy-[1,1'-binaphthalen]-2-ol (23)

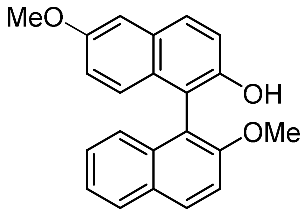

6-Bromo-2'-methoxy-[1,1'-binaphthalen]-2-ol (**21**) (190 mg, 0.500 mmol, 1.00 equiv.) was dissolved in DMF (5.0 mL) then CuCl (149 mg, 1.50 mmol 3.00 equiv.) and NaOMe (4.4 M in MeOH, 1.37 mL, 6.00 mmol, 12.0 equiv.) were added sequentially. The reaction was heated at 100 °C for 1 h then cooled to room temperature. Ice water was added followed by hydrochloric acid (3 M). The resulting suspension was extracted 3 times with EtOAc and the combined organic layers were washed with brine, dried over anhydrous sodium sulfate, filtered and concentrated to give the crude product. Purification *via* flash column chromatography (3:1 CH_2Cl_2 – petroleum ether 40-60 to 9:1 CH_2Cl_2 – petroleum ether 40-60) afforded 2',6-dimethoxy-[1,1'-binaphthalen]-2-ol (**23**) as an off-white solid (104 mg, 63%).

m.p. = 160-162 °C (CH_2Cl_2/petroleum ether 40-60).

IR (film) ν_{max}/cm^{-1}: 3501, 3430, 3056, 3007, 2936, 2839, 1620, 1600, 1508, 1464, 1433, 1366, 1331, 1265, 1249, 1236, 1209, 1171, 1148, 1125, 1083, 1056, 1033, 852, 811, 752

^{1}H NMR (500 MHz, $CDCl_3$) δ = 8.04 (d, J = 9.1 Hz, 1H), 7.90 (d, J = 8.2 Hz, 1H), 7.81 (d, J = 8.9 Hz, 1H), 7.47 (d, J = 9.1 Hz, 1H), 7.38 (ddd, J = 1.2, 6.9, 8.1 Hz, 1H), 7.35 (d, J = 8.9 Hz, 1H), 7.29 (ddd, J = 1.3, 6.9, 8.4 Hz, 1H), 7.22-7.18 (m, 2H), 6.98 (d, J = 9.2 Hz, 1H), 6.92 (dd, J = 2.6, 9.2 Hz, 1H), 4.82 (s, 1H), 3.90 (s, 3H), 3.81 (s, 3H).

^{13}C NMR (126 MHz, $CDCl_3$) δ = 156.0, 155.9, 149.8, 134.1, 131.1, 130.1, 129.5, 129.1, 128.5, 128.3, 127.4, 126.5, 125.0, 124.3, 119.0, 118.0, 115.6, 115.4, 113.9, 106.6, 56.8, 55.4.

HRMS (ESI$^+$) $C_{22}H_{19}O_3^+$ $[M+H]^+$: requires: 331.13287, found: 331.13303, Δ +0.5 ppm.

2'-Methoxy-6-morpholino-[1,1'-binaphthalen]-2-ol (19)

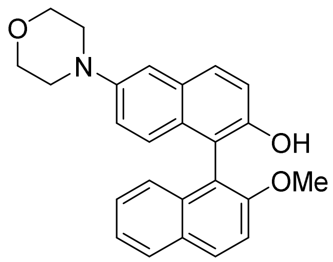

6-Bromo-2'-methoxy-[1,1'-binaphthalen]-2-ol (**21**) (379 mg, 1.00 mmol, 1.00 equiv.), $Pd_2(dba)_3$ (9.2 mg, 0.0100 mmol, 1 mol%) and DavePhos (4.7 mg, 0.0120 mmol, 1.2 mol%) were added to a Schlenk flask. The flask was evacuated and backfilled with argon three times. Morpholine (105 mg, 105 µL, 1.20 mmol, 1.20 equiv.) was added followed by LiHMDS solution (1 M in THF, 2.2 mL, 2.2 mmol, 2.2 equiv.) and the reaction was stirred at room temperature for 16 hours. 1 M HCl solution was added and the reaction was stirred for 30 minutes. 1 M $NaHCO_3$ solution was added and the aqueous solution was extracted three times with CH_2Cl_2. The combined organic layers were dried over anhydrous sodium sulfate, filtered and concentrated to give the crude product which was purified by column chromatography (3:2 petroleum ether 40-60 – EtOAc) to give 2'-methoxy-6-morpholino-[1,1'-binaphthalen]-2-ol (**19**) (244 mg, 63%) as an off-white solid.

m.p. = 231-232 °C (EtOAc/petroleum ether 40-60).

IR (film) ν_{max}/cm^{-1}: 3283, 1608, 1591, 1506, 1461, 1449, 1380, 1356, 1335, 1309, 1282, 1265, 1253, 1228, 1199, 1182, 1167, 1146, 1134, 1113, 1088, 1062, 1044, 1022, 960, 934, 879, 856, 848, 817, 798, 779, 772, 759, 747, 731, 699, 666, 638, 616.

^{1}H NMR (400 MHz, $CDCl_3$) δ_H = 7.94 (d, *J* = 9.0 Hz, 1H), 7.79 (d, *J* = 8.1 Hz, 1H), 7.66 (d, *J* = 8.8 Hz, 1H), 7.37 (d, *J* = 9.1 Hz, 1H), 7.26 (ddd, *J* = 8.1, 6.7, 1.3 Hz, 1H), 7.20 (d, *J* = 8.9 Hz, 1H), 7.18–7.14 (m, 1H), 7.09 (m, 2H), 6.93–6.83 (m, 2H), 4.69 (br. s, 1H), 3.81–3.74 (m, 4H), 3.69 (s, 3H), 3.15–3.05 (m, 4H).

^{13}C NMR (101 MHz, $CDCl_3$) δ_C = 155.9, 149.8, 147.2, 134.0, 130.9, 130.0, 129.4, 128.9, 128.6, 128.1, 127.3, 125.9, 125.0, 124.2, 119.6, 117.9, 115.6, 115.1, 113.9, 111.0, 67.0, 56.7, 50.1.

HRMS (ESI^+) $C_{25}H_{23}O_3N$ $[M+H]^+$ requires 386.17507; found 386.17477, Δ -0.8 ppm.

2'-Methoxy-6-phenyl-[1,1'-binaphthalen]-2-ol (20)

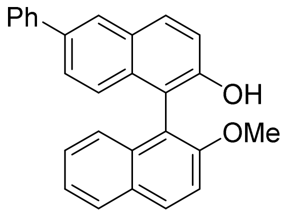

6-Bromo-2'-methoxy-[1,1'-binaphthalen]-2-ol (**21**) (365 mg, 1.00 mmol, 1.00 equiv.), phenyl boronic acid (144 mg, 1.20 mmol, 1.20 equiv.) and $Pd(PPh_3)_4$ (57.8 mg, 0.0500 mmol, 0.0500 equiv.) were charged to a flask fitted with a water-cooled condenser. The flask was evacuated and backfilled with argon three times. Argon sparged 1,2-dimethoxyethane (10 mL) was added followed by argon sparged aqueous Na_2CO_3 solution (2 M, 1.25 mL, 2.50 equiv.). The flask was fitted with an argon balloon and heated at 90 °C for 16 hours. After cooling to room temperature, 1 M HCl solution was added and the solution was extracted three times with CH_2Cl_2, the combined organic layers were dried over anhydrous sodium sulfate, filtered and concentrated to give the crude product which was purified by column chromatography (9:1 petroleum ether 40-60 – EtOAc) to give 2'-methoxy-6-phenyl-[1,1'-binaphthalen]-2-ol (**20**) (233 mg, 62%) as a white solid.

m.p. = 208-209 °C (EtOAc/petroleum ether 40-60).

IR (film) ν_{max}/cm^{-1}: 3513, 1507, 1493, 1145, 817, 755, 705.

^{1}H NMR (400 MHz, $CDCl_3$) δ = 8.01–7.93 (m, 2H), 7.87 (d, *J* = 8.9 Hz, 1H), 7.82 (d, *J* = 8.1 Hz, 1H), 7.58 (m, 2H), 7.44–7.11 (m, 9H), 7.05–7.01 (m, 1H), 4.86 (br. S, 1H), 3.73 (s, 3H)

^{13}C NMR (101 MHz, $CDCl_3$) δ = 156.0, 151.4, 141.2, 136.0, 134.0, 133.0, 131.2, 130.1, 129.5, 129.4, 128.8, 128.2, 127.4, 127.2, 127.0, 126.1, 126.1, 125.4, 124.9, 124.2, 117.9, 115.2, 115.0, 113.8, 56.7.

HRMS (ESI$^+$) $C_{27}H_{20}O_2$ $[M+H]^+$ requires 377.15361; found 377.15365, Δ +0.1 ppm.

4.3. 5-Substituted BINOLs

1-Bromo-6-methoxynaphthalene (57)

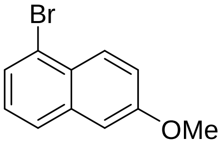

Following a modified literature procedure.[6] Triphenylphosphite (28.9 mL, 34.1 g, 110 mmol, 1.1 equiv.) was dissolved in CH_2Cl_2 (300 mL) and the resulting solution was cooled to -78 °C. Bromine (6.15 mL, 20.3 g, 120 mmol, 1.20 equiv.) was added followed by dropwise addition of Et_3N (18.1 mL, 13.2 g, 130 mmol, 1.30 equiv.) over 30 minutes. 6-Methoxy-3,4-dihydronaphthalen-1(2H)-one (17.6 g, 100 mmol, 1.00 equiv.) was added and the reaction was warmed to room temperature and stirred for 16 hours after which time the reaction was heated at 45 °C for 2 hours. Upon cooling to room temperature saturated Na_2SO_3 solution was added and the resulting solution was extracted three times with CH_2Cl_2. The combined organic layers were washed with water, dried over anhydrous sodium sulfate, filtered and concentrated to give the crude product. Purification *via* flash column chromatography (39:1 petroleum ether 40-60 – CH_2Cl_2 to 19:1 petroleum ether 40-60 – CH_2Cl_2) afforded the vinyl bromide which was found to be unstable and was therefore used immediately in the next reaction.

The product of the previous step (18.5 g, crude) was dissolved in benzene (155 mL) and DDQ (19.3 g, 85.1 mmol) was added and the reaction was stirred at room temperature for 2 hours after which time saturated Na_2SO_3 solution was added and the resulting suspension was extracted 3 times with EtOAc. The combined organic layers were washed 3 times with saturated $NaHCO_3$ solution, brine, dried over anhydrous sodium sulfate, filtered and concentrated to give the crude product. Purification *via* flash column chromatography (39:1 petroleum ether 40-60 – CH_2Cl_2 to 19:1 petroleum ether 40-60 – CH_2Cl_2) afforded 1-bromo-6-methoxynaphthalene (**57**) (6.93 g, 29% over 2 steps) as a light-yellow oil.

IR (film) ν_{max}/cm^{-1} 1623, 1501, 1469, 1424, 1359, 1263, 1236, 1197, 1169, 1125, 1032, 957, 916, 840, 818, 803, 775, 745, 658.

^{1}H NMR (500 MHz, $CDCl_3$) δ = 8.16 (d, J = 9.28 Hz, 1H), 7.72 (d, J = 8.35 Hz, 1H), 7.64 (d, J = 7.19 Hz, 1H), 7.32-7.24 (m, 2H), 7.15 (d, J = 2.50 Hz, 1H), 3.96 (s, 3H).

^{13}C NMR (126 MHz, $CDCl_3$) δ =158.2, 135.9, 128.8, 127.6, 127.5, 126.8, 126.7, 122.7, 120.0, 106.1, 55.4.

Data in agreement with literature reported[8]

5-Bromonaphthalen-2-ol (51)

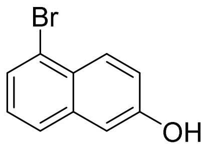

1-Bromo-6-methoxynaphthalene (**57**) (6.93 g, 29.2 mmol, 1.00 equiv.) was dissolved in CH_2Cl_2 (146 mL) and the resulting solution was cooled to 0 °C. BBr_3 (3.37 mL, 8.75 g, 35.4 mmol, 1.20 equiv.) was added dropwise over 5 minutes. The reaction was allowed to warm to room temperature and was stirred for 16 hours. The reaction was quenched with water and extracted 3 times with CH_2Cl_2. The combined organic layers were dried over anhydrous sodium sulfate, filtered and concentrated to give the crude product. Purification *via* flash column chromatography (1:1 CH_2Cl_2 – petroleum ether) gave 5-bromonaphthalen-2-ol (**51**) (6.35 g, 97%) as an off-white solid.

m.p. = 104-105 °C (CH_2Cl_2/petroleum ether 40-60).

IR (film) ν_{max}/cm^{-1}: 3244, 1625, 1563, 1504, 1427, 1382, 1252, 1230, 965, 773.

^{1}H NMR (400 MHz, $CDCl_3$) δ = 8.18 (d, *J* = 9.1 Hz, 1H), 7.69-7.62 (m, 2H), 7.28 (app. t, partially obscured, *J* = 8.8 Hz), 7.22 (dd, *J* = 9.1, 2.6 Hz, 1H), 7.18 (d, *J* = 2.5, 1H), 5.09 (br. s, 1H).

^{13}C NMR (101 MHz, $CDCl_3$) δ = 154.0, 135.8, 129.4, 127.7, 127.5, 127.0, 126.4, 122.8, 119.0, 109.9.

HRMS (ESI^-) $C_{10}H_7{}^{79}BrO$ $[M-H]^-$ requires 220.96081, found: 220.96070, Δ -0.5 ppm.

Data in agreement with literature reported[8]

5,5'-Dibromo-[1,1'-binaphthalene]-2,2'-diol (55)

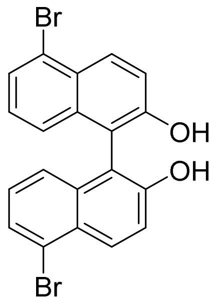

5-Bromonaphthalen-2-ol (**51**) (6.35 g, 28.5 mmol, 1.00 equiv.) was dissolved in CH_2Cl_2 (190 mL) and $[Cu(TMEDA)OHCl)]_2$ (130 mg, 0.280 mmol, 1 mol%) was added. The reaction was stirred for 16 hours at room temperature open to air. The reaction mixture was filtered through a short silica plug eluting with CH_2Cl_2. Evaporation of the solvent gave 5,5'-dibromo-[1,1'-binaphthalene]-2,2'-diol (**55**) (6.28 g, 100%) as an off-white solid.

m.p. = 79-80 °C (CH_2Cl_2).

IR (film) ν_{max}/cm^{-1}: 3367, 2923, 2360, 1698, 1615, 1572, 1541, 1497, 1395, 1331, 1252, 1197, 1132, 981, 913, 820, 799, 656.

^{1}H NMR (500 MHz, $CDCl_3$) δ = 8.46 (d, J = 9.48 Hz, 2H), 7.70 (dd, J = 7.15, 0.89, 2H), 7.51 (d, J = 9.30 Hz, 2H), 7.17 (dd, J = 8.41, 7.33 Hz, 2H), 7.09 (d, J = 8.59 Hz, 2H), 5.09 (s, 2H).

^{13}C NMR (126 MHz, $CDCl_3$) δ = 153.4, 134.7, 131.0, 128.4, 128.0, 128.0, 124.0, 123.5, 119.0, 111.0.

HRMS (ESI^+) $C_{20}H_{11}{}^{79}Br_2O_2$ $[M+H]^+$ requires 440.91203, found: 440.91260, Δ +1.3 ppm.

5,5'-Dibromo-2'-methoxy-[1,1'-binaphthalen]-2-ol (38)

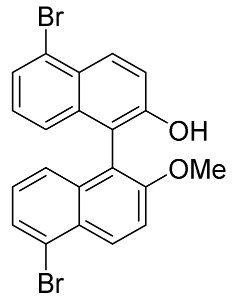

5,5'-Dibromo-[1,1'-binaphthalene]-2,2'-diol (**55**) (5.80 g, 13.1 mmol, 1.00 equiv.) was dissolved in acetone (115 mL) and K_2CO_3 (2.35 g, 17.0 mmol, 1.30 equiv.) followed by iodomethane (900 µL, 2.04 g, 14.4 mmol, 1.10 equiv.) were added. The reaction was heated under reflux for 16 hours. Upon cooling to room temperature, the solvent was removed *in vacuo*. The residue was partitioned between water and CH_2Cl_2 and the phases were separated. The aqueous layer was extracted twice with CH_2Cl_2 and the organic layers were combined, dried over anhydrous sodium sulfate, filtered and concentrated to give the crude product. Purification *via* flash column chromatography (1:1 CH_2Cl_2 – petroleum ether 40-60) gave 5,5'-dibromo-2'-methoxy-[1,1'-binaphthalen]-2-ol (**38**) (3.02 g, 50%) as an off-white solid.

m.p. = 133-135 °C (CH_2Cl_2/petroleum ether 40-60).

IR (film) ν_{max}/cm^{-1}: 3525, 2923, 2848, 2360, 2341, 1772, 1733, 1716, 1698, 1684, 1652, 1611, 1588, 1560, 1394, 1371, 1192, 1050, 913, 752, 690, 668, 655.

^{1}H NMR (500 MHz, $CDCl_3$) δ_H: 8.53 (d, *J* = 9.5 Hz, 1H), 8.37 (d, *J* = 9.3 Hz, 1H), 7.69 (dd, *J* = 6.49, 1.9 Hz, 1H), 7.63 (dd, *J* = 7.38, 1.0 Hz, 1H), 7.60 (d, *J* = 9.4 Hz, 1H), 7.47 (d, *J* = 9.2 Hz, 1H), 7.16-7.10 (m, *2*H), 7.07 (dd, *J* = 8.40, 7.4 Hz, 1H), 6.98 (d, *J* = 8.5 Hz, 1H), 4.91 (s, 1H), 3.86 (s, 3H).

^{13}C NMR (127 MHz, $CDCl_3$) δ_C: 156.6, 151.9, 135.2, 135.0, 130.9, 129.4, 128.3, 127.9, 127.8, 127.6, 127.5, 126.9, 124.7, 124.7, 123.2, 123.2, 118.8, 115.1, 115.1, 114.6, 56.6.

HRMS (ESI^+) $C_{21}H_{13}O_2{}^{79}Br_2$ $[M-H]^-$ requires 454.92878; found 454.92850, Δ −0.6 ppm.

2'-methoxy-5,5'-dimethyl-[1,1'-binaphthalen]-2-ol (39)

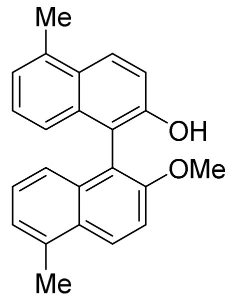

To a flask containing 5,5'-dibromo-2'-methoxy-[1,1'-binaphthalen]-2-ol (**38**) (200 mg, 0.437 mmol, 1.00 equiv.), Pd(dppf)Cl_2·CH_2Cl_2 (35.6 mg, 0.044 mmol, 0.10 equiv.), cesium carbonate (569 mg, 1.75 mmol, 4.00 equiv.) and methylboronic acid (104 mg, 1.75 mmol, 4.00 equiv.), was added 1,4-dioxane (4.50 mL, N_2 sparged). The resulting suspension was heated at 105 °C for 48 h. The mixture was cooled to room temperature, diluted with EtOAc (10.0 mL) and filtered through a short plug of silica (eluting with EtOAc). The resulting solution was concentrated *in vacuo* and purified by flash column chromatography (2:1 petroleum ether 40-60 – diethyl ether) to give 2'-methoxy-5,5'-dimethyl-[1,1'-binaphthalen]-2-ol (**39**) as an off-white powder (135 mg, 94%)

m.p. = 142-144°C. (CH_2Cl_2/petroleum ether 40-60)

IR (film) ν_{max}/cm^{-1}: 3530, 2917, 2849, 1613, 1595, 1511, 1458, 1405, 1381, 1327, 1266, 1210, 1180, 1153, 1091, 1074, 1050, 802, 757

^{1}H NMR (500 MHz, $CDCl_3$) δ_H: 8.23 (dd, *J* = 9.2, 0.4, 1H), 8.08 (d, *J* = 9.2 Hz, 1H), 7.51 (d, *J* = 9.4 Hz, 1H), 7.38 (d, *J* = 9.2 Hz, 1H), 7.20 (d, *J* = 6.8 Hz), 7.18-7.13 (m, 2H), 7.10 (dd, *J* = 8.4, 7.0 Hz, 1H), 7.03 (d, *J* = 8.5, 1H), 6.90 (d, *J* = 8.3 Hz, 1H), 4.85 (s, 1H), 3.80 (s, 3H), 2.76 (s, 3H), 2.74 (s, 3H)

^{13}C NMR (126 MHz, $CDCl_3$) δ_C: 155.9, 151.1, 134.7, 134.6, 134.5, 134.1, 128.7, 128.3, 127.4, 127.3, 126.4, 126.1, 125.2, 124.4, 123.5, 117.0, 116.1, 113.4, 56.8, 19.8, 19.8.

HRMS (ESI$^+$) $C_{23}H_{21}O_2^+$ [M+H]$^+$ requires 329.15361; found: 329.15364, Δ +0.1 ppm

2'-methoxy-5,5'-diphenyl-[1,1'-binaphthalen]-2-ol (40)

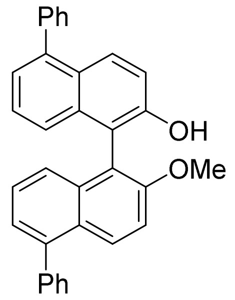

To a flask containing **38** (500 mg, 1.09 mmol, 1.00 equiv), phenylboronic acid (333 mg, 2.73 mmol, 1.00 equiv) and $Pd(PPh_3)_4$ (63.1 mg, 54.6 µmol, 0.05 equiv) was added 1,2-dimethoxyethane (11.50 mL, Ar sparged) and sodium carbonate (2.0 M aq, 2.72 mL, Ar sparged). The resulting mixture was heated to 90 °C for 16 hours and cooled to room temperature before addition of water (10 mL) and EtOAc (10 mL). The layers were separated and the aqueous layer was extracted with EtOAc (10 mL × 2). The combined organic layers were washed with brine, dried over anhydrous $MgSO_4$, filtered and the solvent removed under reduced pressure. The resulting solid was purified by flash column chromatography (1:1 CH_2Cl_2 – petroleum ether 40-60 to 2:1 CH_2Cl_2 – petroleum ether 40-60) to give the title compound **40** as a white solid (360 mg, 0.80 mmol, 73% yield).

m.p. = 134-136 °C. (CH_2Cl_2/petroleum ether 40-60)

IR (film) ν_{max}/cm^{-1}: 3534, 3058, 2917, 2360, 1608, 1510, 1491, 1463, 1405, 1324, 1264, 1187, 1118, 1086, 809, 760, 702.

^{1}H NMR (500 MHz, $CDCl_3$) δ_H: 8.14 (d, *J* = 9.4 Hz, 1H), 7.99 (d, *J* = 9.4 Hz, 1H), 7.61-7.51 (m, 8H), 7.51-7.41 (m, 3H), 7.37-7.23 (m, 7H), 7.13 (dd, *J* = 8.0, 1.1 Hz, 1H), 4.94 (s, 1H), 3.83 (s, 3H).

^{13}C NMR (126 MHz, $CDCl_3$) δ_C: 156.0, 151.3, 141.4, 141.0, 140.8, 140.7, 134.7, 134.3, 130.3, 130.3, 129.6, 128.5, 128.4, 128.2, 127.8, 127.5, 127.4, 127.3, 127.1, 126.2, 125.5, 124.7, 124.7, 117.5, 115.7, 115.6, 113.7, 56.8.

HRMS (ESI$^-$) $C_{33}H_{23}O_2^+$ [M-H]$^-$ requires 451.17035; found: 451.17035, Δ -0.0 ppm

5-Bromo-2'-methoxy-[1,1'-binaphthalen]-2-ol (24)

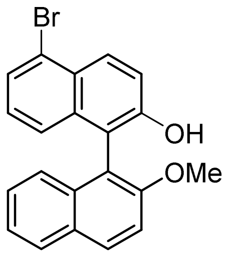

5,5'-Dibromo-2'-methoxy-[1,1'-binaphthalen]-2-ol (**38**) (2.37 g, 5.17 mmol, 1.00 equiv.) was dissolved in THF (52 mL) and cooled to −78 °C. ^{n}BuLi (2.5 M in hexane, 4.14 mL, 10.3 mmol, 2.00 equiv.) was added by syringe pump over 1 hour after which time the reaction was stirred for a further 1 hour at −78 °C. The reaction was quenched by dropwise addition of water. After warming to room temperature, saturated ammonium chloride solution was added. EtOAc was added and the phases were separated. The aqueous phase was extracted twice with EtOAc and the combined organic layers were washed with brine, dried over anhydrous sodium sulfate, filtered and concentrated to give the crude product. Purification *via* flash column chromatography (4:1 petroleum ether 40-60 – EtOAc) afforded 5-bromo-2'-methoxy-[1,1'-binaphthalen]-2-ol (**24**) (1.03 g, 52%)

m.p. = 152-153 °C (EtOAc/petroleum ether 40-60).

IR (film) ν_{max}/cm^{-1}: 3486, 3409, 2980, 1613, 1592, 1507, 1461, 1369, 1346, 1333, 1268, 1252, 1231, 1214, 1192, 1173, 1147, 1131, 1086, 1055, 807, 751.

^{1}H NMR (400 MHz, $CDCl_3$) δ = 8.35 (d, *J* = 9.3 Hz, 1H), 8.07 (d, *J* = 9.1 Hz, 1H), 7.91 (d, *J* = 8.4 Hz, 1H), 7.61 (dd, *J* = 5.9, 2.5 Hz, 1H), 7.48 (d, *J* = 9.0 Hz, 1H), 7.45 (d, *J* = 9.0 Hz, 1H), 7.38 (ddd, *J* = 8.1, 6.8, 1.3 Hz, 1H), 7.30 (ddd, *J* = 8.3, 6.8, 1.4 Hz, 1H), 7.13 (d, *J* = 7.5 Hz, 1H), 7.07–7.00 (m, 2H), 4.98 (s, 1H), 3.81 (s, 3H).

^{13}C NMR (101 MHz, $CDCl_3$) δ = 156.1, 152.1, 135.2, 134.0, 131.5, 129.5, 129.2, 128.4, 127.7, 127.7, 127.5, 126.9, 125.0, 124.8, 124.4, 123.3, 118.9, 115.6, 114.8, 113.8, 56.7.

HRMS (ESI$^+$) $C_{21}H_{13}O_2{}^{79}Br$ $[M+H]^+$ requires 377.01827; found 377.01822, Δ −0.1 ppm.

2',5-dimethoxy-[1,1'-binaphthalen]-2-ol (23)

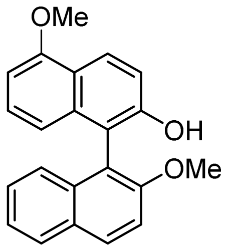

5-Bromo-2'-methoxy-[1,1'-binaphthalen]-2-ol (**24**) (190 mg, 0.500 mmol, 1.00 equiv.) was dissolved in DMF (5 mL) then CuCl (149 mg, 1.50 mmol 3.00 equiv.) and NaOMe (4.4 M in MeOH, 1.37 mL, 6.00 mmol, 12.0 equiv.) were added sequentially. The reaction was heated at 100 °C for 1 h then cooled to room temperature. Ice water was added followed by hydrochloric acid (3 M). The resulting suspension was extracted 3 times with EtOAc and the combined organic layers were washed with brine, dried over anhydrous sodium sulfate, filtered and concentrated to give the crude product. Purification *via* flash column chromatography (1:1 CH_2Cl_2 – petroleum ether 40-60 to 4:1 CH_2Cl_2 – petroleum ether 40-60) afforded 2',5-dimethoxy-[1,1'-binaphthalen]-2-ol (**23**) as an off-white solid (123 mg, 75%).

m.p. = 80-81 °C (CH_2Cl_2/ petroleum ether 40-60).

^{1}H NMR (400 MHz, $CDCl_3$) δ = 8.38 (d, J = 9.1 Hz, 1H), 8.04 (d, J = 9.1 Hz, 1H), 7.97-7.86 (m, 1H), 7.47 (d, J = 9.1 Hz, 1H), 7.42-7.32 (m, 2H), 7.29 (ddd, J = 8.2, 6.7, 1.4 Hz, 1H), 7.20 (dd, J = 8.5, 1.2 Hz, 1H), 7.14 (dd, J = 8.5, 7.6 Hz, 1H), 6.68 (d, J = 7.6 Hz, 1H), 6.64 (d, J = 8.5 Hz, 1H), 4.93 (s, 1H), 4.02 (s, 3H), 3.81 (s, 3H).

^{13}C NMR (101 MHz, $CDCl_3$) δ = 156.0, 155.9, 151.8, 135.0, 134.1, 131.0, 129.4, 128.1, 127.3, 126.7, 125.0, 124.2, 124.0, 121.0, 117.4, 116.4, 115.6, 114.9, 113.8, 101.7, 56.7, 55.5.

IR (film) ν_{max}/cm^{-1}: 3493, 3434, 3063, 3003, 2958, 2936, 2838, 1620, 1591, 1464, 1431, 1415, 1333, 1301, 1268, 1252, 1223, 1165, 1149, 1122, 1076, 1052, 1020.

HRMS (ESI$^+$) $C_{22}H_{18}O_3$ [M+H]$^+$ requires 331.13287; found 31.13293, Δ +0.2 ppm

2'-Methoxy-5-methyl-[1,1'-binaphthalen]-2-ol (22)

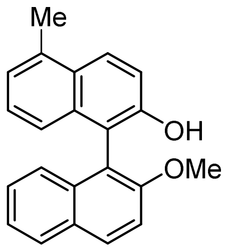

5-Bromo-2'-methoxy-[1,1'-binaphthalen]-2-ol (**24**) (379 mg, 1.00 mmol, 1.00 equiv.) and Pd(dppf)Cl_2.CH_2Cl_2 (98 mg, 0.100 mmol, 10 mol%) were dissolved in THF (10 mL) and MeMgCl solution (3 M in Et_2O, 1.70 mL, 5.00 mmol, 5.00 equiv.) was added. The resulting solution was heated under reflux for 16 hours. On cooling to room temperature, saturated ammonium chloride solution was added and phases were separated. The aqueous phase was extracted twice with EtOAc and the combined organic layers were washed with brine, dried over anhydrous sodium sulfate, filtered and concentrated to give the crude product which was purified by column chromatography (9:1 petroleum ether 40-60 – EtOAc) to give 2'-methoxy-7-methyl-[1,1'-binaphthalen]-2-ol (**22**) (334 mg, 88%) as an off-white solid.

m.p. = 135-136 °C (EtOAc/petroleum ether 40-60).

IR (film) ν_{max}/cm^{-1}: 3507, 1592, 1272, 1210, 1186, 1067, 808, 800, 752.

^{1}H NMR (400 MHz, $CDCl_3$) δ = 8.00 (d, J = 9.1 Hz, 1H), 7.94 (d, J = 9.0 Hz, 1H), 7.80 (d, J = 8.3 Hz, 1H), 7.37 (d, J = 9.1 Hz, 1H), 7.30 (d, J = 9.2 Hz, 1H), 7.27–7.24 (m, 1H), 7.20–7.14 (m, 1H), 7.09–6.98 (m, 3H), 6.83 (d, J = 8.2 Hz, 1H), 4.81 (s, 1H), 3.70 (s, 3H), 2.65 (s, 3H).

^{13}C NMR (101 MHz, $CDCl_3$) δ = 156.0, 151.0, 134.5, 134.1, 133.9, 131.0, 129.4, 128.2, 128.1, 127.3, 126.3, 126.0, 125.0, 124.3, 124.2, 123.4, 117.0, 115.6, 115.6, 113.8, 56.7, 19.8.

HRMS (ESI$^+$) $C_{22}H_{20}O_2$ $[M+H]^+$ requires 315.13796; found 315.13797, Δ 0.0 ppm.

4.4. 4-Substituted BINOLs

8-Bromo-6-methoxy-3,4-dihydronaphthalen-1(2H)-one (58)

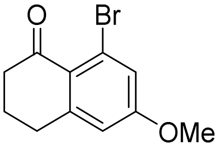

Following a modified literature procedure,[9] 6-methoxy-3,4-dihydronaphthalen-1(2H)-one (25.0 g, 142 mmol, 1.00 equiv.) and methoxyamine hydrochloride (23.4 g, 284 mmol, 2.00 equiv.) were dissolved in ethanol (284 mL) and pyridine (28.4 mL) was added. The resulting solution was stirred at room temperature for 16 hours. The solution was concentrated *in vacuo* and 2 M HCl solution was added followed by CH_2Cl_2. The organic phase was separated and the aqueous phase was extracted twice with CH_2Cl_2. The combined organic layers were washed with 1 M $NaHCO_3$ solution, brine, dried over anhydrous sodium sulfate, filtered and concentrated to give the crude product which was used without further purification.

The crude oxime ether was dissolved in acetic acid (568 mL). $Pd(OAc)_2$ (1.59 g, 7.01 mmol, 5 mol% equiv.) and N-bromosuccinimide (30.3 g, 170 mmol, 1.20 equiv.) were added and the reaction was heated at 80°C for 1 hour. On cooling to room temperature, the reaction mixture was filtered through celite and concentrated. The residue was dissolved in Et_2O and the resulting solution was washed with water, washed three times with NaOH solution (1 M), washed with brine and concentrated to give the crude product which was used without further purification.

The crude brominated oxime ether was dissolved in 2:3 dioxane-6M HCl (710 mL) and the solution was heated under reflux for 1 hour. On cooling to room temperature, the solution was extracted three times with Et_2O and the combined organic layers were washed with NaOH solution (1 M), brine and concentrated to give the crude product. Recrystallization from hexane-EtOAc afforded 8-bromo-6-methoxy-3,4-dihydronaphthalen-1(2H)-one (**58**) (21.0 g, 58%) as an off-white solid.

m.p. = 115-117 °C (*n*-hexane-EtOAc)

IR (film) ν_{max}/cm^{-1}: 1946, 1675, 1586, 1555, 1245, 1121, 1038, 884, 874, 841, 791.

^{1}H NMR ($CDCl_3$, 400 MHz) δ = 7.06 (d, *J* = 2.6 Hz, 1H), 6.68 (dd, *J* = 2.6, 1.1 Hz, 1H), 3.82 (s, 3H), 2.93 (app. t, *J* = 6.1 Hz, 2H), 2.62 (dd, *J* = 7.2, 6.0 Hz, 2H) 2.05 (tt, *J* = 7.3, 6.2 Hz, 2H).

^{13}C NMR ($CDCl_3$, 101 MHz) δ = 195.4, 161.8, 148.8, 124.2, 123.6, 119.9, 113.0, 55.7, 39.9, 31.5, 22.5.

HRMS (ESI^+) $C_{11}H_{11}O_2Br$ $[M+H]^+$ requires 255.00152; found 255.00160, Δ +0.3 ppm.

Data in agreement with literature reported[9]

1-Bromo-3-methoxynaphthalene (56)

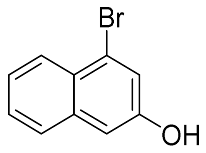

8-Bromo-6-methoxy-3,4-dihydronaphthalen-1(2H)-one (**58**) (21.0 g, 82.3 mmol, 1.00 equiv.) was dissolved in ethanol (164 mL) and the solution was cooled to 0 °C. $NaBH_4$ (6.22 g, 165 mmol, 2.00 equiv.) was added portionwise and the reaction was allowed to warm to room temperature over 16 hours. Saturated ammonium chloride solution was added and the mixture was extracted three times with CH_2Cl_2. The combined organic layers were washed with water, dried over anhydrous sodium sulfate, filtered and concentrated to give the crude product which was used without further purification.

The crude alcohol was dissolved in PhMe (411 mL), $TsOH.H_2O$ (1.57 g, 8.23 mmol, 0.100 equiv.) was added and the reaction was heated under reflux for 30 minutes. On cooling to room temperature, powdered KOH (462 mg, 8.23 mmol, 0.100 equiv.) and stirring was continued for 10 minutes. DDQ (24.2 g, 107 mmol, 1.30 equiv.) was added and the reaction was heated at 40 °C for 2 hours and cooled to room temperature. The solvent was removed *in vacuo* and the residue was suspended in 1:4 CH_2Cl_2 – petroleum ether 40-60 and filtered through a short silica plug eluting with the same solvent mixture. The solvent was removed *in vacuo* to give the crude product which was used without further purification.

The crude alkene was dissolved in CH_2Cl_2 (411 mL) and cooled to 0 °C. BBr_3 (9.50 mL, 24.7 g, 99.0 mmol, 1.20 equiv.) was added dropwise over 5 minutes and the reaction was allowed to warm to room temperature and stirred for 16 hours. The reaction was cooled to 0 °C and quenched by dropwise addition of water. The resulting biphasic solution was extracted three times with CH_2Cl_2 and the combined organic layers were washed with water, dried over anhydrous sodium sulfate, filtered and concentrated to give the crude product which was recrystallised from hexane-EtOAc to give give 1-bromo-3-methoxynaphthalene (**56**) (12.9 g, 70%) as a yellow solid.

m.p. = 91-92 °C (*n*-hexane/EtOAc)

IR (film) ν_{max}/cm^{-1}: 3173, 2944, 2914, 1599, 1566, 1243, 1117, 1078, 1041, 1015, 855, 807.

^{1}H NMR (400 MHz, $CDCl_3$) δ = 8.14 (d, J = 7.4 Hz, 1H), 7.66 (d, J = 7.6 Hz, 1H), 7.55–7.35 (m, 3H, H_7), 7.14 (d, J = 2.4 Hz, 1H), 5.38 (s, 1H).

^{13}C NMR (101 MHz, $CDCl_3$) δ = 153.1, 135.3, 127.7, 127.4, 127.1, 127.0, 125.1, 123.8, 122.0), 109.9.

HRMS (ESI^+) $C_{10}H_5O^{79}Br$ $[M+H]^+$ requires 220.96075; found 220.96065, Δ −0.5 ppm.

Data in agreement with literature reported[8]

4,4'-Dibromo-[1,1'-binaphthalene]-2,2'-diol (41)

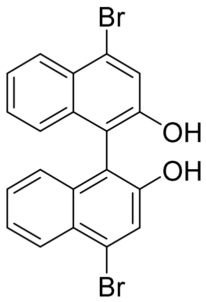

4-Bromonaphthalen-2-ol (**56**) (12.9 g, 58.0 mmol, 1.00 equiv.) was dissolved in CH_2Cl_2 (400 mL) and $[Cu(TMEDA)OHCl)]_2$ (269 mg, 0.580 mmol, 1 mol%) was added. The reaction was stirred for 16 hours at room temperature open to air. The reaction mixture was filtered through a short silica plug eluting with CH_2Cl_2. Evaporation of the solvent gave 4,4'-dibromo-[1,1'-binaphthalene]-2,2'-diol (**41**) (12.9 g, 100%) as an off-white solid.

m.p. = 212-213°C (CH_2Cl_2).

IR (film) ν_{max}/cm^{-1}: 3509, 3464, 1573, 1371, 1217, 1189, 1175, 1148, 1131, 933, 871, 758.

^{1}H NMR (400 MHz, $CDCl_3$) δ = 8.29 (d, J = 8.8 Hz, 2H), 7.74 (s, 2H), 7.49 (ddd, J = 8.3, 6.8, 1.2 Hz, 2H), 7.36 (ddd, J = 8.2, 6.8, 1.3 Hz, 2H), 7.14 (d, J = 8.4 Hz, 2H), 5.04 (s, 2H, OH).

^{13}C NMR (101 MHz, $CDCl_3$) δ = 152.4, 134.0, 128.6, 128.3, 128.0, 126.1, 125.7, 124.6, 122.1, 110.6.

HRMS (ESI$^-$) $C_{20}H_{12}O_2{}^{79}Br_2$ $[M-H]^-$ requires 442.91108; found 442.91085, Δ -0.2 ppm.

Data in agreement with literature reported[10]

4,4'-Dibromo-2'-methoxy-[1,1'-binaphthalen]-2-ol (38)

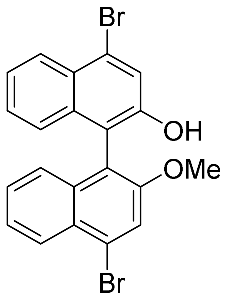

4,4'-Dibromo-[1,1'-binaphthalene]-2,2'-diol (**41**) (12.9 g, 29.0 mmol, 1.00 equiv.) was dissolved in acetone (230 mL) and K_2CO_3 (5.20 g, 37.7 mmol, 1.30 equiv.) followed by iodomethane (1.99 mL, 4.53 g, 31.9 mmol, 1.10 equiv.) were added. The reaction was heated under reflux for 16 hours. Upon cooling to room temperature, the solvent was removed *in vacuo*. The residue was partitioned between water and CH_2Cl_2 and the phases were separated. The aqueous layer was extracted twice with CH_2Cl_2 and the organic layers were combined, dried over anhydrous sodium sulfate, filtered and concentrated to give the crude product. Purification *via* flash column chromatography (1:1 CH_2Cl_2 – petroleum ether) gave 4,4'-dibromo-2'-methoxy-[1,1'-binaphthalen]-2-ol (**38**) (6.42 g, 47%) as an off-white solid.

m.p. = 83-84 °C (CH_2Cl_2/petroleum ether 40-60)

IR (film) ν_{max}/cm^{-1} 3511, 3405, 3394, 3383, 3360, 3352, 3333, 1581, 1499, 1316, 1258, 1241, 1211, 1193, 1169, 1149, 1131, 1098, 1060, 1027, 946, 912, 842, 755, 744.

^{1}H NMR ($CDCl_3$, 400 MHz) δ = 8.32–8.24 (m, 2H), 7.82 (s, 1H), 7.71 (s, 1H), 7.49 (ddd, *J* = 8.4, 6.8, 1.2 Hz, 1H), 7.43 (ddd, *J* = 8.3, 6.9, 1.2 Hz, 1H), 7.33 (ddd, *J* = 8.1, 6.7, 1.2 Hz, 1H), 7.27 (ddd, *J* = 8.2, 5.6, 1.2 Hz, 1H), 7.19 (br. d, *J* = 8.5 Hz, 1H), 7.04 (br. d, *J* = 8.7 Hz, 1H), 4.89–4.84 (m, 1H), 3.81 (s, 3H).

^{13}C NMR ($CDCl_3$, 121 MHz) δ = 155.6, 151.1, 134.6, 134.4, 128.4, 128.2, 127.9, 127.7, 127.5, 127.5, 125.9, 125.8, 125.3, 125.2, 124.8, 124.2, 121.8, 118.2, 114.9, 114.7, 57.0.

HRMS (ESI$^-$) $C_{21}H_{14}O_2{}^{79}Br_2$ [M-H]$^-$ requires 456.92673; found 456.92651, Δ +0.1 ppm.

4-Bromo-2'-methoxy-[1,1'-binaphthalen]-2-ol (28)

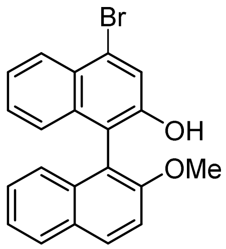

4,4'-Dibromo-2'-methoxy-[1,1'-binaphthalen]-2-ol (**38**) (3.66 g, 8.00 mmol, 1.00 equiv.) was dissolved in THF (80 mL) and cooled to -78 °C. ^{n}BuLi (2.5 M in hexane, 6.40 mL, 16.0 mmol, 2.00 equiv.) was added by syringe pump over 1 hour after which time the reaction was stirred for a further 1 hour at -78 °C. The reaction was quenched by dropwise addition of water. After warming to room temperature, saturated ammonium chloride solution was added. EtOAc was added and the phases were separated. The aqueous phase was extracted twice with EtOAc and the combined organic layers were washed with brine, dried over anhydrous sodium sulfate, filtered and concentrated to give the crude product which was purified by column chromatography (4:1 petroleum ether 40-60 – EtOAc) to give 7-bromo-2'-methoxy-[1,1'-binaphthalen]-2-ol (**28**) (2.09 g, 69%).

m.p. = 171-172 °C (EtOAc/petroleum ether 40-60).

IR (film) ν_{max}/cm^{-1}: 3457, 3435, 3424, 3416, 1588, 1261, 1247, 1209, 1173, 1149, 806, 745.

^{1}H NMR (400 MHz, $CDCl_3$) δ = 8.16 (d, J = 8.7 Hz, 1H), 7.97 (d, J = 9.1 Hz, 1H), 7.81 (d, J = 8.5 Hz, 1H), 7.63 (s, 1H), 7.38 (d, J = 9.1 Hz, 1H), 7.31 (m, 2H), 7.23–7.15 (m, 2H), 7.06 (d, J = 8.5 Hz, 1H), 6.97 (d, J = 7.7 Hz, 1H), 4.84 (s, 1H), 3.72 (s, 3H).

^{13}C NMR (101 MHz, $CDCl_3$) δ = 155.9, 151.0, 134.5, 133.8, 131.4, 129.4, 128.2, 127.7, 127.5, 127.4, 127.2, 125.3, 124.7, 124.6, 124.3, 123.7, 121.7, 115.3, 114.4, 113.6, 56.6.

HRMS (ESI^{+}) $C_{21}H_{13}O_2Br$ $[M+H]^{+}$ requires 377.01827; found 377.01785, Δ -1.1 ppm.

2',4-Dimethoxy-[1,1'-binaphthalen]-2-ol (29)

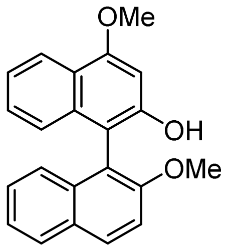

4-Bromo-2'-methoxy-[1,1'-binaphthalen]-2-ol (**28**) (379 mg, 1.00 mmol, 1.00 equiv.) was dissolved in DMF (10 mL) then CuCl (279 mg, 3.00 mmol, 3.00 equiv.) and NaOMe (4.4 M in MeOH, 2.70 mL, 12.0 mmol, 12.0 equiv.) were added sequentially. The reaction was heated at 100 °C for 1 h then cooled to room temperature. Ice water was added followed by hydrochloric acid (3 M). The resulting suspension was extracted 3 times with EtOAc and the combined organic layers were washed with brine, dried over anhydrous sodium sulfate, filtered and concentrated to give the crude product. Purification *via* flash column chromatography (1:1 CH_2Cl_2 – petroleum ether 40-60 to 4:1 CH_2Cl_2 – petroleum ether 40-60) afforded 2',4-dimethoxy-[1,1'-binaphthalen]-2-ol (**29**) as an off-white solid (276 mg, 84%).

m.p. = 171-172 °C (CH_2Cl_2/petroleum ether 40-60).

IR (film) ν_{max}/cm^{-1}: 3490, 3430, 2961, 2938, 1619, 1591, 1388, 1263, 1247, 1230, 1174, 1115, 763.

^{1}H NMR (400 MHz, $CDCl_3$) δ = 8.17 (d, *J* = 8.4 Hz, 1H), 7.93 (d, *J* = 9.1 Hz, 1H), 7.80 (d, *J* = 8.1 Hz, 1H), 7.37 (d, *J* = 9.1 Hz, 1H), 7.27 (ddd, *J* = 8.0, 6.4, 1.6 Hz, 1H), 7.23–7.10 (m, 4H), 6.90 (d, *J* = 8.3 Hz, 1H), 6.66 (s, 1H), 4.85 (s, 1H), 3.98 (s, 3H), 3.71 (s, 3H).

^{13}C NMR (101 MHz, $CDCl_3$) δ = 156.9, 156.3, 151.5, 134.5, 134.3, 130.9, 129.5, 128.1, 127.2, 127.1, 125.1, 124.5, 124.1, 122.6, 122.2, 121.6, 115.4, 113.9, 106.9, 96.5, 56.7, 55.7.

HRMS (ESI$^+$) $C_{22}H_{18}O_3$ $[M+H]^+$ requires 331.13287; found 31.13272, Δ −0.5 ppm.

2'-Methoxy-4-methyl-[1,1'-binaphthalen]-2-ol (26)

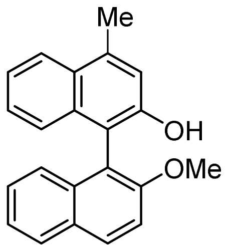

4-Bromo-2'-methoxy-[1,1'-binaphthalen]-2-ol (**28**) (379 mg, 1.00 mmol, 1.00 equiv.) and Pd(dppf)Cl_2.CH_2Cl_2 (82.6 mg, 0.100 mmol, 10 mol%) were dissolved in THF (10 mL) and MeMgCl solution (3 M in Et_2O, 1.7 mL, 5.00 mmol, 5.00 equiv.) was added. The resulting solution was heated under reflux for 16 hours. On cooling to room temperature, saturated ammonium chloride solution was added and phases were separated. The aqueous phase was extracted twice with EtOAc and the combined organic layers were washed with brine, dried over anhydrous sodium sulfate, filtered and concentrated to give the crude product which was purified by column chromatography (9:1 petroleum ether 40-60 – EtOAc) to give 2'-methoxy-4-methyl-[1,1'-binaphthalen]-2-ol (**28**) (249 mg, 79%) as an off-white solid.

m.p. = 137-138 °C (EtOAc/petroleum ether 40-60).

IR (film) ν_{max}/cm^{-1}: 3504, 3434, 3388, 1592, 1508, 1262, 1247, 1223, 1183, 1093, 1065, 804, 754, 744, 666, 643

^{1}H NMR (400 MHz, $CDCl_3$) δ = 7.96–7.87 (m, 2H), 7.79 (d, J = 7.3 Hz, 1H), 7.37 (d, J = 9.1 Hz, 1H), 7.26 (m, 2H), 7.19–7.08 (m, 4H), 6.98 (d, J = 8.6 Hz, 1H), 4.78 (br. s, 1H), 3.70 (s, 3H), 2.69 (s, 3H).

^{13}C NMR (101 MHz, $CDCl_3$) δ = 156.1, 150.7, 136.5, 134.2, 134.0, 131.0, 129.4, 128.5, 128.1, 127.3, 126.2, 125.4, 125.1, 124.4, 124.1, 123.1, 118.3, 115.6, 113.8, 113.0, 56.7, 19.6.

HRMS (ESI^+) $C_{22}H_{18}O_2$ $[M+H]^+$ requires 315.13796; found 315.13791, Δ −0.2 ppm.

2'-Methoxy-4-phenyl-[1,1'-binaphthalen]-2-ol (27)

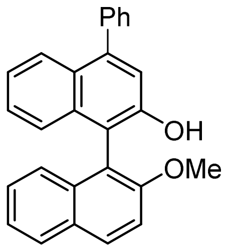

4-Bromo-2'-methoxy-[1,1'-binaphthalen]-2-ol (**28**) (379 mg, 1.00 mmol, 1.00 equiv.), phenyl boronic acid (144 mg, 1.20 mmol, 1.20 equiv.) and $Pd(PPh_3)_4$ (57.8 mg, 0.0500 mmol, 0.0500 equiv.) were charged to a flask fitted with a water-cooled condenser. The flask was evacuated and backfilled with argon three times. 1,2-Dimethoxyethane (Ar sparged, 10 mL) was added followed by aqueous Na_2CO_3 solution (Ar sparged, 2 M, 1.25 mL, 2.50 equiv.). The condenser was fitted with an argon balloon and heated at 90 °C for 16 hours. After cooling to room temperature, 1 M HCl solution was added and the solution was extracted three times with CH_2Cl_2, the combined organic layers were dried over anhydrous sodium sulfate, filtered and concentrated to give the crude product which was purified by column chromatography (9:1 petroleum ether 40-60 – EtOAc) to give 2'-methoxy-4-phenyl-[1,1'-binaphthalen]-2-ol (**27**) (314 mg, 84%) as a white solid.

m.p. = 103-104 °C (EtOAc/petroleum ether 40-60).

IR (film) ν_{max}/cm^{-1}: 3657, 2980, 2888, 1382, 1261, 1169, 1142, 1088, 1060, 945, 809, 762, 749, 702.

^{1}H NMR (400 MHz, $CDCl_3$) δ = 7.96 (d, J = 9.0 Hz, 1H), 7.85–7.75 (m, 2H), 7.53 (dt, J = 6.1, 1.4 Hz, 2H), 7.47–7.34 (m, 4H), 7.29 (ddd, J = 8.1, 6.3, 1.7 Hz, 1H), 7.25–7.09 (m, 5H), 7.03 (dd, J = 8.1, 1.7 Hz, 1H), 4.87 (s, 1H), 3.74 (s, 3H).

^{13}C NMR (101 MHz, $CDCl_3$) δ = 156.1, 150.6, 142.1, 140.5, 134.2, 134.1, 131.1, 130.2, 129.5, 128.3, 128.2, 127.6, 127.4, 127.4, 126.4, 126.3, 125.2, 125.0, 124.2, 123.3, 118.5, 115.3, 114.6, 113.8, 56.7.

HRMS (ESI^+) $C_{27}H_{20}O_2$ $[M+H]^+$ requires 377.15361; found 377.15358, Δ −0.1 ppm.

2'-Methoxy-4,4'-diphenyl-[1,1'-binaphthalen]-2-ol (42)

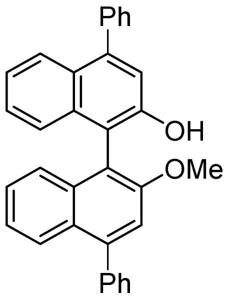

4,4'-Dibromo-2'-methoxy-[1,1'-binaphthalen]-2-ol (**38**) (458 mg, 1.00 mmol, 1.00 equiv.), phenyl boronic acid (293 mg, 2.40 mmol, 2.40 equiv.) and $Pd(PPh_3)_4$ (57.8 mg, 0.05 mmol, 5 mol%) were charged to a flask fitted with a water-cooled condenser. The flask was evacuated and backfilled with argon three times. 1,2-Dimethoxyethane (Argon sparged, 10 mL) was added followed by aqueous Na_2CO_3 solution (Argon sparged, 2 M, 2.50 mL, 5.00 equiv.). The flask was fitted with an argon balloon and heated at 90 °C for 16 hours. After cooling to room temperature, 1 M HCl solution was added and the solution was extracted three times with CH_2Cl_2, the combined organic layers were dried over anhydrous sodium sulfate, filtered and concentrated to give the crude product which was purified by column chromatography (9:1 petroleum ether 40-60 – EtOAc) to give 2'-methoxy-4,4'-diphenyl-[1,1'-binaphthalen]-2-ol (**42**) (384 mg, 85%) as a white solid.

m.p. = 118-120 °C (EtOAc – petroleum ether 40-60)

IR (film) ν_{max}/cm^{-1}: 3526, 3407, 3056, 2970, 2935, 1587, 1344, 1224, 1175, 1144, 763, 702, 658.

^{1}H NMR ($CDCl_3$, 400 MHz) δ = 7.87–7.82 (m, 2H), 7.58–7.35 (m, 11H), 7.29–7.11 (m, 7H), 4.95 (s, 1H), 3.77 (s, 3H).

^{13}C NMR ($CDCl_3$, 121 MHz) δ =155.4, 150.7, 143.6, 142.2, 140.5, 140.5, 134.6, 134.3, 130.2, 130.1, 128.5, 128.3, 127.8, 127.8, 127.6, 127.4, 127.3, 126.5, 126.4, 126.4, 125.4, 125.3, 124.3, 123.3, 118.5, 114.9, 114.7, 114.6, 56.7.

HRMS (ESI^+) $C_{33}H_{24}O_2$ $[M+H]^+$ requires 453.18491; found 453.18411, Δ −1.8 ppm.

2'-Methoxy-4,4'-dimethyl-[1,1'-binaphthalen]-2-ol (43)

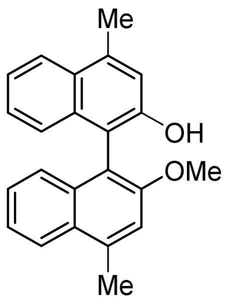

4,4'-dibromo-2'-methoxy-[1,1'-binaphthalen]-2-ol (**38**) (687 mg, 1.50 mmol, 1.00 equiv.) and Pd(dppf)Cl_2.CH_2Cl_2 (122 mg, 0.15 mmol, 10 mol%) were dissolved in THF (15 mL) and MeMgCl solution (3 M in Et_2O, 5.00 mL, 15.0 mmol, 10.0 equiv.) was added. The resulting solution was heated under reflux for 16 hours. On cooling to room temperature, saturated ammonium chloride solution was added and phases were separated. The aqueous phase was extracted twice with EtOAc and the combined organic layers were washed with brine, dried over anhydrous sodium sulfate, filtered and concentrated to give the crude product which was purified by column chromatography (9:1 petroleum ether 40-60 – EtOAc) to give 2'-methoxy-4,4'-dimethyl-[1,1'-binaphthalen]-2-ol (**43**) (224 mg, 45%) as an off-white solid.

m.p. = 178-180 °C (EtOAc – petroleum ether 40-60)

IR (film) ν_{max}/cm^{-1}: 3519, 2980, 1591, 1342, 1264, 1220, 1179, 1171, 1136, 1111, 1058, 999, 869, 758

^{1}H NMR ($CDCl_3$, 400 MHz) δ = 8.13–7.92 (m, 2H), 7.48–7.21 (m, 7H), 7.15–7.09 (m, 1H), 4.92 (s, 1H), 3.83 (s, 3H), 2.89 (s, 3H), 2.82 (s, 3H).

^{13}C NMR ($CDCl_3$, 121 MHz) δ = 155.6, 150.8, 137.9, 136.4, 134.3, 134.1, 128.7, 128.5, 127.0, 126.1, 125.6, 125.5, 124.4, 124.3, 124.0, 123.0, 118.2, 115.0, 113.4, 113.2, 56.7, 20.1, 19.6.

HRMS (ESI$^+$) $C_{23}H_{20}O_2$ $[M+H]^+$ requires 329.15361; found 329.15279, Δ −1.4 ppm.

4.5 BINOLs Prepared by Other Synthetic Routes

2'-Methoxy-6'-nitro-[1,1'-binaphthalen]-2-ol was prepared following a reported method.[7]

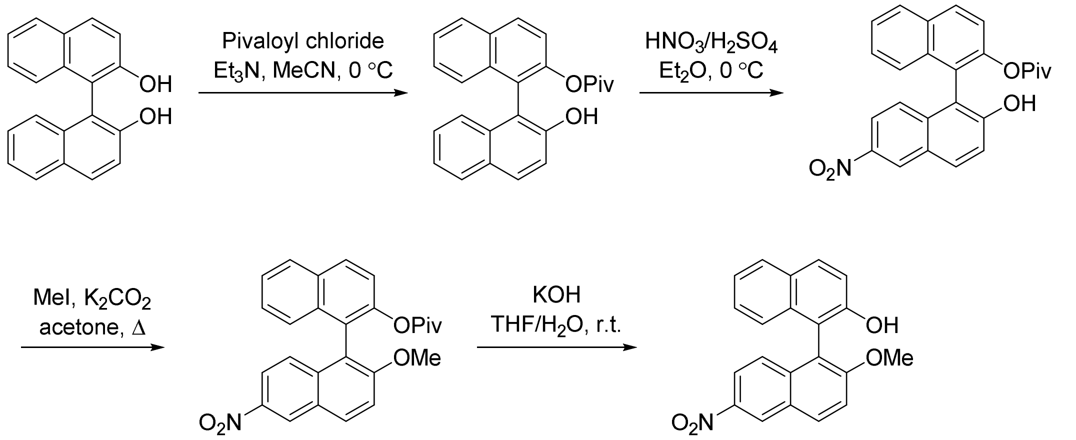

BINOLs **1**, **9** and **10** were prepared by alkylation of racemic [1,1'-binaphthalene]-2,2'-diol.

BINOLs **12**, **17** and **25** were prepared by deprotection of racemic benzylated compounds.[11]

BINOL **31** was prepared by dimerization of commercially available 7-methoxy-2-naphthol, followed by mono-alkylation.

2'-Methoxy-[1,1'-binaphthalen]-2-ol (1)

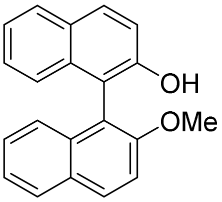

[1,1'-binaphthalene]-2,2'-diol (5.00 g, 17.4 mmol, 1.00 equiv.) and K_2CO_3 (3.14 g, 22.7 mmol, 1.30 equiv.) were suspended in acetone (140 mL) and iodomethane (2.73 g, 1.20 mL, 19.2 mmol, 1.10 equiv) was added. The resulting suspension was heated under reflux for 16 hours after which time the reaction was cooled to room temperature. The solvent was removed by evaporation and the residue was partitioned between water and CH_2Cl_2. The phases were separated and the aqueous layer was extracted twice with CH_2Cl_2. The combined organic layers were dried over anhydrous sodium sulfate, filtered and concentrated to give the crude product. Purification *via* flash column chromatography (1:1 CH_2Cl_2 – petroleum ether 40-60 to 3:1 CH_2Cl_2 – petroleum ether 40-60) afforded 2'-methoxy-[1,1'-binaphthalen]-2-ol (**1**) (2.27 g, 43%) as a white solid.

m.p. = 151-153 °C (CH_2Cl_2/petroleum ether 40-60)

^{1}H NMR (500 MHz, $CDCl_3$) δ = 8.06 (d, *J* = 9.1 Hz, 1H), 7.95 (d, *J* = 9.2 Hz, 1H), 7.93 (d, *J* = 9.3 Hz, 1H), 7.91 (d, *J* = 10.6 Hz, 1H), 7.49 (d, *J* = 9.1 Hz, 1H), 7.42 (d, *J* = 1.9 Hz, 2H), 7.36 (t, *J* = 7.4 Hz, 1H), 7.32 (t, *J* = 7.7 Hz, 1H), 7.27 (t, *J* = 8.3 Hz, 1H), 7.24 (d, *J* = 9.0 Hz, 1H), 7.12 (d, *J* = 8.4 Hz, 1H), 5.00 (s, 1H), 3.81 (s, 3H).

^{13}C NMR (101 MHz, $CDCl_3$) δC = 156.1, 151.3, 134.1, 133.9, 131.1, 129.9, 129.5, 129.2, 128.2, 127.4, 126.5, 125.0, 124.9, 124.3, 123.3, 117.6, 115.4, 115.1, 113.9, 56.7

Data in agreement with literature[12]

2'-Isopropoxy-[1,1'-binaphthalen]-2-ol (9)

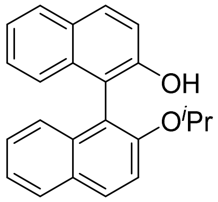

Following a modified literature procedure,[10] to a solution of [1,1'-binaphthalene]-2,2'-diol (1.43 g, 5.00 mmol, 1.00 equiv), triphenylphosphine (1.31 g, 5.00 mmol, 1.00 equiv) and isopropyl alcohol (1.50 g, 1.91 mL, 25.0 mmol, 5.00 equiv) in tetrahydrofuran (50 mL) was added diisopropyl azodicarboxylate (1.01 g, 0.98 mL, 5.00 mmol, 1.00 equiv) dropwise. The resulting mixture was stirred at room temperature for 24 h before being concentrated *in vacuo*. The crude product was purified by flash column chromatography (3:2 CH_2Cl_2 – petroleum ether 40-60) to give 2'-Isopropoxy-[1,1'-binaphthalen]-2-ol (**9**) (910 mg, 55%) as a white solid.

^{1}H NMR (500 MHz, $CDCl_3$) δ 8.00 (d, *J* = 9.0 Hz, 1H), 7.93-7.88 (m, 2H), 7.86 (d, *J* = 8.1 Hz, 1H), 7.45 (d, *J* = 9.0 Hz, 1H), 7.40-7.33 (m, 2H), 7.31 (ddd, *J* = 0.8, 7.2, 7.7 Hz, 1H), 7.29-7.24 (m, 1H), 7.21 (ddd, *J* = 1.3, 6.9, 8.3 Hz, 1H), 7.18 (d, *J* = 8.5 Hz, 1H), 7.07 (d, *J* = 8.4 Hz, 1H), 5.08 (s, 1H), 4.44 (sept, *J* = 6.1 Hz, 1H), 1.13 (d, *J* = 6.1 Hz, 3H), 0.99 (d, *J* = 6.1 Hz, 3H).

^{13}C NMR (126 MHz, $CDCl_3$) δ 154.8, 151.4, 134.4, 134.0, 130.8, 129.9, 129.7, 129.2, 128.2, 128.1, 127.2, 126.3, 125.4, 125.3, 124.5, 123.2, 118.2, 118.0, 117.7, 115.7, 72.7, 22.4, 22.3.

HRMS (ESI$^+$): $C_{23}H_{21}O_2^+$ $[M+H]^+$: requires 329.15361, found: 329.15366, Δ +0.2 ppm.

Data in agreement with literature[13]

2'-(Benzyloxy)-[1,1'-binaphthalen]-2-ol (10)

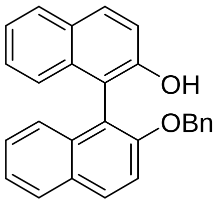

[1,1'-binaphthalene]-2,2'-diol (2.00 g, 6.98 mmol, 1.00 equiv.) and K_2CO_3 (1.25 g, 9.08 mmol, 1.30 equiv.) were suspended in acetone (56.0 mL) and benzyl bromide (1.31 g, 0.91 mL, 7.68 mmol, 1.10 equiv) was added. The resulting suspension was heated under reflux for 7 hours after which time the reaction was cooled to room temperature. The solvent was removed by evaporation and the residue was partitioned between water and CH_2Cl_2. The phases were separated and the aqueous layer was extracted twice with CH_2Cl_2. The combined organic layers were dried over anhydrous sodium sulfate, filtered and concentrated to give the crude product. Purification *via* flash column chromatography (1:2 CH_2Cl_2 – petroleum ether 40-60 to 1:1 CH_2Cl_2 – petroleum ether 40-60) afforded 2'-(benzyloxy)-[1,1'-binaphthalen]-2-ol (**10**) (1.27 g, 48%) as a light brown solid.

m.p. = 108-110 °C (CH_2Cl_2/petroleum ether 40-60)

^{1}H NMR (500 MHz, $CDCl_3$) δ 7.99 (d, *J* = 9.0 Hz, 1H), 7.96 (d, *J* = 8.9 Hz, 1H), 7.91 (t, *J* = 7.3 Hz, 1H), 7.48 (d, *J* = 9.1 Hz, 1H), 7.43-7.38 (m, 2H), 7.37-7.24 (m, 3H), 7.23-7.18 (m, 3H), 7.13 (d, *J* = 8.5 Hz, 1H), 7.10-7.04 (m, 2H), 5.16-5.06 (m, 2H), 5.00 (s, 1H).

^{13}C NMR (126 MHz, $CDCl_3$) δ 155.0, 151.4, 137.0, 134.2, 133.9, 130.9, 129.9, 129.8, 129.3, 128.2, 128.2, 127.8, 127.4, 127.0, 126.5, 125.1, 125.1, 124.5, 123.4, 117.6, 116.9, 116.0, 115.2, 71.2

Data in agreement with literature[13]

2'-Hydroxy-[1,1'-binaphthalen]-2-yl pivalate (59)

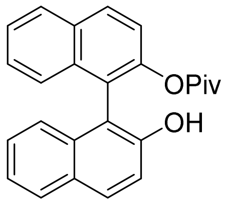

(±)-BINOL (5.72 g, 20.0 mmol, 1.00 equiv.) was dissolved in MeCN and Et_3N (8.40 mL, 11.6 g, 60.0 mmol, 3.00 equiv.) was added. The resulting solution was cooled to 0 °C and pivaloyl chloride (2.40 mL, 2.44 g, 20.2 mmol, 1.01 equiv.) was added dropwise. The reaction was warmed to room temperature and stirred for an additional 5 hours. Et_2O was added and the mixture was washed with aqueous HCl (1 M), aqueous $NaHCO_3$ then brine. The organic phase was dried over anhydrous sodium sulfate, filtered and concentrated *in vacuo* to give the crude product which was purified *via* flash column chromatography (4:1 petroleum ether 40-60 – EtOAc) to give 2'-hydroxy-[1,1'-binaphthalen]-2-yl pivalate (**59**) (4.00 g, 54%) as a white solid.

m.p. = 118-120 °C (EtOAc/petroleum ether 40-60).

^{1}H NMR (400 MHz, $CDCl_3$) δ = 8.11 (d, J = 8.8 Hz, 1H), 8.01 (d, J = 8.3 Hz, 1H), 7.92 (d, J = 8.9 Hz, 1H), 7.86 (d, J = 8.0 Hz, 1H), 7.54 (ddd, J = 8.1, 5.9, 2.1 Hz, 1H), 7.42 (d, J = 8.9 Hz, 1H), 7.40–7.33 (m, 4H), 7.29 (ddd, J = 8.2, 6.6, 1.4 Hz, 1H), 7.10 (dd, J = 8.4, 1.1 Hz, 1H), 5.19 (s, 1H), 0.82 (s, 9H).

^{13}C NMR (101 MHz, $CDCl_3$) δ = 177.8, 151.8, 148.3, 133.6, 133.5, 132.2, 130.7, 130.3, 129.0, 128.3, 127.9, 127.5, 126.7, 126.2, 125.6, 124.6, 123.5, 123.0, 121.8, 118.2, 114.2, 38.8, 26.5.

LRMS (ESI$^-$) $C_{25}H_{22}O_3$ $[M-H]^-$ requires 369.1; found 369.0.

Data in agreement with literature[7]

2'-Hydroxy-6'-nitro-[1,1'-binaphthalen]-2-yl pivalate (60)

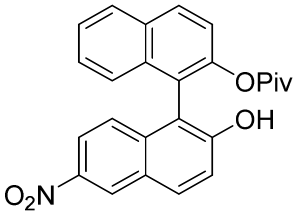

2'-Hydroxy-[1,1'-binaphthalen]-2-yl pivalate (**59**) (2.00 g, 5.40 mmol, 1.00 equiv.) was dissolved in Et_2O (54 mL) and the resulting solution was cooled to 0 °C. HNO_3 (conc., 3.35 mL) followed by H_2SO_4 (conc., 1.35 mL) were added. After 2 hours the mixture was poured into ice/water and diluted with Et_2O. The organic phase was washed three times with water, dried over anhydrous sodium sulfate, filtered and concentrated to give the crude product. The crude oil was triturated with 1:1 CH_2Cl_2 – petroleum ether 40-60 to give 2'-hydroxy-6'-nitro-[1,1'-binaphthalen]-2-yl pivalate (**60**) (1.16 g, 52%) was a yellow solid.

m.p. = 204-206 °C (CH_2Cl_2/petroleum ether 40-60).

IR (film) v_{max}/cm^{-1}: 3374, 1737, 1618, 1530, 1494, 1479, 1331, 1292, 1283, 1128, 741.

^{1}H NMR (400 MHz, DMSO-*d*6) δ = 10.51 (br. s, 1H), 8.97 (d, *J* = 2.5 Hz, 1H), 8.30 (d, *J* = 9.1 Hz, 1H), 8.12 (d, *J* = 8.9 Hz, 1H), 8.08 (d, *J* = 8.4 Hz, 1H), 7.95 (dd, *J* = 9.4, 2.5 Hz, 1H), 7.51 (m, 3H), 7.38 (ddd, *J* = 8.2, 6.8, 1.3 Hz, 1H), 7.16 (d, *J* = 8.4, 1H), 7.02 (d, *J* = 9.4 Hz, 1H), 0.70 (s, 9H).

^{13}C NMR (101 MHz, DMSO-*d*6) δ = 175.9, 157.6, 147.4, 142.8, 137.3, 133.3, 132.8, 131.9, 129.7, 128.8, 127.3, 126.5, 126.1, 126.1, 125.6, 125.5, 124.4, 122.8, 120.8, 119.9, 114.6, 26.5.

LRMS (ESI^-) $C_{25}H_{21}NO_5$ $[M-H]^-$ requires 414.1; found 414.0.

Data in agreement with literature[7]

2'-Methoxy-6'-nitro-[1,1'-binaphthalen]-2-ol (11)

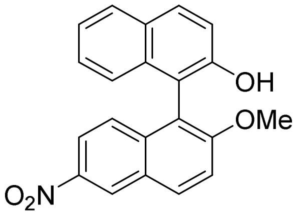

2'-Hydroxy-6'-nitro-[1,1'-binaphthalen]-2-yl pivalate (**60**) (1.20 g, 2.89 mmol, 1.00 equiv.) was dissolved in acetone (30 mL) and K_2CO_3 (798 mg, 5.78 mmol, 2.00 equiv.) followed by iodomethane (0.27 mL, 616 mg, 4.33 mmol, 1.50 equiv.) were added. The reaction was heated under reflux for 24 hours then cooled to room temperature. The crude mixture was concentrated to approximately 10 mL before addition of water (50 mL). A yellow solid was precipitated and collected by filtration. The crude solid was dissolved in CH_2Cl_2 and the resulting solution was washed with brine, dried over anhydrous sodium sulfate , filtered and concentrated to give the crude product which was used without further purification.

The crude methylated BINOL was dissolved in 3:1 THF/water (14 mL) and KOH (196 mg) was added. The reaction was stirred for 6 hours at 70 °C, cooled to room temperature then partially concentrated *in vacuo* to remove THF. 1 M HCl was added and the solution was extracted three times with CH_2Cl_2. The combined organic layers were washed 5 times with 1 M HCl, dried over anhydrous sodium sulfate, filtered and concentrated to give the crude product. Purification *via* flash column chromatography (4:1 CH_2Cl_2 – petroleum ether 40-60) gave 2'-methoxy-6'-nitro-[1,1'-binaphthalen]-2-ol (**11**) (582 mg, 55%).

m.p. = 234-236 °C (CH_2Cl_2/petroleum ether 40-60).

IR (film) ν_{max}/cm^{-1}: 3058, 2934, 2840, 1597, 1493, 1472, 1467, 1327, 1267, 1244, 1198, 1142, 1128, 1093, 1081, 1047, 910, 812, 798, 740.

^{1}H NMR (400 MHz, $CDCl_3$) δ = 8.86 (d, J = 2.3 Hz, 1H), 8.25 (d, J = 9.1 Hz, 1H), 8.01 (dd, J = 9.3, 2.4 Hz, 1H), 7.94 (d, J = 8.9 Hz, 1H), 7.89 (d, J = 8.1 Hz, 1H), 7.64 (d, J = 9.2 Hz, 1H), 7.38–7.30 (m, 2H), 7.30–7.21 (m, 3H), 6.94 (d, J = 8.4 Hz, 1H), 3.88 (s, 3H).

^{13}C NMR (101 MHz, $CDCl_3$) δ = 158.9, 151.3, 144.1, 137.0, 133.4, 133.3, 130.4, 129.2, 128.4, 127.5, 126.8, 126.6, 125.2, 124.3, 123.6, 120.6, 117.6, 116.3, 115.3, 113.8, 56.7.

HRMS (ESI^-) $C_{21}H_{15}NO_4$ $[M-H]^-$ requires 344.09283; found 344.09256, Δ −0.8 ppm.

Data in agreement with literature[7]

2'-Methoxy-6'-methyl-[1,1'-binaphthalen]-2-ol (12)

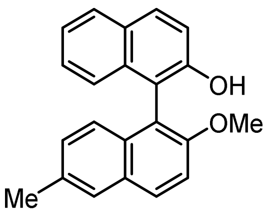

Ammonium formate (473 mg, 7.50 mmol, 6.00 equiv.) and Pd/C (10% wt, 40 mg) were added to a solution of 2'-Methoxy-6'-methyl-1,1'-binaphthalen]-2-ol[11] (505 mg, 1.25 mmol) in 9:1 ethyl acetate/methanol in a sealed pressure tube. The suspension was then stirred at 50 °C for 24 h. after cooling to room temperature, the reaction mixture was filtered through celite, washing the filtercake with ethyl acetate, and the filtrate was subsequently washed twice with water and concentrated under reduced pressure. Purification of the residue *via* column chromatography eluting with a gradient from 1:2 dichloromethane/petrol 40-60 to 1:1 dichloromethane/petrol 40-60 afforded 2'-methoxy-6'-methyl-[1,1'-binaphthalen]-2-ol (**12**) (379 mg, 97 %) as a white solid.

m.p. = 81-82 °C (dichloromethane/petrol 40-60).

^{1}H NMR (400 MHz, $CDCl_3$) δ = 7.97 (d, *J* = 9.1 Hz, 1H), 7.92 (d, *J* = 8.9 Hz, 1H), 7.88 (dd, *J* = 8.1, 0.9 Hz, 1H), 7.69 (d, *J* = 1.8, Hz, 1H), 7.45 (d, *J* = 9.1 Hz, 1H), 7.38 (d, *J* = 8.9 Hz, 1H), 7.33 (ddd, *J* = 8.1, 6.7, 1.2 Hz, 1H), 7.24 (ddd, *J* = 8.3, 6.8, 1.4 Hz, 1H), 7.14 (dd, *J* = 8.7, 1.7 Hz, 1H), 7.12-7.06 (m, 2H,), 4.97 (br s, 1H, O*H*), 3.79 (s, 3H), 2.49 (s, 3H).

^{13}C NMR (101 MHz, $CDCl_3$) δ = 155.5, 151.3, 133.9, 133.9, 132.3, 130.4, 129.8, 129., 129.7, 129.2, 128.2, 127.2, 126.5, 125.0, 124.9, 123.3, 117.6, 115.4, 115.3, 114.0, 56.8, 21.5.

IR (film) ν_{max}/cm^{-1}: 3490, 3424, 3058, 3017, 2937, 2918, 2839, 1620, 1595, 1515, 1501, 1463, 1379, 1353, 1334, 1300, 1250, 1228, 1147, 1084, 1051.

HRMS (ESI^-): $C_{22}H_{18}O_2$ $[M-H]^-$ requires = 313.1234; found = 313.1233, Δ +0.3 ppm.

7-Fluoro-2'-methoxy-[1,1'-binaphthalen]-2-ol (17)

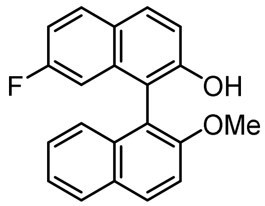

A solution of 2-(benzyloxy)-7-fluoro-2'-methoxy-1,1'-binaphthalene[11] (579 mg, 1.42 mmol) in degassed 1:9 methanol/ethyl acetate (1 mL) was added to palladium on carbon (10 wt %, 42 mg). The reaction mixture was stirred under an atmosphere of hydrogen (balloon) for 24 hours. The reaction mixture was then filtered through a plug of celite which was subsequently washed with ethyl acetate. The filtrate was concentrated under reduced pressure. Purification of the residue *via* column chromatography eluting with a gradient from 1:1 dichloromethane/petrol 40-60 to 2:1 dichloromethane/petrol 40-60 afforded 7-fluoro-2'-methoxy-[1,1'-binaphthalen]-2-ol (**17**) (454 mg, 99 %) as a white solid.

m.p. = 201-202 °C (dichloromethane/petrol 40-60).

^{1}H NMR (400 MHz, $CDCl_3$) δ = 8.06 (d, *J* = 9.1 Hz, 1H), 7.93-7.87 (m, 2H), 7.84 (dd, *J* = 8.9, 5.8 Hz, 1H), 7.48 (d, *J* = 9.1 Hz, 1H), 7.39 (ddd, *J* = 8.1, 6.8, 1.3 Hz, 1H), 7.35-7.28 (m, 2H), 7.20-7.15 (m, 1H), 7.09 (ddd, *J* = 8.9, 8.3, 2.6 Hz, 1H), 6.67 (dd, *J* = 11.3, 2.6 Hz, 1H), 4.99 (br s, 1H), 3.82 (s, 3H).

^{13}C NMR (101 MHz, $CDCl_3$) δ = 161.7 (d, *J* = 244.8 Hz), 156.2, 152.3, 135.2 (d, *J* = 9.4 Hz), 133.9, 131.5, 130.6 (d, *J* = 9.4 Hz), 129.8, 129.6 , 128.4, 127.6 , 126.2 , 124.7, 124.4, 116.8 (d, *J* = 2.5 Hz), 114.9 (d, *J* = 5.3 Hz), 113.8, 113.5 (d, *J* = 25.4 Hz), 108.5 (d, *J* = 22.1 Hz), 56.7.

^{19}F NMR (376 MHz, $CDCl_3$) ? = –114.0.

IR (film) ν_{max}/cm^{-1}: 3492, 3428, 3062, 2840, 1624, 1592, 1508, 1472, 1431, 1378, 1360, 1230, 1200, 1166, 1149, 1135, 1080, 1053, 1021.

HRMS (ESI^-): $C_{21}H_{15}FO_2$ $[M-H]^-$ requires 317.0983; found 317.0981, Δ = –0.6 ppm.

1-(2-Methoxynaphthalen-1-yl)phenanthren-2-ol (25)

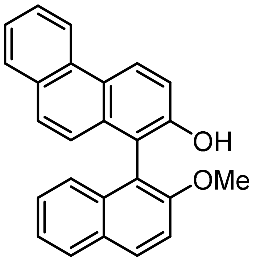

Ammonium formate (260 mg, 4.12 mmol) and Pd/C (10 wt %, 105 mg) were added to a solution of 2-(benzyloxy)-1-(2-Methoxynaphthalen-1-yl)phenanthren-2-ol[11] (303 mg, 0.687 mmol) in 9:1 ethyl acetate/methanol in a sealed pressure tube. The suspension was then stirred at 50 °C for 48 h. after cooling to room temperature, the reaction mixture was filtered through celite, washing the filtercake with ethyl acetate, and the filtrate was subsequently washed twice with water and concentrated under reduced pressure. Purification of the residue *via* column chromatography eluting with a gradient from 1:2 dichloromethane/petrol 40-60 to 1:1 dichloromethane/petrol 40-60 afforded the title compound 1-(2-Methoxynaphthalen-1-yl)phenanthren-2-ol (**25**) (228 mg, 95 %) as a white solid.

m.p. = 123-125 °C (dichloromethane/petrol 40-60).

^{1}H NMR (400 MHz, $CDCl_3$) δ = 8.79 (d, J = 9.0 Hz, 1H), 8.71 (d, J = 8.2 Hz, 1H), 8.08 (d, J = 9.1 Hz, 1H), 7.93 (dd, J = 8.2, 0.9 Hz, 1H), 7.81 (dd, J = 7.8, 1.3 Hz, 1H), 7.67 (ddd, J = 8.4, 7.0, 1.4 Hz, 1H), 7.58-7.47 (m, 4H), 7.40 (ddd, J = 8.1, 6.7, 1.3 Hz, 1H), 7.30 (ddd, J = 8.1, 6.7, 1.3 Hz, 1H), 7.24-7.18 (m, 1H), 7.05 (d, J = 9.1 Hz, 1H), 4.98 (br s, 1H), 3.82 (s, 3H).

^{13}C NMR (101 MHz, $CDCl_3$) δ = 156.1, 152.3, 134.2, 132.6, 131.3, 130.0, 130.8, 129.5, 128.6, 128.3, 127.7, 127.6, 126.8, 125.7, 124.9, 124.9, 124., 124.4, 124.4, 122.4, 117.3, 116.5, 115.6, 113.9, 56.8.

IR (film) ν_{max}/cm^{-1}: 3495, 3424, 3057, 2936, 2839, 1620, 1592, 1529, 1506, 1460, 1431, 1420, 1332, 1306, 1268, 1250, 1214, 1203, 1171, 1146, 1119, 1076, 1057, 1034, 1020.

HRMS (ESI$^-$): $C_{25}H_{18}O_2$ [M-H]$^-$ requires 349.1234; found 349.1233, Δ −0.3.

7,7'-Dimethoxy-[1,1'-binaphthalene]-2,2'-diol (61)

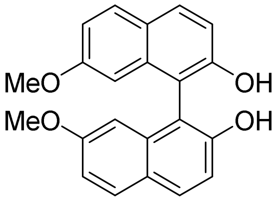

Following a modified literature procedure,[3] to a solution of 7-methoxynaphth-2-ol (1.50 g, 8.61 mmol) in CH_2Cl_2 (15.0 mL) was added Cu-TMEDA complex (400 mg, 0.86 mmol). The mixture was sonicated in air for 30 seconds and then stirred for 16 hours at room temperature under an air atmosphere. The solvent was removed under reduced pressure and the resulting crude mixture was purified by flash column chromatography (1:1 pet ether 40-60/CH_2Cl_2 to CH_2Cl_2) to give the title compound **61** as a yellow-brown solid (826 mg, 2.38 mmol, 55% yield).

^{1}H NMR (400 MHz, $CDCl_3$) δ_H: 7.85 (d, *J* = 8.8 Hz, 2H), 7.77 (d, *J* = 8.9 Hz, 2H), 7.21 (d, *J* = 8.9 Hz, 2H), 7.03 (d, *J* = 8.9 Hz, 2H), 6.47 (s, 2H), 5.08 (s, 2H), 3.57 (s, 6H)

^{13}C NMR (101 MHz, $CDCl_3$) δ_C: 159.2, 153.4, 134.8, 131.2, 130.1, 124.9, 116.1, 115.2, 110.2, 103.2, 55.3

HRMS (ESI$^-$) $C_{22}H_{17}O_4^-$ [M-H]$^-$: requires 345.1132, found: 345.1134, Δ +0.58 ppm.

Data in agreement with literature reported.[14]

2',7,7'-Trimethoxy-[1,1'-binaphthalen]-2-ol (31)

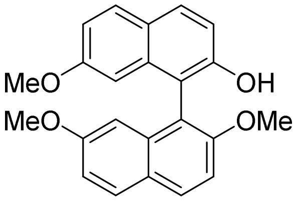

To a solution of **61** (826 mg, 2.38 mmol) in acetone (24 mL) was added K_2CO_3 (493 mg, 3.57 mmol) and methyl iodide (0.22 mL, 3.57 mmol). The resulting suspension was heated at 70 °C for 16 hours and cooled to room temperature. The solvent was removed under reduced pressure followed by addition of water (20 mL) and CH_2Cl_2 (30 mL). The layers were separated and the aqueous layer was extracted with CH_2Cl_2 (20 mL × 3). The combined organic layers were dried over anhydrous $MgSO_4$, filtered and the solvent removed under reduced pressure. The resulting crude mixture was purified by flash column chromatography (2:1 petroleum ether 40-60 – CH_2Cl_2 to 1:1 petroleum ether 40-60 – CH_2Cl_2) to give the title compound **31** as an off-white solid (577 mg, 1.60 mmol, 67% yield).

m.p. = 96-98 °C (CH_2Cl_2/petroleum ether 40-60)

IR (ν_{max}/cm^{-1}, ATR): 3484, 2937, 1621, 1511, 1462, 1262, 1221, 813, 756.

^{1}H NMR (400 MHz, $CDCl_3$) δ_H: 7.97 (d, *J* = 8.8 Hz, 1H, H_4), 7.87-7.75 (m, 3H, H_6, H_{16} and H_{18}), 7.33 (d, *J* = 8.8 Hz, 1H, H_3), 7.23 (d, *J* = 8.5 Hz, 1H, H_{15}), 7.06 (d, *J* = 9.1 Hz, 1H, H_{19}), 7.01 (d, *J* = 8.8 Hz, H_7), 6.51 (s, 1H, H_{21}), 6.43 (s, 1H, H_9), 4.98 (s, 1H, OH), 3.82 (s, 3H, H_{12}), 3.56 (s, 3H, H_{11} or H_{23}), 3.54 (s, 3H, H_{11} or H_{23}).

^{13}C NMR (101 MHz, $CDCl_3$) δ_C: 158.9, 158.2, 156.6, 151.7, 135.3, 134.9, 130.7, 129.8, 129.7, 129.5, 124.9, 124.6, 116.8, 115.1, 114.9, 114.3, 114.2, 111.0, 104.1, 103.2, 56.5, 55.0.

HRMS (ESI$^-$): $C_{23}H_{19}O_4^-$ [M-H]$^-$: requires 359.1289, found: 359.1290, Δ +0.28 ppm.

5. General Procedure for the Counterion Mediated Kinetic Resolution of BINOLs

General Procedure A:

The appropriate methyl BINOL (0.150 mmol, 1.00 equiv.), benzyl tosylate* (59.1 mg, 0.225 mmol, 1.50 equiv.) and *N*-(2,3,4-trifluorobenzyl)hydrocinchoninium bromide (7.8 mg, 0.015 mmol, 0.10 equiv.) were added to a 7 mL vial equipped with a magnetic stirrer bar (pictured below) under air. Benzene (5.40 mL) and diethyl ether (0.60 mL) were added followed by K_2CO_3 (sat. aq., 208 µL). The reaction was stirred at 1000-1400 rpm at room temperature (20-25°C) for the required reaction time**. Piperidine*** (0.30 mL) was added and stirring was continued for a further 30 minutes. Hydrochloric acid (3 M aq., 0.50 mL) was added dropwise and the phases were separated and the aqueous phase was extracted with EtOAc (2 × 5.00 mL). The combined organic layers were concentrated *in vacuo* and purified by flash column chromatography using the appropriate eluent to give the corresponding enantioenriched benzylated product and recovered starting material.

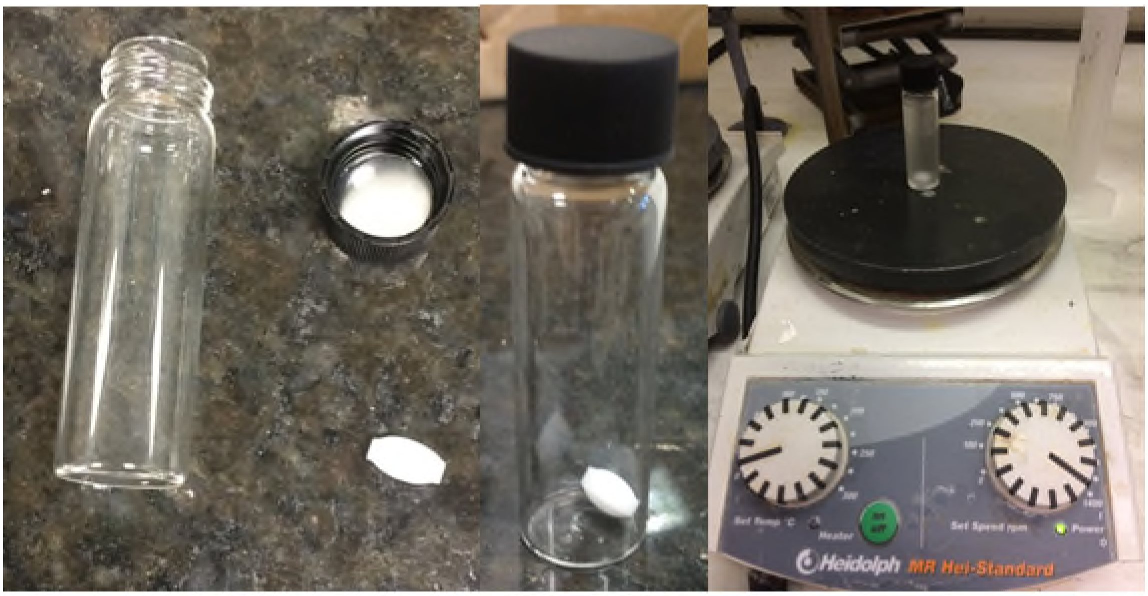

Reaction setup for general procedure A.

*Benzyl tosylate was prepared following a literature procedure[12] and stored at −20 °C in the dark.

**The reaction time was varied between different substrates to achieve the desired conversion. Typical reaction times were between 24 and 48 hours.

***Piperidine was added to consume excess benzyl tosylate. This is done to stop the reaction at the desired conversion and to prevent side product formation during purification.

Kinetic resolution equations[15]

Consider a reaction in which enantiomers R and S of the starting material react to give enantiomers R' and S' of the product, respectively.

After a certain period of time, assuming, without loss of generality that enantiomer S reacts faster than enantiomer R, we have the enantiomeric excess of the starting material given by

$$ee = \frac{[R]-[S]}{[R]+[S]}$$

Similarly, the enantiomeric excess of the product can be expressed as

$$ee' = \frac{[S']-[R']}{[S']+[R']}$$

Considering also that the conversion c is given by the formula

$$c = \frac{[S']+[R']}{[S]+[R]+[S']+[R']}$$

We can deduce a relationship between the c, ee and ee' in the form of Equation 1.

$$ee = ee'\frac{c}{1-c} \qquad (Equation\ 1)$$

Assuming that each enantiomer reacts according to first order kinetics and that there is no background reaction and no other side-reactions, the ratio between the reaction rate of enantiomer S (k_S) and that of enantiomer R (k_R) is called the S-factor (s). From these assumptions an equation can be derived for s as a function of c and ee:

$$s = \frac{\log[(1-c)(1-ee)]}{\log[(1-c)(1+ee)]} \qquad (Equation\ 2)$$

6. Counterion Mediated Kinetic Resolution of BINOLs

Kinetic resolution of 2'-methoxy-[1,1'-binaphthalen]-2-ol (1)

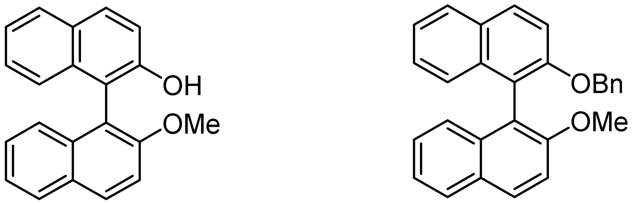

Following **General procedure A,** 2'-methoxy-[1,1'-binaphthalen]-2-ol (**1**) (45.0 mg, 0.15 mmol) was reacted for 24 h. Purification *via* flash column chromatography (30% CH_2Cl_2 – petroleum ether 40-60 to 80% CH_2Cl_2 – petroleum ether 40-60) afforded 2-(benzyloxy)-2'-methoxy-1,1'-binaphthalene (**Bn-1**) as an off-white solid (28.6 mg, 49%, 90:10 e.r.) and recovered starting material (**1**) (21.2 mg, 47%, 98:2 e.r.).

(S)-2-(Benzyloxy)-2'-methoxy-1,1'-binaphthalene (Bn-1):

m.p. = 130-132 °C (CH_2Cl_2/petroleum ether 40-60).

IR (film) ν_{max}/cm^{-1}: 3058, 3037, 2933, 1591, 1506, 1262, 1249, 1220, 1090, 1061, 806, 746, 734.

^{1}H NMR (400 MHz, $CDCl_3$) δ_H = 8.01 (d, *J* = 9.0 Hz, 1H), 7.92 (d, *J* = 9.4 Hz, 1H), 7.90–7.85 (m, 2H), 7.47 (d, *J* = 9.0 Hz, 1H), 7.44 (d, *J* = 9.0 Hz, 1H), 7.34 (m, 1.3 Hz, 2H), 7.25–7.20 (m, 3H), 7.20–7.12 (m, 4H), 7.02–6.95 (m, 2H), 5.35–4.85 (m, 2H), 3.77 (s, 3H).

^{13}C NMR (101 MHz, $CDCl_3$) δ_C = 155.1, 154.2, 137.7, 134.3, 134.2, 129.6, 129.6, 129.5, 129.4, 129.1, 128.3, 128.1, 128.0, 127.4, 126.9, 126.5, 126.4, 125.6, 125.5, 123.9, 123.6, 121.0, 119.6, 116.3, 114.0, 71.4, 56.8.

HRMS (ESI$^+$) $C_{28}H_{22}O_2$ $[M+Na]^+$ requires 413.15120; found 413.15147, Δ 0.6 ppm.

Chiral HPLC: (Chiralpak IC, 1% ^{i}PrOH, 99% hexane, 1.0 mL min^{-1}, λ = 289 nm) τ_R (major) = 8.0 min, τ_R (minor) = 11.8 min.

$[\alpha]_D^{25}$ = -58.7 (*c* = 1.00, $CHCl_3$).

Data in agreement with literature reported.[11]

(R)-2'-Methoxy-[1,1'-binaphthalen]-2-ol (1):

m.p. = 108-109 °C (CH_2Cl_2/petroleum ether b.p. 40-60 °C).

Chiral HPLC: (Chiralpak ADH, 7.5% ^{i}PrOH, 90% hexane, 1.0 mL min^{-1}, λ = 290 nm) τ_R (major) = 25.7 min, τ_R (minor) = 16.6 min.

$[\alpha]_D^{25}$ = +42.0(*c* = 0.50, acetone).

Kinetic resolution of 2'-isopropoxy-[1,1'-binaphthalen]-2-ol (9)

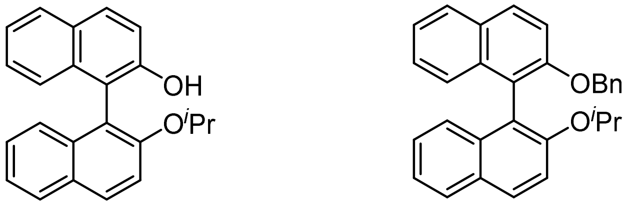

Following **General procedure A,** 2'-isopropoxy-[1,1'-binaphthalen]-2-ol (**9**) (49.3 mg, 0.150 mmol) was reacted for 27 h. Purification *via* flash column chromatography (3:1 petroleum ether 40-60 – CH_2Cl_2 to 1:1 CH_2Cl_2 – petroleum ether 40-60) afforded 2-(benzyloxy)-2'-isopropoxy-1,1'-binaphthalene (**Bn-9**) as an off-white solid (33.4 mg, 53%, 89.5:10.5 e.r.) and recovered starting material (**9**) (23.3 mg, 47%, 96.0:4.0 e.r.)

(S)-2-(benzyloxy)-2'-isopropoxy-1,1'-binaphthalene (Bn-9):

m.p. = 43-45 °C (CH_2Cl_2/petroleum ether 40-60).

IR (film) ν_{max}/cm^{-1}: 3059, 2975, 2928, 1622, 1592, 1506, 1454, 1327, 1262, 1241, 1223, 1147, 1132, 1112, 1088, 1054, 1018, 1001, 806, 748, 696

^{1}H NMR (500 MHz, $CDCl_3$) δ_H: 7.94 (d, *J* = 9.1 Hz, 1H), 7.92-7.86 (m, 2H), 7.85 (d, *J* = 8.2 Hz, 1H), 7.43 (d, *J* = 8.8 Hz, 1H), 7.39 (d, *J* = 8.8 Hz, 1H), 7.35-7.29 (m, 2H), 7.25-7.19 (m, 3H), 7.19-7.12 (m, 4H), 7.07-7.00 (m, 2H), 5.11-5.02 (m, 2H), 4.41 (sept, *J* = 6.1 Hz, 1H), 1.04 (d, *J* = 6.2 Hz, 1H), 0.95 (d, *J* = 6.0 Hz, 1H)

^{13}C NMR (126 MHz, $CDCl_3$) δ_C: 154.2, 153.9, 137.9, 134.5, 134.3, 129.6, 129.5, 129.2, 129.1, 128.2, 127.9, 127.9, 127.4, 126.9, 126.3, 126.2, 125.8, 125.7, 123.7, 123.7, 122.0, 121.3, 118.1, 116.2, 72.3, 71.3, 22.6, 22.4

HRMS (ESI^+) $C_{30}H_{27}O_2$ $[M+H]^+$ requires 419.20056; found 419.20059, Δ +0.1 ppm.

Chiral HPLC: (Chiralpak ADH, 5% ^{i}PrOH, 95% hexane, 1.00 mL min^{-1}, λ = 290 nm) τ_R (major) = 5.1 min, τ_R (minor) = 5.6 min

$[\alpha]_D^{25}$ = −80.0 (*c* = 1.00, $CHCl_3$).

(R)-2'-isopropoxy-[1,1'-binaphthalen]-2-ol (9):

m.p. = 54-56 °C (CH_2Cl_2/petroleum ether 40-60).

Chiral HPLC: (Chiralpak ADH, 10% ^{i}PrOH, 90% hexane, 1.00 mL min^{-1}, λ = 290 nm) τ_R (major) = 21.1 min, τ_R (minor) = 6.7 min

$[\alpha]_D^{25}$ = −68.9 (*c* = 1.00, $CHCl_3$).

Kinetic resolution of 2'-(benzyloxy)-[1,1'-binaphthalen]-2-ol (10)

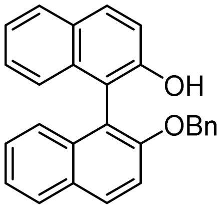

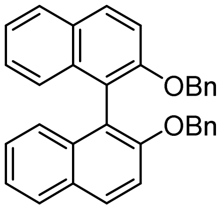

Following **General procedure A,** 2'-(benzyloxy)-[1,1'-binaphthalen]-2-ol (**10**) (56.5 mg, 0.150 mmol) was reacted for 27 h. Purification *via* flash column chromatography (2:1 petroleum ether 40-60 – CH_2Cl_2 to 1:2 CH_2Cl_2 – petroleum ether 40-60) afforded 2,2'-bis(benzyloxy)-1,1'-binaphthalene (**Bn-10**) as an off-white solid (29.2 mg, 42%, 90.5:9.5 e.r.) and recovered starting material (**9**) (25.3 mg, 45%, 94.5:5.5 e.r.)

(S)- 2,2'-bis(benzyloxy)-1,1'-binaphthalene (Bn-10):

m.p. = 97-99 °C (CH_2Cl_2/petroleum ether 40-60).

^{1}H NMR (500 MHz, $CDCl_3$) δ_H: 7.85 (d, *J* = 9.01 Hz, 2H), 7.80 (d, *J* = 8.12 Hz, 2H), 7.34 (d, *J* = 9.01 Hz, 2H), 7.28-7.23 (m, 2H), 7.18-7.12 (m, 4H), 7.08-6.98 (m, 6H), 6.89 (d, *J* = 6.94 Hz, 1H), 5.00-4.92 (m, 2H).

^{13}C NMR (126 MHz, $CDCl_3$) δ_C: 154.2, 137.7, 134.3, 129.6, 129.4, 128.2, 128.0, 127.4, 126.8, 126.4, 125.7, 123.8, 120.9, 116.1, 71.3

Chiral HPLC: (Chiralpak ADH, 10% ^{i}PrOH, 90% hexane, 1.00 mL min^{-1}, λ = 290 nm) τ_R (major) = 7.3 min, τ_R (minor) = 8.7 min

$[\alpha]_D^{25}$ = −34.6 (*c* = 1.00, $CHCl_3$).

Data in agreement with literature reported.[16]

(R)-2'-(benzyloxy)-[1,1'-binaphthalen]-2-ol (10):

m.p. = 108-110 °C (CH_2Cl_2/petroleum ether 40-60).

Chiral HPLC: (Chiralpak ADH, 20% ^{i}PrOH, 80% hexane, 1.00 mL min^{-1}, λ = 290 nm) τ_R (major) = 35.2 min, τ_R (minor) = 19.5 min

$[\alpha]_D^{25}$ = +1.5 (*c* = 1.00, $CHCl_3$).

Kinetic resolution of 2'-methoxy-6'-nitro-[1,1'-binaphthalen]-2-ol (11)

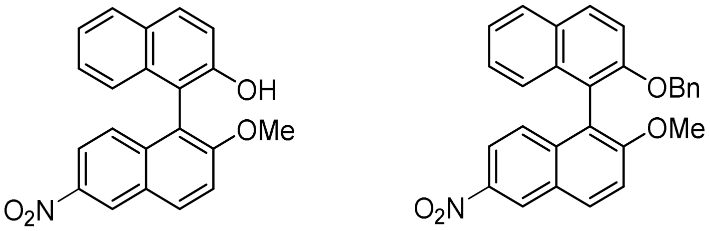

Following **General procedure A**, 2'-methoxy-6'-nitro-[1,1'-binaphthalen]-2-ol (**11**) (51.8 mg, 0.150 mmol) was reacted for 24 h. Purification *via* flash column chromatography (3:7 petroleum ether 40-60 – CH_2Cl_2 to 4:1 CH_2Cl_2 – petroleum ether 40-60) afforded 2'-(benzyloxy)-2-methoxy-6-nitro-1,1'-binaphthalene (**Bn-11**) as an off-white solid (35.0 mg, 54%, 90:10 e.r.) and recovered starting material (**11**) (20.1 mg, 39%, 99.5:0.5 e.r.).

(S)- 2'-(benzyloxy)-2-methoxy-6-nitro-1,1'-binaphthalene (Bn-11):

m.p. = 111-112 °C (CH_2Cl_2/petroleum ether 40-60).

IR (film) ν_{max}/cm^{-1}: 3062, 3031, 3010, 2960, 2938, 2841, 1619, 1599, 1594, 1529, 1496, 1462, 1328, 1247, 1220, 1098, 1055, 1046, 807, 740.

^{1}H NMR (400 MHz, $CDCl_3$) δ = 8.86 (d, *J* = 2.3 Hz, 1H), 8.18 (d, *J* = 8.7 Hz, 1H), 7.97 (d, *J* = 9.3 Hz, 1H), 7.94 (dd, *J* = 9.4, 2.4 Hz, 1H), 7.89 (d, *J* = 8.2 Hz, 1H), 7.59 (d, *J* = 9.1 Hz, 1H), 7.46 (d, *J* = 9.1 Hz, 1H), 7.36 (ddd, *J* = 8.1, 6.7, 1.2 Hz, 1H), 7.29–7.23 (m, 1H), 7.21 (d, *J* = 9.4 Hz, 1H), 7.16 (dd, *J* = 5.1, 2.0 Hz, 3H), 7.11–7.06 (m, 1H), 7.02–6.96 (m, 2H), 5.09 (d, *J* = 2.1 Hz, 2H), 3.81 (s, 3H).

^{13}C NMR (101 MHz, $CDCl_3$) δ = 158.3, 154.1, 143.8, 137.4, 137.1, 133.9, 132.0, 130.1, 129.5, 128.3, 128.3, 127.7, 127.2, 127.0, 126.8, 126.8, 125.2, 124.9, 124.1, 120.2, 119.8, 119.1, 115.8, 115.4, 71.2, 56.6.

HRMS (ESI^+) $C_{28}H_{21}O_4N$ $[M+Na]^+$ requires 458.13738; found 458.13644, Δ −2.0 ppm.

Chiral HPLC: (Chiralpak ADH, 10% ^{i}PrOH, 90% hexane, 1.0 mL min^{-1}, λ = 279 nm) τ_R (major) = 14.2 min, τ_R (minor) = 26.1 min.

$[\alpha]_D^{25}$ = +46.4 (*c* = 1.00, $CHCl_3$).

(R)- 2'-methoxy-6'-nitro-[1,1'-binaphthalen]-2-ol (11):

m.p. = 195-197 °C (CH_2Cl_2/petroleum ether 40-60).

Chiral HPLC: (Chiralpak ADH, 10% ^{i}PrOH, 90% hexane, 1.0 mL min^{-1}, λ = 277 nm) τ_R (major) = 27.4 min, τ_R (minor) = 29.8 min.

$[\alpha]_D^{25}$ = −114.5 (*c* = 1.00, $CHCl_3$).

Kinetic resolution of 2'-methoxy-6'-methyl-[1,1'-binaphthalen]-2-ol (12)

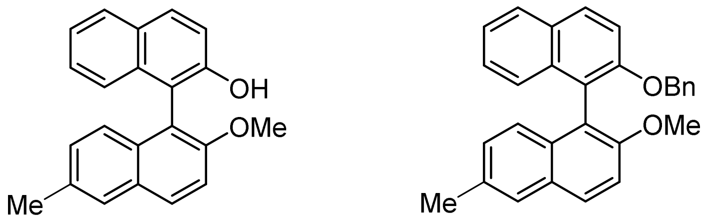

Following **General procedure A,** 2'-methoxy-6'-methyl-[1,1'-binaphthalen]-2-ol (**12**) (47.1 mg, 0.150 mmol) was reacted for 24 h. Purification *via* flash column chromatography (3:7 CH_2Cl_2 – petroleum ether 40-60 to 4:1 CH_2Cl_2 – petroleum ether 40-60) afforded 2'-(benzyloxy)-2-methoxy-6-methyl-1,1'-binaphthalene (**Bn-12**) as an off-white solid (32.6 mg, 54%, 80:20 e.r.) and recovered starting material (**12**) (18.6 mg, 39%, 99:1 e.r.).

(R)-2'-(Benzyloxy)-2-methoxy-6-methyl-1,1'-binaphthalene (Bn-12):

m.p. = 109-110 °C (CH_2Cl_2/petroleum ether 40-60).

IR (film) ν_{max}/cm^{-1}: 3060, 3031, 2934, 2920, 1594, 1504, 1272, 1263, 1252, 1221, 1093, 1060, 805, 734.

^{1}H NMR (400 MHz, $CDCl_3$) *δ* = 7.92 (dd, *J* = 9.0, 2.2 Hz, 2H), 7.87 (d, *J* = 8.2 Hz, 1H), 7.71–7.62 (m, 1H), 7.45 (d, *J* = 5.0 Hz, 1H), 7.43 (d, *J* = 5.0 Hz, 1H), 7.34 (ddd, *J* = 8.1, 6.4, 1.6 Hz, 1H), 7.28–7.21 (m, 2H), 7.19–7.17 (m, 3H), 7.07 (m, 2H), 7.05–7.00 (m, 2H), 5.09 (d, *J* = 12.6 Hz, 1H), 5.05 (d, *J* = 12.7 Hz, 1H), 3.75 (s, 3H), 2.48 (s, 2H).

^{13}C NMR (101 MHz, $CDCl_3$) *δ* = 154.9, 154.5, 138.1, 134.6, 133.3, 132.7, 129.9, 129.9, 129.6, 129.1, 128.6, 128.3, 127.7, 127.3, 127.2, 126.7, 125.9, 125.7, 124.2, 121.5, 119.8, 116.8, 114.5, 71.8, 57.2, 21.9.

HRMS (ESI^+) $C_{29}H_{24}O_2$ $[M+Na]^+$ requires 427.16685; found 427.16644, Δ 1.0 ppm.

Chiral HPLC: (Chiralpak IC, 1% ^{i}PrOH, 99% hexane, 1.0 mL min^{-1}, λ = 295 nm) τ_R (major) = 12.2 min, τ_R (minor) = 7.8 min.

$[\alpha]_D^{25}$ = −32.5 (*c* = 1.00, $CHCl_3$).

Data in agreement with literature reported.[11]

(S)- 2'-Methoxy-6'-methyl-[1,1'-binaphthalen]-2-ol (12):

m.p. = 137-138 °C (CH_2Cl_2/petroleum ether 40-60).

Chiral HPLC: (Chiralpak ADH, 10% ^{i}PrOH, 90% hexane, 1.0 mL min^{-1}, λ = 341 nm) τ_R (minor) = 11.5 min, τ_R (major) = 12.9 min.

$[\alpha]_D^{25}$ = −80.6 (*c* = 1.00, $CHCl_3$).

Kinetic resolution of 7-bromo-2'-methoxy-[1,1'-binaphthalen]-2-ol (13)

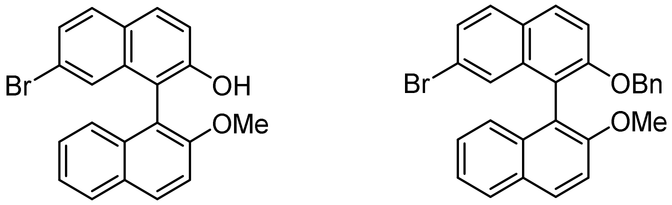

Following **General procedure A,** 7-bromo-2'-methoxy-[1,1'-binaphthalen]-2-ol (**13**) (56.9 mg, 0.150 mmol) was reacted for 36 h. Purification *via* flash column chromatography (3:7 CH_2Cl_2 – petroleum ether 40-60 to 4:1 CH_2Cl_2 – petroleum ether 40-60) afforded 2-(benzyloxy)-7-bromo-2'-methoxy-1,1'-binaphthalene (**Bn-13**) as an off-white solid (37.3 mg, 53%, 85:15 e.r.) and recovered starting material (**13**) (22.5 mg, 40%, 99.5:0.5 e.r.).

(S)- [2-(Benzyloxy)-7-bromo-2'-methoxy-1,1'-binaphthalene] (Bn-13):

m.p. = 64-65 °C (CH_2Cl_2/petroleum ether 40-60).

IR (film) ν_{max}/cm^{-1}: 3061, 3032, 2837, 1613, 1497, 1267, 1255.

^{1}H NMR (400 MHz, $CDCl_3$) δ = 7.92 (d, *J* = 9.1 Hz, 1H), 7.81 (d, *J* = 8.2 Hz, 1H), 7.77 (d, *J* = 9.3 Hz, 1H), 7.62 (d, *J* = 8.7 Hz, 1H), 7.37 (d, *J* = 9.0 Hz, 1H), 7.33–7.23 (m, 4H), 7.19–7.12 (m, 1H), 7.10–6.99 (m, 4H), 6.91–6.85 (m, 2H), 5.03–4.91 (m, 3H), 3.68 (s, 4H).

^{13}C NMR (101 MHz, $CDCl_3$) δ = 154.9, 154.7, 137.3, 135.4, 133.9, 129.8, 129.7, 129.2, 129.2, 128.2, 128.0, 127.8, 127.4, 127.4, 127.2, 126.7, 126.5, 125.1, 123.6, 121.0, 120.1, 118.4, 116.2 , 113.7, 71.0, 56.6.

HRMS (ESI^+) $C_{28}H_{21}O_2{}^{79}Br$ $[M+Na]^+$ requires 491.06171; found 491.06180, Δ +0.2 ppm.

Chiral HPLC: (Chiralpak ADH, 10% ^{i}PrOH, 98% hexane, 1.0 mL min^{-1}, λ = 295 nm) τ_R (major) = 7.5 min, τ_R (minor) = 8.7 min.

$[\alpha]_D^{25}$ = −23.2 (*c* = 1.00, $CHCl_3$).

Data in agreement with literature reported.[11]

(R)- 7-Bromo-2'-methoxy-[1,1'-binaphthalen]-2-ol (13):

m.p. = 178-179 °C (CH_2Cl_2/petroleum ether 40-60).

Chiral HPLC: (Chiralpak ADH, 10% ^{i}PrOH, 90% hexane, 1.0 mL min^{-1}, λ = 295 nm) τ_R (minor) = 11.6 min, τ_R (major) = 13.7 min.

$[\alpha]_D^{25}$ = −53.8 (*c* = 1.00, $CHCl_3$).

Kinetic resolution of 2',7-dimethoxy-[1,1'-binaphthalen]-2-ol (14)

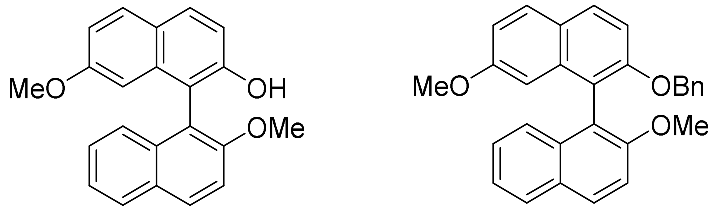

Following **General procedure A**, 2',7-dimethoxy-[1,1'-binaphthalen]-2-ol (**14**) (49.6 mg, 0.150 mmol) was reacted for 27 h. Purification *via* flash column chromatography (3:1 petroleum ether 40-60/CH_2Cl_2 to 1:3 petroleum ether 40-60/CH_2Cl_2) afforded 2-(benzyloxy)-2',7-dimethoxy-1,1'-binaphthalene (**Bn-14**) as an off-white solid (32.5 mg, 52%, 88.5:11.5 e.r.) and recovered starting material (**14**) (21.2 mg, 43%, 99.0:1.0 e.r.).

(S)-2-(benzyloxy)-2',7-dimethoxy-1,1'-binaphthalene (Bn-14)

m.p. = 51-53 °C (CH_2Cl_2/petroleum ether 40-60).

IR (film) ν_{max}/cm^{-1}: 3060, 2934, 2836, 1624, 1593, 1509, 1461, 1426, 1354, 1318, 1264, 1224, 1178, 1147, 1092, 1063, 1045, 1029, 828, 812, 749, 696

^{1}H NMR (500 MHz, $CDCl_3$) δ_H: 8.00 (d, *J* = 9.13 Hz, 1H), 7.90 (d, *J* = 8.07 Hz, 1H), 7.84 (d, *J* = 8.92 Hz, 1H), 7.77 (d, *J* = 8.92 Hz, 1H), 7.47 (d, *J* = 9.13 Hz, 1H), 7.34 (ddd, *J* = 8.07, 6.58, 1.43 Hz, 1H), 7.28 (d, *J* = 8.91 Hz, 1H), 7.26-7.19 (m, 2H), 7.19-7.12 (m, 3H), 7.02 (dd, *J* = 8.90, 2.45 Hz, 1H), 7.00-6.93 (m, 2H), 6.49 (d, *J* = 2.39 Hz, 1H), 5.10-5.00 (m, 2H), 3.78 (s, 3H), 3.51 (s, 3H)

^{13}C NMR (126 MHz, $CDCl_3$) δ_C: 158.2, 155.1, 154.8, 137.8, 135.6, 13.1, 129.6, 129.5, 129.4, 129.1, 128.2, 128.0, 127.4, 126.8, 126.4, 125.6, 125.1, 123.6, 119.8, 119.7, 116.3, 114.0, 113.6, 104.2, 71.2, 56.8, 55.1.

HRMS (ESI^+) $C_{29}H_{25}O_3^+$ $[M+H]^+$ requires 421.17982; found: 421.17953, Δ -0.68 ppm.

Chiral HPLC: (Chiralpak ODH, 5% ^{i}PrOH, 95% hexane, 1.00 mL min^{-1}, λ = 290 nm) τ_R (major) = 8.8 min, τ_R (minor) = 9.9 min.

$[\alpha]_D^{25}$ = −5.2 (*c* = 1.00, $CHCl_3$).

(R)-2',7-dimethoxy-[1,1'-binaphthalen]-2-ol (14)

m.p. = 103-105 °C (CH_2Cl_2/petroleum ether 40-60).

Chiral HPLC: (Chiralpak ADH, 15% ^{i}PrOH, 85% hexane, 1.00 mL min^{-1}, λ = 290 nm) τ_R (major) = 17.9 min, τ_R (minor) = 13.5 min.

$[\alpha]_D^{25}$ = −105.3 (*c* = 1.00, $CHCl_3$).

Kinetic resolution of 2'-methoxy-7-methyl-[1,1'-binaphthalen]-2-ol] (15)

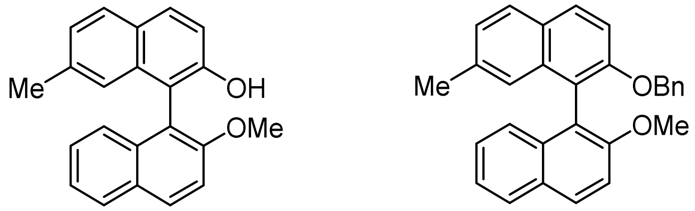

Following **General procedure A,** 2'-methoxy-7-methyl-[1,1'-binaphthalen]-2-ol (**15**) (47.2 mg, 0.150 mmol) was reacted for 24 h. Purification *via* flash column chromatography (3:7 CH_2Cl_2 – petroleum ether 40-60 to 4:1 CH_2Cl_2 – petroleum ether 40-60) afforded 2-(benzyloxy)-2'-methoxy-7-methyl-1,1'-binaphthalene (bn-**15**) as an off-white solid (34.0 mg, 56%, 86:14 e.r.) and recovered starting material (**15**) (18.6 mg, 39%, 99.5:0.5 e.r.).

(S)- 2-(benzyloxy)-2'-methoxy-7-methyl-1,1'-binaphthalene (Bn-15):

m.p. = 69-70 °C (CH_2Cl_2/petroleum ether 40-60).

IR (film) ν_{max}/cm^{-1}: 3054, 2836, 1624, 1593, 1506, 1470, 1452, 1263, 826, 810.

^{1}H NMR (400 MHz, $CDCl_3$) δ = 8.02 (d, *J* = 9.0 Hz, 1H), 7.91 (d, *J* = 8.2 Hz, 1H), 7.88 (d, *J* = 9.0 Hz, 1H), 7.77 (d, *J* = 8.3 Hz, 1H), 7.48 (d, *J* = 9.0 Hz, 1H), 7.41–7.31 (m, 2H), 7.25–7.13 (m, 6H), 6.99–6.95 (m, 3H), 3.77 (s, 2H), 2.27 (s, 3H).

^{13}C NMR (101 MHz, $CDCl_3$) δ = 155.0, 154.1, 137.7, 136.0, 134.3, 134.2, 129.3, 129.2, 129.0, 128.1, 127.9, 127.8, 127.8, 127.3, 126.7, 126.2, 125.5, 124.3, 124.2, 123.5, 120.2, 119.7, 115.2, 114.0, 71.2, 56.7, 22.0.

HRMS (ESI^+) $C_{29}H_{24}O_2$ $[M+H]^+$ requires 405.18491; found 405.18488, Δ −0.1 ppm.

Chiral HPLC: (Chiralpak OJH, 3% EtOH, 97% hexane, 1.0 mL min^{-1}, λ = 300 nm) τ_R (minor) = 9.4 min, τ_R (major) = 15.1 min.

$[\alpha]_D^{25}$ = −23.8 (*c* = 1.00, $CHCl_3$).

Data in agreement with literature reported.[11]

(R)- 2'-methoxy-7-methyl-[1,1'-binaphthalen]-2-ol (15):

m.p. = 124-125 °C (CH_2Cl_2/petroleum ether 40-60).

Chiral HPLC: (Chiralpak ADH, 10% ^{i}PrOH, 90% hexane, 1.0 mL min^{-1}, λ = 290 nm) τ_R (minor) = 12.8 min, τ_R (major) = 15.6 min.

$[\alpha]_D^{25}$ = −59.0 (*c* = 1.00, $CHCl_3$).

Kinetic resolution of 2'-methoxy-7-phenyl-[1,1'-binaphthalen]-2-ol (16)

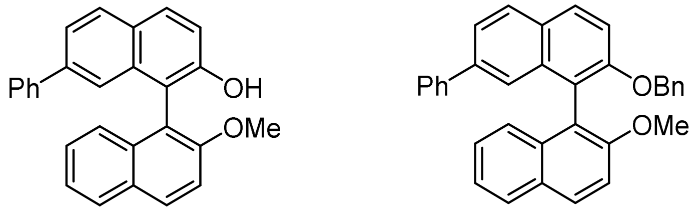

Following **General procedure A,** 2'-methoxy-7-phenyl-[1,1'-binaphthalen]-2-ol (**16**) (56.4 mg, 0.150 mmol) was reacted for 24 h. Purification *via* flash column chromatography (3:7 CH_2Cl_2 – petroleum ether 40-60 to 4:1 CH_2Cl_2 – petroleum ether 40-60) afforded 2-(benzyloxy)-2'-methoxy-7-phenyl-1,1'-binaphthalene (**Bn-16**) as an off-white solid (35.1 mg, 50%, 90:10 e.r.) and recovered starting material (**16**) (23.6 mg, 42%, 97:3 e.r.).

(S)-2-(benzyloxy)-2'-methoxy-7-phenyl-1,1'-binaphthalene (Bn-16):

m.p. = 68-89 °C (CH_2Cl_2/petroleum ether 40-60).

IR (film) ν_{max}/cm^{-1}: 3058, 3032, 2935, 2837, 1621, 1594, 1509, 1493, 1454, 1265, 1250, 1221, 1093, 1063, 908, 809, 747, 733, 697.

^{1}H NMR (400 MHz, $CDCl_3$) δ = 7.90 (d, *J* = 9.1 Hz, 1H), 7.83 (d, *J* = 8.7 Hz, 2H), 7.79 (d, *J* = 8.2 Hz, 1H), 7.50 (dd, *J* = 8.5, 1.8 Hz, 1H), 7.36 (d, *J* = 9.0 Hz, 1H), 7.33 (d, *J* = 9.0 Hz, 1H), 7.30–7.26 (m, 3H), 7.21 (m, 3H), 7.17–7.10 (m, 4H), 7.08–7.04 (m, 3H), 6.92–6.84 (m, 2H), 5.02–4.91 (m, 2H), 3.67 (s, 3H).

^{13}C NMR (101 MHz, $CDCl_3$) δ = 155.0, 154.5, 141.5, 139.0, 137.6, 134.4, 134.2, 129.6, 129.2, 129.0, 128.7, 128.6, 128.5, 128.2, 127.9, 127.5, 127.3, 127.1, 126.8, 126.4, 125.4, 123.7, 123.5, 123.5, 121.2, 119.3, 116.3, 113.8, 71.3, 56.7.

HRMS (ESI^+) $C_{34}H_{26}O_2$ $[M+Na]^+$ requires 489.18250; found 489.18233, Δ -0.3 ppm.

Chiral HPLC: (Chiralpak IC, 2% ^{i}PrOH, 98% hexane, 1.0 mL min^{-1}, λ = 289 nm) τ_R (major) = 20.5 min, τ_R (minor) = 23.5 min.

$[\alpha]_D^{25}$ = +28.3 (*c* = 1.00, $CHCl_3$).

Data in agreement with literature reported.[11]

(R)-2'-methoxy-7-phenyl-[1,1'-binaphthalen]-2-ol (16):

m.p. = 190-191 °C (CH_2Cl_2/petroleum ether 40-60).

Chiral HPLC: (Chiralpak ADH, 10% ^{i}PrOH, 90% hexane, 1.0 mL min^{-1}, λ = 272 nm) τ_R (major) = 11.6 min, τ_R (minor) = 14.1 min.

$[\alpha]_D^{25}$ = −118.1 (*c* = 1.00, $CHCl_3$).

Kinetic resolution of 7-fluoro-2'-methoxy-[1,1'-binaphthalen]-2-ol (17)

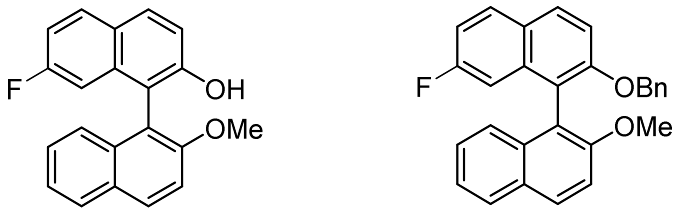

Following **General procedure A,** 7-fluoro-2'-methoxy-[1,1'-binaphthalen]-2-ol (**17**) (47.7 mg, 0.150 mmol) was reacted for 36 h. Purification *via* flash column chromatography (3:7 CH_2Cl_2 – petroleum ether 40-60 to 4:1 CH_2Cl_2 – petroleum ether 40-60) afforded 2-(benzyloxy)-7-fluoro-2'-methoxy-1,1'-binaphthalene (**Bn-17**) as an off-white solid (33.7 mg, 50%, 88:12 e.r.) and recovered starting material (**17**) (18.9 mg, 40%, 99:1 e.r.).

(S)- 2-(Benzyloxy)-7-fluoro-2'-methoxy-1,1'-binaphthalene (Bn-17):

m.p. = 88-90 °C (CH_2Cl_2/petroleum ether 40-60).

IR (film) ν_{max}/cm^{-1}: 3061, 2935, 2838, 1629, 1510, 1268, 1255, 1212, 811.

^{1}H NMR (500 MHz, $CDCl_3$) δ = 8.01 (d, *J* = 8.8 Hz, 1H), 7.93–7.87 (m, 2H), 7.83 (dd, *J* = 8.9, 5.8 Hz, 1H), 7.46 (d, *J* = 9.1 Hz, 1H), 7.37 (d, *J* = 9.2 Hz, 1H), 7.35–7.32 (m, 1H), 7.26–7.21 (m, 1H), 7.19–7.07 (m, 6H), 7.02–6.96 (m, 2H), 6.78 (dd, *J* = 11.4, 2.6 Hz, 1H), 5.09 (d, *J* = 12.7 Hz, 1H), 5.06 (d, *J* = 12.7 Hz, 1H), 3.78 (s, 4H).

^{13}C NMR (126 MHz, $CDCl_3$) δ = 161.5 (d, *J* = 245.1 Hz), 155.0, 155.0, 137.5, 135.5 (d, *J* = 9.3 Hz), 134.0, 130.5, 130.4, 129.8, 129.3 (d, *J* = 1.5 Hz), 128.3, 128.1, 127.5, 126.8, 126.6, 126.5, 125.2, 123.7, 120.4 (d, *J* = 5.6 Hz), 118.9, 115.2 (d, *J* = 2.6 Hz), 114.3, 114.1, 113.9, 108.9, 108.7, 71.1, 56.7.

^{19}F NMR: (471 MHz, $CDCl_3$) δ = -114.0

HRMS (ESI^+) $C_{28}H_{21}O_2F$ $[M+Na]^+$ requires 431.14178; found 431.14145, Δ −0.8 ppm.

Chiral HPLC: (Chiralpak IC, 1% ^{i}PrOH, 99% hexane, 1.0 mL min^{-1}, λ = 294 nm) τ_R (major) = 7.0 min, τ_R (minor) = 9.3 min.

$[\alpha]_D^{25}$ = −58.0 (*c* = 1.00, $CHCl_3$).

Data in agreement with literature reported.[11]

(R)- 7-Fluoro-2'-methoxy-[1,1'-binaphthalen]-2-ol (17):

m.p. = 118-119 °C (CH_2Cl_2/petroleum ether 40-60).

Chiral HPLC: (Chiralpak ADH, 10% ^{i}PrOH, 90% hexane, 1.0 mL min^{-1}) τ_R (major) = 11.6 min, τ_R (minor) = 14.1 min.

$[\alpha]_D^{25}$ = −13.5 (*c* = 1.00, $CHCl_3$).

Kinetic resolution of 2',6-dimethoxy-[1,1'-binaphthalen]-2-ol

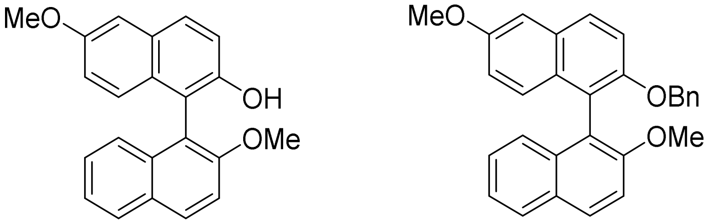

Following **General procedure A,** 2',6-dimethoxy-[1,1'-binaphthalen]-2-ol (**18**) (49.6 mg, 0.150 mmol) was reacted for 27 h. Purification *via* flash column chromatography (1:1 petroleum ether 40-60 – CH_2Cl_2 to 1:3 petroleum ether 40-60 – CH_2Cl_2) afforded 2-(benzyloxy)-2',6-dimethoxy-1,1'-binaphthalene (**Bn-18**) as an off-white solid (35.8 mg, 57%, 87.5:12.5 e.r.) and recovered starting material (**18**) (20.8 mg, 42%, 99.5:0.5 e.r.).

(S)-2-(benzyloxy)-2',6-dimethoxy-1,1'-binaphthalene (Bn-18)

m.p. = 108-100 °C (CH_2Cl_2/petroleum ether 40-60).

IR (film) ν_{max}/cm^{-1}: 3061, 3004, 2935, 2836, 1624, 1594, 1506, 1462, 1433, 1376, 1351, 1332, 1267, 1251, 1239, 1222, 1168, 1148, 1130, 1090, 1061, 1024, 849, 808, 748, 696

^{1}H NMR (500 MHz, $CDCl_3$) δ_H: 8.00 (d, *J* = 9.05 Hz, 1H), 7.89 (d, *J* = 8.10 Hz, 1H), 7.82 (d, *J* = 8.95 Hz, 1H), 7.46 (d, *J* = 9.05 Hz, 1H), 7.40 (d, *J* = 9.05 Hz, 1H), 7.37-7.31 (m, 1H), 7.26-7.21 (m, 1H), 7.21-7.13 (m, 4H), 7.11 (d, *J* = 9.12 Hz, 1H), 7.01-6.92 (m, 2H), 6.94 (dd, *J* = 9.22, 2.56 Hz, 1H), 5.06-4.96 (m, 2H), 3.91 (s, 3H), 3.77 (s, 3H).

^{13}C NMR (126 MHz, $CDCl_3$) δ_C: 156.4, 155.1, 152.8, 137.9, 134.2, 130.6, 129.6, 129.5, 129.3, 128.2, 128.0, 127.9, 127.4, 127.2, 127.0, 126.5, 125.5, 123.6, 121.5, 119.6, 119.2, 117.3, 114.0, 106.1, 71.8, 56.8, 55.4.

HRMS (ESI$^+$) $C_{29}H_{25}O_3$ $[M+H]^+$ requires 421.17982; found 421.17975, Δ −0.2 ppm.

Chiral HPLC: (Chiralpak IC, 1% ^{i}PrOH, 99% hexane, 1.00 mL min^{-1}, λ = 290 nm) τ_R (major) = 15.3 min, τ_R (minor) = 30.6 min

$[\alpha]_D^{25}$ = −34.9 (*c* = 1.00, $CHCl_3$).

(R)-2',6-dimethoxy-[1,1'-binaphthalen]-2-ol (18)

m.p. = 160-162 °C (CH_2Cl_2/petroleum ether 40-60).

Chiral HPLC: (Chiralpak ADH, 15% ^{i}PrOH, 85% hexane, 1.00 mL min^{-1}, λ = 290 nm) τ_R (major) = 27.0 min, τ_R (minor) = 13.9 min

$[\alpha]_D^{25}$ = −38.6 (*c* = 1.00, $CHCl_3$).

Kinetic resolution of 2'-methoxy-6-morpholino-[1,1'-binaphthalen]-2-ol (19)

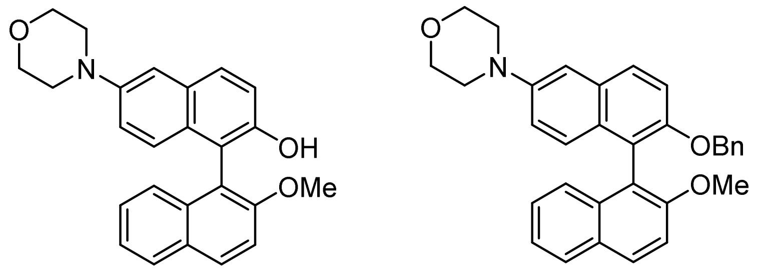

Following **General procedure A,** 2'-methoxy-6-morpholino-[1,1'-binaphthalen]-2-ol (**27**) (57.8 mg, 0.150 mmol) was reacted for 24 h. Purification *via* flash column chromatography (4:1 petroleum ether 40-60 – EtOAc to 7:3 petroleum ether 40-60 – EtOAc) afforded 4-(2-(benzyloxy)-2'-methoxy-[1,1'-binaphthalen]-6-yl)morpholine (**Bn-19**) as an off-white solid (33.3 mg, 47%, 87:13 e.r.) and recovered starting material (**19**) (19.6 mg, 34%, 99:1 e.r.).

(S)- 4-(2-(benzyloxy)-2'-methoxy-[1,1'-binaphthalen]-6-yl)morpholine (Bn-19):

m.p. = 144-145 °C (EtOAc/petroleum ether 40-60).

IR (film) ν_{max}/cm^{-1}: 3059, 3031, 2954, 2928, 2853, 2837, 1592, 1506, 1266, 1250, 1227, 1120, 1092.

^{1}H NMR (400 MHz, $CDCl_3$) δ = 7.99 (d, *J* = 9.0 Hz, 1H), 7.89 (d, *J* = 8.2 Hz, 1H), 7.78 (d, *J* = 9.0 Hz, 1H), 7.45 (d, *J* = 9.0 Hz, 1H), 7.37 (d, *J* = 9.0 Hz, 1H), 7.33 (app. t, *J* = 8.0 Hz, 1H), 7.22 (m, 1H), 7.16 (m, 5H), 7.10 (d, *J* = 9.4 Hz, 1H), 7.02 (dd, *J* = 9.3, 2.5 Hz, 1H), 6.99–6.91 (m, 2H), 5.06–4.92 (m, 2H), 3.92–3.85 (m, 4H), 3.76 (s, 3H), 3.24–3.16 (m, 4H).

^{13}C NMR (101 MHz, $CDCl_3$) δ = 155.1, 152.8, 147.8, 137.9, 134.3, 130.6, 129.5, 129.4, 129.3, 128.2, 128.1, 128.0, 127.4, 127.0, 126.7, 126.4, 125.6, 123.6, 121.3, 119.8, 119.7, 117.3, 114.0, 110.6, 71.8, 67.1, 56.8, 50.1.

HRMS (ESI$^+$) $C_{31}H_{29}O_3N$ $[M+H]^+$ requires 476.22202; found 476.22162, Δ −0.8 ppm.

Chiral HPLC: (Chiralpak IC, 3% ^{i}PrOH, 97% hexane, 1.0 mL min^{-1}, λ = 289 nm) τ_R (major) = 23.3 min, τ_R (minor) = 30.4 min.

$[\alpha]_D^{25}$ = −4.2 (*c* = 1.00, $CHCl_3$).

(R)- 2'-methoxy-6-morpholino-[1,1'-binaphthalen]-2-ol (19):

m.p. = 124-125 °C (EtOAc/petroleum ether 40-60).

Chiral HPLC: (Chiralpak ADH, 15% ^{i}PrOH, 85% hexane, 1.0 mL min^{-1}, λ = 272 nm) τ_R (minor) = 20.4 min, τ_R (major) = 30.4 min.

$[\alpha]_D^{25}$ = −31.2 (*c* = 1.00, $CHCl_3$).

Kinetic resolution of [2'-methoxy-6-phenyl-[1,1'-binaphthalen]-2-ol] (20)

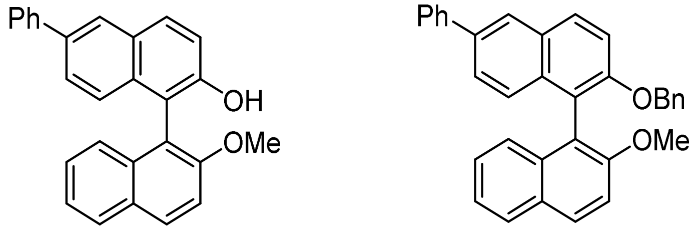

Following **General procedure A,** 2'-methoxy-6-phenyl-[1,1'-binaphthalen]-2-ol (**20**) (56.4 mg, 0.150 mmol) was reacted for 24 h. Purification *via* flash column chromatography (3:7 CH_2Cl_2 – petroleum ether 40-60 to 4:1 CH_2Cl_2 – petroleum ether 40-60) afforded 2-(benzyloxy)-2'-methoxy-6-phenyl-1,1'-binaphthalene (**Bn-20**) as an off-white solid (39.1 mg, 56%, 86:14 e.r.) and recovered starting material (**20**) (22.0 mg, 39%, 99:1 e.r.).

(S)- 2-(benzyloxy)-2'-methoxy-6-phenyl-1,1'-binaphthalene (Bn-20):

m.p. = 78-79°C (CH_2Cl_2/petroleum ether 40-60).

IR (film) ν_{max}/cm^{-1}: 3060, 3030, 2933, 2856, 2837, 1743, 1592, 1507, 1492, 1454, 1349, 1268, 1222, 1093, 1061, 1027, 1020, 806, 757, 750, 736, 696.

^{1}H NMR (400 MHz, $CDCl_3$) δ = 8.10 (d, *J* = 1.9 Hz, 1H), 8.05 (d, *J* = 9.1 Hz, 1H), 8.01 (d, *J* = 9.0 Hz, 1H), 7.94 (d, *J* = 8.1 Hz, 1H), 7.76–7.66 (m, 2H), 7.59 – 7.45 (m, 5H), 7.38 (ddd, *J* = 8.2, 6.5, 1.5 Hz, 2H), 7.32–7.25 (m, 2H), 7.25–7.16 (m, 4H), 7.08–6.99 (m, 2H), 5.18–5.03 (m, 2H), 3.82 (s, 3H).

^{13}C NMR (126 MHz, $CDCl_3$) δ = 155.0, 154.2, 141.2, 137.6, 136.5, 134.7, 134.1, 133.4, 129.7, 129.6, 129.5, 129.2, 128.8, 128.2, 128.0, 127.4, 127.3, 127.1, 126.8, 126.4, 126.1, 126.0, 125.9, 125.4, 123.5, 120.7, 119.3, 116.6, 113.9, 71.3, 56.7.

Chiral HPLC: (Chiralpak IC, 1% ^{i}PrOH, 99% hexane, 1.0 mL min^{-1}, λ = 259 nm) τ_R (major) = 10.2 min, τ_R (minor) = 19.7 min.

$[\alpha]_D^{25}$ = −77.0 (*c* = 1.00, $CHCl_3$).

Data in agreement with literature reported.[11]

(R)- 2'-methoxy-6-phenyl-[1,1'-binaphthalen]-2-ol (20):

m.p. = 58-59 °C (CH_2Cl_2/petroleum ether 40-60).

Chiral HPLC: (Chiralpak ADH, 10% ^{i}PrOH, 90% hexane, 1.0 mL min^{-1}, λ = 259 nm) τ_R (minor) = 13.2 min, τ_R (major) = 21.2 min.

$[\alpha]_D^{25}$ = −5.7 (*c* = 1.00, $CHCl_3$).

Kinetic resolution of 6-bromo-2'-methoxy-[1,1'-binaphthalen]-2-ol (21)

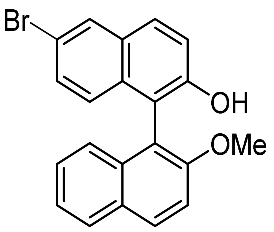

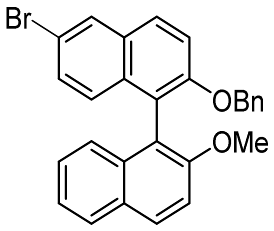

Following **General procedure A,** 6-bromo-2'-methoxy-[1,1'-binaphthalen]-2-ol (**21**) (56.9 mg, 0.150 mmol) was reacted for 24 h. Purification *via* flash column chromatography (3:7 CH_2Cl_2 – petroleum ether 40-60 to 4:1 CH_2Cl_2 – petroleum ether 40-60) afforded 2-(benzyloxy)-6-bromo-2'-methoxy-1,1'-binaphthalene (**Bn-21**) as an off-white solid (34.3 mg, 49%, 87:13 e.r.) and recovered starting material (**21**) (22.0 mg, 39%, 99:1 e.r.).

(S)- 2-(Benzyloxy)-6-bromo-2'-methoxy-1,1'-binaphthalene (Bn-21):

m.p. = 115-115 °C (CH_2Cl_2/petroleum ether 40-60).

IR (film) ν_{max}/cm^{-1}: 3061, 3033, 2837, 1584, 1494, 1268, 1065, 734.

^{1}H NMR (400 MHz, $CDCl_3$) *δ* = 7.93–7.88 (m, 2H), 7.79 (d, *J* = 8.2 Hz, 1H), 7.71 (d, *J* = 9.0 Hz, 1H), 7.34 (app. t, *J* = 9.4 Hz, 2H), 7.26–7.22 (m, 1H), 7.21–7.11 (m, 3H), 7.11–7.05 (m, 3H), 7.01 (d, *J* = 8.6 Hz, 1H), 6.96 (d, *J* = 9.0 Hz, 1H), 6.91–6.86 (m, 2H), 5.15–4.79 (m, 2H), 3.66 (s, 3H).

^{13}C NMR (101 MHz, $CDCl_3$) *δ* = 155.1, 154.4, 137.5, 134.1, 132.8, 130.6, 129.9, 129.8, 129.7, 129.3, 128.4, 128.3, 128.1, 127.5, 127.5, 126.8, 126.6, 125.3, 123.7, 121.2, 118.8, 117.7, 117.2, 113.8, 71.3, 56.7.

HRMS (ESI$^+$) $C_{28}H_{21}O_2{}^{79}Br$ $[M+Na]^+$ requires 491.06171; found 491.06180, Δ +0.2 ppm.

Chiral HPLC: (Chiralpak IC, 1% ^{i}PrOH, 99% hexane, 1.0 mL min^{-1}, λ = 295nm) τ_R (major) = 6.2 min, τ_R (minor) = 9.7 min.

$[\alpha]_D^{25}$ = −25.8 (*c* = 1.00, $CHCl_3$).

(R)- 6-Bromo-2'-methoxy-[1,1'-binaphthalen]-2-ol (21):

m.p. = 71-72 °C (CH_2Cl_2/petroleum ether 40-60).

Chiral HPLC: (Chiralpak ADH, 1% ^{i}PrOH, 99% hexane, 1.0 mL min^{-1}, λ = 281 nm) τ_R (major) = 19.1 min, τ_R (minor) = 14.3 min.

$[\alpha]_D^{25}$ = −49.6 (*c* = 1.00, $CHCl_3$).

Kinetic resolution of 2'-methoxy-5-methyl-[1,1'-binaphthalen]-2-ol (22)

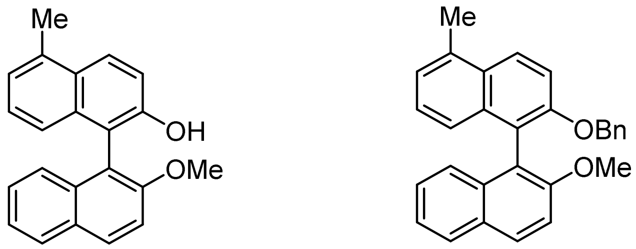

Following **General procedure A,** 2'-methoxy-5-methyl-[1,1'-binaphthalen]-2-ol (**21**) (47.2 mg, 0.150 mmol) was reacted for 24 h. Purification *via* flash column chromatography (3:7 CH_2Cl_2 – petroleum ether 40-60 to 4:1 CH_2Cl_2 – petroleum ether 40-60) afforded 2-(benzyloxy)-2'-methoxy-5-methyl-1,1'-binaphthalene (**Bn-21**) as an off-white solid (33.8 mg, 56%, 89:11 e.r.) and recovered starting material (**21**) (18.9 mg, 40%, 99.5:0.5 e.r.).

(S)-2-(benzyloxy)-2'-methoxy-5-methyl-1,1'-binaphthalene (Bn-21):

m.p. = 84-85 °C (CH_2Cl_2/petroleum ether 40-60).

IR (film) ν_{max}/cm^{-1}: 3020, 3937, 1801, 1742, 1521, 1494, 1347, 1265, 1237, 1217, 1175, 1053, 749, 691, 666.

^{1}H NMR (400 MHz, $CDCl_3$) δ = 8.10 (d, *J* = 9.2 Hz, 1H), 8.01 (d, *J* = 9.0 Hz, 1H), 7.90 (d, *J* = 8.3 Hz, 1H), 7.51–7.42 (m, 2H), 7.33 (t, *J* = 7.4 Hz, 1H), 7.24–7.04 (m, 8H), 7.03–6.97 (m, 2H), 5.15–5.02 (m, 2H), 3.77 (s, 3H), 2.75 (s, 3H).

^{13}C NMR (101 MHz, $CDCl_3$) δ = 155.1, 153.9, 137.8, 134.4, 134.4, 134.3, 129.5, 129.3, 128.7, 128.3, 128.0, 127.4, 126.9, 126.4, 126.3, 125.6, 124.8, 124.1, 123.6, 121.4, 119.9, 115.8, 114.0, 71.2, 56.8, 19.8.

HRMS (ESI$^+$) $C_{29}H_{24}O_2$ $[M+H]^+$ requires 405.18491; found 405.18494, Δ +0.1 ppm.

Chiral HPLC: (Chiralpak IC, 1% ^{i}PrOH, 99% hexane, 1.0 mL min^{-1}, λ = 295 nm) τ_R (major) = 6.9 min, τ_R (minor) = 13.6 min.

$[\alpha]_D^{25}$ = −41.4 (*c* = 1.00, $CHCl_3$).

Data in agreement with literature reported.[11]

(R)-2'-methoxy-5-methyl-[1,1'-binaphthalen]-2-ol (21):

m.p. = 74-75 °C (CH_2Cl_2/petroleum ether 40-60).

Chiral HPLC: (Chiralpak ADH, 10% ^{i}PrOH, 90% hexane, 1.0 mL min^{-1}, λ = 295 nm,) τ_R (major) = 20.0 min, τ_R (minor) = 12.8 min.

$[\alpha]_D^{25}$ = −13.3 (*c* = 1.00, $CHCl_3$).

Kinetic resolution of 2',5-dimethoxy-[1,1'-binaphthalen]-2-ol (23)

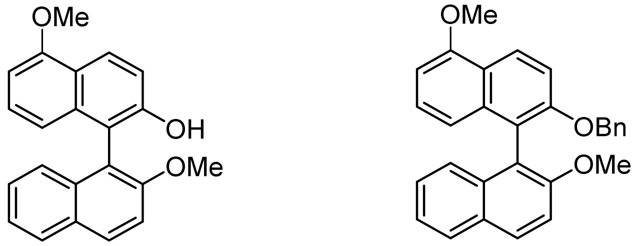

Following **General procedure A,** 2',5-dimethoxy-[1,1'-binaphthalen]-2-ol (**23**) (49.6 mg, 0.150 mmol) was reacted for 30 h. Purification *via* flash column chromatography (3:7 CH_2Cl_2 – petroleum ether 40-60 to 4:1 CH_2Cl_2 – petroleum ether 40-60) afforded 2-(benzyloxy)-2',5-dimethoxy-1,1'-binaphthalene (**Bn-23**) as an off-white solid (37.6 mg, 59%, 77:23 e.r.) and recovered starting material (**23**) (16.4 mg, 33%, 99.5:0.5 e.r.).

(S)- 2-(Benzyloxy)-2',5-dimethoxy-1,1'-binaphthalene (Bn-23):

m.p. = 151-152 °C (CH_2Cl_2/petroleum ether 40-60).

IR (film) ν_{max}/cm^{-1}:

^{1}H NMR (400 MHz, $CDCl_3$) δ = 8.38 (d, *J* = 8.4 Hz, 1H), 7.99 (d, *J* = 9.0 Hz, 1H), 7.89 (d, *J* = 8.2 Hz, 1H), 7.46 (d, *J* = 9.0 Hz, 1H), 7.41 (d, *J* = 9.3 Hz, 1H), 7.33 (ddd, *J* = 8.1, 6.6, 1.3 Hz, 1H), 7.23 (m, 1.4 Hz, 1H), 7.16 (m, 5H), 7.00 (m, 2H), 6.77 (d, *J* = 8.6 Hz, 1H), 6.68 (d, *J* = 6.8 Hz, 1H), 5.15–4.95 (m, 2H), 4.02 (s, 3H), 3.76 (s, 3H).

^{13}C NMR (101 MHz, $CDCl_3$) δ = 155.8, 155.1, 154.7, 137.7, 135.5, 134.2, 129.4, 129.3, 128.2, 128.0, 127.4, 126.9, 126.6, 126.4, 125.6, 123.6, 121.6, 120.5, 120.0, 118.0, 115.1, 114.1, 102.0, 71.1, 56.8, 55.6.

HRMS (ESI^+) $C_{29}H_{24}O_3$ $[M+H]^+$ requires 421.17982; found 421.17923, Δ −1.4 ppm.

Chiral HPLC: (Chiralpak ADH, 10% ^{i}PrOH, 90% hexane, 1.0 mL min^{-1}, λ = 293 nm) τ_R (minor) = 17.2 min, τ_R (major) = 27.9 min.

$[\alpha]_D^{25}$ = −37.3 (*c* = 1.00, $CHCl_3$).

(R)- 2',5-Dimethoxy-[1,1'-binaphthalen]-2-ol (23):

m.p. = 165-167 °C (CH_2Cl_2/petroleum ether 40-60).

Chiral HPLC: (Chiralpak ADH, 10% ^{i}PrOH, 90% hexane, 1.0 mL min^{-1}, λ = 293 nm) τ_R (major) = 17.3 min, τ_R (minor) = 28.0 min.

$[\alpha]_D^{25}$ = −35.7 (*c* = 1.00, $CHCl_3$).

Kinetic resolution of 5-bromo-2'-methoxy-[1,1'-binaphthalen]-2-ol (24)

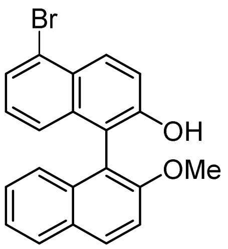

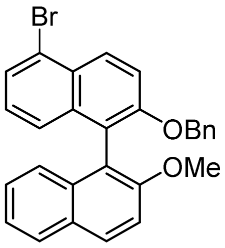

Following **General procedure A**, 5-bromo-2'-methoxy-[1,1'-binaphthalen]-2-ol (**24**) (56.9 mg, 0.150 mmol) was reacted for 24 h. Purification *via* flash column chromatography (3:7 CH_2Cl_2 – petroleum ether 40-60 to 4:1 CH_2Cl_2 – petroleum ether 40-60) afforded 2-(benzyloxy)-5-bromo-2'-methoxy-1,1'-binaphthalene (**Bn-24**) as an off-white solid (33.6 mg, 48%, 89:11 e.r.) and recovered starting material (**24**) (22.4 mg, 39%, 98:2 e.r.).

(S)-2-(benzyloxy)-5-bromo-2'-methoxy-1,1'-binaphthalene (Bn-24):

IR (film) ν_{max}/cm^{-1}: 3077, 3063, 3019, 2941, 1802, 1747, 1521, 1495, 1347, 1267, 1233, 1176, 1053, 749.

^{1}H NMR (500 MHz, $CDCl_3$) δ = 8.35 (d, *J* = 9.3 Hz, 1H), 8.02 (d, *J* = 9.0 Hz, 1H), 7.90 (d, *J* = 8.2 Hz, 1H), 7.62 (d, *J* = 7.3 Hz, 1H), 7.52 (d, *J* = 9.4 Hz, 1H), 7.47 (d, *J* = 9.0 Hz, 1H), 7.35 (dd, *J* = 8.1, 6.8 Hz, 1H), 7.25–7.21 (m, 1H), 7.20–7.15 (m, 4H), 7.12–7.04 (m, 2H), 7.04–6.93 (m, 2H), 5.35–4.88 (m, 2H), 3.77 (s, 3H).

^{13}C NMR (126 MHz, $CDCl_3$) δ = 155.0, 154.6, 137.3, 135.4, 134.0, 129.7, 129.2, 128.6, 128.2, 128.0, 127.8, 127.4, 126.7, 126.7, 126.5, 125.5, 125.2, 123.6, 122.9, 121.0, 118.9, 116.9, 113.7, 70.9, 56.6.

HRMS (ESI^+) $C_{28}H_{21}O_2{}^{79}Br$ $[M+Na]^+$ requires 491.06171; found 491.06180, Δ +0.2 ppm.

Chiral HPLC: (Chiralpak ADH, 10% ^{i}PrOH, 90% hexane, 1.0 mL min^{-1}, λ = 295 nm) τ_R (major) = 22.2 min, τ_R (minor) = 12.3 min.

$[\alpha]_D^{25}$ = −56.3 (*c* = 1.00, $CHCl_3$).

Data in agreement with literature reported.[11]

(R)-5-Bromo-2'-methoxy-[1,1'-binaphthalen]-2-ol (24):

m.p. = 109-110 °C (CH_2Cl_2/petroleum ether 40-60).

Chiral HPLC: (Chiralpak ADH, 10% ^{i}PrOH, 90% hexane, 1.0 mL min^{-1}, λ = 295 nm) τ_R (major) = 22.5 min, τ_R (minor) = 12.5 min.

$[\alpha]_D^{25}$ = +6.5 (*c* = 1.00, $CHCl_3$).

Kinetic resolution of 1-(2-methoxynaphthalen-1-yl)phenanthren-2-ol (25)

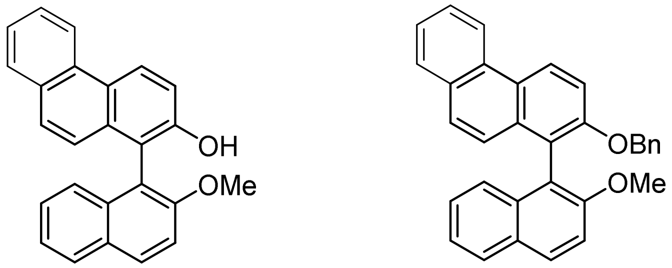

Following **General procedure A**, 1-(2-methoxynaphthalen-1-yl)phenanthren-2-ol (**25**) (52.6 mg, 0.150 mmol) was reacted for 24 h. Purification *via* flash column chromatography (3:7 CH_2Cl_2 – petroleum ether 40-60 to 4:1 CH_2Cl_2 – petroleum ether 40-60) afforded 2-(benzyloxy)-1-(2-methoxynaphthalen-1-yl)phenanthrene (**Bn-25**) as an off-white solid (33.0 mg, 50%, 87:13 e.r.) and recovered starting material (**25**) (21.0 mg, 40%, 99:1 e.r.).

Data in agreement with literature reported.[11]

(S)- 2-(benzyloxy)-1-(2-methoxynaphthalen-1-yl)phenanthrene (Bn-25):

m.p. = 130-132 °C (CH_2Cl_2/petroleum ether 40-60).

IR (film) ν_{max}/cm^{-1}: 3036, 2934, 1592, 1460, 1301, 1269, 1251, 1231, 1086, 1060, 810, 749.

^{1}H NMR (400 MHz, $CDCl_3$) δ = 8.77 (d, J = 9.1 Hz, 1H), 8.69 (d, J = 8.4 Hz, 1H), 8.03 (d, J = 9.0 Hz, 1H), 7.92 (d, J = 8.4 Hz, 1H), 7.80 (d, J = 7.9 Hz, 1H), 7.71–7.59 (m, 1H), 7.56–7.45 (m, 4H), 7.38–7.32 (m, 1H), 7.26–7.21 (m, 1H), 7.20–7.14 (m, 5H), 7.06–6.99 (m, 2H), 5.21–5.05 (m, 2H), 3.78 (s, 3H).

^{13}C NMR (101 MHz, $CDCl_3$) δ = 155.1, 137.7, 134.3, 133.0, 131.1, 130.8, 129.6, 129.3, 128.6, 128.3, 128.1, 127.6, 127.4, 126.8, 126.7, 126.5, 125.9, 125.5, 125.4, 124.7, 123.9, 123.6, 122.4, 122.3, 119.8, 114.7, 114.0, 70.9, 56.8.

HRMS (ESI$^+$) $C_{32}H_{24}O_2$ $[M+Na]^+$ requires 463.16685; found 463.16660, Δ −0.6 ppm.

Chiral HPLC: (Chiralpak IC, 1% ^{i}PrOH, 99% hexane, 1.0 mL min^{-1}, λ = 222 nm) τ_R (major) = 20.8 min, τ_R (minor) = 33.0 min.

$[\alpha]_D^{25}$ = −31.3 (c = 1.00, $CHCl_3$).

(R)- 1-(2-methoxynaphthalen-1-yl)phenanthren-2-ol (25):

m.p. = 62-64 °C (CH_2Cl_2/petroleum ether 40-60).

Chiral HPLC: (Chiralpak ADH, 15% ^{i}PrOH, 85% hexane, 1.0 mL min^{-1}, λ = 277 nm) τ_R (major) = 16.6 min, τ_R (minor) = 26.3 min.

$[\alpha]_D^{25}$ = −15.2 (c = 0.50, $CHCl_3$).

Kinetic resolution of 2'-methoxy-4-methyl-[1,1'-binaphthalen]-2-ol (26)

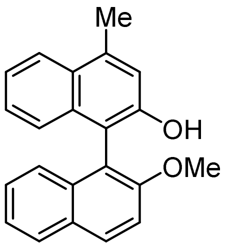

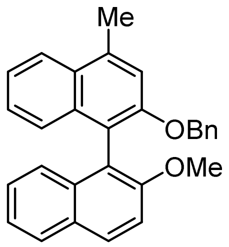

Following **General procedure A,** 2'-methoxy-4-methyl-[1,1'-binaphthalen]-2-ol (**26**) (49.2 mg, 0.150 mmol) was reacted for 36 h. Purification *via* flash column chromatography (3:7 CH_2Cl_2 – petroleum ether 40-60 to 4:1 CH_2Cl_2 – petroleum ether 40-60) afforded 2-(benzyloxy)-2'-methoxy-4-methyl-1,1'-binaphthalene (**Bn-26**) as an off-white solid (37.4 mg, 61%, 81:19 e.r.) and recovered starting material (**26**) (16.4 mg, 33%, >99.5:0.5 e.r.).

Data in agreement with literature reported.[11]

(S)- 2-(benzyloxy)-2'-methoxy-4-methyl-1,1'-binaphthalene (Bn-26):

m.p. = 54-55 °C (CH_2Cl_2/petroleum ether 40-60).

IR (film) ν_{max}/cm^{-1}: 3062, 3033, 2936, 2836, 1593, 1508, 1342, 1266, 1252, 1101, 754.

^{1}H NMR (400 MHz, $CDCl_3$) δ = 8.06–7.97 (m, 2H), 7.90 (d, *J* = 8.2 Hz, 1H), 7.47 (d, *J* = 9.1 Hz, 1H), 7.43–7.30 (m, 3H), 7.29–7.11 (m, 7H), 7.03–6.94 (m, 2H), 5.10–5.01 (m, 3H), 3.77 (s, 3H), 2.80 (s, 1H).

^{13}C NMR (101 MHz, $CDCl_3$) δ = 155.2, 153.7, 137.8, 136.0, 134.4, 134.4, 129.4, 129.3, 128.9, 128.2, 128.0, 127.4, 126.9, 126.4, 126.2, 125.6, 124.3, 123.7, 123.5, 119.7, 119.0, 117.5, 117.5, 114.0, 71.5, 56.8, 20.1.

HRMS (ESI$^+$) $C_{29}H_{24}O_2$ $[M+H]^+$ requires 405.18491; found 405.18494, Δ +0.1 ppm.

Chiral HPLC: (Chiralpak IC, 2% ^{i}PrOH, 98% hexane, 1.0 mL min^{-1}, λ = 291 nm) τ_R (major) = 15.6 min, τ_R (minor) = 24.7.

$[\alpha]_D^{25}$ = −33.4 (*c* = 1.00, $CHCl_3$).

(R)- 2'-methoxy-4-methyl-[1,1'-binaphthalen]-2-ol (26):

m.p. = 170-171 °C (CH_2Cl_2/petroleum ether 40-60).

Chiral HPLC: (Chiralpak ADH, 10% ^{i}PrOH, 90% hexane, 1.0 mL min^{-1}, λ = 291 nm) τ_R (major) = 15.6 min, τ_R (minor) = 20.7 min.

$[\alpha]_D^{25}$ = +37.8 (*c* = 1.00, $CHCl_3$).

Kinetic resolution of 2'-methoxy-4-phenyl-[1,1'-binaphthalen]-2-ol (27)

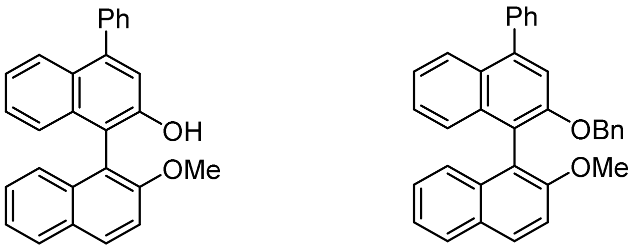

Following **General procedure A**, 2'-methoxy-4-phenyl-[1,1'-binaphthalen]-2-ol (**27**) (56.4 mg, 0.150 mmol) was reacted for 24 h. Purification *via* flash column chromatography (3:7 CH_2Cl_2 – petroleum ether 40-60 to 4:1 CH_2Cl_2 – petroleum ether 40-60) afforded 2-(benzyloxy)-2'-methoxy-4-phenyl-1,1'-binaphthalene (**Bn-27**) as an off-white solid (35.4 mg, 51%, 91:9 e.r.) and recovered starting material (**27**) (26.0 mg, 46%, 95:5 e.r.).

(S)-2-(Benzyloxy)-2'-methoxy-4-phenyl-1,1'-binaphthalene (Bn-27):

m.p. = 120-121 °C (CH_2Cl_2/petroleum ether 40-60).

IR (film) ν_{max}/cm^{-1}: 3066, 1585, 1342, 1188, 1024, 911, 766, 731, 705.

^{1}H NMR (400 MHz, $CDCl_3$) δ = 7.92 (d, J = 9.1 Hz, 1H), 7.86–7.77 (m, 2H), 7.49 (m, 2H), 7.46–7.41 (m, 2H), 7.40–7.34 (m, 2H), 7.31 (s, 1H), 7.24 (m, 1H), 7.20–7.11 (m, 5H), 7.04 (m, 3H), 6.86 (m, 2H), 5.02–4.91 (m, 2H), 3.69 (s, 3H).

^{13}C NMR (101 MHz, $CDCl_3$) δ = 155.2, 153.5, 141.7, 140.9, 137.6, 134.7, 134.3, 130.3, 129.6, 129.3, 128.4, 128.2, 128.0, 127.9, 127.5, 127.4, 127.0, 126.5, 126.3, 126.3, 125.9, 125.6, 124.0, 123.6, 120.5, 119.5, 117.6, 114.0, 71.5, 56.8.

HRMS (ESI^+) $C_{34}H_{27}O_2$ $[M+H]^+$ requires 467.20056; found 467.20050, Δ −0.1 ppm.

Chiral HPLC: (Chiralpak ADH, 2% ^{i}PrOH, 98% hexane, 1.0 mL min^{-1}, λ = 297 nm) τ_R (minor) = 14.8 min, τ_R (major) = 24.5 min.

$[\alpha]_D^{25}$ = −54.9 (c = 1.00, $CHCl_3$).

(R)- 2'-methoxy-4-phenyl-[1,1'-binaphthalen]-2-ol (27):

m.p. = 88-89 °C (CH_2Cl_2/petroleum ether 40-60).

Chiral HPLC: (Chiralpak ADH, 10% ^{i}PrOH, 90% hexane, 1.0 mL min^{-1}, λ = 295 nm) τ_R (minor) = 18.8 min, τ_R (major) = 26.2 min.

$[\alpha]_D^{25}$ = −1.5 (c = 1.00, $CHCl_3$).

Kinetic resolution of 4-bromo-2'-methoxy-[1,1'-binaphthalen]-2-ol (28)

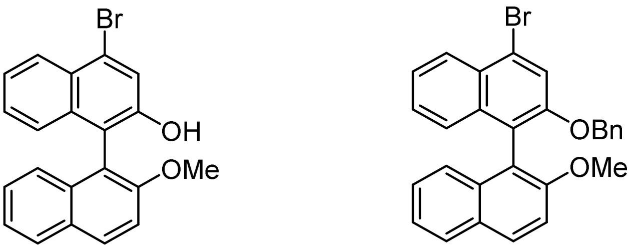

Following **General procedure A,** 4-bromo-2'-methoxy-[1,1'-binaphthalen]-2-ol (**28**) (56.9 mg, 0.150 mmol) was reacted for 24 h. Purification *via* flash column chromatography (3:7 CH_2Cl_2 – petroleum ether 40-60 to 4:1 CH_2Cl_2 – petroleum ether 40-60) afforded 2-(benzyloxy)-4-bromo-2'-methoxy-1,1'-binaphthalene (**Bn-28**) as an off-white solid (38.0 mg, 54%, 86:14 e.r.) and recovered starting material (**28**) (23.0 mg, 40%, 99:1 e.r.).

(S)- 2-(Benzyloxy)-4-bromo-2'-methoxy-1,1'-binaphthalene (n-28**):**

m.p. = 54-55 °C (CH_2Cl_2/petroleum ether 40-60).

IR (film) ν_{max}/cm^{-1}: 3062, 3033, 2930, 2837, 1583, 1501, 1453, 1315, 1270, 1261, 1251.

^{1}H NMR (400 MHz, $CDCl_3$) δ = 8.15 (d, *J* = 7.2 Hz, 1H), 7.91 (d, *J* = 9.0 Hz, 1H), 7.80 (d, *J* = 8.2 Hz, 1H), 7.68 (s, 1H), 7.37–7.31 (m, 2H), 7.26–7.21 (m, 1H), 7.20–7.09 (m, 3H), 7.08–7.00 (m, 4H), 6.89–6.83 (m, 2H), 5.04–4.87 (m, 2H), 3.66 (s, 3H).

^{13}C NMR (101 MHz, $CDCl_3$) δ = 154.9, 153.7, 137.0, 134.8, 133.9, 129.8, 129.1, 128.2, 128.1, 128.0, 127.5, 127.1, 127.1, 126.8, 126.5, 125.9, 125.2, 123.6, 123.2, 121.2, 120.6, 120.6, 118.5, 113.6, 71.6, 56.6.

HRMS (ESI^+) $C_{28}H_{21}O_2{}^{79}Br$ $[M+Na]^+$ requires 491.06171; found 491.06171, Δ 0.0 ppm.

Chiral HPLC: (Chiralpak IC, 1% ^{i}PrOH, 99% hexane, 1.0 mL min^{-1}, λ = 232 nm) τ_R (major) = 15.0 min, τ_R (minor) = 18.0 min.

$[\alpha]_D^{25}$ = −39.6 (*c* = 1.00, $CHCl_3$).

(R)- 4-Bromo-2'-methoxy-[1,1'-binaphthalen]-2-ol (28):

Chiral HPLC: (Chiralpak ADH, 10% ^{i}PrOH, 90% hexane, 1.0 mL min^{-1}, λ = 293 nm) τ_R (major) = 13.2 min, τ_R (minor) = 23.0 min.

Kinetic resolution of 2',4-dimethoxy-[1,1'-binaphthalen]-2-ol (29)

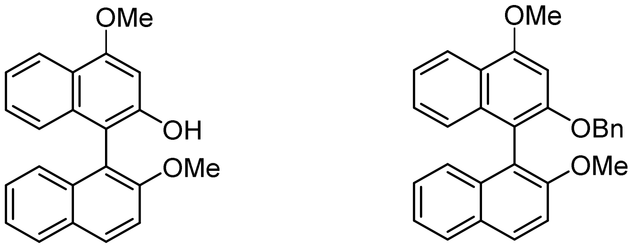

Following **General procedure A**, 2',4-dimethoxy-[1,1'-binaphthalen]-2-ol (**29**) (49.6 mg, 0.150 mmol) was reacted for 30 h. Purification *via* flash column chromatography (3:7 CH_2Cl_2 – petroleum ether 40-60 to 4:1 CH_2Cl_2 – petroleum ether 40-60) afforded 2-(benzyloxy)-2',4-dimethoxy-1,1'-binaphthalene (**Bn-29**) as an off-white solid (34.0 mg, 54%, 85:15 e.r.) and recovered starting material (**29**) (17.0 mg, 34%, 99:1 e.r.).

(S)- 2-(Benzyloxy)-2',4-dimethoxy-1,1'-binaphthalene (Bn-29):

m.p. = 125-126 °C (CH_2Cl_2/petroleum ether 40-60).

IR (film) ν_{max}/cm^{-1}: 2996, 2980, 2971, 2936, 1620, 1588, 1508, 1460, 1344, 1265, 1253, 1241, 1195, 1144, 1116, 1097, 1066, 1022, 906, 809, 764, 733, 697.

^{1}H NMR (400 MHz, $CDCl_3$) δ = 8.16 (d, *J* = 8.4 Hz, 1H), 7.89 (d, *J* = 9.0 Hz, 1H), 7.81–7.76 (m, 1H), 7.35 (d, *J* = 9.1 Hz, 1H), 7.22 (m, 2H), 7.17–7.10 (m, 3H), 7.09–7.01 (m, 4H), 6.91–6.86 (m, 2H), 6.70 (s, 1H), 4.90 (m, 2H), 3.92 (s, 3H), 3.66 (d, *J* = 1.1 Hz, 3H).

^{13}C NMR (101 MHz, $CDCl_3$) δ = 156.4, 155.3, 154.4, 137.7, 134.5, 134.5, 129.3, 129.2, 128.2, 127.9, 127.4, 127.0, 127.0, 126.3, 125.6, 125.2, 123.4, 123.2, 122.0, 122.0, 119.6, 114.0, 113.3, 96.8, 72.0, 56.7, 55.6.

Chiral HPLC: (Chiralpak IC, 3% ^{i}PrOH, x% hexane, 1.0 mL min^{-1}, λ = 292 nm) τ_R (major) = 6.1 min, τ_R (minor) = 8.2 min.

$[\alpha]_D^{25}$ = −46.0 (*c* = 1.00, $CHCl_3$).

(R)- 2',4-Dimethoxy-[1,1'-binaphthalen]-2-ol (29):

m.p. = 161-162 °C (CH_2Cl_2/petroleum ether 40-60).

Chiral HPLC: (Chiralpak ADH, 15% ^{i}PrOH, 85% hexane, 1.0 mL min^{-1}, λ = 231 nm) τ_R (minor) = 8.7 min, τ_R (major) = 12.7 min.

$[\alpha]_D^{25}$ = −2.0 (*c* = 1.00, $CHCl_3$).

Kinetic resolution of 7,7'-dibromo-2'-methoxy-[1,1'-binaphthalen]-2-ol (30)

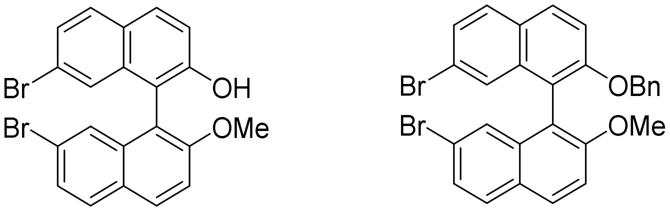

Following **General procedure A,** 7,7'-dibromo-2'-methoxy-[1,1'-binaphthalen]-2-ol (**30**) (68.7 mg, 0.150 mmol) was reacted for 48 h. Purification *via* flash column chromatography (3:1 petroleum ether 40-60/CH_2Cl_2 to 1:1 petroleum ether 40-60/CH_2Cl_2) afforded 2-(benzyloxy)-7,7'-dibromo-2'-methoxy-1,1'-binaphthalene (**Bn-30**) as an off-white solid (41.6 mg, 51%, 92.0:8.0 e.r.) and recovered starting material (**30**) (30.0 mg, 44%, 94.0:6.0 e.r.).

(S)-2-(benzyloxy)-7,7-dibromo-2'-methoxy-1,1'-binaphthalene (Bn-30)

m.p. = 66-68°C (CH_2Cl_2/petroleum ether 40-60).

IR (film) ν_{max}/cm^{-1}: 2935.6, 2360.5, 2341.5, 1612.9, 1583.0, 1496.4, 1456.24, 1343.2, 1321.1, 1256.3, 1343.2, 1321.1, 1256.3, 1221.9, 1171.5, 1151.9, 1095.9, 1067.1, 923.4, 872.5, 827.1, 796.2, 756.3, 695.7, 668.8.

^{1}H NMR (500 MHz, $CDCl_3$) δ_H: 7.96 (d, *J* = 9.0 Hz, 1H), 7.87 (d, *J* = 9.0 Hz, 1H), 7.75 (d, *J* = 8.6 Hz, 1H), 7.71 (d, *J* = 8.8 Hz), 7.45 (d, *J* = 9.2 Hz, 1H), 7.43-7.37 (m, 3H), 7.30-7.25 (m, 2H), 7.23-7.15 (m, 3H), 7.05-6.97 (m, 2H), 5.13-5.04 (m, 2H), 3.77 (s, 3H)

^{13}C NMR (126 MHz, $CDCl_3$) δ_C: 155.7, 154.7, 137.3, 135.4, 135.4, 129.9, 129.9, 129.7, 128.5, 127.9, 127.7, 127.6, 127.4, 127.2, 127.2, 126.8, 121.4, 121.3, 119.0, 118.0, 115.9, 114.0, 70.9, 56.6

HRMS (MSS-ESI$^+$) $C_{30}H_{20}{}^{79}Br_2O_2Na^+$ $[M+Na]^+$ requires 568.97463; found: 568.97223, Δ -4.2 ppm.

Chiral HPLC: (Chiralpak ADH, 5% ^{i}PrOH, 95% hexane, 1.00 mL min^{-1}, λ = 290 nm) τ_R (major) = 7.6 min, τ_R (minor) = 6.6 min

$[\alpha]_D^{25}$ = +13.9° (*c* = 1.00, $CHCl_3$).

(R)-7,7'-dibromo-2'-methoxy-[1,1'-binaphthalen]-2-ol (30)

m.p. = 116-118 °C (CH_2Cl_2/petroleum ether 40-60).

Chiral HPLC: (Chiralpak ADH, 5% ^{i}PrOH, 95% hexane, 1.00 mL min^{-1}, λ = 290 nm) τ_R (major) = 26.7 min, τ_R (minor) = 21.7 min

$[\alpha]_D^{25}$ = −156.2° (*c* = 1.00, $CHCl_3$).

Kinetic resolution of 2',7,7'-trimethoxy-[1,1'-binaphthalen]-2-ol (31)

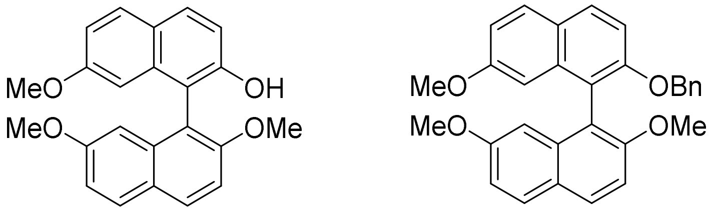

Following **General procedure A,** 2',7,7'-trimethoxy-[1,1'-binaphthalen]-2-ol (**31**) (54.1 mg, 0.150 mmol) was reacted for 27 h. Purification *via* flash column chromatography (2:1 petroleum ether 40-60 – CH_2Cl_2 to 1:2 petroleum ether 40-60 – CH_2Cl_2) afforded 2-(benzyloxy)-2',7,7'-trimethoxy-[1,1'-binaphthalen]-2-ol (**Bn-31**) as an off-white solid (35.1 mg, 52%, 89.5:10.5 e.r.) and recovered starting material (**31**) (24.2 mg, 45%, 94.0:6.0 e.r.).

(S)-2-(benzyloxy)-2',7,7'-trimethoxy-[1,1'-binaphthalen]-2-ol (Bn-31)

m.p. = 61-63 °C (CH_2Cl_2/petroleum ether 40-60).

IR (film) ν_{max}/cm^{-1}: 2924, 1621, 1509, 1458, 1261, 1220, 1029, 826, 734.

^{1}H NMR (500 MHz, $CDCl_3$) δ_H: 7.93 (d, *J* = 8.9 Hz, 1H), 7.85 (d, *J* = 8.9 Hz, 1H), 7.80 (d, *J* = 8.9 Hz, 1H), 7.77 (d, *J* = 8.9 Hz, 1H), 7.32 (d, *J* = 9.0 Hz, 1H), 7.29 (d, *J* = 9.6 Hz, 1H), 7.20-7.15 (m, 3H), 7.05-7.00 (m, 4H), 6.54 (d, *J* = 2.4 Hz, 1H), 6.47 (d, *J* = 2.4 Hz, 1H), 5.13-5.05 (m, 2H), 3.78 (s, 3H), 3.55 (s, 3H), 3.49 (s, 3H).

^{13}C NMR (126 MHz, $CDCl_3$) δ_C: 158.2, 158.2, 155.7, 154.7, 137.8, 135.4, 129.6, 129.6, 129.2, 129.1, 128.2, 127.4, 126.8, 125.2, 124.9, 119.9, 118.7, 116.3, 116.1, 113.6, 111.4, 104.1, 104.0, 71.2, 56.7, 55.2, 55.1

HRMS (ESI$^+$) $C_{30}H_{26}O_4Na^+$ [M+Na]$^+$ requires 473.17233; found: 473.17258, Δ +0.5 ppm.

Chiral HPLC: (Chiralpak ADH, 5% ^{i}PrOH, 95% hexane, 1.00 mL min^{-1}, λ = 290 nm) τ_R (major) = 27.4 min, τ_R (minor) = 11.6 min

$[\alpha]_D^{25}$ = +32.9 (*c* = 1.00, $CHCl_3$).

(R)-2',7,7'-trimethoxy-[1,1'-binaphthalen]-2-ol (31)

m.p. = 73-75 °C (CH_2Cl_2/petroleum ether 40-60).

Chiral HPLC: (Chiralpak ADH, 5% ^{i}PrOH, 95% hexane, 1.00 mL min^{-1}, λ = 290 nm) τ_R (major) = 47.8 min, τ_R (minor) = 40.7 min

$[\alpha]_D^{25}$ = −179.1(*c* = 1.00, $CHCl_3$).

Kinetic resolution of 2'-methoxy-7,7'-dimethyl-[1,1'-binaphthalen]-2-ol (32)

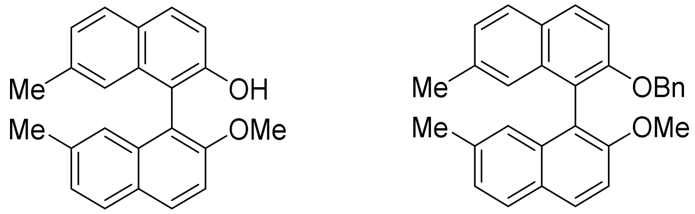

Following **General procedure A,** 2'-methoxy-7,7'-dimethyl-[1,1'-binaphthalen]-2-ol (**32**) (49.3 mg, 0.150 mmol) was reacted for 39 h. Purification *via* flash column chromatography (3:1 petroleum ether 40-60 – CH_2Cl_2 to 1:1 petroleum ether 40-60 – CH_2Cl_2) afforded 2-(benzyloxy)-2'-methoxy-7,7'-dimethyl-1,1'-binaphthalene (**Bn-32**) as an off-white solid (32.0 mg, 51%, 84.0:16.0 e.r.) and recovered starting material (**32**) (21.0 mg, 43%, 95.5:4.5 e.r.).

(S)-2-(benzyloxy)-2'-methoxy-7,7'-dimethyl-1,1'-binaphthalene (Bn-32)

m.p. = 55-57 °C (CH_2Cl_2/petroleum ether 40-60).

IR (film) ν_{max}/cm^{-1}: 3044, 2935, 2837, 1626, 1597, 1509, 1454, 1353, 1320, 1262, 1223, 1152, 1094, 1065, 826, 737, 696

^{1}H NMR (500 MHz, $CDCl_3$) δ_H: 7.96 (d, *J* = 8.9 Hz, 1H), 7.87 (d, *J* = 9.1 Hz, 1H), 7.81 (d, *J* = 8.3 Hz, 1H), 7.77 (d, *J* = 8.3 Hz, 1H), 7.40 (d, *J* = 8.9 Hz, 1H), 7.36 (d, *J* = 8.9 Hz, 1H), 7.22-7.13 (m, 5H), 7.05-6.90 (m, 4H), 5.10-4.99 (m, 2H), 3.75 (s, 3H), 2.28 (s, 3H), 2.26 (s, 3H)

^{13}C NMR (126 MHz, $CDCl_3$) δ_C: 155.2, 154.2, 137.9, 136.0, 136.0, 134.5, 129.1, 128.9, 128.2, 127.9, 127.8, 127.8, 127.6, 127.4, 126.9, 126.3, 126.0, 124.5, 124.4, 120.6, 119.3, 115.4, 113.1, 71.3, 56.8, 22.1, 22.1

HRMS (ESI^+) $C_{30}H_{27}O_2^+$ $[M+H]^+$ requires 419.20056; found: 419.20062 Δ +0.2 ppm.

Chiral HPLC: (Chiralpak ADH, 5% ^{i}PrOH, 95% hexane, 1.00 mL min^{-1}, λ = 290 nm) τ_R (major) = 8.4 min, τ_R (minor) = 5.5 min

$[\alpha]_D^{25}$ = −17.9 (*c* = 1.00, $CHCl_3$).

(R)-2'-methoxy-7,7'-dimethyl-[1,1'-binaphthalen]-2-ol (32)

m.p. = 76-78 °C (CH_2Cl_2/petroleum ether 40-60).

Chiral HPLC: (Chiralpak ADH, 5% ^{i}PrOH, 95% hexane, 1.00 mL min^{-1}, λ = 290 nm) τ_R (major) = 19.5 min, τ_R (minor) = 22.1 min

$[\alpha]_D^{25}$ = −84.0 (*c* = 1.00, $CHCl_3$).

Kinetic resolution of 6,6'-dibromo-2'-methoxy-[1,1'-binaphthalen]-2-ol (33)

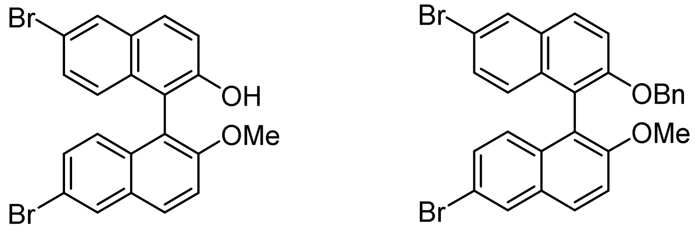

Following **General procedure A,** 6,6'-dibromo-2'-methoxy-[1,1'-binaphthalen]-2-ol (**33**) (68.7 mg, 0.150 mmol) was reacted for 42 h. Purification *via* flash column chromatography (3:1 petroleum ether 40-60/CH_2Cl_2 to 1:1 petroleum ether 40-60/CH_2Cl_2) afforded 2-(benzyloxy)-6,6'-dibromo-2'-methoxy-1,1'-binaphthalene (**Bn-33**) as an off-white solid (45.1 mg, 55%, 88.5:11.5 e.r.) and recovered starting material (**33**) (29.6 mg, 43%, 99.0:1.0 e.r.).

(S)-2-(benzyloxy)-6,6'-dibromo-2'-methoxy-1,1'-binaphthalene (Bn-33)

m.p. = 61-63 °C (CH_2Cl_2/petroleum ether 40-60).

IR (film) ν_{max}/cm^{-1}: 2917, 1584, 1494, 1327, 1268, 1069, 906, 730.

^{1}H NMR (500 MHz, $CDCl_3$) δ_H: 8.06 (d, *J* = 1.8 Hz, 1H, H_6), 8.03 (d, *J* = 1.7 Hz, 1H, H_{17}), 7.93 (d, *J* = 9.1 Hz, 1H, H_4), 7.84 (d, *J* = 9.0 Hz, 1H, H_{15}), 7.48 (d, *J* = 9.1 Hz, 1H, H_3), 7.46 (d, *J* = 9.1 Hz, 1H, H_{14}), 7.33-7.27 (m, 2H, H_8 and H_{19}), 7.22-7.17 (m, 3H, H_{24} and H_{26}), 7.03-6.96 (m, 4H, H_9, H_{20} and H_{25}), 5.09 (s, 2H, H_{22}), 3.78 (s, 3H, H_{11}).

^{13}C NMR (126 MHz, $CDCl_3$) δ_C: 155.2, 154.3, 137.2, 132.51, 132.48, 130.5, 130.2, 129.92, 129.87, 129.8, 128.8, 128.6, 128.3, 127.5, 127.0, 126.7, 120.3, 119.0, 117.7, 117.3, 116.9, 114.7, 71.1, 56.6.

HRMS ($APCI^-$) $C_{28}H_{21}{}^{79}Br_2O_2^-$ $[M\text{-}H]^-$ requires 546.9914; found: 546.9907, Δ -1.10 ppm.

Chiral HPLC: (Chiralpak ADH, 7.5% ^{i}PrOH, 92.5% hexane, 1.00 mL min^{-1}, λ = 290 nm) τ_R (major) = 13.3 min, τ_R (minor) = 10.8 min.

$[\alpha]_D^{25}$ = −9.5° (*c* = 1.00, $CHCl_3$)

(R)-6,6'-Dibromo-2'-methoxy-[1,1'-binaphthalen]-2-ol (33)

m.p. = 82-84 °C (CH_2Cl_2/petroleum ether 40-60).

Chiral HPLC: (Chiralpak ADH, 7.5% ^{i}PrOH, 92.5% hexane, 1.00 mL min^{-1}, λ = 290nm) τ_R (major) = 25.4 min, τ_R (minor) = 20.2 min.

$[\alpha]_D^{25}$ = −103.3 (*c* = 1.00, $CHCl_3$).

Kinetic resolution of 2'-Methoxy-6,6'-diphenyl-[1,1'-binaphthalen]-2-ol

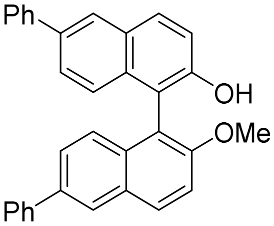

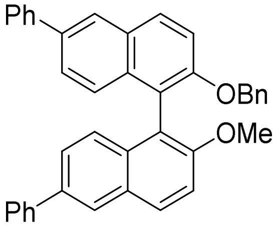

Following **General procedure A,** 2'-Methoxy-6,6'-diphenyl-[1,1'-binaphthalen]-2-ol (**34**) (67.9 mg, 0.150 mmol) was reacted for 24 h. Purification *via* flash column chromatography (3:1 petroleum ether 40-60/CH_2Cl_2 to 1:1 petroleum ether 40-60/CH_2Cl_2) afforded 2-(Benzyloxy)-2'-methoxy-6,6'-diphenyl-1,1'-binaphthalene (**Bn-34**) as an off-white solid (36.8 mg, 45%, 93.0:7.0 e.r.) and recovered starting material (**34**) (32.2 mg, 47%, 95.0:5.0 e.r.)

(S)-2-(Benzyloxy)-2'-methoxy-6,6'-diphenyl-1,1'-binaphthalene (Bn-34)

m.p. = 98-100 °C (CH_2Cl_2/petroleum ether 40-60).

IR (film) ν_{max}/cm^{-1}: 3028, 2917, 1622, 1592, 1491, 1454, 1251, 1223, 1176, 1096, 1061, 1028, 757, 732, 695.

^{1}H NMR (500 MHz, $CDCl_3$) δ_H: 8.15 (s, 1H), 8.13-8.09 (m, 2H), 8.02 (d, *J* = 9.0 Hz, 1H), 7.76-7.69 (m, 4H), 7.58 – 7.53 (m, 3H), 7.52-7.46 (m, 5H), 7.40-7.28 (m, 4H), 7.23-7.18 (m, 3H), 7.10-7.05 (m, 2H), 5.14 (s, 2H), 3.83 (s, 3H)

^{13}C NMR (126 MHz, $CDCl_3$) δ_C: 155.2, 154.2, 141.3, 141.2, 137.6, 136.5, 136.2, 133.4, 129.9, 129.73, 129.67, 129.4, 128.8, 128.2, 127.4, 127.27, 127.25, 127.08, 127.06, 126.8, 126.2, 126.0, 125.93, 125.88, 120.6, 119.3, 116.6, 114.3, 71.3, 56.7.

HRMS (ESI$^+$) $C_{40}H_{30}O_2Na^+$ [M+Na]$^+$ requires 565.21380, found: 565.21381, Δ +0.0 ppm.

Chiral HPLC: (Chiralpak ADH, 12.5% ^{i}PrOH, 87.5% hexane, 1.00 mL min^{-1}, λ = 290 nm) τ_R (major) = 12.3 min, τ_R (minor) = 19.5 min

$[\alpha]_D^{25}$ = +70.5 (*c* = 1.00, $CHCl_3$).

(R)-2'-Methoxy-6,6'-diphenyl-[1,1'-binaphthalen]-2-ol (34)

m.p. = 120-122 °C (CH_2Cl_2/petroleum ether 40-60).

Chiral HPLC: (Chiralpak ADH, 12.5% ^{i}PrOH, 87.5% hexane, 1.00 mL min^{-1}, λ = 290 nm) τ_R (major) = 25.6 min, τ_R (minor) = 17.7 min

$[\alpha]_D^{25}$ = −189.4 (*c* = 1.00, $CHCl_3$).

Kinetic resolution of 6,6'-bis(4-fluorophenyl)-2'-methoxy-[1,1'-binaphthalen]-2-ol (35)

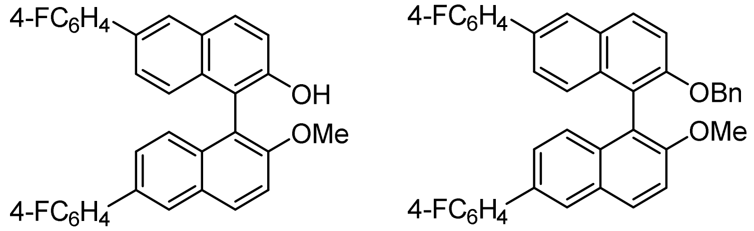

Following **General procedure A,** 6,6'-bis(4-fluorophenyl)-2'-methoxy-[1,1'-binaphthalen]-2-ol (**35**) (73.3 mg, 0.150 mmol) was reacted for 24 h. Purification *via* flash column chromatography (3:1 petroleum ether 40-60 – CH_2Cl_2 to 1:1 petroleum ether 40-60 – CH_2Cl_2) afforded 2-(Benzyloxy)-6,6'-bis(4-fluorophenyl)-2'-methoxy-1,1'-binaphthalene (**Bn-35**) as an off-white solid (49.7 mg, 57%, 85.0:15.0 e.r.) and recovered starting material (**35**) (30.6 mg, 42%, 99.5:0.5 e.r.).

(S)-2-(Benzyloxy)-6,6'-bis(4-fluorophenyl)-2'-methoxy-1,1'-binaphthalene (Bn-35)

m.p. = 185-187 °C (CH_2Cl_2/petroleum ether 40-60).

IR (film) ν_{max}/cm^{-1}: 2918, 1602, 1514, 1496, 1251, 1224, 1159, 1096, 1061, 820, 735.

^{1}H NMR (500 MHz, $CDCl_3$) δ_H: 8.11-8.06 (m, 2H), 8.04 (d, *J* = 1.7 Hz, 1H), 8.00 (d, *J* = 9.0 Hz, 1H), 7.69-7.61 (m, 4H), 7.53 (d, *J* = 9.0 Hz, 1H), 7.51-7.44 (m, 3H), 7.32-7.24 (m, 2H), 7.21-7.13 (m, 7H), 7.08-7.03 (m, 2H), 5.13 (s, 2H), 3.82 (s, 3H).

^{13}C NMR (126 MHz, $CDCl_3$) δ_C: 162.3 (d, *J* = 246 Hz), 155.2, 154.3, 137.5, 137.3 (app t, *J* = 4 Hz), 135.5, 135.2, 133.3, 129.8, 129.65, 129.60, 129.3, 128.7 (app dd, *J* = 8.0, 2.5 Hz), 128.2, 127.4, 126.8, 126.1, 126.0, 125.8, 125.7, 120.6, 119.2, 116.7, 115.7 (d, *J* = 21.3 Hz), 114.3, 71.3, 56.7.

^{19}F NMR (470 MHz, $CDCl_3$, ^{1}H decoupled) δ_F: -116.0 (s), -116.1 (s).

HRMS (ESI^+) $C_{40}H_{28}F_2O_2Na^+$ $[M+Na]^+$: requires 601.19496, found: 601.19489, Δ -0.1 ppm.

Chiral HPLC: (Chiralpak ADH, 12.5% ^{i}PrOH, 87.5% hexane, 1.00 mL min^{-1}, λ = 290 nm) τ_R (major) = 16.6 min, τ_R (minor) = 26.6 min

$[\alpha]_D^{25}$ = +48.4 (*c* = 1.00, $CHCl_3$).

(R)-2'-Methoxy-6,6'-bis(4-(trifluoromethoxy)phenyl)-[1,1'-binaphthalen]-2-ol (35)

m.p. = 113-115 °C (CH_2Cl_2/petroleum ether 40-60).

Chiral HPLC: (Chiralpak ADH, 12.5% ^{i}PrOH, 87.5% hexane, 1.00 mL min^{-1}, λ = 290 nm) τ_R (major) = 41.2 min, τ_R (minor) = 24.3 min

$[\alpha]_D^{25}$ = -231.6 (*c* = 1.00, $CHCl_3$).

Kinetic resolution of 1,1'-((2-hydroxy-2'-methoxy-[1,1'-binaphthalene]-6,6'-diyl)bis(4,1-phenylene))bis(ethan-1-one) (36)

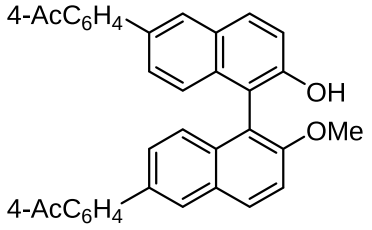

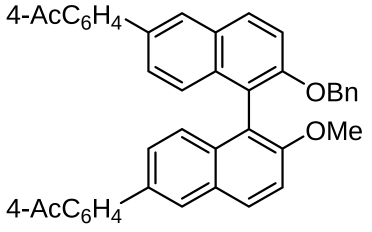

Following **General procedure A,** 1,1'-((2-hydroxy-2'-methoxy-[1,1'-binaphthalene]-6,6'-diyl)bis(4,1-phenylene))bis(ethan-1-one) (**36**) (80.5 mg, 0.150 mmol) was reacted for 30 h. Purification *via* flash column chromatography (19:1 CH_2Cl_2 – diethyl ether to 9:1 CH_2Cl_2 – diethyl ether) afforded 1,1'-((2-(benzyloxy)-2'-methoxy-[1,1'-binaphthalene]-6,6'-diyl)bis(4,1-phenylene))bis(ethan-1-one) (**Bn-36**) as an off-white solid (50.0 mg, 53%, 90.5:9.5 e.r.) and recovered starting material (**36**) (34.3 mg, 43%, 97.0:3.0 e.r.)

(S)-1,1'-((2-(benzyloxy)-2'-methoxy-[1,1'-binaphthalene]-6,6'-diyl)bis(4,1-phenylene))bis(ethan-1-one) (Bn-36)

m.p. = 128-130 °C (CH_2Cl_2/diethyl ether).

IR (film) ν_{max}/cm^{-1}: 2923, 2851, 1677, 1599, 1493, 1268, 1250, 1059, 818, 732.

^{1}H NMR (500 MHz, $CDCl_3$) δ_H: 8.19 (s, 1H), 8.16 (s, 1H), 8.12 (d, *J* = 9.0 Hz), 8.10-8.00 (m, 5H), 7.84-7.76 (m, 4H), 7.58-7.49 (m, 4H), 7.35-7.25 (m, 2H), 7.24-7.15 (m, 3H), 7.10-7.02 (m, 2H), 5.15 (s, 2H), 3.83 (s, 3H), 2.67 (s, 3H), 2.66 (s, 3H).

^{13}C NMR (126 MHz, $CDCl_3$) δ_C: 197.8, 155.6, 154.6, 145.8, 145.7, 137.4, 135.7, 135.6, 135.0, 134.8, 133.8, 130.2, 130.0, 129.6, 129.3, 129.0, 128.2, 127.5, 127.20, 127.18, 126.7, 126.6, 126.5, 126.2, 125.7, 120.4, 119.1, 116.6, 114.4, 71.2, 56.6, 26.7.

HRMS (ESI$^+$) $C_{44}H_{34}O_4Na^+$ [M+Na]$^+$: requires 649.23493, found: 649.23499, Δ +0.1 ppm

Chiral HPLC: (Chiralpak ADH, 50% ^{i}PrOH, 50% hexane, 1.00 mL min^{-1}, λ = 290 nm) τ_R (major) = 37.6 min, τ_R (minor) = 42.0 min

$[\alpha]_D^{25}$ = +135.3 (*c* = 1.00, $CHCl_3$).

(R)-1,1'-((2-hydroxy-2'-methoxy-[1,1'-binaphthalene]-6,6'-diyl)bis(4,1-phenylene))bis(ethan-1-one) (36)

m.p. = 161-163 °C (CH_2Cl_2/diethyl ether).

Chiral HPLC: (Chiralpak ADH, 50% ^{i}PrOH, 50% hexane, 1.00 mL min^{-1}, λ = 290 nm) τ_R (major) = 27.8 min, τ_R (minor) = 17.7 min

$[\alpha]_D^{25}$ = −316.1 (*c* = 1.00, $CHCl_3$).

Kinetic resolution of 2'-methoxy-6,6'-bis(4-(trifluoromethoxy)phenyl)-[1,1'-binaphthalen]-2-ol (37)

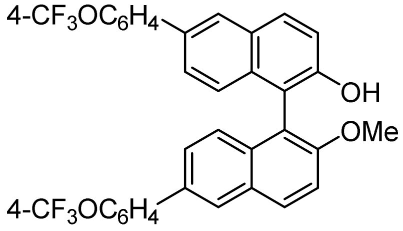

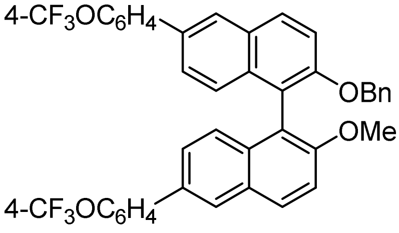

Following **General procedure A,** 2'-methoxy-6,6'-bis(4-(trifluoromethoxy)phenyl)-[1,1'-binaphthalen]-2-ol (**37**) (93.1 mg, 0.150 mmol) was reacted for 24 h. Purification *via* flash column chromatography (3:1 petroleum ether 40-60 – CH_2Cl_2 to 1:1 petroleum ether 40-60 – CH_2Cl_2) afforded 2-(Benzyloxy)-2'-methoxy-6,6'-bis(4-(trifluoromethoxy)phenyl)-1,1'-binaphthalene (**Bn-37**) as an off-white solid (61.2 mg, 57%, 85:15 e.r.) and recovered starting material (**37**) (37.9 mg, 41%, 99.5:0.5 e.r.)

(S)-2-(Benzyloxy)-2'-methoxy-6,6'-bis(4-(trifluoromethoxy)phenyl)-1,1'-binaphthalene (Bn-37)

m.p. = 85-87 °C (CH_2Cl_2/petroleum ether 40-60).

IR (film) ν_{max}/cm^{-1}: 2917, 1597, 1514, 1496, 1252, 1222, 1167, 907, 730.

^{1}H NMR (500 MHz, $CDCl_3$) δ_H: 8.12-8.08 (m, 2H), 8.06 (d, *J* = 1.6 Hz, 1H), 8.01 (d, *J* = 9.0 Hz, 1H), 7.74-7.66 (m, 4H), 7.54 (d, *J* = 9.1 Hz, 1H), 7.52-7.44 (m, 3H), 7.36-7.24 (m, 6H), 7.22-7.15 (m, 3H), 7.09-7.02 (m, 2H), 5.13 (s, 2H), 3.83 (s, 3H).

^{13}C NMR (126 MHz, $CDCl_3$) δ_C: 155.4, 154.4, 148.5, 140.0, 139.9, 137.4, 135.1, 134.8, 133.4, 129.9, 129.7, 129.6, 129.3, 128.49, 128.47, 128.2, 127.4, 126.7, 126.11, 126.08, 126.03, 125.8, 121.3, 120.6 (q, *J* = 257.1 Hz), 120.5, 119.5, 119.1, 116.7, 114.4, 71.2, 56.6.

^{19}F NMR (470 MHz, $CDCl_3$) δ_F: -57.79 (s).

HRMS (ESI$^+$) $C_{42}H_{28}F_6O_4Na^+$ $[M+Na]^+$: requires 744.17840, found: 744.17834, Δ -0.1 ppm.

Chiral HPLC: (Chiralpak IC, 1% ^{i}PrOH, 99% hexane, 1.00 mL min^{-1}, λ = 290 nm) τ_R (major) = 6.9 min, τ_R (minor) = 9.6 min

$[\alpha]_D^{25}$ = +49.0 (*c* = 1.00, $CHCl_3$).

(R)-2'-methoxy-6,6'-bis(4-(trifluoromethoxy)phenyl)-[1,1'-binaphthalen]-2-ol (37)

m.p. = 93-95 °C (CH_2Cl_2/petroleum ether 40-60).

Chiral HPLC: (Chiralpak ADH, 12.5% ^{i}PrOH, 87.5% hexane, 1.00 mL min^{-1}, λ = 290 nm) τ_R (major) = 16.6 min, τ_R (minor) = 12.4 min

$[\alpha]_D^{25}$ = −172.8 (*c* = 1.00, $CHCl_3$).

Kinetic resolution of 5,5'-Dibromo-2'-methoxy-[1,1'-binaphthalen]-2-ol (38)

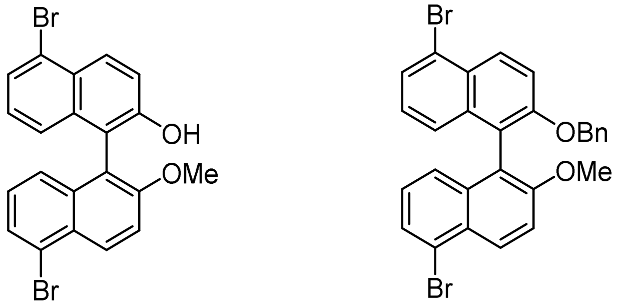

Following **General procedure A,** 5,5'-Dibromo-2'-methoxy-[1,1'-binaphthalen]-2-ol (**38**) (68.7 mg, 0.150 mmol) was reacted for 48 h. Purification *via* flash column chromatography (3:1 petroleum ether 40-60/CH_2Cl_2 to 1:1 petroleum ether 40-60/CH_2Cl_2) afforded 2-(benzyloxy)-5,5'-dibromo-2'-methoxy-1,1'-binaphthalene (**Bn-38**) as an off-white solid (43.4 mg, 53%, 87.5:12.5 e.r.) and recovered starting material (**38**) (29.4 mg, 43%, 98.0:2.0 e.r.).

(S)-2-(benzyloxy)-5,5'-dibromo-2'-methoxy-1,1'-binaphthalene (Bn-38)

m.p. = 62-64 °C (CH_2Cl_2/petroleum ether 40-60).

IR (film) ν_{max}/cm^{-1}: 2935.9, 2360.2, 2341.5, 1611.3, 1588.1, 1560.7, 1497.2, 1454.9, 1394.1, 1347.2, 1324.7, 1264.5, 1216.4, 1200.4, 1140.0, 1100.5, 1072.5, 1049.6, 965.1, 798.7, 753.8, 693.0, 655.7

^{1}H NMR (500 MHz, $CDCl_3$) δ_H: 8.44 (d, *J* = 9.4Hz, 1H), 8.35 (d, *J* = 9.4 Hz), 7.64-7.60 (m, 2H), 7.54 (d, *J* = 9.4 Hz, 1H), 7.50 (d, *J* = 9.4 Hz, 1H), 7.20-7.16 (m, 3H), 7.10-6.99 (m, 6H), 5.15-5.06 (m, 2H), 3.78 (s, 3H).

^{13}C NMR (126 MHz, $CDCl_3$) δ_C: 155.7, 154.7, 137.3, 135.4, 135.3, 129.2, 129.0, 128.4, 128.0, 127.9, 127.8, 127.7, 127.6, 126.9, 126.8, 125.4, 125.3, 123.1, 123.1, 120.5, 119.3, 116.8, 114.7, 71.0, 56.6.

HRMS ($APCI^-$) $C_{30}H_{20}{}^{79}Br_2O_2Na^+$ $[M+Na]^+$ requires 568.97463; found: 568.97223, Δ -4.2 ppm.

Chiral HPLC: (Chiralpak IC, 2% ^{i}PrOH, 98% hexane, 1.00 mL min^{-1}, λ = 290 nm) τ_R (major) = 5.2 min, τ_R (minor) = 6.9 min

$[\alpha]_D^{25}$ = −103.3 (*c* = 1.00, $CHCl_3$).

(R)-5,5'-Dibromo-2'-methoxy-[1,1'-binaphthalen]-2-ol (38)

m.p. = 94-96 °C (CH_2Cl_2/petroleum ether 40-60).

Chiral HPLC: (Chiralpak IC, 2% ^{i}PrOH, 98% hexane, 1.00 mL min^{-1}, λ = 290 nm) τ_R (major) = 11.8 min, τ_R (minor) = 23.1 min

$[\alpha]_D^{25}$ = +48.1 (*c* = 1.00, $CHCl_3$).

Kinetic resolution of 2'-methoxy-5,5'-dimethyl-[1,1'-binaphthalen]-2-ol (39)

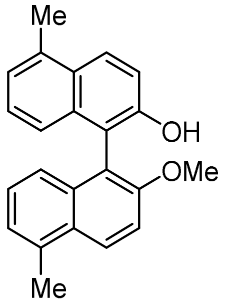

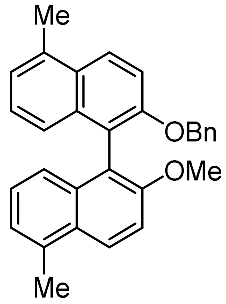

Following **General procedure A,** 2'-methoxy-5,5'-dimethyl-[1,1'-binaphthalen]-2-ol (**39**) (49.3 mg, 0.150 mmol) was reacted for 21 h. Purification *via* flash column chromatography (3:1 petroleum ether 40-60/CH_2Cl_2 to 1:1 petroleum ether 40-60/CH_2Cl_2) afforded 2-(benzyloxy)-2'-methoxy-5,5'-dimethyl-1,1'-binaphthalene (**Bn-39**) as an off-white solid (30.0 mg, 48%, 93.0:7.0 e.r.) and recovered starting material (**39**) (25.5 mg, 52%, 93.0:7.0 e.r.).

(S)-2-(benzyloxy)-2'-methoxy-5,5'-dimethyl-1,1'-binaphthalene (Bn-39)

m.p. = 60-62 °C (CH_2Cl_2/petroleum ether 40-60).

IR (film) ν_{max}/cm^{-1}: 3062, 3031, 2935, 2837, 1614, 1594, 1511, 1454, 1401, 1325, 1265, 1223, 1091, 1077, 1063, 1038, 800, 758, 696

^{1}H NMR (500 MHz, $CDCl_3$) δ_H: 8.19 (d, *J* = 9.22 Hz, 1H), 8.10 (d, *J* = 9.22 Hz, 1H), 7.51 (d, *J* = 9.22 Hz, 1H), 7.45 (d, *J* = 9.22 Hz, 1H), 7.22-7.17 (m, 5H), 7.16-7.10 (m, 2H), 7.09-7.01 (m, 4H), 5.14-5.04 (m, 2H), 3.79 (s, 3H), 2.80 (s, 3H), 2.76 (s, 3H).

^{13}C NMR (126 MHz, $CDCl_3$) δ_C: 154.9, 153.9, 137.9, 134.5, 134.5, 134.3, 134.3, 128.7, 128.4, 128.2, 127.4, 126.9, 126.3, 125.6, 125.5, 124.8, 124.5, 124.2, 124.1, 121.7, 120.4, 115.8, 113.4, 71.2, 56.7, 19.8, 19.8.

HRMS (ESI^+) $C_{30}H_{27}O_2^+$ $[M+H]^+$ requires 419.20056, found: 419.20060, Δ +0.10 ppm.

Chiral HPLC: (Chiralpak ADH, 12.5% ^{i}PrOH, 87.5% hexane, 1.00 mL min^{-1}, λ = 290 nm) τ_R (major) = 8.9 min, τ_R (minor) = 8.2 min.

$[\alpha]_D^{25}$ = −53.0 (*c* = 1.00, $CHCl_3$).

(R)-2'-methoxy-5,5'-dimethyl-[1,1'-binaphthalen]-2-ol (39)

m.p. = 76-78 °C (CH_2Cl_2/petroleum ether 40-60).

Chiral HPLC: (Chiralpak ADH, 12.5% ^{i}PrOH, 87.5% hexane, 1.00 mL min^{-1}, λ = 290 nm) τ_R (major) = 29.1 min, τ_R (minor) = 9.7 min.

$[\alpha]_D^{25}$ = +2.6 (*c* = 1.00, $CHCl_3$).

Kinetic resolution of 2'-methoxy-5,5'-diphenyl-[1,1'-binaphthalen]-2-ol (40)

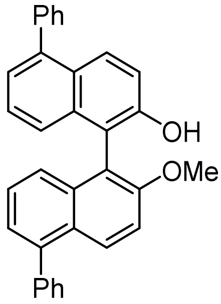

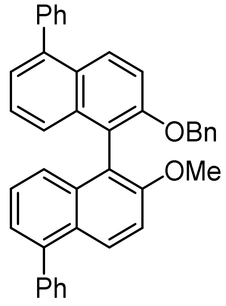

Following **General procedure A,** 2'-methoxy-5,5'-diphenyl-[1,1'-binaphthalen]-2-ol (**40**) (67.9 mg, 0.150 mmol) was reacted for 36 h. Purification *via* flash column chromatography (3:1 petroleum ether 40-60/CH_2Cl_2 to 1:1 petroleum ether 40-60/CH_2Cl_2) afforded 2-(benzyloxy)-2'-methoxy-5,5'-diphenyl-1,1'-binaphthalene (**Bn-40**) as an off-white solid (39.8 mg, 49%, 81.5:18.5 e.r.) and recovered starting material (**40**) (25.3 mg, 37%, 95.0:5.0 e.r.).

(S)-2-(benzyloxy)-2'-methoxy-5,5'-diphenyl-1,1'-binaphthalene (Bn-40)

m.p. = 97-99 °C (CH_2Cl_2/petroleum ether 40-60).

IR (film) ν_{max}/cm^{-1}: 3058, 3030, 2933, 2838, 2359, 2336, 2211, 2155, 1947, 1890, 1726, 1609, 1590, 1511, 1491, 1475, 1461, 1454, 1444, 1402, 1371, 1360, 1322, 1263, 1226, 1179, 1156, 1121, 1089, 1044, 1028, 971, 922, 906, 847, 806, 759, 702, 667, 647, 620

^{1}H NMR (500 MHz, $CDCl_3$) δ_H: 8.09 (d, *J* = 9.44 Hz, 1H), 8.01 (d, *J* = 9.18 Hz, 1H), 7.66-7.44 (m, 10H), 7.43 (d, *J* = 9.44 Hz, 1H), 7.38 (d, *J* = 9.18 Hz, 1H), 7.34-7.27 (m, 5H), 7.26-7.23 (m, 1H), 7.20-7.15 (m, 3H), 7.06-7.00 (m, 2H), 5.15-5.05 (m, 2H), 3.80 (s, 3H).

^{13}C NMR (126 MHz, $CDCl_3$) δ_C: 155.0, 154.0, 141.4, 141.3, 140.4, 140.4, 137.8, 134.7, 134.7, 130.4, 130.3, 128.4, 128.3, 128.3, 127.9, 127.8, 127.7, 127.5, 127.5, 127.3, 127.3, 126.8, 126.1, 125.3, 125.1, 124.8, 121.1, 119.8, 116.0, 113.7, 71.2, 56.7.

HRMS (ESI$^+$) $C_{40}H_{30}O_2Na^+$ [M+Na]$^+$ requires 565.21380; found: 565.21362, Δ -0.3 ppm.

Chiral HPLC: (Chiralpak IC, 2% ^{i}PrOH, 99% hexane, 1.00 mL min^{-1}, λ = 290 nm) τ_R (major) = 5.0 min, τ_R (minor) = 6.2 min

$[\alpha]_D^{25}$ = −69.7 (*c* = 1.00, $CHCl_3$).

(R)-2'-methoxy-5,5'-diphenyl-[1,1'-binaphthalen]-2-ol (40)

m.p. = 117-119 °C (CH_2Cl_2/petroleum ether 40-60).

Chiral HPLC: (Chiralpak IC, 2% ^{i}PrOH, 98% hexane, 1.00 mL min^{-1}, λ = 290 nm) τ_R (major) = 8.0 min, τ_R (minor) = 14.9 min

$[\alpha]_D^{25}$ = +73.2 (*c* = 0.50, $CHCl_3$).

Kinetic resolution of 4,4'-dibromo-2'-methoxy-[1,1'-binaphthalen]-2-ol (41)

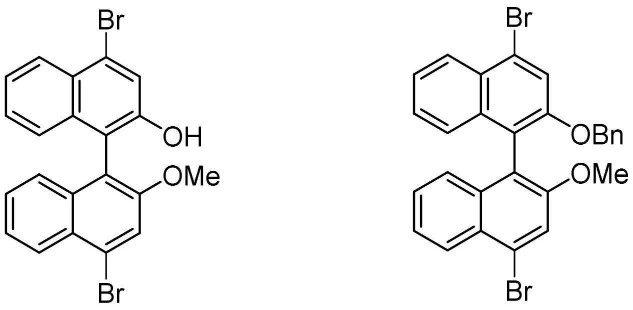

Following **General procedure A**, 4,4'-dibromo-2'-methoxy-[1,1'-binaphthalen]-2-ol (**41**) (68.7 mg, 0.150 mmol) was reacted for 36 h. Purification *via* flash column chromatography (3:7 CH_2Cl_2 – petroleum ether 40-60 to 4:1 CH_2Cl_2 – petroleum ether 40-60) afforded 2-(benzyloxy)-4,4'-dibromo-2'-methoxy-1,1'-binaphthalene (**Bn-41**) as an off-white solid (30.7 mg, 45%, 88:12 e.r.) and recovered starting material (**41**) (41.4 mg, 50%, 93:7 e.r.).

(S)-2-(Benzyloxy)-4,4'-dibromo-2'-methoxy-1,1'-binaphthalene (Bn-41):

m.p. = 148-150 °C (CH_2Cl_2/petroleum ether 40-60).

IR (film) ν_{max}/cm^{-1}: 3064, 2934, 1582, 1497, 1314, 1259, 1243, 1025, 907, 756, 733.

^{1}H NMR (400 MHz, $CDCl_3$) δ = 8.35–8.19 (m, 3H), 7.79–7.76 (m, 2H), 7.52–7.39 (m, 2H), 7.32–7.22 (m, 2H), 7.21–7.11 (m, 5H), 7.05–6.92 (m, 2H), 5.12–4.92 (m, 2H), 3.75 (s, 3H).

^{13}C NMR (101 MHz, $CDCl_3$) δ = 154.7, 153.8, 136.9, 134.7, 128.4, 128.2, 127.9, 127.8, 127.4, 127.3, 126.9, 125.8, 125.8, 125.4, 125.1, 123.9, 123.7, 120.4, 120.2, 118.9, 118.2, 71.6, 56.9.

HRMS (ESI^+) $C_{28}H_{20}O_2{}^{79}Br$ $[M+Na]^+$ requires 568.97223; found 568.97217, Δ −0.1 ppm.

Chiral HPLC: (Chiralpak ADH, 1% ^{i}PrOH, 99% hexane, 1.0 mL min^{-1}, λ = 295 nm) τ_R (major) = 16.2 min, τ_R (minor) = 11.1 min.

$[\alpha]_D^{25}$ = −47.2 (c = 0.50, $CHCl_3$).

(R)-4,4'-Dibromo-2'-methoxy-[1,1'-binaphthalen]-2-ol (41):

m.p. = 178-180 °C (CH_2Cl_2/petroleum ether 40-60).

Chiral HPLC: (Chiralpak ADH, 10% ^{i}PrOH, 90% hexane, 1.0 mL min^{-1}, λ = 293 nm) τ_R (major) = 15.3 min, τ_R (minor) = 31.2 min.

$[\alpha]_D^{25}$ = +23.4 (c = 0.50, $CHCl_3$).

Kinetic resolution of [2'-methoxy-4,4'-diphenyl-[1,1'-binaphthalen]-2-ol] (42)

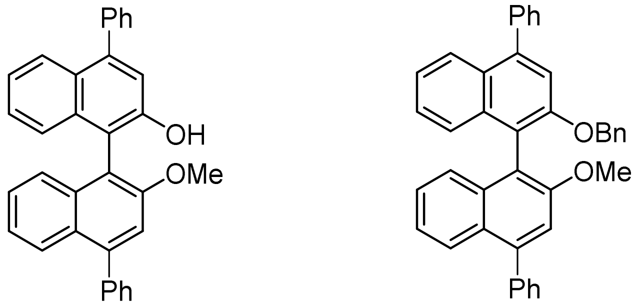

Following **General procedure A,** 2'-methoxy-4,4'-diphenyl-[1,1'-binaphthalen]-2-ol (**42**) (67.9 mg, 0.150 mmol) for 30 h. Purification *via* flash column chromatography (3:7 CH_2Cl_2 – petroleum ether 40-60 to 4:1 CH_2Cl_2 – petroleum ether 40-60) afforded 2-(benzyloxy)-2'-methoxy-4,4'-diphenyl-1,1'-binaphthalene (**Bn-42**) as an off-white solid (50.3 mg, 62%, 80:20 e.r.) and recovered starting material (**42**) (22.2 mg, 33%, 99:1 e.r.).

(S)- 2-(Benzyloxy)-2'-methoxy-4,4'-diphenyl-1,1'-binaphthalene (Bn-42):

m.p. = 117-119 °C (CH_2Cl_2/petroleum ether 40-60).

IR (film) ν_{max}/cm^{-1}: 3060, 3029, 2935, 1586, 1342, 1224, 764, 703.

^{1}H NMR (500 MHz, $CDCl_3$) δ = 7.94 (m, 2H), 7.71–7.65 (m, 2H), 7.63–7.47 (m, 8H), 7.44 (m, 2H), 7.39 (m, 1H), 7.35–7.22 (m, 5H), 7.17 (m, 2H), 7.00 (m, 3H), 5.42–4.89 (m, 2H), 3.81 (s, 3H).

^{13}C NMR (126 MHz, $CDCl_3$) δ = 154.4, 153.5, 141.9, 141.6, 141.1, 140.8, 137.6, 134.6, 134.6, 130.2, 128.4, 128.3, 128.1, 127.9, 127.5, 127.5, 127.4, 127.4, 126.9, 126.3, 126.3, 126.2, 126.2, 125.9, 125.9, 123.9, 123.6, 120.5, 118.9, 117.6, 115.0, 71.5, 56.7.

HRMS (ESI^+) $C_{40}H_{30}O_2$ $[M+H]^+$ requires 543.23186; found 543.23132, Δ −0.1 ppm.

Chiral HPLC: (Chiralpak ODH, 2% ^{i}PrOH, 98% hexane, 1.0 mL min^{-1}, λ = 295 nm) τ_R (minor) = 9.0 min, τ_R (major) = 10.7 min.

$[\alpha]_D^{25}$ = −31.1(c = 1.00, $CHCl_3$).

(R)- [2'-methoxy-4,4'-diphenyl-[1,1'-binaphthalen]-2-ol] (42):

m.p. = 125-127 °C (CH_2Cl_2/petroleum ether 40-60).

Chiral HPLC: (Chiralpak ADH, 20% ^{i}PrOH, 80% hexane, 1.0 mL min^{-1}, λ = 300 nm) τ_R (major) = 20.8 min, τ_R (minor) = 36.0 min..

$[\alpha]_D^{25}$ = +16.9 (c = 1.00, $CHCl_3$).

Kinetic resolution of 2'-methoxy-4,4'-dimethyl-[1,1'-binaphthalen]-2-ol (43)

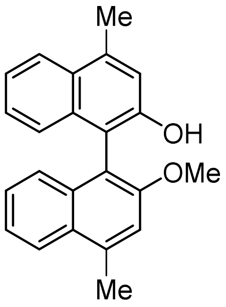

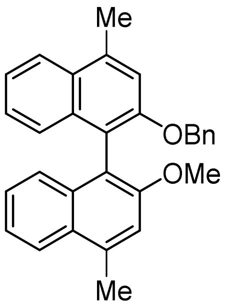

Following **General procedure A**, 2'-methoxy-4,4'-dimethyl-[1,1'-binaphthalen]-2-ol (**43**) (49.3 mg, 0.150 mmol) was reacted for 30 h. Purification *via* flash column chromatography (3:7 CH_2Cl_2 – petroleum ether 40-60 to 4:1 CH_2Cl_2 – petroleum ether 40-60) afforded 2-(benzyloxy)-2'-methoxy-4,4'-dimethyl-1,1'-binaphthalene (**Bn-43**) as an off-white solid (33.8 mg, 54%, 88:12 e.r.) and recovered starting material (**43**) (19.4 mg, 39%, 99:1 e.r.).

(S)- 2-(Benzyloxy)-2'-methoxy-4,4'-dimethyl-1,1'-binaphthalene (Bn-43):

m.p. = 155-157 °C (CH_2Cl_2/petroleum ether 40-60).

IR (film) ν_{max}/cm^{-1}: 3063, 2937, 2911, 1593, 1342, 757.

^{1}H NMR (500 MHz, $CDCl_3$) δ = 8.06 (d, *J* = 8.5, 1H), 8.03 (d, *J* = 8.5, 1H), 7.42–7.36 (m, 2H), 7.35 (s, 1H), 7.33 (s, 1H), 7.28–7.21 (m, 4H), 7.22–7.15 (m, 3H), 7.06–6.98 (m, 2H), 5.09 (d, *J* = 12.5 Hz, 1H), 5.05 (d, *J* = 12.6 Hz, 1H), 3.78 (s, 3H), 2.88 (s, 3H), 2.81 (s, 3H).

^{13}C NMR (126 MHz, $CDCl_3$) δ = 154.5, 153.7, 137.8, 135.9, 135.8, 134.5, 128.8, 128.4, 128.1, 127.2, 126.8, 126.2, 126.2, 126.0, 126.0, 124.2, 124.1, 123.6, 123.3, 119.2, 117.7, 117.5, 115.1, 71.3, 56.7, 20.0, 20.0.

HRMS (ESI$^+$) $C_{30}H_{26}O$ $[M+Na]^+$ requires 441.18250; found 441.18280, Δ +0.7 ppm.

Chiral HPLC: (Chiralpak ADH, 3% ^{i}PrOH, 99% hexane, 1.0 mL min^{-1}, λ = 250 nm) τ_R (major) = 19.7 min, τ_R (minor) = 12.0 min.

$[\alpha]_D^{25}$ = −36.0 (*c* = 1.00, $CHCl_3$).

(R)- 2'-Methoxy-4,4'-dimethyl-[1,1'-binaphthalen]-2-ol (43):

m.p. = 201-202 °C (CH_2Cl_2/petroleum ether 40-60).

Chiral HPLC: (Chiralpak ADH, 10% ^{i}PrOH, 90% hexane, 1.0 mL min^{-1}, λ = 293 nm) τ_R (major) = 20.9 min, τ_R (minor) = 23.3 min.

$[\alpha]_D^{25}$ = −31.7 (*c* = 1.00, $CHCl_3$).

7. 10 g Scale Kinetic Resolution

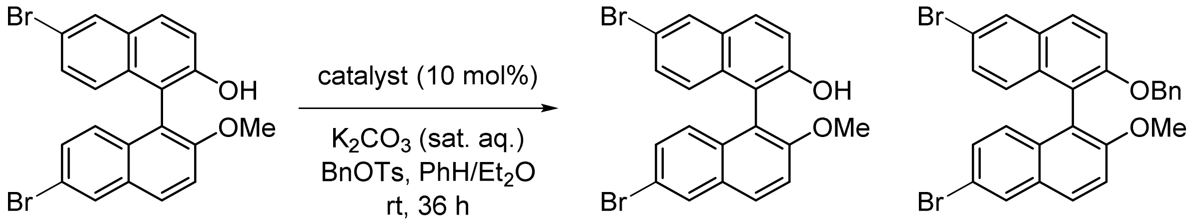

6,6'-Dibromo-2'-methoxy-[1,1'-binaphthalen]-2-ol (**33**) (10.1 g, 22.0 mmol, 1.00 equiv.), benzyl tosylate (8.66 g, 33.0 mmol, 1.50 equiv.) and *N*-(2,3,4-trifluorobenzyl)hydrocinchoninium bromide (1.15 g, 2.20 mmol, 1.00 equiv.) were added to a 2 L 3 necked round bottom flask fitted with an overhead stirrer (pictured below). 9:1 PhH-Et_2O (880 mL) was added followed by K_2CO_3 (saturated aq., 30.4 mL) and stirring was commenced (1100 RPM). After 36 hours piperidine (44.0 mL) was added and stirring was continued for a further 30 minutes. NH_4Cl (saturated aq., 300 mL) was added and the mixture was transferred to a separating funnel. The phases were separated and the aqueous phase was extracted with EtOAc (2 × 300 mL). The combined organic layers were washed with water (200 mL), dried over anhydrous sodium sulfate, concentrated *in vacuo* and purified by flash column chromatography (3:7 CH_2Cl_2 – petroleum ether 40-60 to 3:2 CH_2Cl_2 – petroleum ether 40-60) to give 2-(benzyloxy)-6,6'-dibromo-2'-methoxy-1,1'-binaphthalene (**Bn-33**) (6.32 g, 52%, 90:10 e.r.) and recovered starting material (**33**) (4.32 g, 43%, 99:1 e.r.).

2-(Benzyloxy)-6,6'-dibromo-2'-methoxy-1,1'-binaphthalene (Bn-33):

m.p. = 54-55 °C (CH_2Cl_2/petroleum ether 40-60).

$[\alpha]_D^{25}$ = −9.8 (*c* = 1.00, $CHCl_3$)

6,6'-Dibromo-2'-methoxy-[1,1'-binaphthalen]-2-ol (33):

m.p. = 71-73 °C (CH_2Cl_2/petroleum ether 40-60).

$[\alpha]_D^{25}$ = −106.0 (*c* = 1.00, $CHCl_3$)

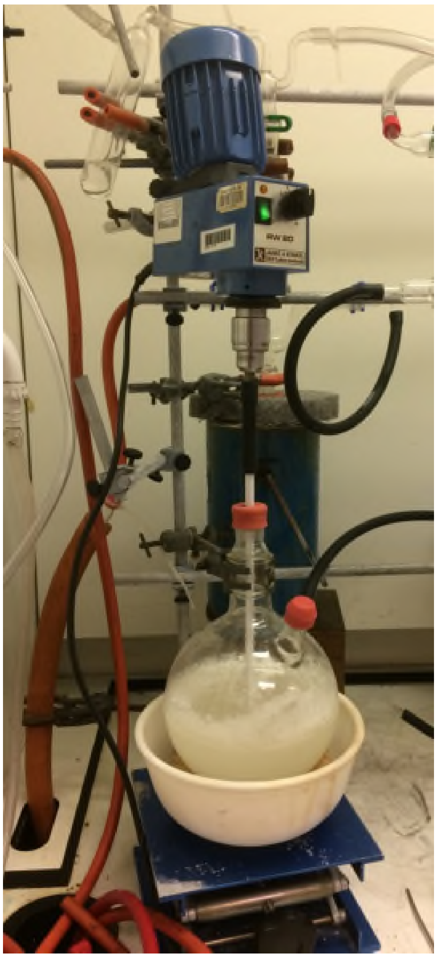

8. Determination of Absolute Stereochemistry

The absolute stereochemistry of MeBINOL (**1**) and MeBnBINOL (**Bn-1**) were determined by chiral HPLC comparison with authentic samples prepared from commercially available (R)-BINOL and (S)-BINOL respectively. The optical rotation of MeBINOL (**1**) is consistent with the value reported in the literature.[17]

Chiral HPLC: (Chiralpak ADH, 7.5% ^{i}PrOH, 99% hexane, 1.00 mL min^{-1}, λ = 290nm) τ_R (major) = 25.7 min, τ_R (minor) = 16.6 min

(R)-MeBINOL prepared by kinetic resolution

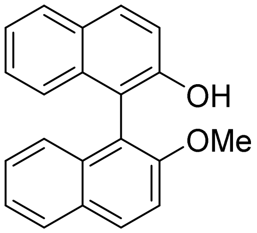

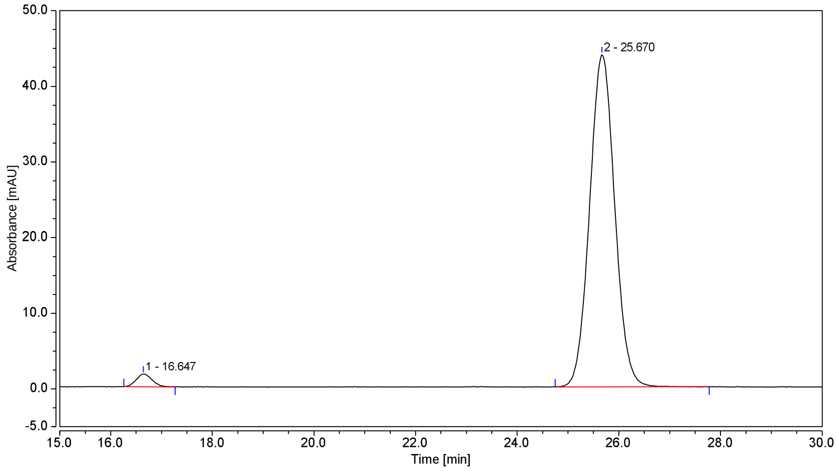

No.	Peak Name	Retention Time	Area	Height	Relative Area
		min	mAU*min	mAU	%
1		16.647	0.622	1.677	2.41
2		25.670	25.162	43.915	97.59
**Total:**			**25.784**	**45.591**	**100.00**

(R)-MeBINOL prepared from commercially available (R)-BINOL

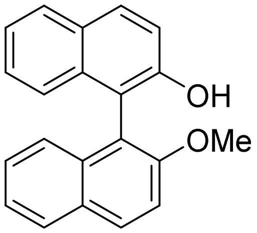

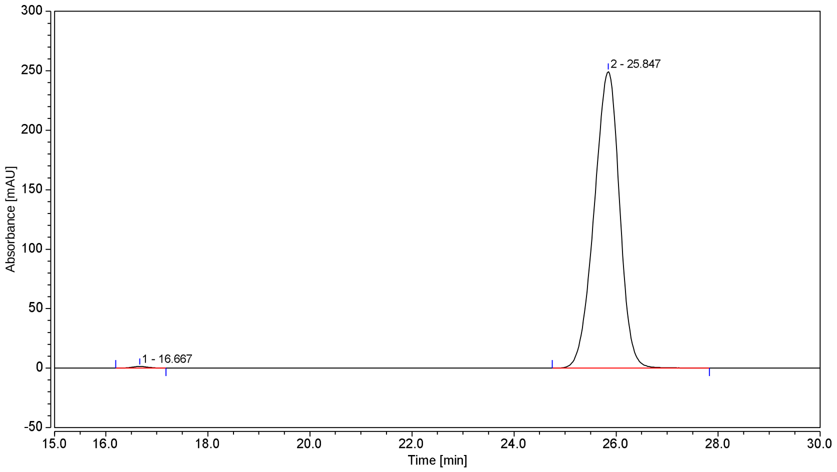

No.	Peak Name	Retention Time	Area	Height	Relative Area
		min	mAU*min	mAU	%
1		16.667	0.518	1.402	0.36
2		25.847	143.619	249.436	99.64
**Total:**			**144.137**	**250.838**	**100.00**

Chiral HPLC: (Chiralpak IC, 1% ^{i}PrOH, 99% hexane, 1.00 mL min^{-1}, λ = 290 nm) τ_R (major) = 7.6 min, τ_R (minor) = 11.0 min

(S)-MeBnBINOL prepared by kinetic resolution

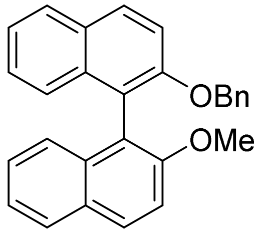

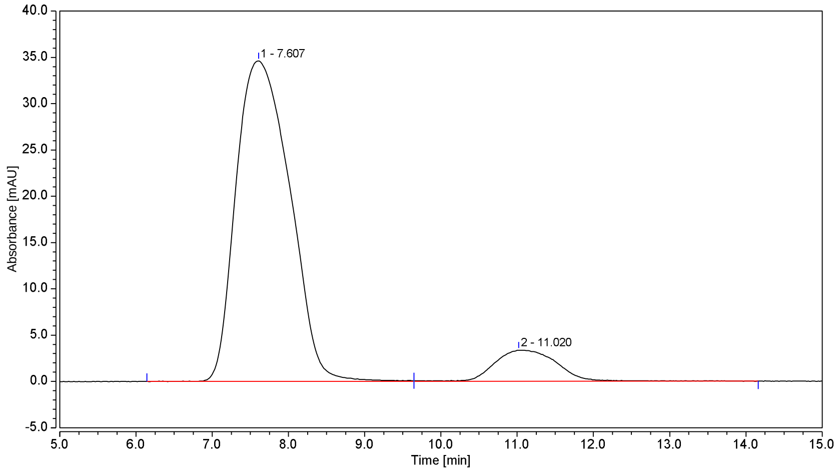

No.	Peak Name	Retention Time	Area	Height	Relative Area
		min	mAU*min	mAU	%
1		7.607	28.297	34.615	89.96
2		11.020	3.156	3.356	10.04
**Total:**			**31.454**	**37.971**	**100.00**

(S)-MeBnBINOL prepared from commercially available (S)-BINOL

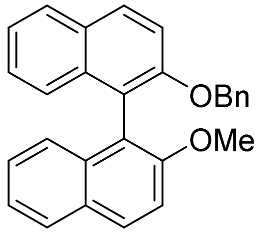

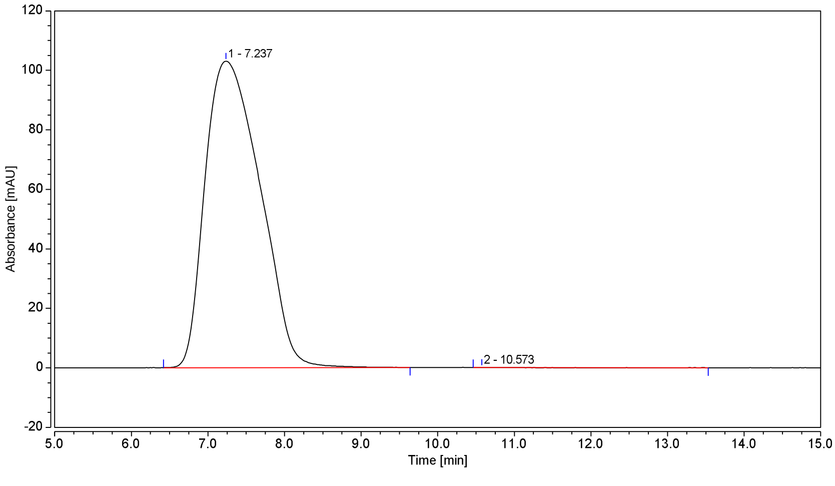

No.	Peak Name	Retention Time	Area	Height	Relative Area
		min	mAU*min	mAU	%
1		7.237	85.670	103.034	99.89
2		10.573	0.098	0.026	0.11
**Total:**			**85.767**	**103.060**	**100.00**

8. References

[1] Pangborn, M.; Giardello, R.; Grubbs, R.; Rosen, R.; Timmers, J. *Organometallics,* **1996**, 15, 1518.

[2] Armarego, W. L. F.; Perrin, D. D.; *Purification of Laboratory Chemicals*, **1996**, Butterworth-Heinemann, Oxford.

[3] Noji, M.; Nakajima, M.; Koga, K. *Tetrahedron Letters,* **1994**, *34*, 7983.

[4] Lustenberger, P.; Diederich, F. *Helvetica Chimica Acta,* **2000**, *83*, 2865.

[5] Aoyagi, N.; Ogawa, N.; Izumi, T. *Tetrahedron Lett*, **2006**, *28*, 4797.

[6] Yu, H.-B.; Zheng, X.-F.; Lin Z.-M.; Hu, Q.-S.; Huang, W.-S.; Pu, L. *J. Org. Chem.*, **1999**, *64*, 8149

[7] Hocke, H.; Uozumi, Y. *Tetrahedron*, **2003**, *5*, 619.

[8] Trivedi-Parmar, V.; Robertson, M. J.; Cisneros, J. A.; Krimmer, S. G.; Jorgensen, W. L. *ChemMedChem*, **2018**, *11*, 1092

[9] Ning, Y.; Fukuda, T.; Ikeda, H.; Otani, Y.; Kawahata, M.; Yamaguchi, K.; Ohwada, T. *Org. Biomol. Chem.* **2017**, *6*, 1381

[10] Chow, H.-F.; Ng, M.-K. *Tetrahedron Asymmetry* **1996**, *8*, 2251

[11] Jolliffe J. D., Armstrong R. J., Smith M. D. *Nature Chem.* **2017**, *9*, 558.

[12] Tao, T., Sun, Z., Fang, W., Xu, M., Zhou, Y. *Org. Lett.* **2012**, *16*, 4250.

[13] Takahashi, M., Kunio O. *Tetrahedron Asymmetry*, **1997**, 8, 3125.

[14] Meesala, Y., Wu, H-L., Koteswararao, B., Kuo, T-S., Lee, W-Z., *Organometallics*, **2014**, *17*, 4385.

[15] Kagan H. B., Fiaud J. C., *Top. Stereochem.* **1988**, *18*, 249-330.

[16] Genov, M., Almorin, A., Espinet, P., *Chem. Eur. J.*, **2006**, *36*, 9346.

[17] Ishiwari, F., Fukasawa, K-I., Sato, T., Nakazono, K., Koyama, Y., Takata, T., *Chem. Eur. J.,* **2011**, *17*, 12067.

9. NMR Spectra and HPLC traces

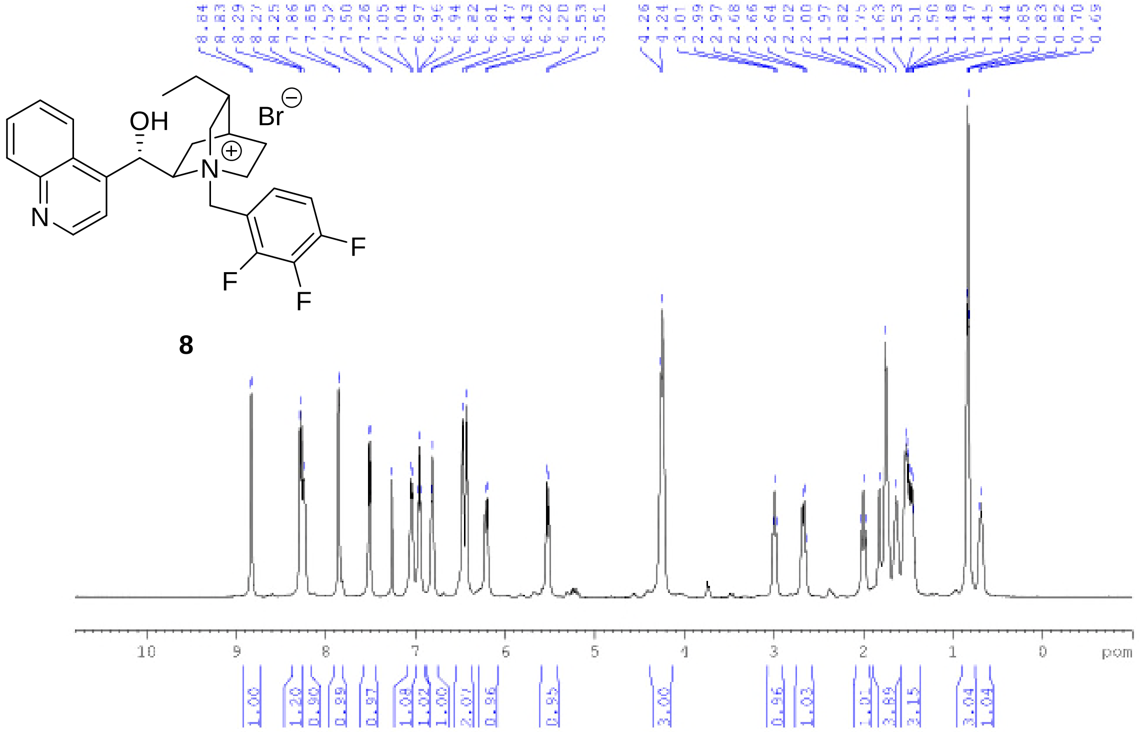

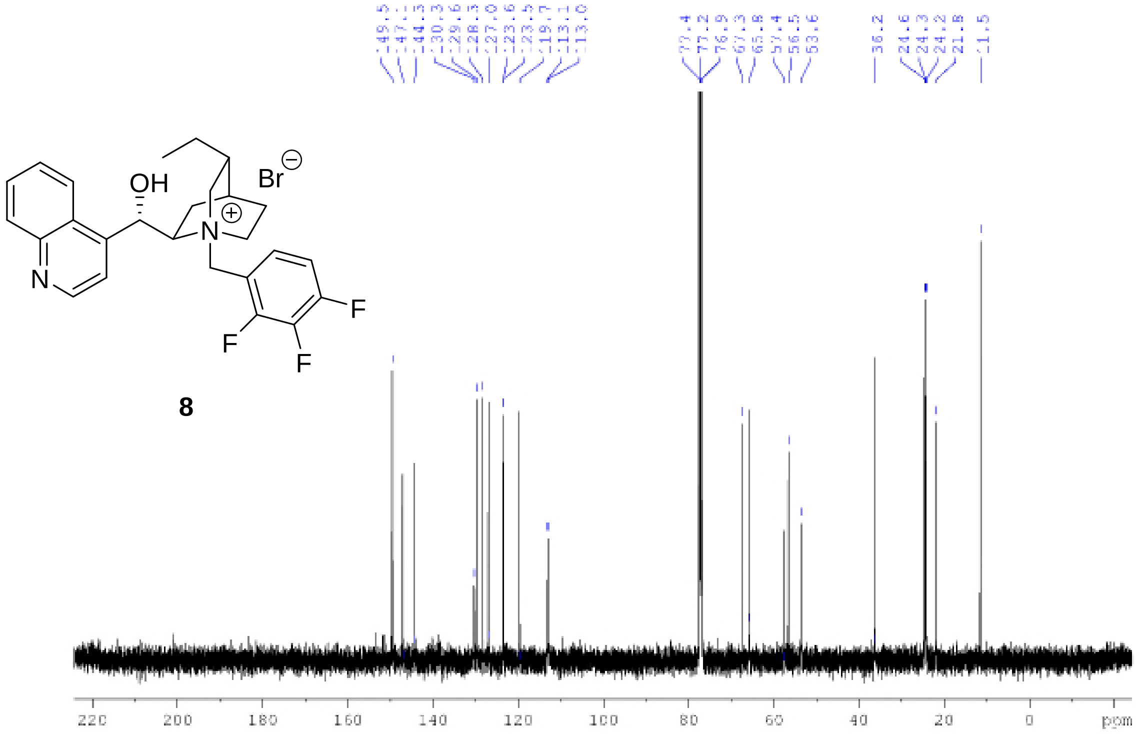

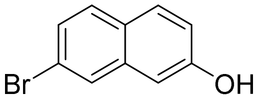

50

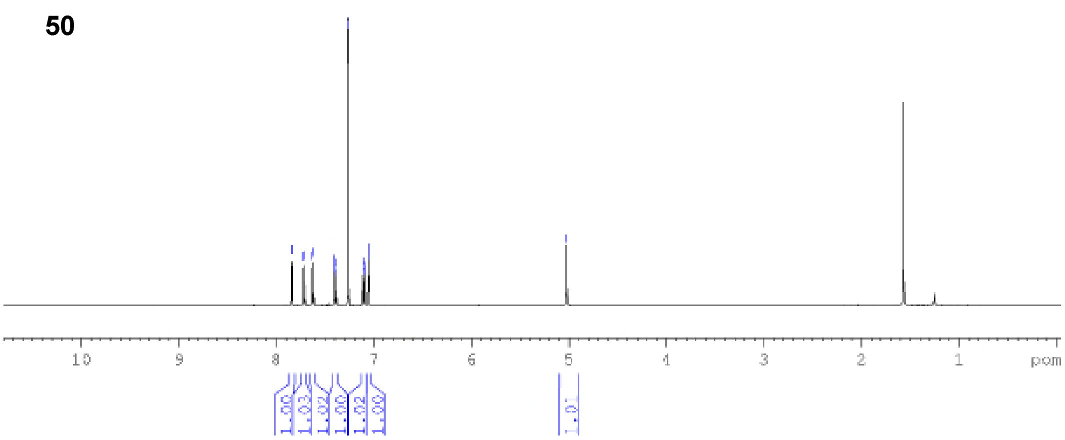

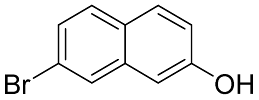

50

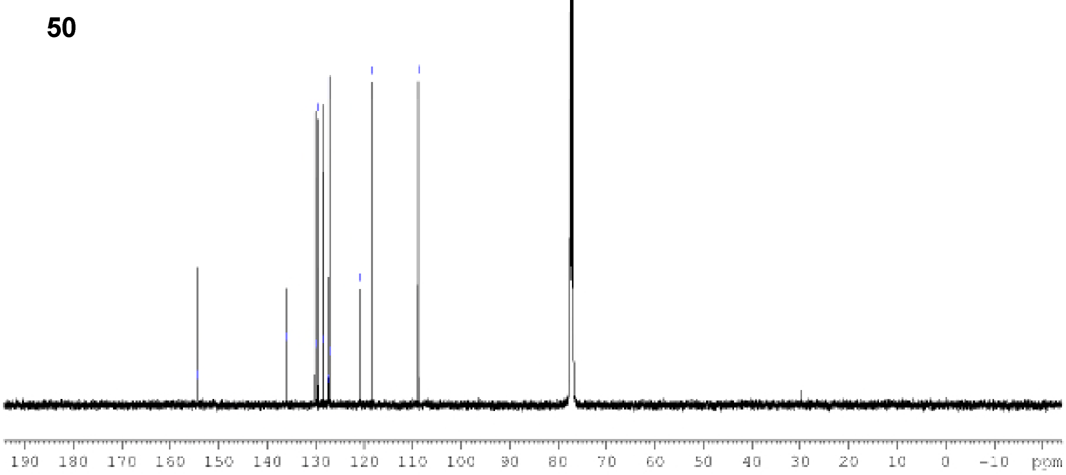

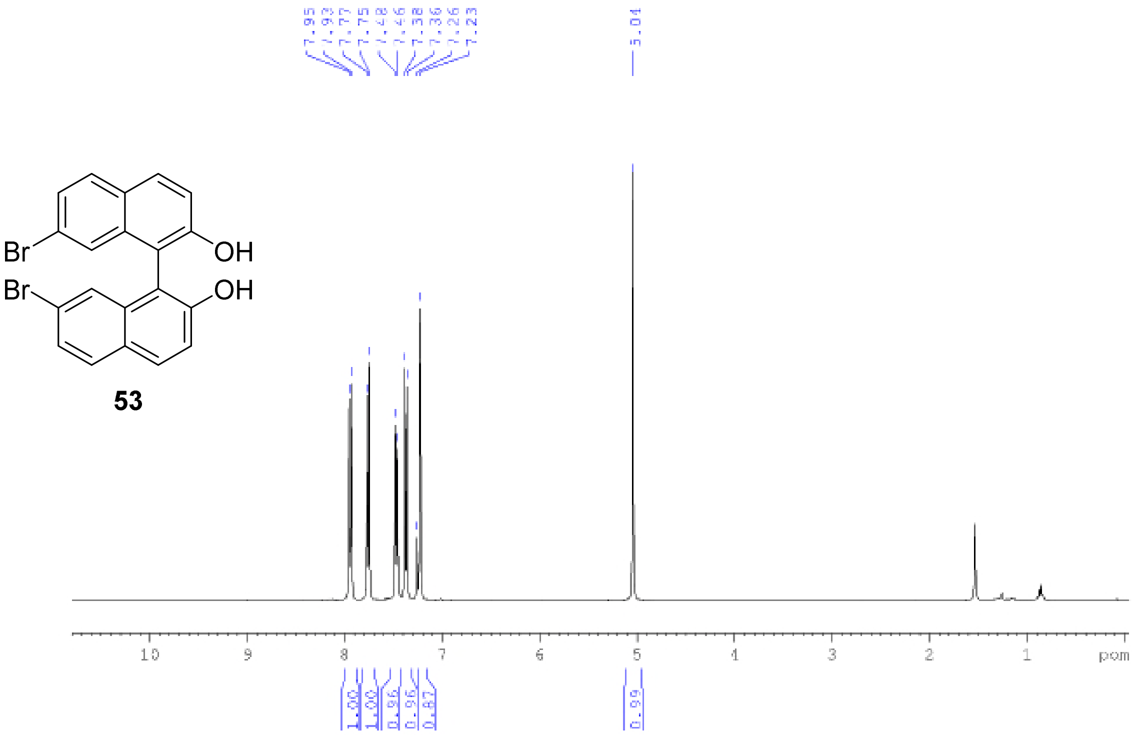
Br
OH
Br
OH
7.95
7.93
7.77
7.75
7.48
7.46
7.38
7.36
7.26
7.23
5.04
10
9
8
7
6
5
4
3
2
1
ppm
1.00
1.00
0.96
0.96
0.87
0.99

53

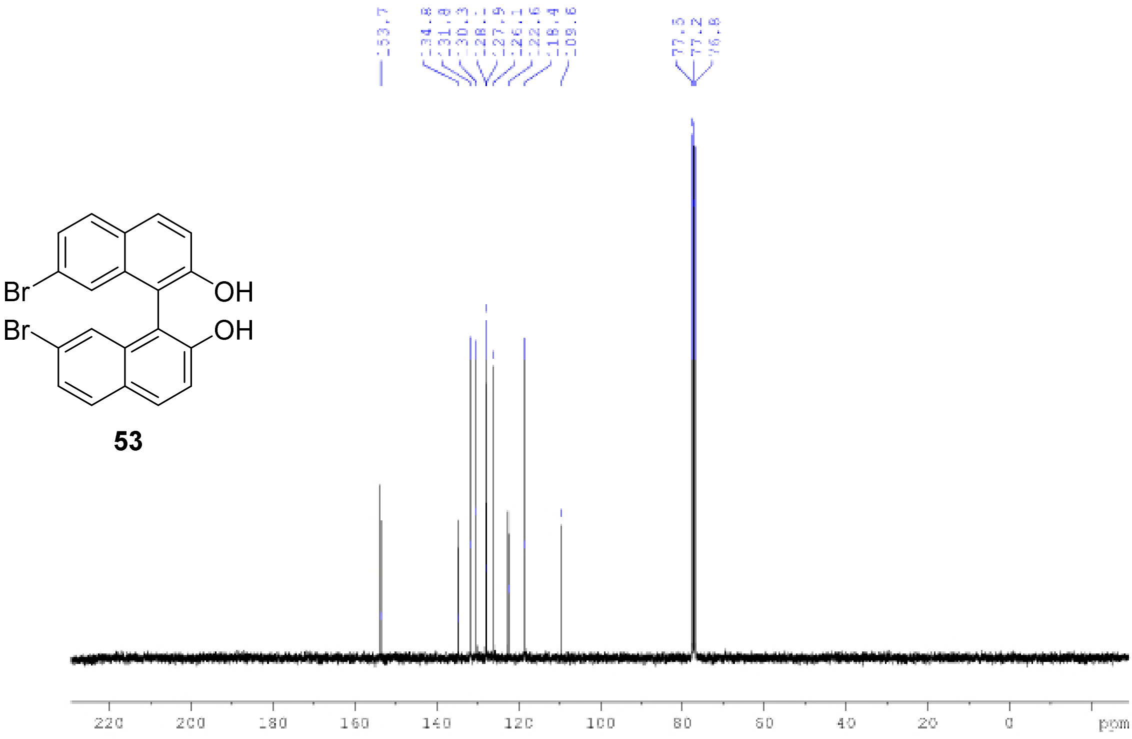
Br
OH
Br
OH
153.7
134.8
131.8
130.3
128.1
127.9
126.1
122.6
118.4
109.6
77.5
77.2
76.8
220
200
180
160
140
120
100
80
60
40
20
0
ppm

53

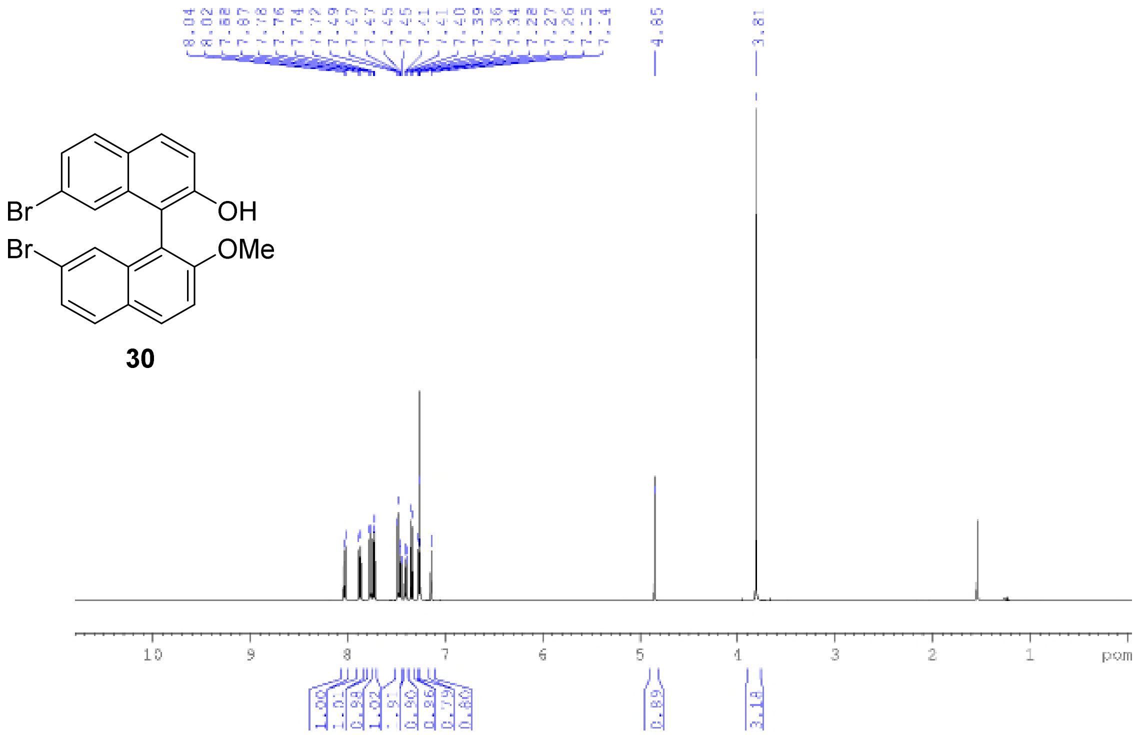

30

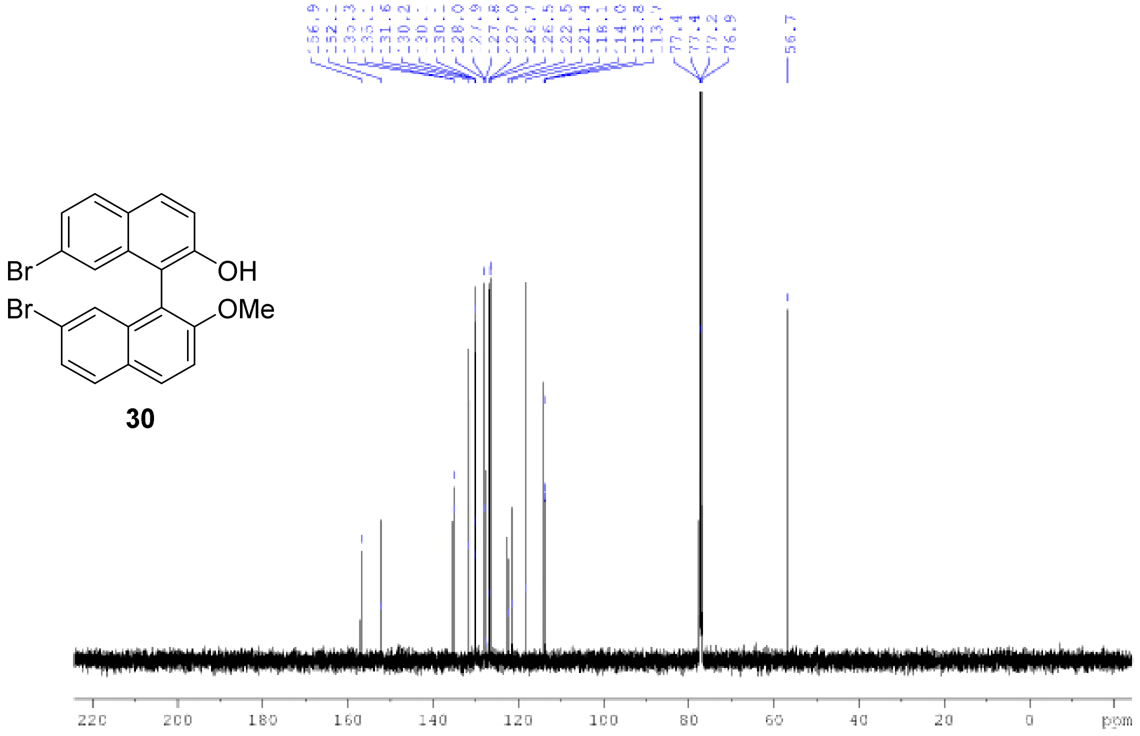

30

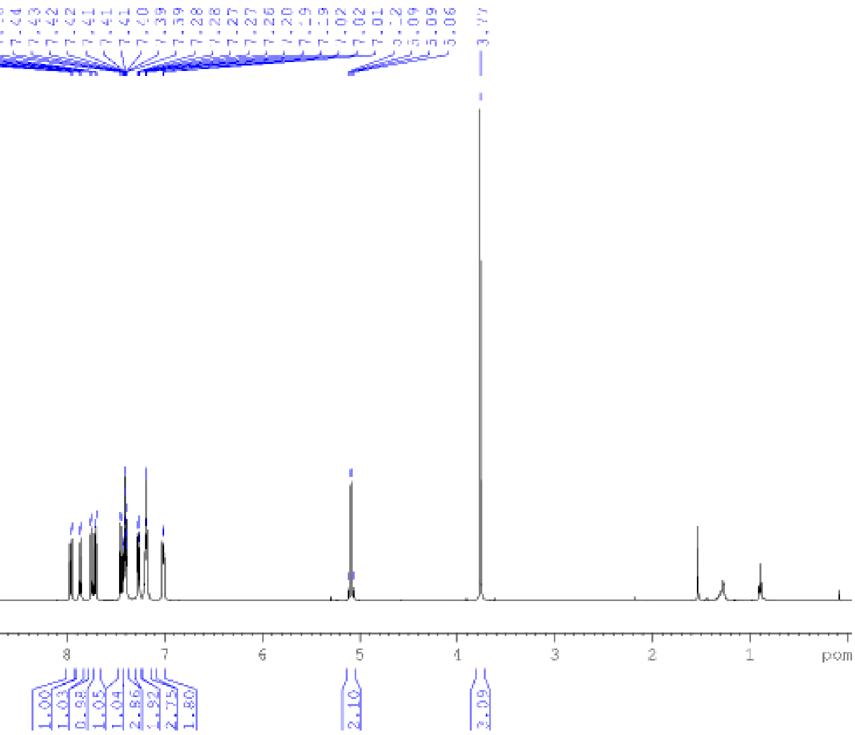

7.97
7.95
7.88
7.86
7.76
7.74
7.72
7.70
7.46
7.44
7.43
7.42
7.42
7.41
7.41
7.41
7.40
7.39
7.39
7.28
7.28
7.27
7.27
7.26
7.20
7.19
7.19
7.02
7.02
7.01
5.12
5.09
5.09
5.06
3.77
10
9
8
7
6
5
4
3
2
1
ppm
1.00
1.03
0.98
1.05
1.04
2.86
1.92
2.75
1.80
2.10
3.09

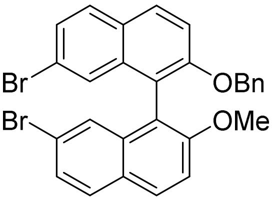

Br
OBn
Br
OMe

Bn-30

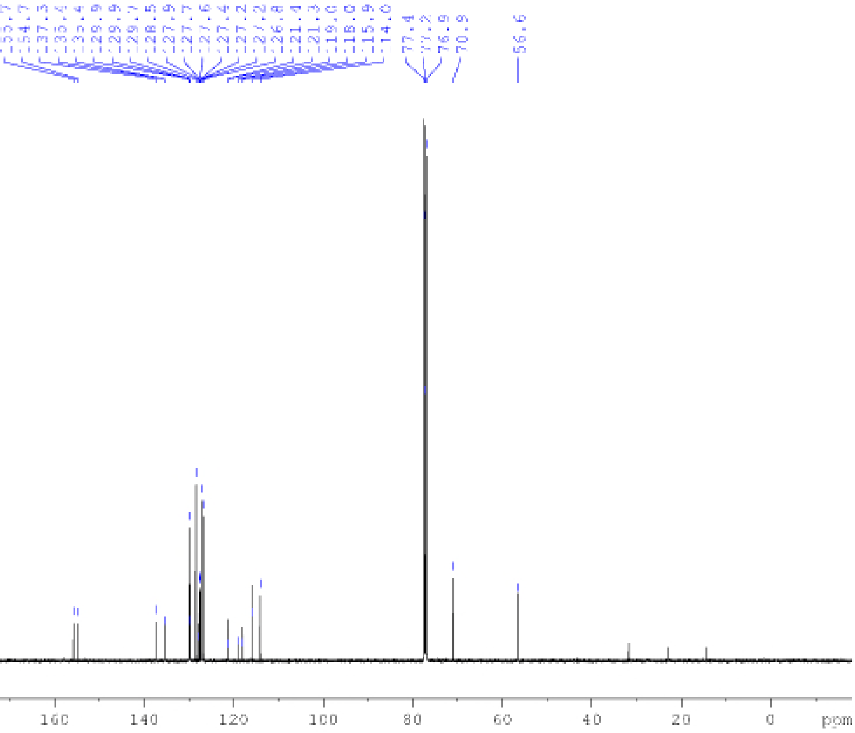

155.7
154.7
137.5
135.4
133.4
129.9
129.9
129.7
128.5
127.9
127.7
127.6
127.4
127.2
126.8
121.4
121.3
119.0
118.0
115.9
114.0
77.4
77.2
76.9
70.9
56.6
200
180
160
140
120
100
80
60
40
20
0
ppm

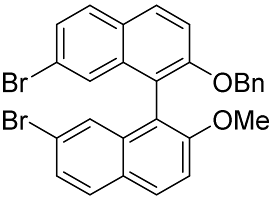

Br
OBn
Br
OMe

Bn-30

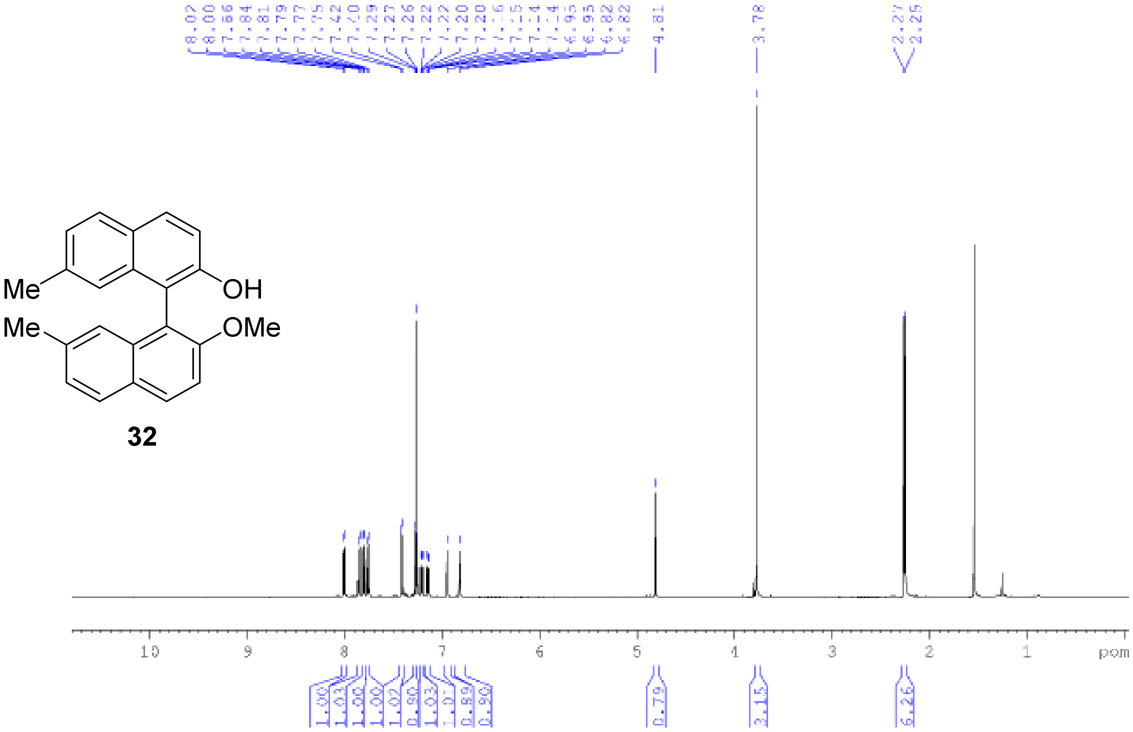
Me
OH
Me
OMe
32
8.02 8.00 7.86 7.84 7.81 7.79 7.77 7.75 7.42 7.40 7.29 7.27 7.26 7.22 7.22 7.20 7.20 7.16 7.15 7.14 7.14 6.95 6.95 6.82 6.82
4.81
3.78
2.27 2.25
10 9 8 7 6 5 4 3 2 1 ppm
1.00 1.03 1.00 1.00 1.02 0.90 1.03 1.07 0.89 0.90
0.79
3.15
6.26

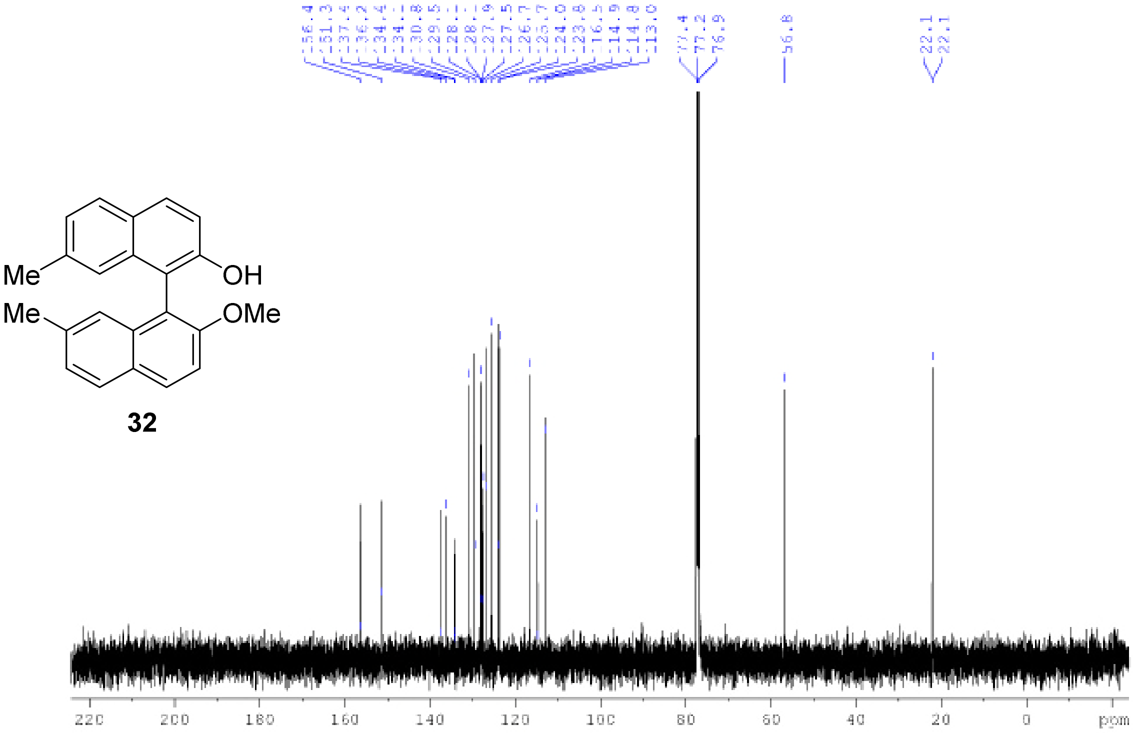
Me
OH
Me
OMe
32
156.4 151.3 137.4 136.2 134.4 134.1 130.8 129.5 128.1 128.1 127.9 127.5 126.7 125.7 124.0 123.8 116.5 114.9 114.8 113.0
77.4 77.2 76.9
56.8
22.1 22.1
220 200 180 160 140 120 100 80 60 40 20 0 ppm

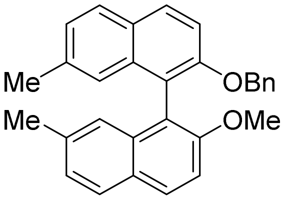
Bn-32

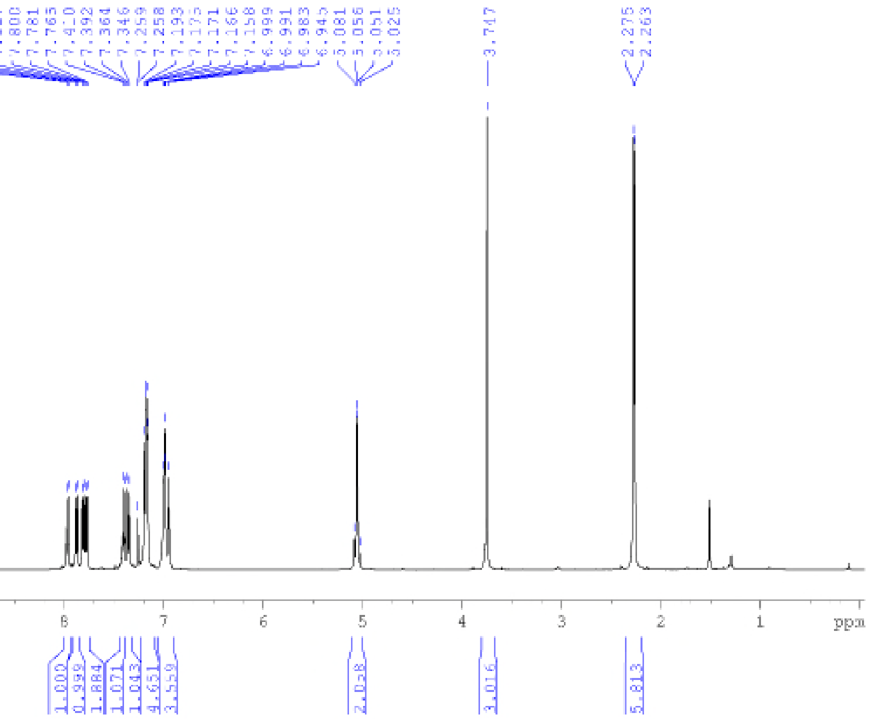

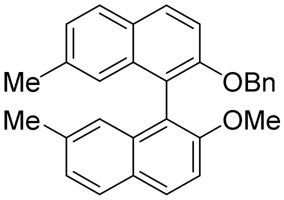
Bn-32

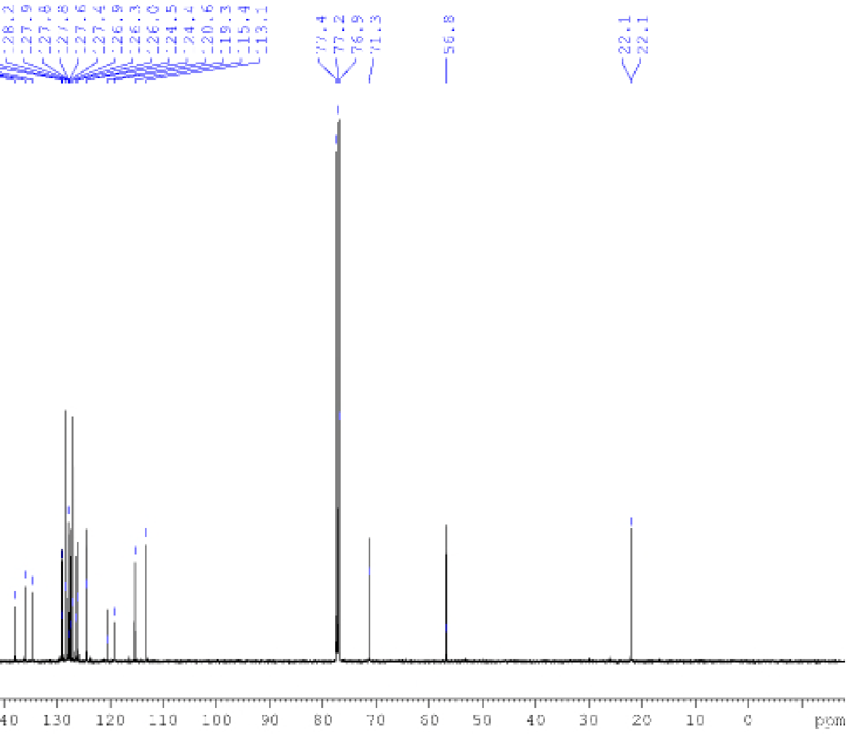

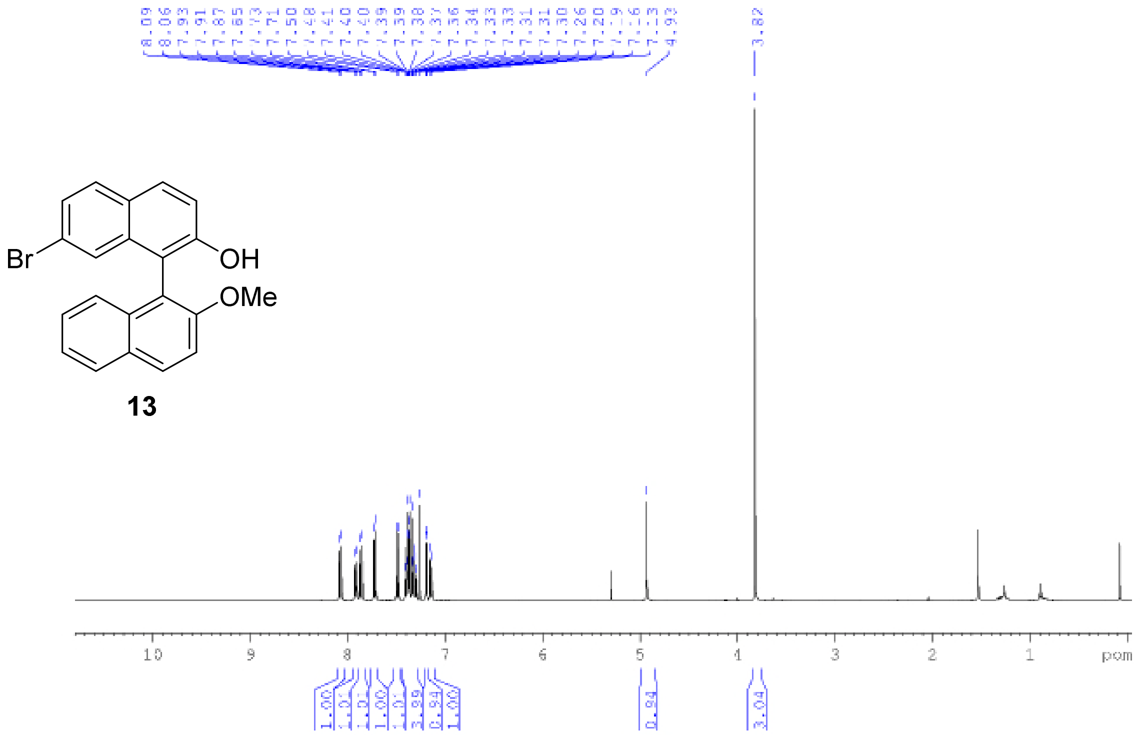

13

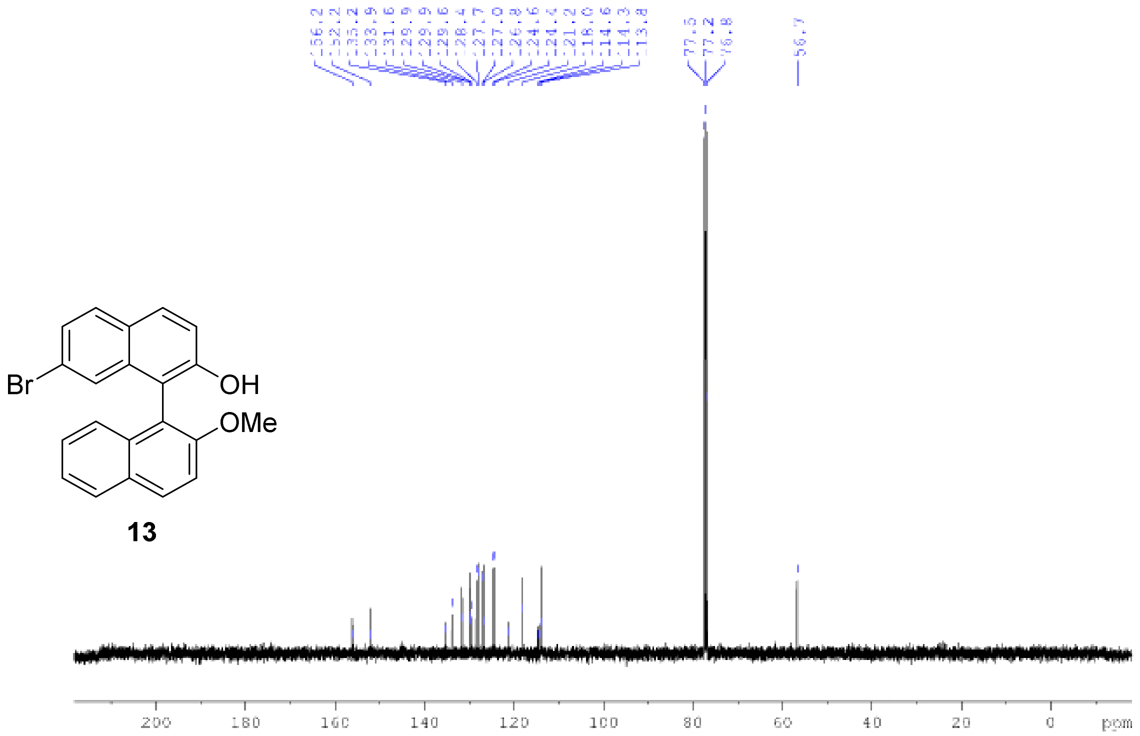

13

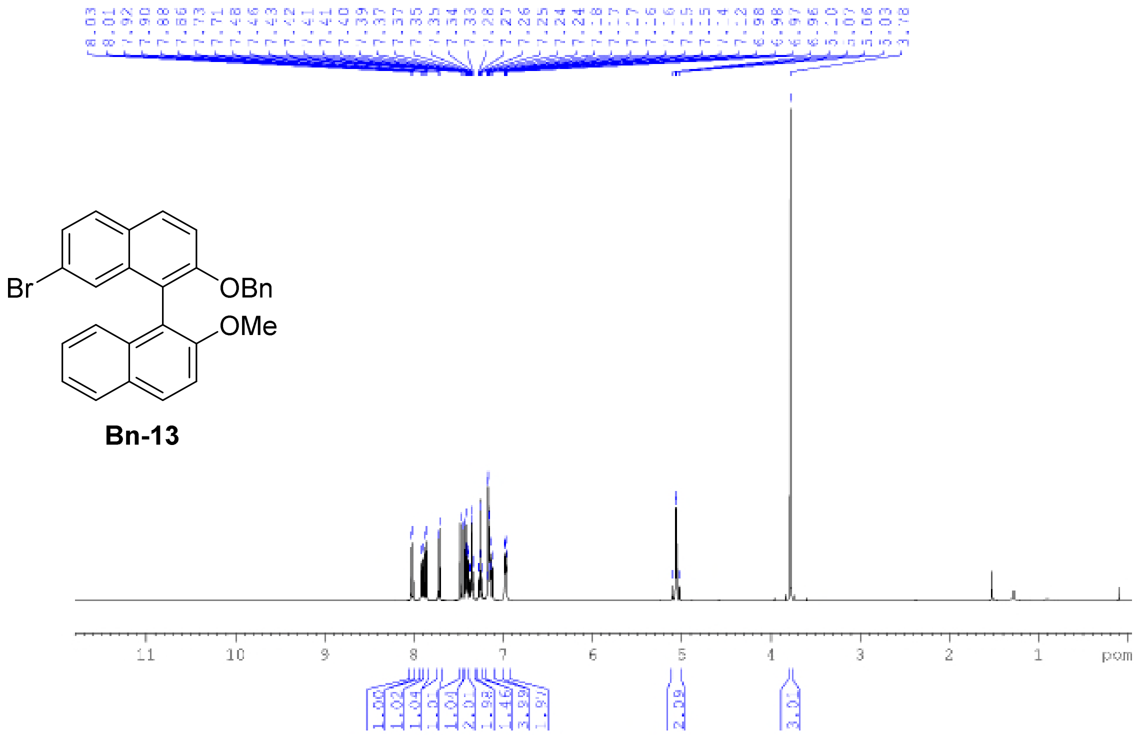

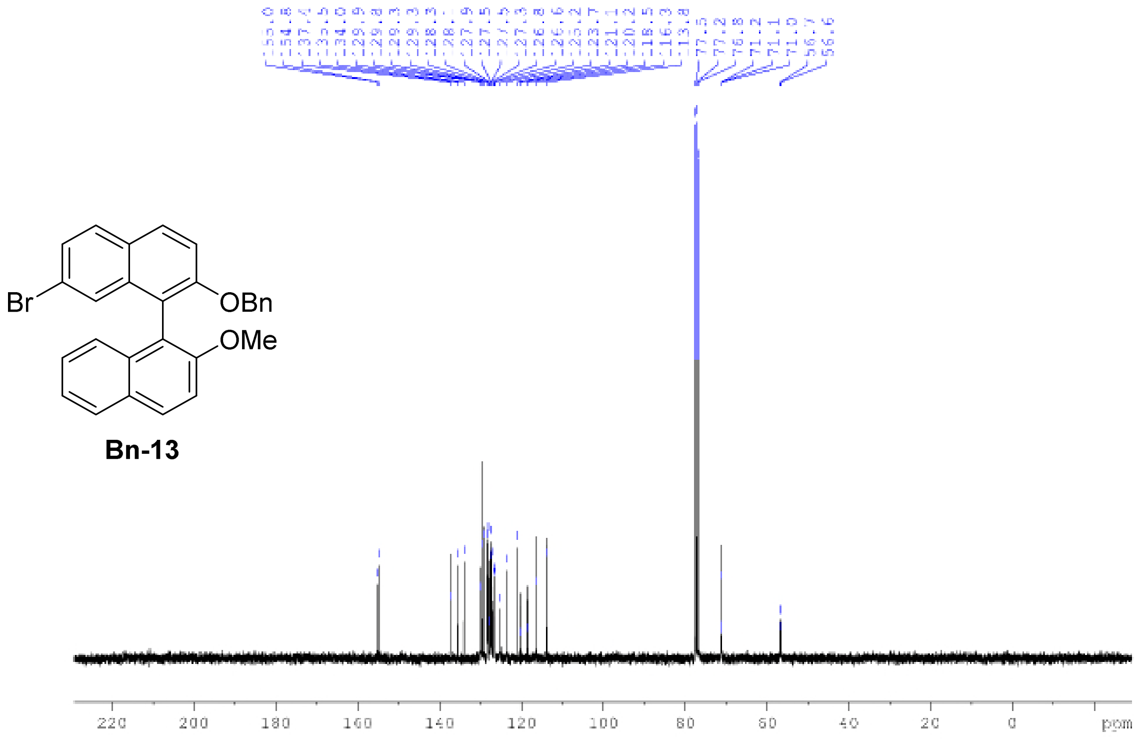

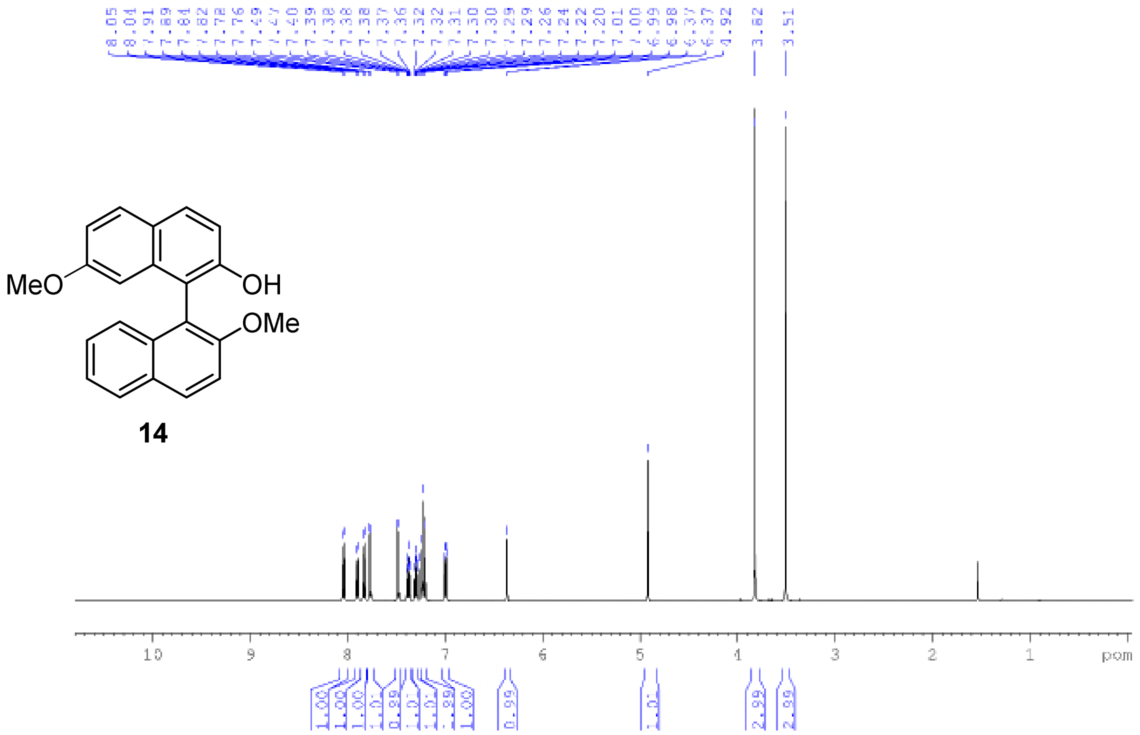
MeO
OH
OMe
14

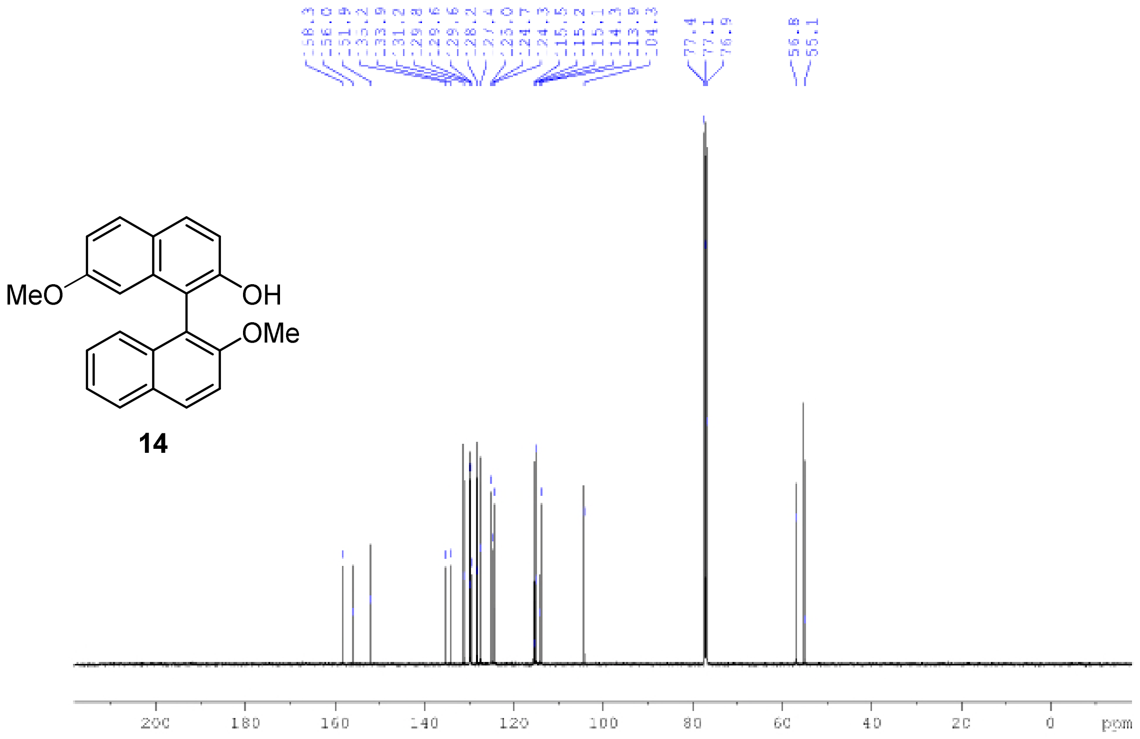
MeO
OH
OMe
14

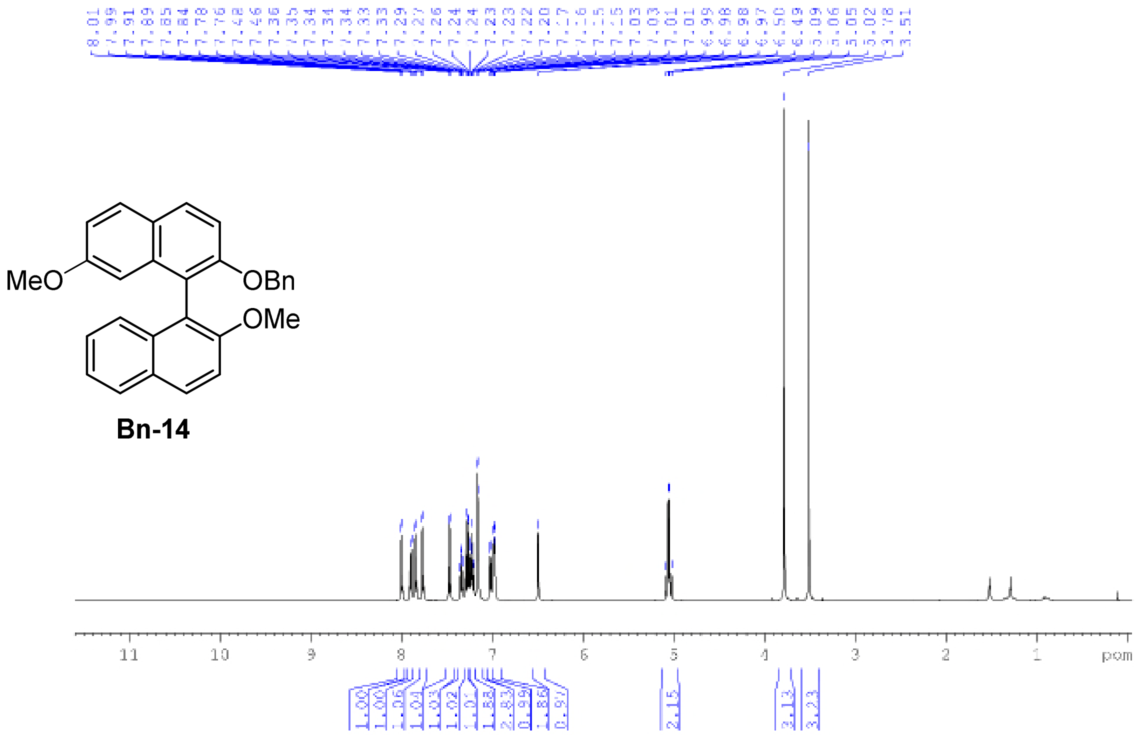

Bn-14

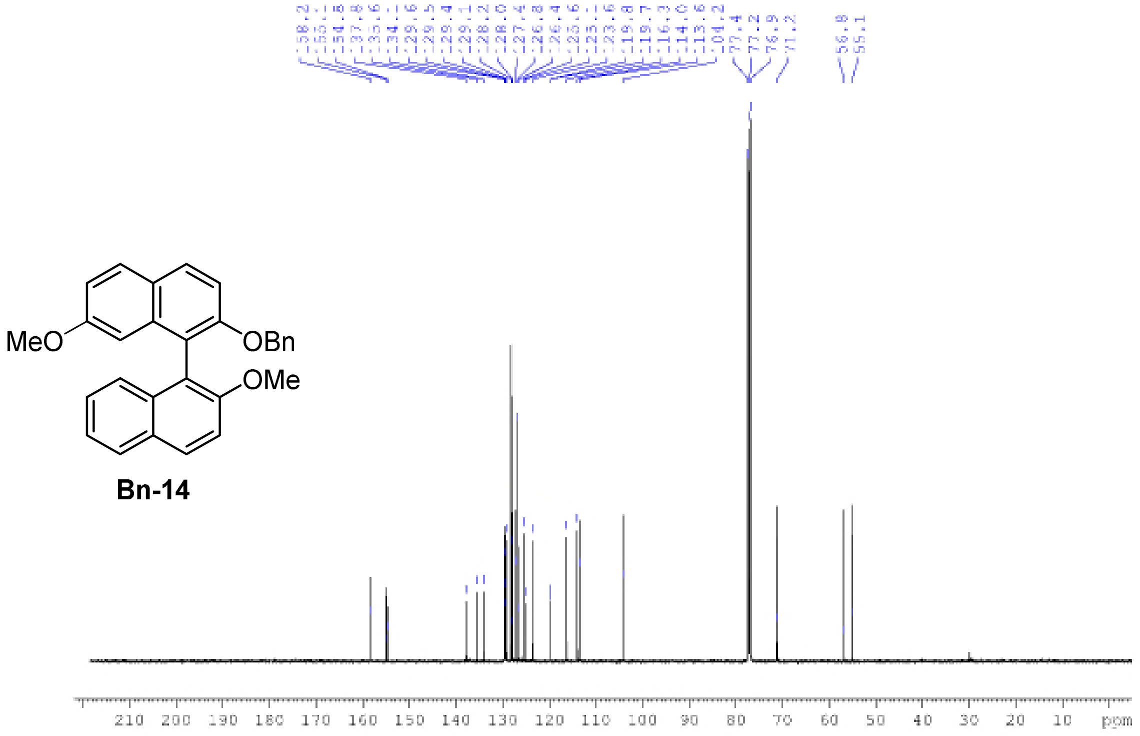

Bn-14

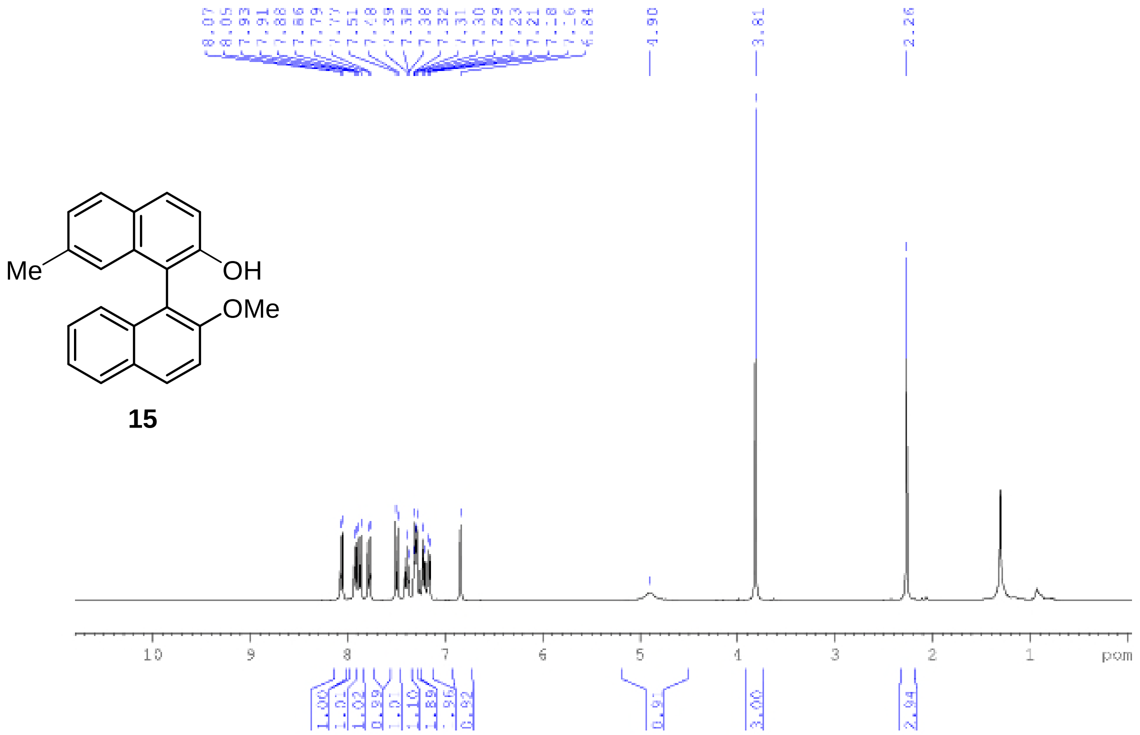

15

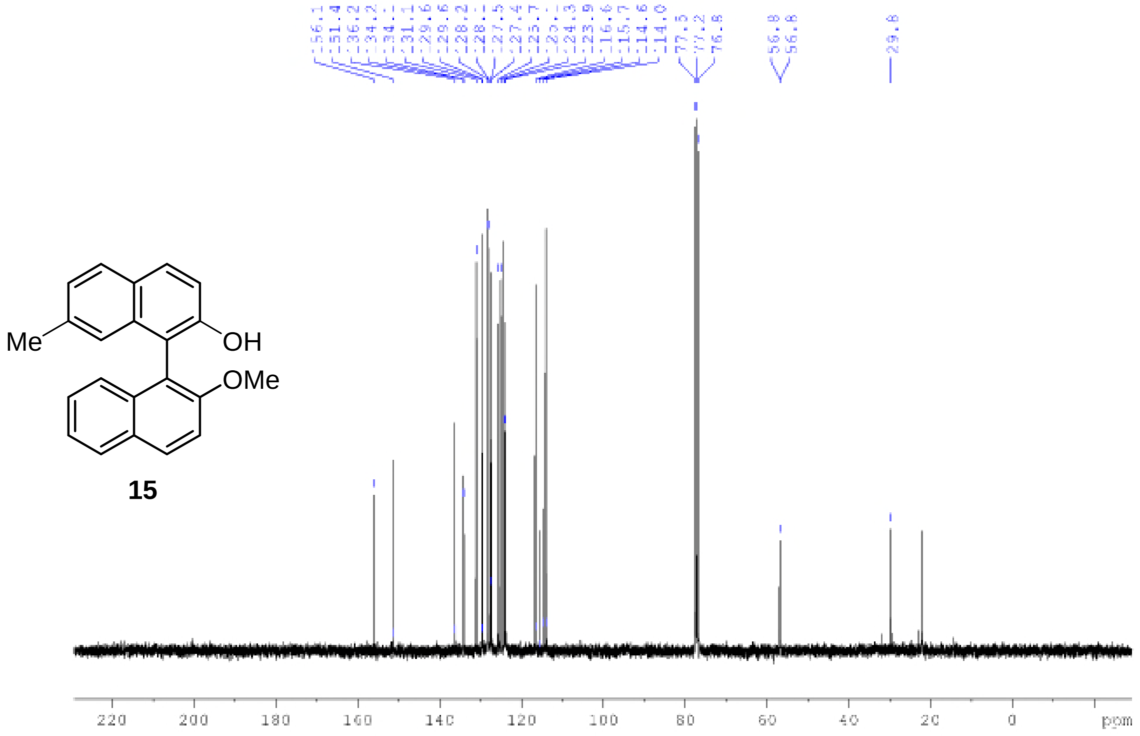

15

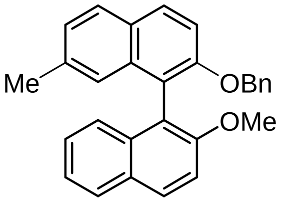
Me
OBn
OMe

Bn-15

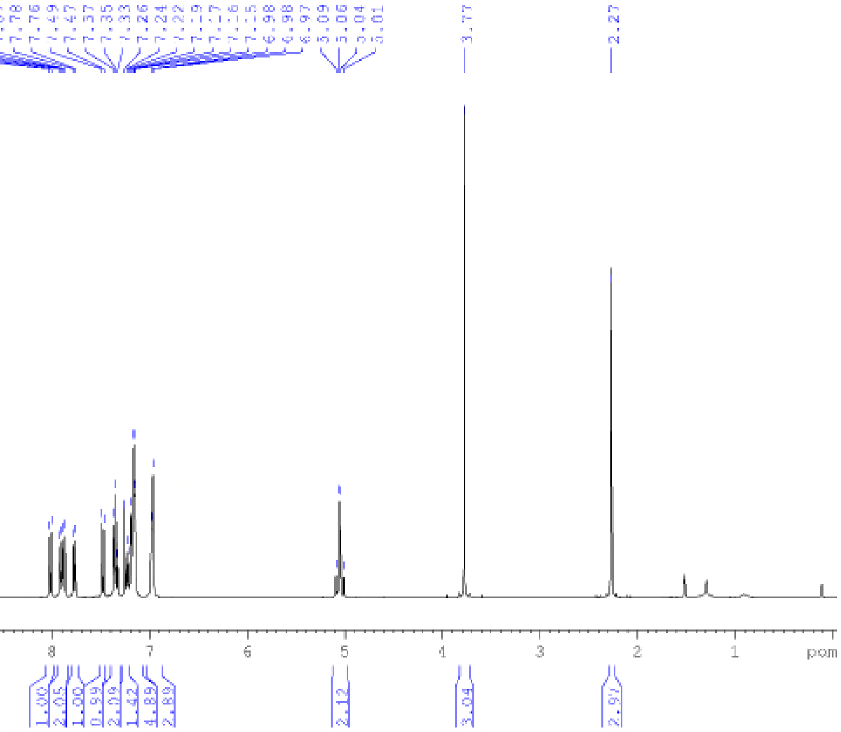
8.03
8.01
7.92
7.90
7.89
7.87
7.78
7.76
7.49
7.47
7.37
7.35
7.33
7.26
7.24
7.22
3.77
2.27
ppm
1.00
2.05
1.00
0.99
2.09
1.42
4.89
2.89
2.12
3.04
2.97

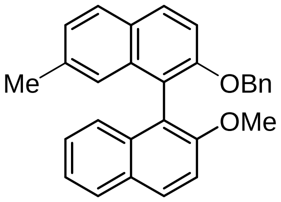
Me
OBn
OMe

Bn-15

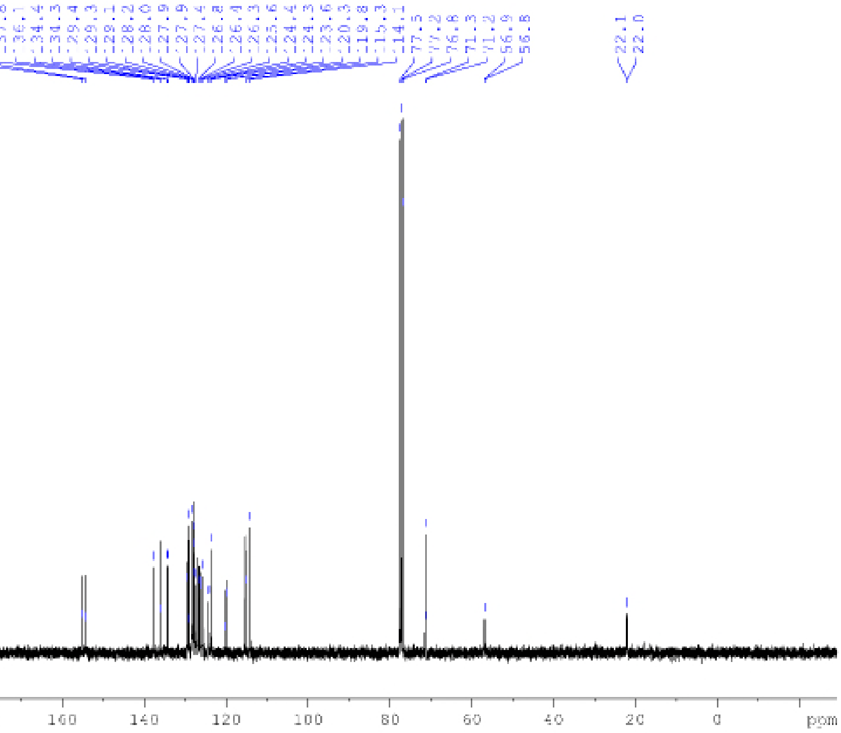
77.5
77.2
76.8
71.3
71.2
56.9
56.8
22.1
22.0
ppm

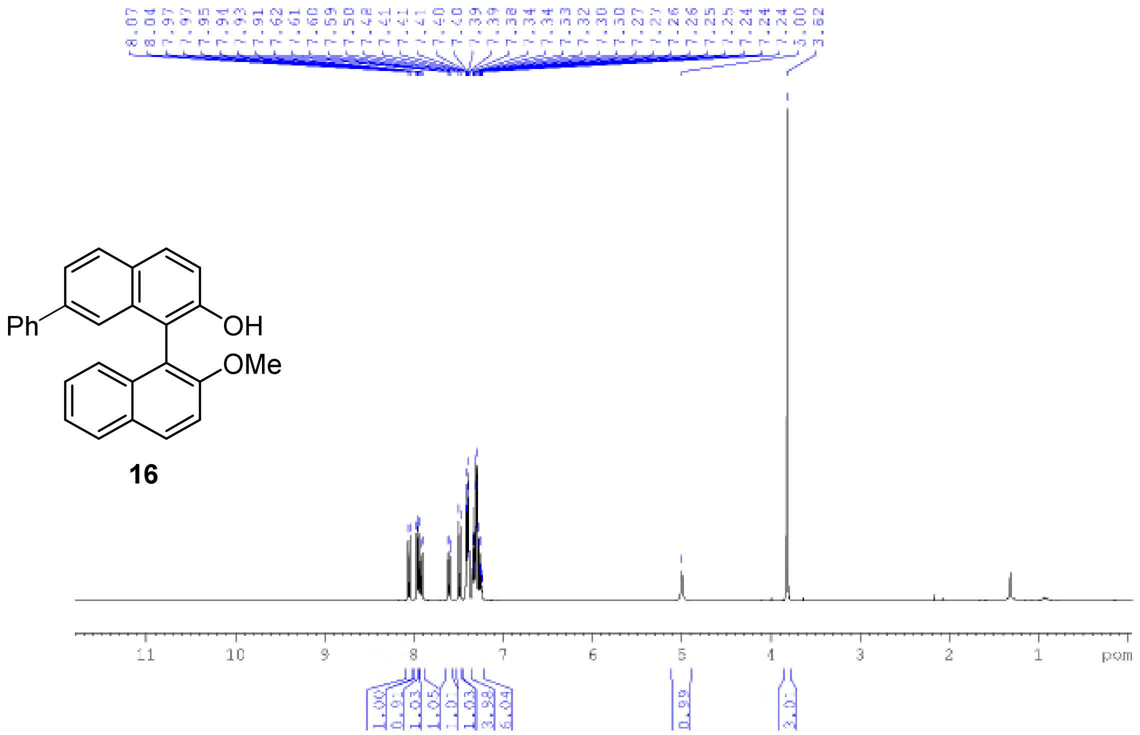
Ph
OH
OMe
16
ppm

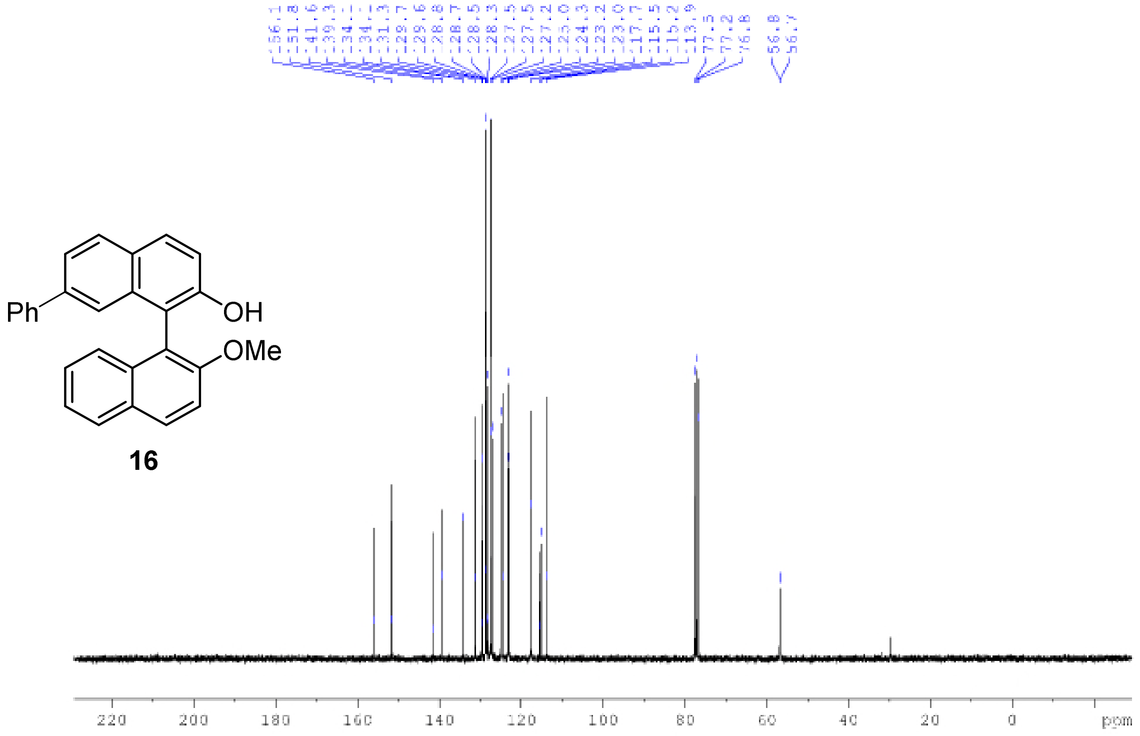
Ph
OH
OMe
16
ppm

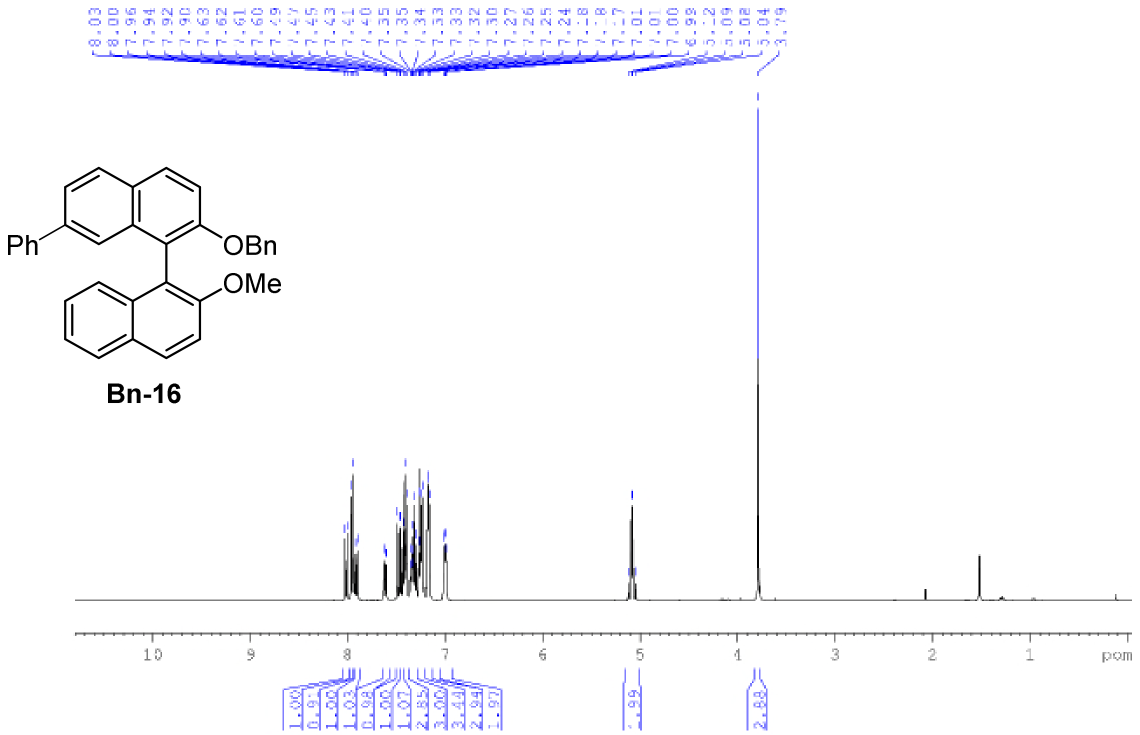
Ph
OBn
OMe
Bn-16

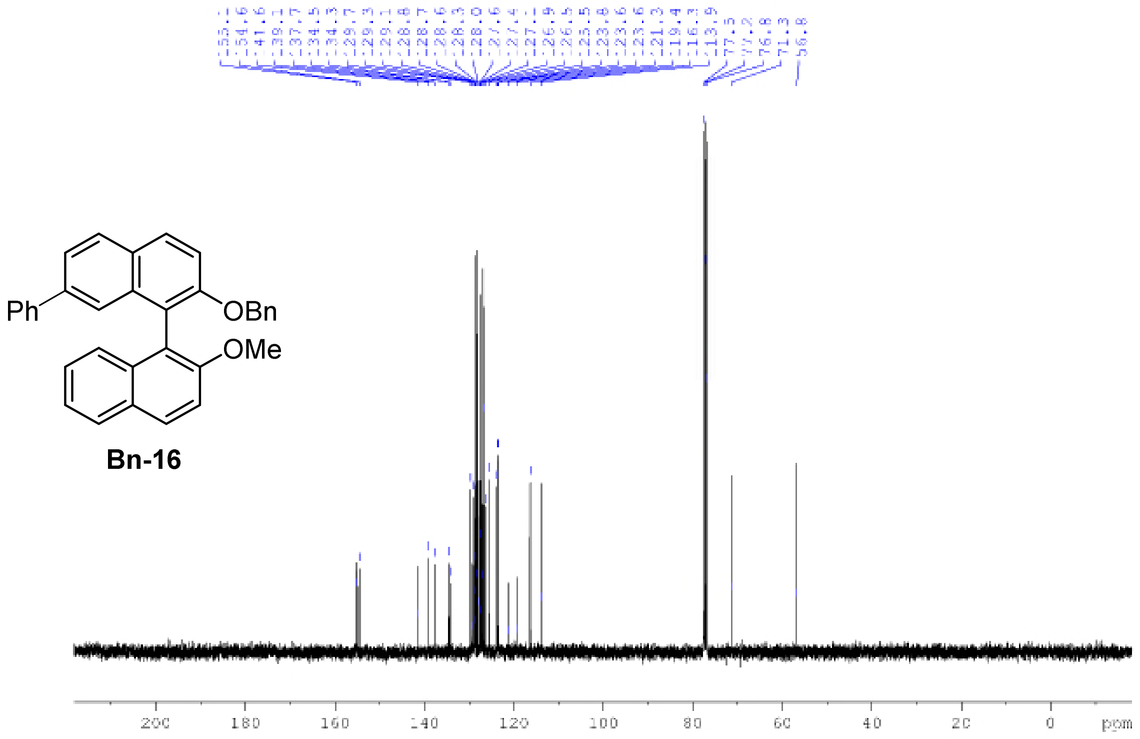
Ph
OBn
OMe
Bn-16

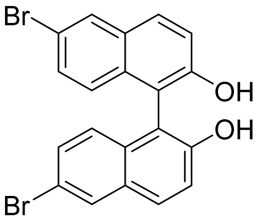

54

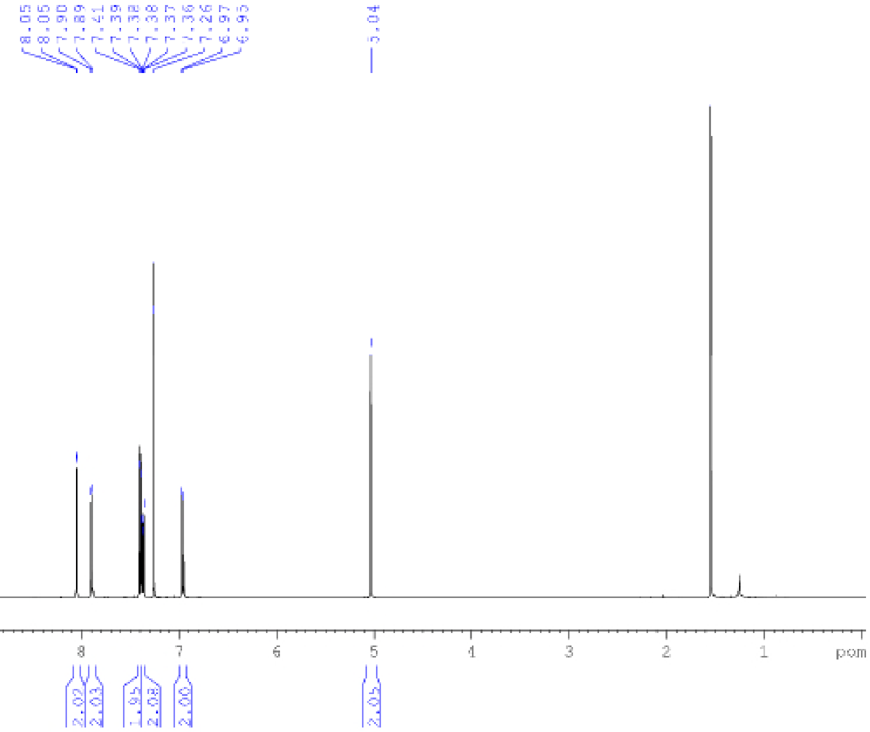

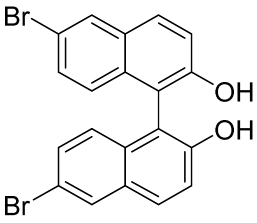

54

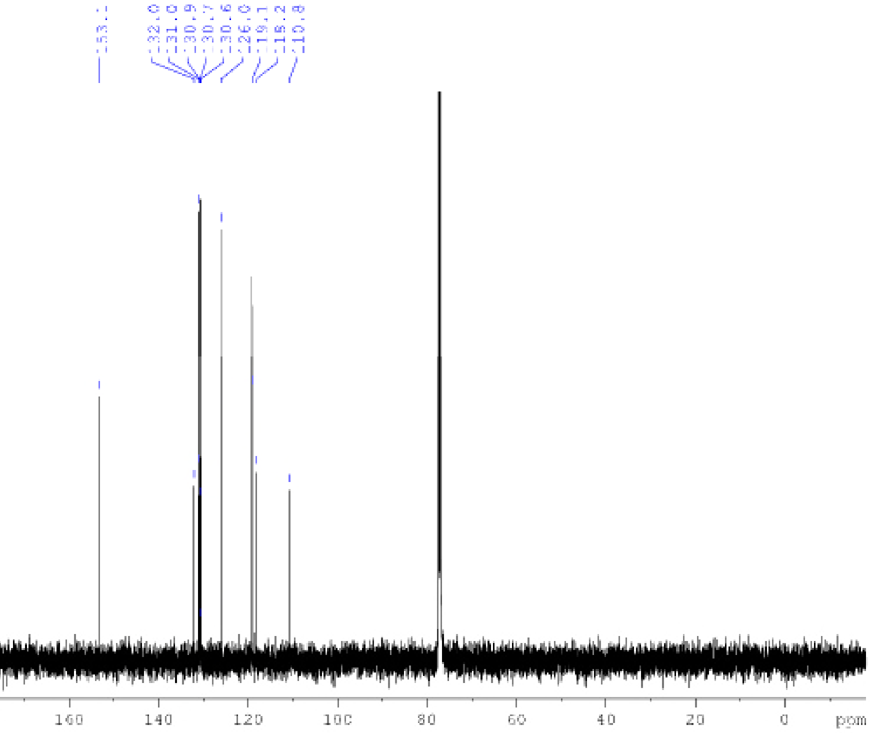

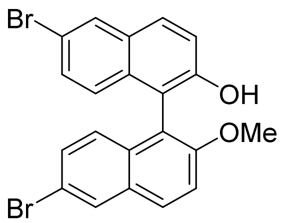
Br
OH
OMe
Br

33

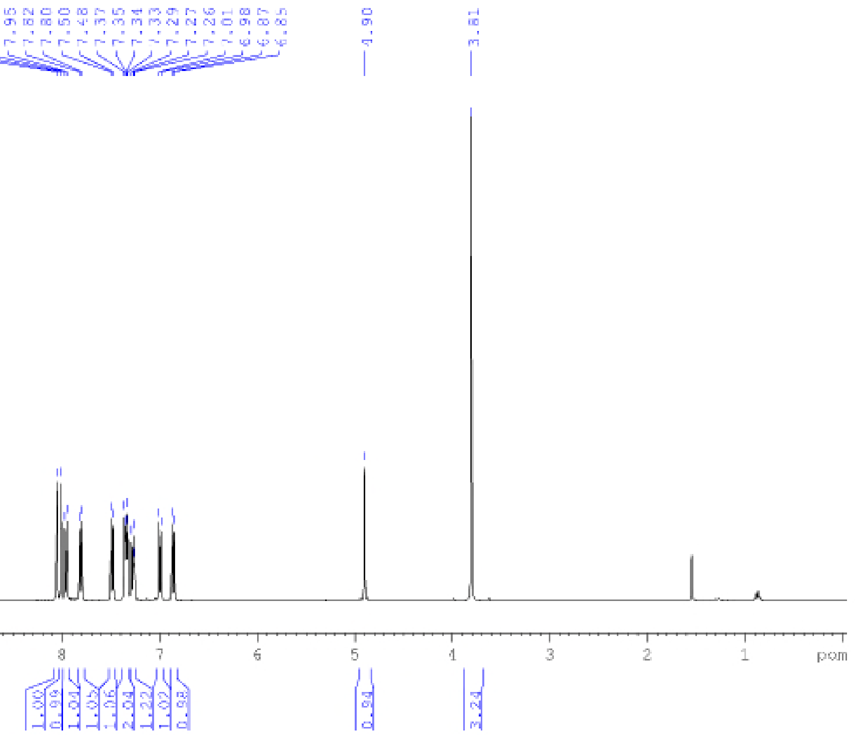
8.05
8.01
7.97
7.95
7.82
7.80
7.50
7.48
7.37
7.35
7.34
7.33
7.29
7.27
7.26
7.01
6.98
6.87
6.85
4.90
3.81
1.00
0.99
1.04
1.05
1.06
2.04
1.22
1.02
0.98
0.94
3.24
ppm

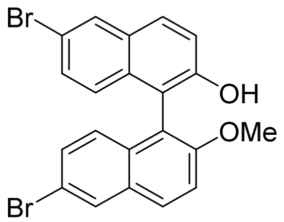
Br
OH
OMe
Br

33

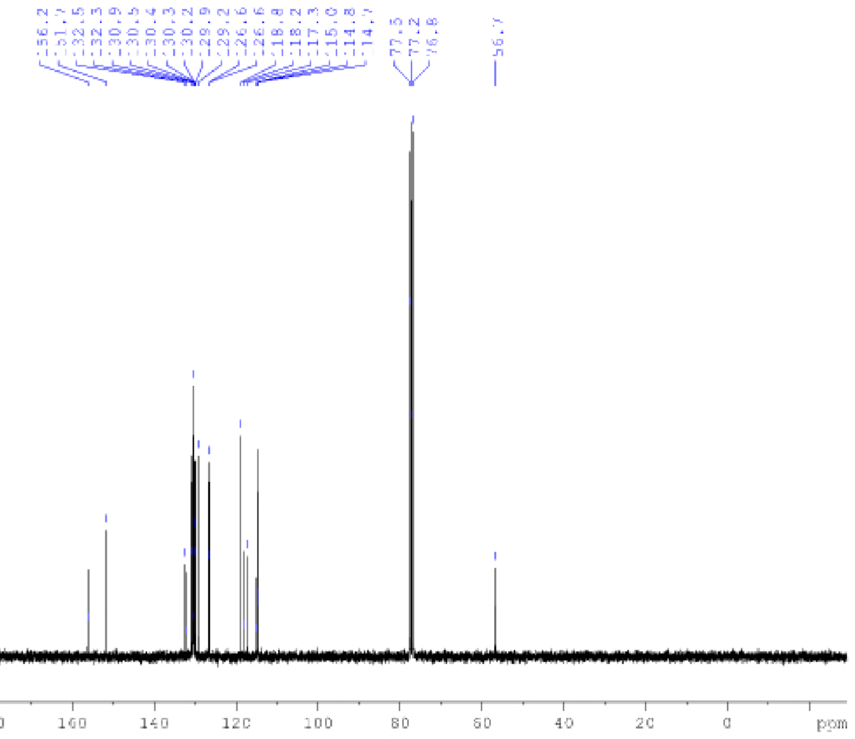
77.5
77.2
76.8
56.7
ppm

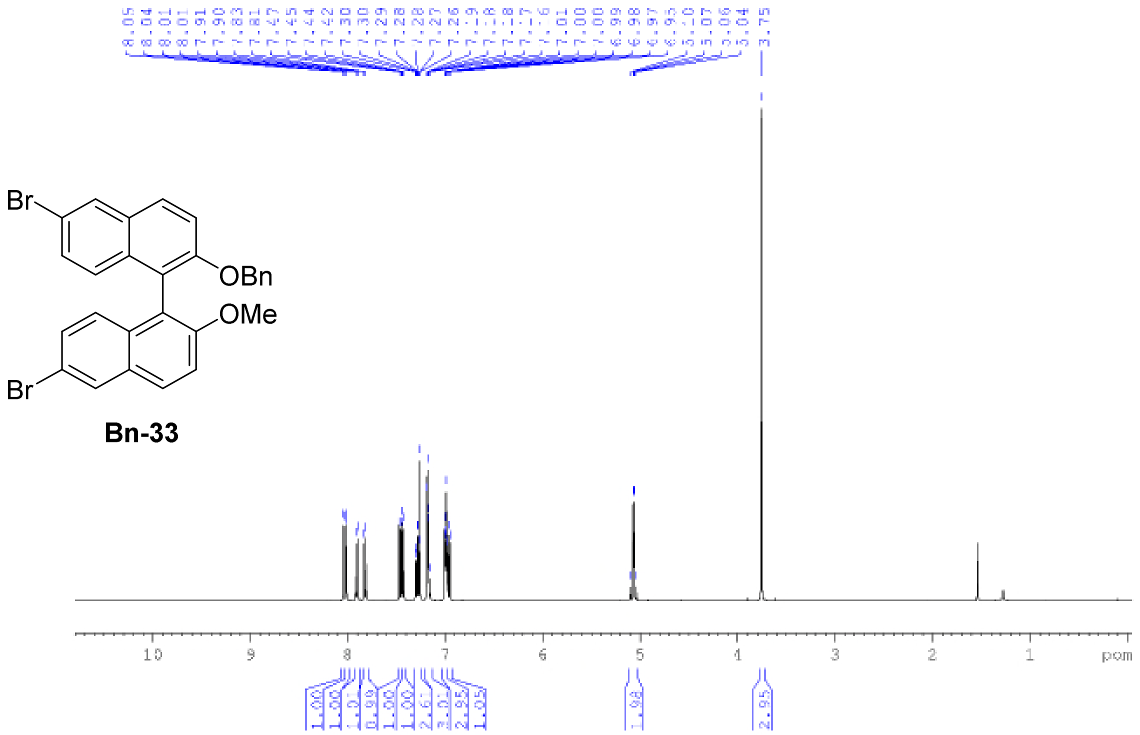

Bn-33

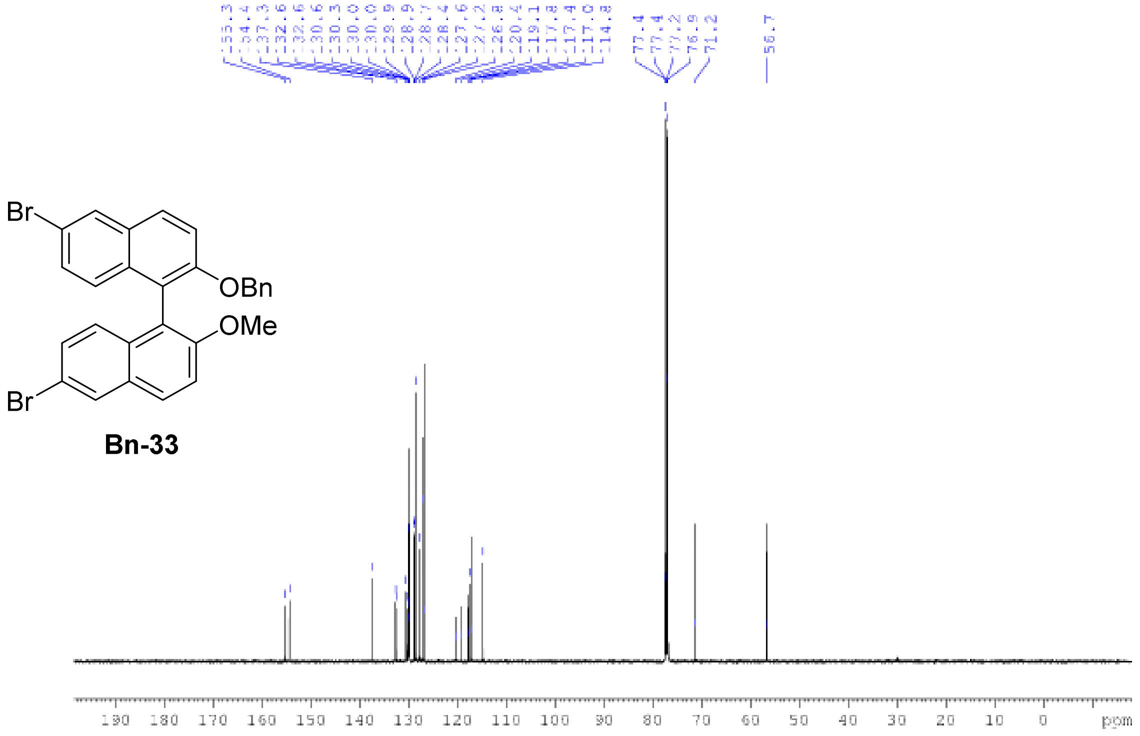

Bn-33

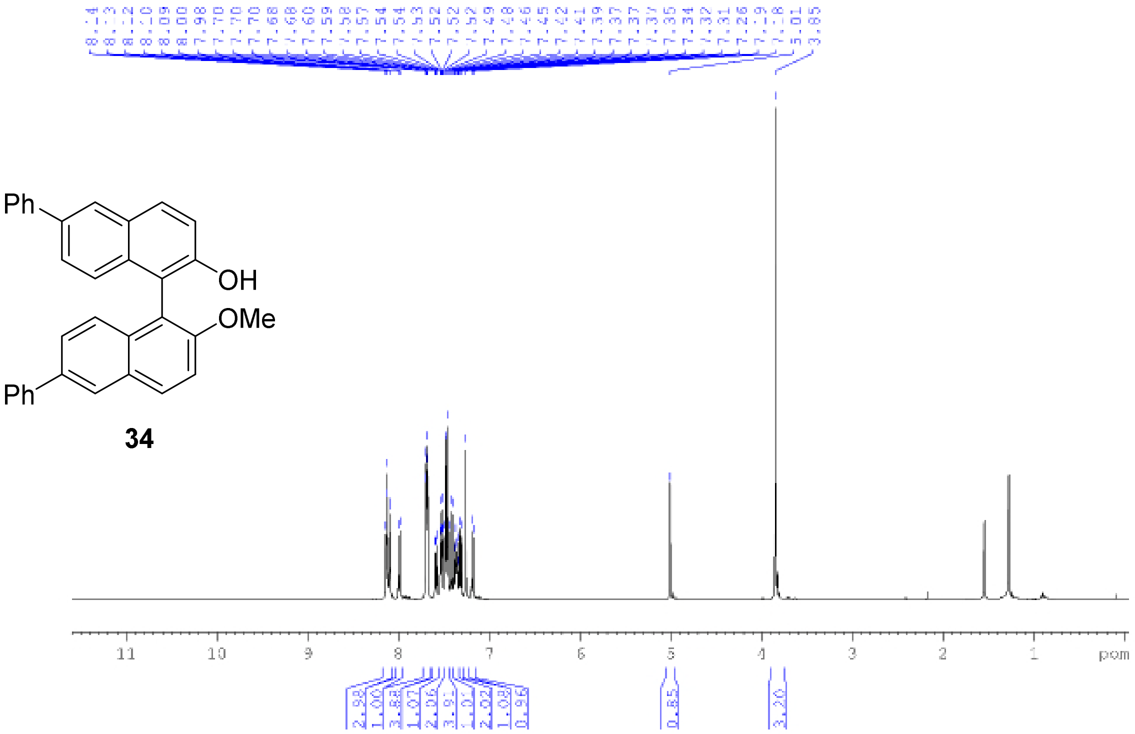
Ph
OH
OMe
Ph
34

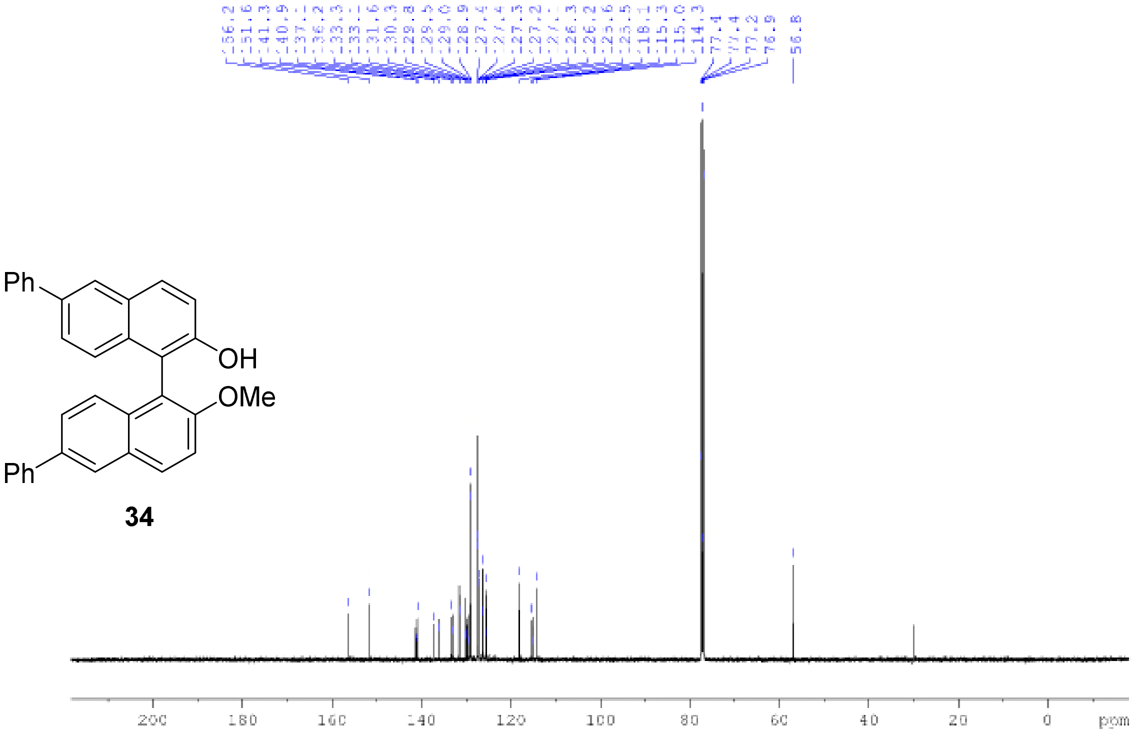
Ph
OH
OMe
Ph
34

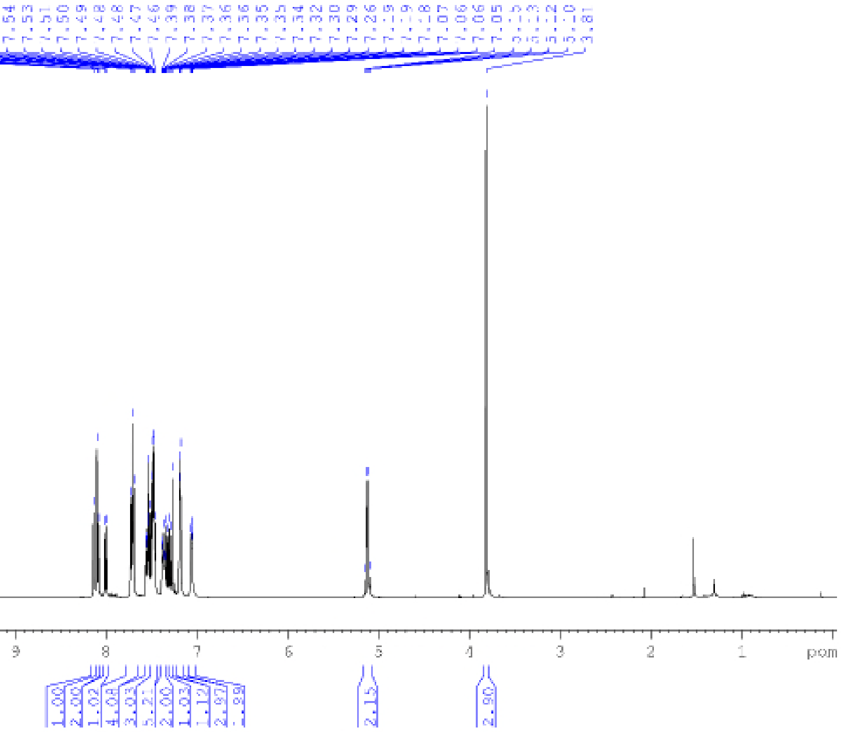

11
10
9
8
7
6
5
4
3
2
1
ppm

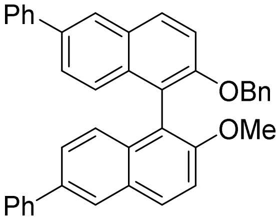

Ph
OBn
OMe
Ph

Bn-34

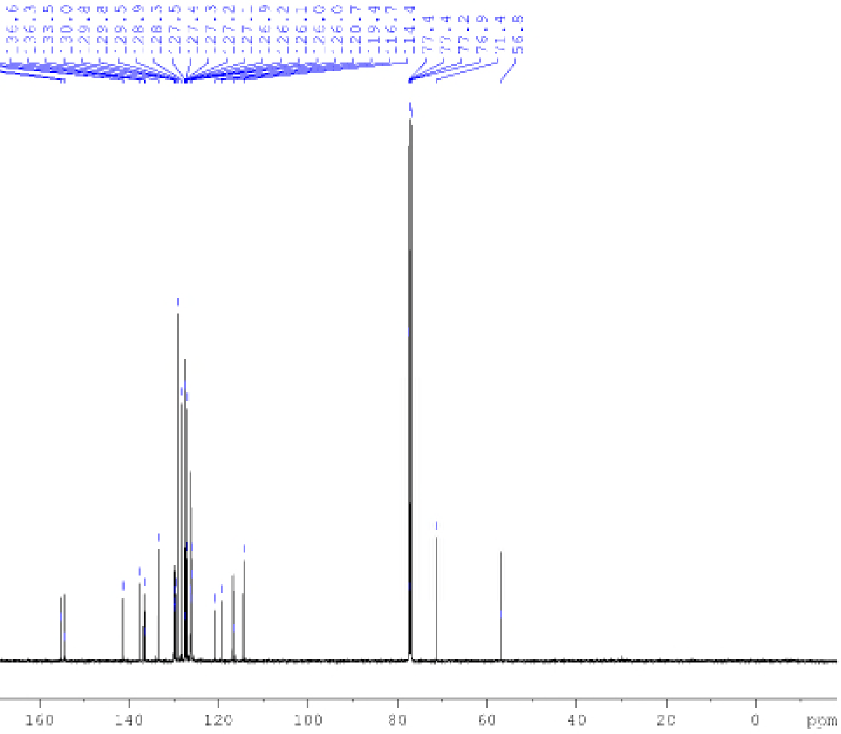

200
180
160
140
120
100
80
60
40
20
0
ppm

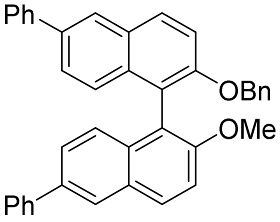

Ph
OBn
OMe
Ph

Bn-34

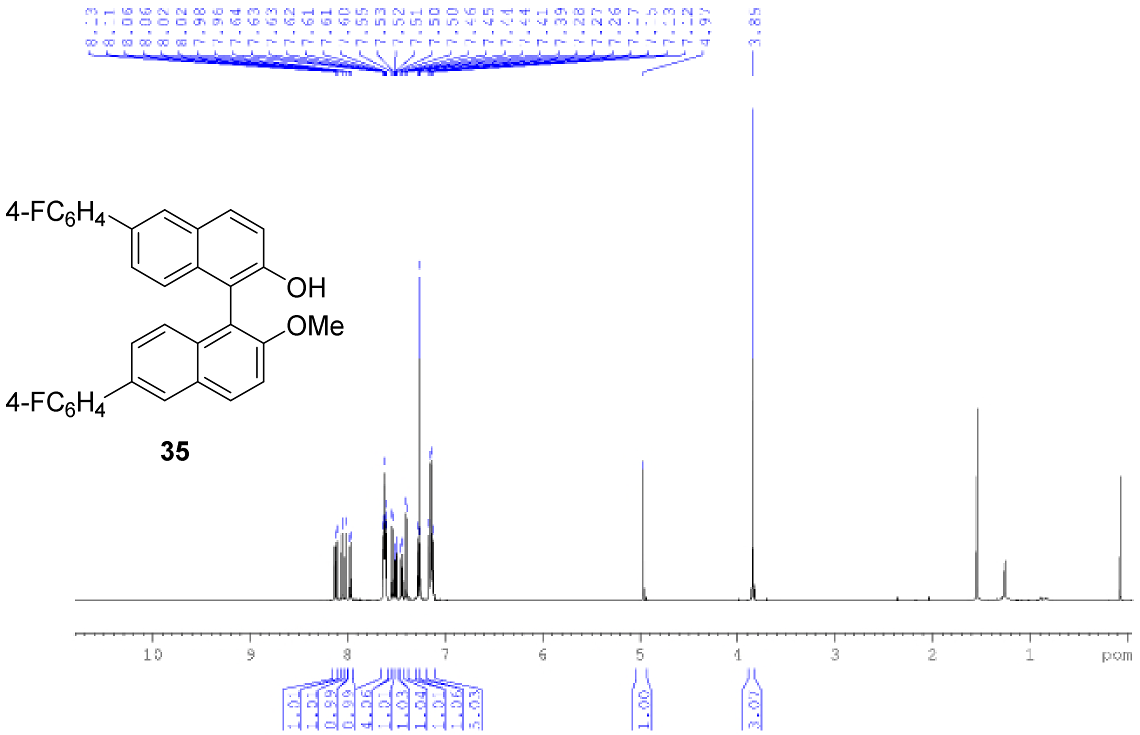

35

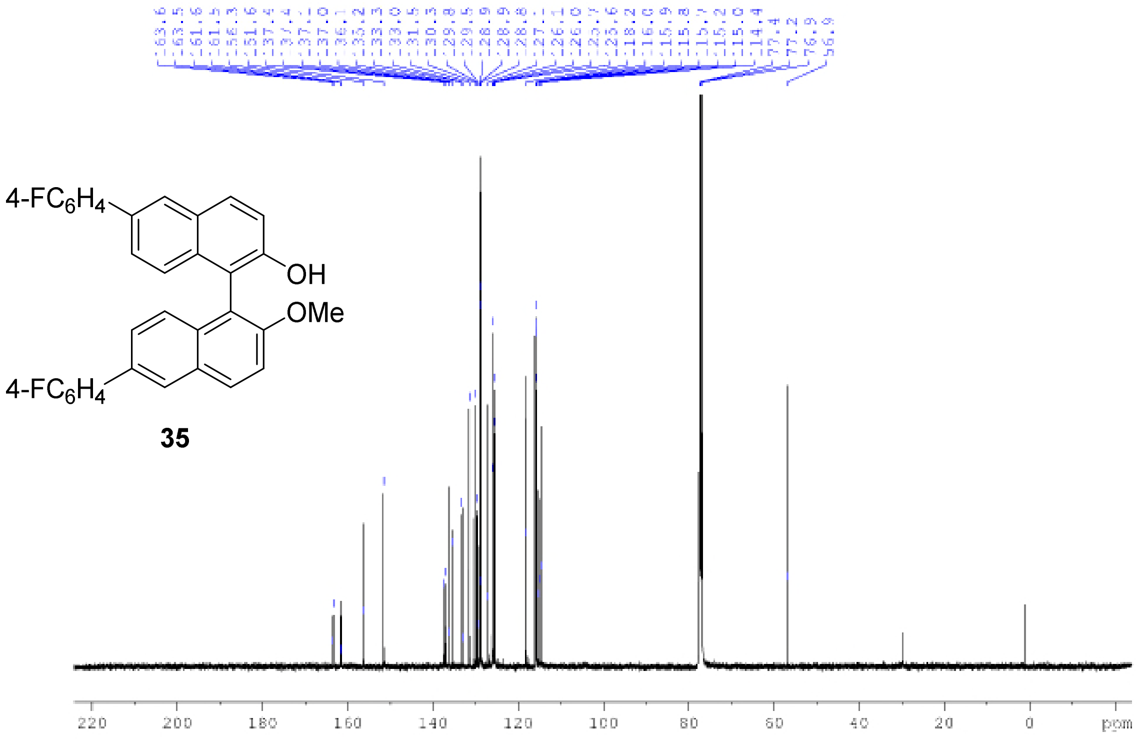

35

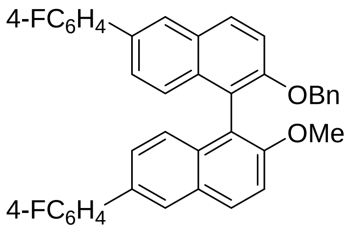

Bn-35

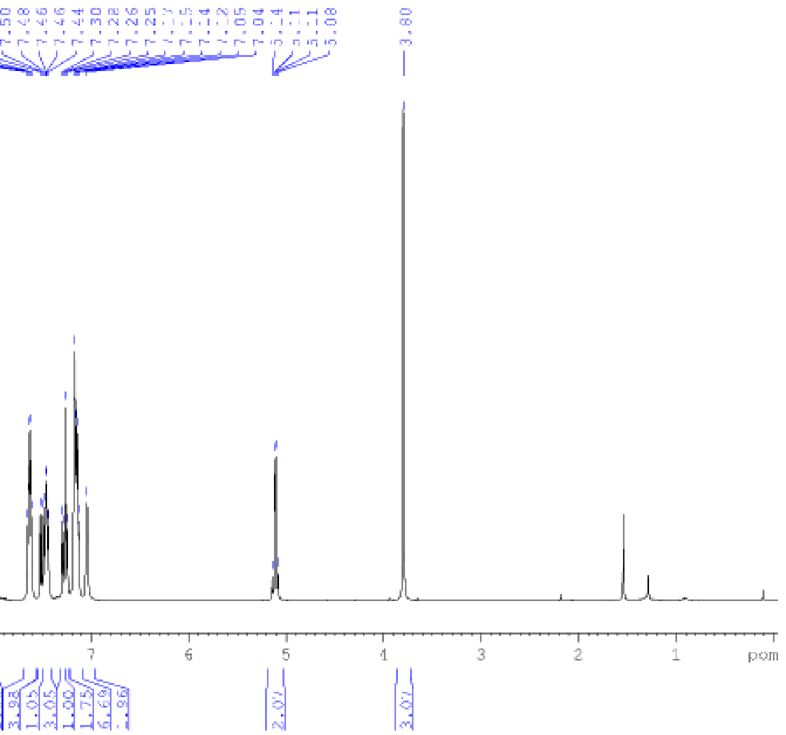

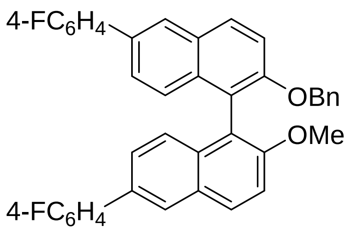

Bn-35

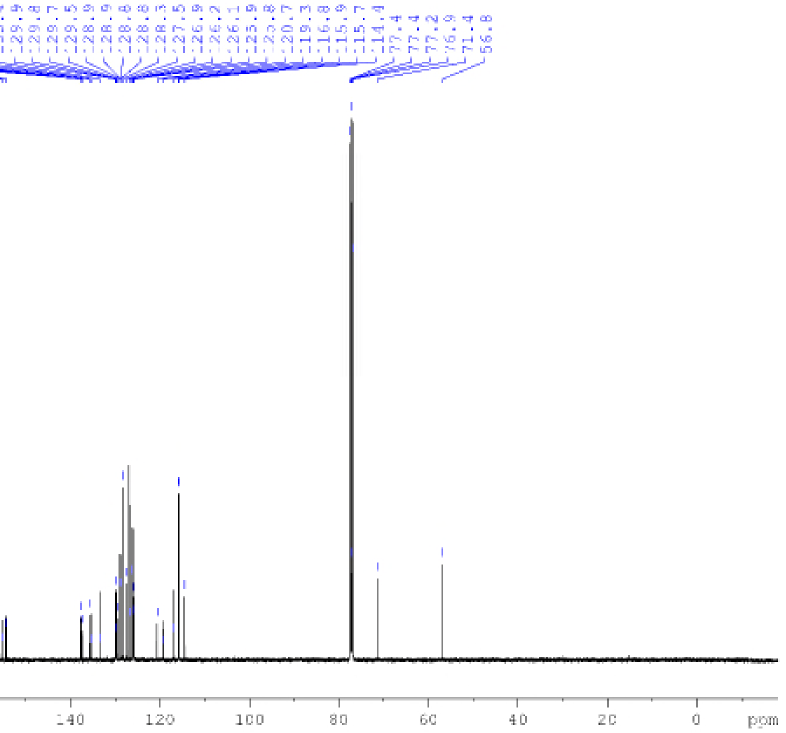

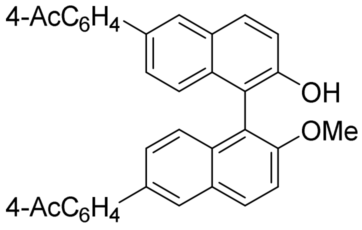

36

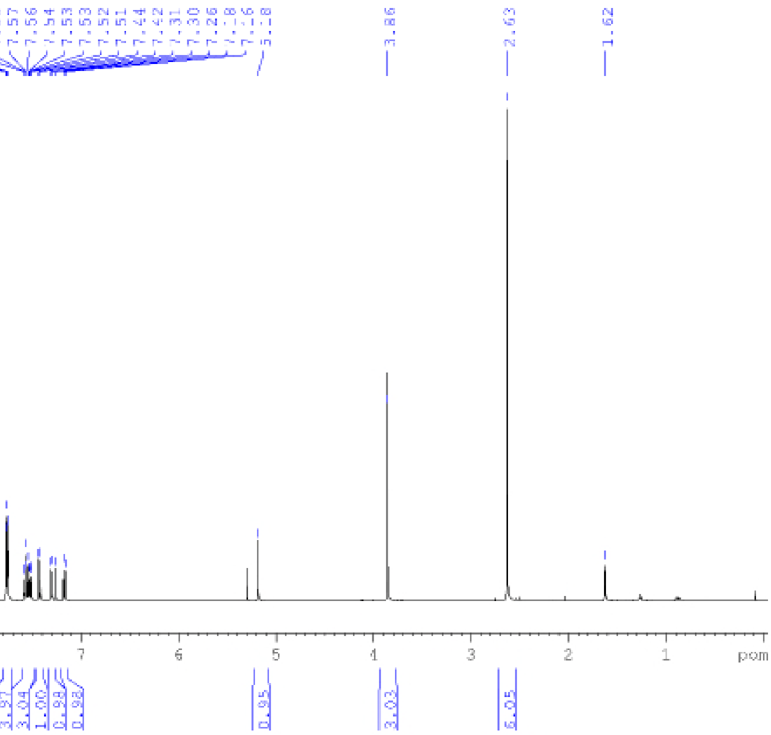

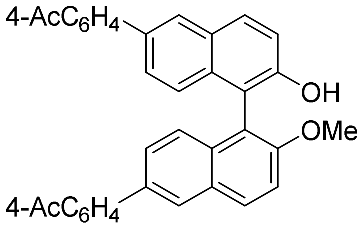

36

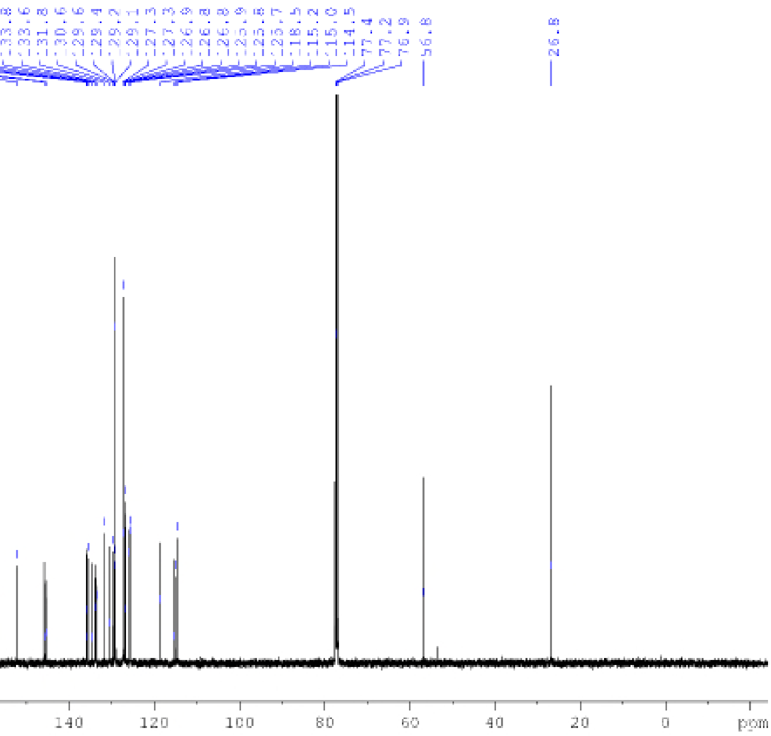

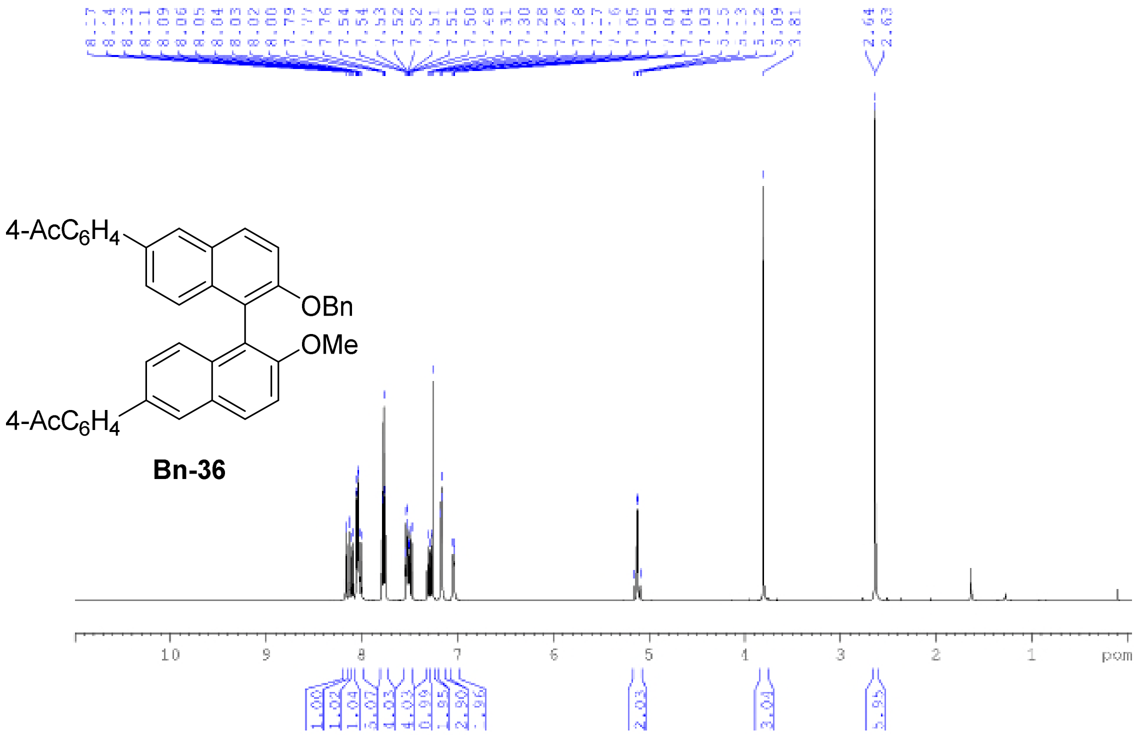

Bn-36

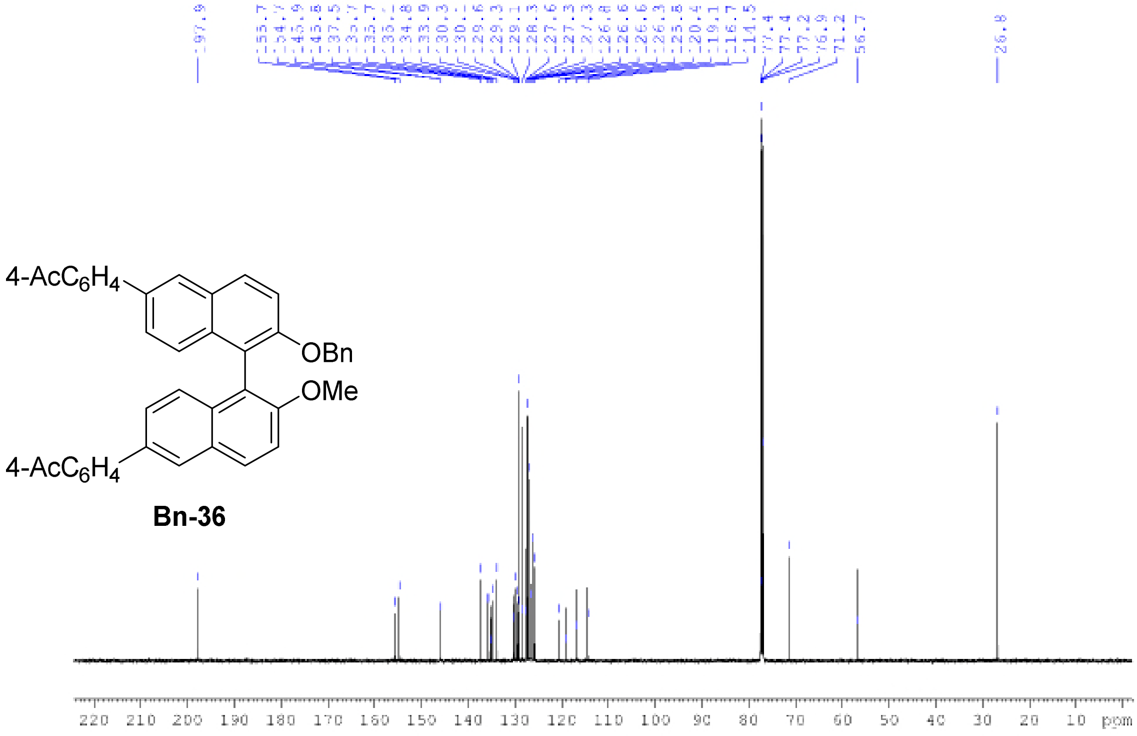

Bn-36

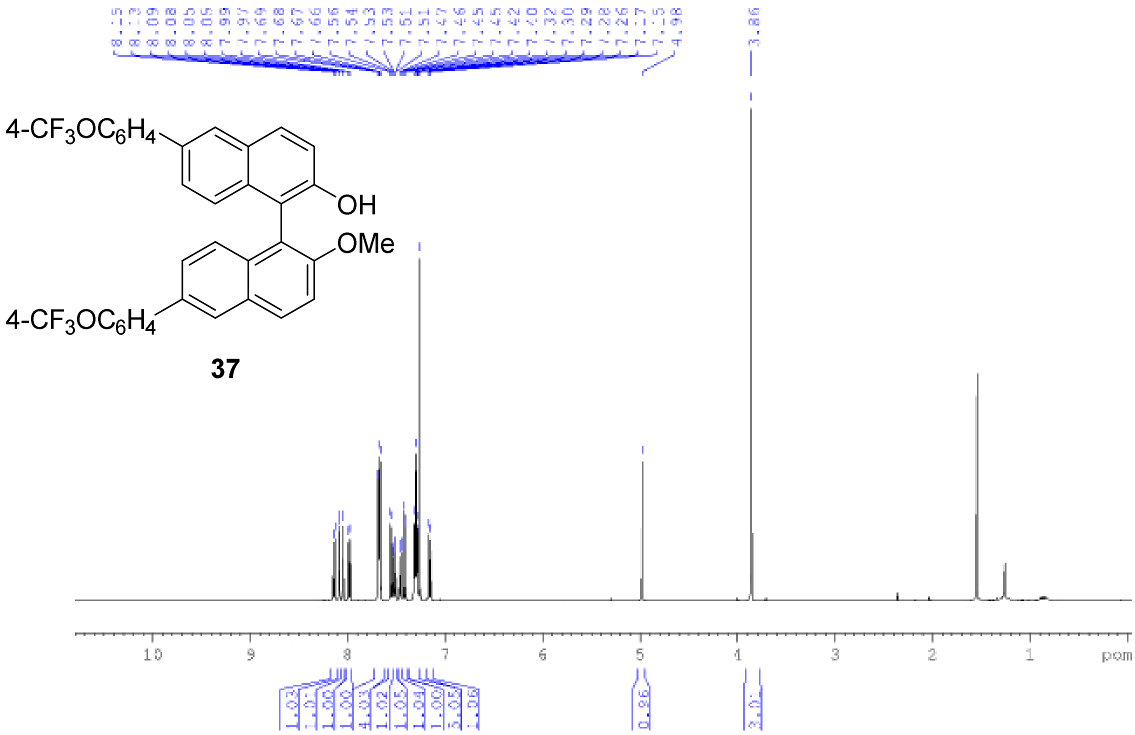
4-CF3OC6H4
OH
OMe
4-CF3OC6H4
37
ppm

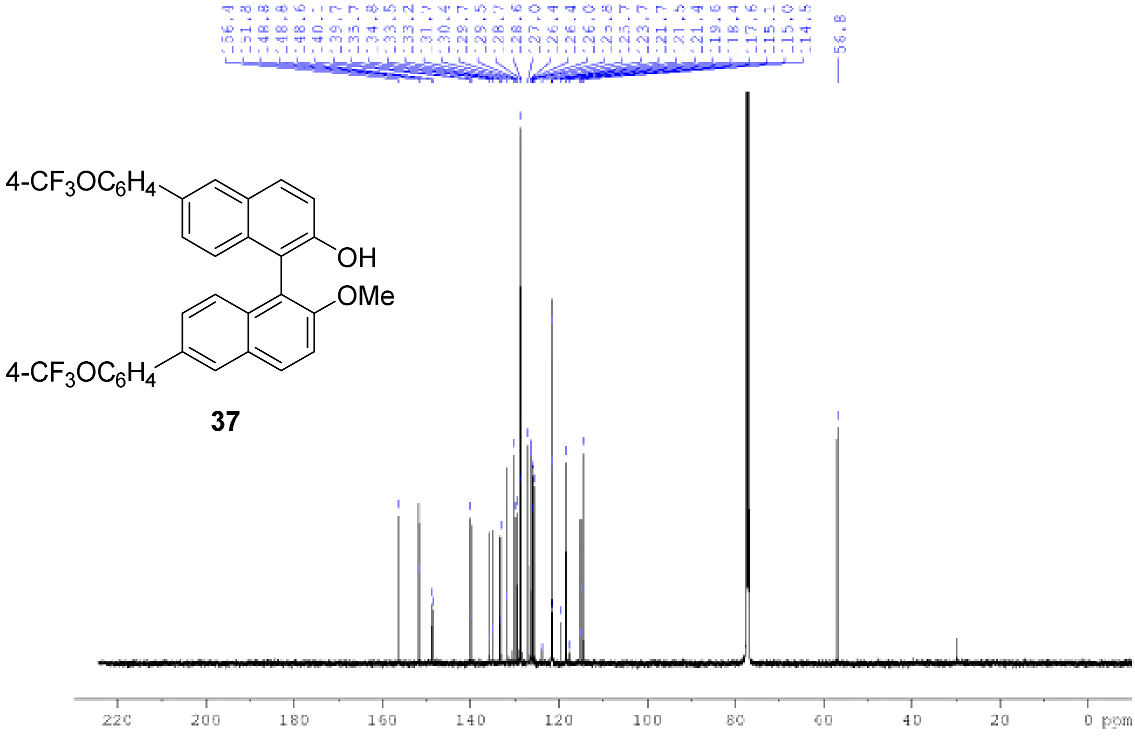
4-CF3OC6H4
OH
OMe
4-CF3OC6H4
37
ppm

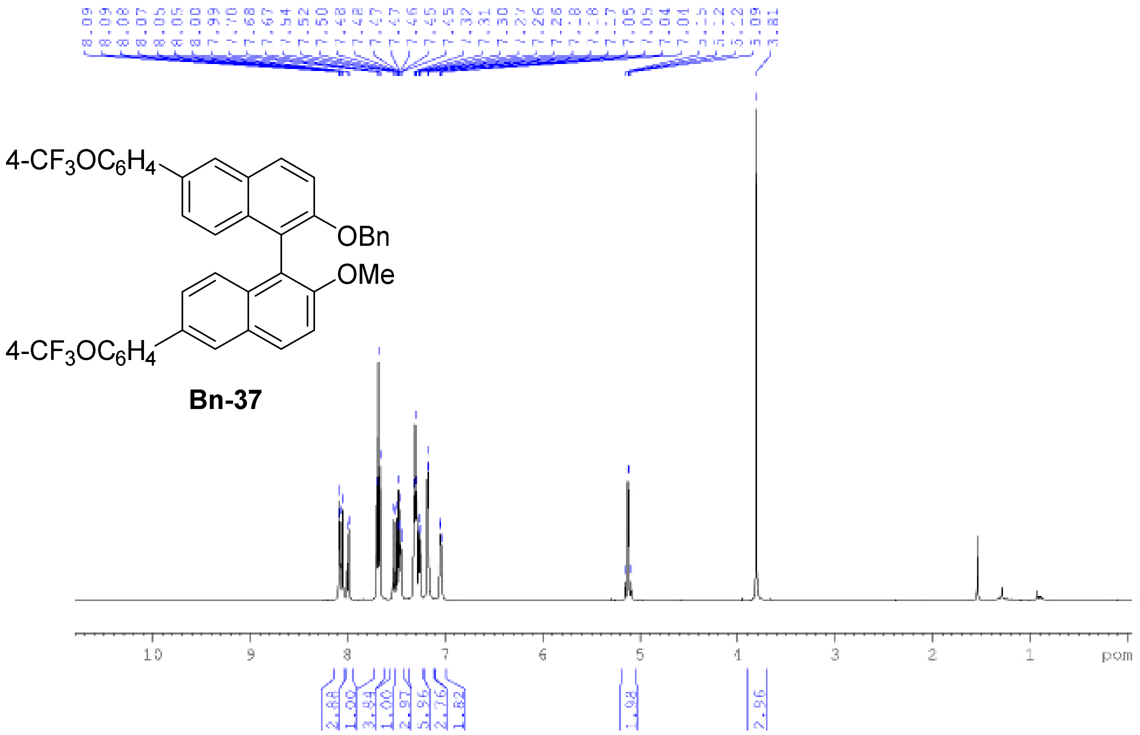

4-CF3OC6H4
OBn
OMe
4-CF3OC6H4
ppm

Bn-37

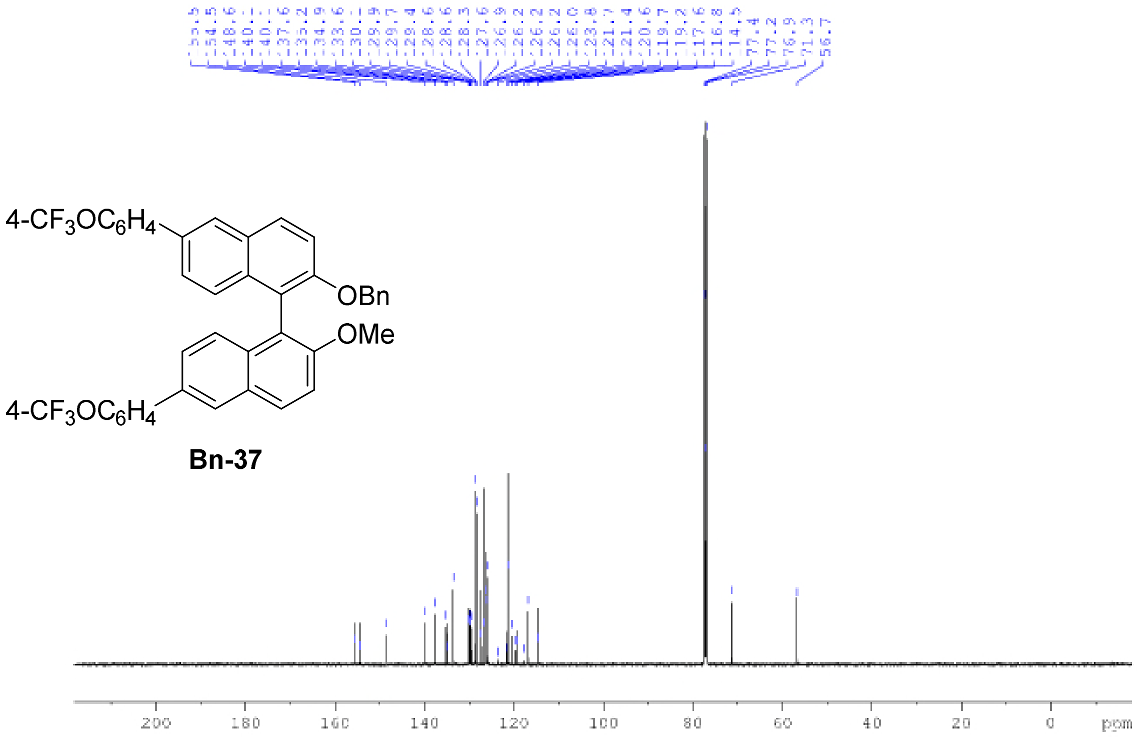

4-CF3OC6H4
OBn
OMe
4-CF3OC6H4
ppm

Bn-37

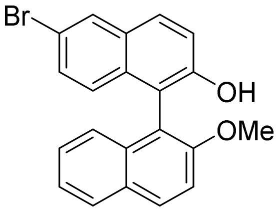

21

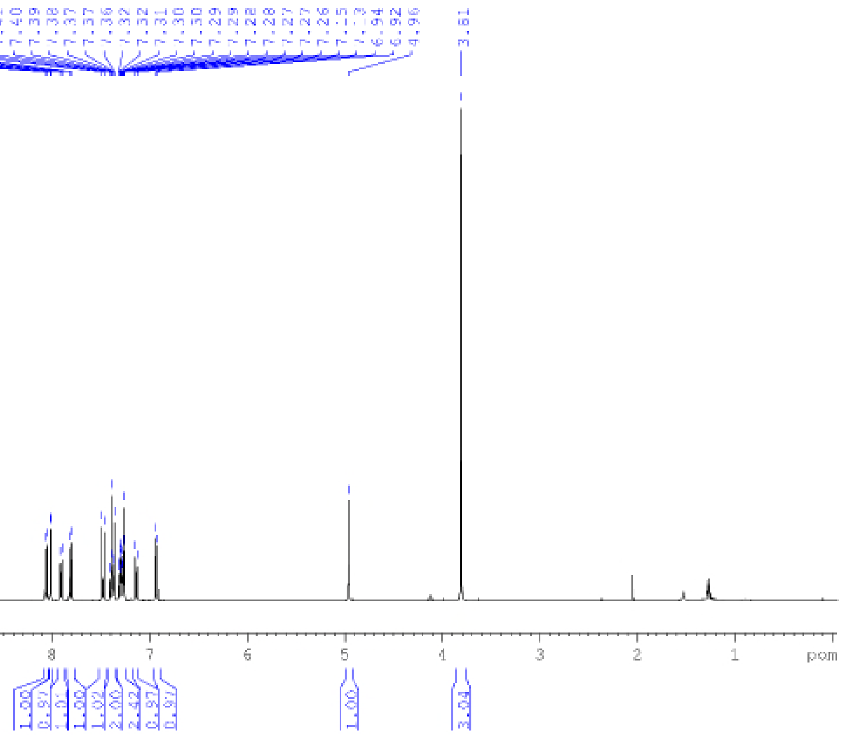

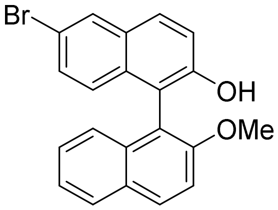

21

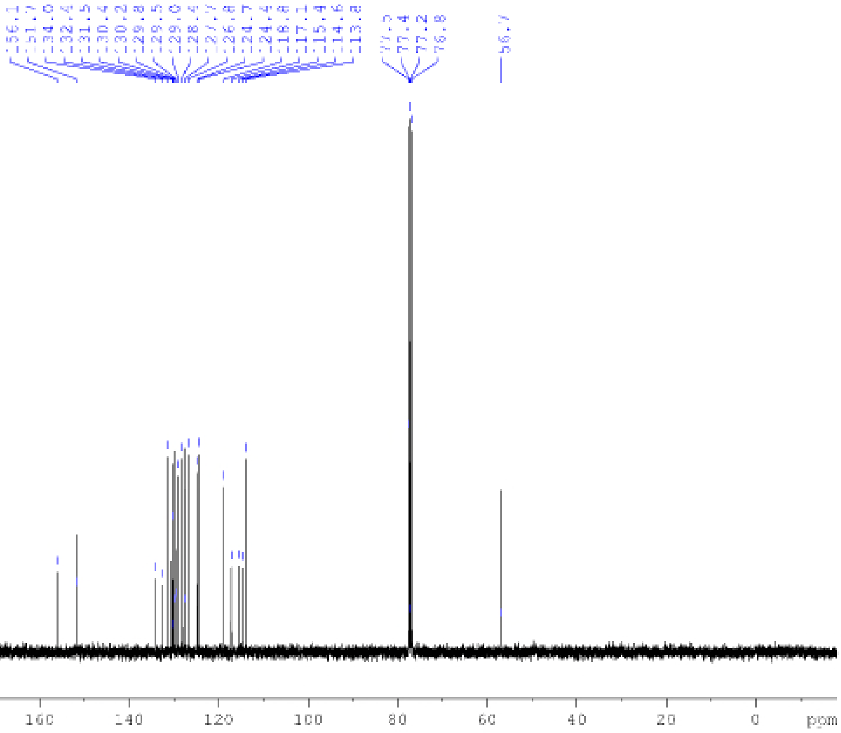

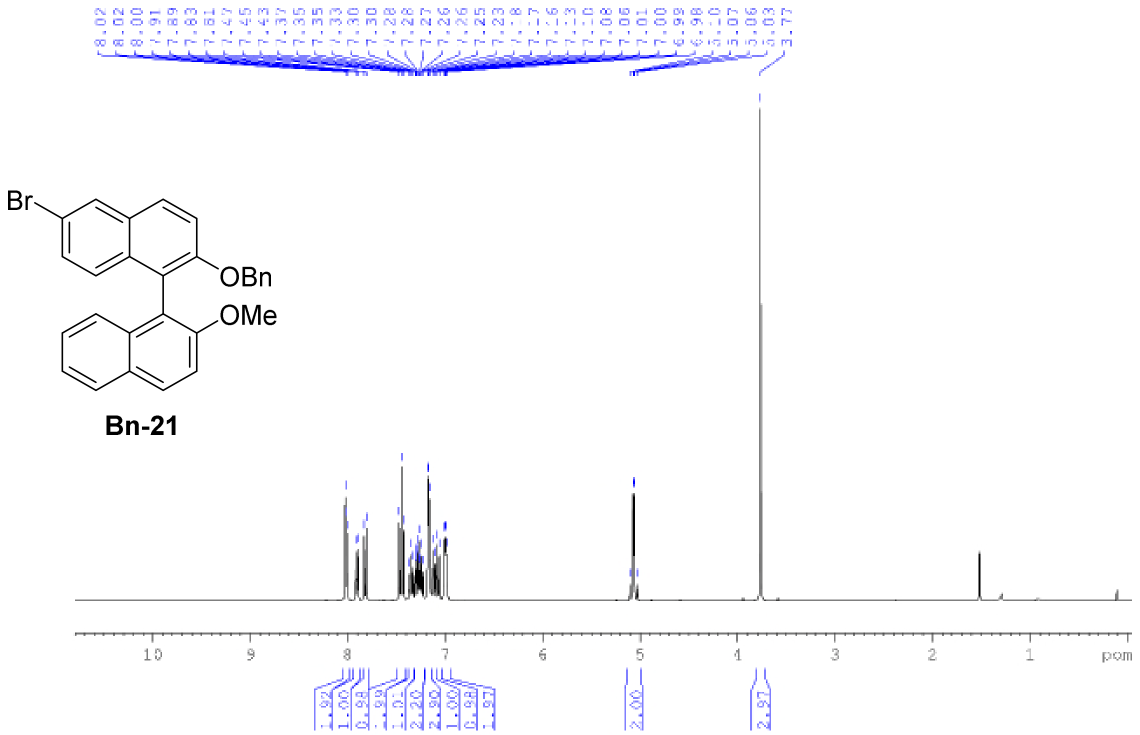
Br
OBn
OMe
Bn-21
ppm

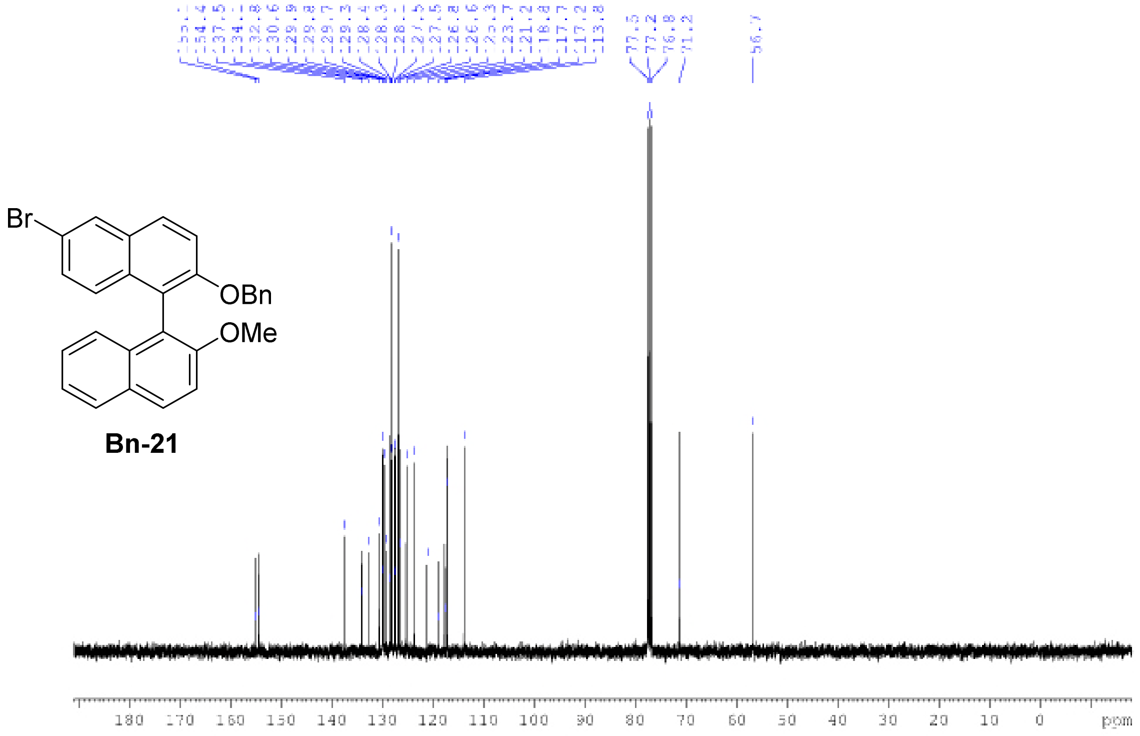
Br
OBn
OMe
Bn-21
ppm

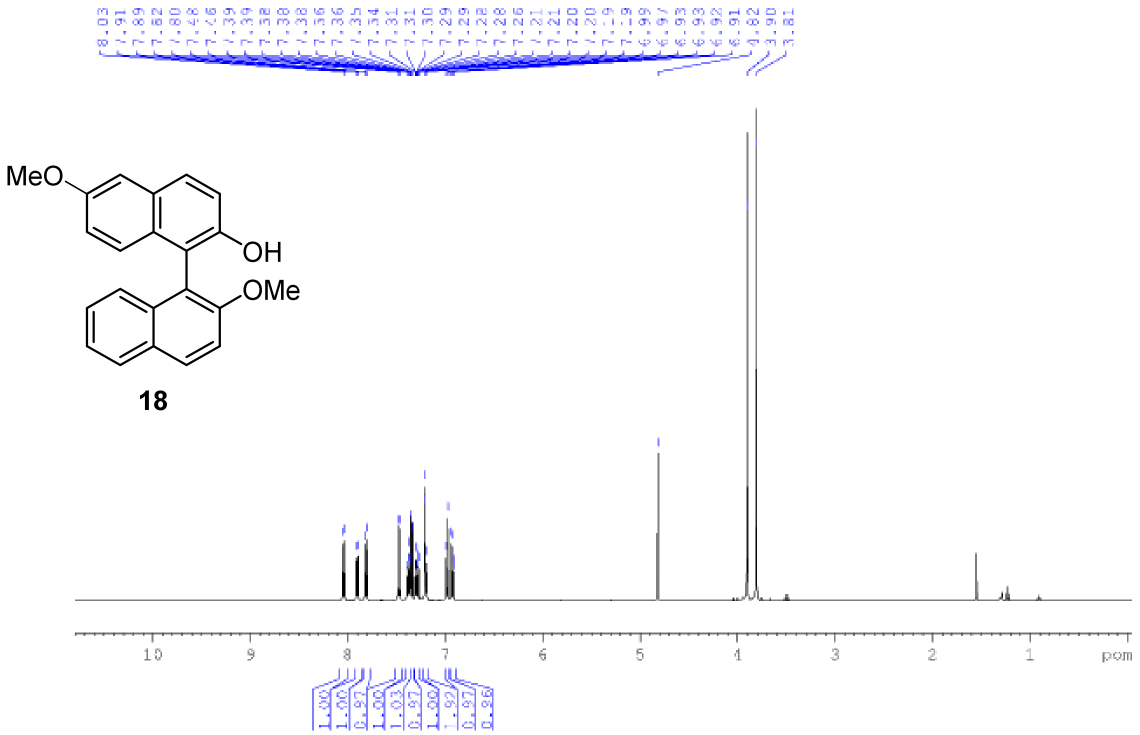
MeO
OH
OMe
18
ppm

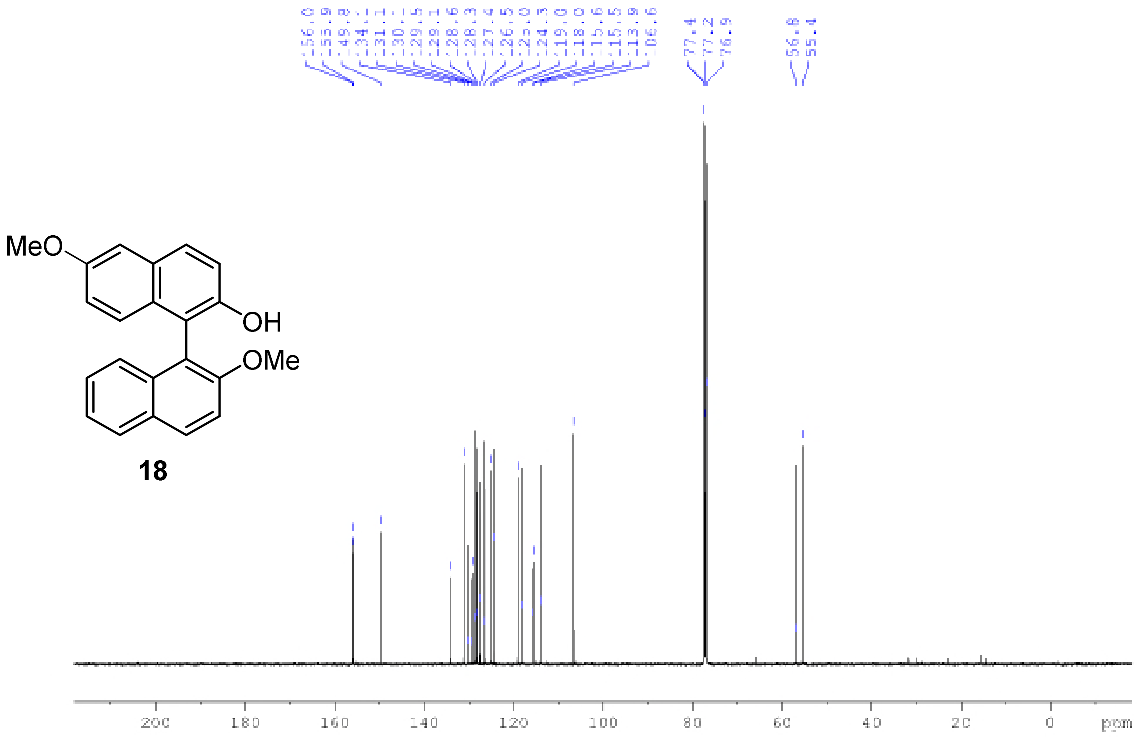
MeO
OH
OMe
18
ppm

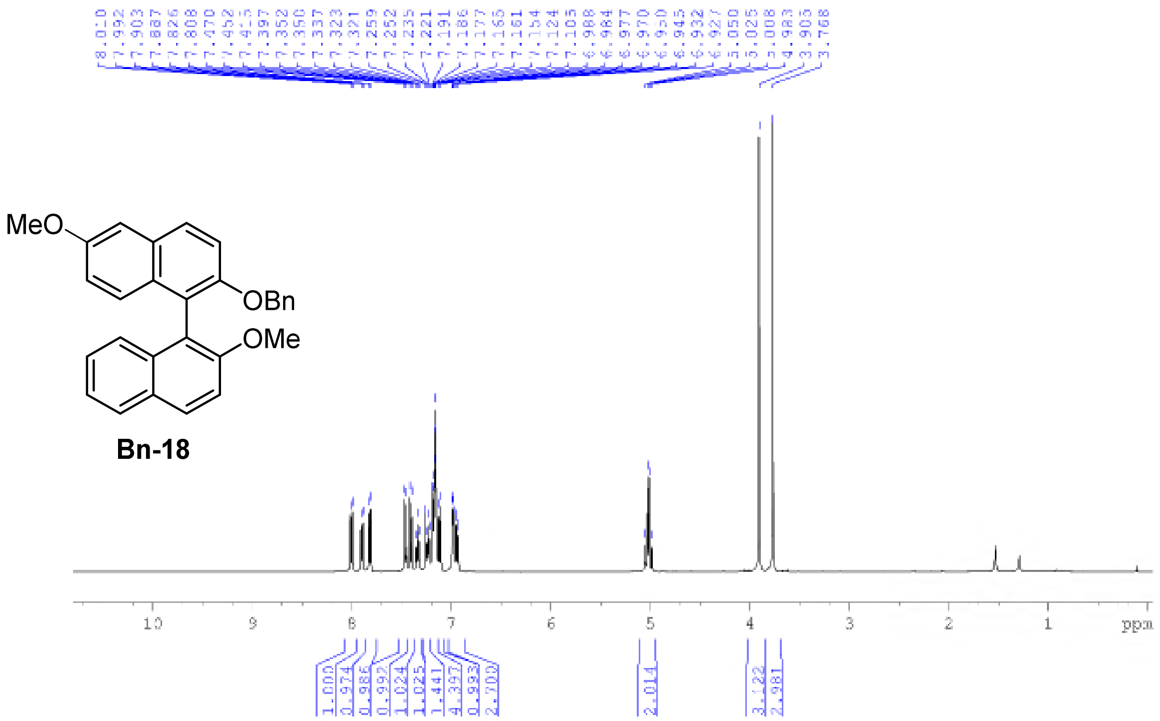
MeO
OBn
OMe
Bn-18
8.010
7.992
7.903
7.887
7.826
7.808
7.470
7.452
7.415
7.397
7.352
7.350
7.337
7.323
7.321
7.259
7.252
7.235
7.221
7.191
7.186
7.177
7.165
7.161
7.154
7.124
7.105
6.988
6.984
6.977
6.970
6.950
6.945
6.932
6.927
5.050
5.025
5.008
4.983
3.905
3.768
ppm

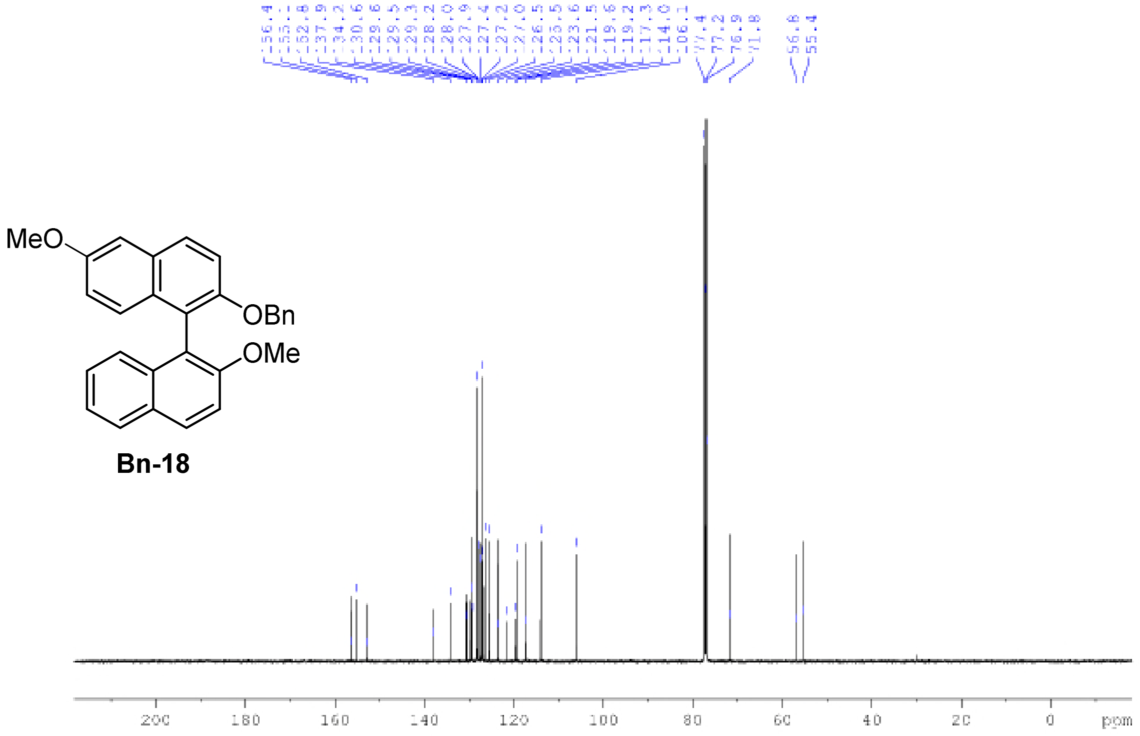
MeO
OBn
OMe
Bn-18
ppm

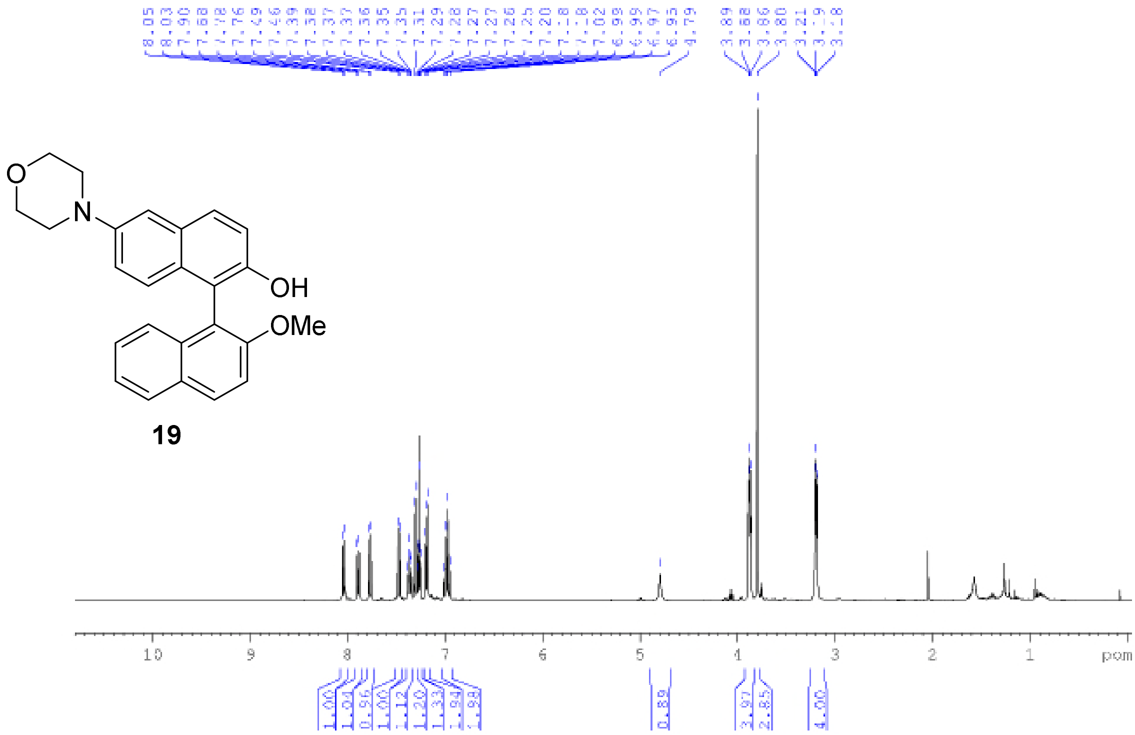

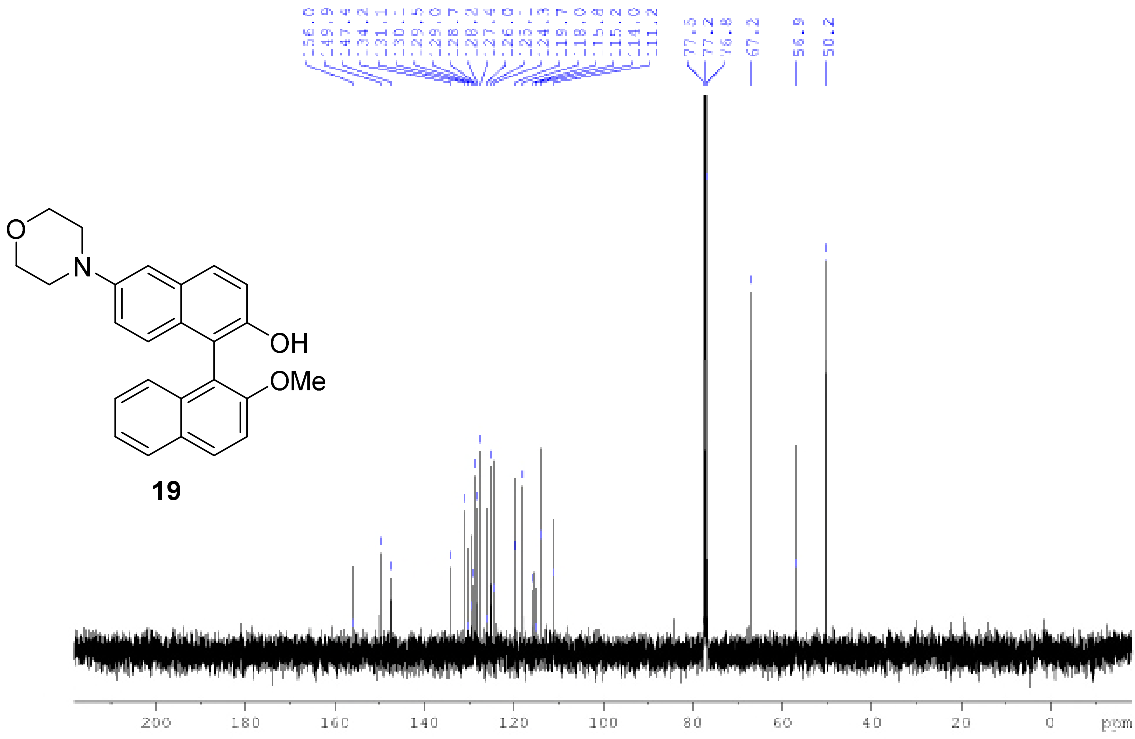

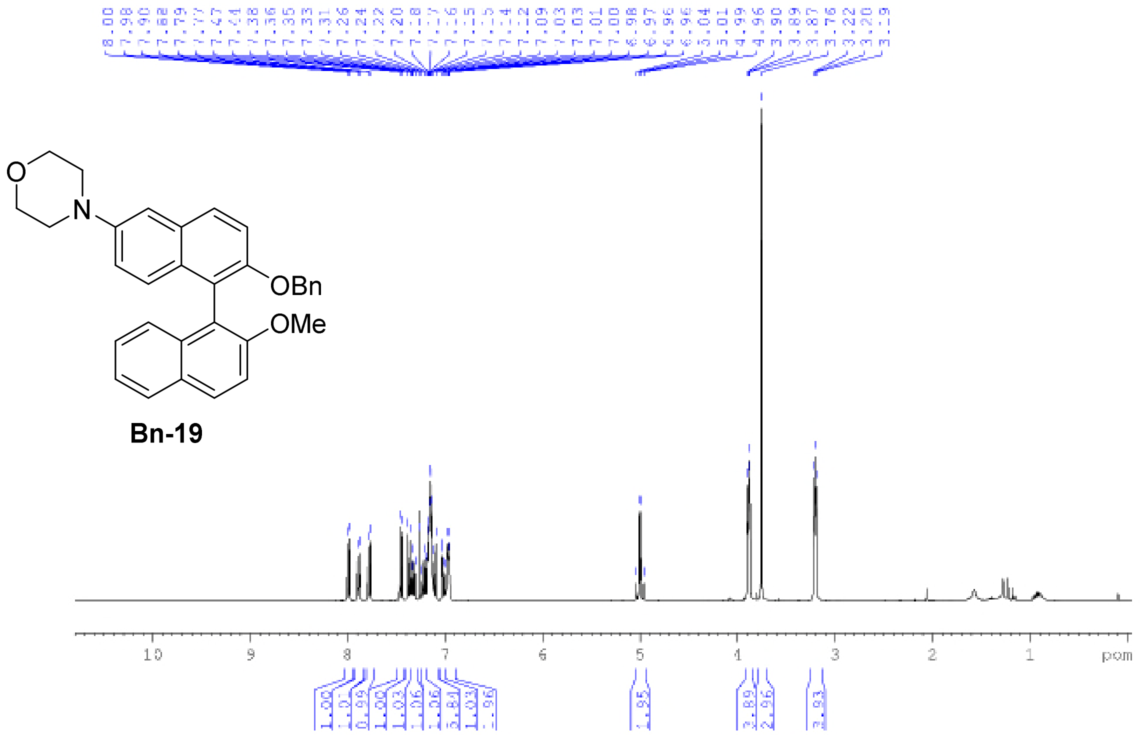

Bn-19

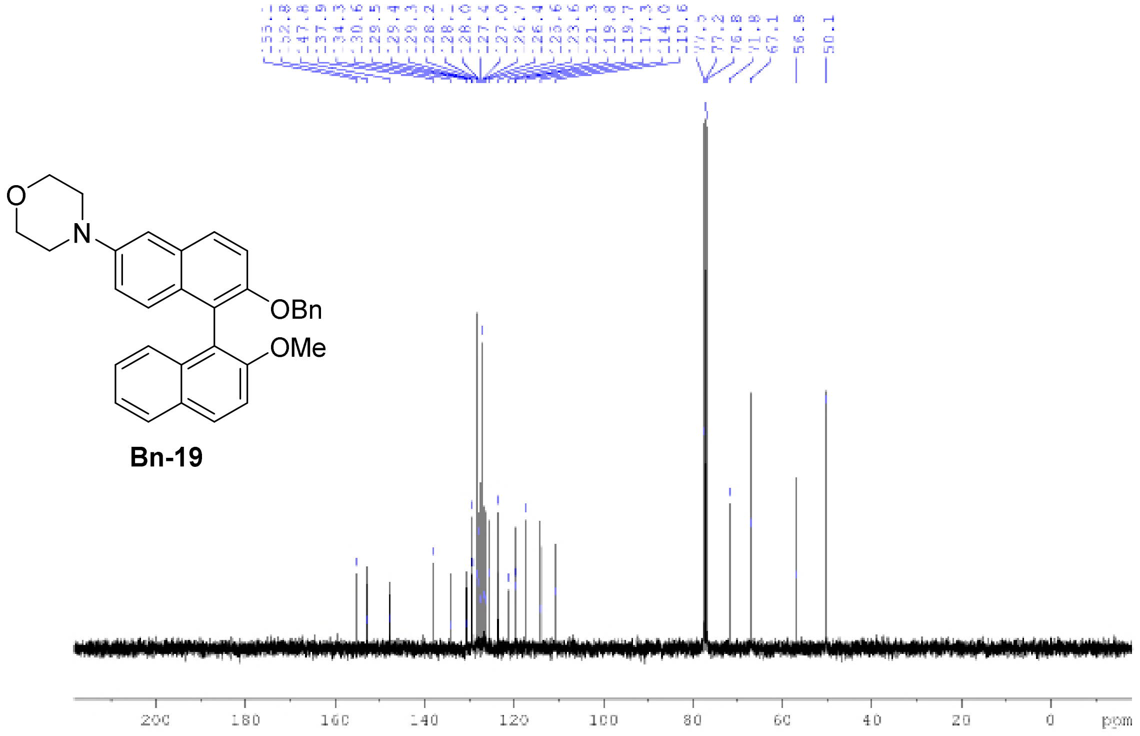

Bn-19

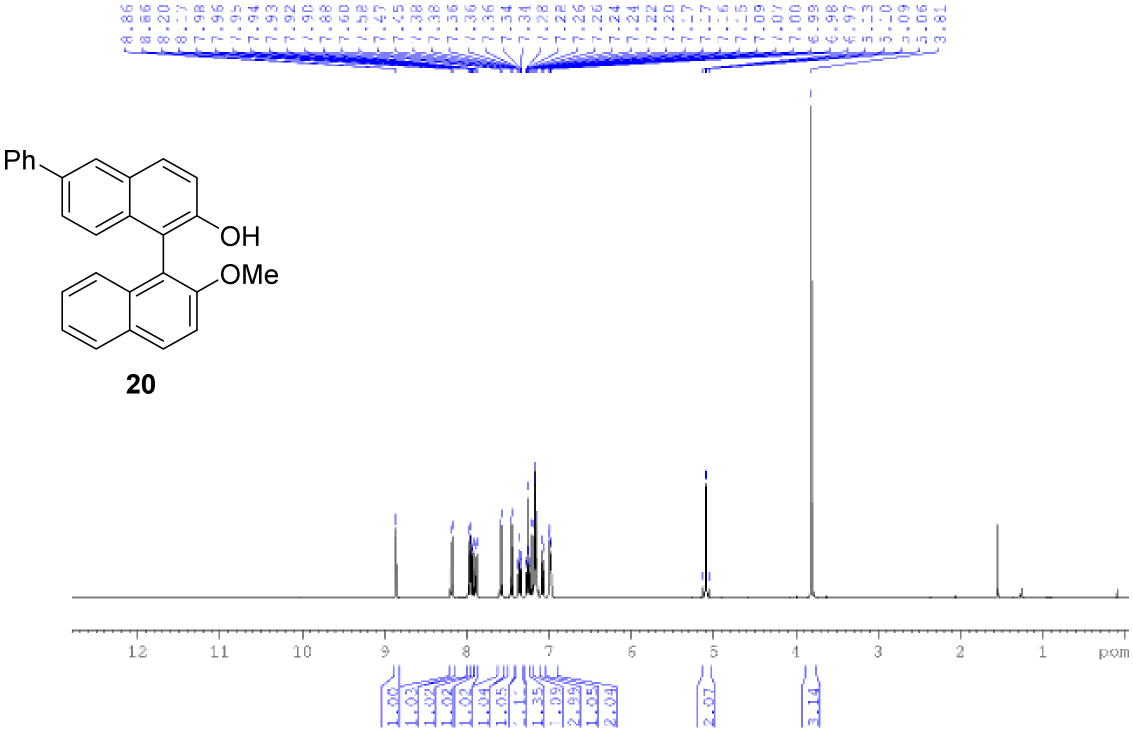
Ph
OH
OMe
20
ppm

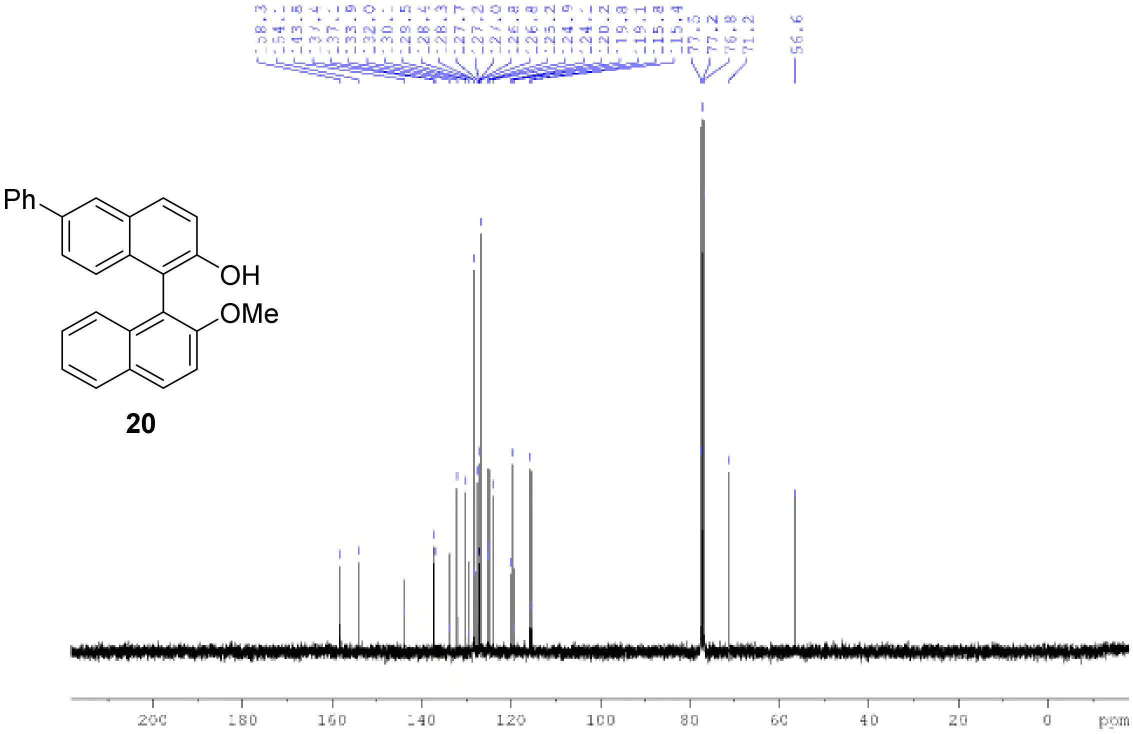
Ph
OH
OMe
20
ppm

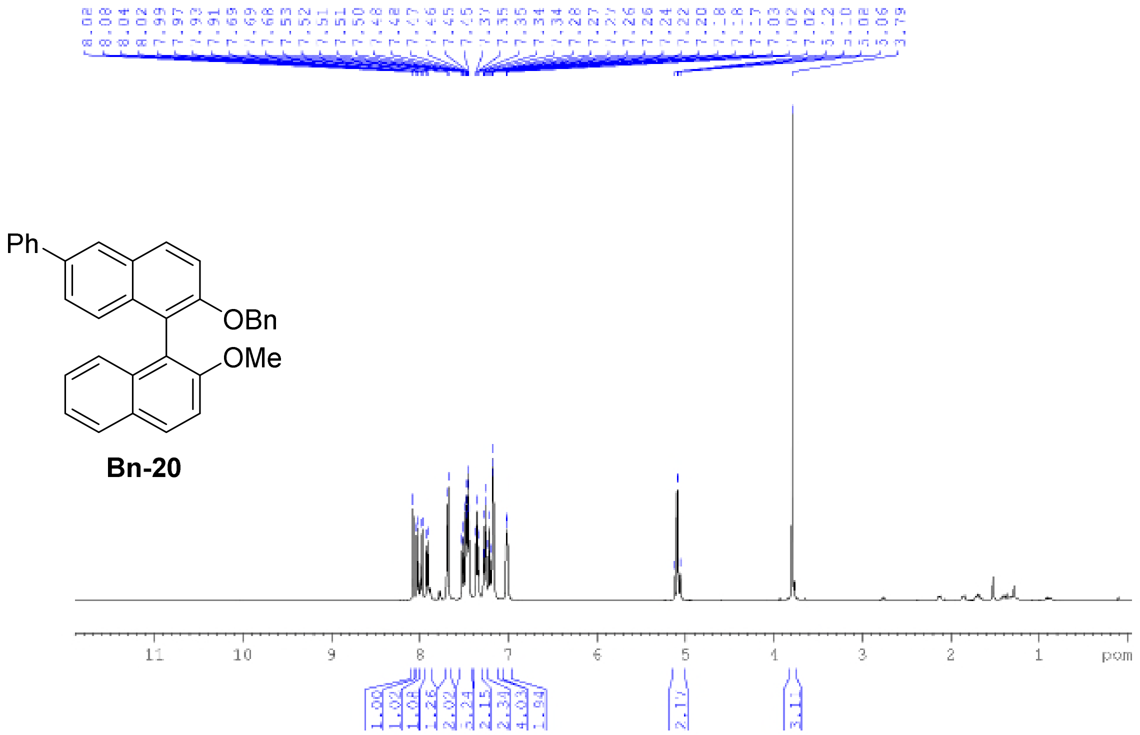

Bn-20

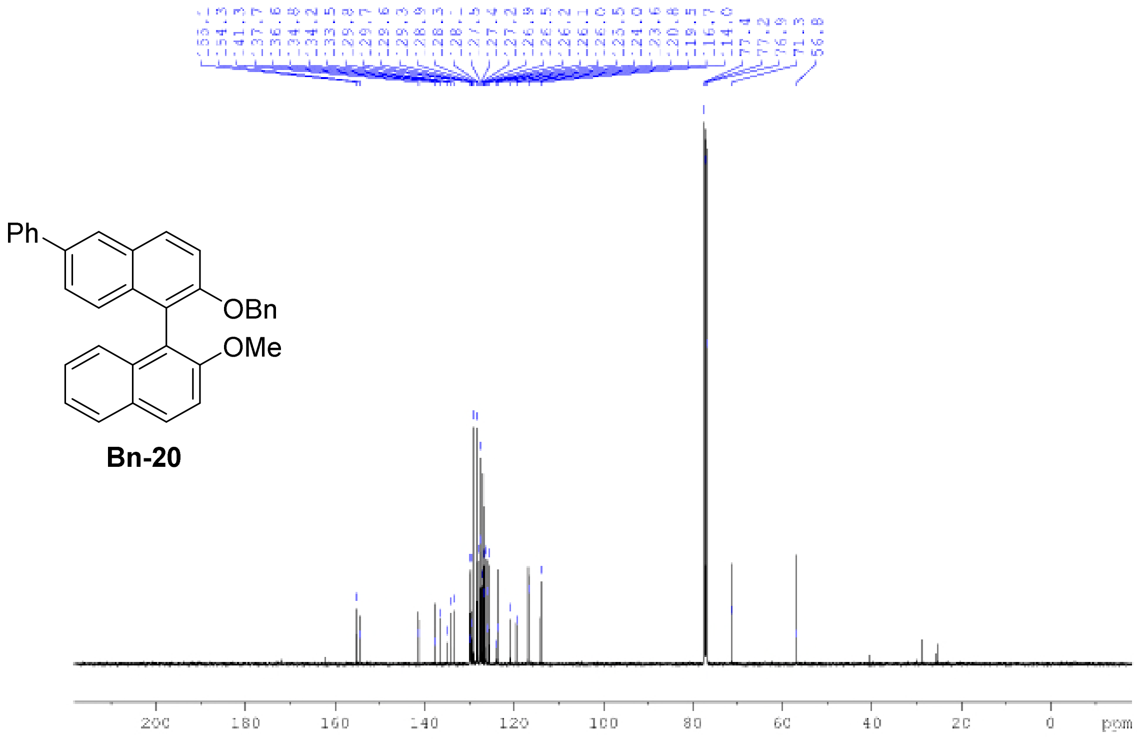

Bn-20

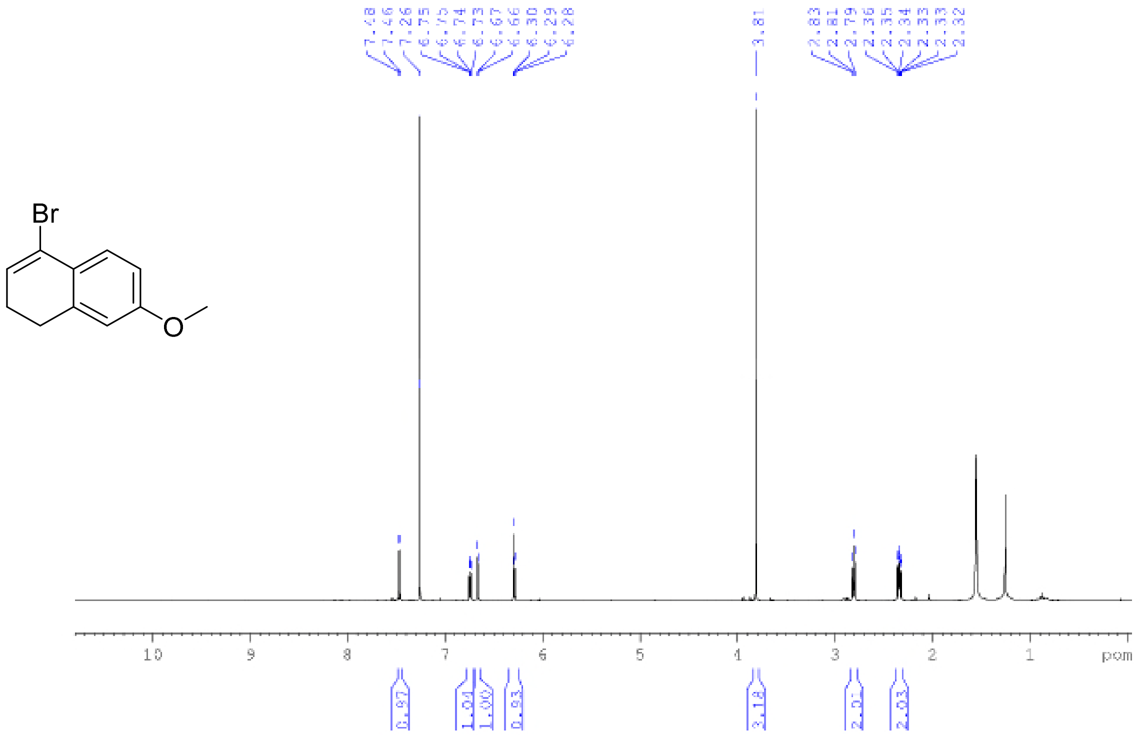
Br
O
7.48
7.46
7.26
6.75
6.75
6.74
6.73
6.67
6.66
6.30
6.29
6.28
3.81
2.83
2.81
2.79
2.36
2.35
2.34
2.33
2.33
2.32
0.97
1.04
1.00
0.93
3.18
2.03
10 9 8 7 6 5 4 3 2 1 ppm

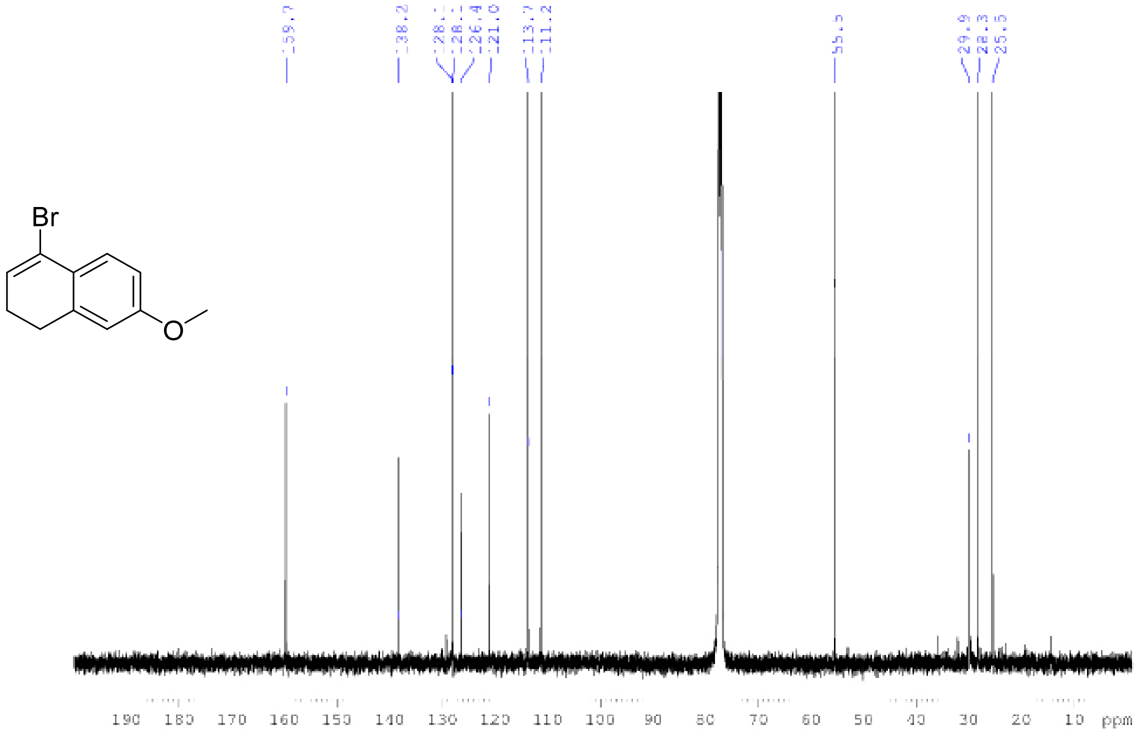
Br
O
159.7
138.2
126.4
121.0
113.7
111.2
55.5
29.9
28.3
25.5
190 180 170 160 150 140 130 120 110 100 90 80 70 60 50 40 30 20 10 ppm

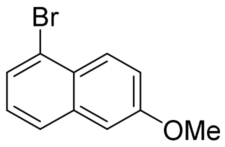
Br
OMe

57

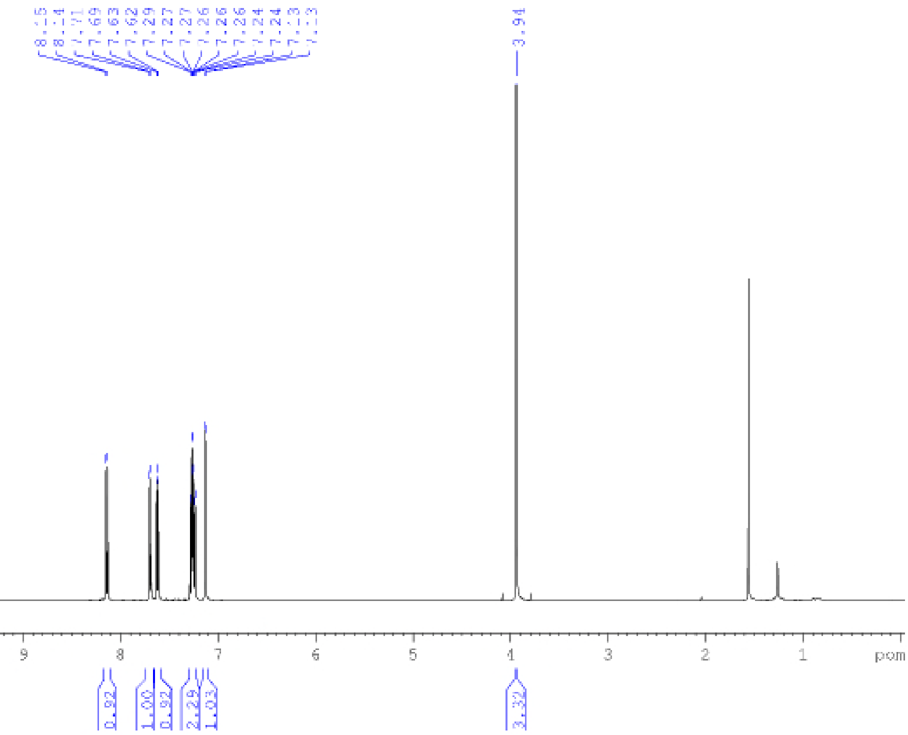
8.15
8.14
7.71
7.69
7.63
7.62
7.29
7.27
7.27
7.26
7.26
7.26
7.24
7.24
3.94
0.92
1.00
0.92
2.29
1.03
3.32
ppm

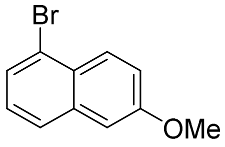
Br
OMe

57

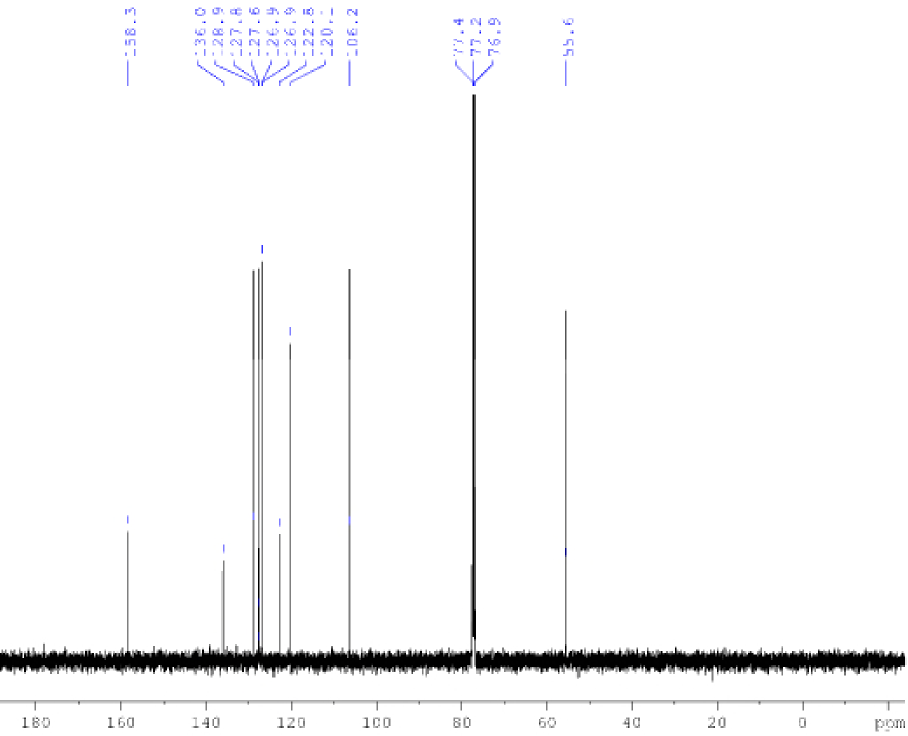
158.3
136.0
128.9
127.8
127.6
126.9
126.9
122.8
106.2
77.4
77.2
76.9
55.6
ppm

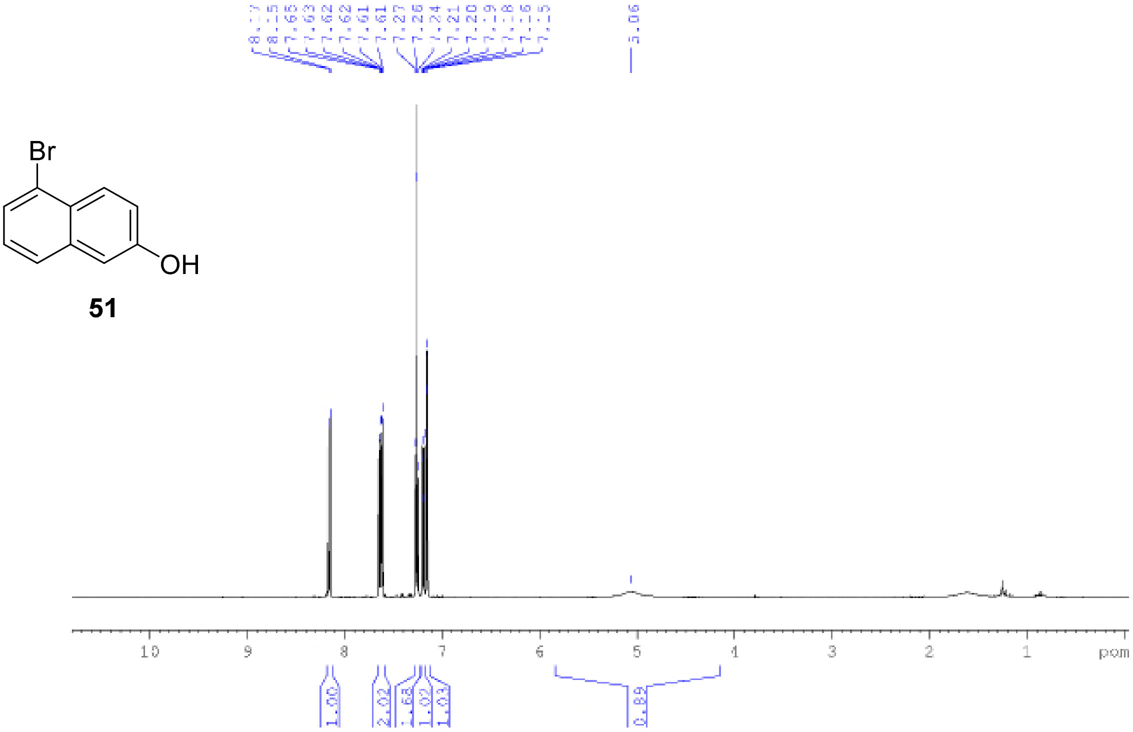

51

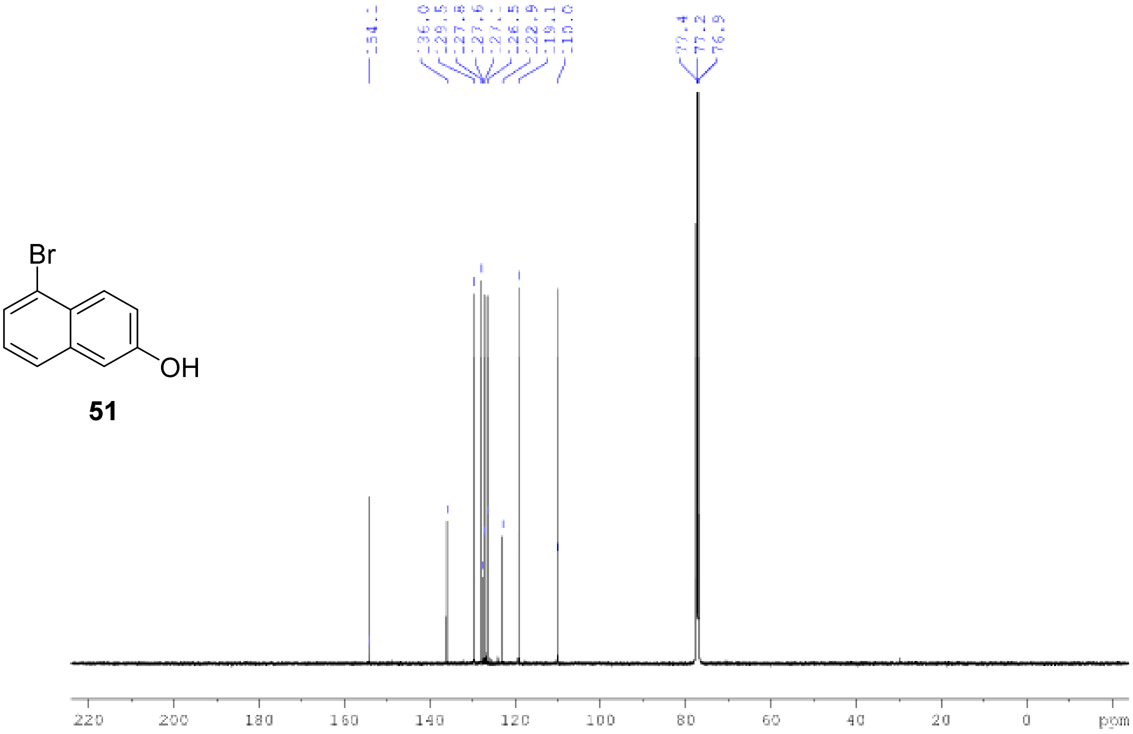

51

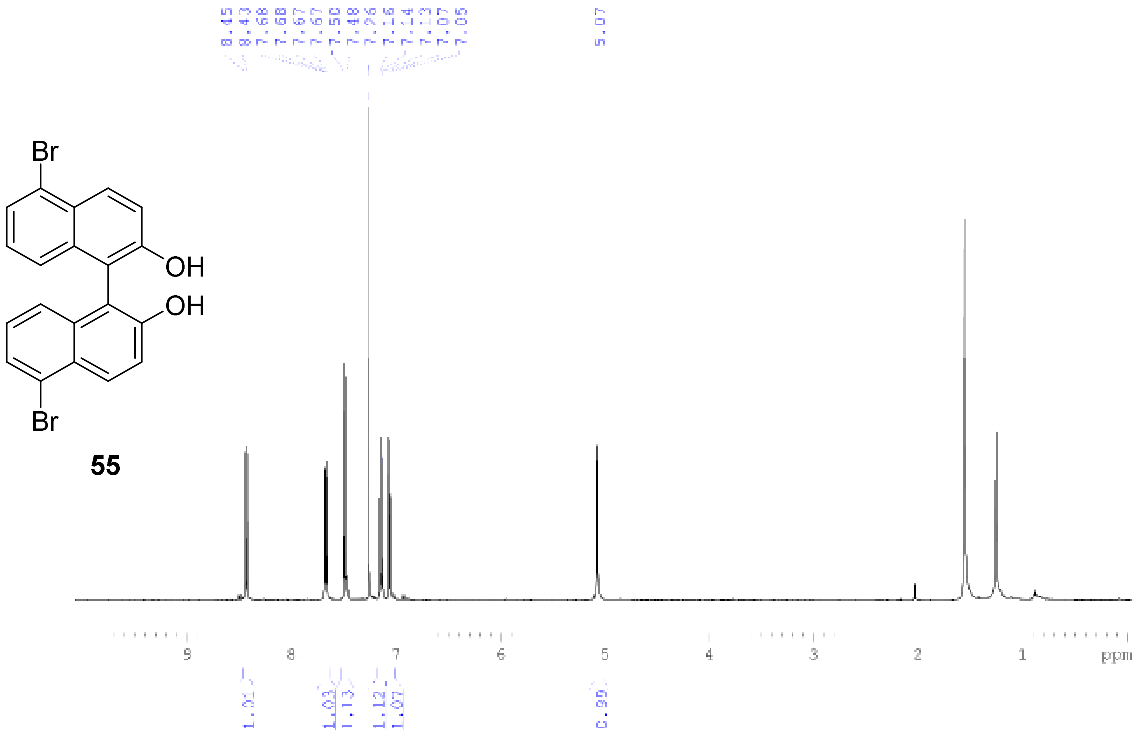
Br
OH
OH
Br
8.45
8.43
7.68
7.68
7.67
7.67
7.50
7.48
7.26
7.16
7.14
7.13
7.07
7.05
5.07
9
8
7
6
5
4
3
2
1
ppm
1.02
1.03
1.13
1.12
1.07
0.99

55

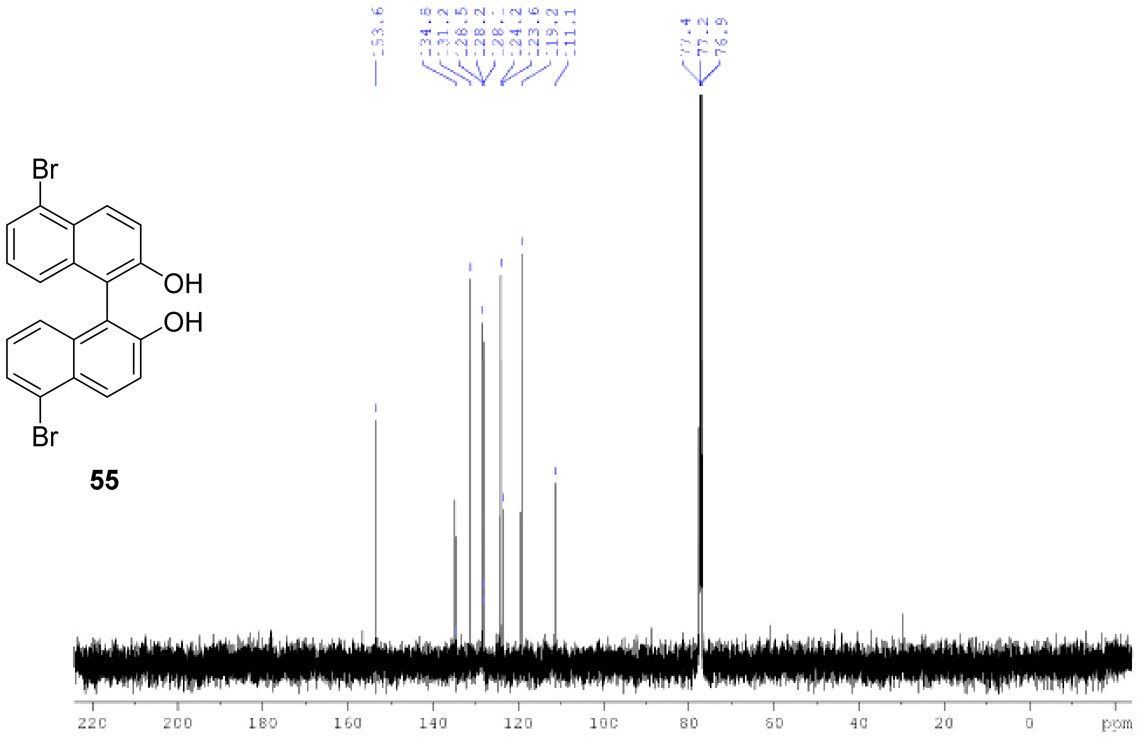
Br
OH
OH
Br
153.6
134.8
131.2
128.5
128.2
128.1
124.2
123.6
119.2
111.1
77.4
77.2
76.9
220
200
180
160
140
120
100
80
60
40
20
0
ppm

55

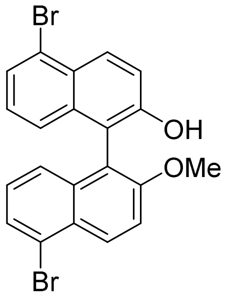

38

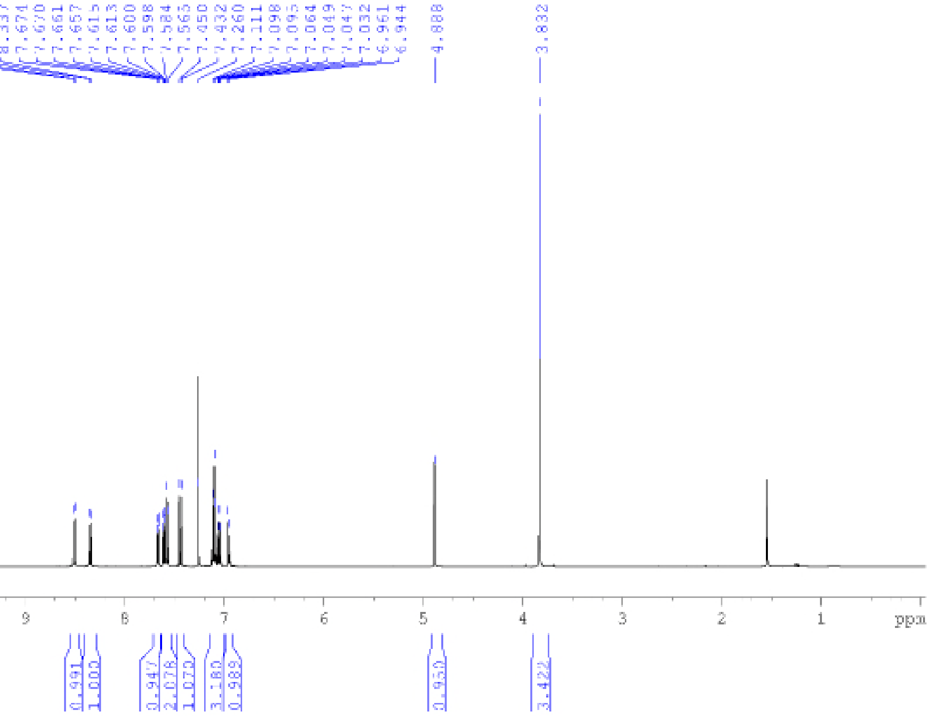

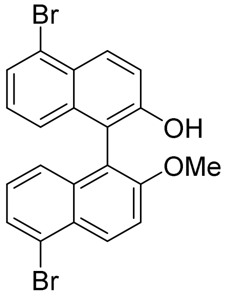

38

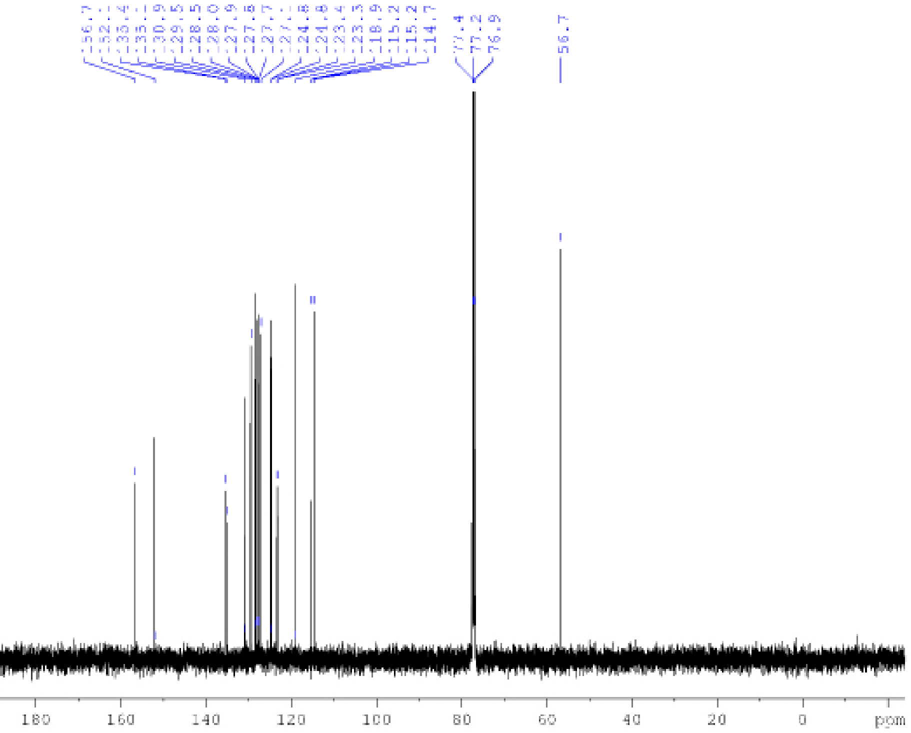

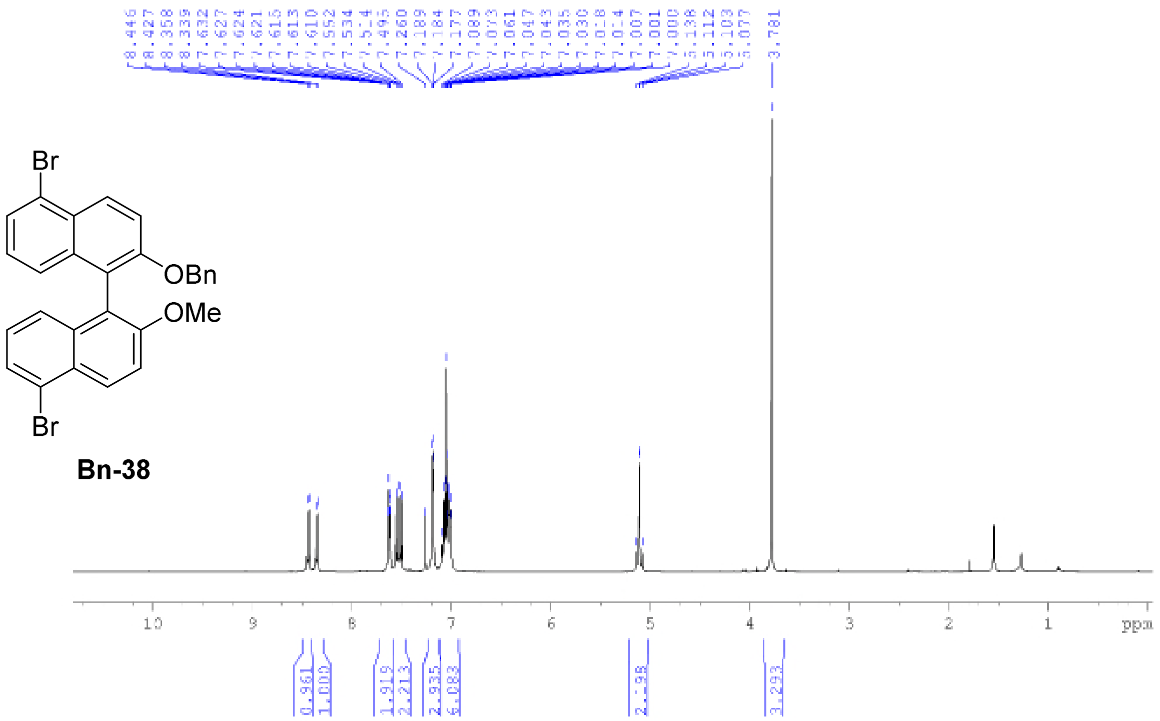
Br
OBn
OMe
Br
Bn-38
ppm

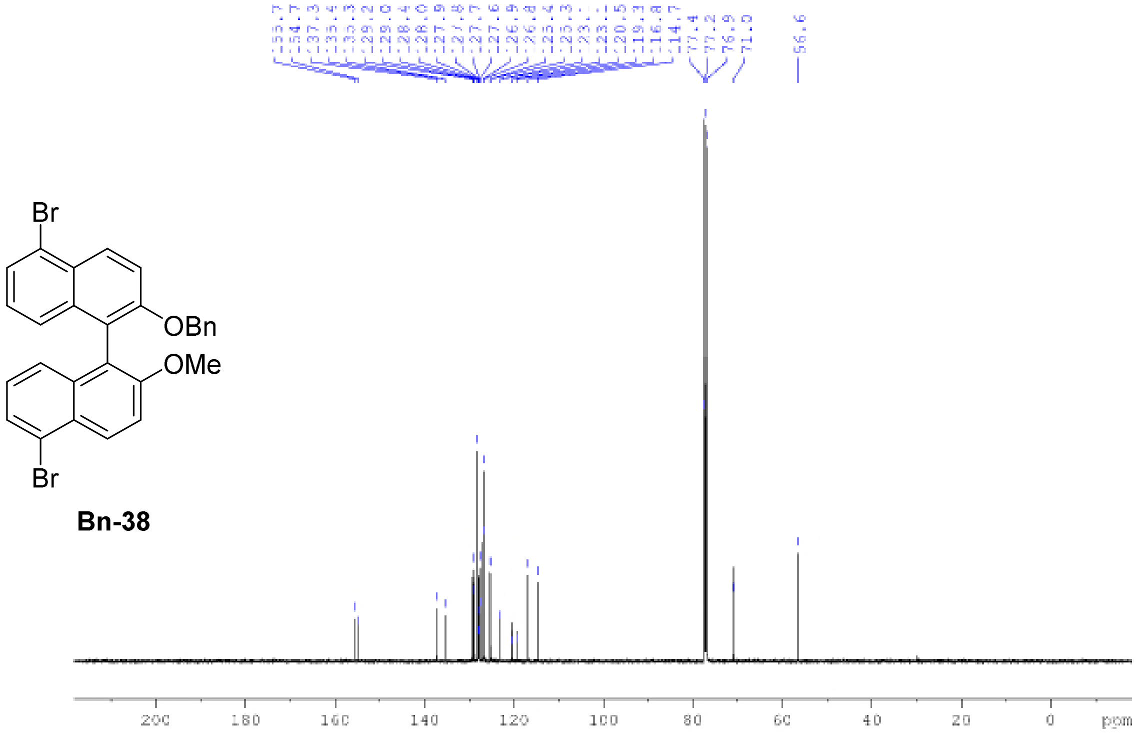
Br
OBn
OMe
Br
Bn-38
ppm

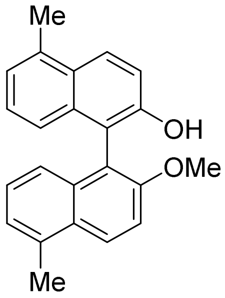
Me
OH
OMe
Me

32

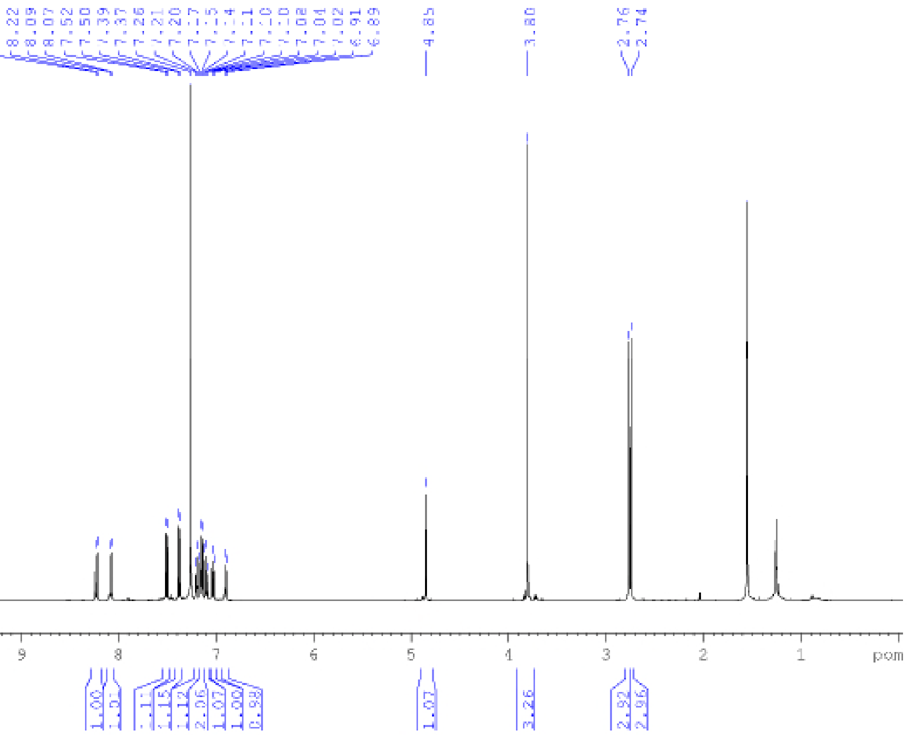
4.85
3.80
2.76
2.74
ppm

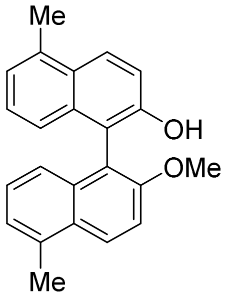
Me
OH
OMe
Me

32

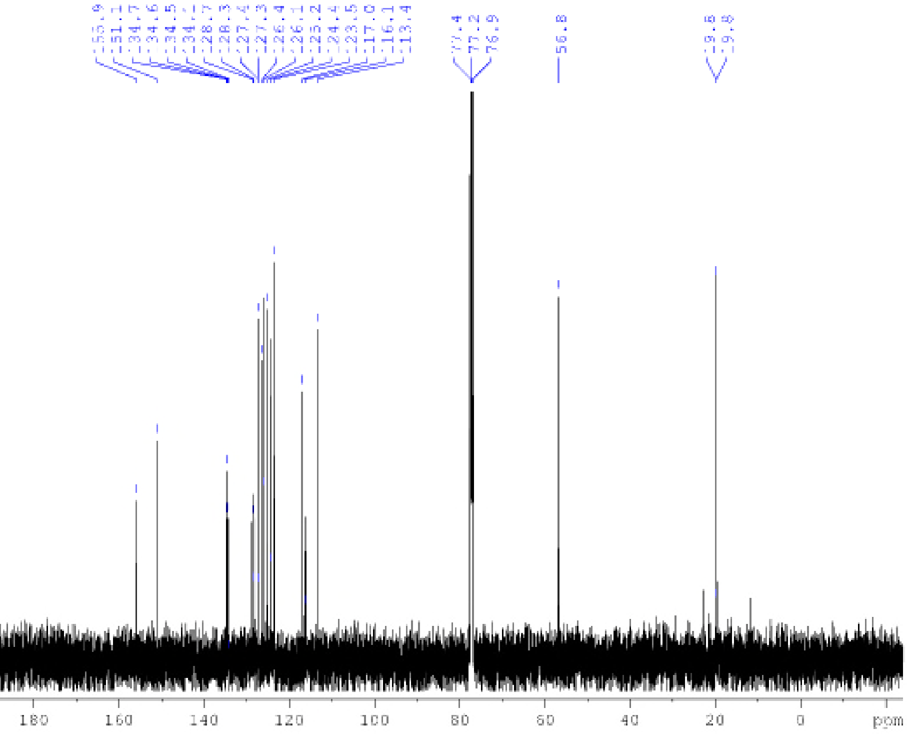
77.4
77.2
76.9
56.8
19.8
19.8
ppm

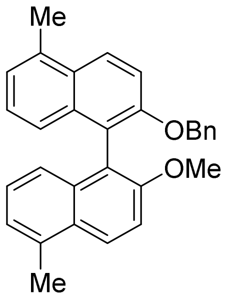

Bn-32

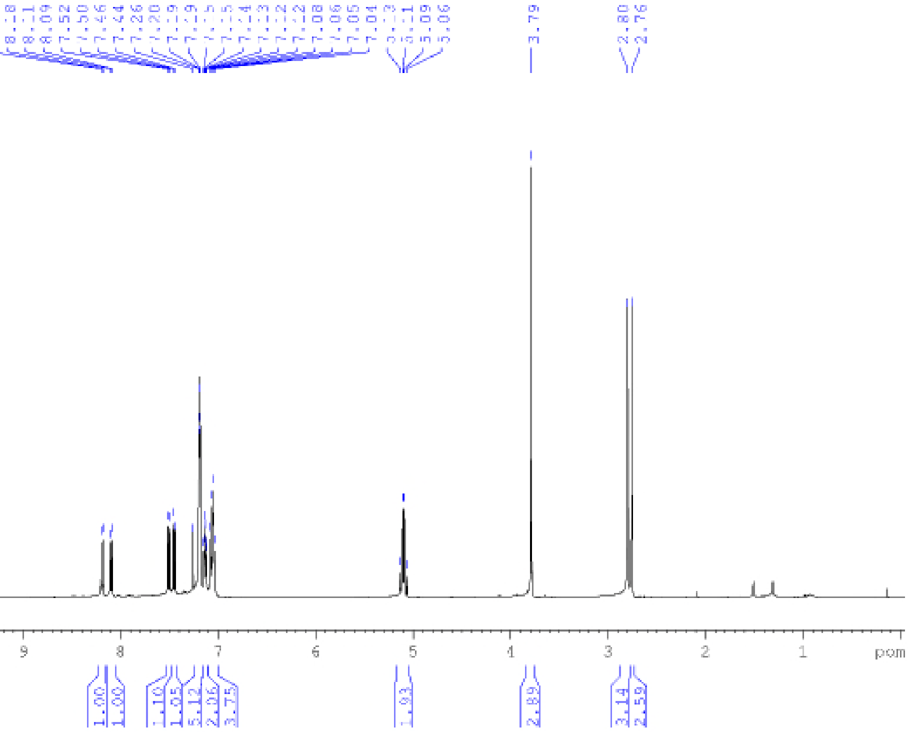

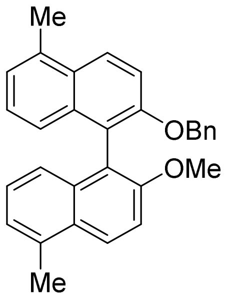

Bn-32

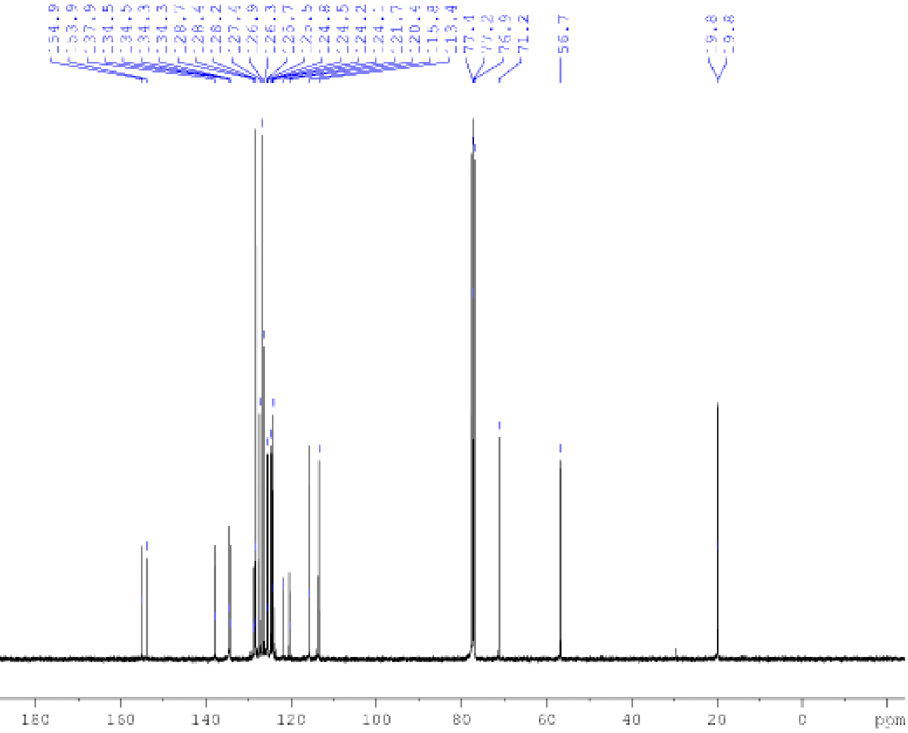

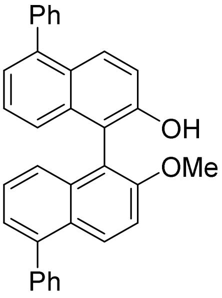

34

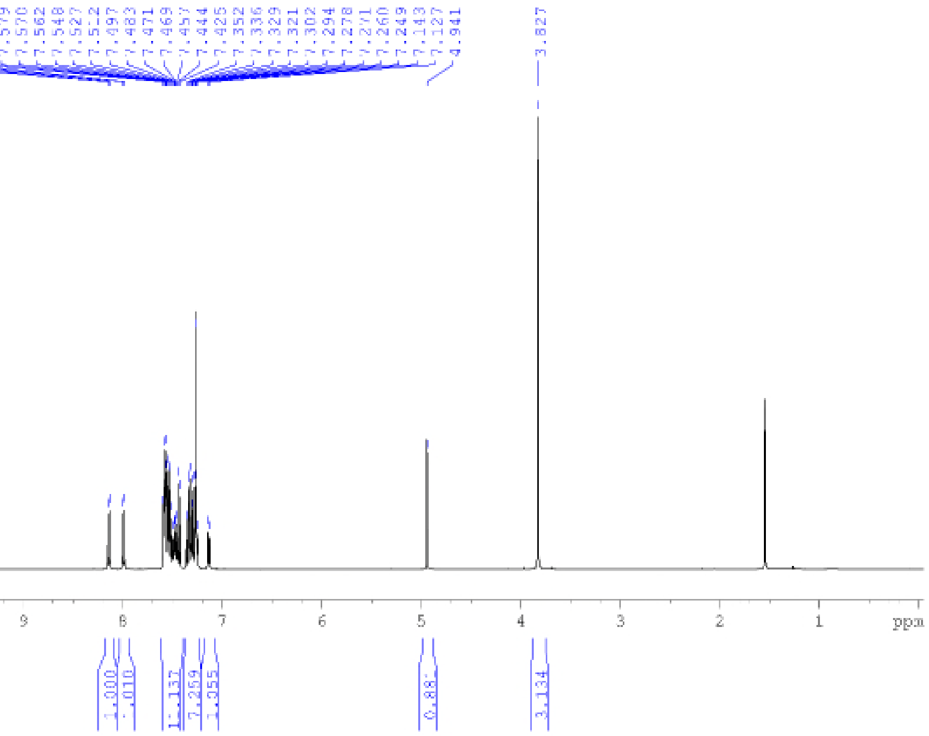

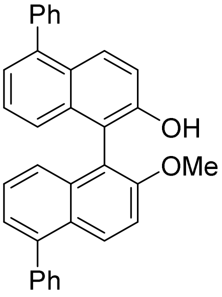

34

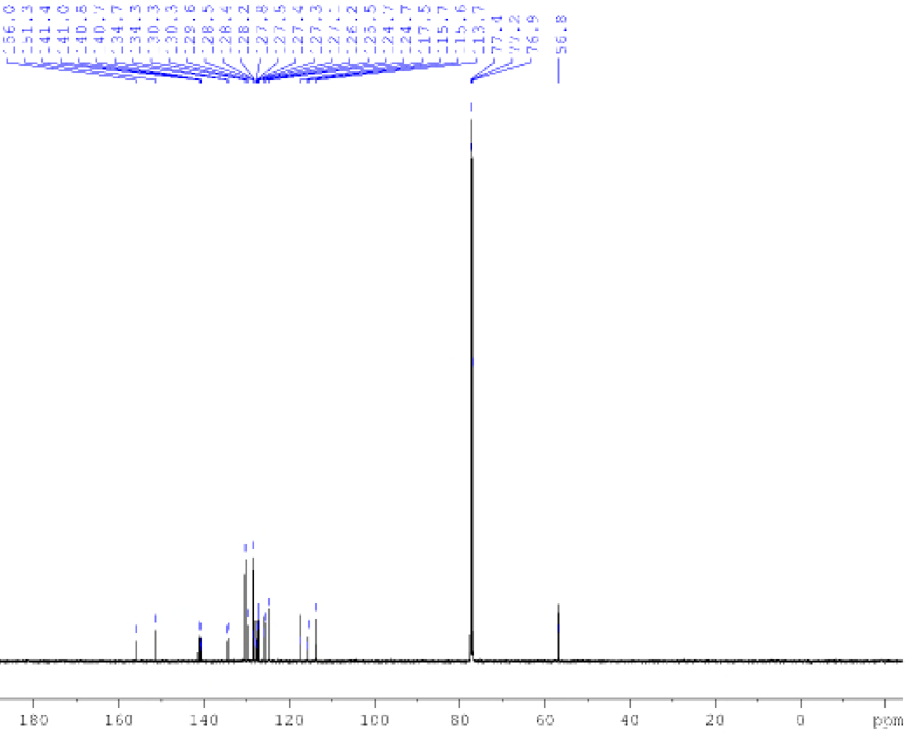

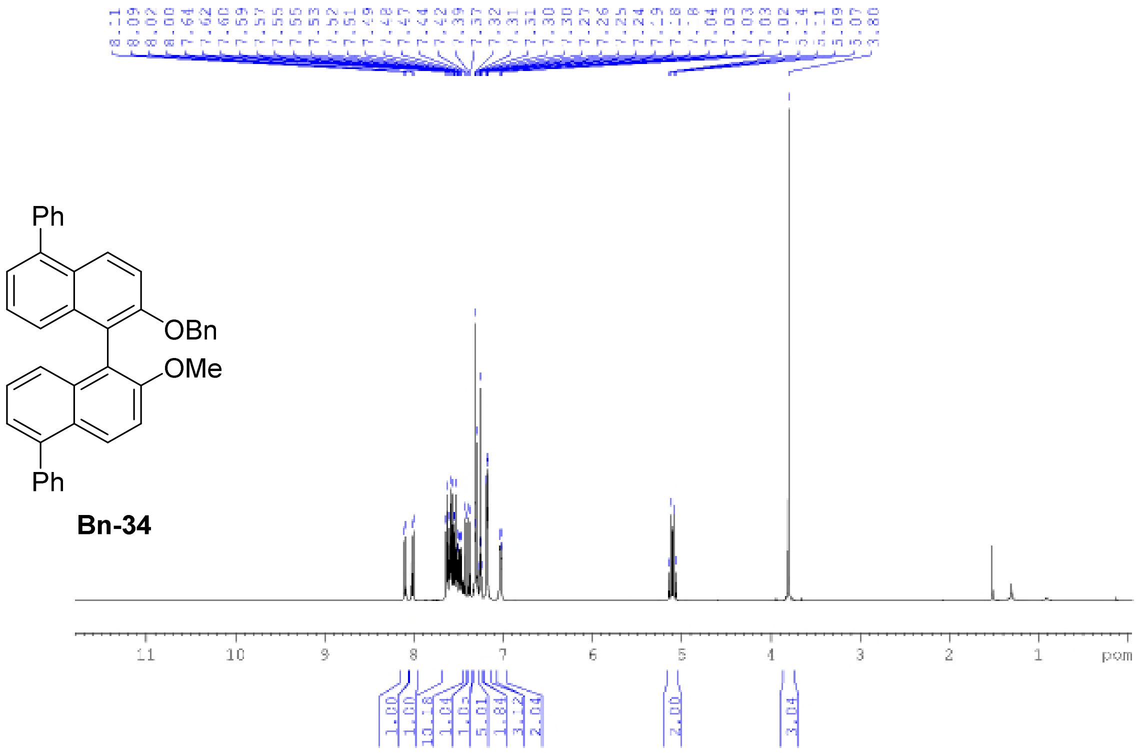
Ph
OBn
OMe
Ph
Bn-34
ppm

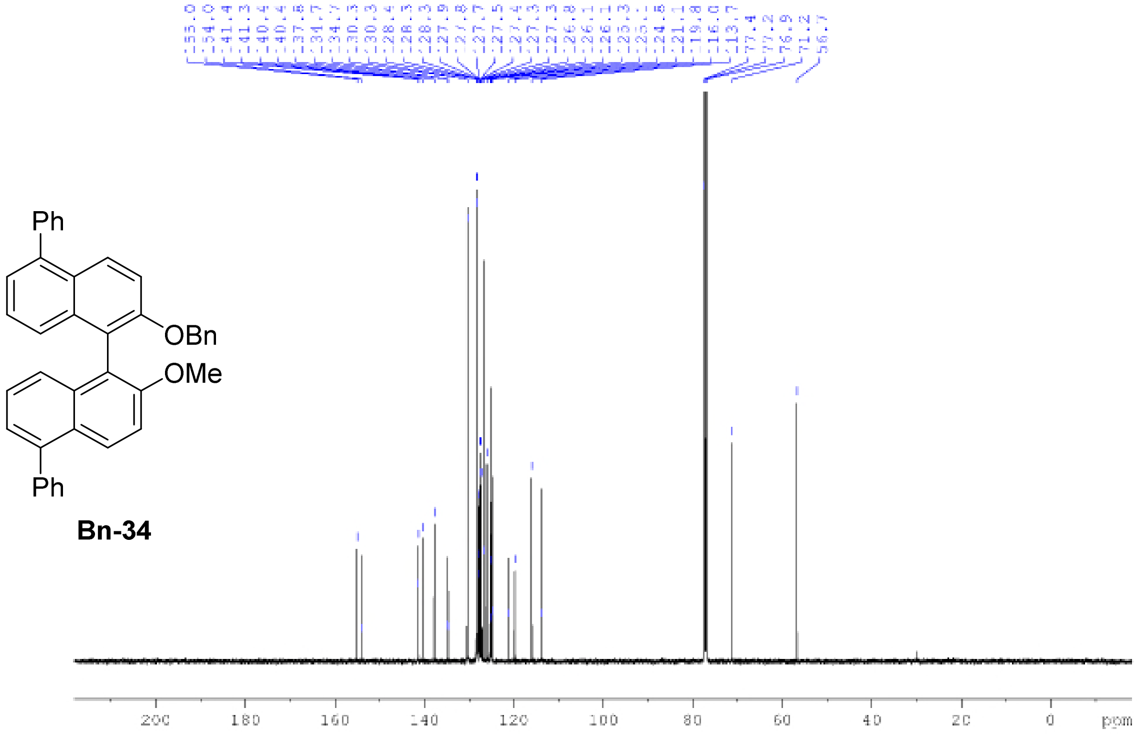
Ph
OBn
OMe
Ph
Bn-34
ppm

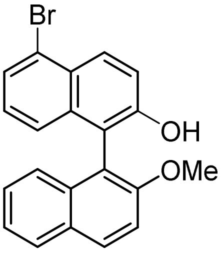

Br
OH
OMe

24

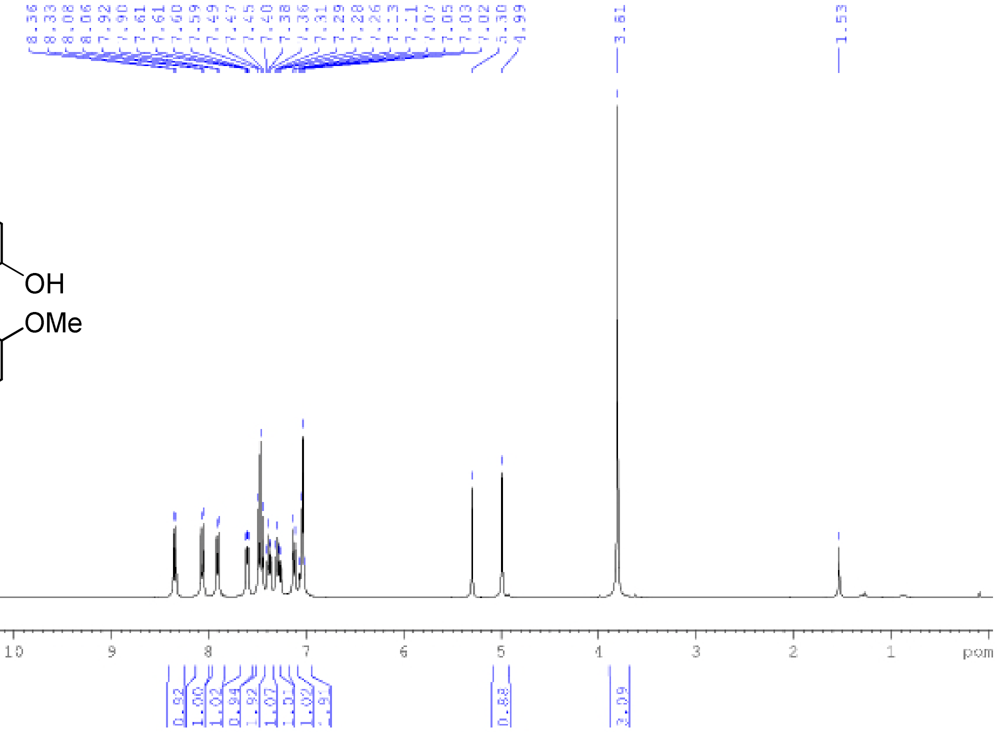

8.36 8.33 8.08 8.06 7.92 7.90 7.61 7.61 7.60 7.59 7.49 7.47 7.45 7.40 7.38 7.36 7.31 7.29 7.28 7.26 7.13 7.11 7.07 7.05 7.03 7.02 5.30 4.99
3.81
1.53
ppm

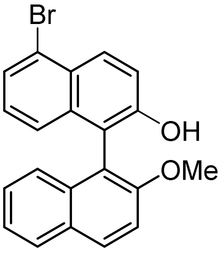

Br
OH
OMe

24

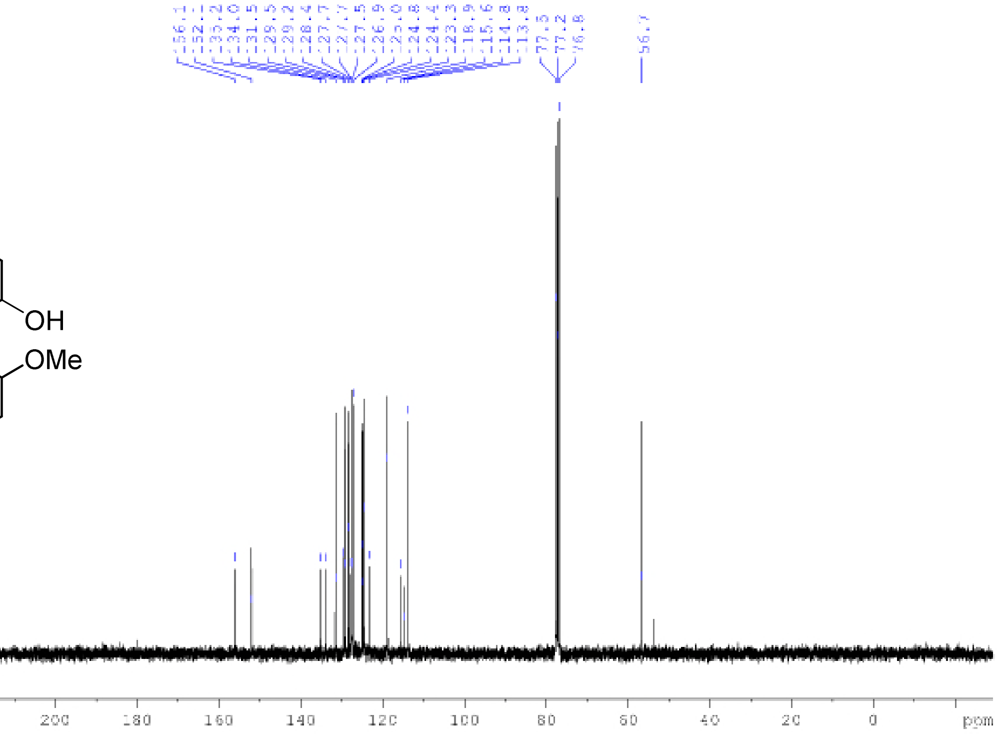

77.5 77.2 76.8
56.7
ppm

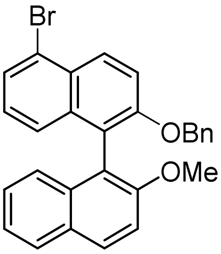

Bn-24

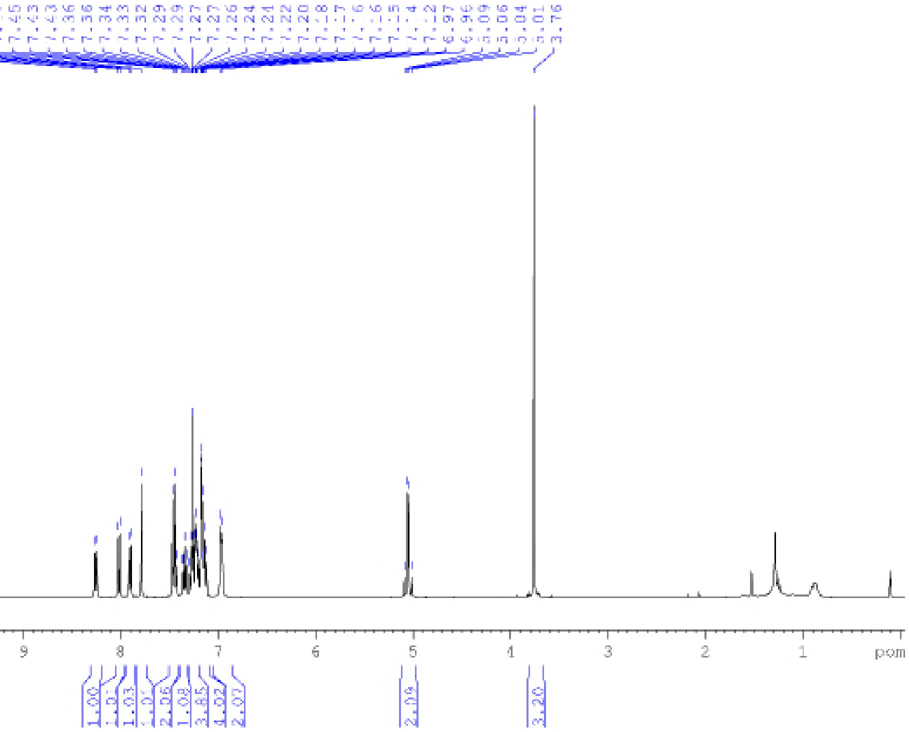

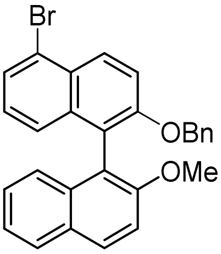

Bn-24

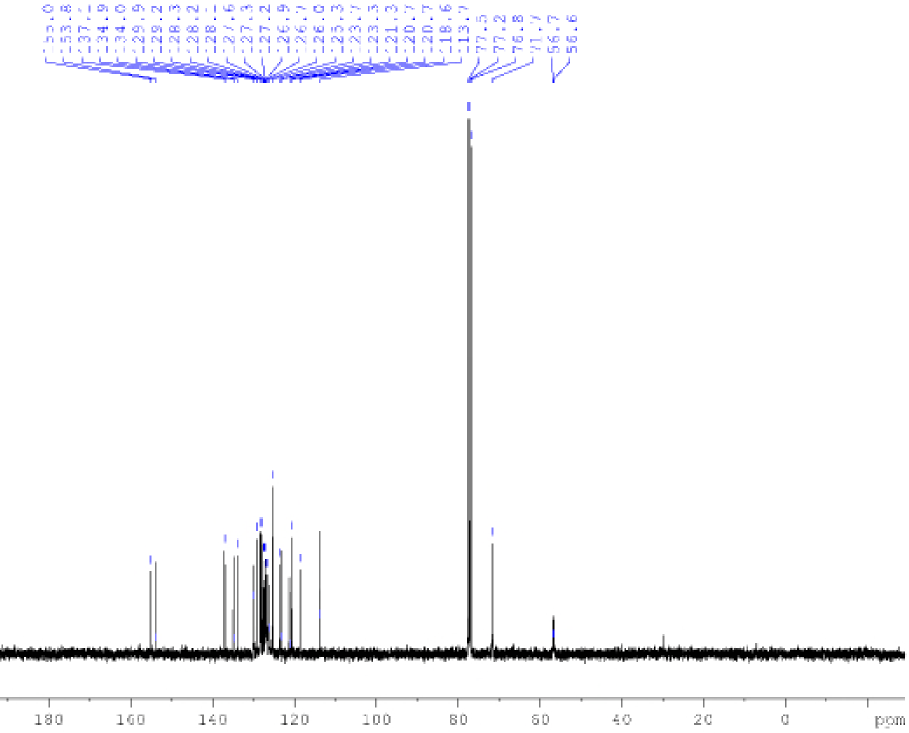

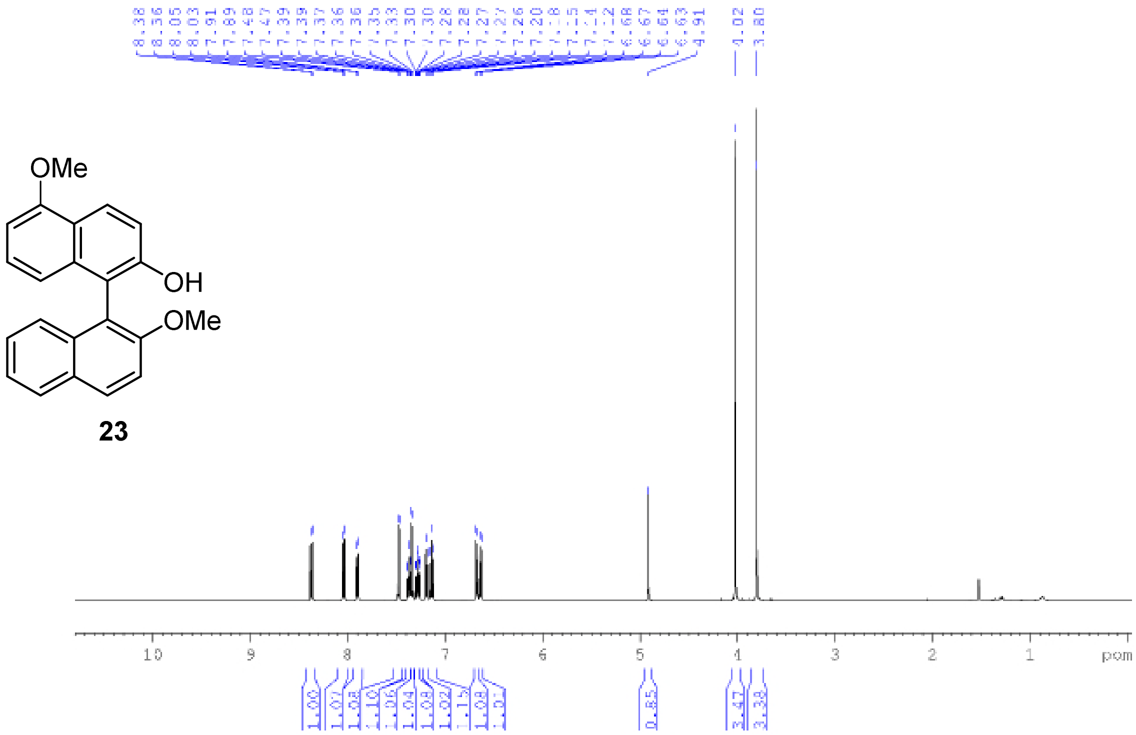

23

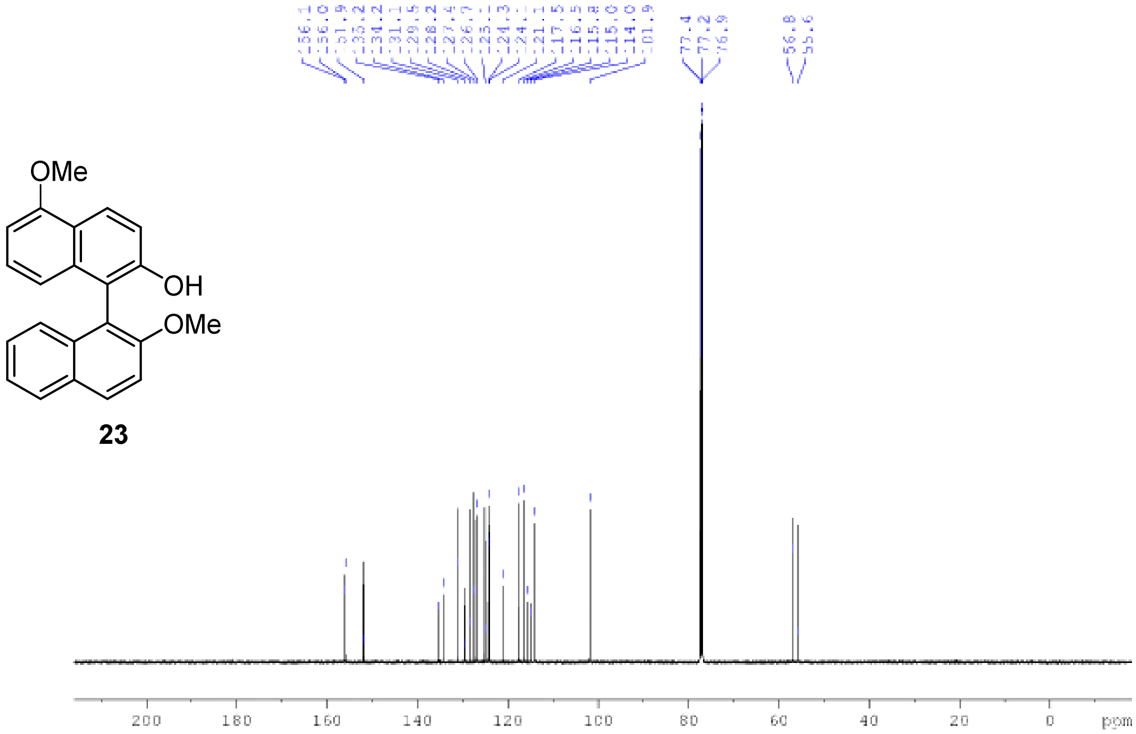

23

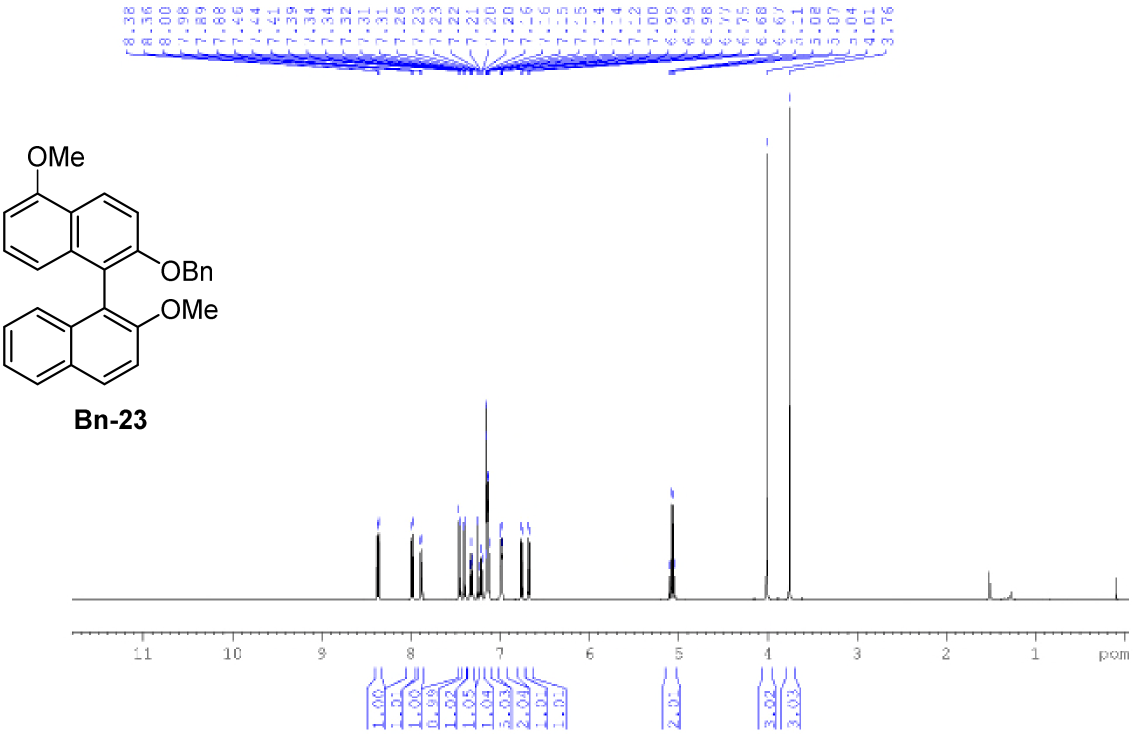

Bn-23

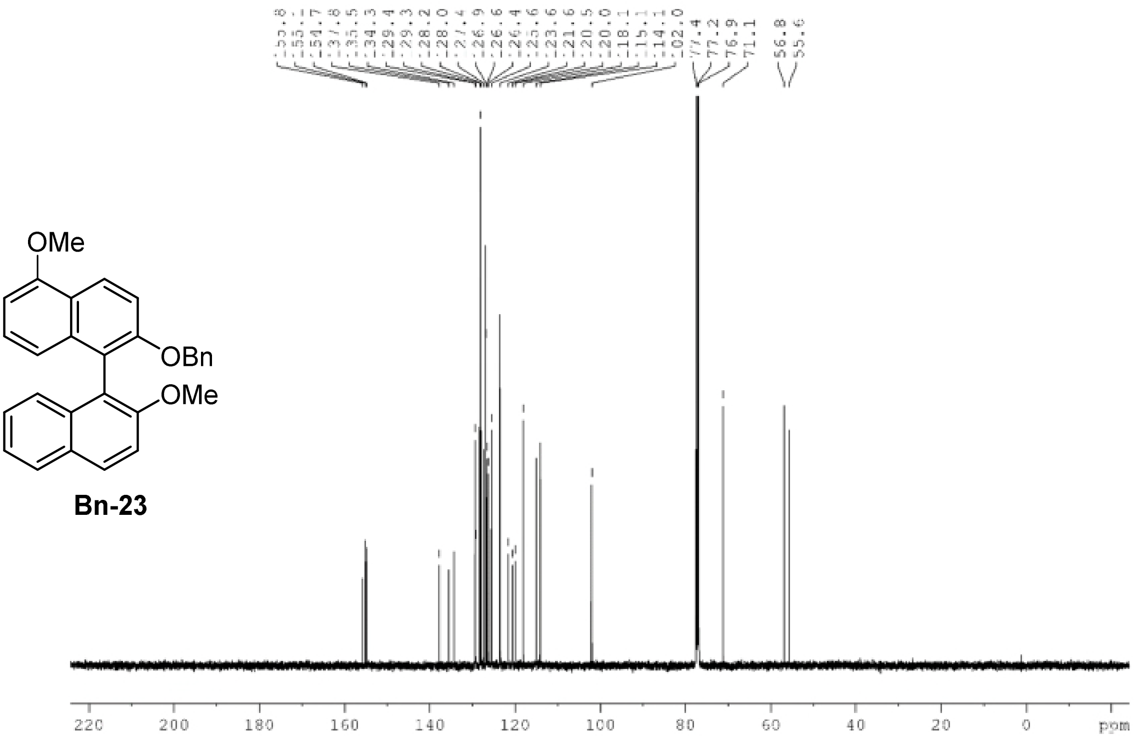

Bn-23

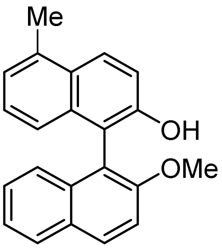

Me
OH
OMe

22

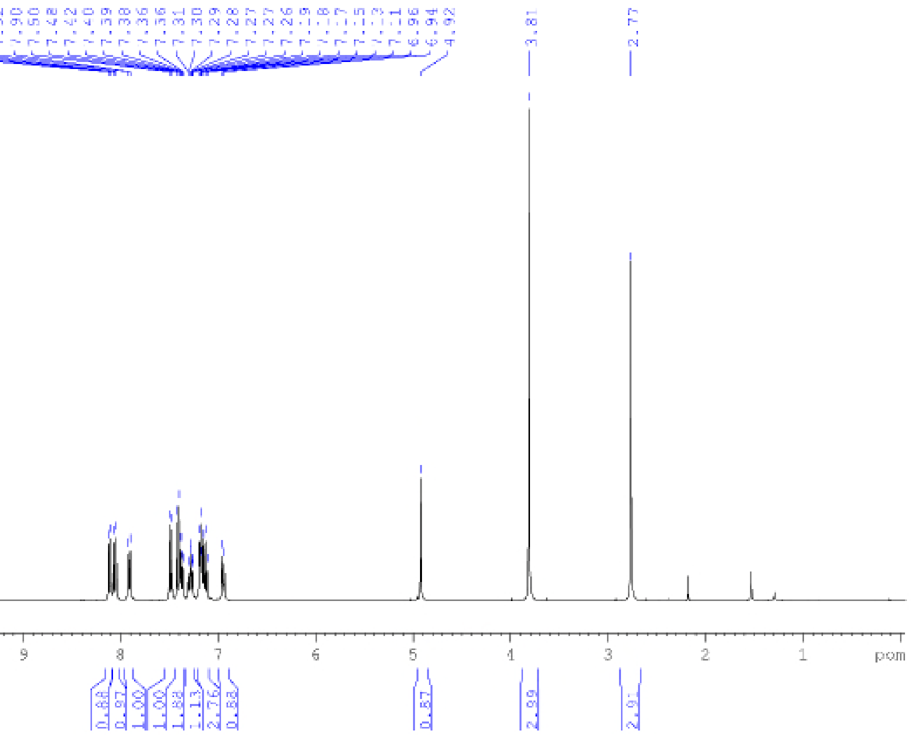

3.81
2.77
10
9
8
7
6
5
4
3
2
1
ppm
0.87
2.99

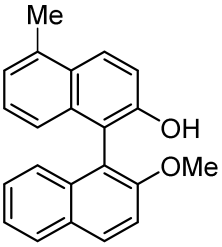

Me
OH
OMe

22

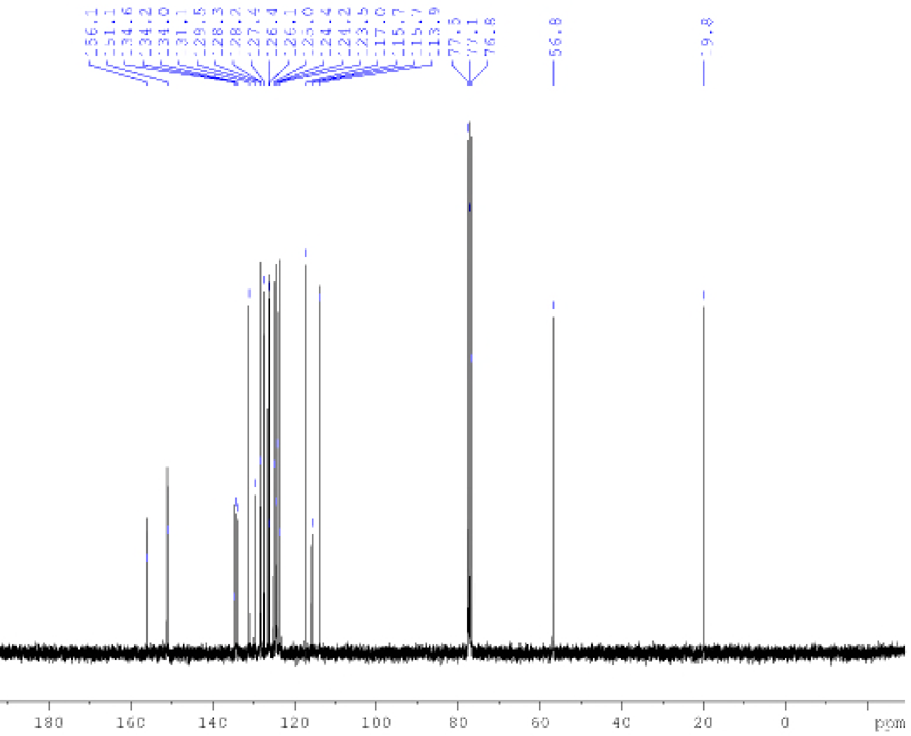

77.5
77.1
76.8
56.8
19.8
220
200
180
160
140
120
100
80
60
40
20
0
ppm

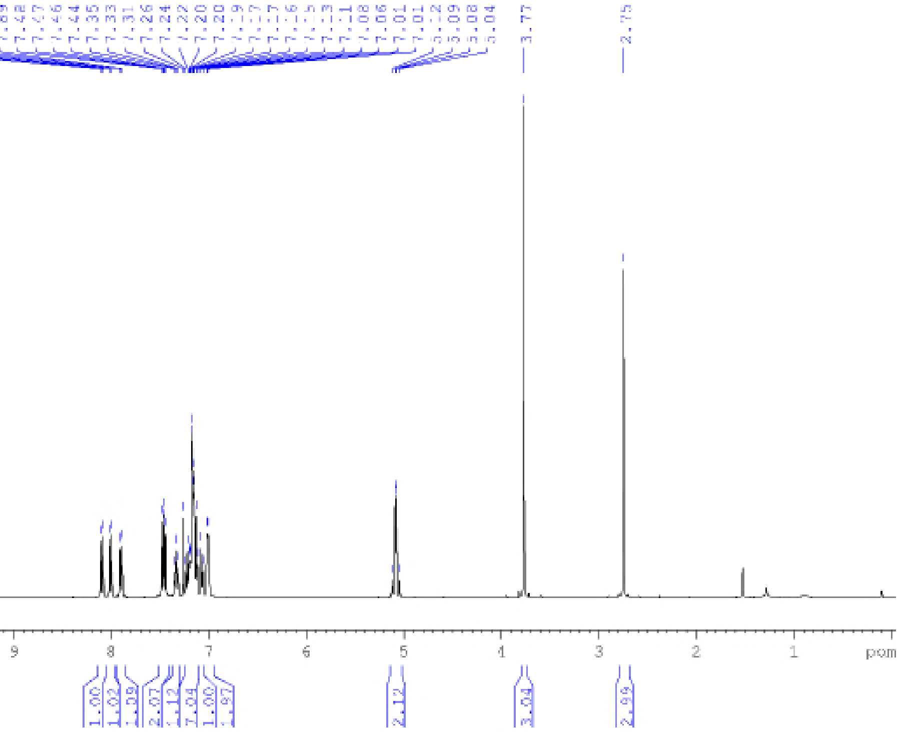

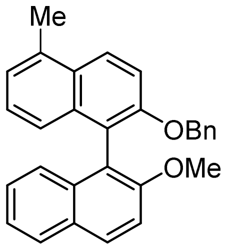

Bn-22

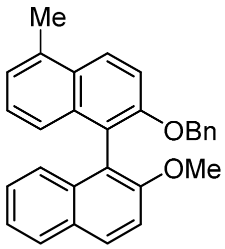

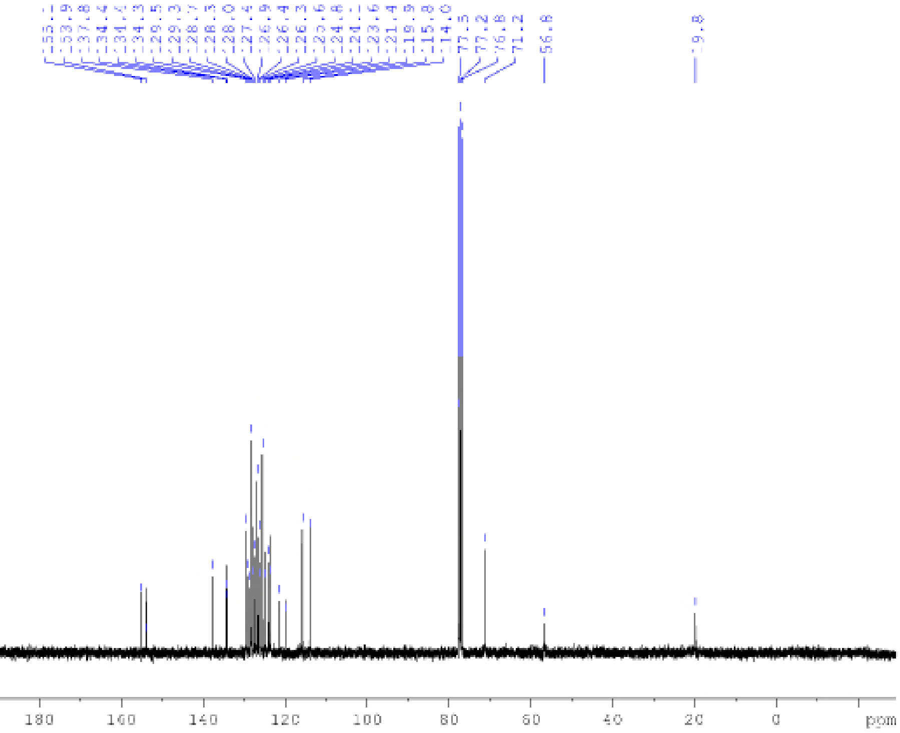

Bn-22

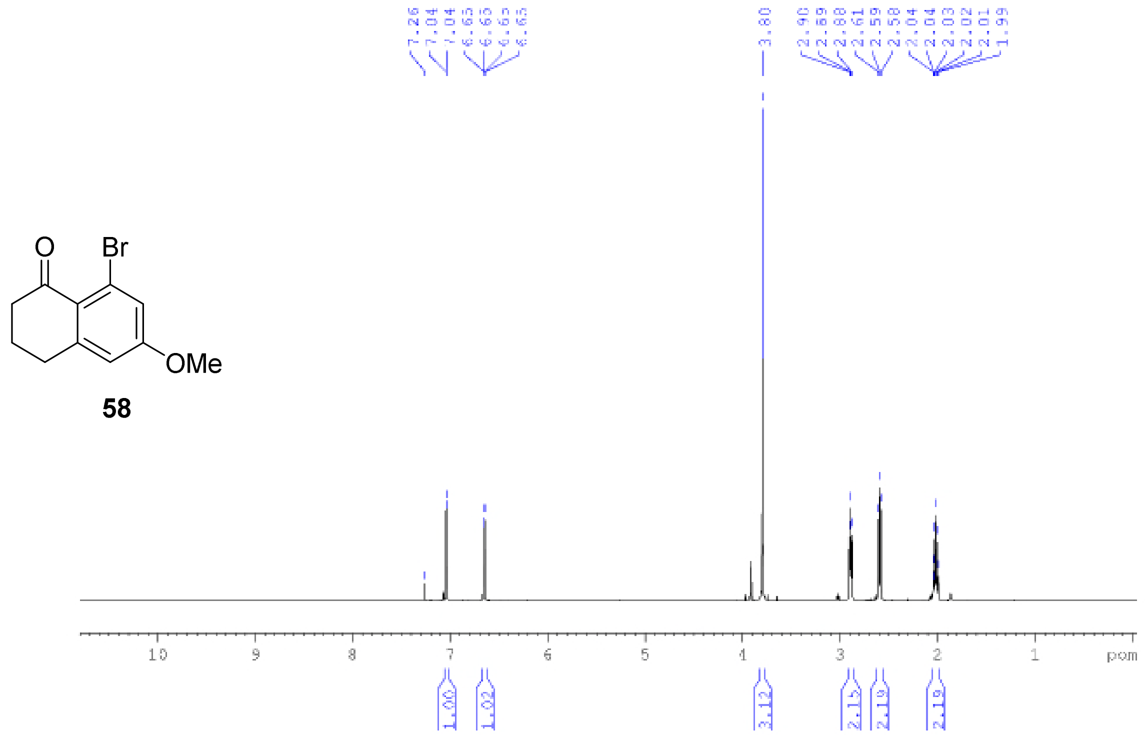
O
Br
OMe
58
7.26
7.04
7.04
6.65
6.65
6.65
6.65
3.80
2.90
2.89
2.88
2.61
2.59
2.58
2.04
2.04
2.03
2.02
2.01
1.99
10
9
8
7
6
5
4
3
2
1
ppm
1.00
1.02
3.12
2.15
2.19
2.19

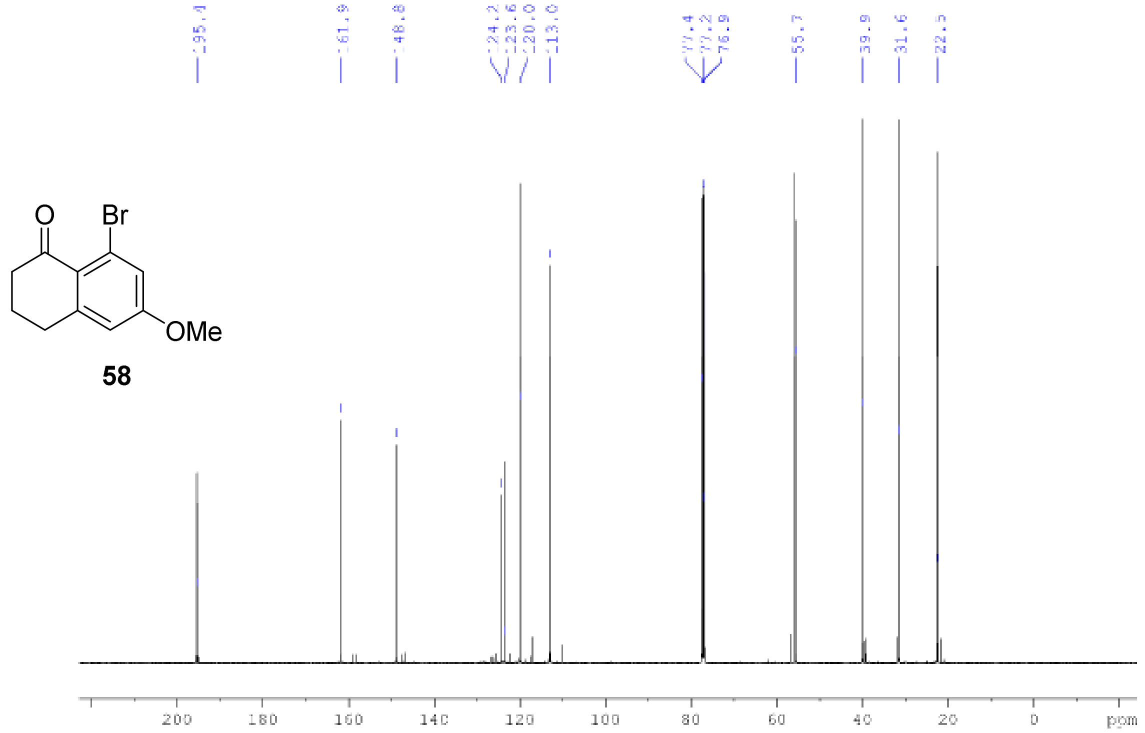
O
Br
OMe
58
195.4
161.9
148.8
124.2
123.6
120.0
113.0
77.4
77.2
76.9
55.7
39.9
31.6
22.5
200
180
160
140
120
100
80
60
40
20
0
ppm

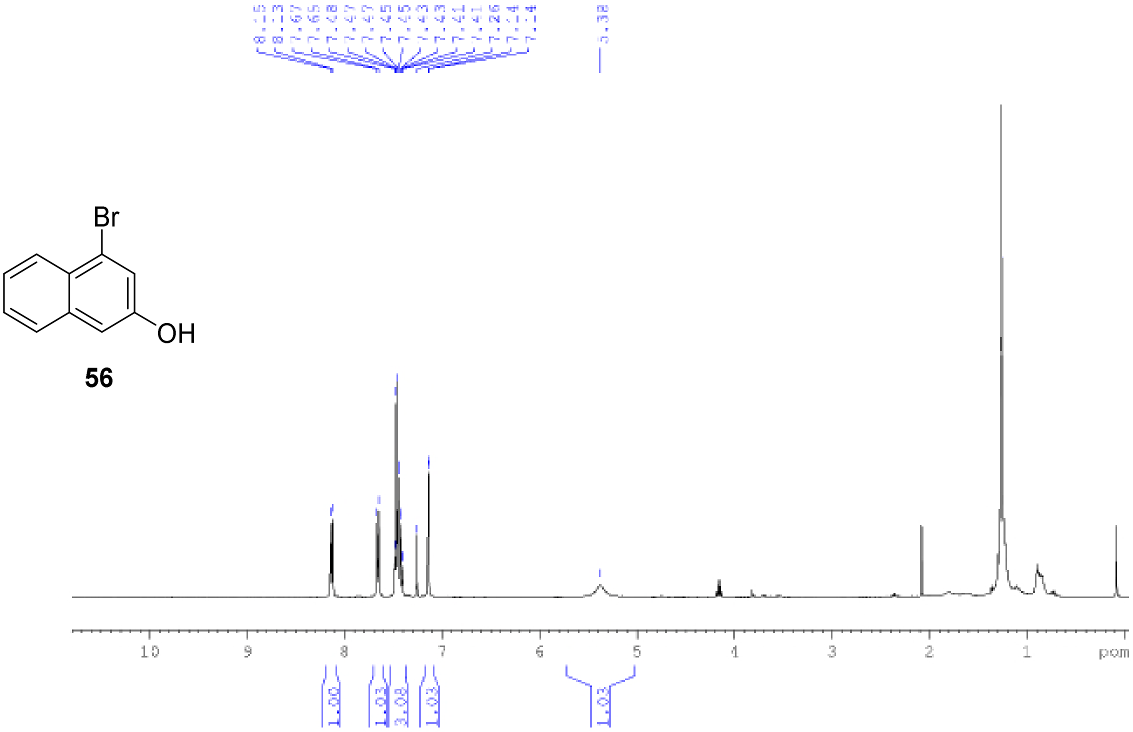
8.15
8.13
7.67
7.65
7.48
7.47
7.47
7.45
7.45
7.43
7.43
7.41
7.41
7.26
7.14
7.14
5.38
1.00
1.03
3.08
1.03
1.03
ppm
Br
OH

56

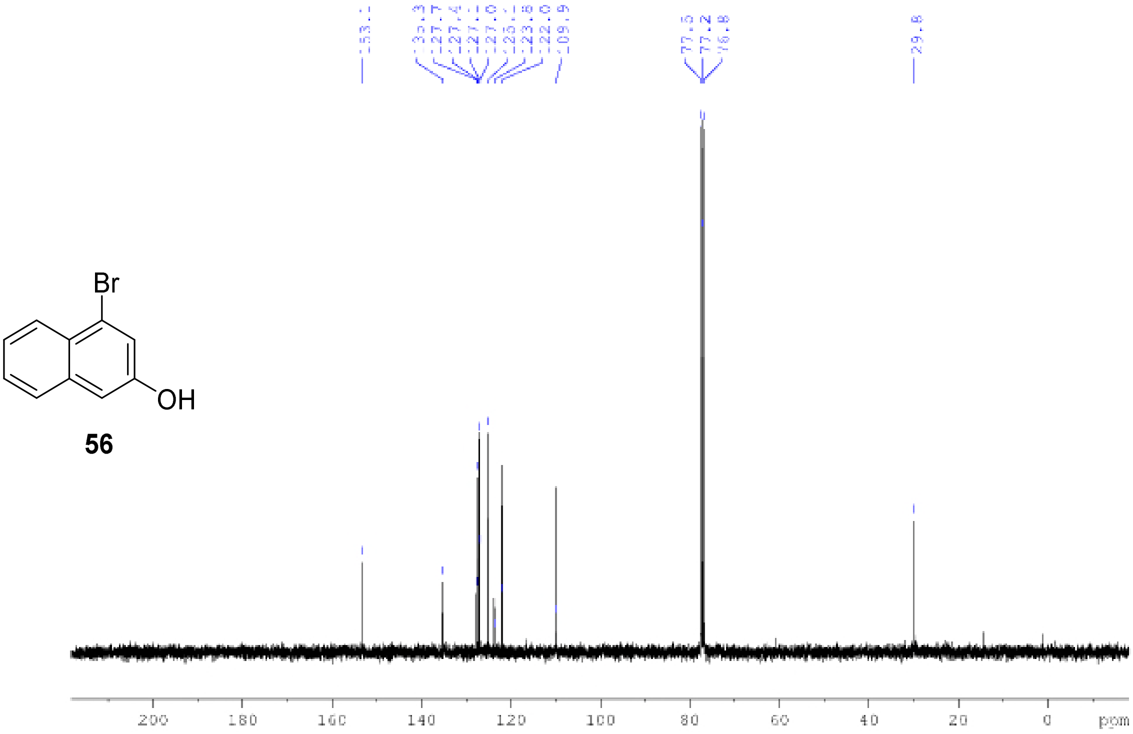
153.1
135.3
127.7
127.4
127.1
127.0
125.1
123.8
122.0
109.9
77.5
77.2
76.8
29.8
ppm
Br
OH

56

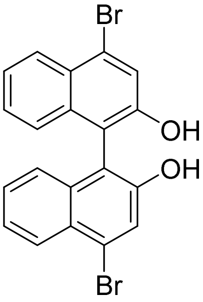

41

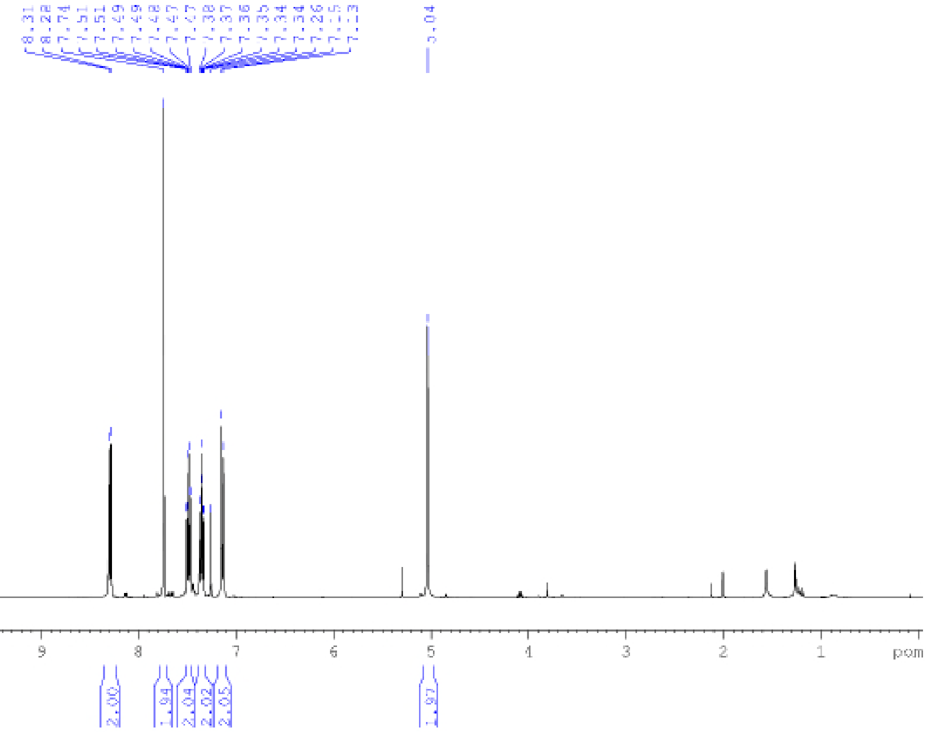

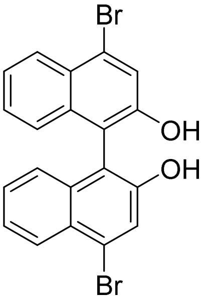

41

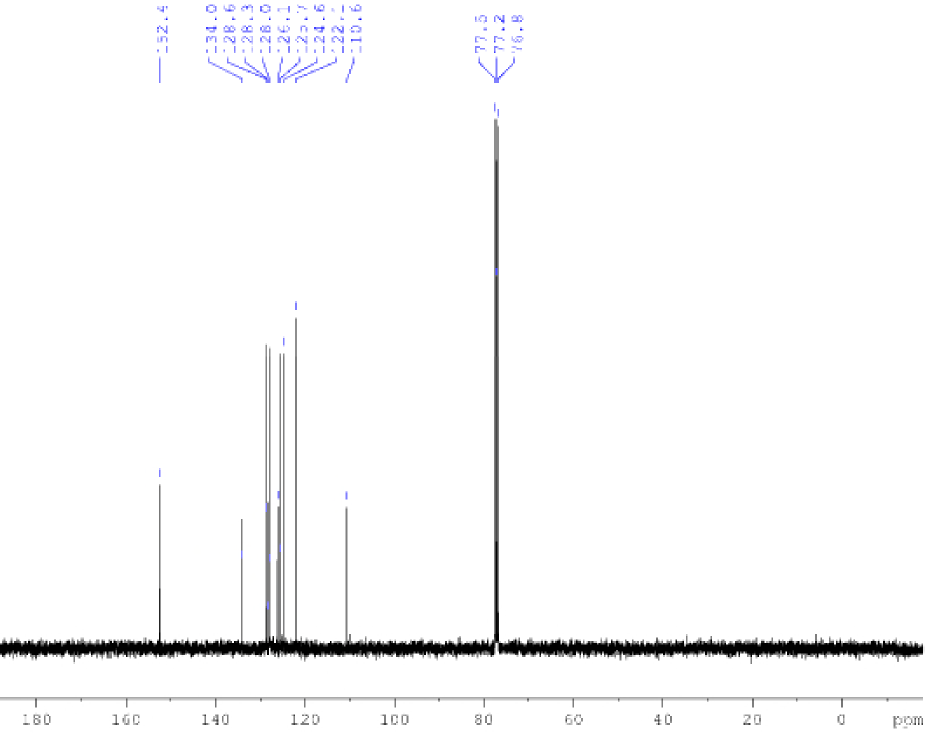

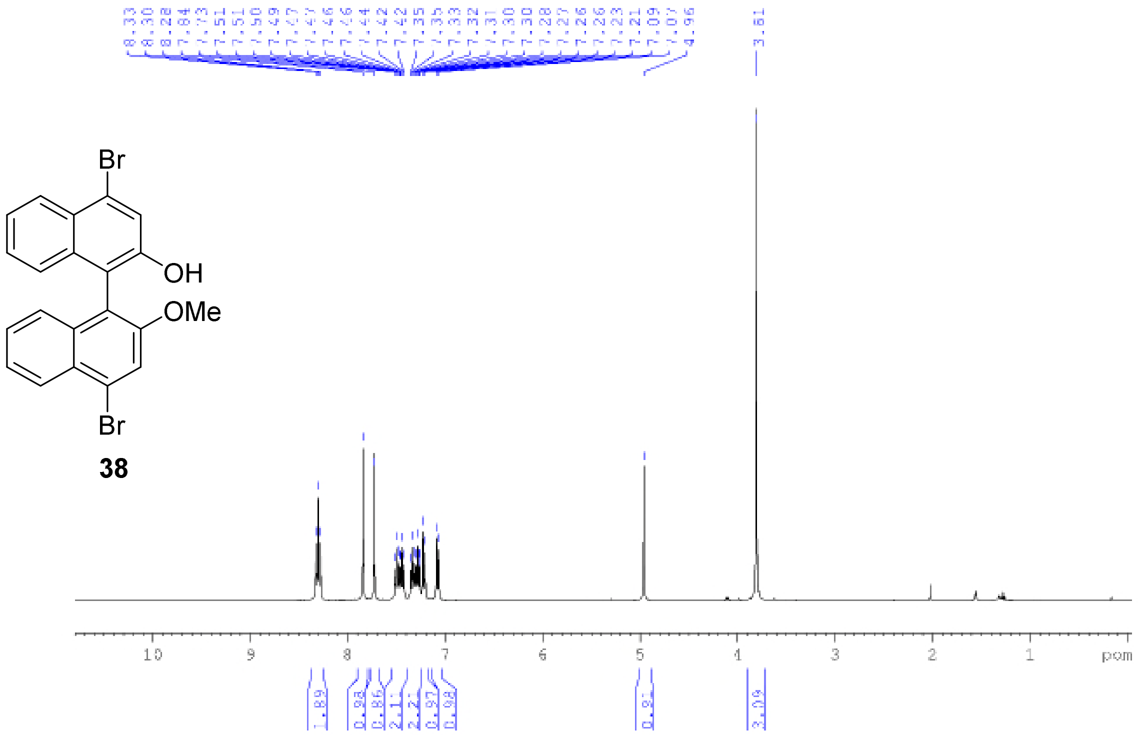

38

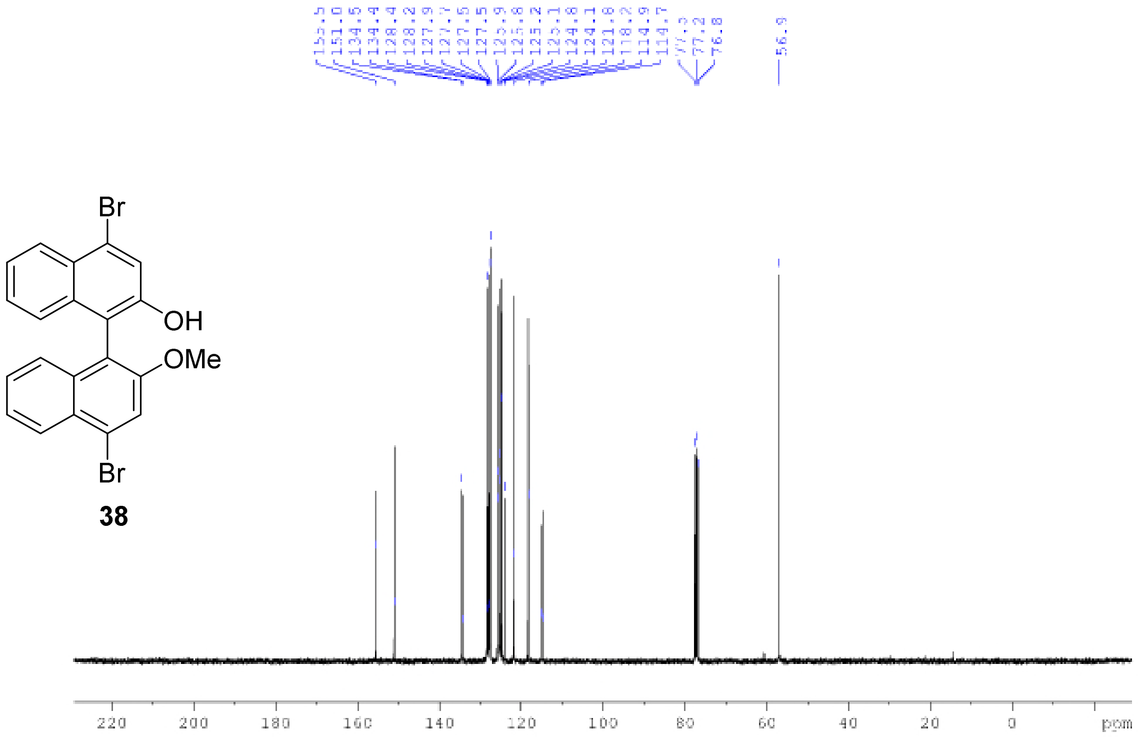

38

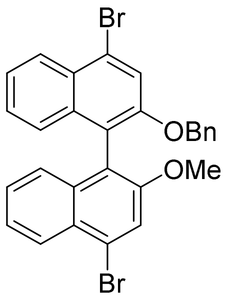

Bn-38

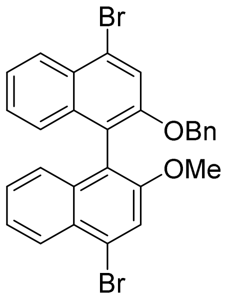

Bn-38

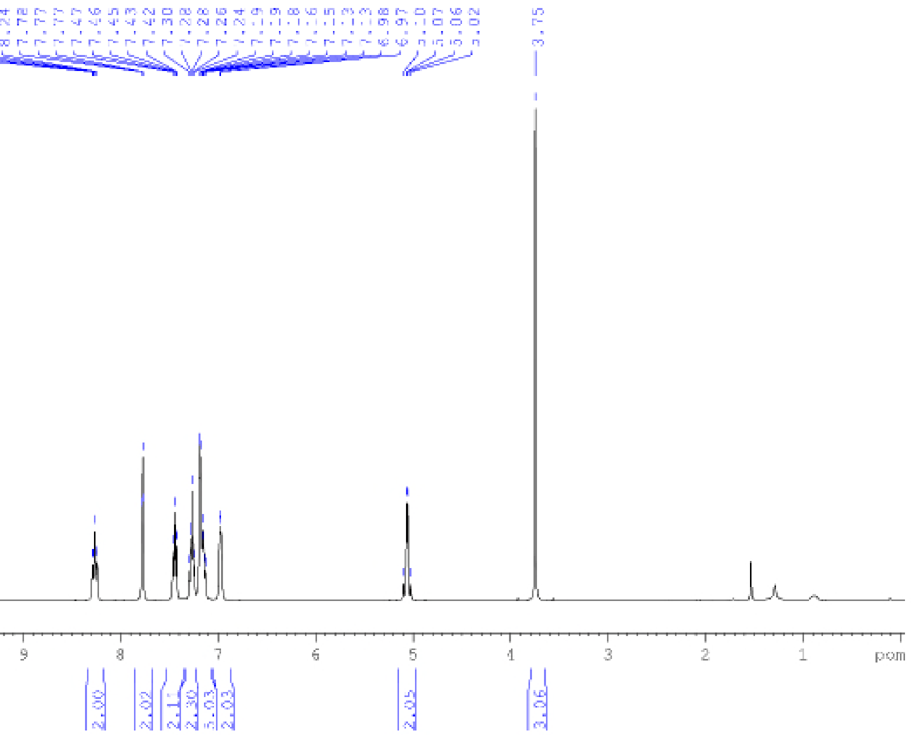

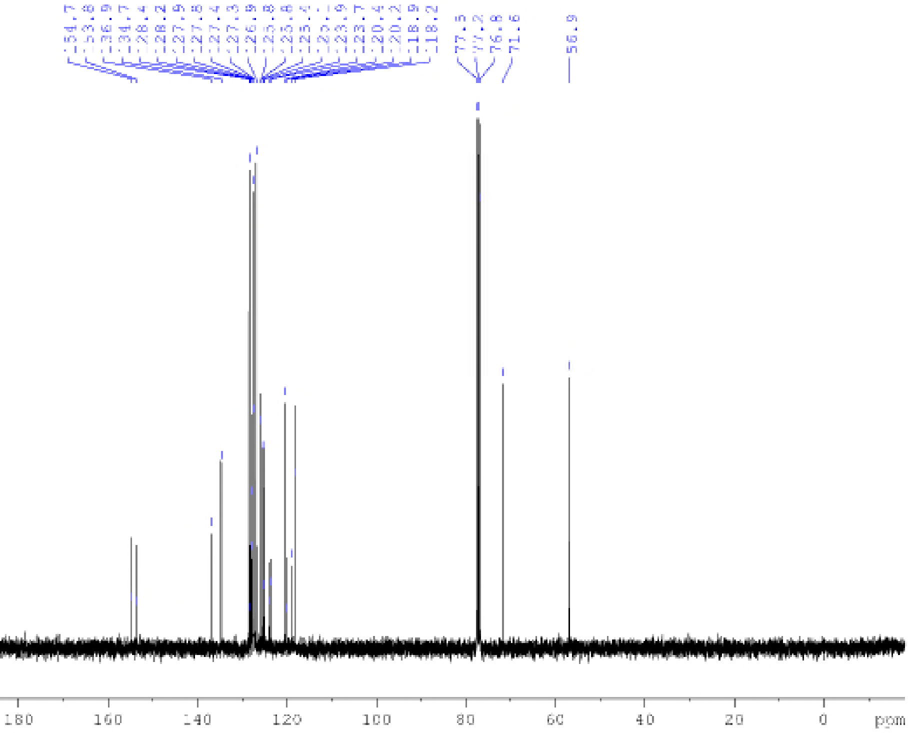

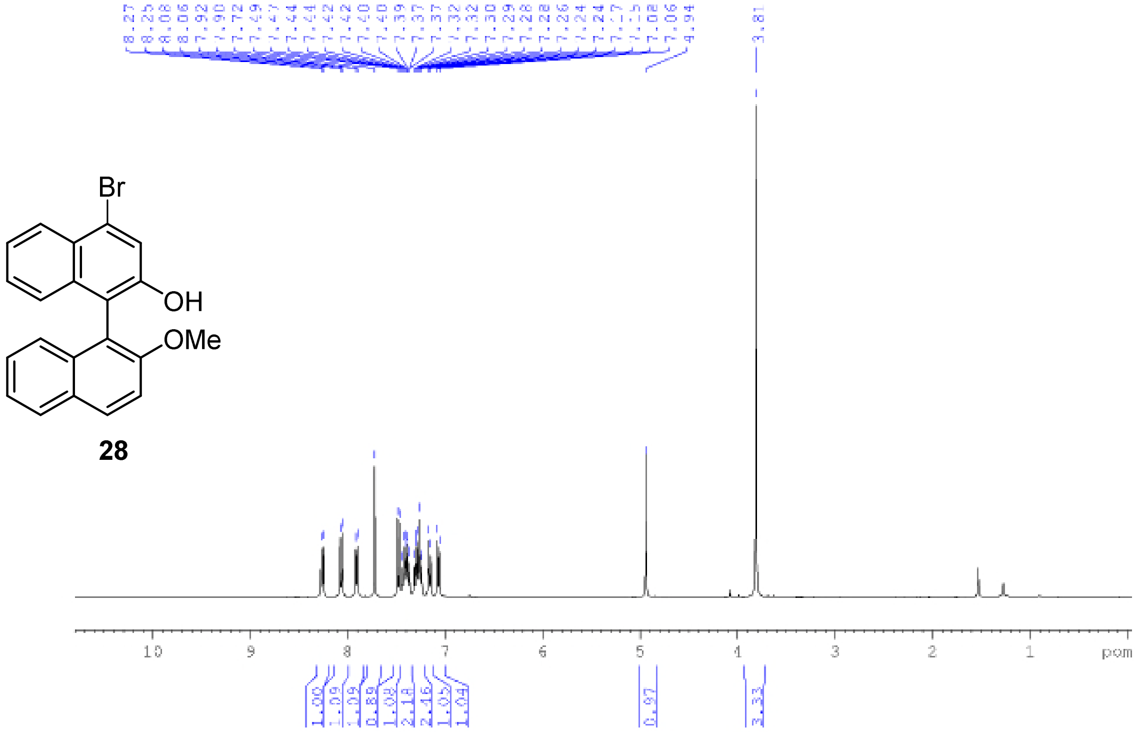

28

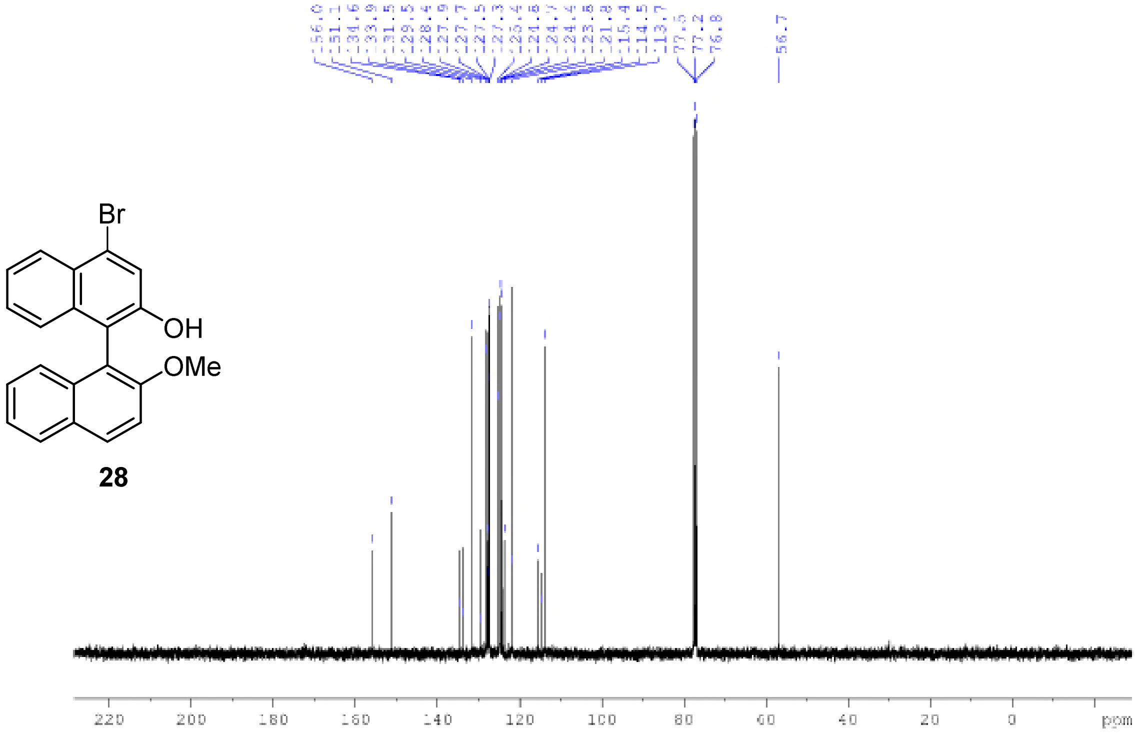

28

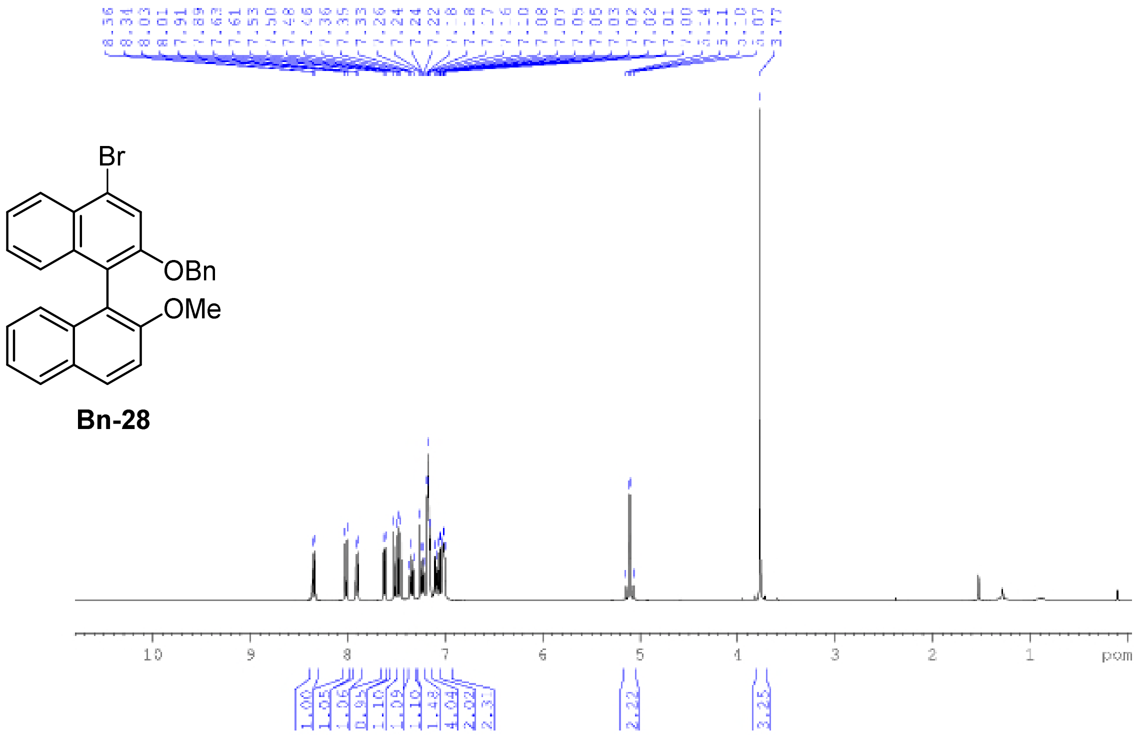

Bn-28

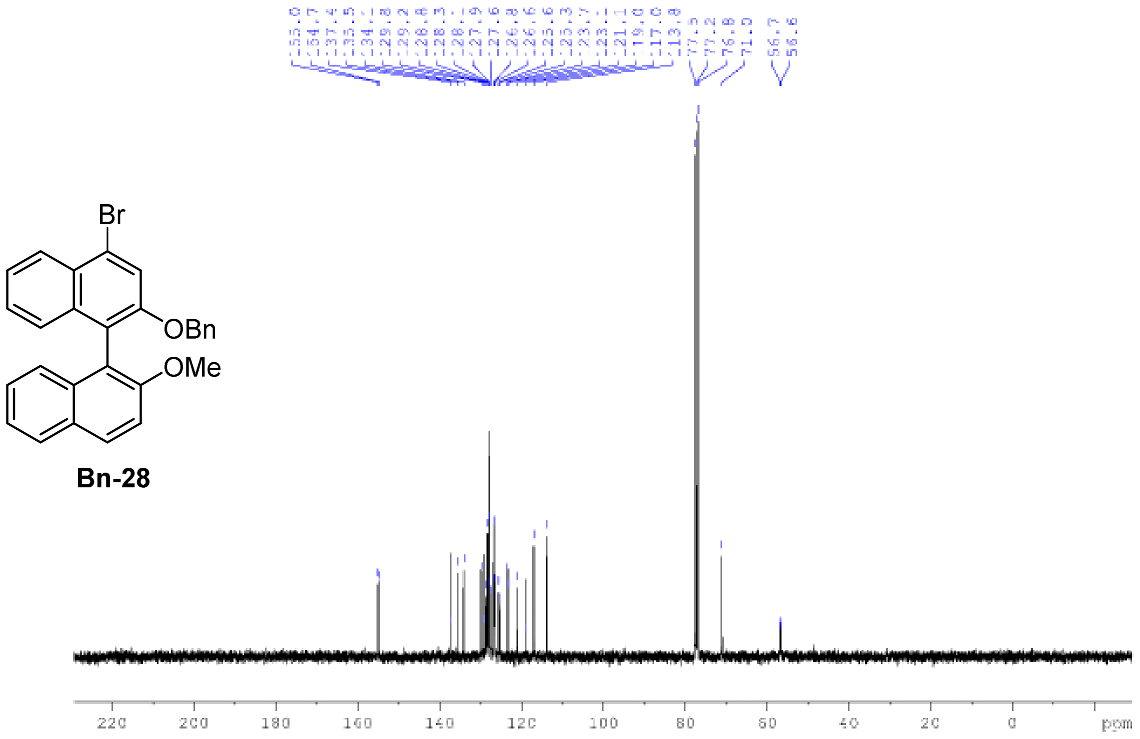

Bn-28

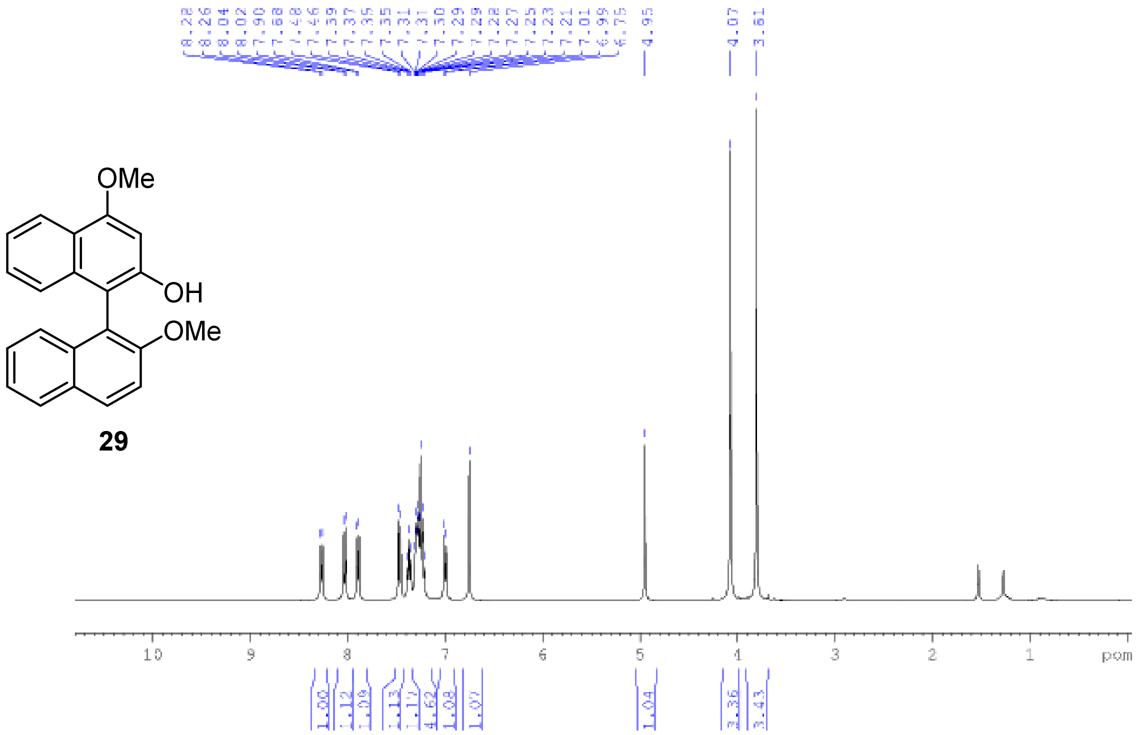
29

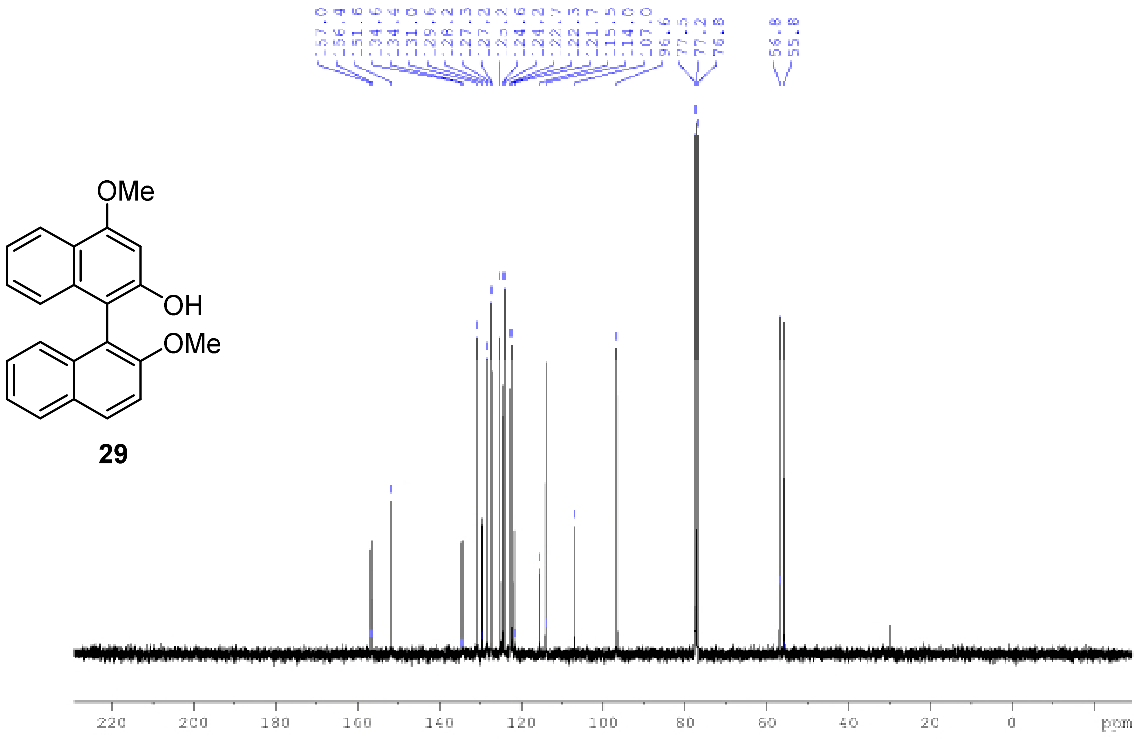
29

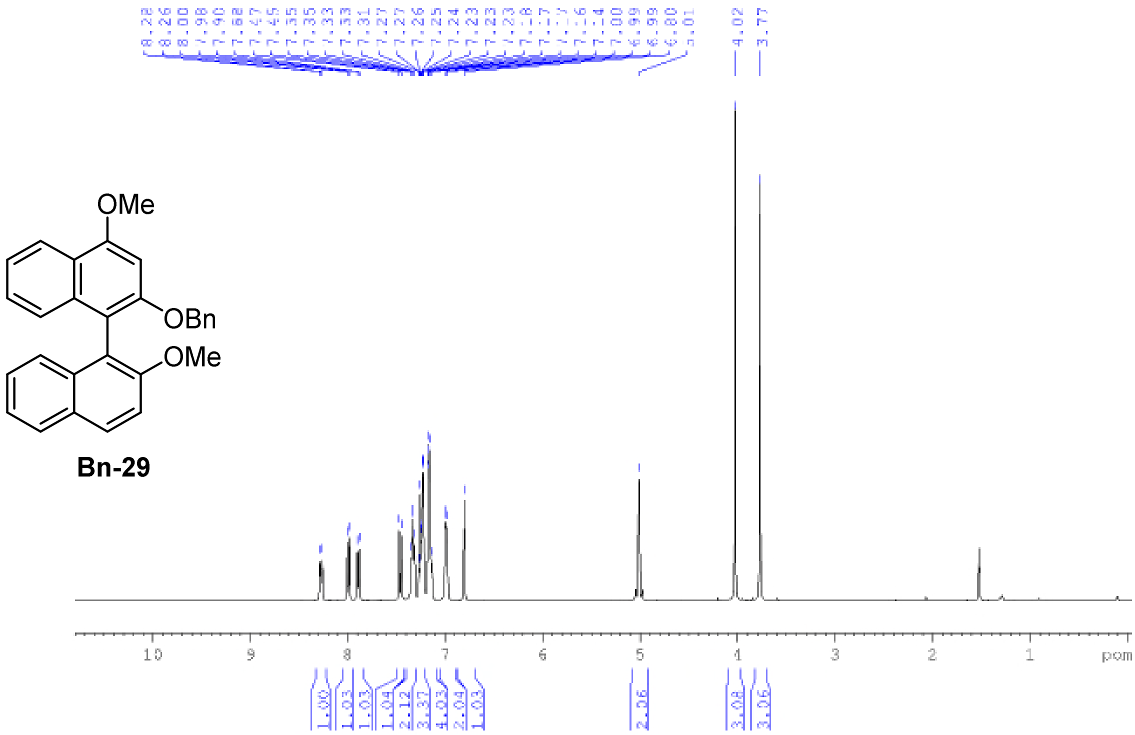
OMe
OBn
OMe
Bn-29
ppm

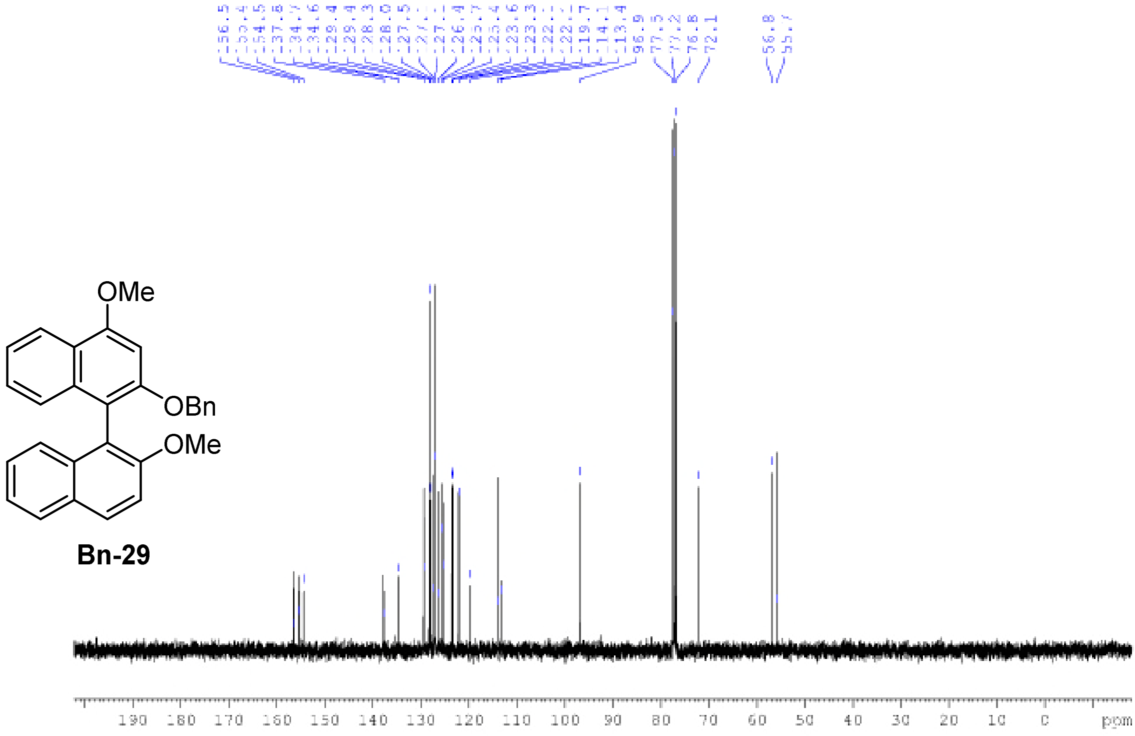
OMe
OBn
OMe
Bn-29
ppm

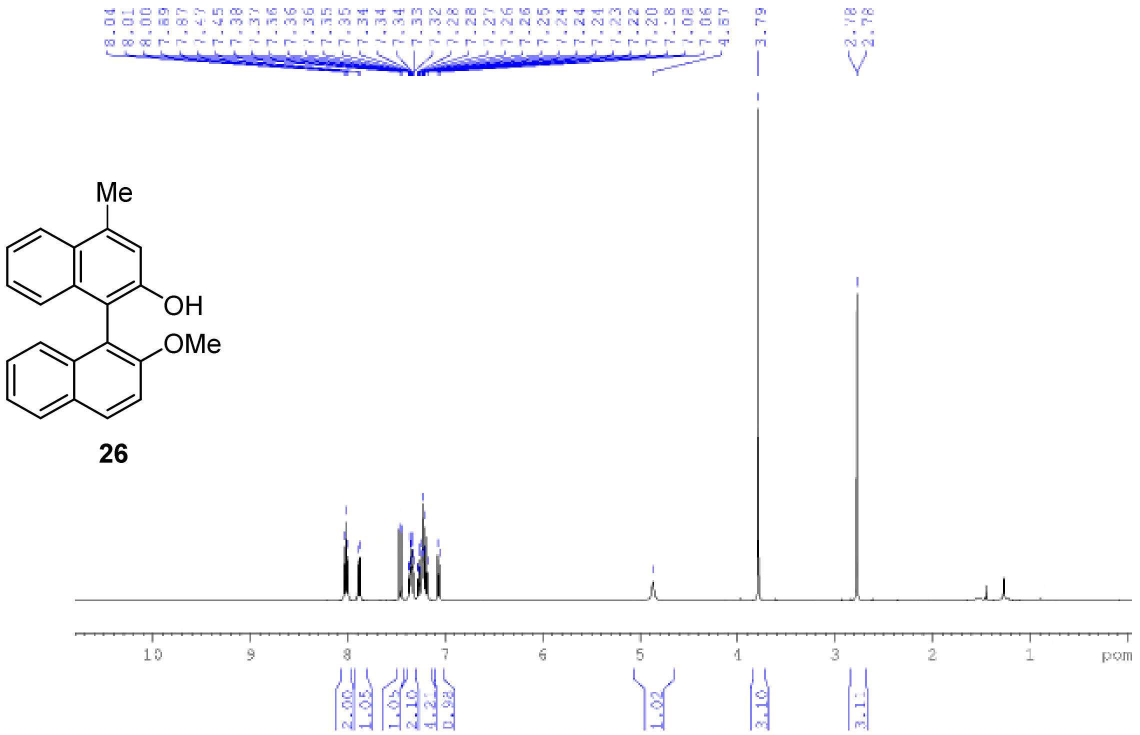

26

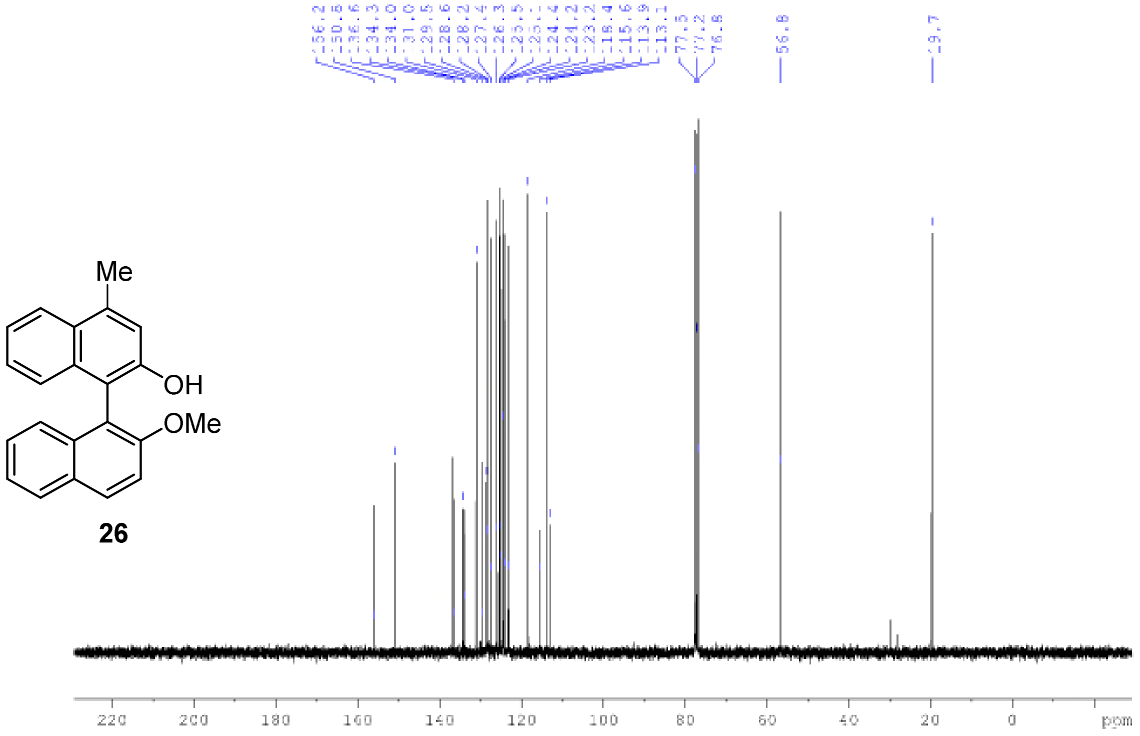

26

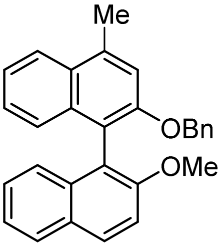

Bn-26

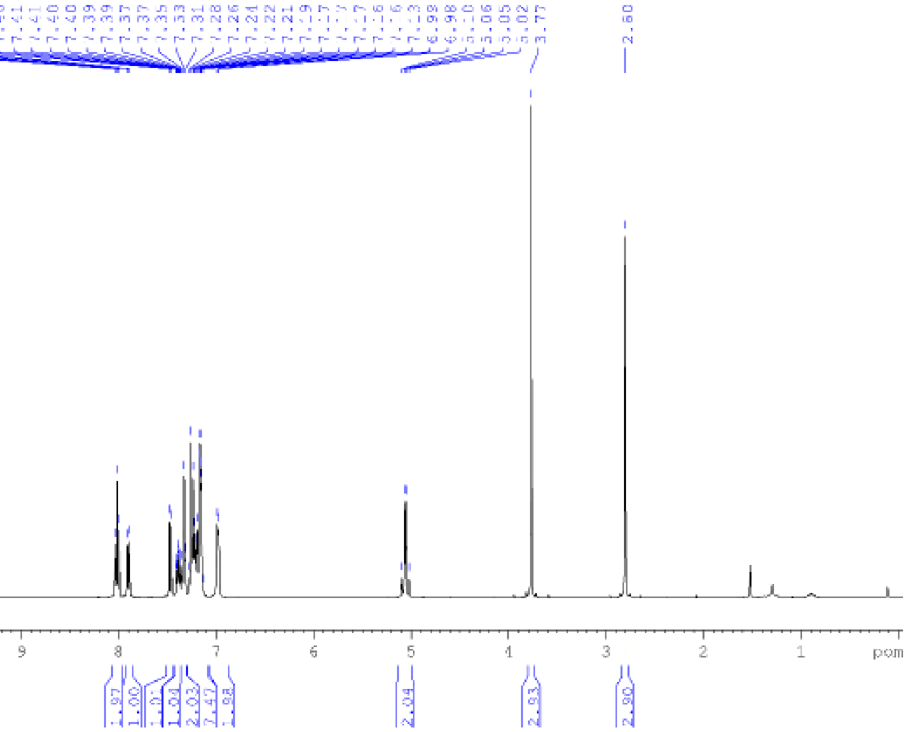

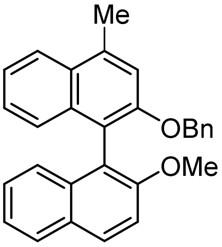

Bn-26

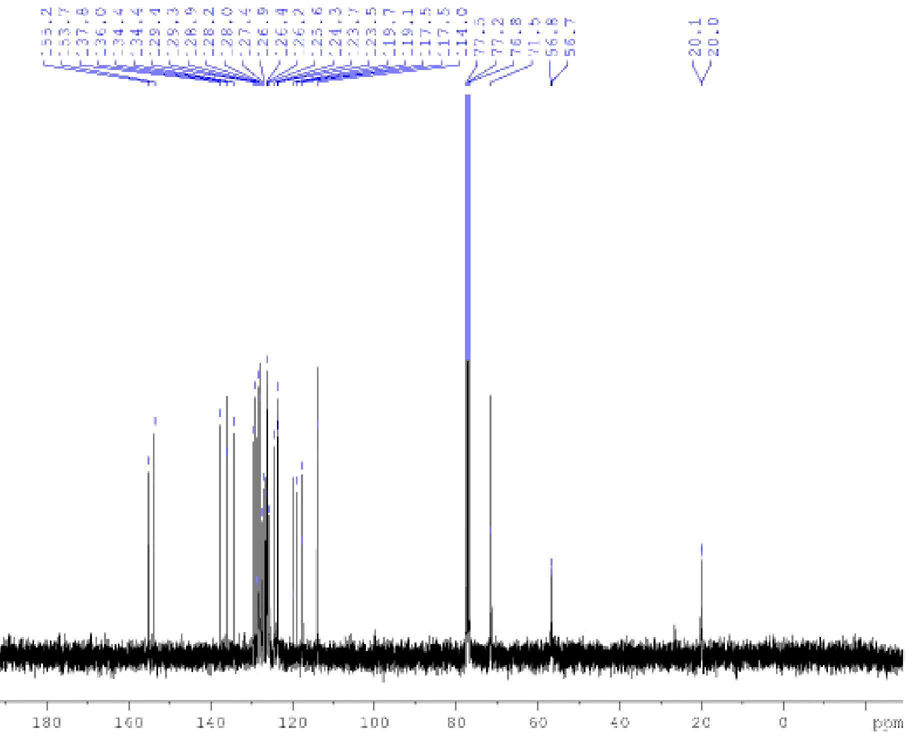

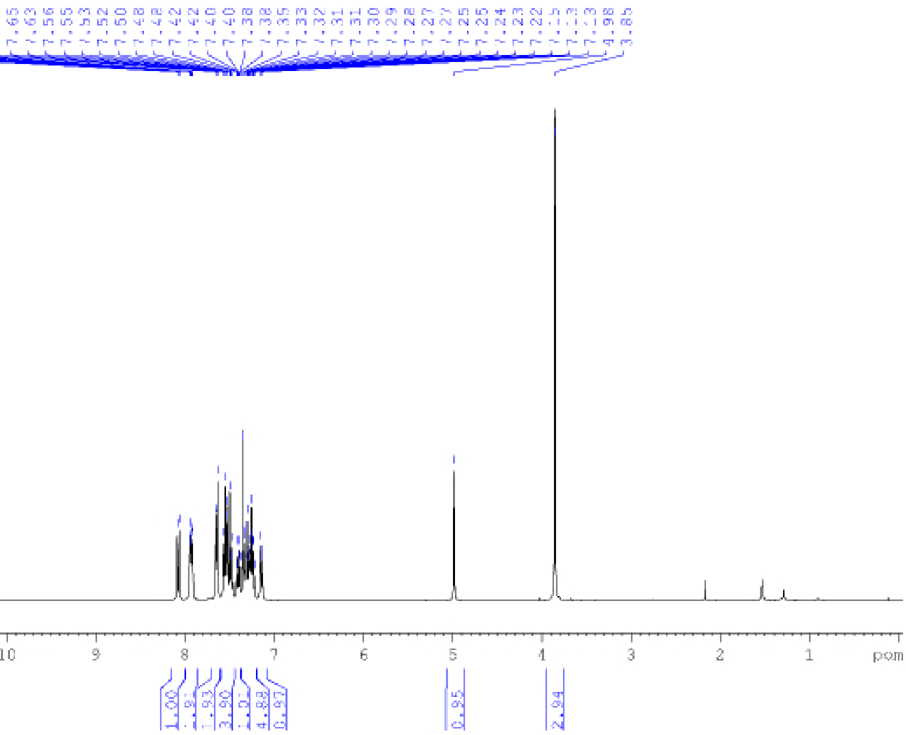

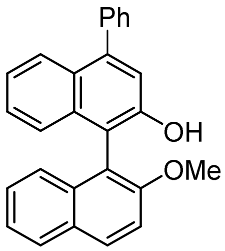

27

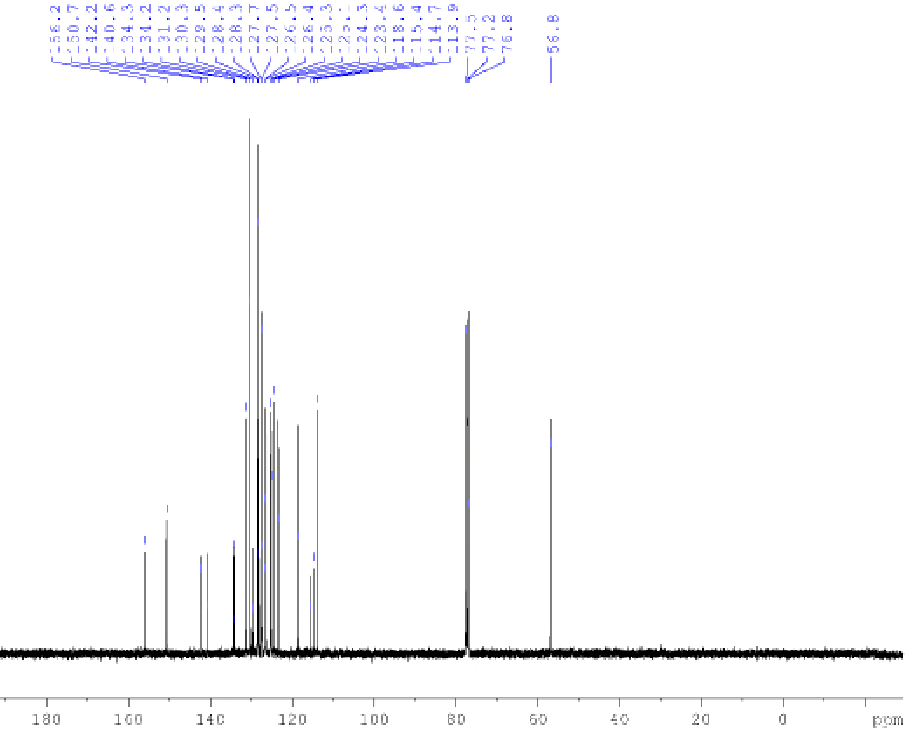

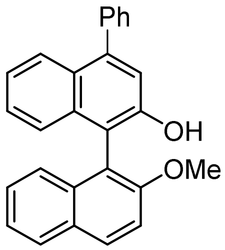

27

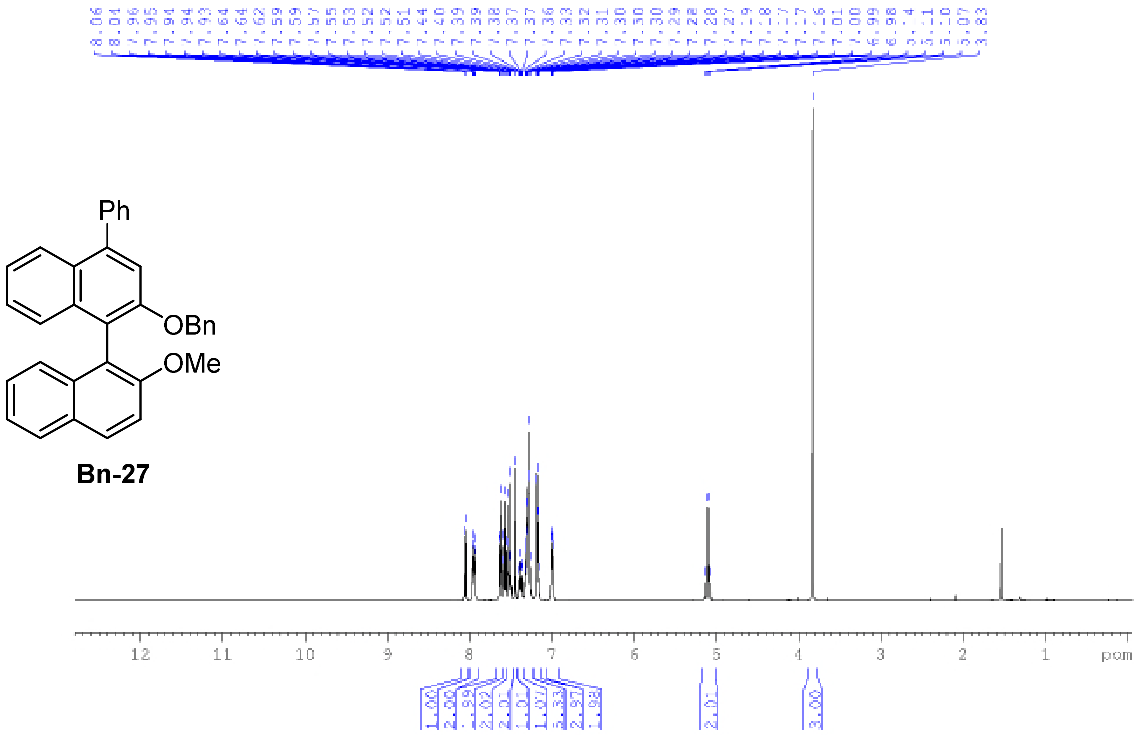

Bn-27

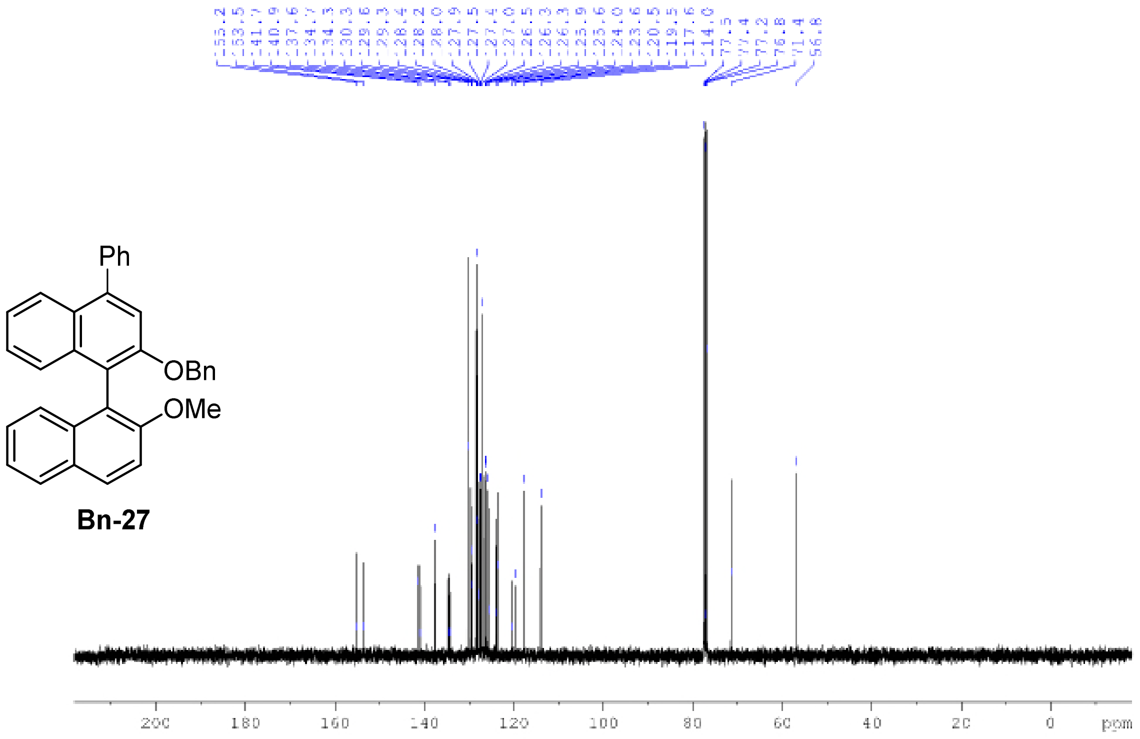

Bn-27

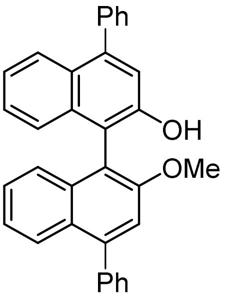

42

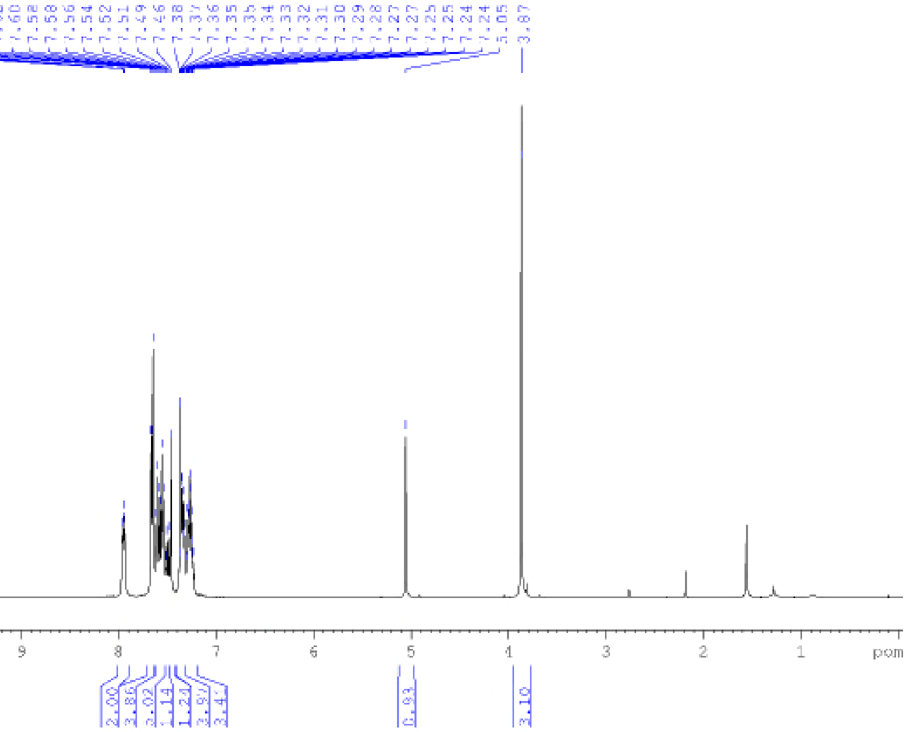

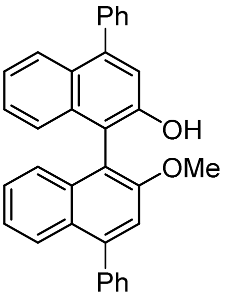

42

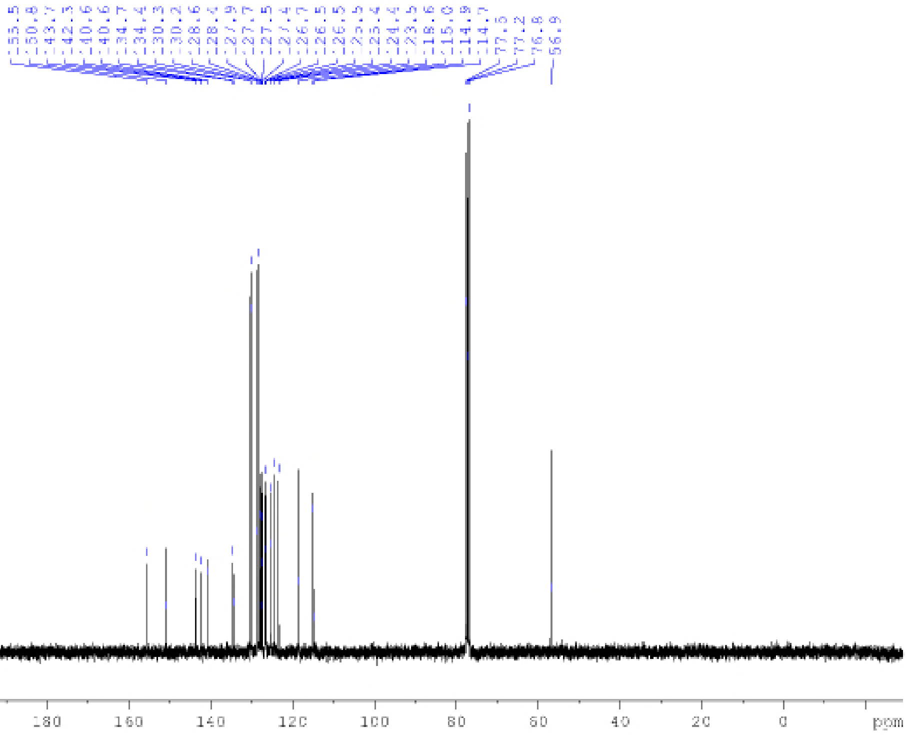

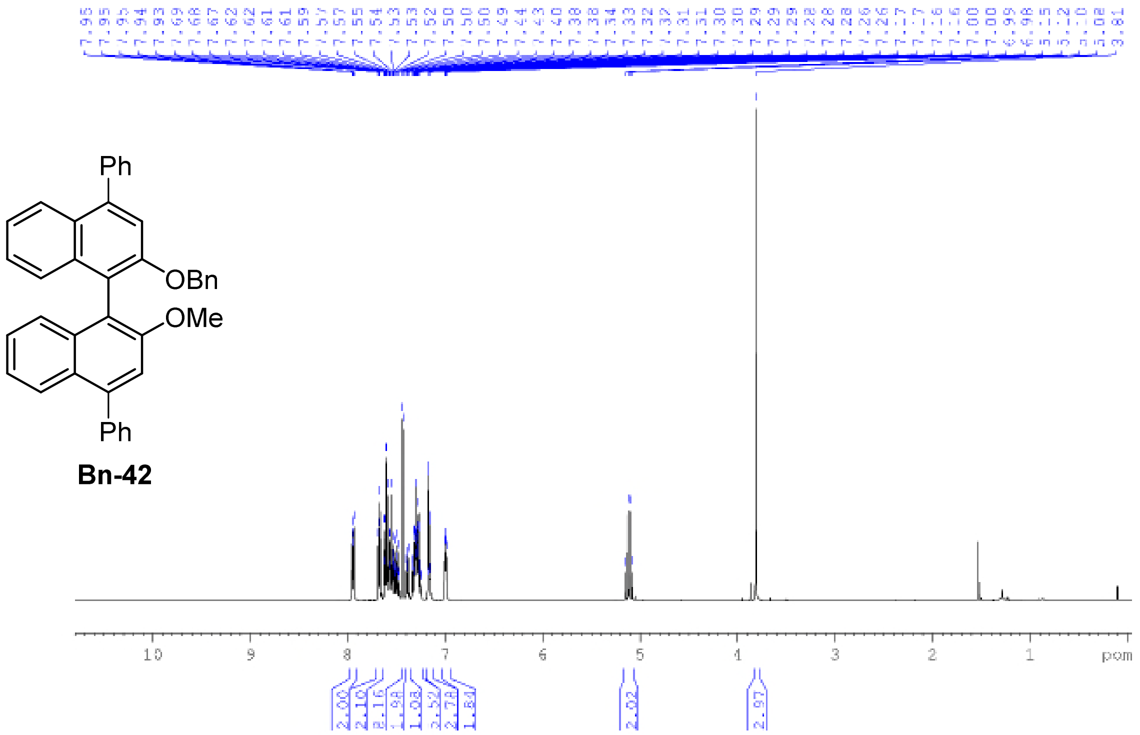

Ph
OBn
OMe
Ph
Bn-42
ppm

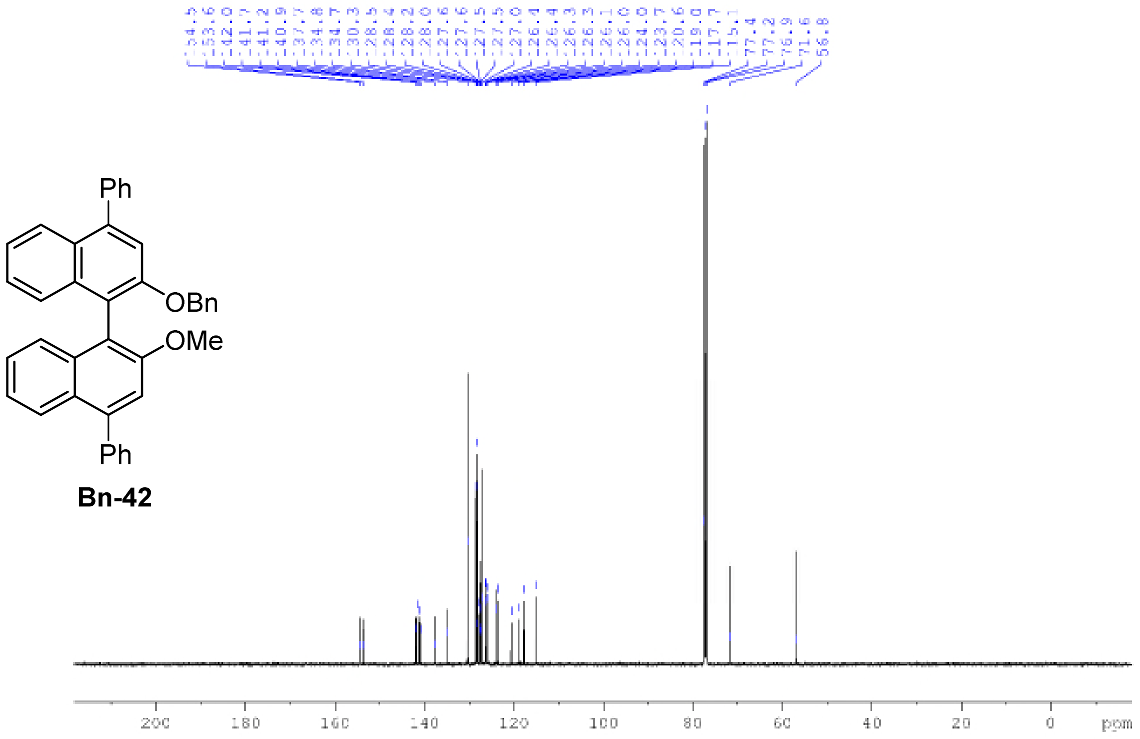

Ph
OBn
OMe
Ph
Bn-42
ppm

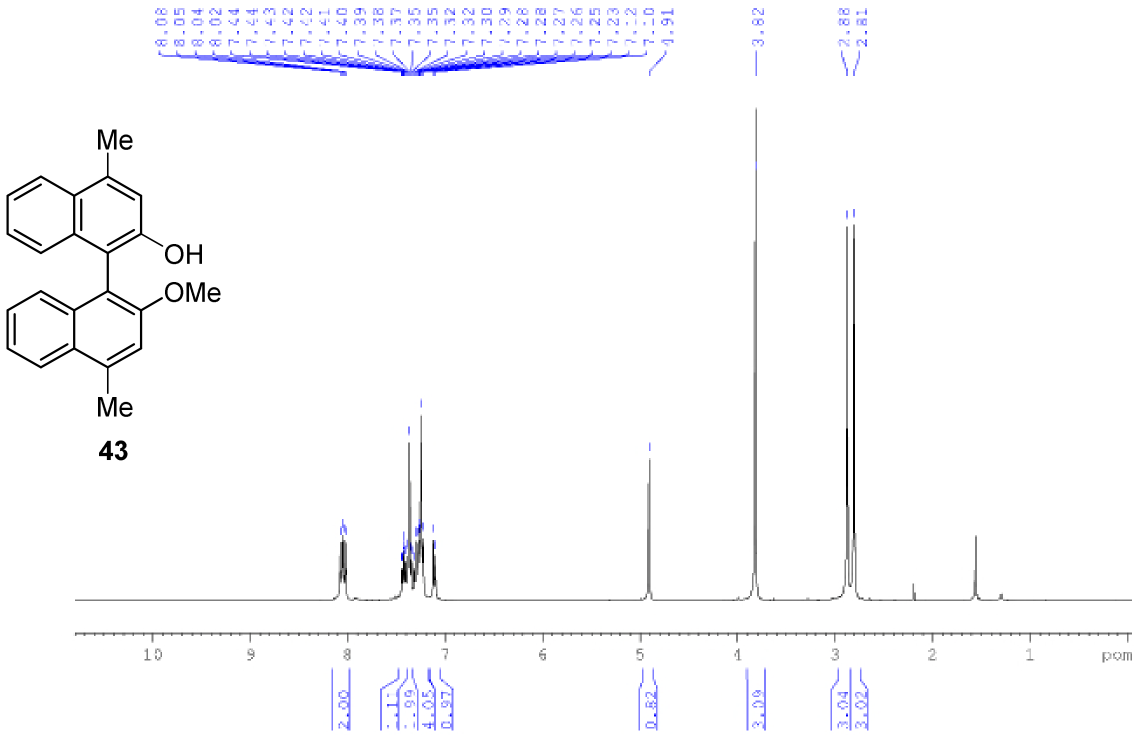
Me
OH
OMe
Me
3.82
2.88
2.81
ppm

43

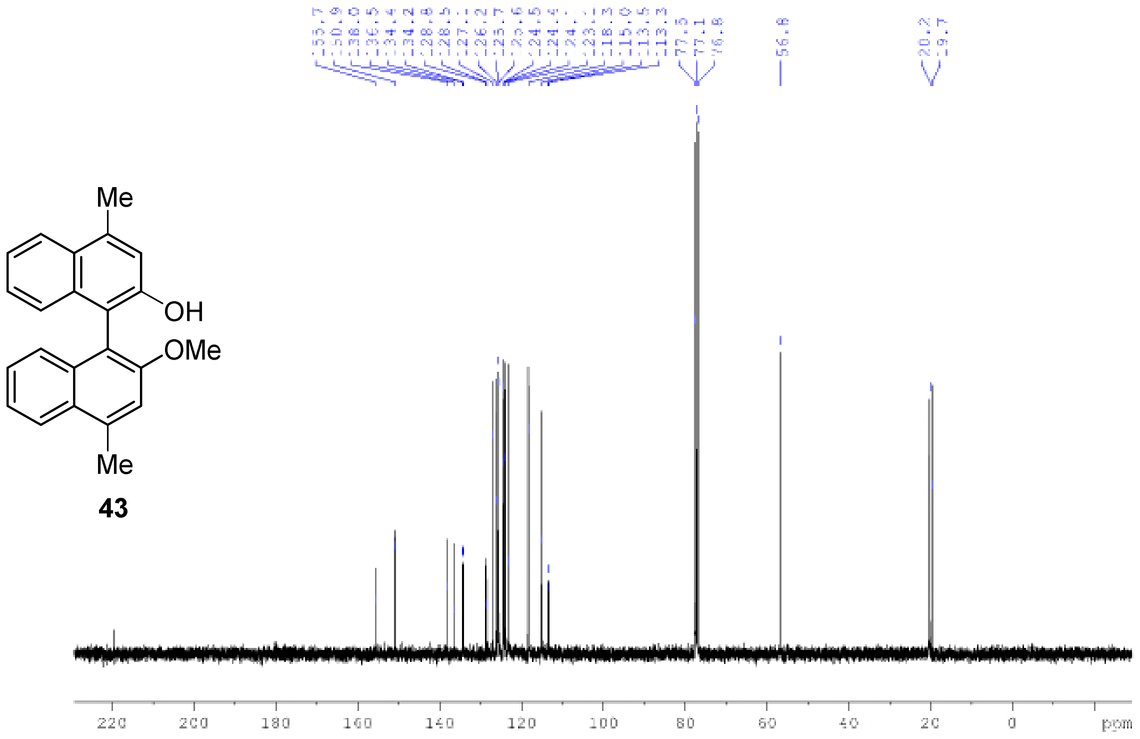
Me
OH
OMe
Me
77.3
77.1
76.8
56.8
20.2
19.7
ppm

43

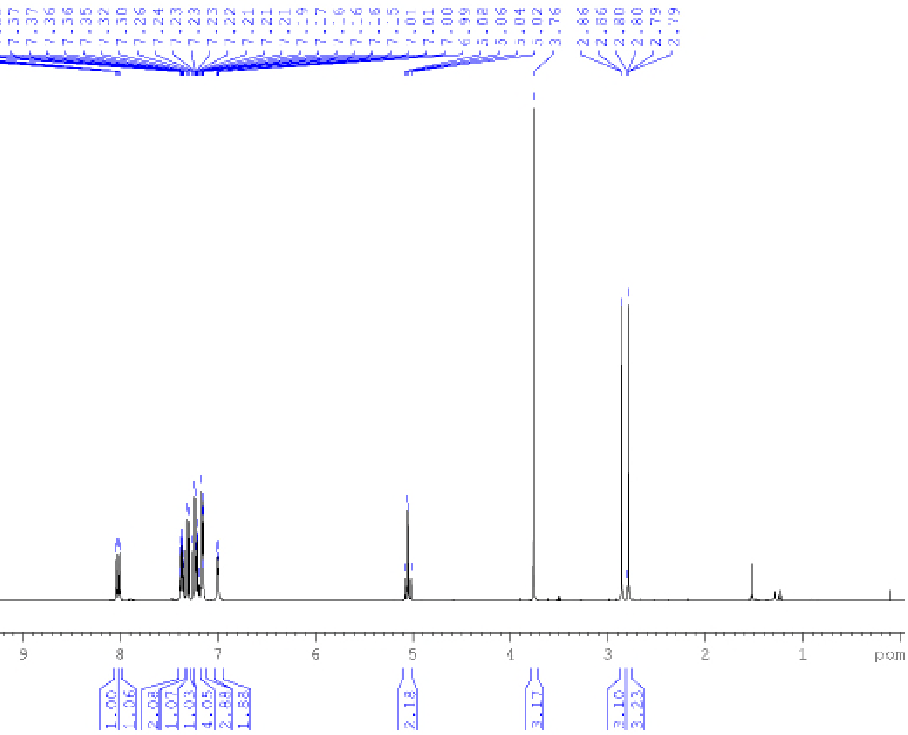

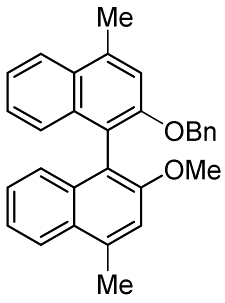

Bn-43

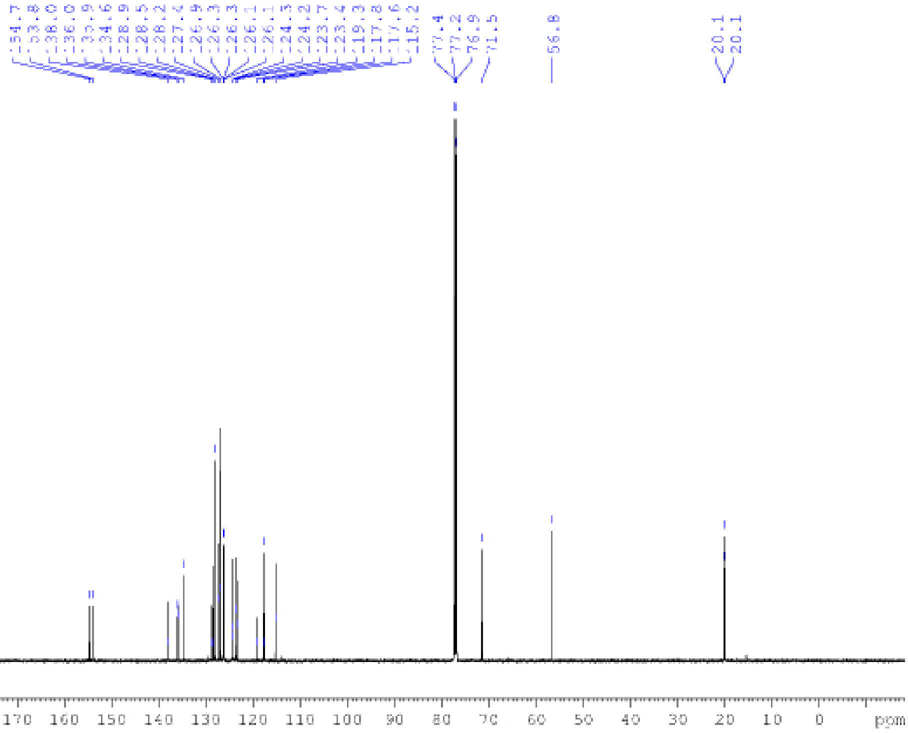

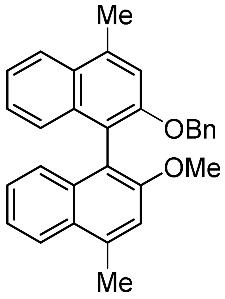

Bn-43

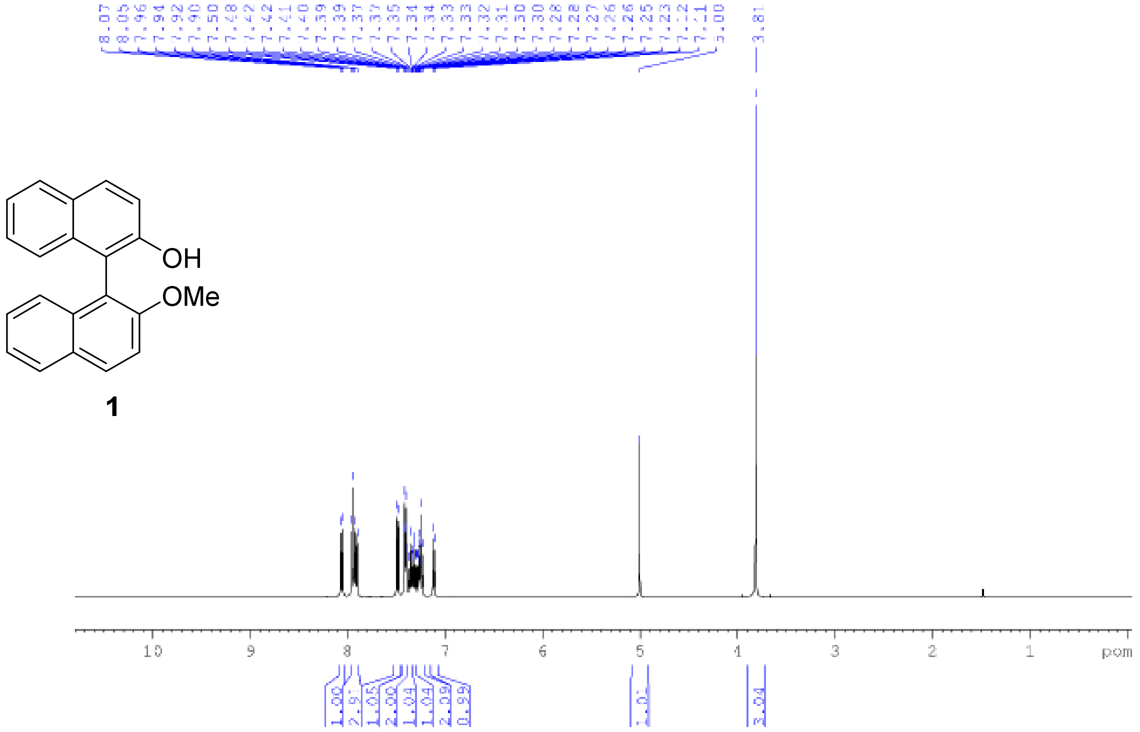

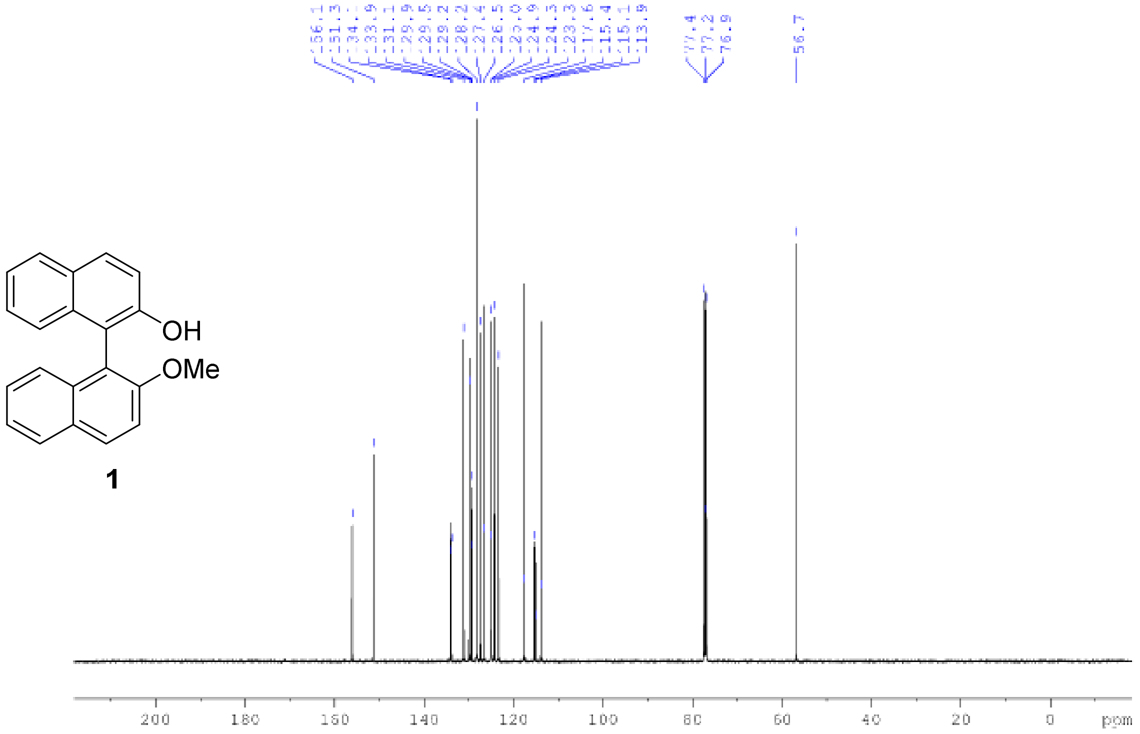

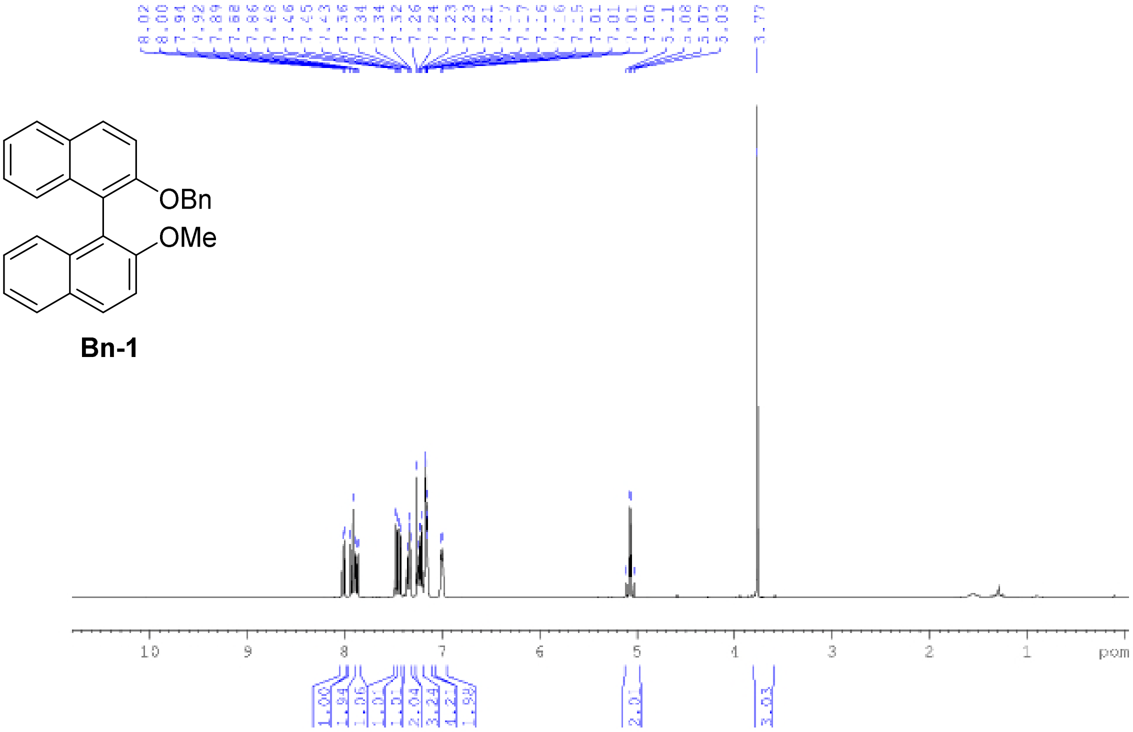

Bn-1

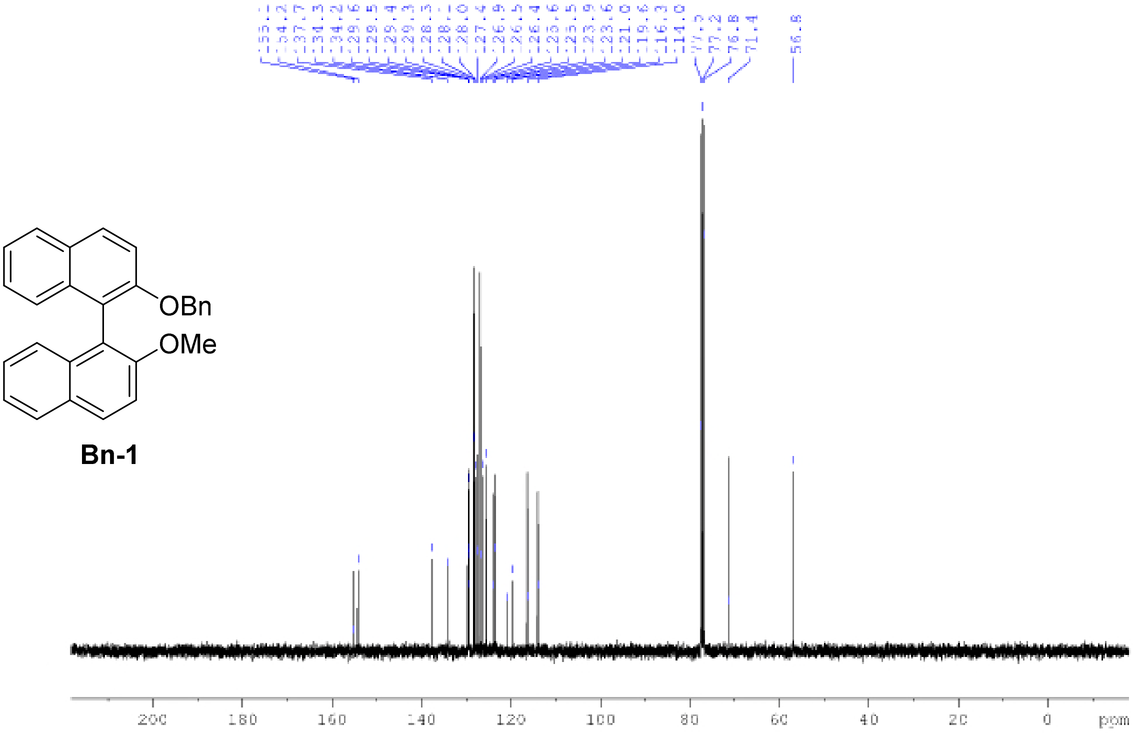

Bn-1

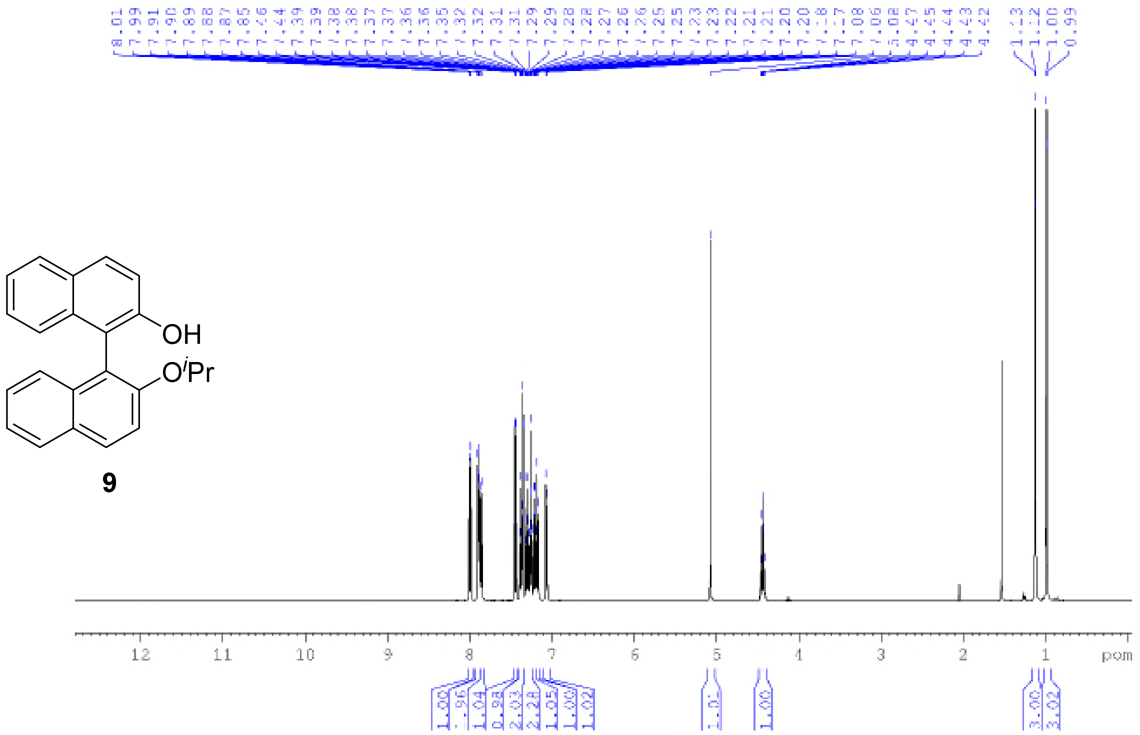

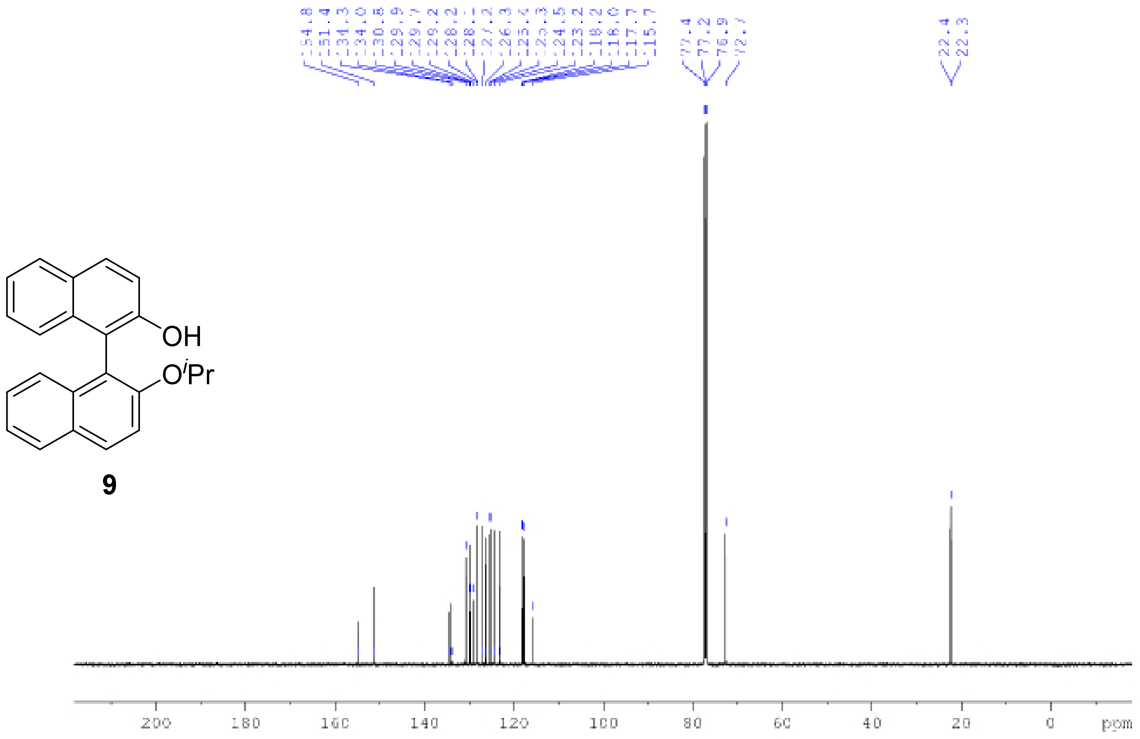

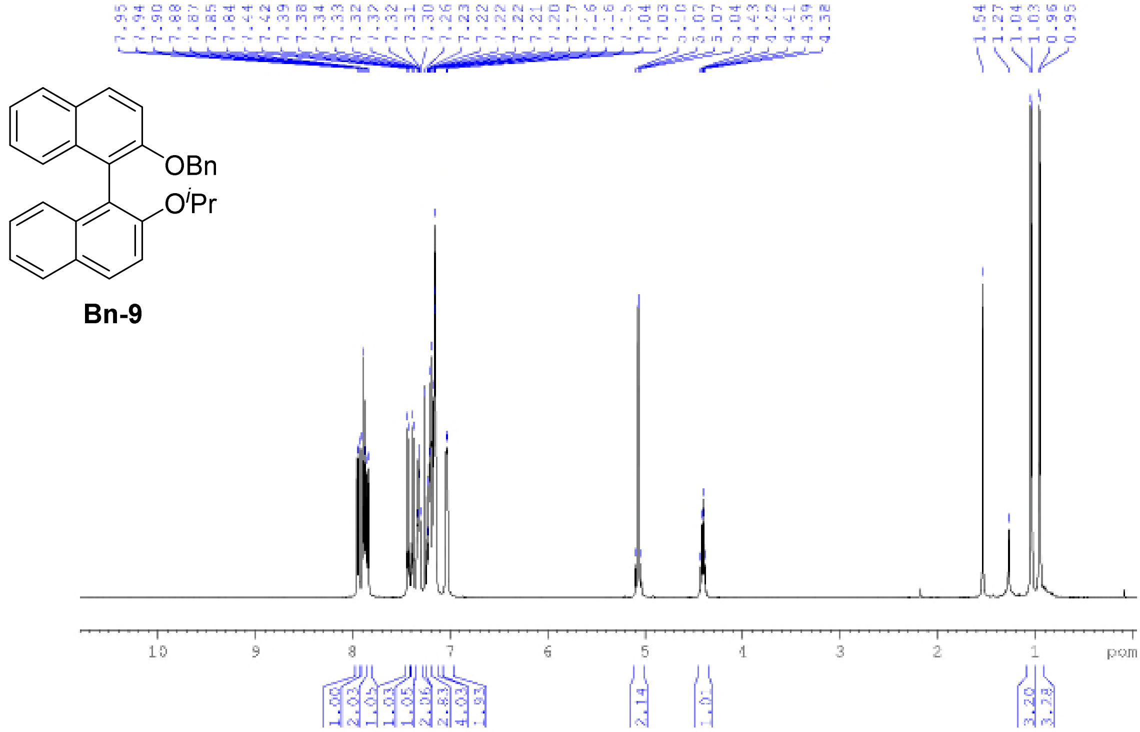

Bn-9

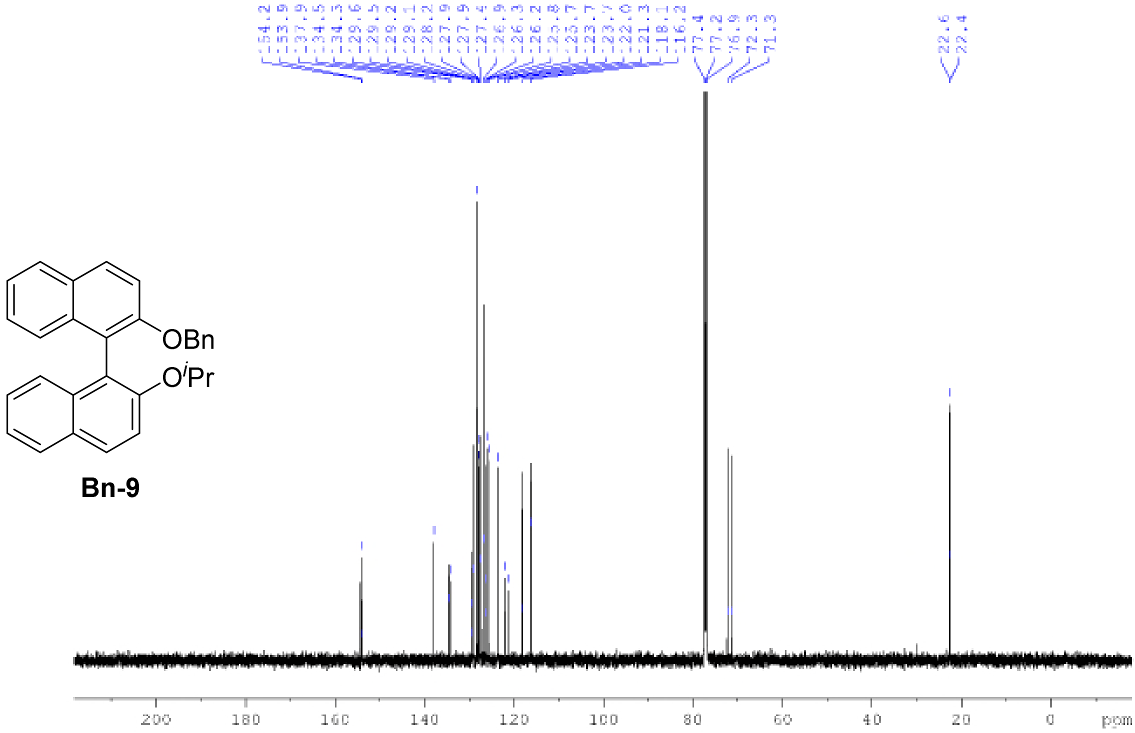

Bn-9

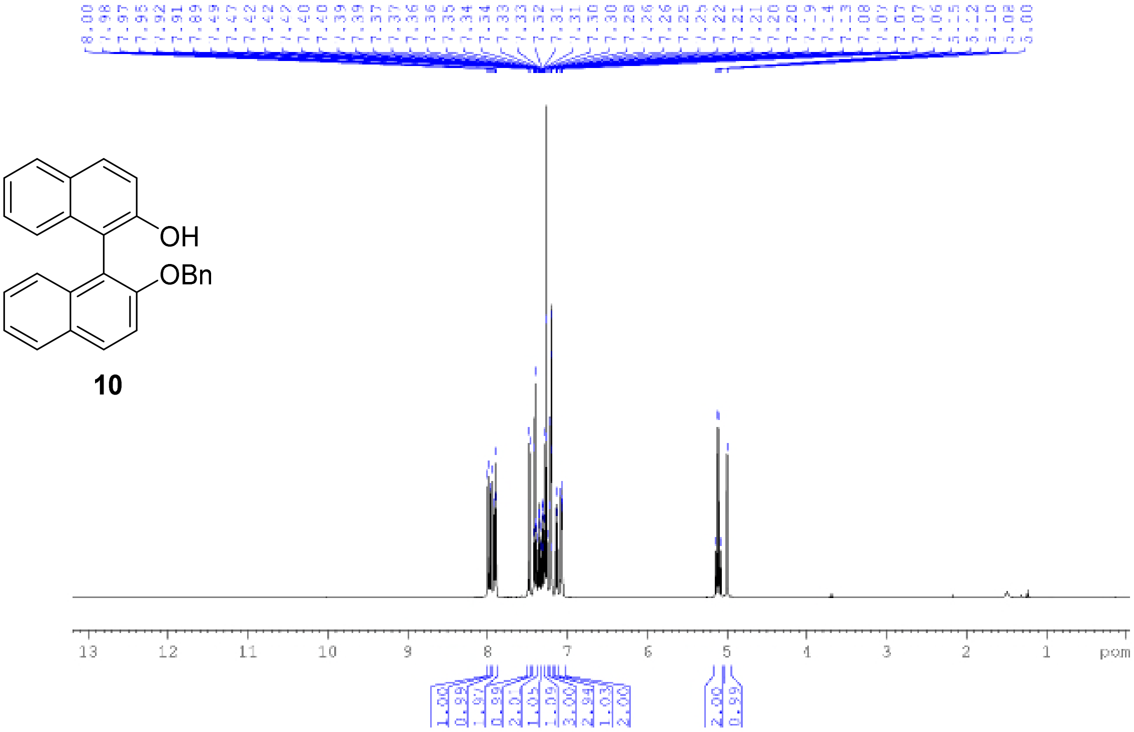

10

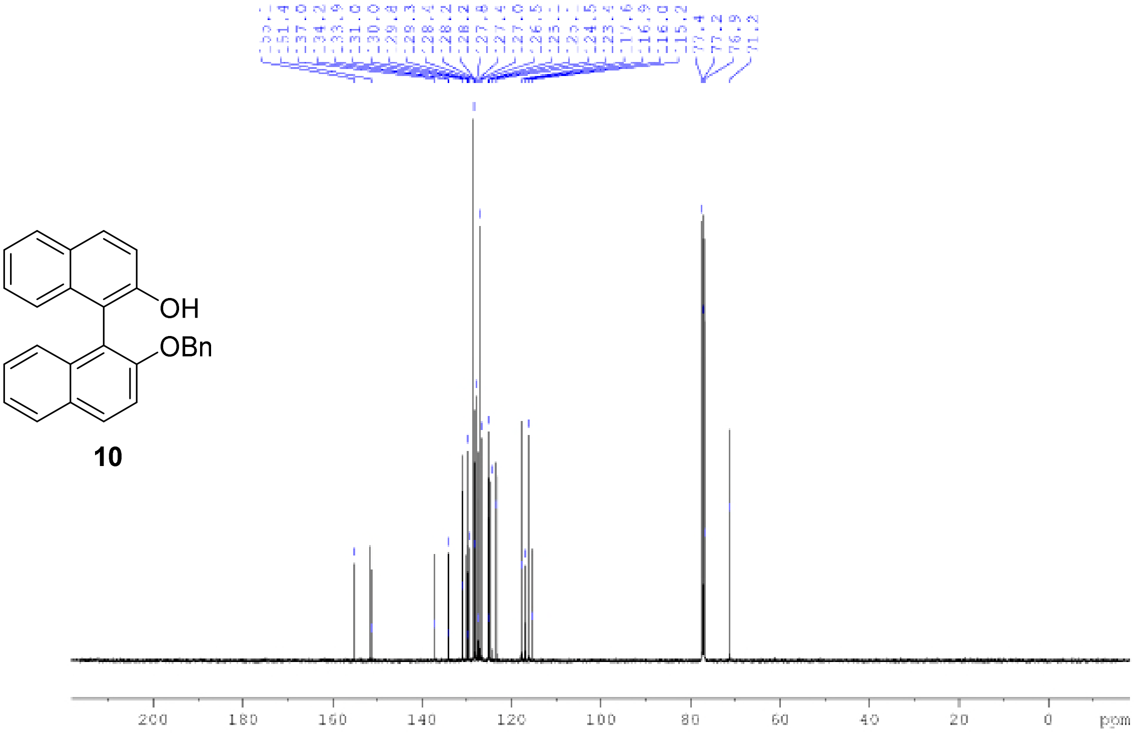

10

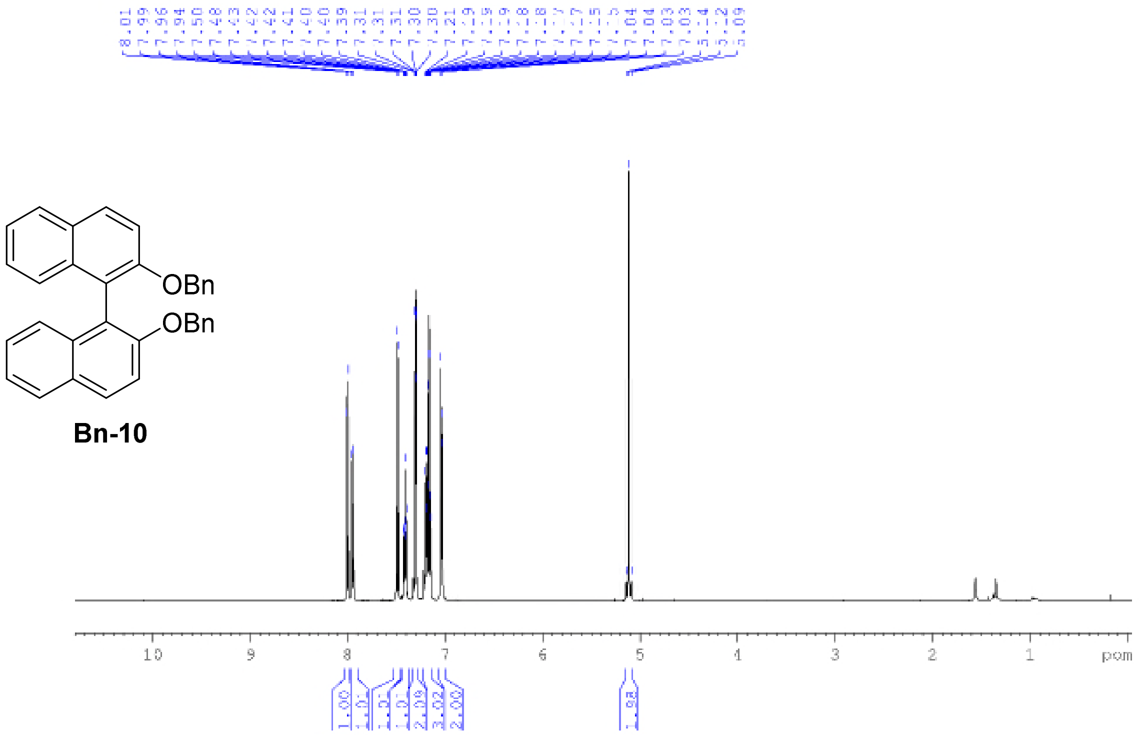

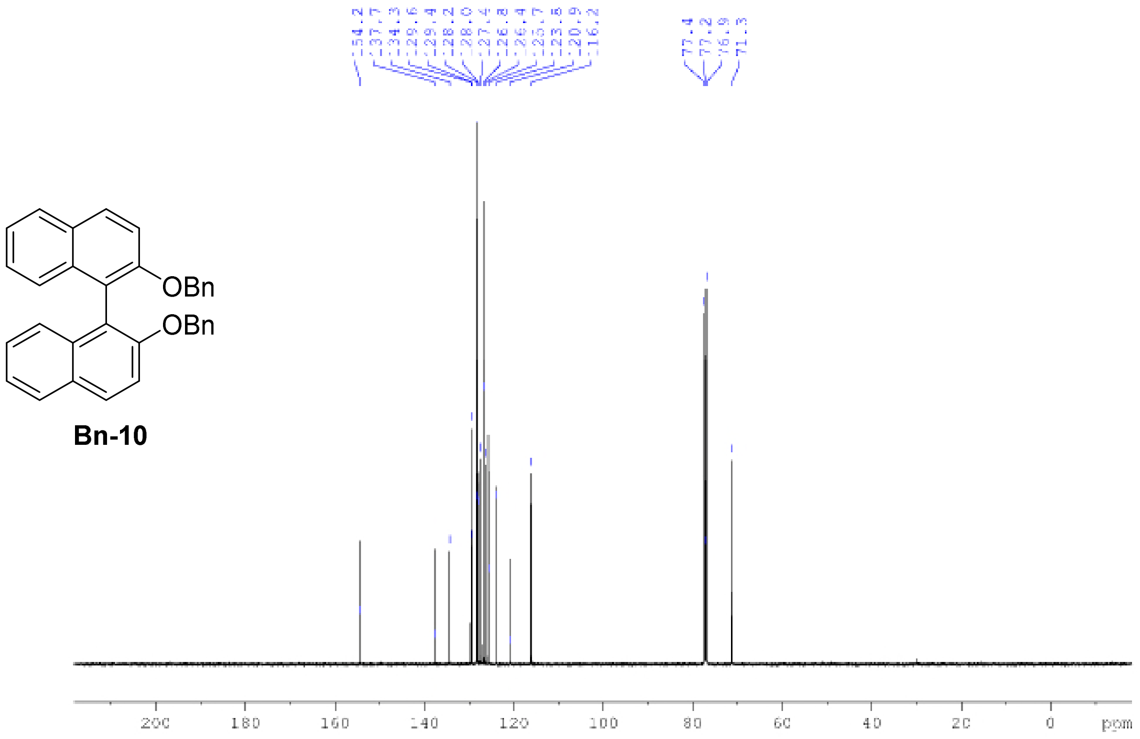

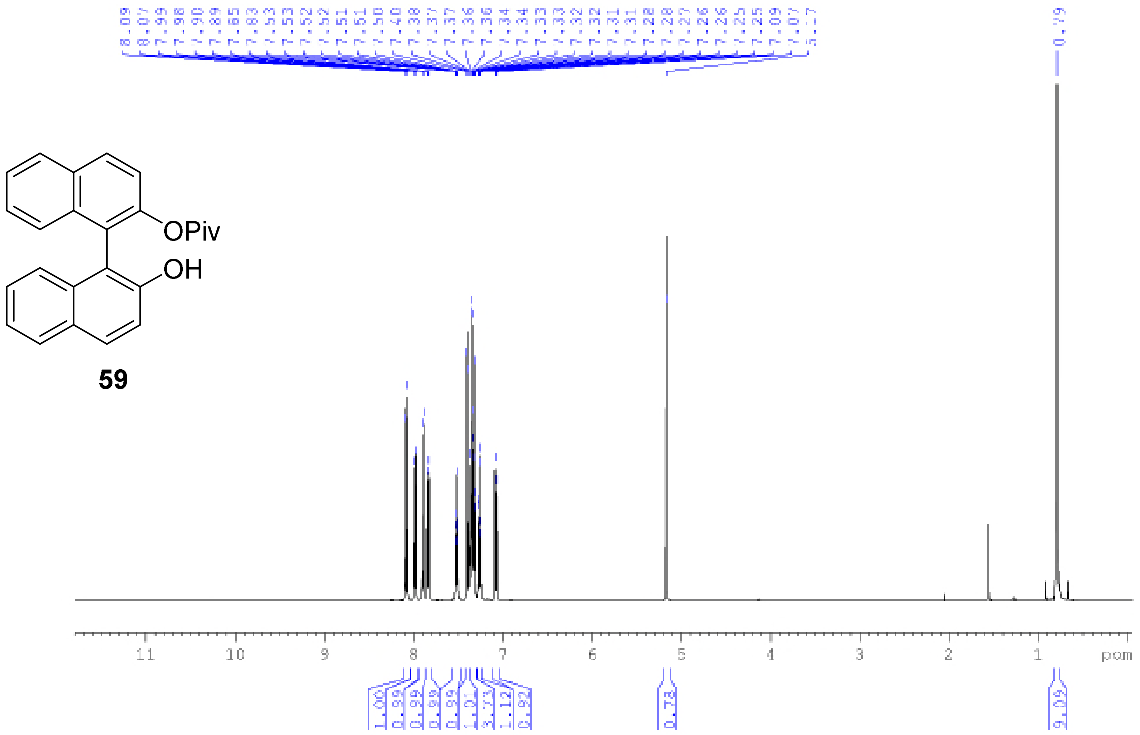

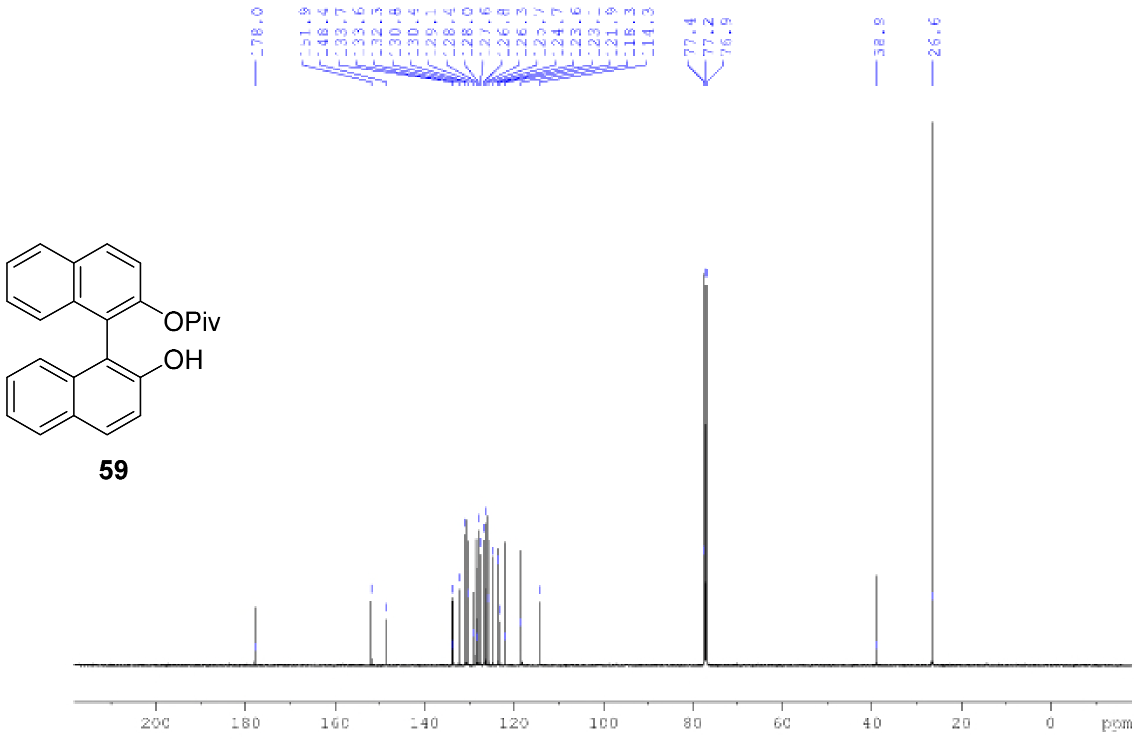

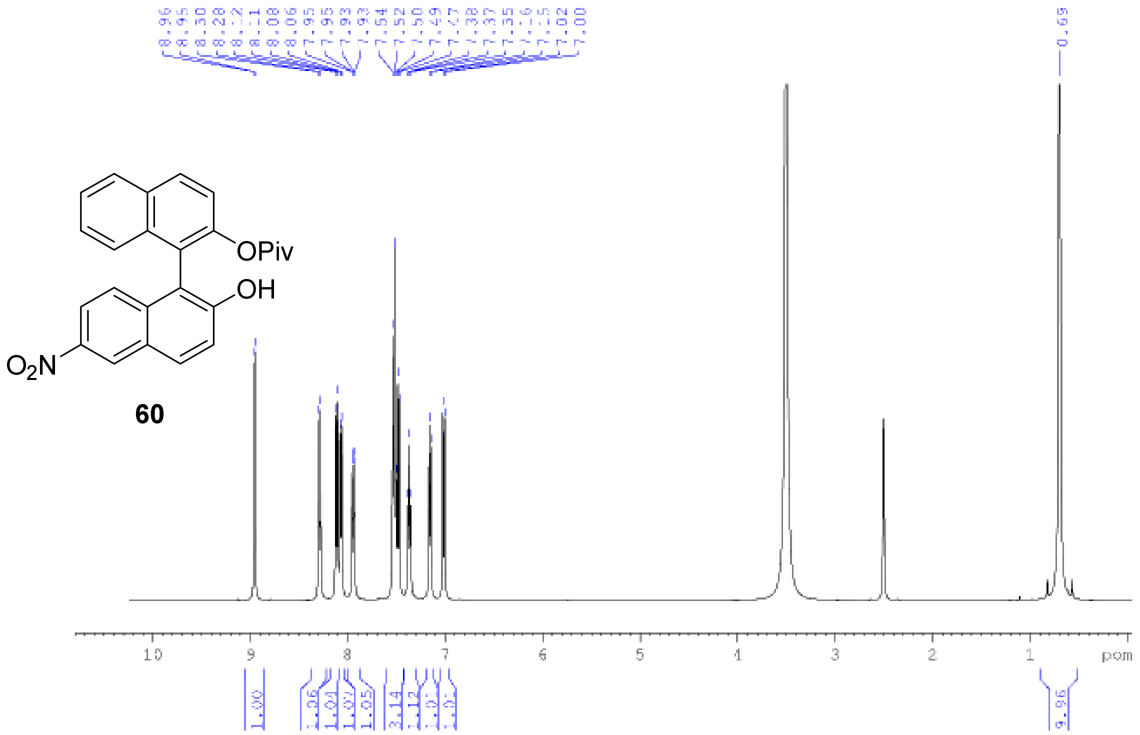
OPiv
OH
O_2N
60
ppm

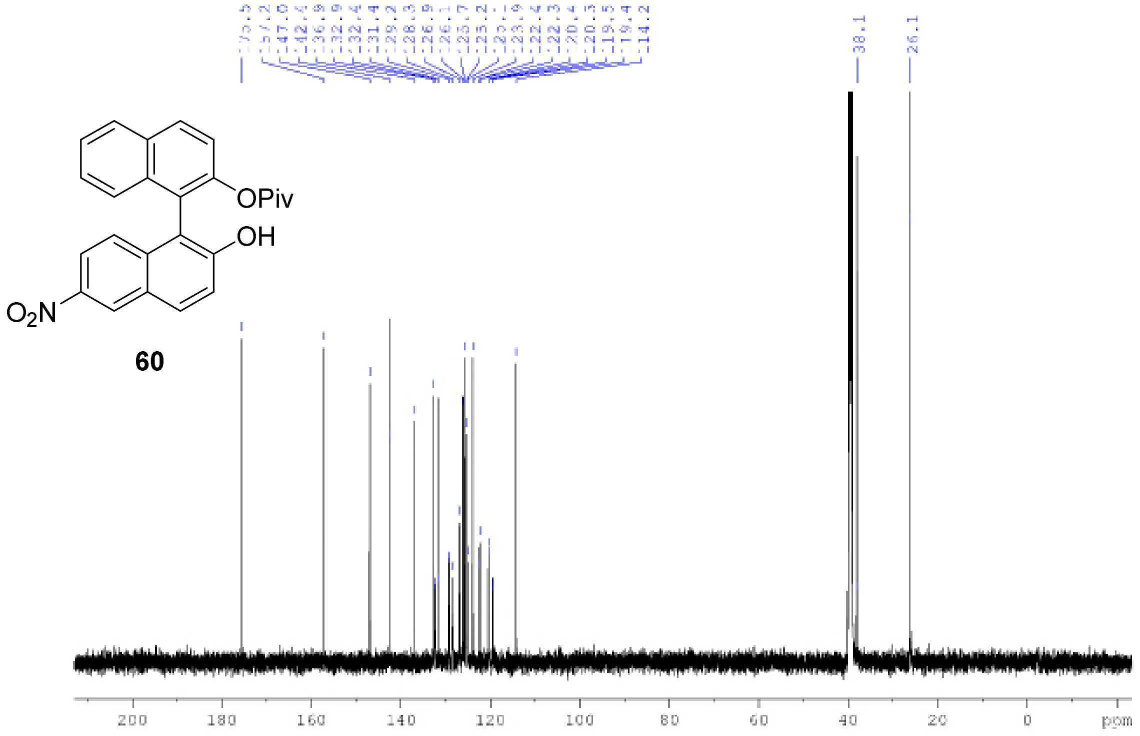
OPiv
OH
O_2N
60
38.1
26.1
ppm

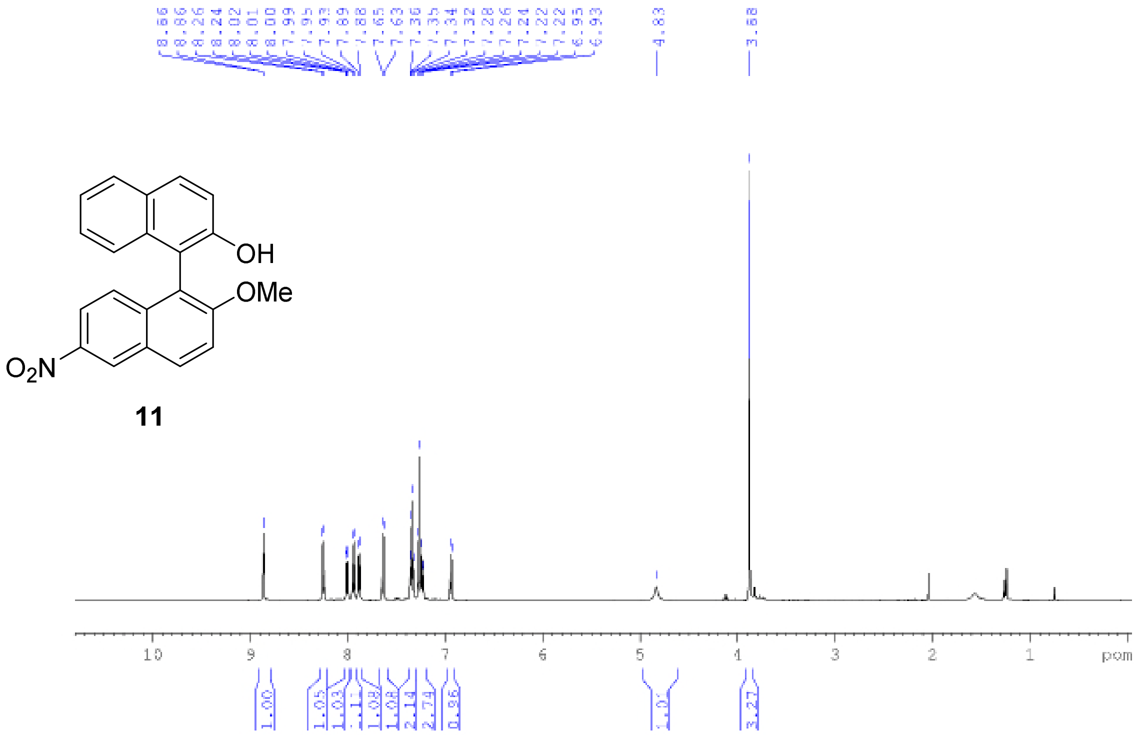

11

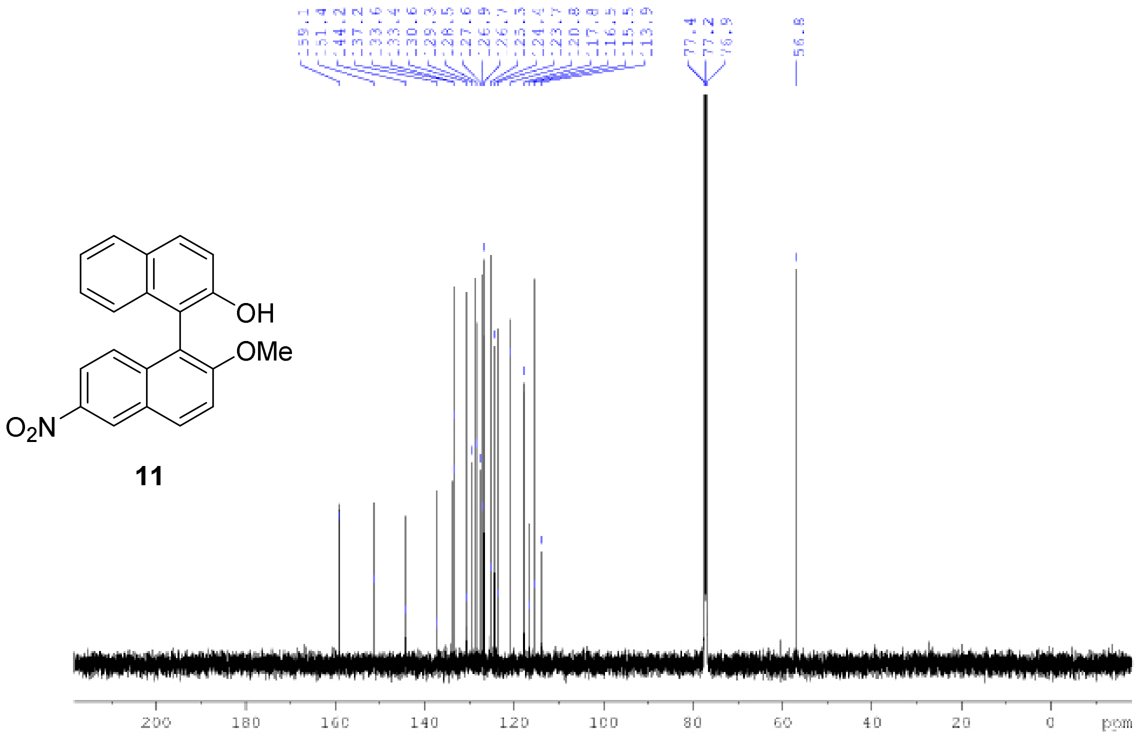

11

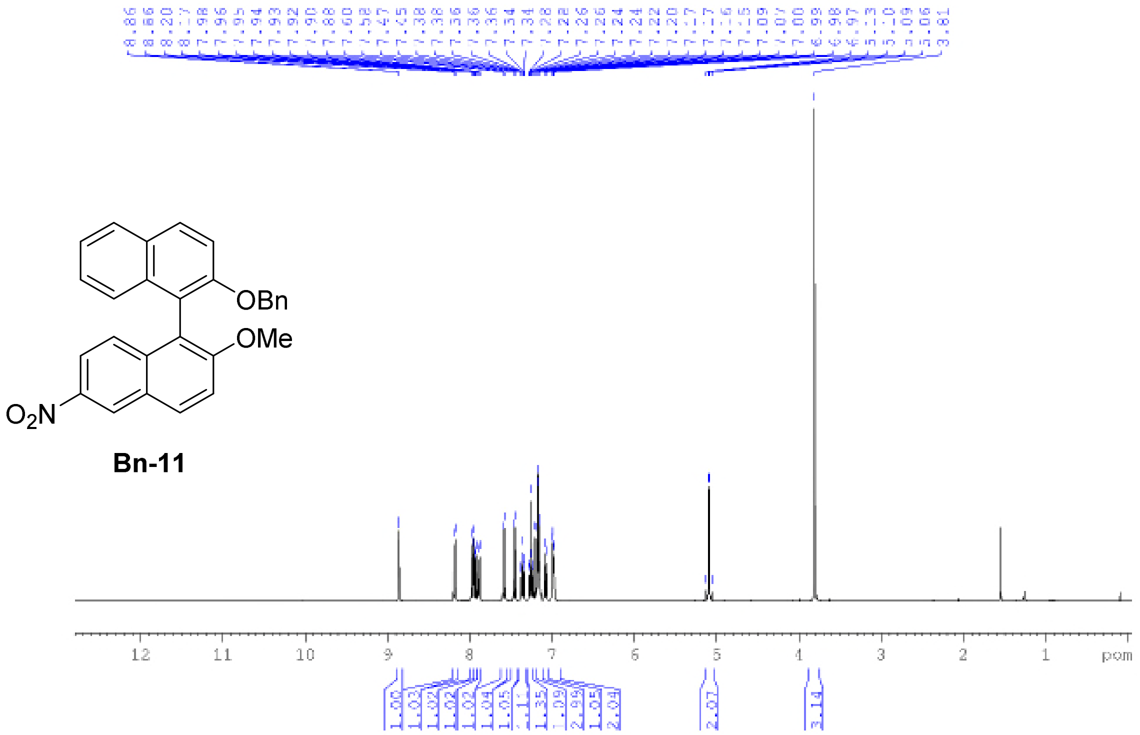
OBn
OMe
O2N
Bn-11
12 11 10 9 8 7 6 5 4 3 2 1 ppm

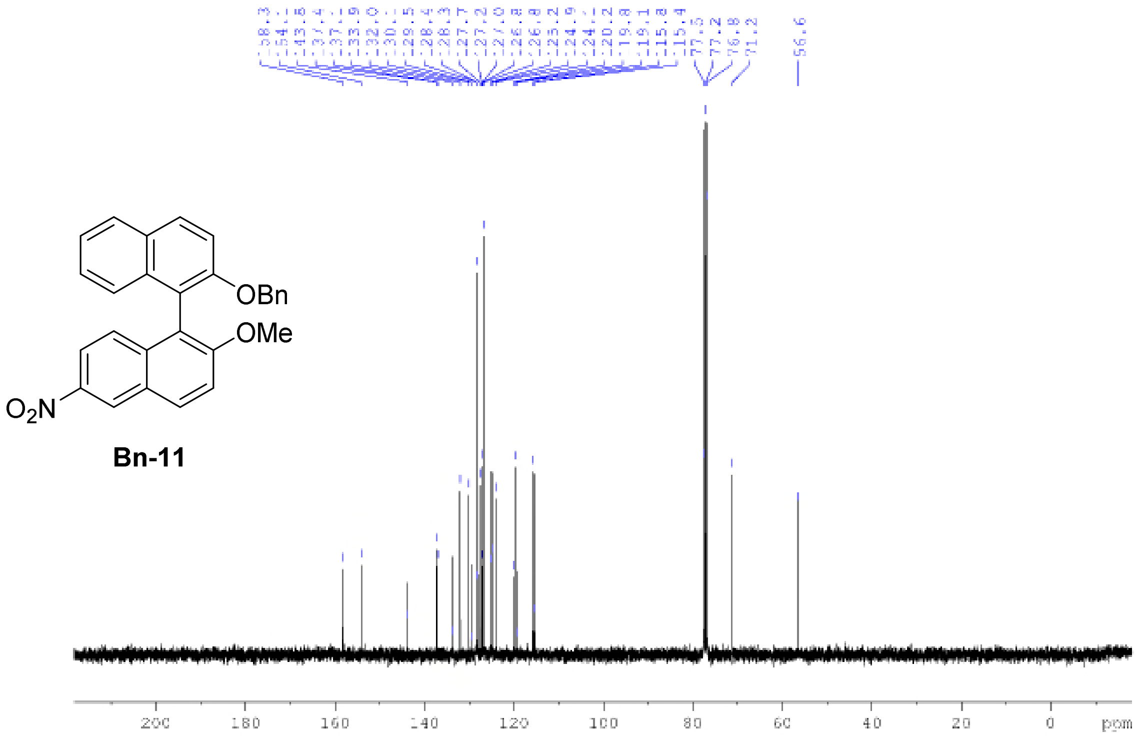
OBn
OMe
O2N
Bn-11
200 180 160 140 120 100 80 60 40 20 0 ppm

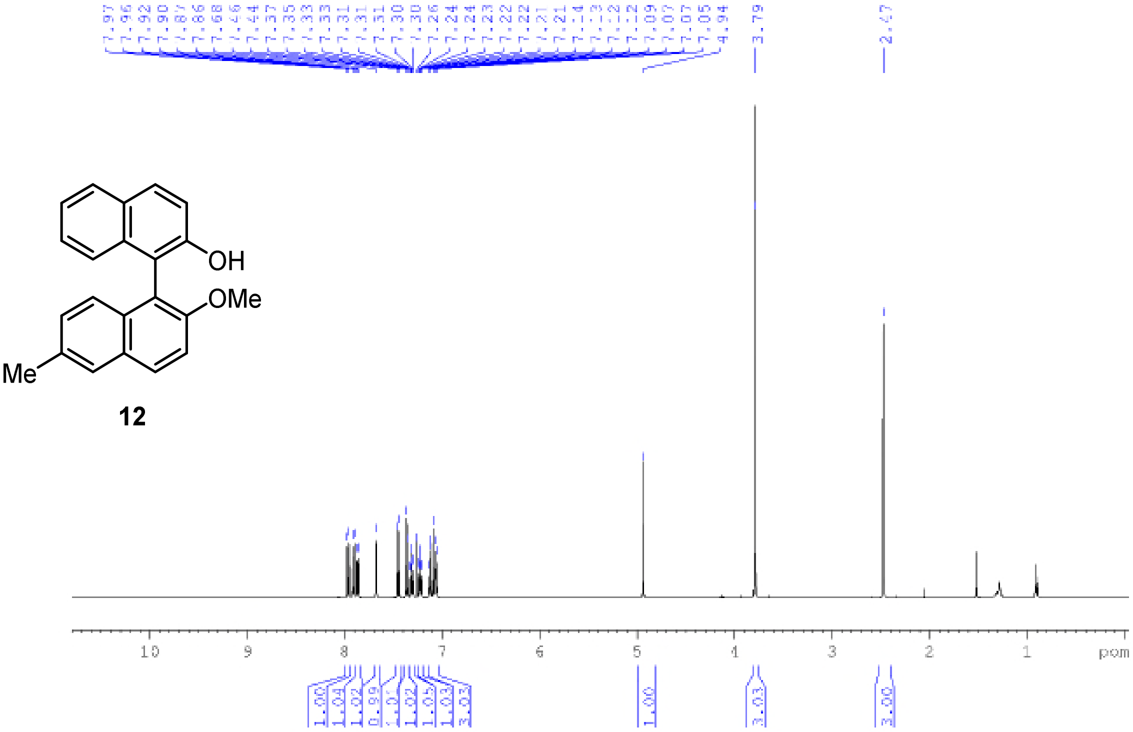
OH
OMe
Me
12

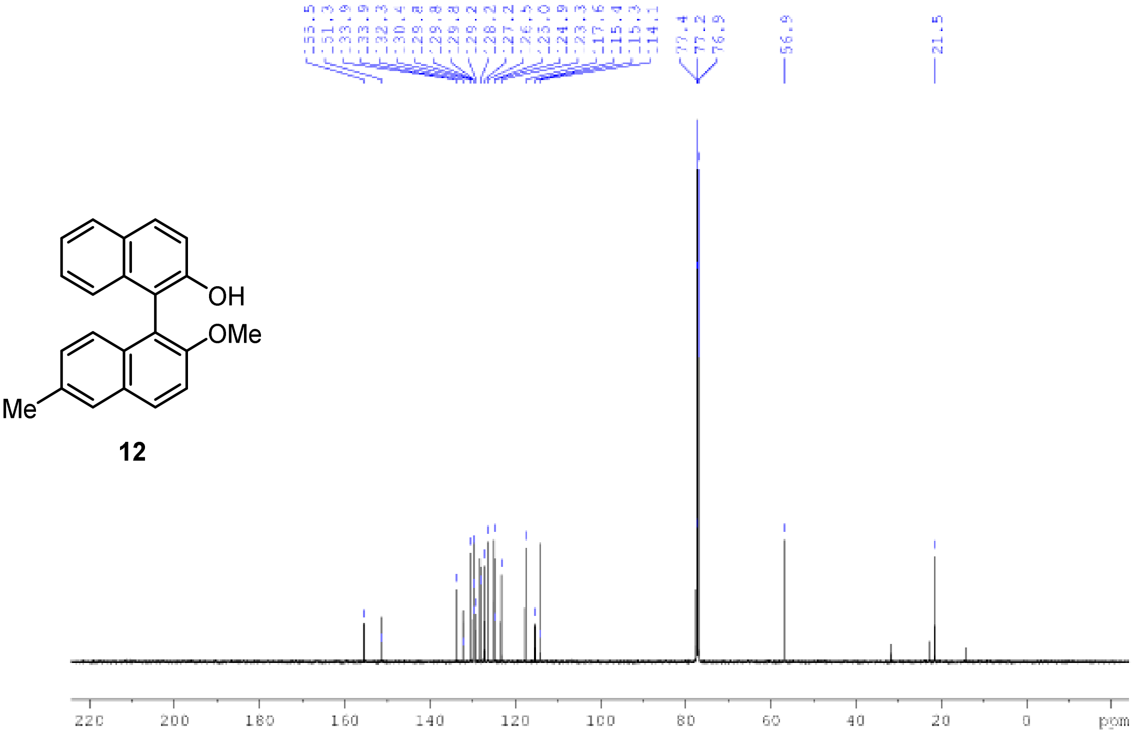
OH
OMe
Me
12

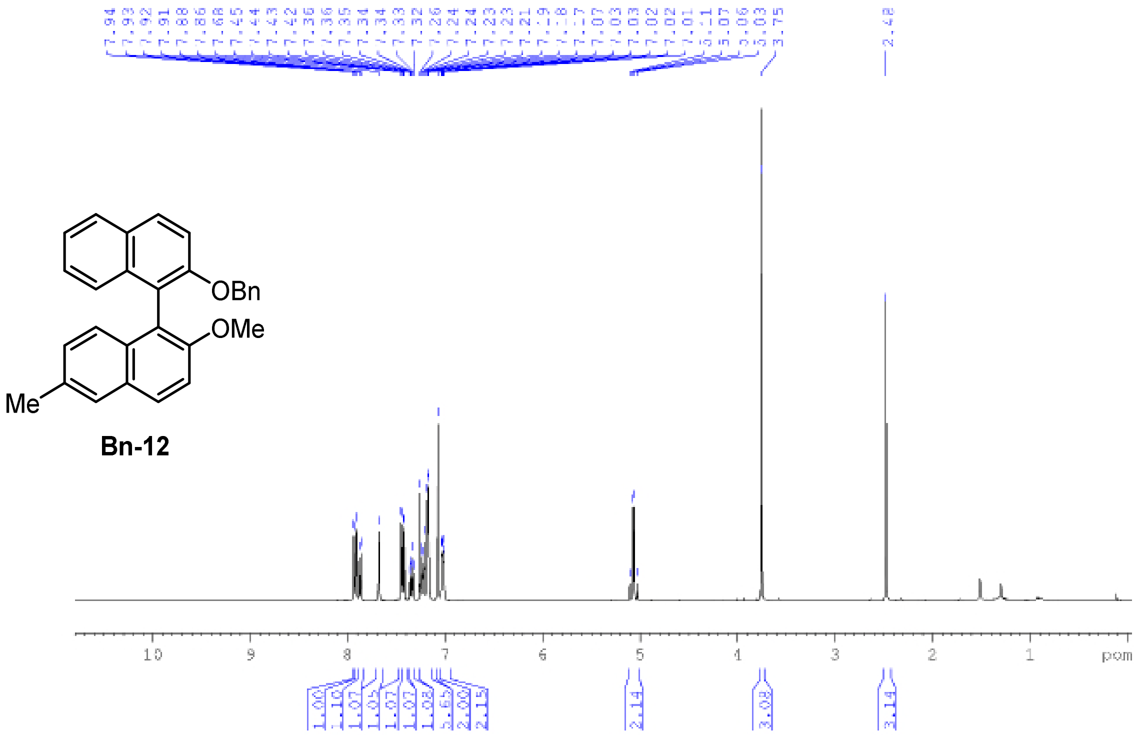
7.94
7.93
7.92
7.91
7.88
7.86
7.68
7.45
7.44
7.43
7.42
7.36
7.35
7.34
7.33
7.32
7.26
7.24
7.23
7.21
7.07
7.03
7.02
7.01
5.07
5.06
5.03
3.75
2.48
OBn
OMe
Me
ppm
1.00
1.07
1.07
1.08
2.00
2.14
3.08
3.14

Bn-12

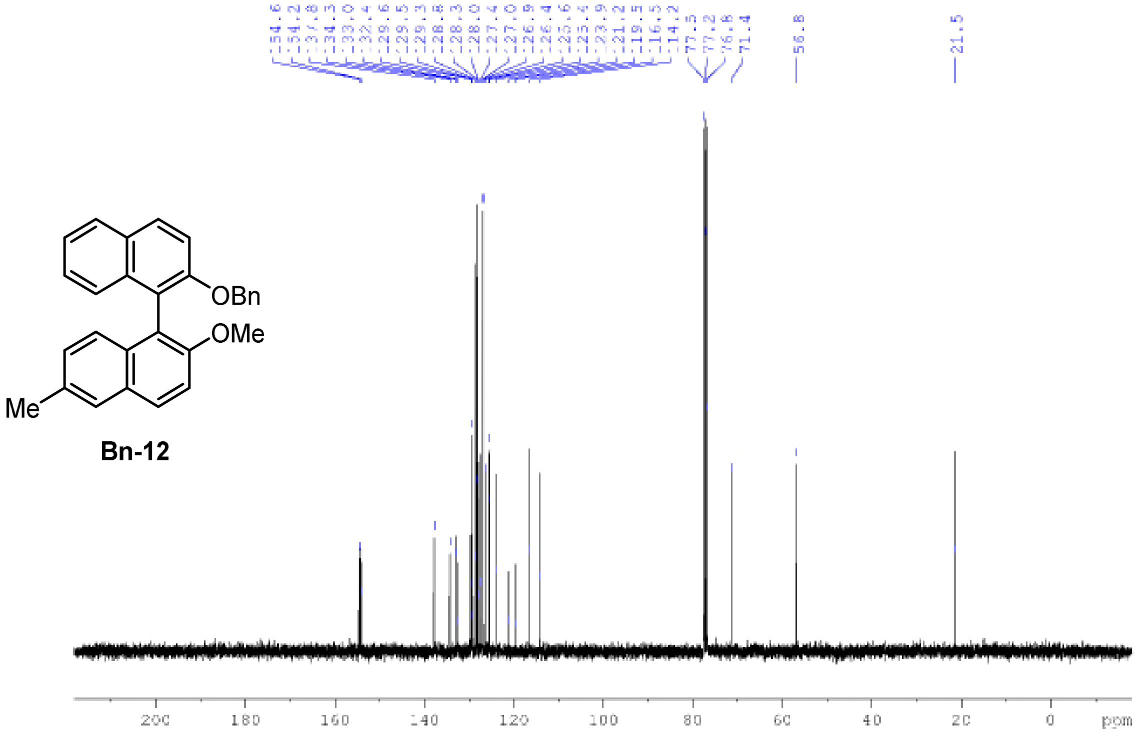
154.6
154.2
137.8
134.3
133.0
132.4
129.6
129.5
129.3
128.8
128.3
128.0
127.4
127.0
126.9
126.4
125.6
125.4
123.9
121.2
119.5
116.5
114.2
77.5
77.2
76.8
71.4
56.8
21.5
OBn
OMe
Me
200
180
160
140
120
100
80
60
40
20
0
ppm

Bn-12

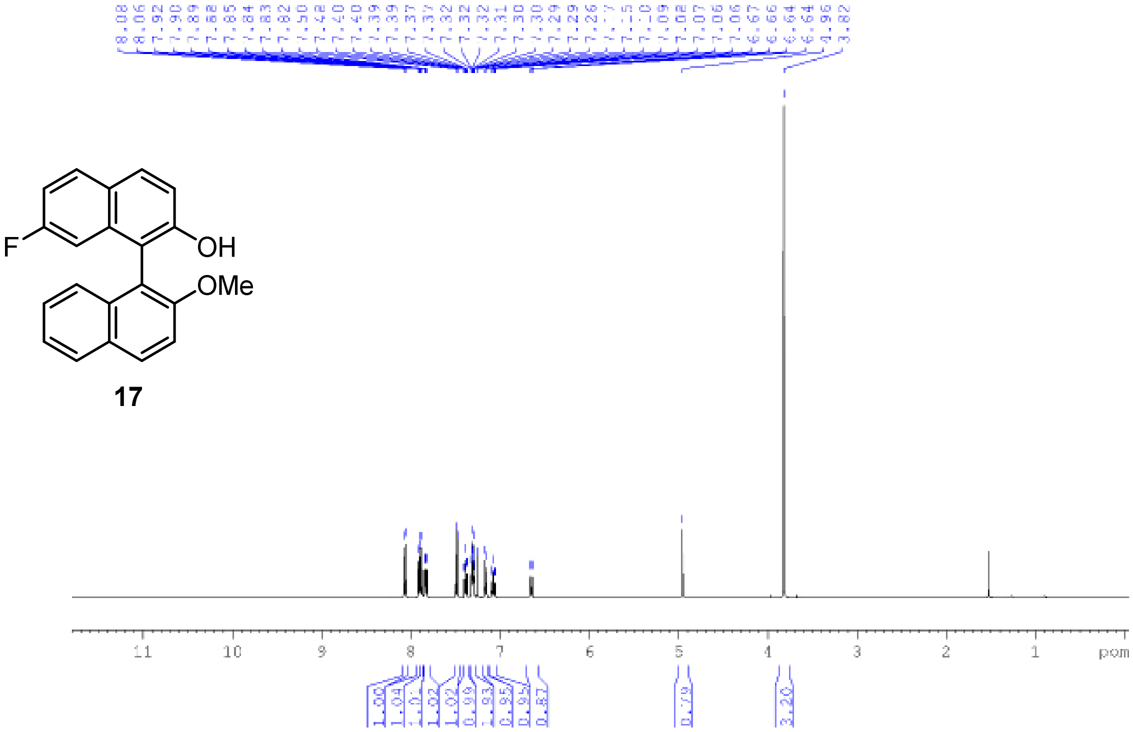

17

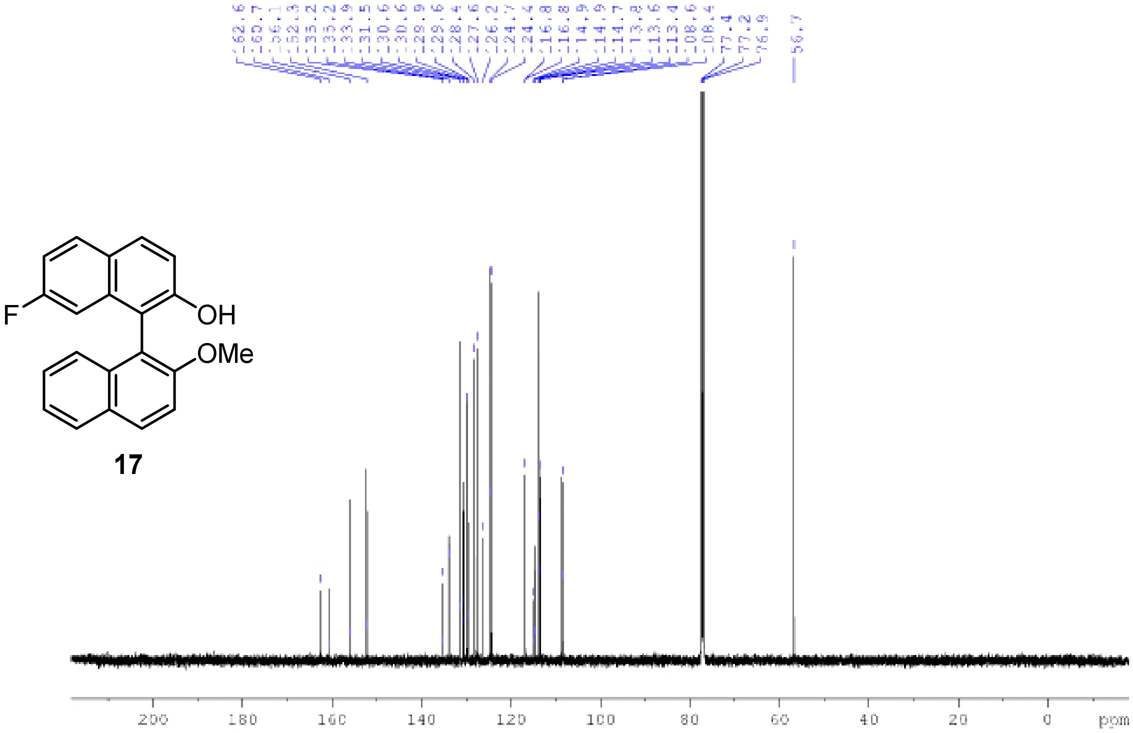

17

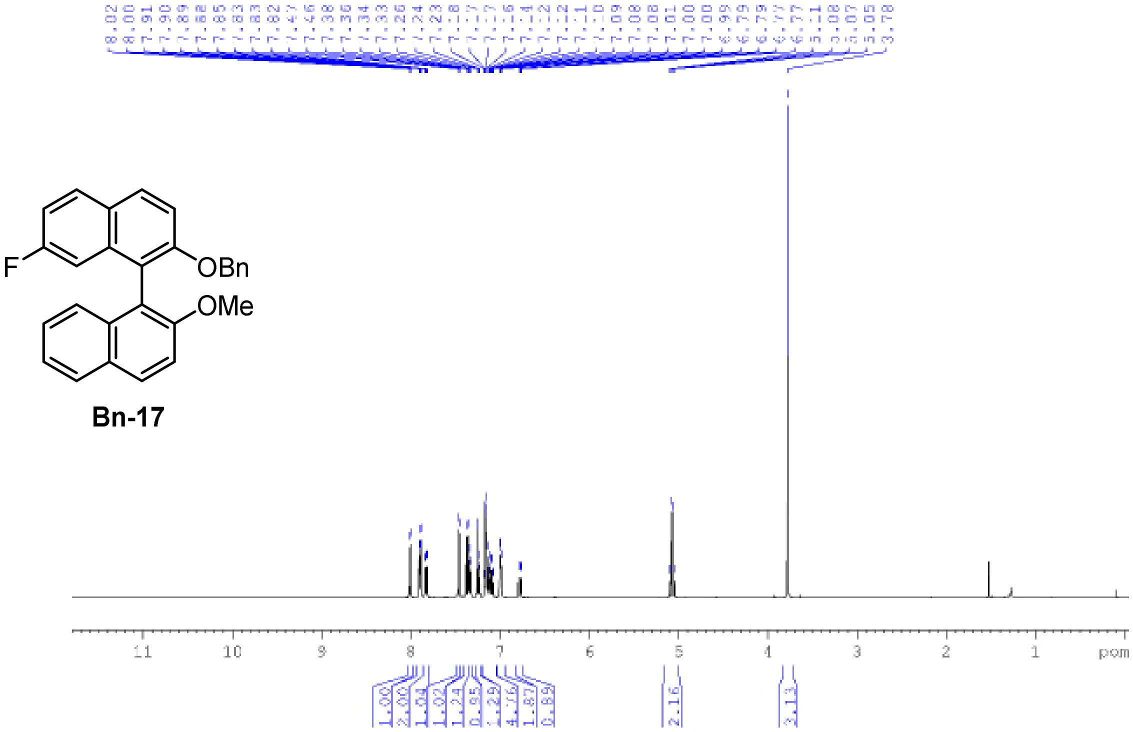
F
OBn
OMe
Bn-17
ppm

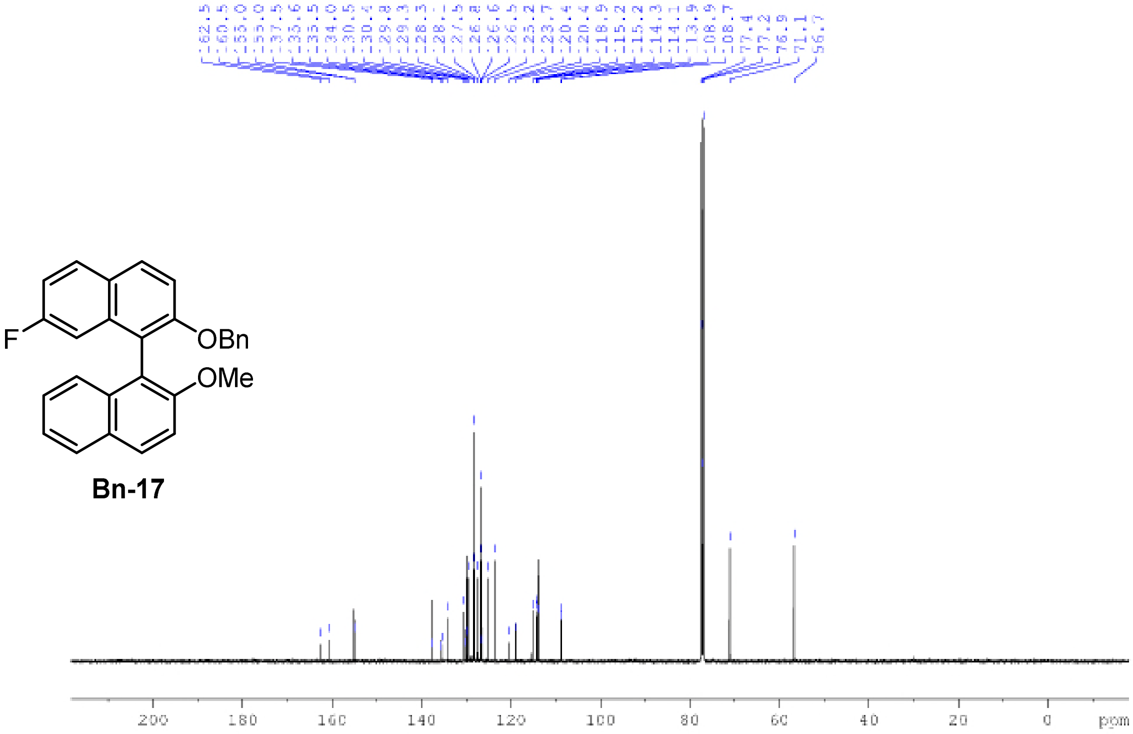
F
OBn
OMe
Bn-17
ppm

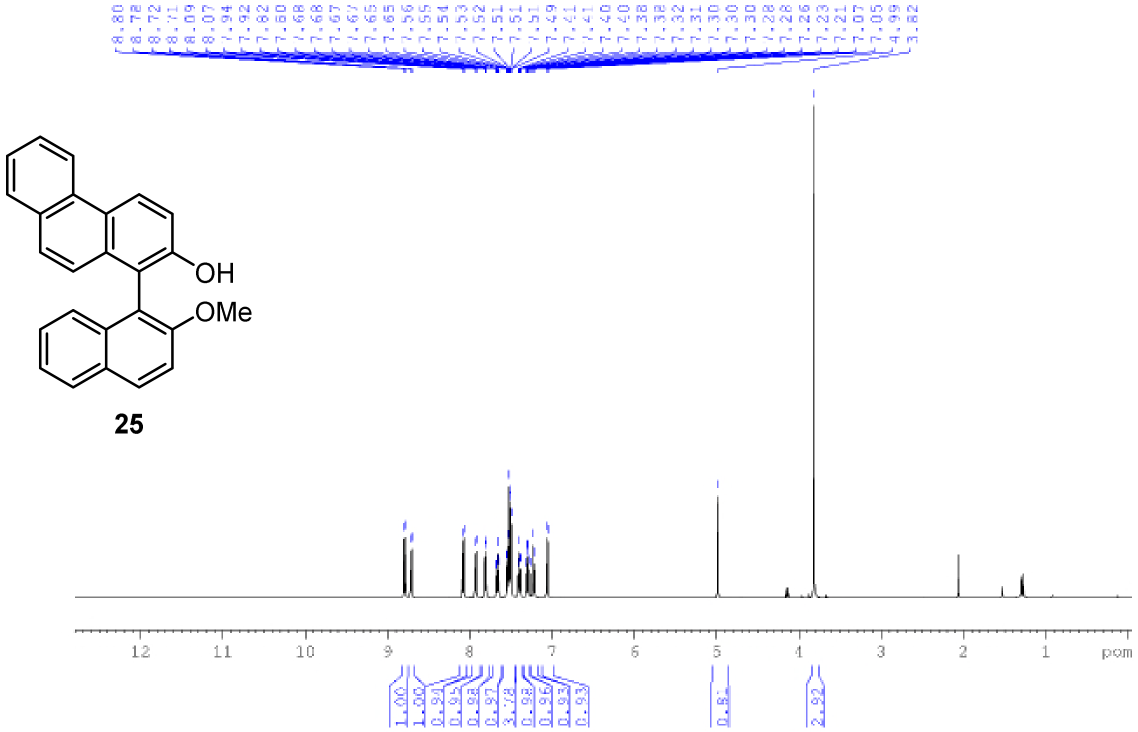
OH
OMe
25
12
11
10
9
8
7
6
5
4
3
2
1
ppm

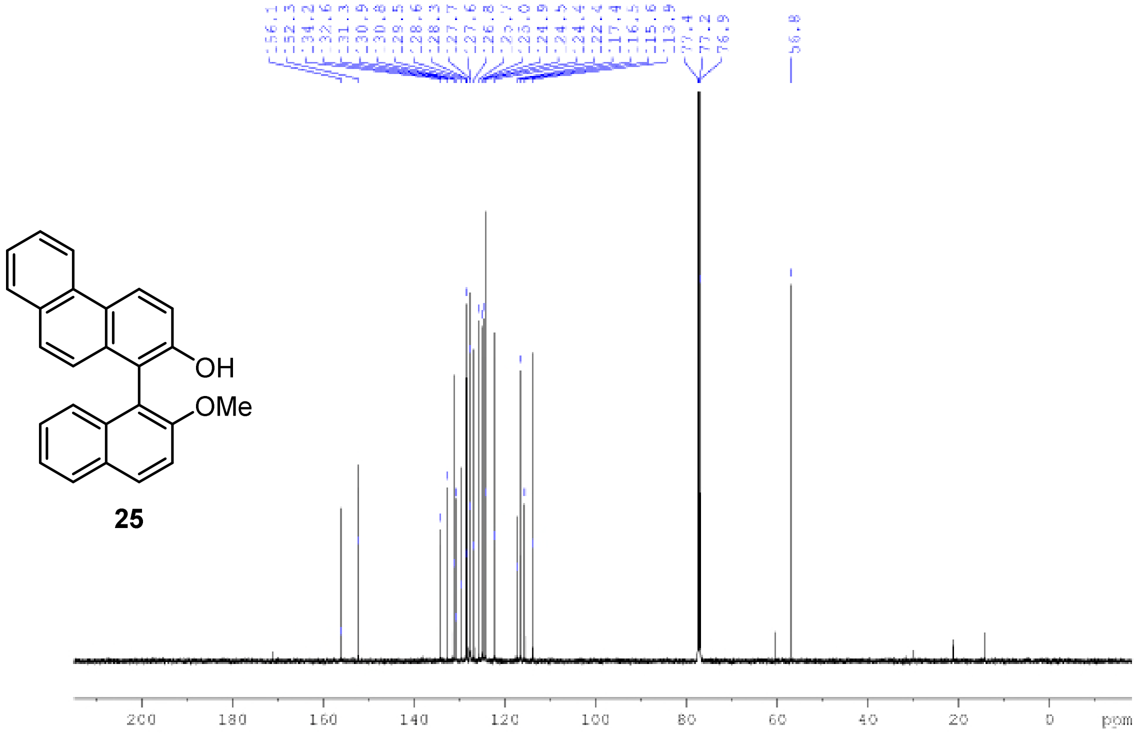
OH
OMe
25
200
180
160
140
120
100
80
60
40
20
0
ppm

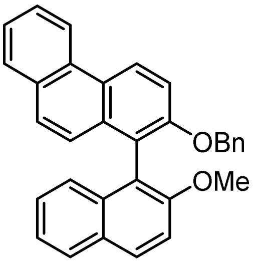

Bn-25

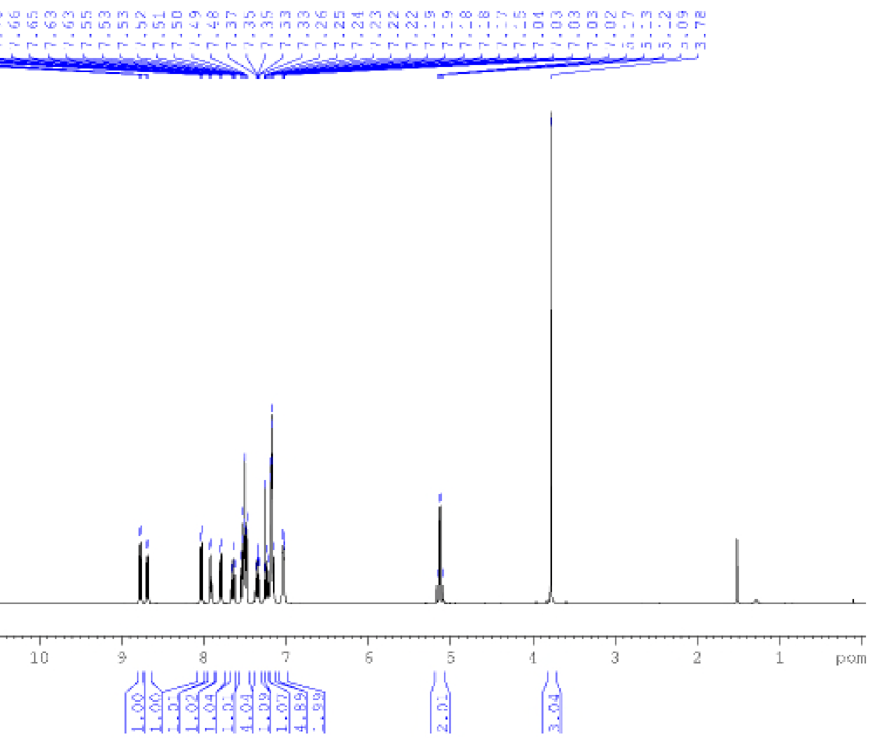

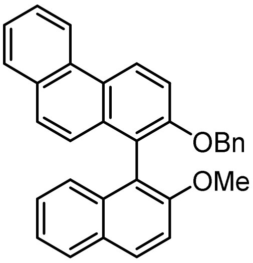

Bn-25

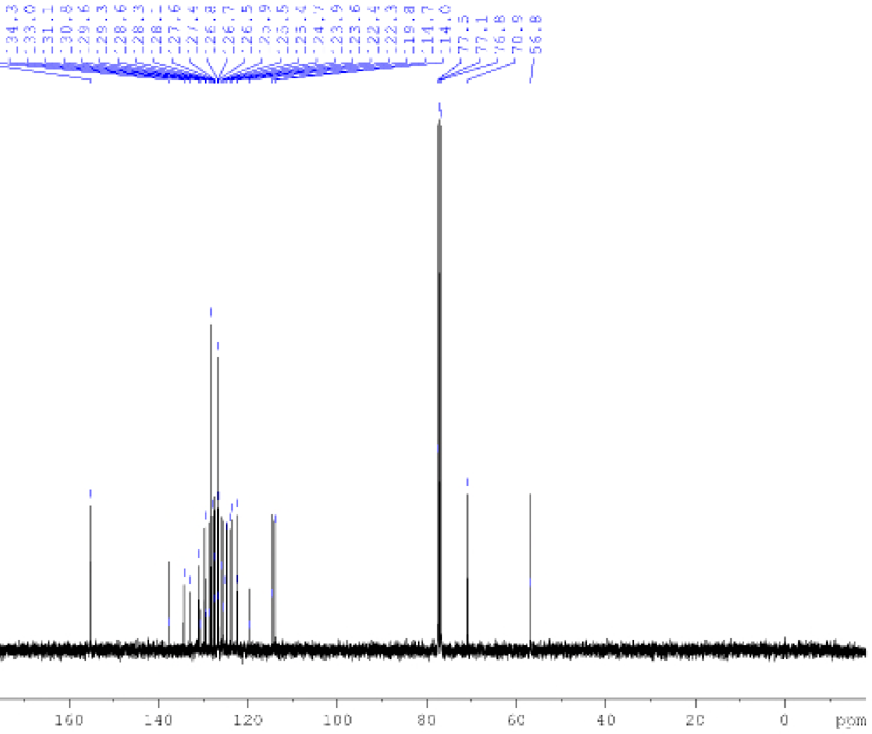

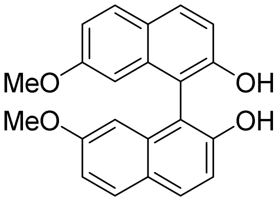
MeO
OH
MeO
OH

61

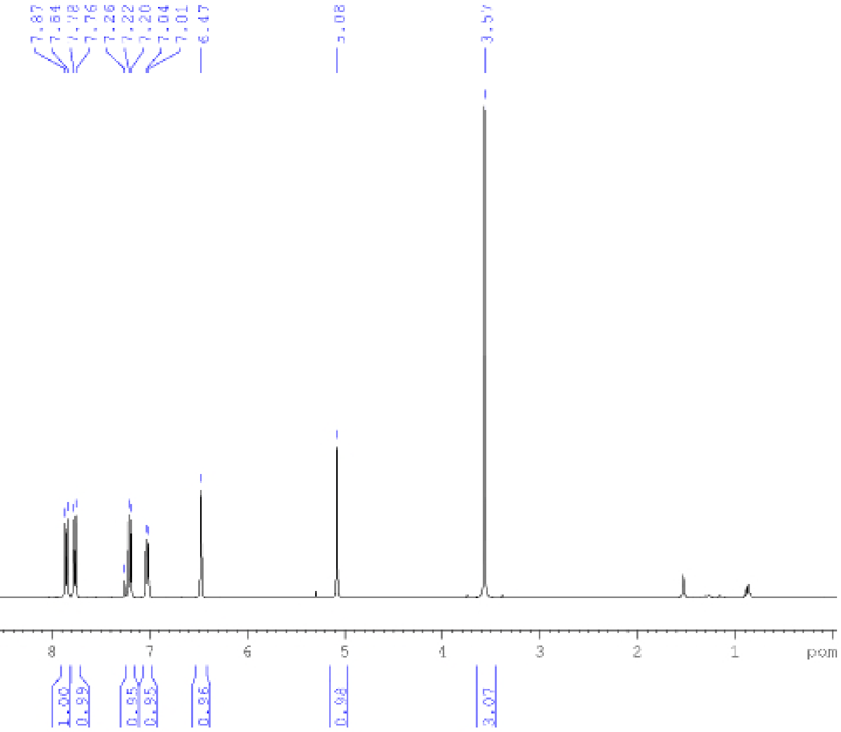
7.87
7.84
7.78
7.76
7.26
7.22
7.20
7.04
7.01
6.47
5.08
3.57
10
9
8
7
6
5
4
3
2
1
ppm
1.00
0.99
0.95
0.95
0.96
0.98
3.07

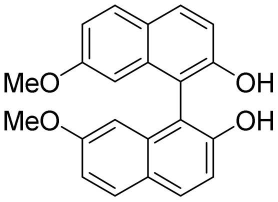
MeO
OH
MeO
OH

61

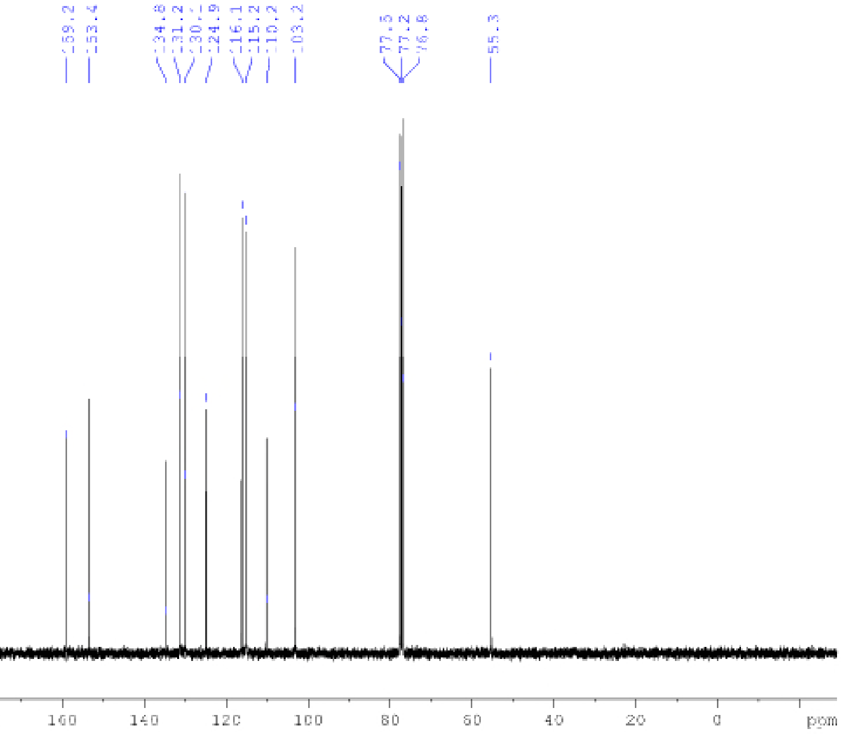
159.2
153.4
134.8
131.2
124.9
116.1
115.2
110.2
103.2
77.5
77.2
76.8
55.3
220
200
180
160
140
120
100
80
60
40
20
0
ppm

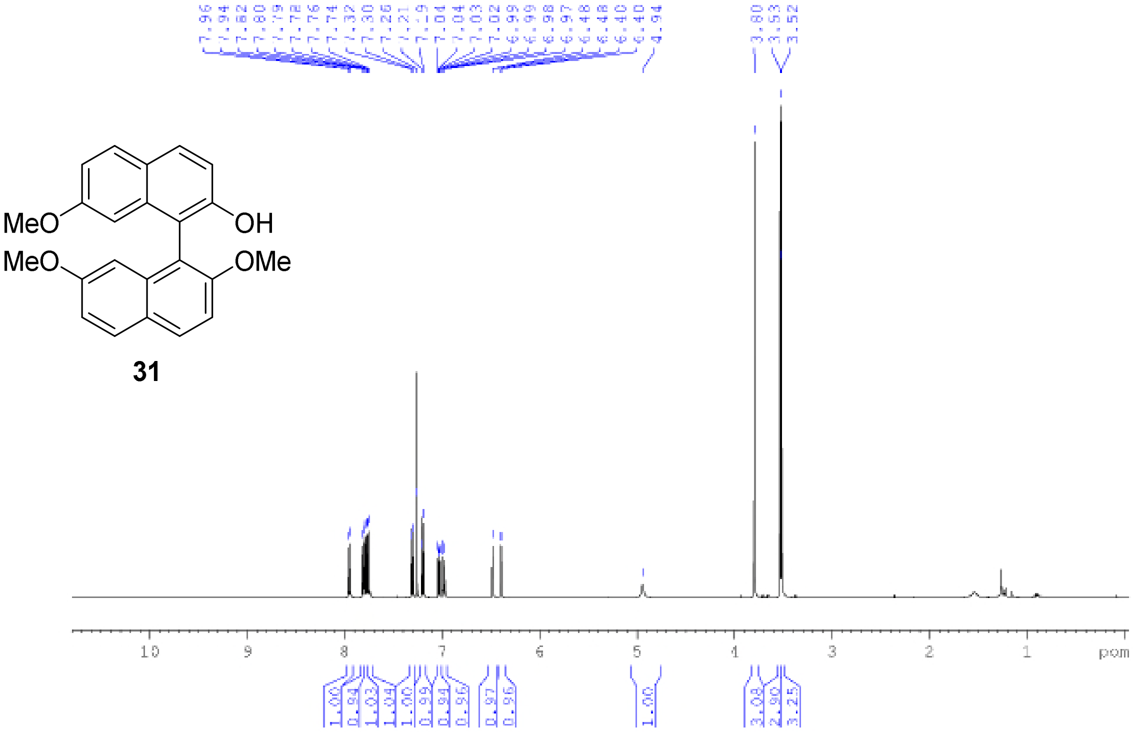

31

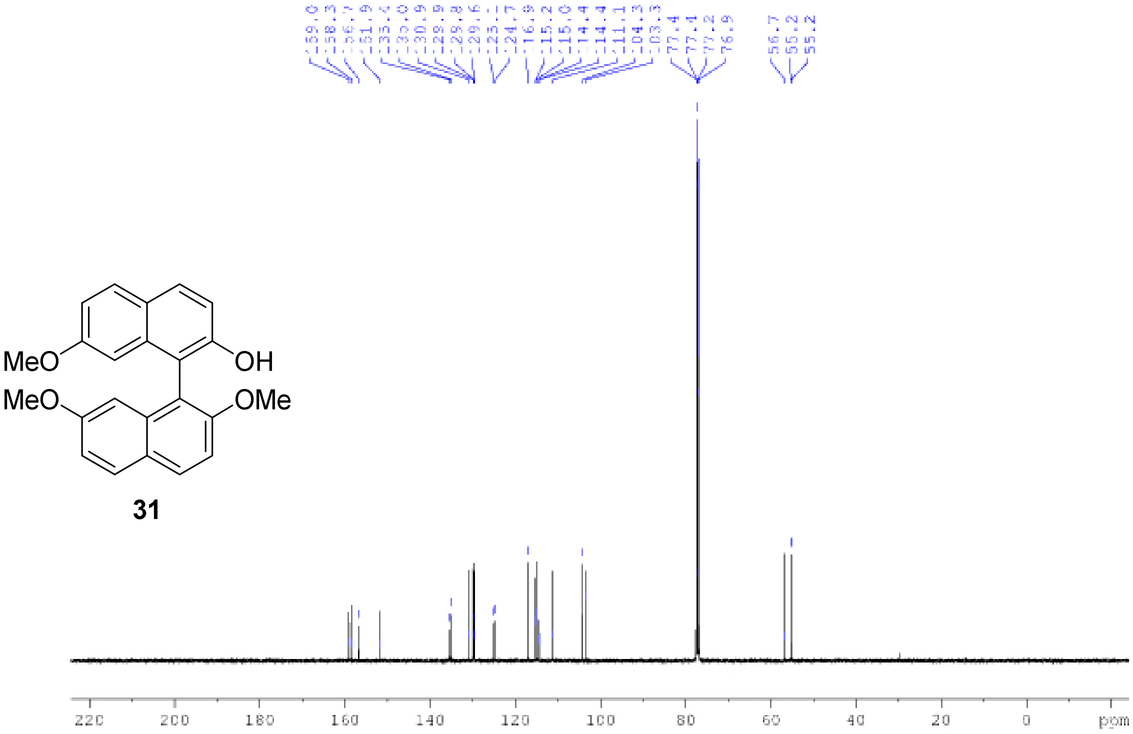

31

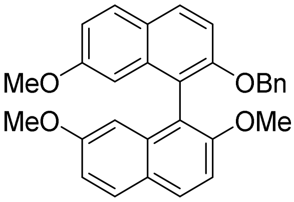

Bn-31

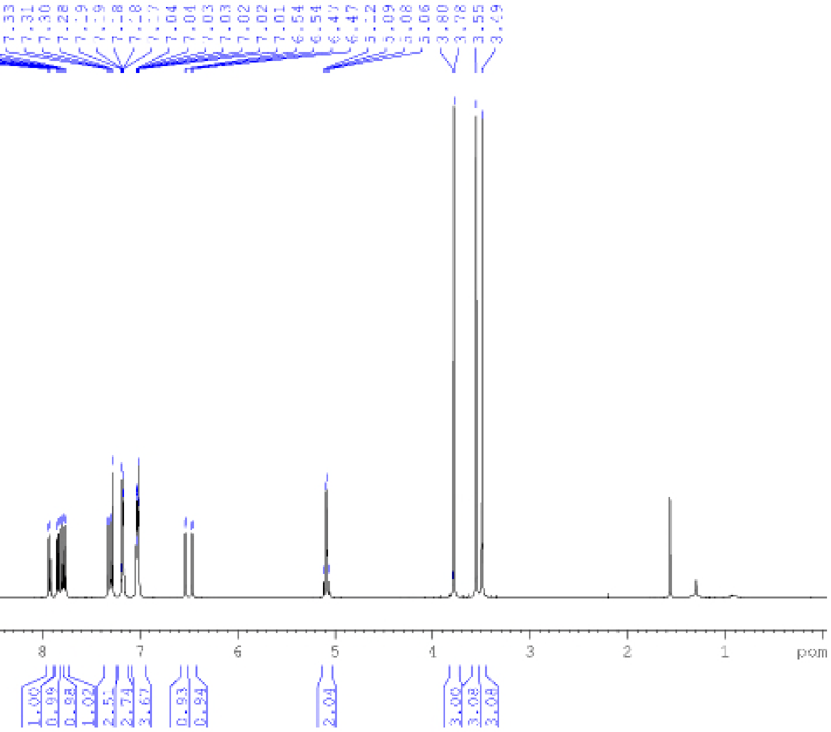

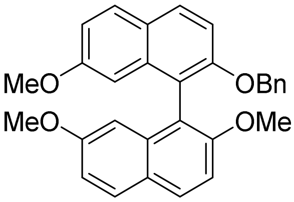

Bn-31

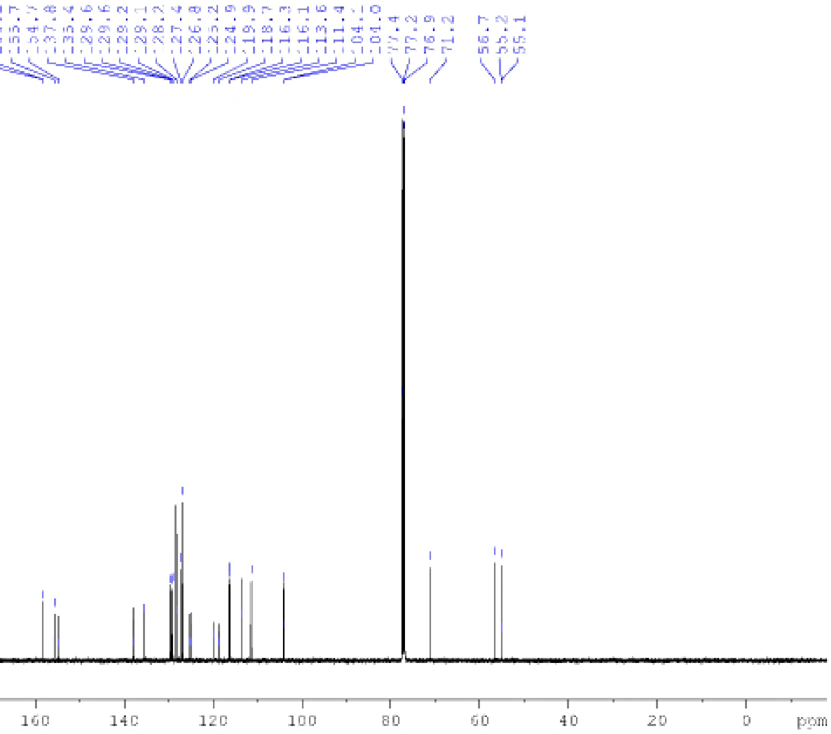

Chiral HPLC: (Chiralpak ADH, 7.5% ^{i}PrOH, 90% hexane, 1.0 mL min^{-1}, λ = 290 nm) τ_R (major) = 25.7 min, τ_R (minor) = 16.6 min.

Racemic

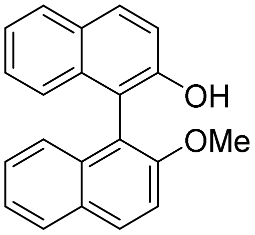

1

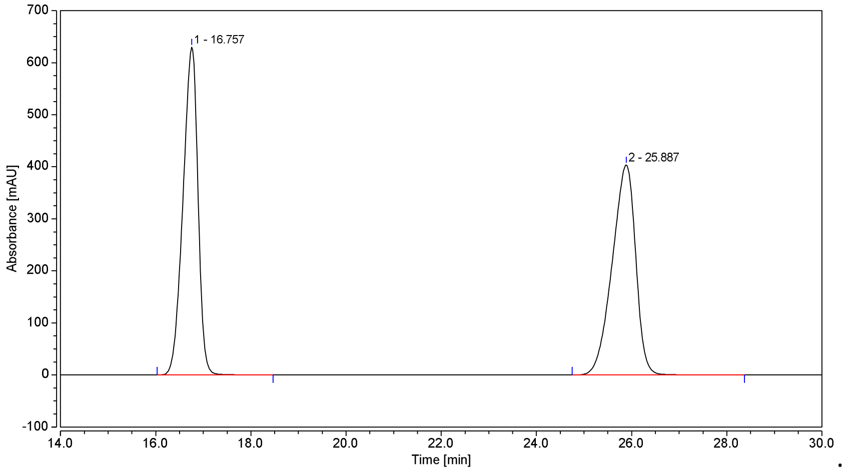

No.	Peak Name	Retention Time	Area	Height	Relative Area
		min	mAU*min	mAU	%
1		16.757	229.643	630.007	49.92
2		25.887	230.356	403.502	50.08
**Total:**			**459.998**	**1033.509**	**100.00**

Asymmetric

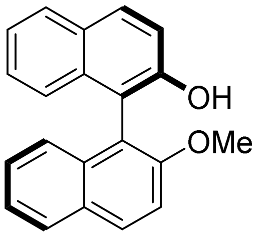

1

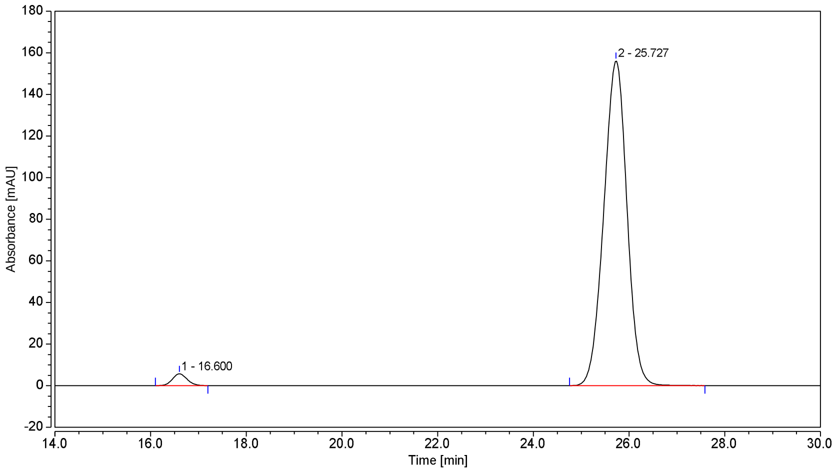

No.	Peak Name	Retention Time	Area	Height	Relative Area
		min	mAU*min	mAU	%
1		16.600	2.041	5.668	2.29
2		25.727	87.190	156.082	97.71
**Total:**			**89.231**	**161.750**	**100.00**

Chiral HPLC: (Chiralpak IC, 1% ^{i}PrOH, 99% hexane, 1.0 mL min^{-1}, λ = 289 nm) τ_R (major) = 8.0 min, τ_R (minor) = 11.8 min.

Racemic.

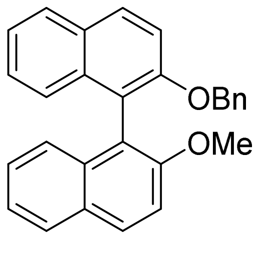

Bn-1

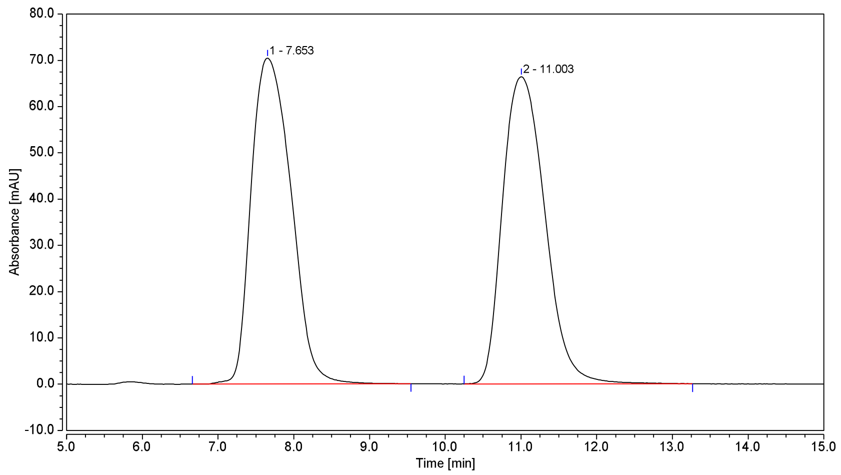

No.	Peak Name	Retention Time	Area	Height	Relative Area
		min	mAU*min	mAU	%
1		7.653	42.993	70.452	49.98
2		11.003	43.028	66.431	50.02
**Total:**			**86.021**	**136.883**	**100.00**

Asymmetric

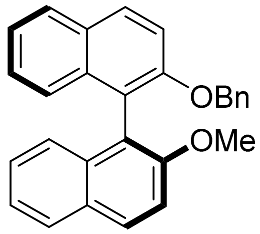

Bn-1

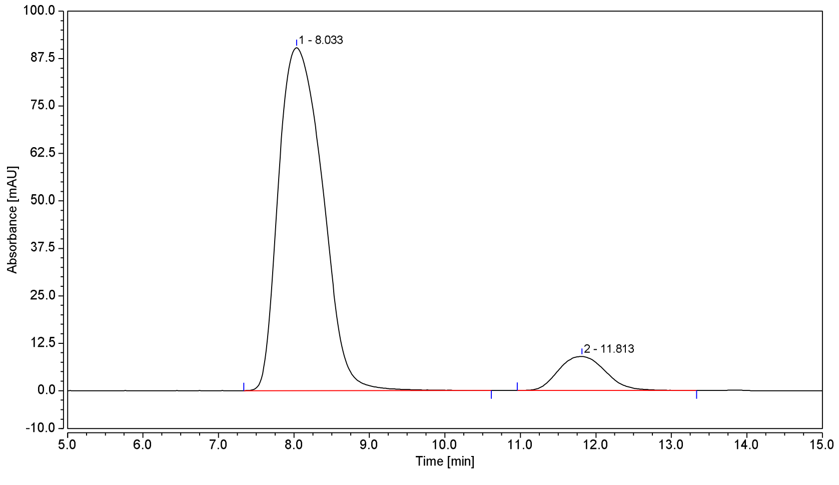

No.	Peak Name	Retention Time	Area	Height	Relative Area
		min	mAU*min	mAU	%
1		8.033	60.986	90.364	90.38
2		11.813	6.489	8.991	9.62
**Total:**			**67.475**	**99.356**	**100.00**

Chiral HPLC: (Chiralpak ADH, 10% ^{i}PrOH, 90% hexane, 1.00 mL min^{-1}, λ = 290 nm) τ_R (major) = 21.1 min, τ_R (minor) = 6.7 min

Racemic

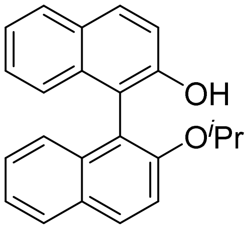

9

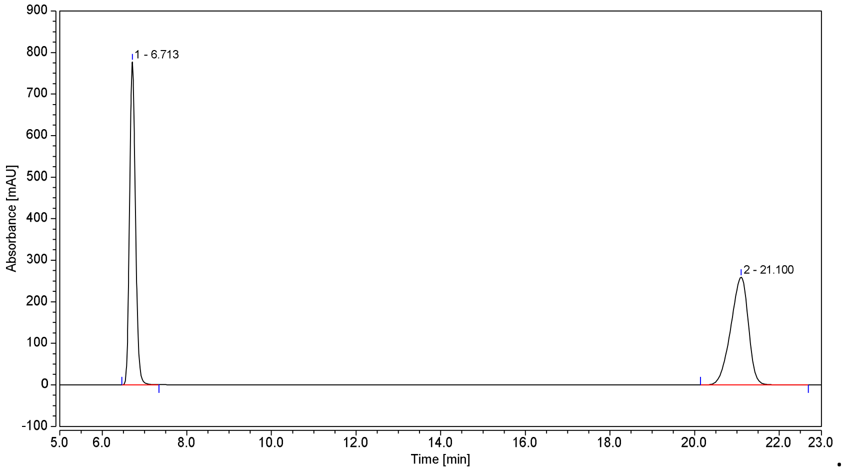

No.	Peak Name	Retention Time	Area	Height	Relative Area
		min	mAU*min	mAU	%
1		6.713	124.143	777.568	49.89
2		21.100	124.675	259.424	50.11
**Total:**			**248.818**	**1036.992**	**100.00**

Asymmetric

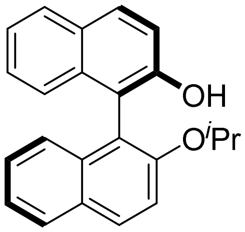

9

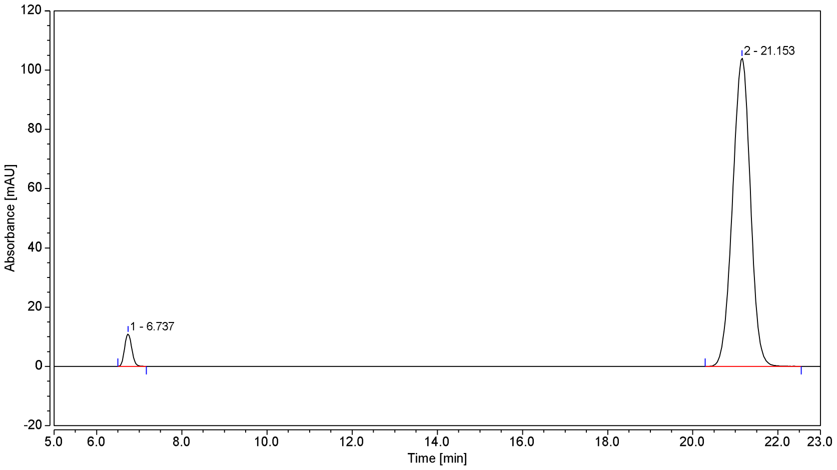

No.	Peak Name	Retention Time	Area	Height	Relative Area
		min	mAU*min	mAU	%
1		6.737	2.135	10.927	4.05
2		21.153	50.573	103.997	95.95
**Total:**			**52.708**	**114.924**	**100.00**

Chiral HPLC: (Chiralpak ADH, 5% ^{i}PrOH, 95% hexane, 1.00 mL min^{-1}, λ = 290nm) τ_R (major) = 5.1 min, τ_R (minor) = 5.6 min

Racemic

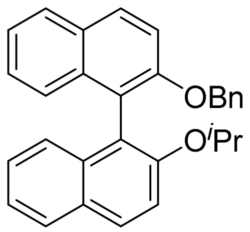

Bn-9

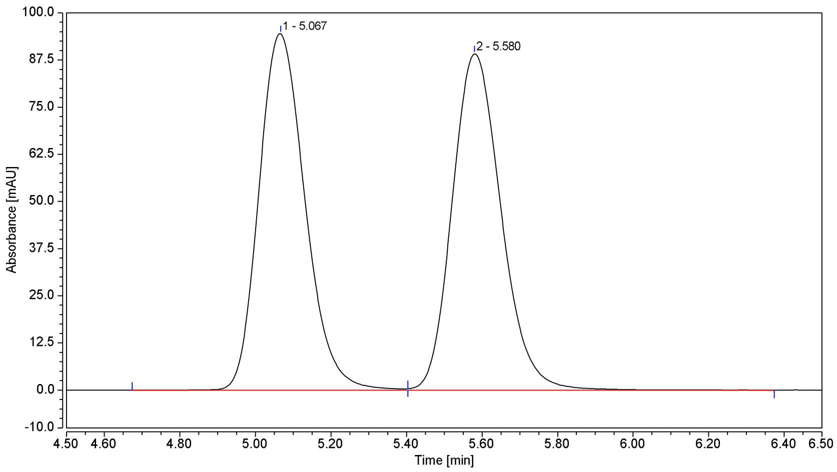

No.	Peak Name	Retention Time	Area	Height	Relative Area
		min	mAU*min	mAU	%
1		5.067	13.320	94.479	49.93
2		5.580	13.360	89.123	50.07
**Total:**			**26.679**	**183.602**	**100.00**

Asymmetric

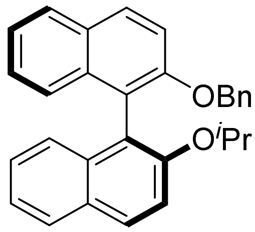

Bn-9

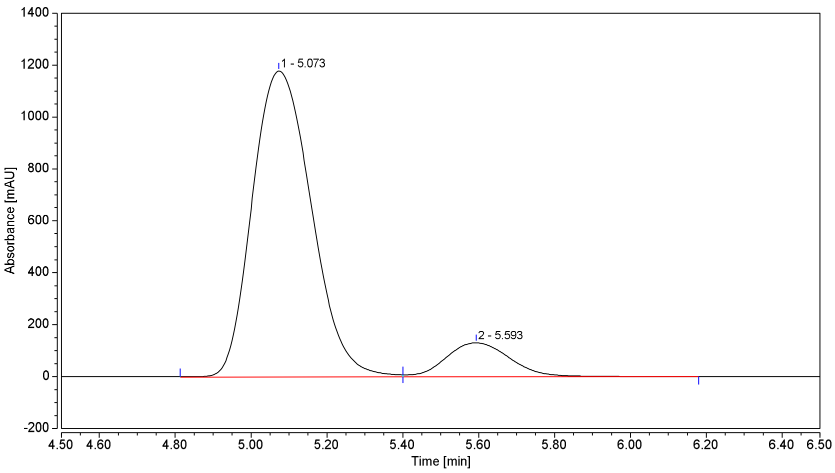

No.	Peak Name	Retention Time	Area	Height	Relative Area
		min	mAU*min	mAU	%
1		5.073	212.451	1178.404	89.27
2		5.593	25.530	130.811	10.73
**Total:**			**237.982**	**1309.215**	**100.00**

Chiral HPLC: (Chiralpak ADH, 20% ^{i}PrOH, 80% hexane, 1.00 mL min^{-1}, λ = 290 nm) τ_R (major) = 35.2 min, τ_R (minor) = 19.5 min

Racemic

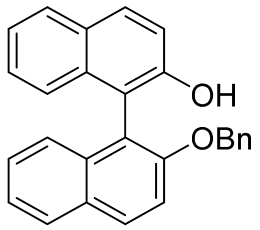

10

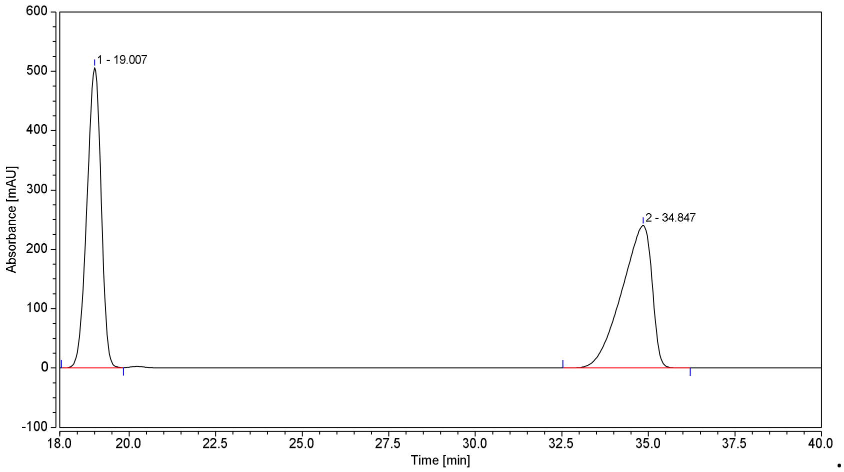

No.	Peak Name	Retention Time	Area	Height	Relative Area
		min	mAU*min	mAU	%
1		19.007	241.636	506.321	49.94
2		34.847	242.227	240.120	50.06
**Total:**			**483.863**	**746.442**	**100.00**

Asymmetric

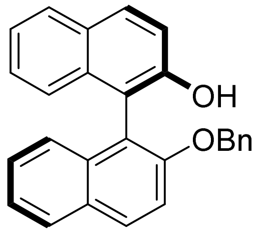

10

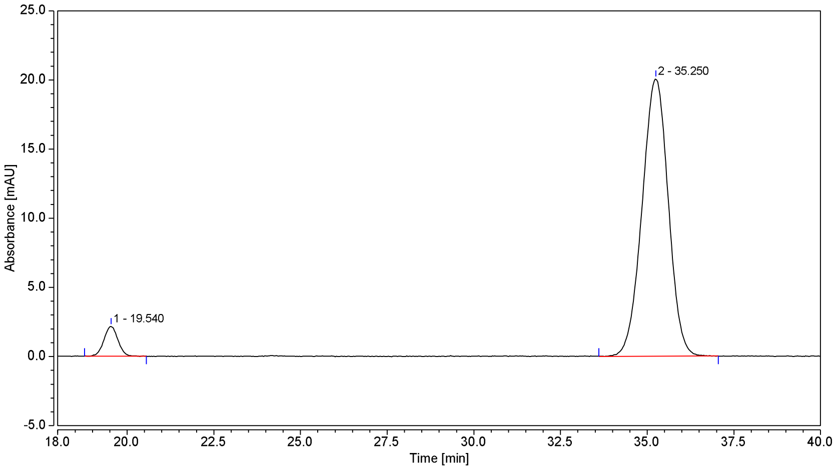

No.	Peak Name	Retention Time	Area	Height	Relative Area
		min	mAU*min	mAU	%
1		19.540	1.000	2.161	5.39
2		35.250	17.563	20.072	94.61
**Total:**			**18.563**	**22.233**	**100.00**

Chiral HPLC: (Chiralpak ADH, 10% ^{i}PrOH, 90% hexane, 1.00 mL min^{-1}, λ = 290 nm) τ_R (major) = 7.3 min, τ_R (minor) = 8.7 min

Racemic

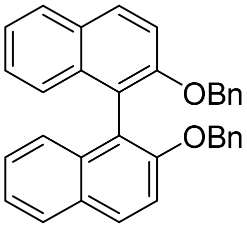

Bn-10

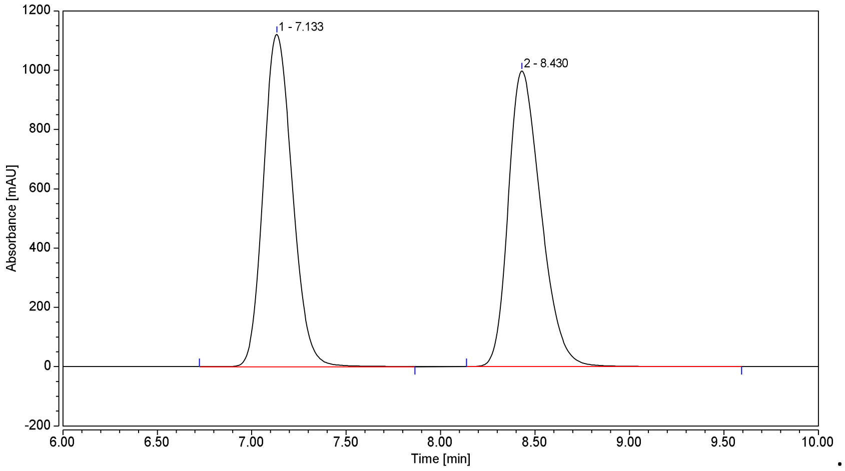

No.	Peak Name	Retention Time	Area	Height	Relative Area
		min	mAU*min	mAU	%
1		7.133	199.858	1121.306	50.00
2		8.430	199.884	997.805	50.00
**Total:**			**399.743**	**2119.111**	**100.00**

Asymmetric

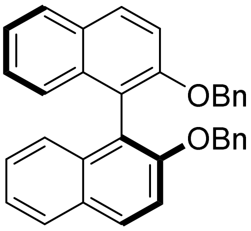

Bn-10

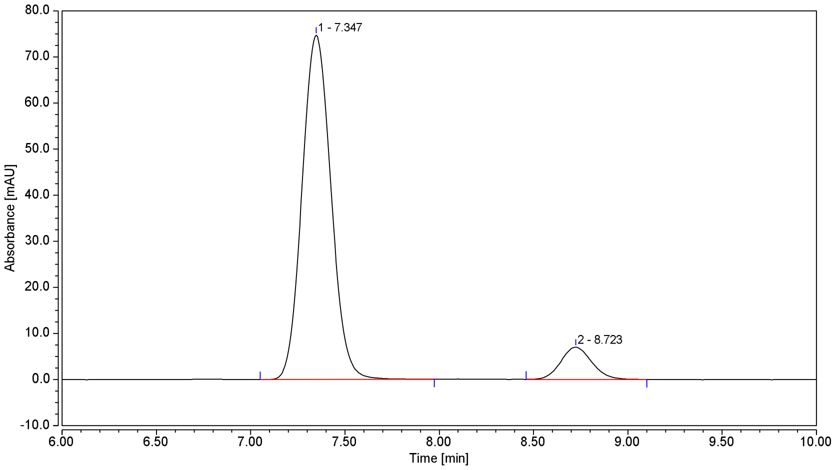

No.	Peak Name	Retention Time	Area	Height	Relative Area
		min	mAU*min	mAU	%
1		7.347	13.264	74.661	90.81
2		8.723	1.343	7.004	9.19
**Total:**			**14.607**	**81.665**	**100.00**

Chiral HPLC: (Chiralpak ADH, 10% ^{i}PrOH, 90% hexane, 1.0 mL min^{-1}, λ = 277 nm) τ_R (major) = 27.4 min, τ_R (minor) = 29.8 min.

Racemic.

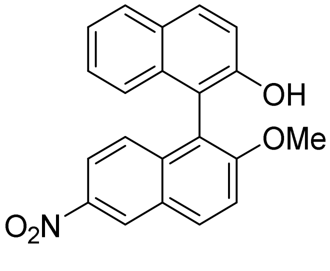

11

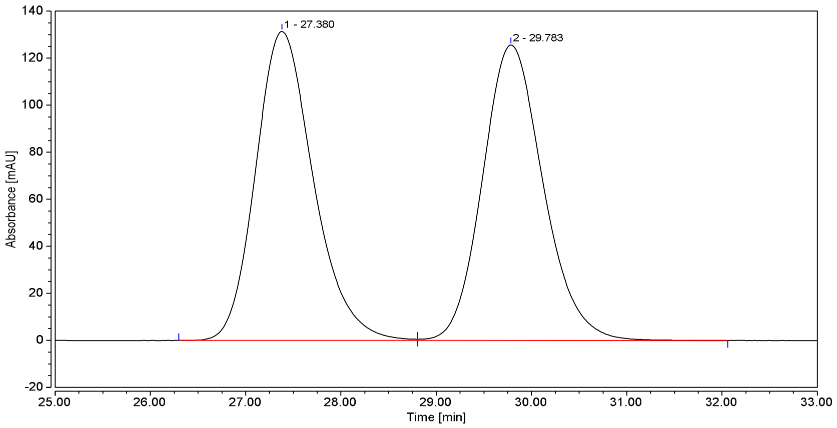

No.	Peak Name	Retention Time	Area	Height	Relative Area
		min	mAU*min	mAU	%
1		27.380	92.599	131.404	49.94
2		29.783	92.812	125.627	50.06
**Total:**			**185.411**	**257.030**	**100.00**

Asymmetric

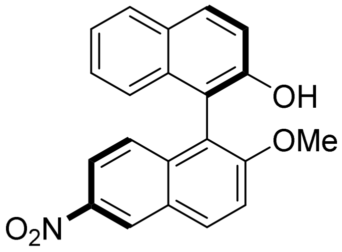

11

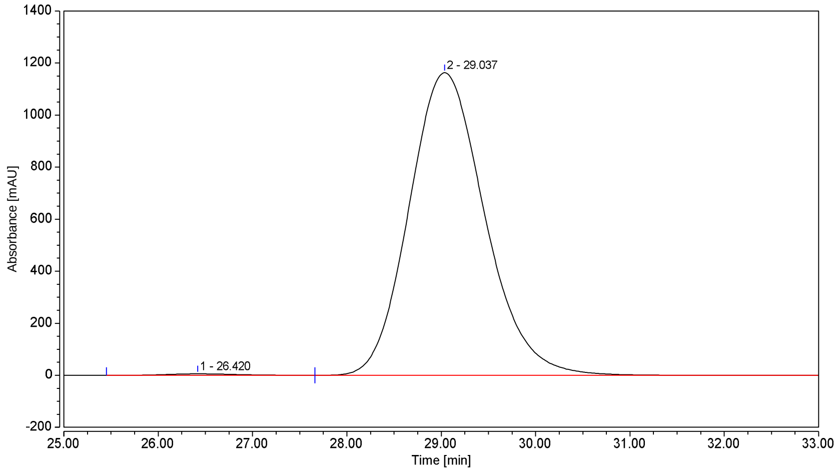

No.	Peak Name	Retention Time	Area	Height	Relative Area
		min	mAU*min	mAU	%
1		26.420	5.501	6.095	0.51
2		29.037	1081.495	1163.649	99.49
**Total:**			**1086.996**	**1169.744**	**100.00**

Chiral HPLC: (Chiralpak ADH, 10% ^{i}PrOH, 90% hexane, 1.0 mL min^{-1}, λ = 279 nm) τ_R (major) = 14.2 min, τ_R (minor) = 26.1 min.

Racemic.

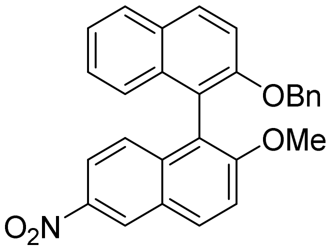

Bn-11

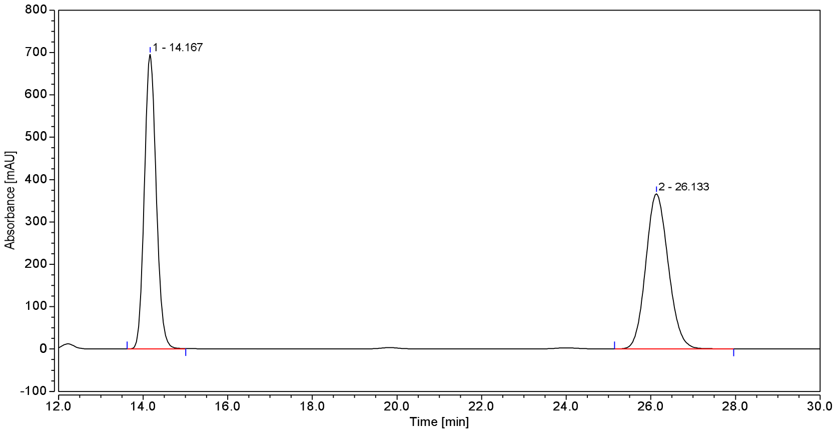

No.	Peak Name	Retention Time	Area	Height	Relative Area
		min	mAU*min	mAU	%
1		14.167	225.271	695.13	49.95
2		26.133	225.718	365.93	50.05
**Total:**			**450.988**	**1061.06**	**100.00**

Asymmetric

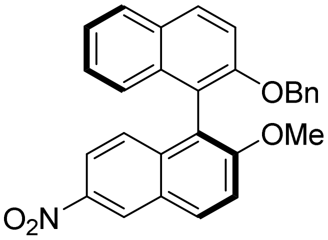

Bn-11

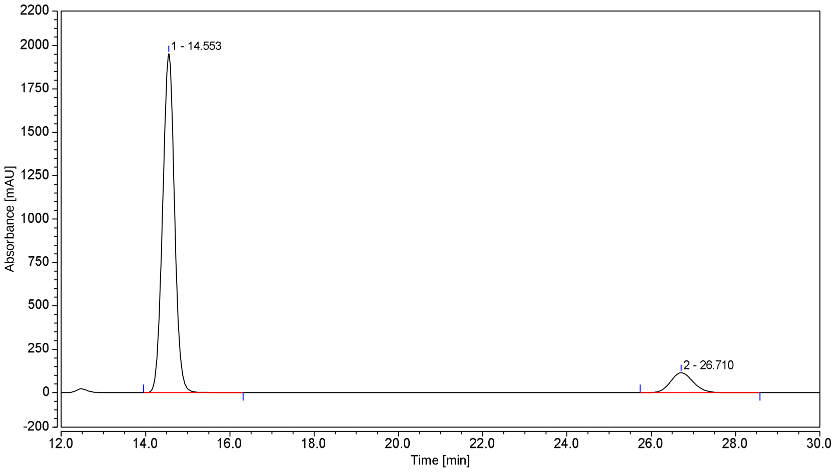

No.	Peak Name	Retention Time	Area	Height	Relative Area
		min	mAU*min	mAU	%
1		14.553	640.989	1953.881	89.95
2		26.710	71.593	114.481	10.05
**Total:**			**712.582**	**2068.363**	**100.00**

Chiral HPLC: (Chiralpak ADH, 10% ^{i}PrOH, 90% hexane, 1.0 mL min^{-1}, λ = 341 nm) τ_R (minor) = 11.5 min, τ_R (major) = 12.9 min.

Racemic

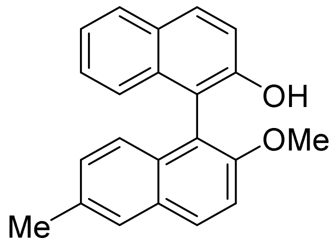

12

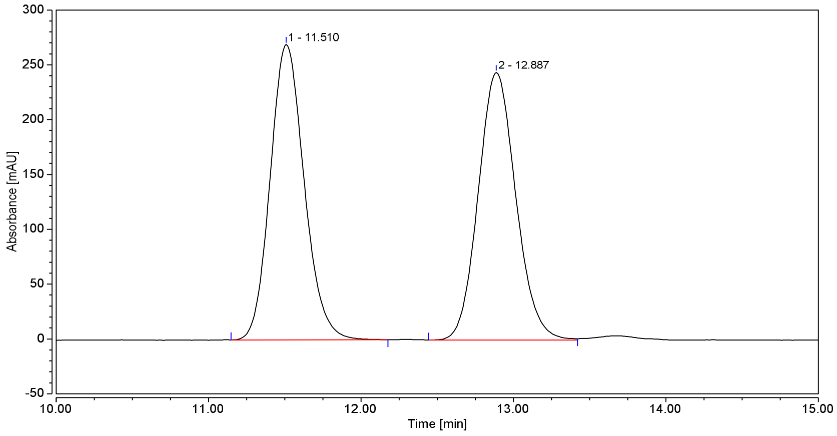

No.	Peak Name	Retention Time	Area	Height	Relative Area
		min	mAU*min	mAU	%
1		11.510	69.672	269.374	49.95
2		12.887	69.824	243.978	50.05
**Total:**			**139.496**	**513.353**	**100.00**

Asymmetric

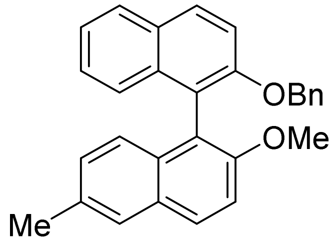

12

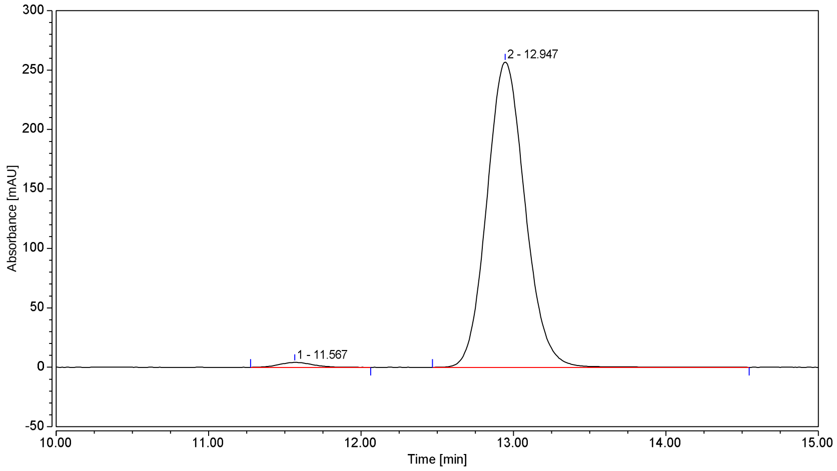

No.	Peak Name	Retention Time	Area	Height	Relative Area
		min	mAU*min	mAU	%
1		11.567	1.060	4.097	1.40
2		12.947	74.538	256.883	98.60
**Total:**			**75.598**	**260.980**	**100.00**

Chiral HPLC: (Chiralpak IC, 1% ^{i}PrOH, 99% hexane, 1.0 mL min^{-1}, λ = 295nm) τ_R (major) = 7.8 min, τ_R (minor) = 12.2 min.

Racemic

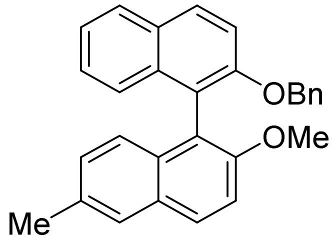

Bn-12

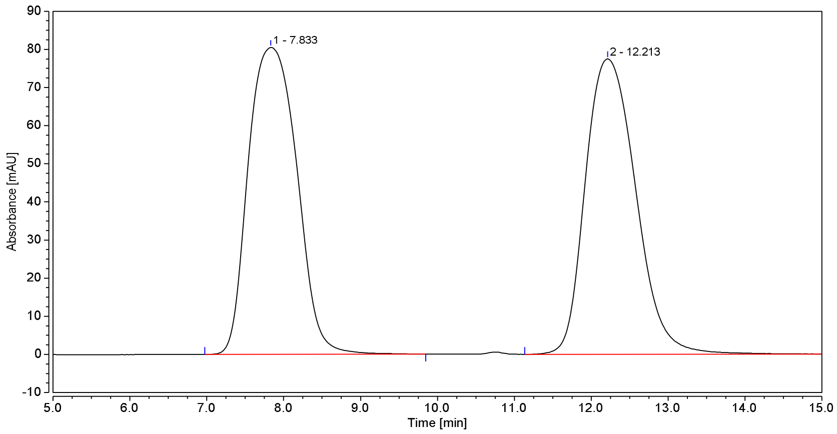

No.	Peak Name	Retention Time	Area	Height	Relative Area
		min	mAU*min	mAU	%
1		7.833	58.952	80.479	49.98
2		12.213	59.000	77.484	50.02
**Total:**			**117.952**	**157.963**	**100.00**

Asymmetric

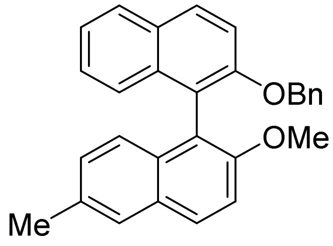

Bn-12

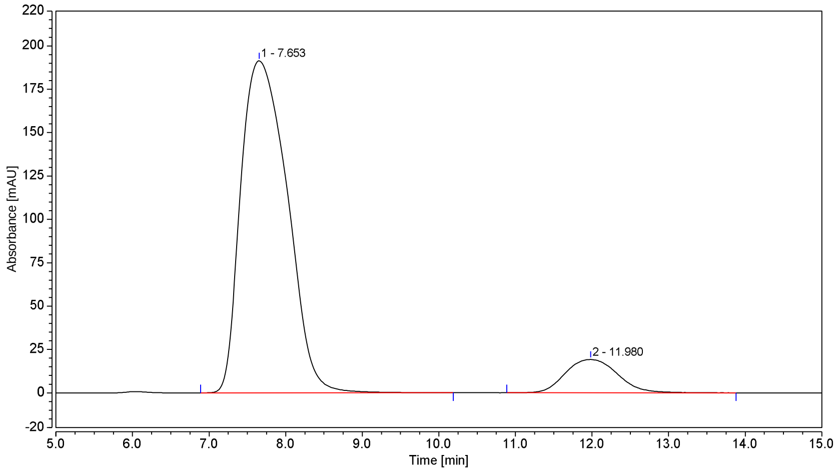

No.	Peak Name	Retention Time	Area	Height	Relative Area
		min	mAU*min	mAU	%
1		7.653	134.553	191.393	89.83
2		11.980	15.241	19.212	10.17
**Total:**			**149.794**	**210.605**	**100.00**

Chiral HPLC: (Chiralpak ADH, 10% ^{i}PrOH, 90% hexane, 1.0 mL min^{-1}, λ = 295 nm) τ_R (major) = 13.7 min, τ_R (minor) = 11.6 min.

<u>**Racemic**</u>.

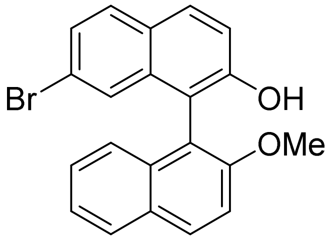

13

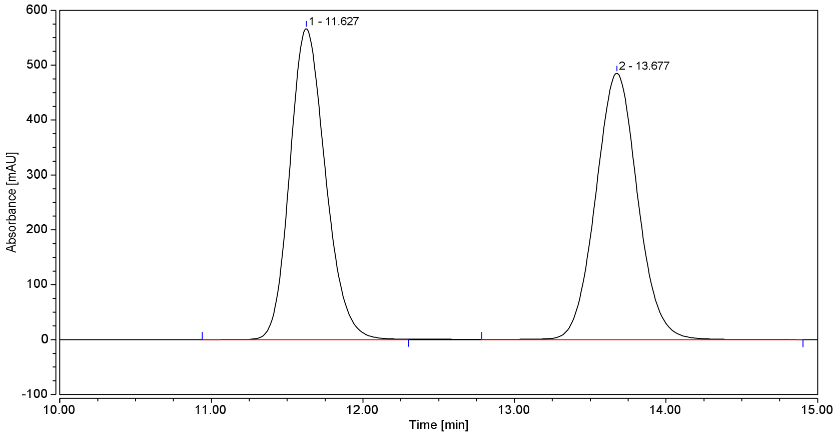

No.	Peak Name	Retention Time	Area	Height	Relative Area
		min	mAU*min	mAU	%
1		11.627	154.798	566.694	49.79
2		13.677	156.097	485.369	50.21
**Total:**			**310.894**	**1052.063**	**100.00**

<u>**Asymmetric**</u>

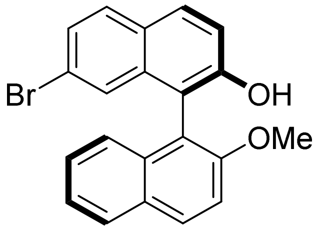

13

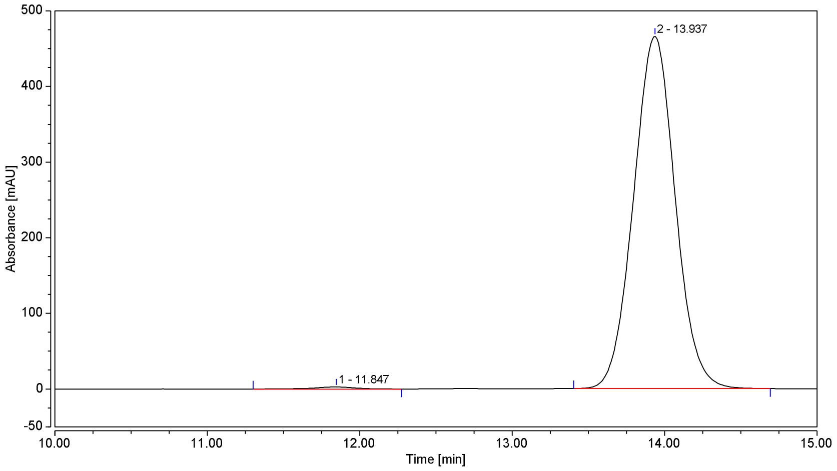

No.	Peak Name	Retention Time	Area	Height	Relative Area
		min	mAU*min	mAU	%
1		11.847	0.839	2.734	0.57
2		13.937	147.445	465.685	99.43
**Total:**			**148.284**	**468.418**	**100.00**

Chiral HPLC: (Chiralpak ADH, 10% ^{i}PrOH, 98% hexane, 1.0 mL min^{-1}, λ = 295 nm) τ_R (major) = 8.7 min, τ_R (minor) = 7.5 min.

Racemic

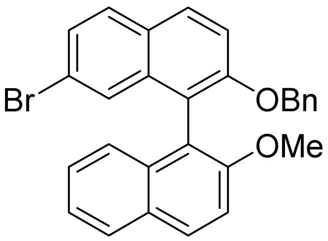

Bn-13

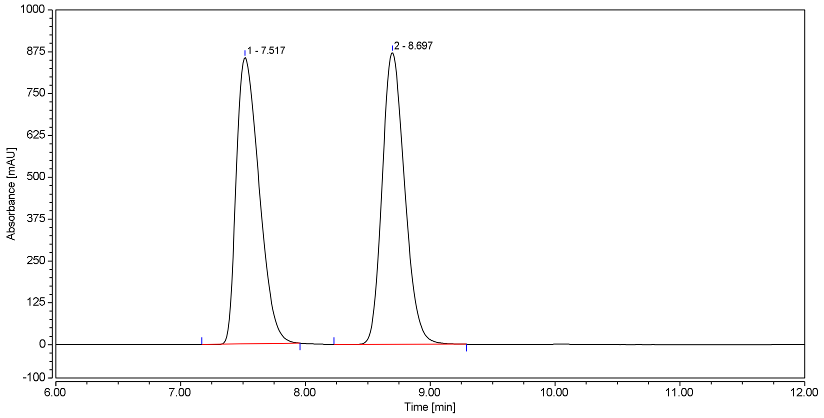

No.	Peak Name	Retention Time	Area	Height	Relative Area
		min	mAU*min	mAU	%
1		7.517	180.898	855.996	49.84
2		8.697	182.024	871.941	50.16
**Total:**			**362.922**	**1727.937**	**100.00**

Asymmetric

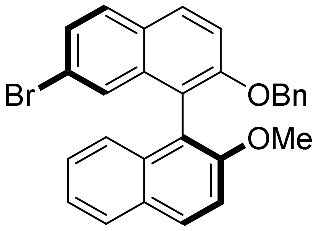

Bn-13

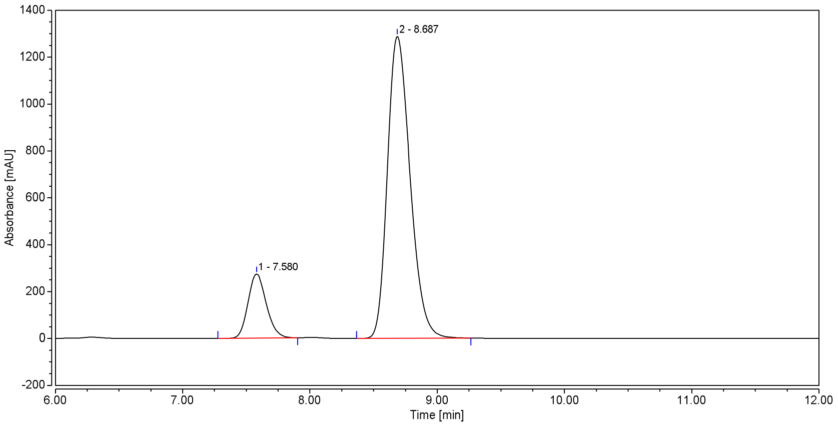

No.	Peak Name	Retention Time	Area	Height	Relative Area
		min	mAU*min	mAU	%
1		7.580	45.946	274.209	14.70
2		8.687	266.507	1289.281	85.30
**Total:**			**312.453**	**1563.489**	**100.00**

Chiral HPLC: (Chiralpak ADH, 15% ^{i}PrOH, 85% hexane, 1.00 mL min^{-1}, λ = 290 nm) τ_R (major) = 17.9 min, τ_R (minor) = 13.5 min

Racemic

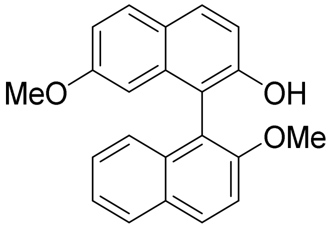

14

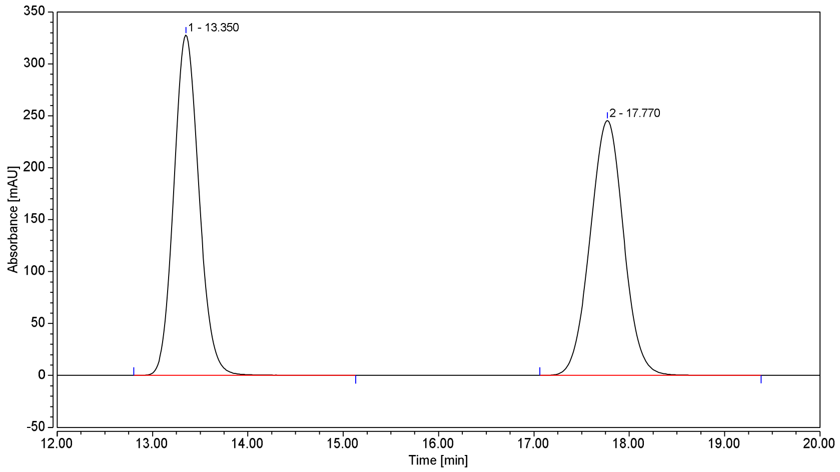

No.	Peak Name	Retention Time	Area	Height	Relative Area
		min	mAU*min	mAU	%
1		13.350	100.173	327.807	50.05
2		17.770	99.983	245.506	49.95
**Total:**			**200.156**	**573.312**	**100.00**

Asymmetric

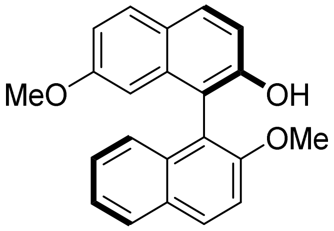

14

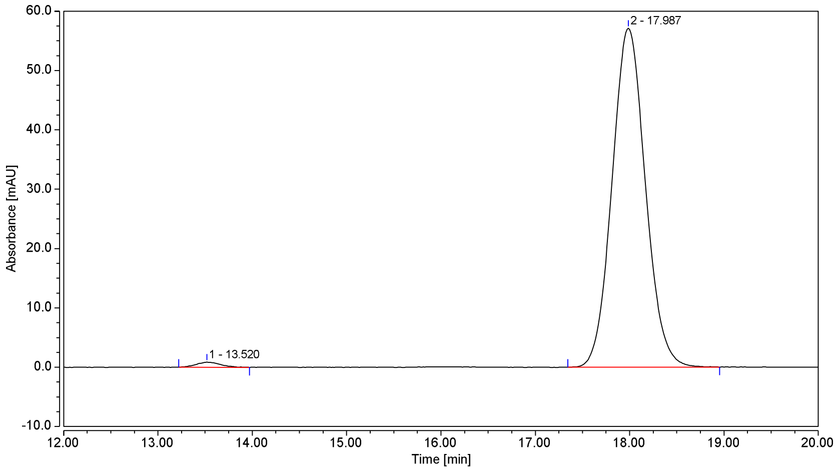

No.	Peak Name	Retention Time	Area	Height	Relative Area
		min	mAU*min	mAU	%
1		13.520	0.259	0.859	1.10
2		17.987	23.386	57.120	98.90
**Total:**			**23.645**	**57.979**	**100.00**

Chiral HPLC: (Chiralpak ODH, 5% ^{i}PrOH, 95% hexane, 1.00 mL min^{-1}, λ = 290 nm) τ_R (major) = 8.8 min, τ_R (minor) = 9.9 min

Racemic

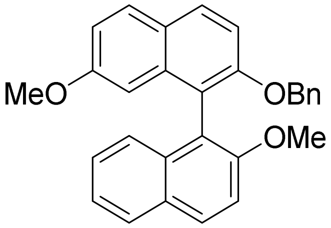

Bn-14

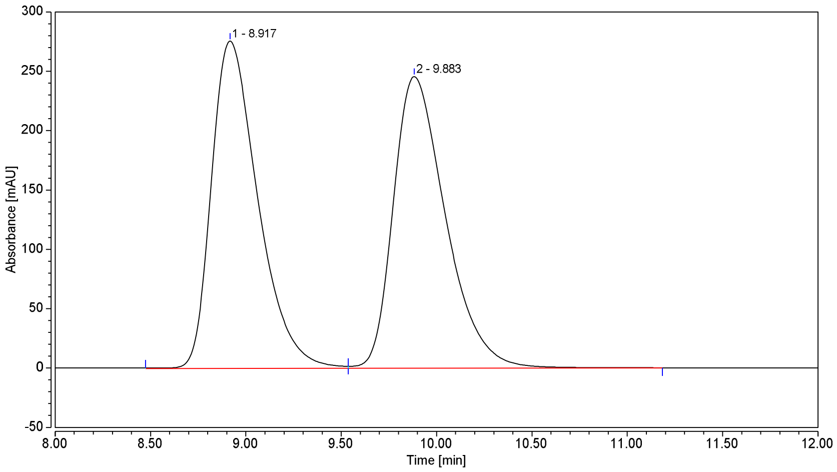

No.	Peak Name	Retention Time	Area	Height	Relative Area
		min	mAU*min	mAU	%
1		8.917	77.872	275.748	49.98
2		9.883	77.938	245.714	50.02
**Total:**			**155.810**	**521.461**	**100.00**

Asymmetric

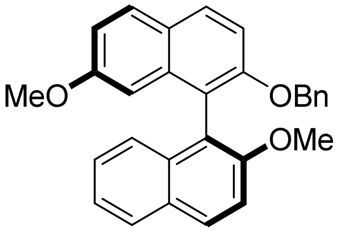

Bn-14

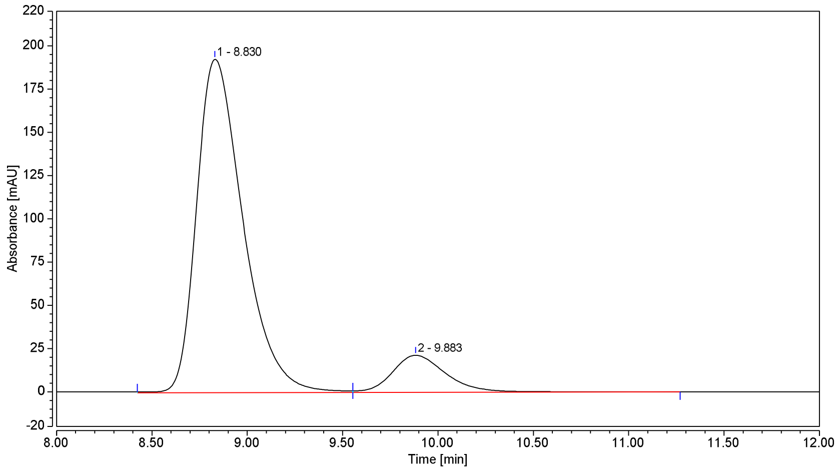

No.	Peak Name	Retention Time	Area	Height	Relative Area
		min	mAU*min	mAU	%
1		8.830	54.413	192.706	88.54
2		9.883	7.043	21.473	11.46
**Total:**			**61.456**	**214.179**	**100.00**

Chiral HPLC: (Chiralpak ADH, 10% ^{i}PrOH, 90% hexane, 1.0 mL min^{-1}, λ = 290 nm) τ_R (minor) = 12.8 min, τ_R (major) = 15.6 min.

Racemic

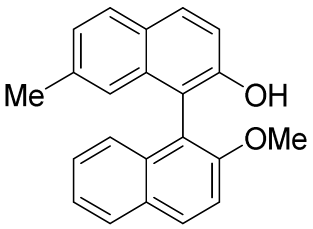

15

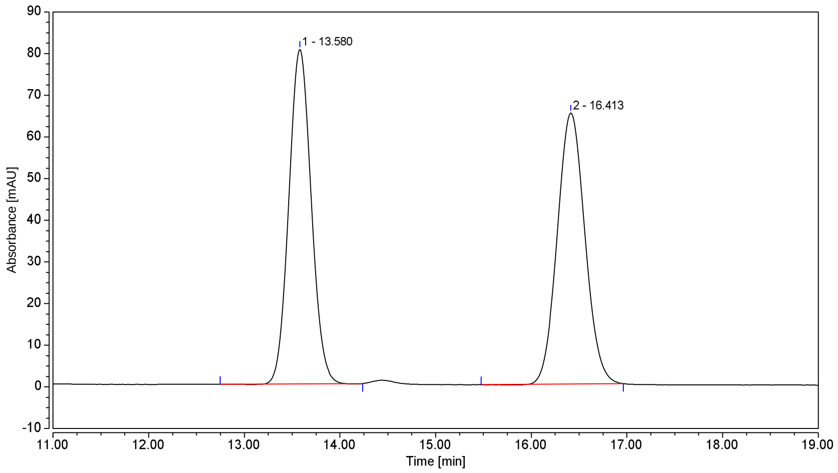

No.	Peak Name	Retention Time	Area	Height	Relative Area
		min	mAU*min	mAU	%
1		13.580	21.684	80.363	49.92
2		16.413	21.752	65.098	50.08
**Total:**			**43.436**	**145.461**	**100.00**

Asymmetric

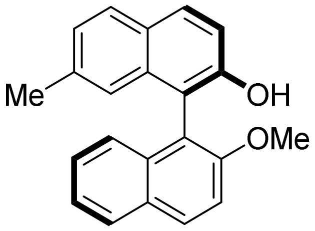

15

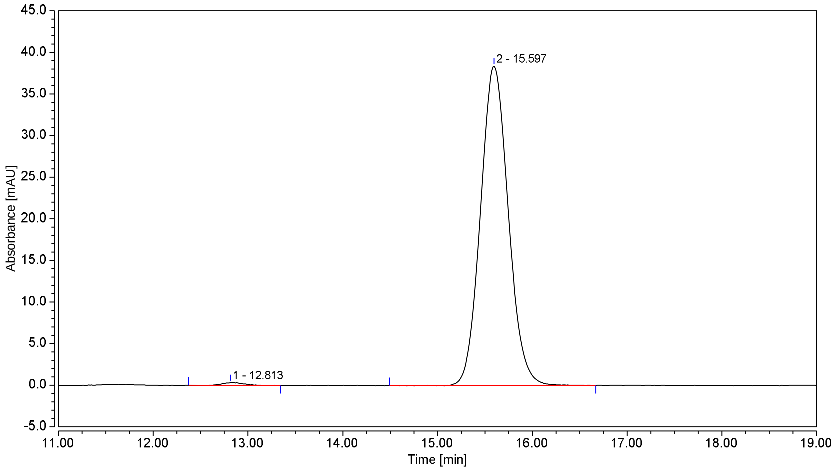

No.	Peak Name	Retention Time	Area	Height	Relative Area
		min	mAU*min	mAU	%
1		12.813	0.089	0.340	0.68
2		15.597	13.044	38.365	99.32
**Total:**			**13.133**	**38.705**	**100.00**

Chiral HPLC: (Chiralpak OJH, 3% EtOH, 97% hexane, 1.0 mL min^{-1}, λ = 300 nm) τ_R (minor) = 9.4 min, τ_R (major) = 15.1 min.

Racemic.

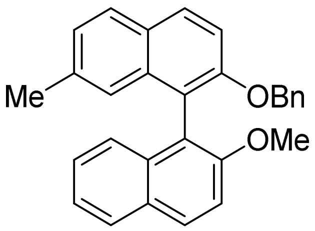

Bn-15

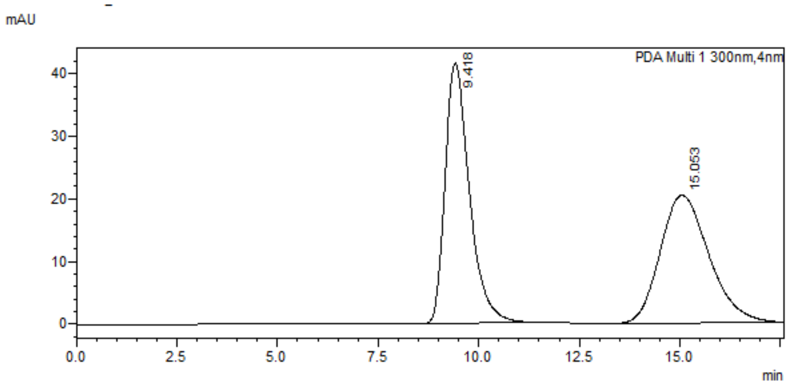

No.	Peak Name	Retention Time	Area	Height	Relative Area
		min	mAU*min	mAU	%
1		9.418	174.663	41.530	50.48
2		15.053	171.314	20.380	49.52
**Total:**			**345.977**	**61.910**	**100.00**

Asymmetric

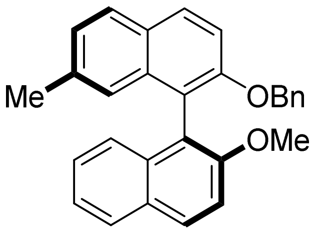

Bn-15

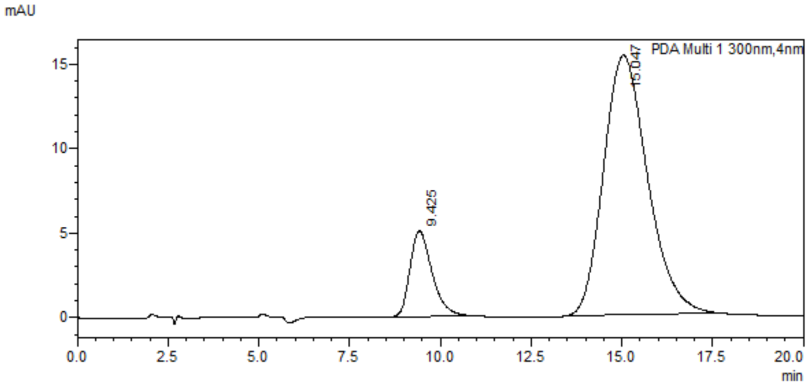

No.	Peak Name	Retention Time	Area	Height	Relative Area
		min	mAU*min	mAU	%
1		9.425	21.003	5.046	13.94
2		15.047	129.697	15.351	86.06
**Total:**			**150.700**	**20397**	**100.00**

Chiral HPLC: (Chiralpak ADH, 10% ^{i}PrOH, 90% hexane, 1.0 mL min^{-1}, λ = 272 nm) τ_R (major) = 11.6 min, τ_R (minor) = 14.1 min.

<u>Racemic</u>.

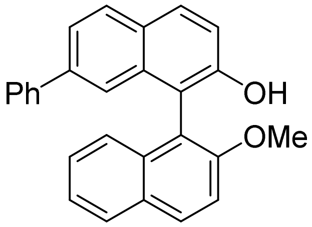

16

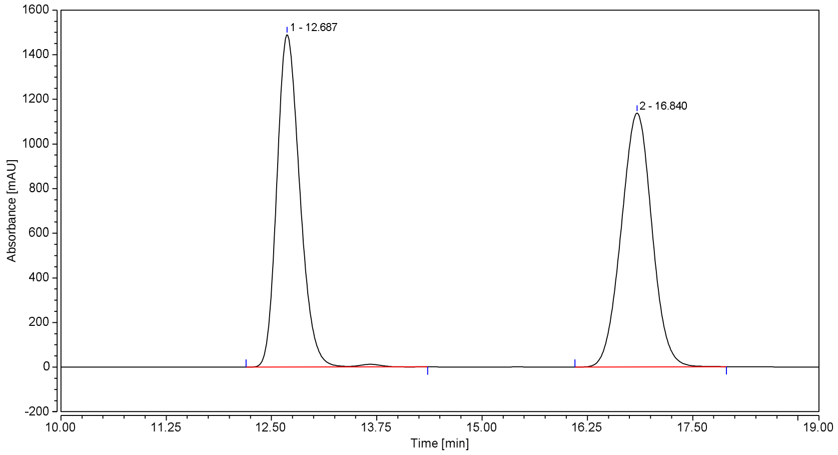

No.	Peak Name	Retention Time	Area	Height	Relative Area
		min	mAU*min	mAU	%
1		12.687	463.864	1485.694	49.45
2		16.840	474.176	1133.273	50.55
**Total:**			**938.040**	**2618.967**	**100.00**

<u>Asymmetric</u>

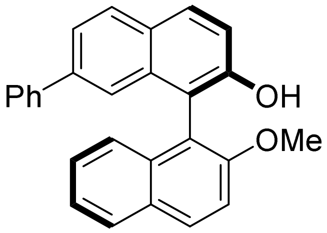

16

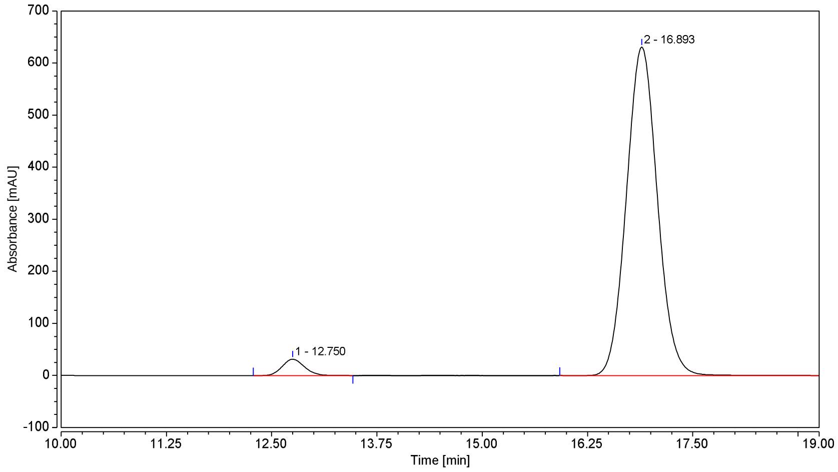

No.	Peak Name	Retention Time	Area	Height	Relative Area
		min	mAU*min	mAU	%
1		12.750	5.253	16.970	3.45
2		16.893	146.800	345.780	96.55
**Total:**			**152.053**	**362.750**	**100.00**

Chiral HPLC: (Chiralpak ADH, 2% ^{i}PrOH, 98% hexane, 1.0 mL min^{-1}, λ = 259 nm) τ_R (major) = 20.5 min, τ_R (minor) = 23.5 min.

Racemic.

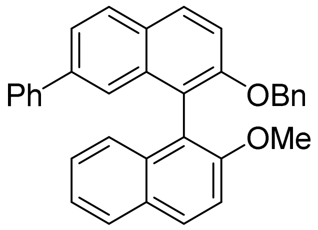

Bn-16

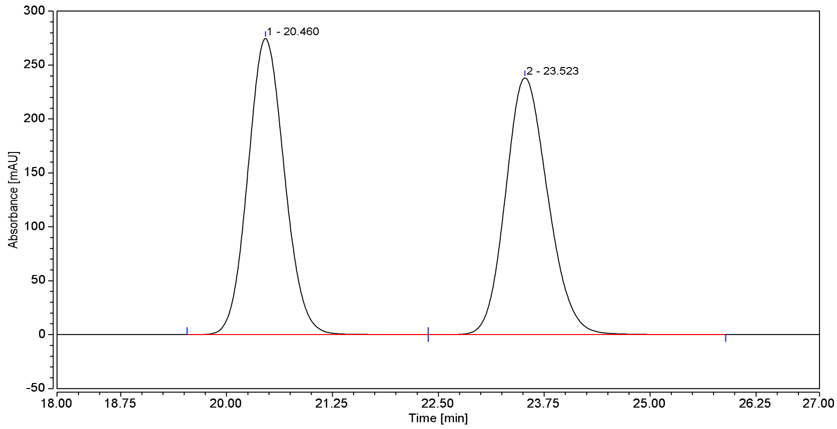

No.	Peak Name	Retention Time	Area	Height	Relative Area
		min	mAU*min	mAU	%
1		20.460	137.453	274.675	49.94
2		23.523	137.773	238.214	50.06
**Total:**			**275.226**	**512.889**	**100.00**

Asymmetric

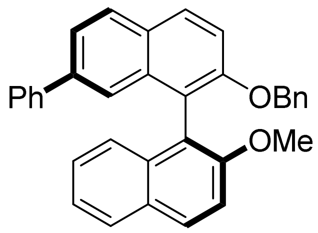

Bn-16

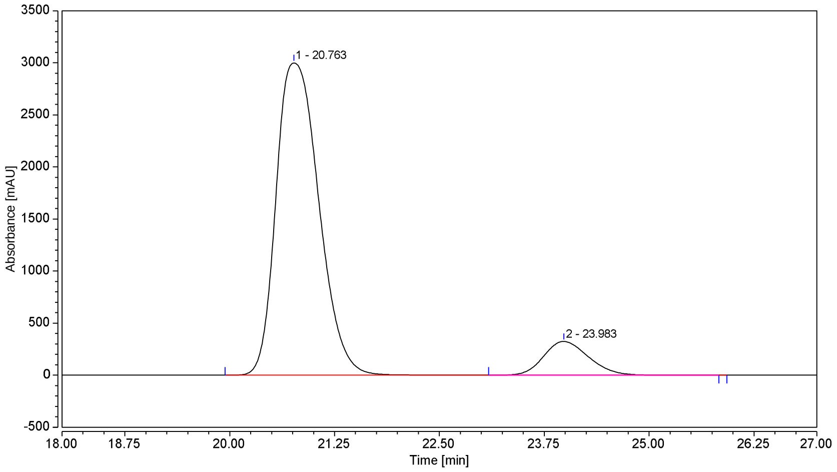

No.	Peak Name	Retention Time	Area	Height	Relative Area
		min	mAU*min	mAU	%
1		20.763	1770.358	2998.801	89.81
2		23.983	200.781	324.122	10.19
**Total:**			**1971.139**	**3322.924**	**100.00**

Chiral HPLC: (Chiralpak ADH, 10% ^{i}PrOH, 90% hexane, 1.0 mL min^{-1}) τ_R (major) = 11.6 min, τ_R (minor) = 14.1 min.

Racemic

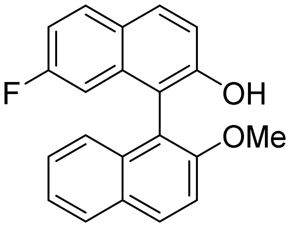

17

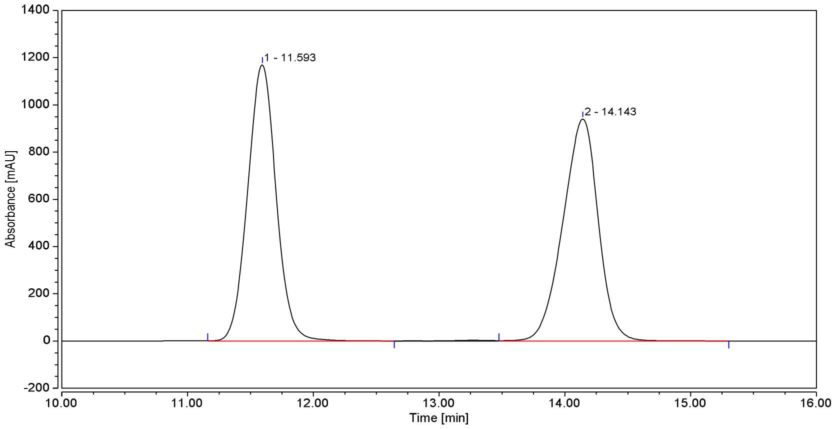

No.	Peak Name	Retention Time	Area	Height	Relative Area
		min	mAU*min	mAU	%
1		11.593	307.179	1170.227	50.05
2		14.143	306.510	940.471	49.95
**Total:**			**613.689**	**2110.699**	**100.00**

Asymmetric

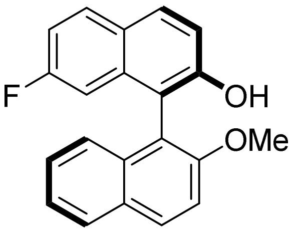

17

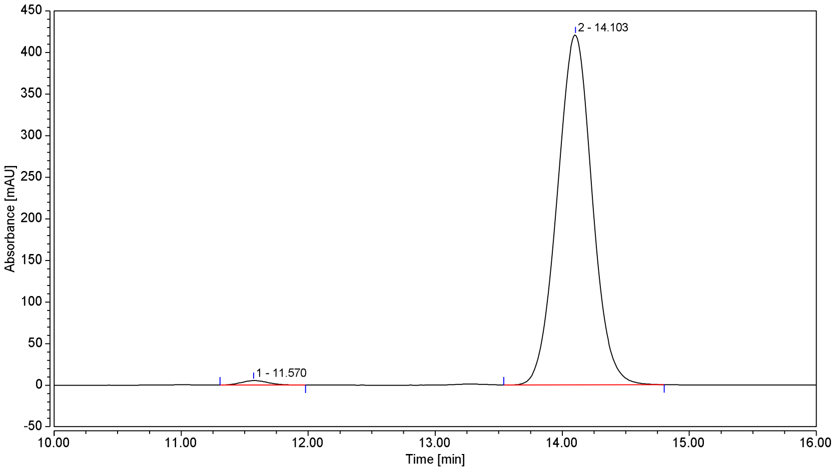

No.	Peak Name	Retention Time	Area	Height	Relative Area
		min	mAU*min	mAU	%
1		11.570	1.329	5.385	0.98
2		14.103	133.970	420.660	99.02
**Total:**			**135.299**	**426.045**	**100.00**

Chiral HPLC: (Chiralpak IC, 1% ^{i}PrOH, 99% hexane, 1.0 mL min^{-1}, λ = 294 nm) τ_R (major) = 7.0 min, τ_R (minor) = 9.3 min.

Racemic.

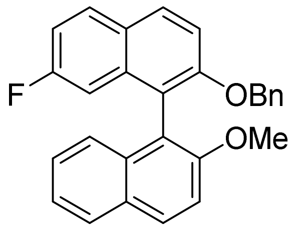

Bn-17

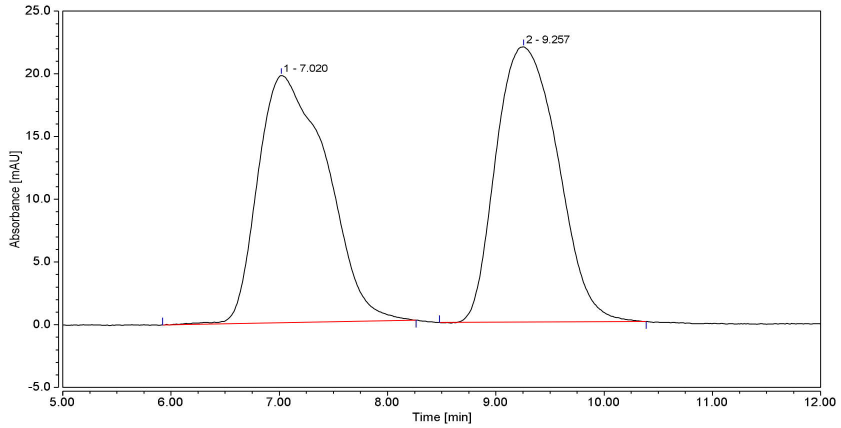

No.	Peak Name	Retention Time	Area	Height	Relative Area
		min	mAU*min	mAU	%
1		7.020	14.521	19.701	50.00
2		9.257	14.523	21.939	50.00
**Total:**			**29.044**	**41.64**	**100.00**

Asymmetric

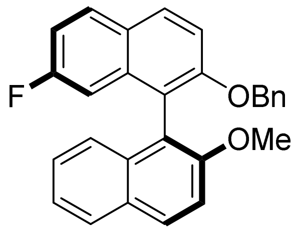

Bn-17

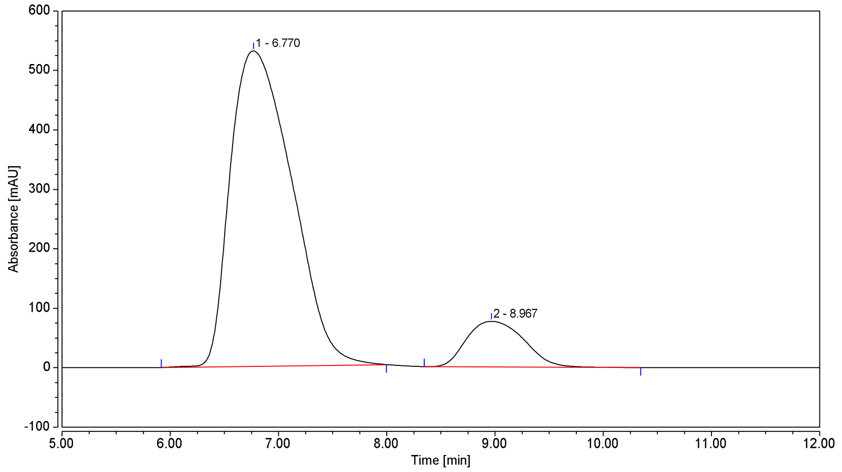

No.	Peak Name	Retention Time	Area	Height	Relative Area
		min	mAU*min	mAU	%
1		6.770	349.536	530.540	88.16
2		8.967	46.933	76.590	11.84
**Total:**			**396.469**	**607.130**	**100.00**

Chiral HPLC: (Chiralpak ADH, 15% ^{i}PrOH, 85% hexane, 1.00 mL min^{-1}, λ = 290 nm) τ_R (major) = 27.0 min, τ_R (minor) = 13.9 min

Racemic

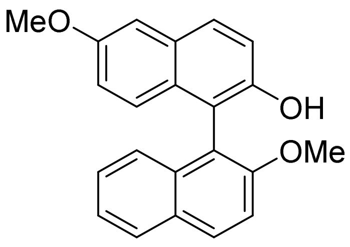

18

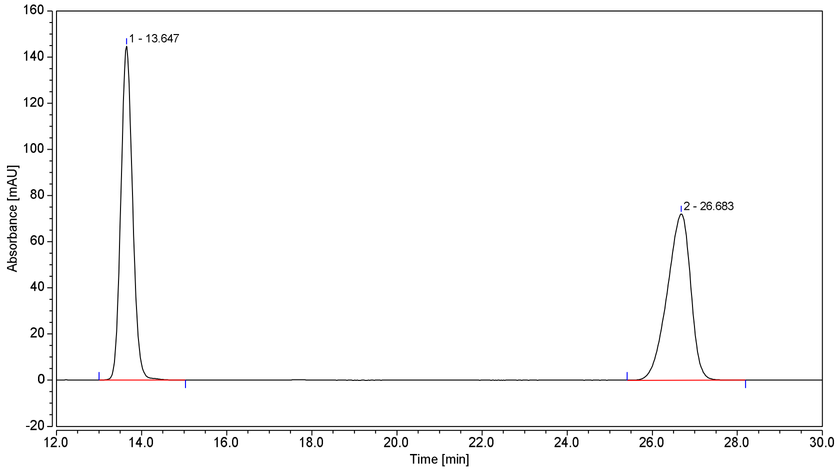

No.	Peak Name	Retention Time	Area	Height	Relative Area
		min	mAU*min	mAU	%
1		13.647	46.109	144.635	50.08
2		26.683	45.955	72.062	49.92
**Total:**			**92.064**	**216.697**	**100.00**

Asymmetric

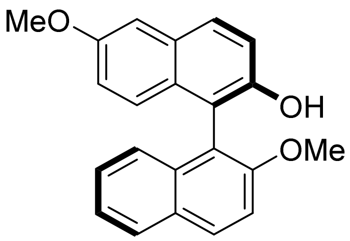

18

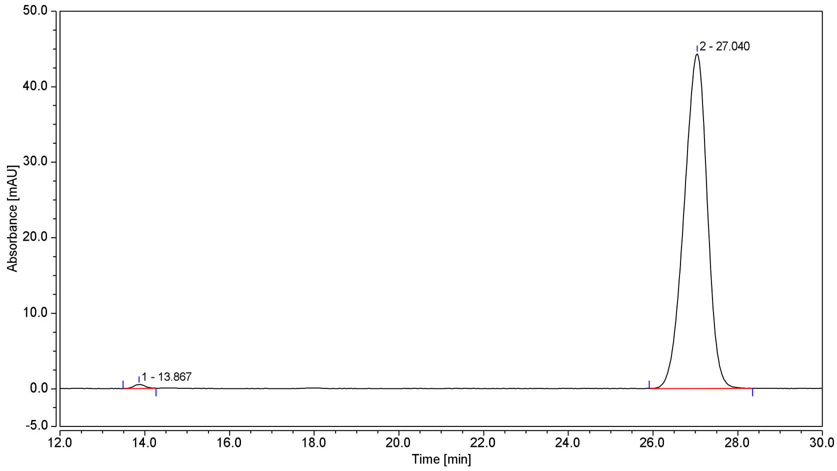

No.	Peak Name	Retention Time	Area	Height	Relative Area
		min	mAU*min	mAU	%
1		13.867	0.168	0.526	0.60
2		27.040	28.042	44.326	99.40
**Total:**			**28.210**	**44.852**	**100.00**

Chiral HPLC: (Chiralpak IC, 1% ^{i}PrOH, 99% hexane, 1.00 mL min^{-1}, λ = 290 nm) τ_R (major) = 15.3 min, τ_R (minor) = 30.6 min

Racemic

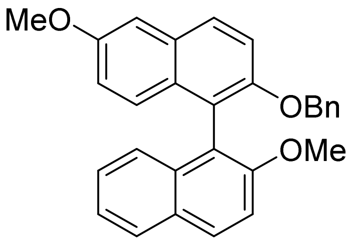

Bn-18

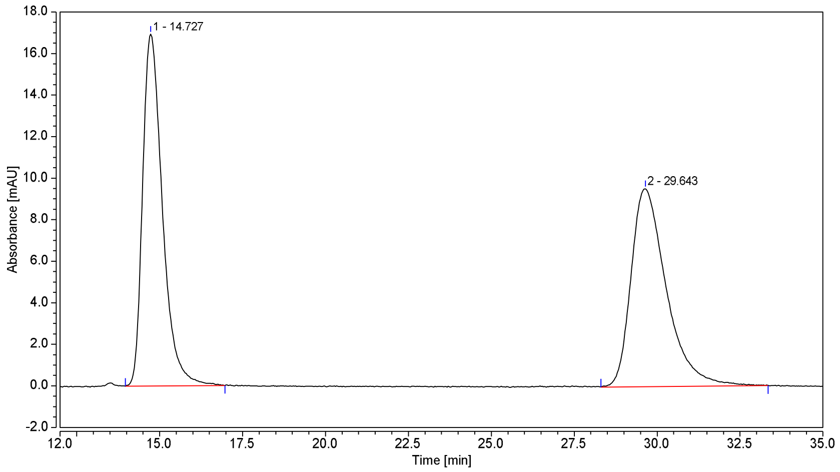

No.	Peak Name	Retention Time	Area	Height	Relative Area
		min	mAU*min	mAU	%
1		14.727	11.743	16.950	49.94
2		29.643	11.773	9.536	50.06
**Total:**			**23.516**	**26.485**	**100.00**

Asymmetric

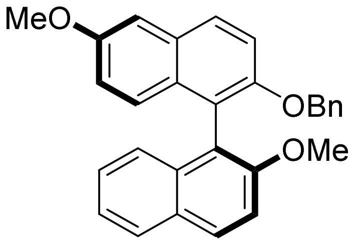

Bn-18

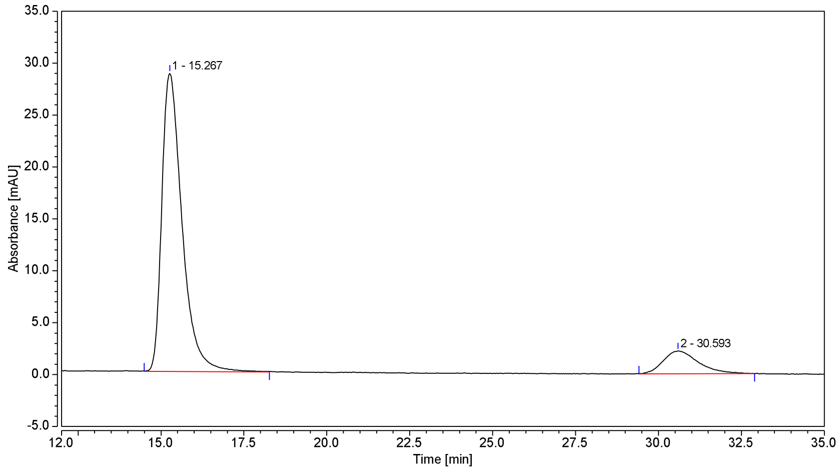

No.	Peak Name	Retention Time	Area	Height	Relative Area
		min	mAU*min	mAU	%
1		15.267	20.306	28.706	88.19
2		30.593	2.719	2.214	11.81
**Total:**			**23.025**	**30.920**	**100.00**

Chiral HPLC: (Chiralpak ADH, 15% ^{i}PrOH, 85% hexane, 1.0 mL min^{-1}, λ = 272 nm) τ_R (minor) = 20.4 min, τ_R (major) = 30.4 min.

Racemic

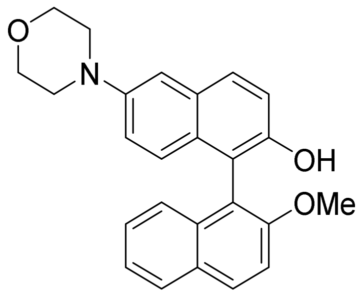

19

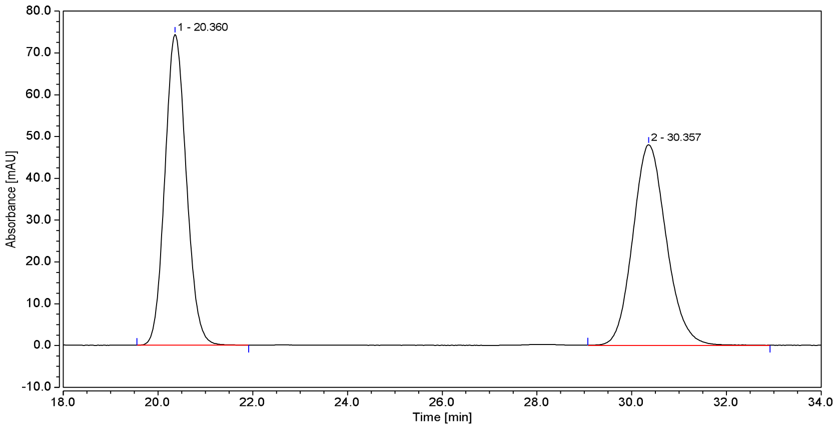

No.	Peak Name	Retention Time	Area	Height	Relative Area
		min	mAU*min	mAU	%
1		20.360	39.036	74.401	49.78
2		30.357	39.382	48.037	50.22
**Total:**			**78.419**	**122.439**	**100.00**

Asymmetric

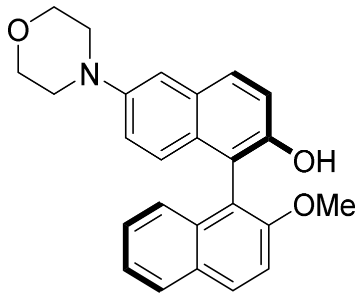

19

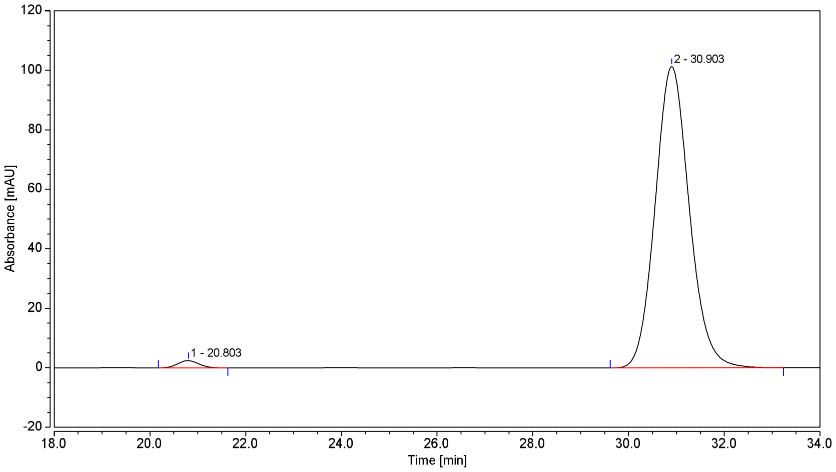

No.	Peak Name	Retention Time	Area	Height	Relative Area
		min	mAU*min	mAU	%
1		20.803	1.270	2.451	1.49
2		30.903	83.750	101.373	98.51
**Total:**			**85.020**	**103.825**	**100.00**

Chiral HPLC: (Chiralpak IC, 3% ^{i}PrOH, 97% hexane, 1.0 mL min^{-1}, λ = 289 nm) τ_R (major) = 23.3 min, τ_R (minor) = 30.4 min.

Racemic.

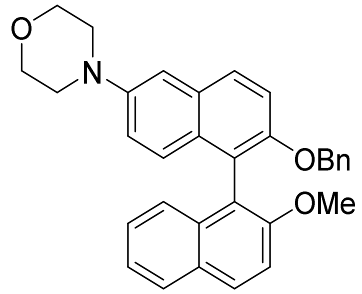

Bn-19

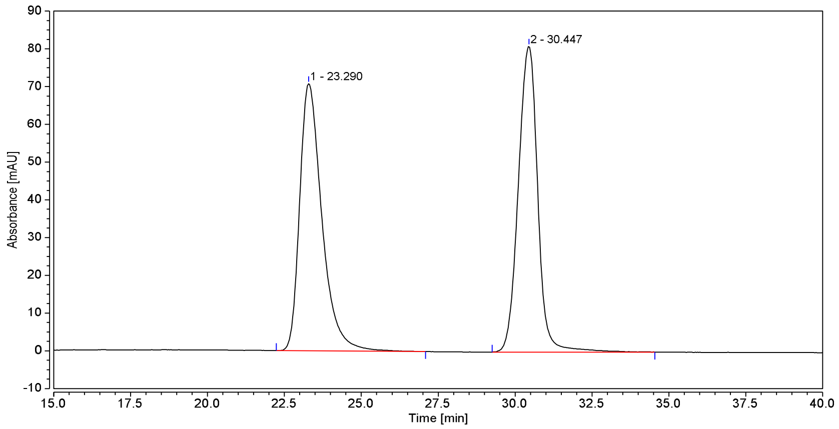

No.	Peak Name	Retention Time	Area	Height	Relative Area
		min	mAU*min	mAU	%
1		23.290	59.430	70.863	49.99
2		30.447	59.446	81.033	50.01
**Total:**			**118.875**	**151.896**	**100.00**

Asymmetric

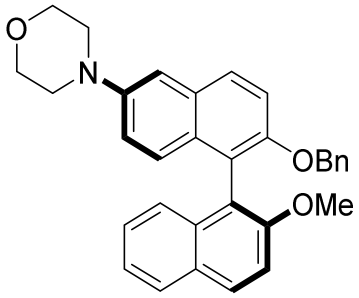

Bn-19

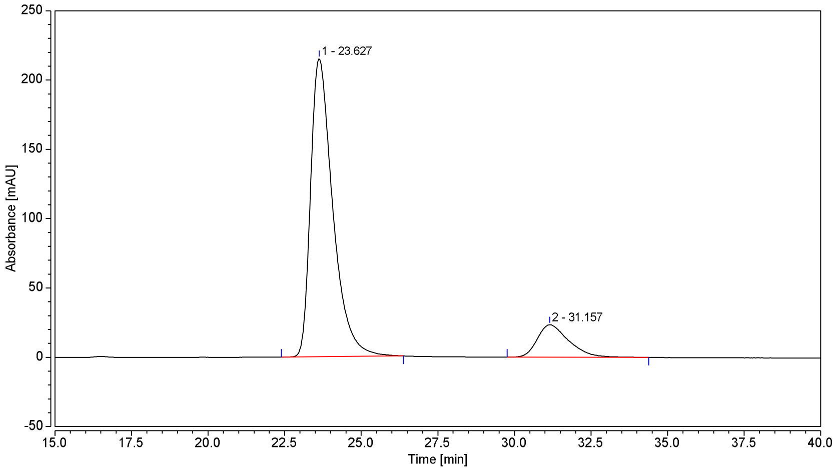

No.	Peak Name	Retention Time	Area	Height	Relative Area
		min	mAU*min	mAU	%
1		23.627	180.787	215.076	86.61
2		31.157	27.957	23.444	13.39
**Total:**			**208.744**	**238.520**	**100.00**

Chiral HPLC: (Chiralpak ADH, 10% ^{i}PrOH, 90% hexane, 1.0 mL min^{-1}, λ = 259 nm) τ_R (major) = 21.2 min, τ_R (minor) = 13.2 min.

Racemic

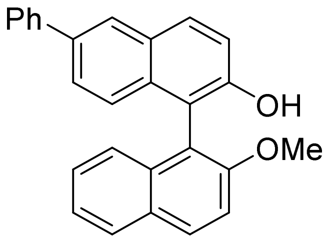

20

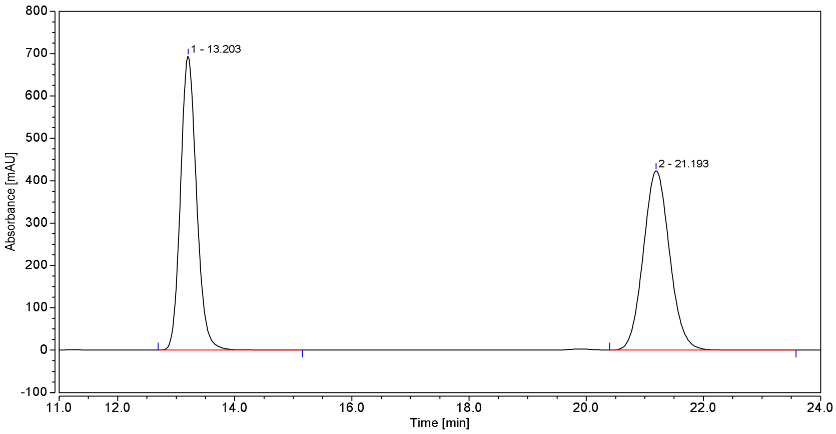

No.	Peak Name	Retention Time	Area	Height	Relative Area
		min	mAU*min	mAU	%
1		13.203	221.030	693.723	50.08
2		21.193	220.318	423.552	49.92
**Total:**			**441.348**	**1117.274**	**100.00**

Asymmetric

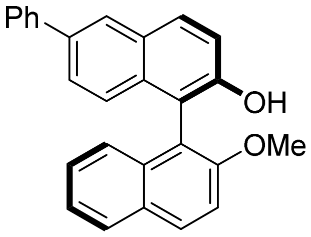

20

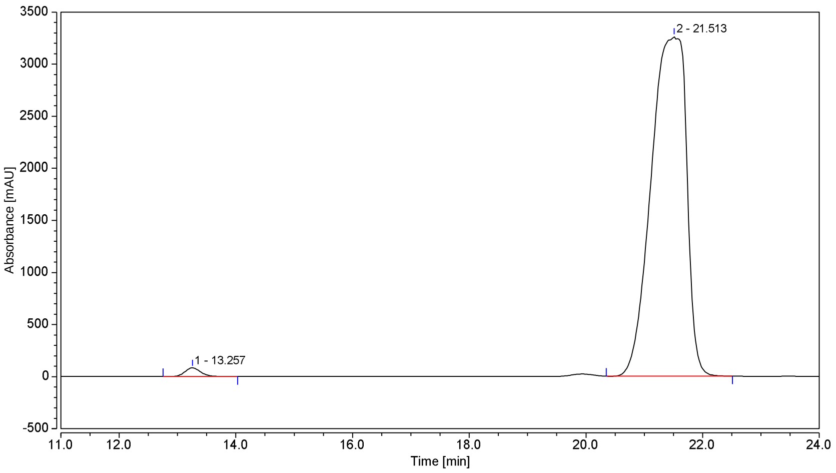

No.	Peak Name	Retention Time	Area	Height	Relative Area
		min	mAU*min	mAU	%
1		13.257	26.512	83.777	1.14
2		21.513	2290.501	3263.822	98.86
**Total:**			**2317.013**	**3347.599**	**100.00**

Chiral HPLC: (Chiralpak IC, 1% ^{i}PrOH, 99% hexane, 1.0 mL min^{-1}, λ = 259 nm) τ_R (major) = 10.2 min, τ_R (minor) = 19.7 min.

Racemic

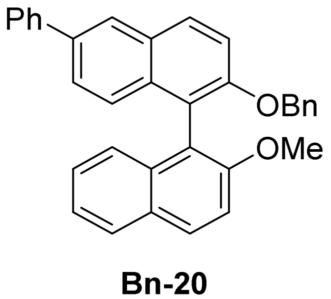

Bn-20

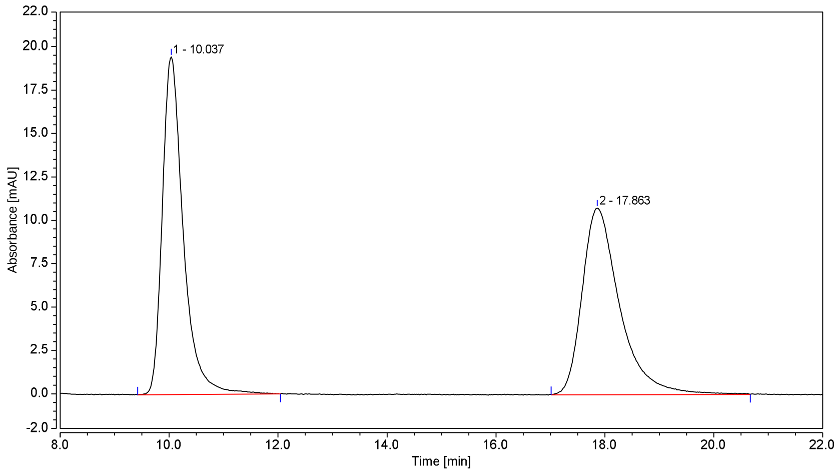

No.	Peak Name	Retention Time	Area	Height	Relative Area
		min	mAU*min	mAU	%
1		10.037	8.170	18.163	50.61
2		17.867	7.972	10.034	49.39
**Total:**			**16.142**	**28.197**	**100.00**

Asymmetric

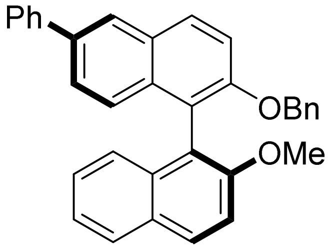

Bn-20

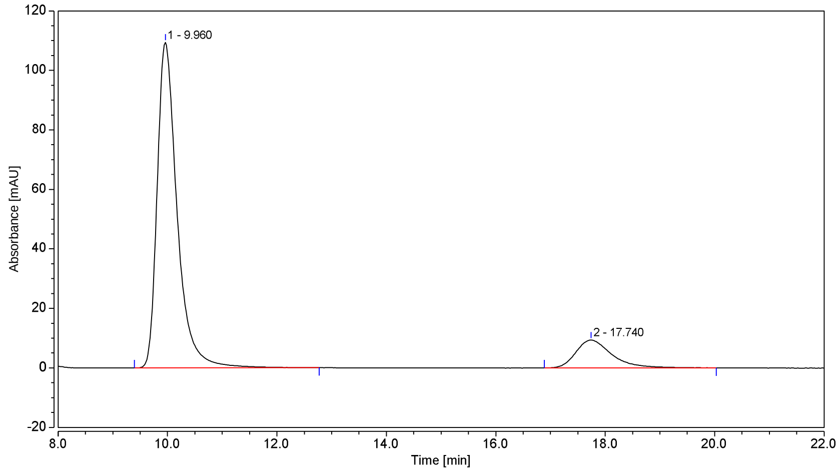

No.	Peak Name	Retention Time	Area	Height	Relative Area
		min	mAU*min	mAU	%
1		9.960	46.267	109.470	86.48
2		17.740	7.234	9.382	13.52
**Total:**			**53.501**	**118.852**	**100.00**

Chiral HPLC: (Chiralpak ADH, 1% ^{i}PrOH, 99% hexane, 1.0 mL min^{-1}, λ = 281 nm) τ_R (major) = 19.1 min, τ_R (minor) = 14.3 min.

Racemic

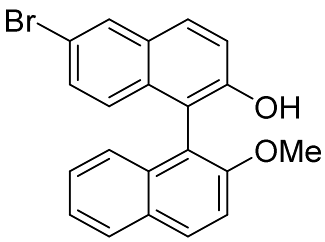

21

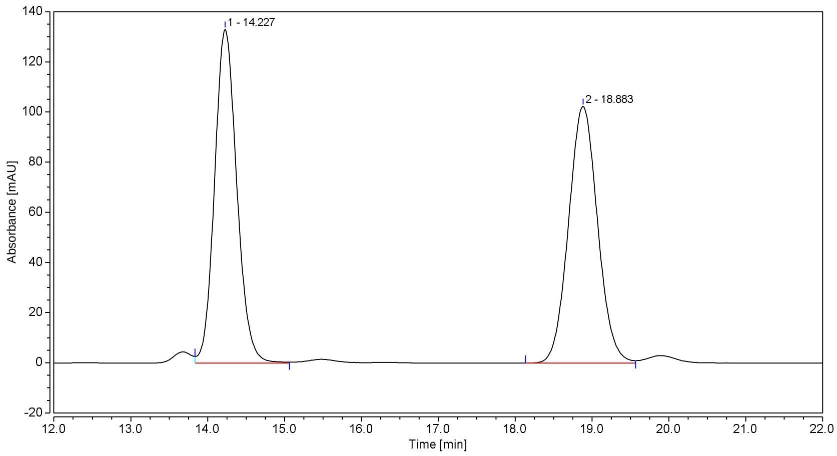

No.	Peak Name	Retention Time	Area	Height	Relative Area
		min	mAU*min	mAU	%
1		14.227	35.322	105.259	49.95
2		18.883	35.392	80.883	50.05
**Total:**			**70.714**	**186.142**	**100.00**

Asymmetric

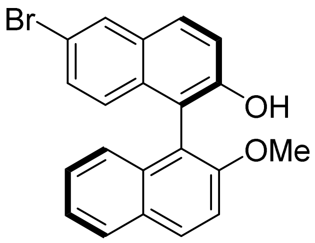

21

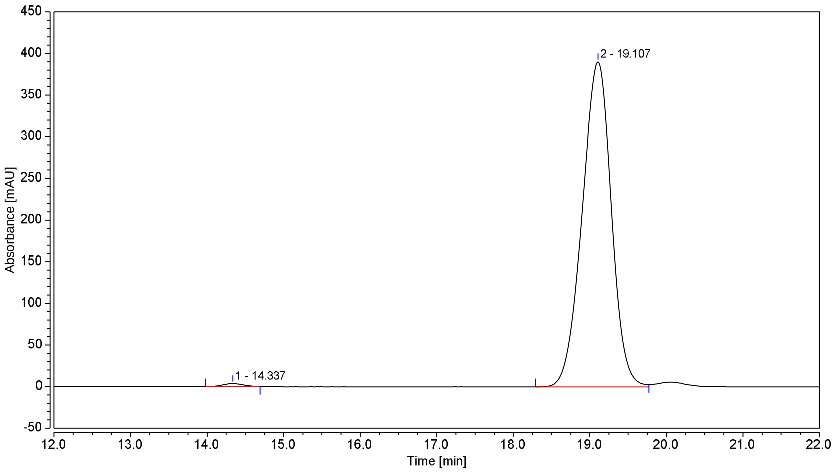

No.	Peak Name	Retention Time	Area	Height	Relative Area
		min	mAU*min	mAU	%
1		14.337	1.109	3.591	0.64
2		19.107	172.680	390.569	99.36
**Total:**			**173.788**	**394.161**	**100.00**

Chiral HPLC: (Chiralpak IC, 1% ^{i}PrOH, 99% hexane, 1.0 mL min^{-1}, λ = 340 nm) τ_R (major) = 6.3 min, τ_R (minor) = 9.8 min.

Racemic.

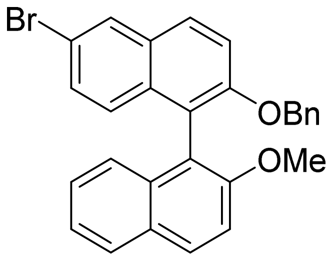

Bn-21

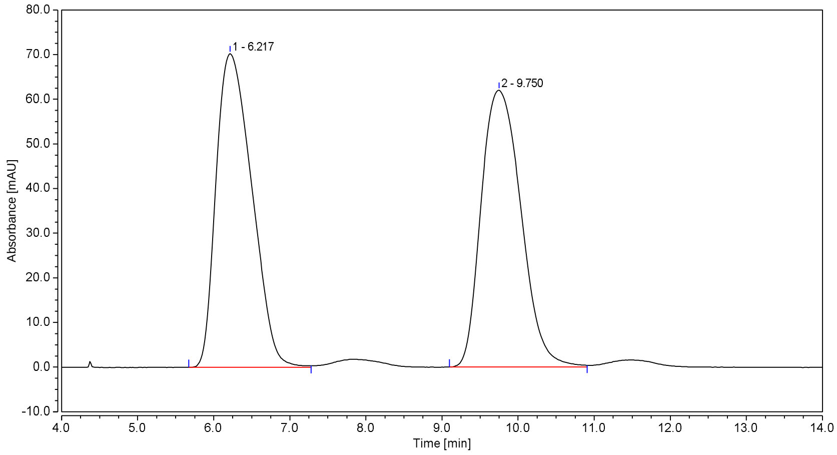

No.	Peak Name	Retention Time	Area	Height	Relative Area
		min	mAU*min	mAU	%
1		6.217	38.203	70.265	50.31
2		9.750	37.736	62.005	49.69
**Total:**			**75.939**	**132.270**	**100.00**

Asymmetric

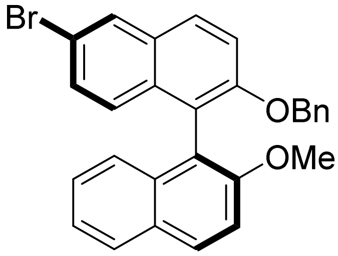

Bn-21

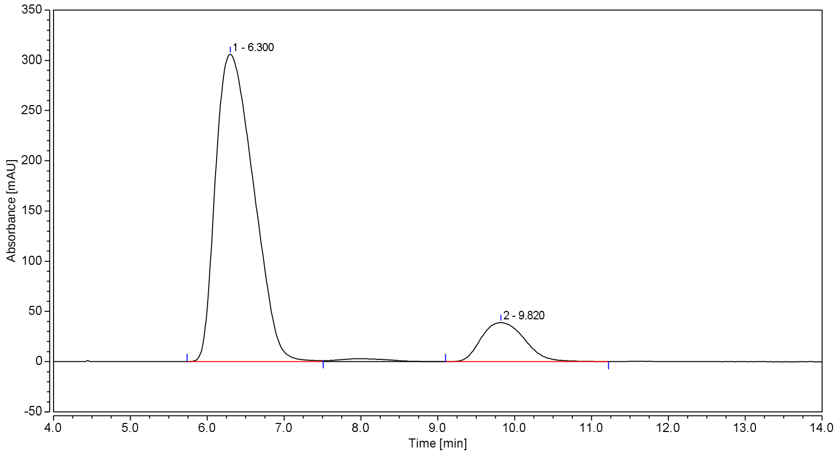

No.	Peak Name	Retention Time	Area	Height	Relative Area
		min	mAU*min	mAU	%
1		6.300	174.680	305.925	87.25
2		9.820	25.517	38.856	12.75
**Total:**			**200.197**	**344.781**	**100.00**

Chiral HPLC: (Chiralpak ADH, 10% ^{i}PrOH, 90% hexane, 1.0 mL min^{-1}, λ = 295 nm,) τ_R (major) = 20.0 min, τ_R (minor) = 12.8 min.

Racemic

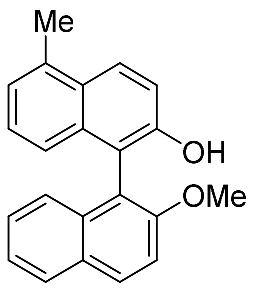

22

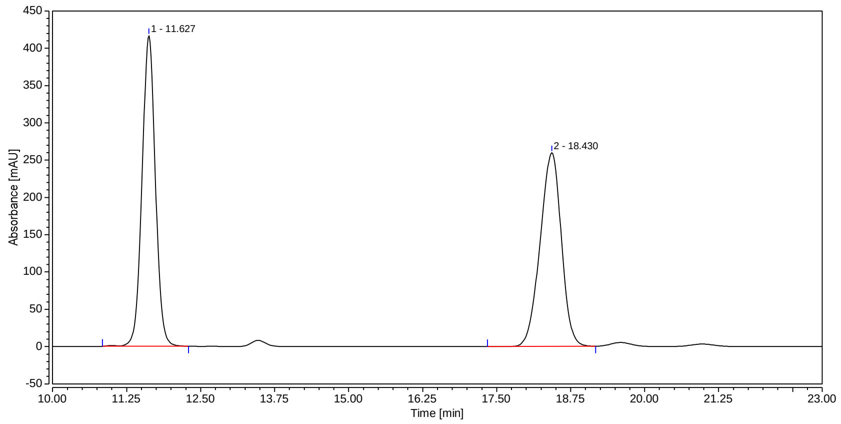

No.	Peak Name	Retention Time	Area	Height	Relative Area
		min	mAU*min	mAU	%
1		11.627	109.329	416.648	50.36
2		18.430	107.746	259.463	49.64
**Total:**			**217.075**	**676.111**	**100.00**

Asymmetric

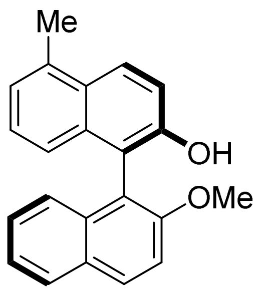

22

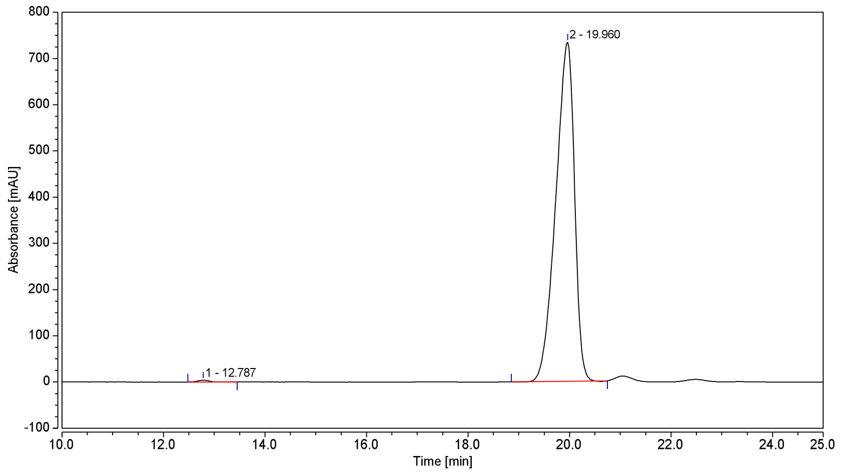

No.	Peak Name	Retention Time	Area	Height	Relative Area
		min	mAU*min	mAU	%
1		12.787	0.969	3.979	0.31
2		19.960	312.986	734.010	99.69
**Total:**			**313.954**	**737.988**	**100.00**

Chiral HPLC: (Chiralpak ADH, 4% ^{i}PrOH, 96% hexane, 1.0 mL min^{-1}, λ = 295 nm) τ_R (major) = 14.2 min, τ_R (minor) = 10.4 min.

Racemic.

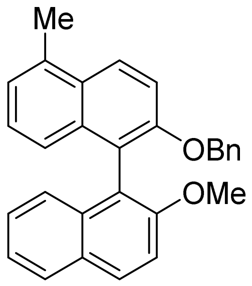

Bn-22

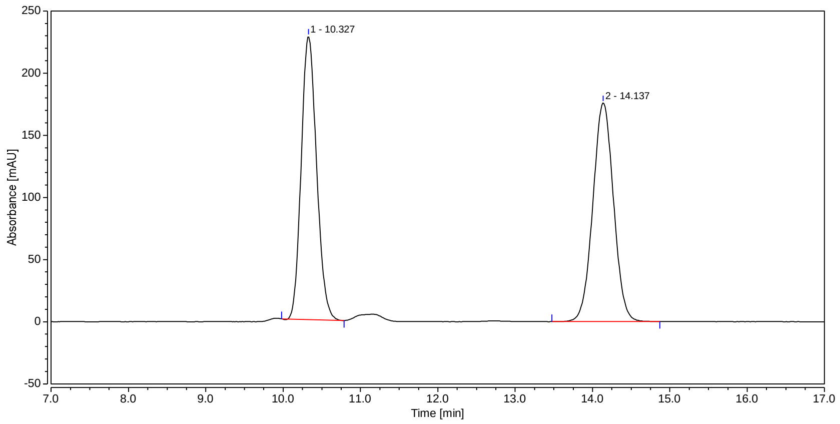

No.	Peak Name	Retention Time	Area	Height	Relative Area
		min	mAU*min	mAU	%
1		10.327	52.602	227.997	49.13
2		14.137	54.463	175.937	50.87
**Total:**			**107.064**	**403.934**	**100.00**

Asymmetric

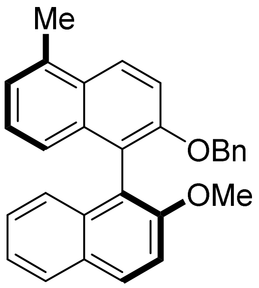

Bn-22

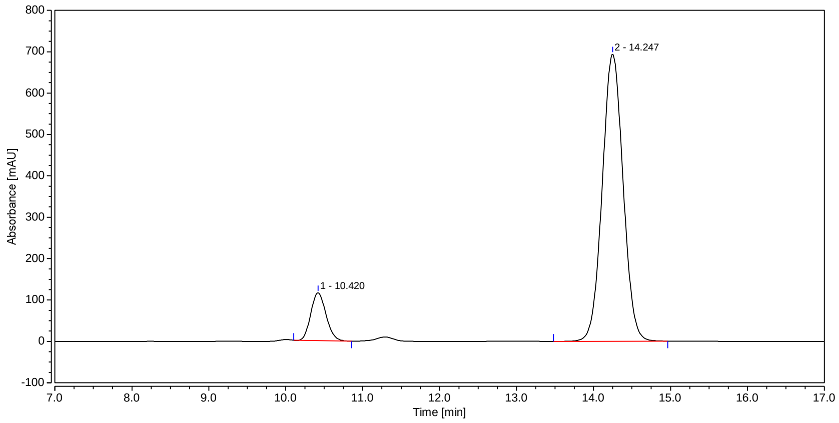

No.	Peak Name	Retention Time	Area	Height	Relative Area
		min	mAU*min	mAU	%
1		10.420	26.627	115.948	10.89
2		14.247	217.768	694.190	89.11
**Total:**			**244.394**	**810.138**	**100.00**

Chiral HPLC: (Chiralpak ADH, 10% ^{i}PrOH, 90% hexane, 1.0 mL min^{-1}, λ = 293 nm) τ_R (minor) = 17.2 min, τ_R (major) = 27.9 min.

Racemic

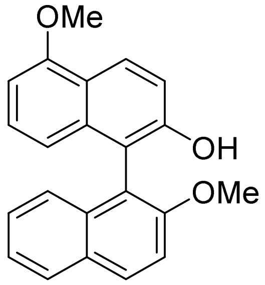

23

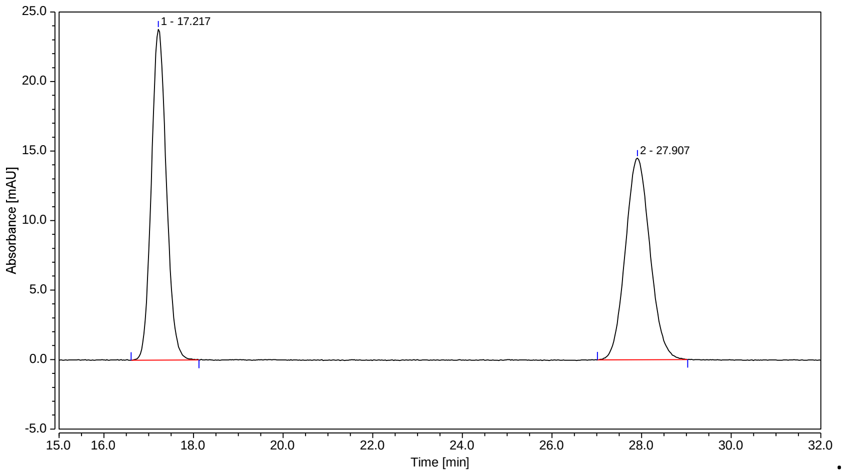

No.	Peak Name	Retention Time	Area	Height	Relative Area
		min	mAU*min	mAU	%
1		17.217	9.284	23.810	50.12
2		27.907	9.238	14.499	49.88
**Total:**			**18.522**	**38.309**	**100.00**

Asymmetric

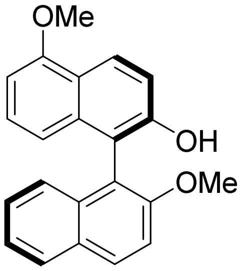

23

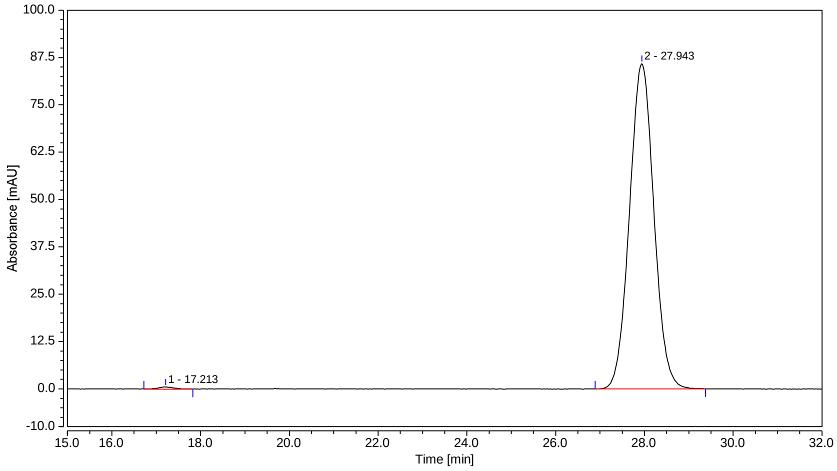

No.	Peak Name	Retention Time	Area	Height	Relative Area
		min	mAU*min	mAU	%
1		17.213	0.221	0.540	0.40
2		27.943	54.686	85.922	99.60
**Total:**			**54.907**	**86.462**	**100.00**

Chiral HPLC: (Chiralpak IC, 2% ^{i}PrOH, 98% hexane, 1.0 mL min^{-1}, λ = 295 nm) τ_R (major) = 10.7 min, τ_R (minor) = 17.3 min.

Racemic

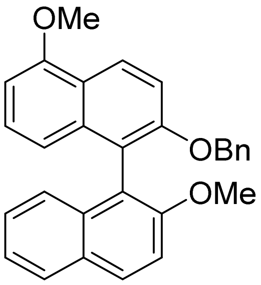

Bn-23

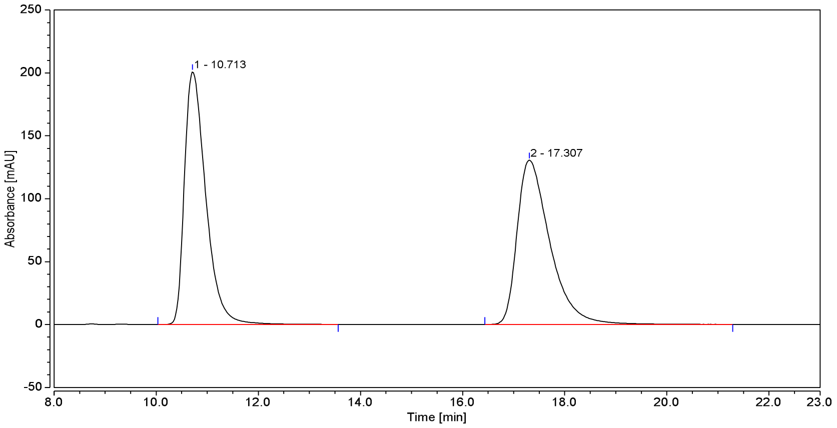

No.	Peak Name	Retention Time	Area	Height	Relative Area
		min	mAU*min	mAU	%
1		10.713	97.421	200.969	50.06
2		17.307	97.204	130.684	49.94
**Total:**			**194.624**	**331.653**	**100.00**

Asymmetric

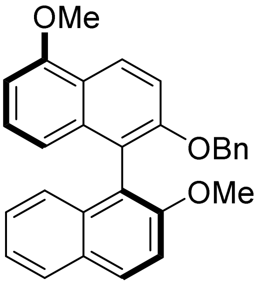

Bn-23

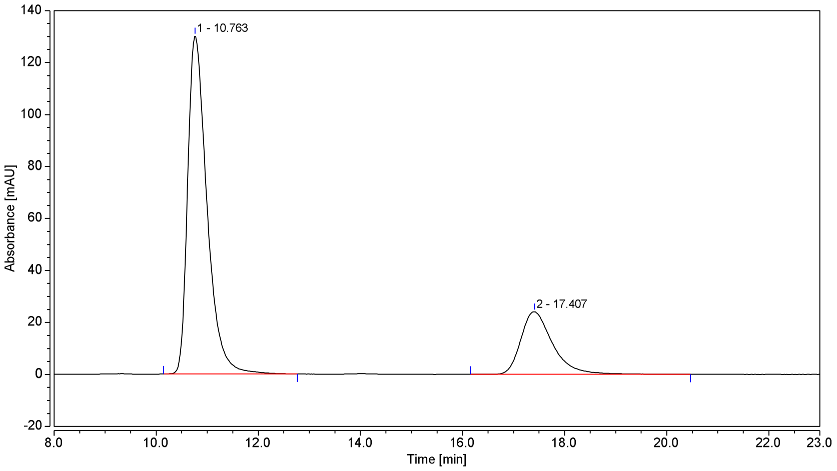

No.	Peak Name	Retention Time	Area	Height	Relative Area
		min	mAU*min	mAU	%
1		10.763	56.868	130.148	76.66
2		17.407	17.316	24.074	23.34
**Total:**			**74.184**	**154.221**	**100.00**

Chiral HPLC: (Chiralpak ADH, 10% ^{i}PrOH, 90% hexane, 1.0 mL min^{-1}, λ = 295 nm) τ_R (major) = 22.2 min, τ_R (minor) = 12.3 min.

Racemic

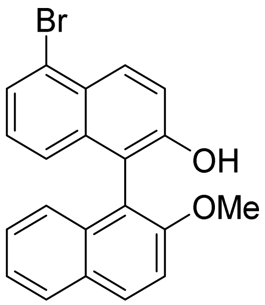

24

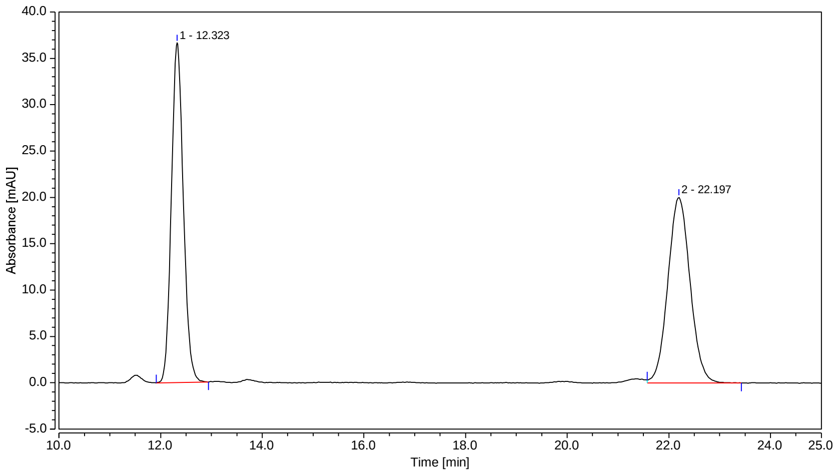

No.	Peak Name	Retention Time	Area	Height	Relative Area
		min	mAU*min	mAU	%
1		12.323	10.030	36.653	49.67
2		22.197	10.164	20.045	50.33
**Total:**			**20.194**	**56.698**	**100.00**

Asymmetric

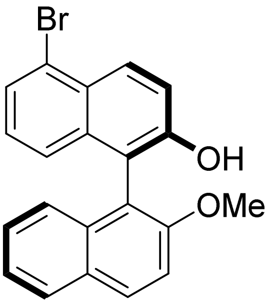

24

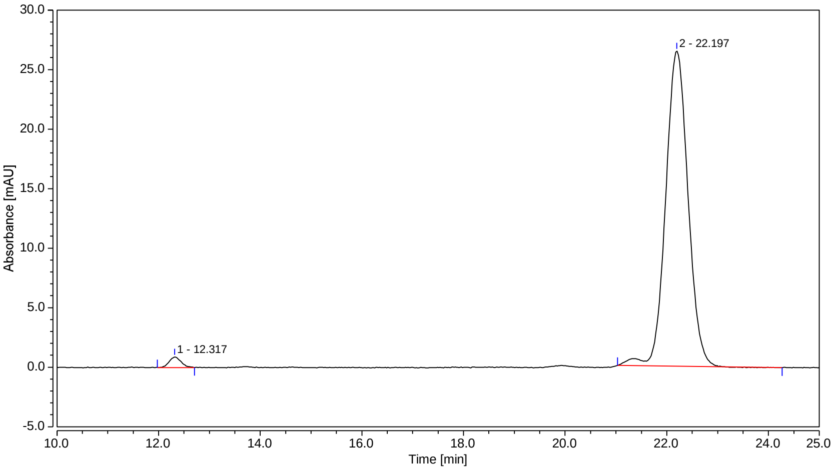

No.	Peak Name	Retention Time	Area	Height	Relative Area
		min	mAU*min	mAU	%
1		12.317	0.246	0.906	1.79
2		22.197	13.555	26.513	98.21
**Total:**			**13.801**	**27.419**	**100.00**

Chiral HPLC: (Chiralpak IC, 1% ^{i}PrOH, 99% hexane, 1.0 mL min^{-1}, λ = 295 nm) τ_R (major) = 5.6 min, τ_R (minor) = 8.5 min.

Racemic.

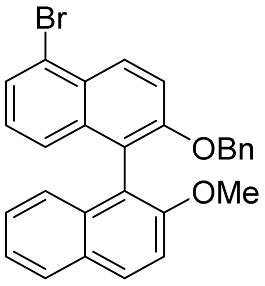

Bn-24

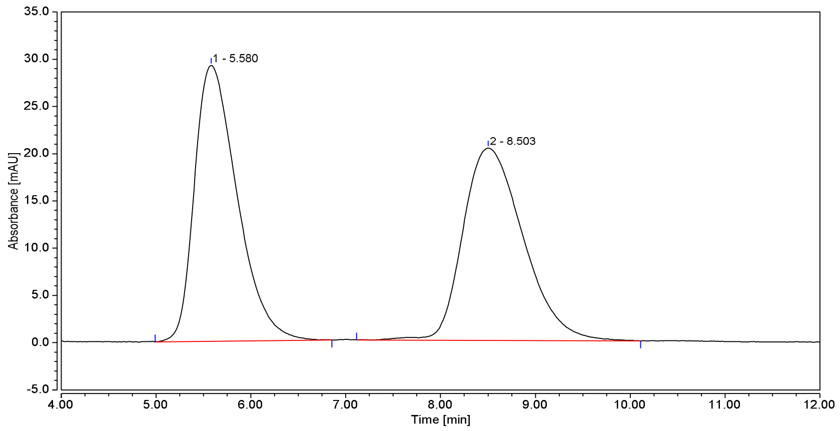

No.	Peak Name	Retention Time	Area	Height	Relative Area
		min	mAU*min	mAU	%
1		5.580	15.222	29.196	50.38
2		8.503	14.992	20.360	49.62
**Total:**			**30.214**	**49.556**	**100.00**

Asymmetric

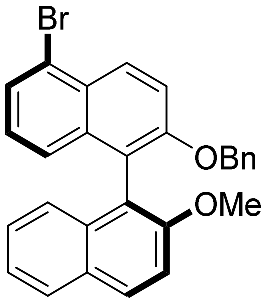

Bn-24

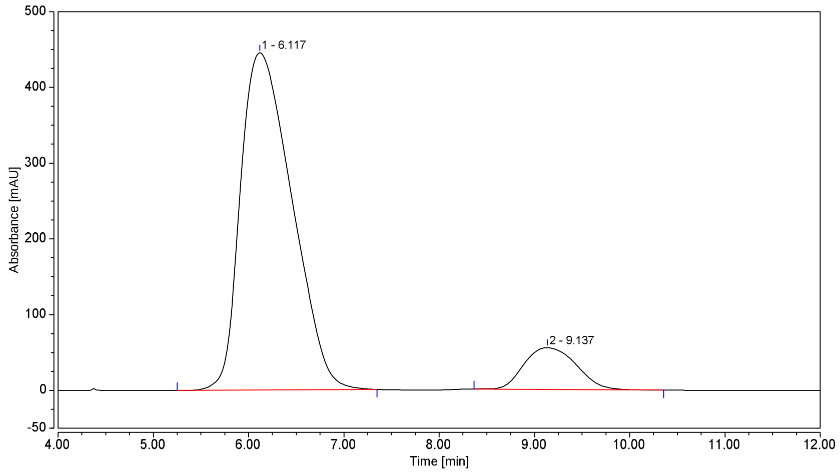

No.	Peak Name	Retention Time	Area	Height	Relative Area
		min	mAU*min	mAU	%
1		6.117	278.615	444.988	88.87
2		9.137	34.893	55.055	11.13
**Total:**			**313.508**	**500.043**	**100.00**

Chiral HPLC: (Chiralpak ADH, 15% ^{i}PrOH, 85% hexane, 1.0 mL min^{-1}, λ = 277 nm) τ_R (major) = 16.6 min, τ_R (minor) = 26.3 min.

Racemic

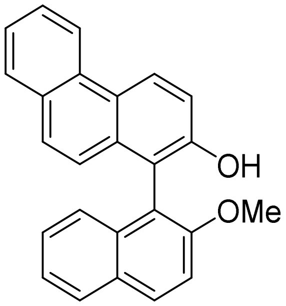

25

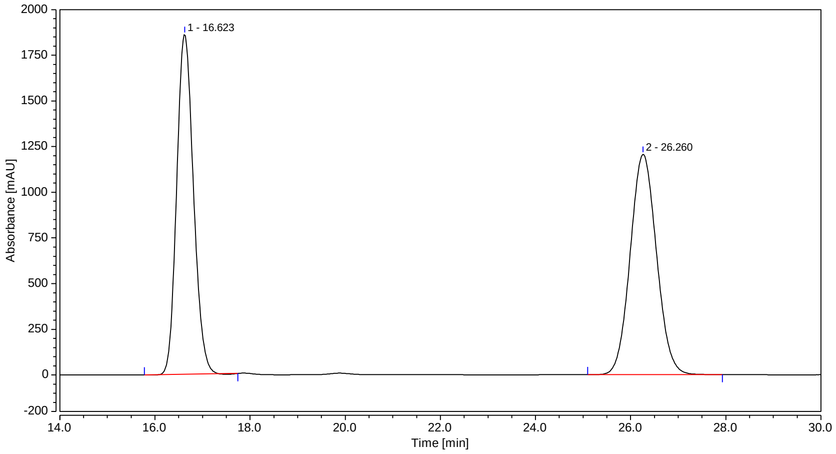

No.	Peak Name	Retention Time	Area	Height	Relative Area
		min	mAU*min	mAU	%
1		16.623	757.925	1861.162	49.58
2		26.260	770.901	1205.848	50.42
**Total:**			**1528.826**	**3067.010**	**100.00**

Asymmetric

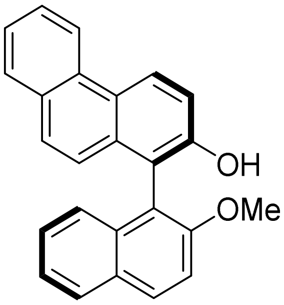

25

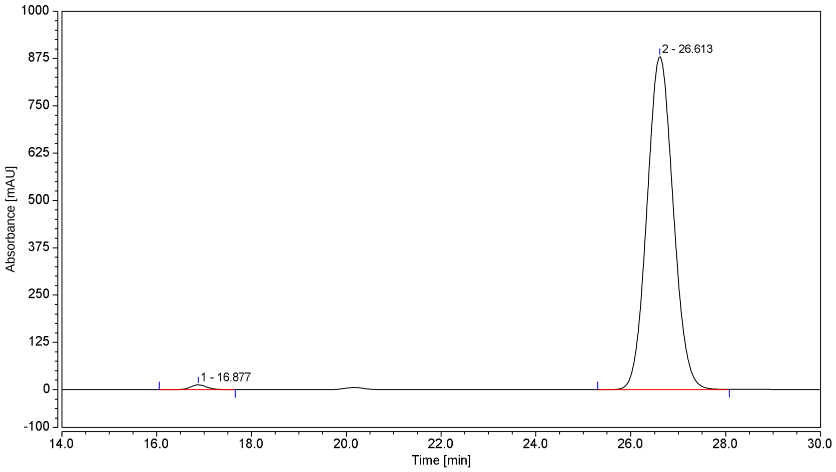

No.	Peak Name	Retention Time	Area	Height	Relative Area
		min	mAU*min	mAU	%
1		16.877	5.163	12.640	0.91
2		26.613	565.014	880.060	99.09
**Total:**			**570.177**	**892.700**	**100.00**

Chiral HPLC: (Chiralpak IC, 1% ^{i}PrOH, 99% hexane, 1.0 mL min^{-1}, λ = 222 nm) τ_R (major) = 20.8 min, τ_R (minor) = 33.0 min.

<u>Racemic</u>.

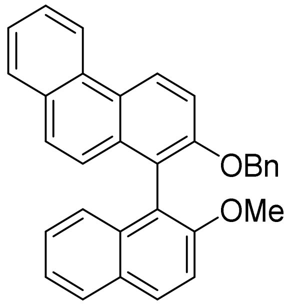

Bn-25

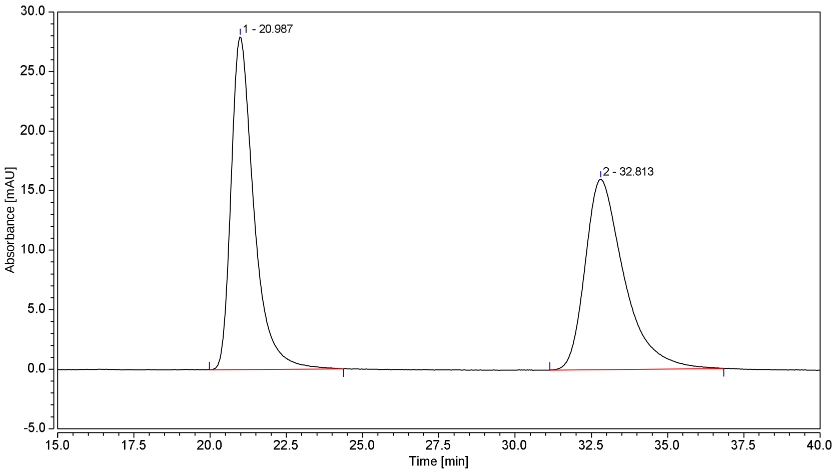

No.	Peak Name	Retention Time	Area	Height	Relative Area
		min	mAU*min	mAU	%
1		20.987	24.316	27.954	50.51
2		32.813	23.825	15.997	49.49
**Total:**			**48.142**	**43.950**	**100.00**

<u>Asymmetric</u>

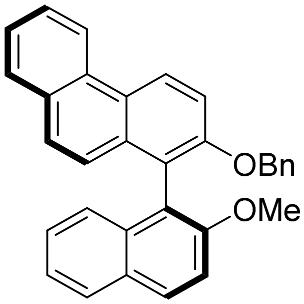

Bn-25

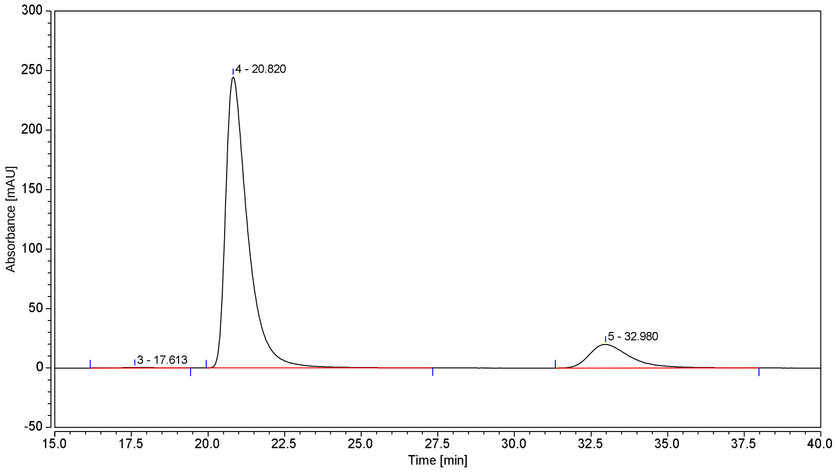

No.	Peak Name	Retention Time	Area	Height	Relative Area
		min	mAU*min	mAU	%
1		20.820	204.797	244.870	86.96
2		32.980	30.698	19.873	13.04
**Total:**			**235.495**	**264.743**	**100.00**

Chiral HPLC: (Chiralpak ADH, 10% ^{i}PrOH, 90% hexane, 1.0 mL min^{-1}, λ = 291 nm) τ_R (major) = 15.6 min, τ_R (minor) = 20.7 min.

Racemic

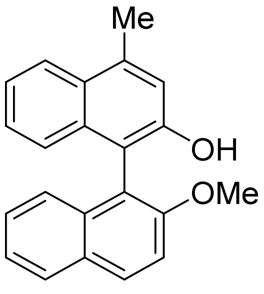

26

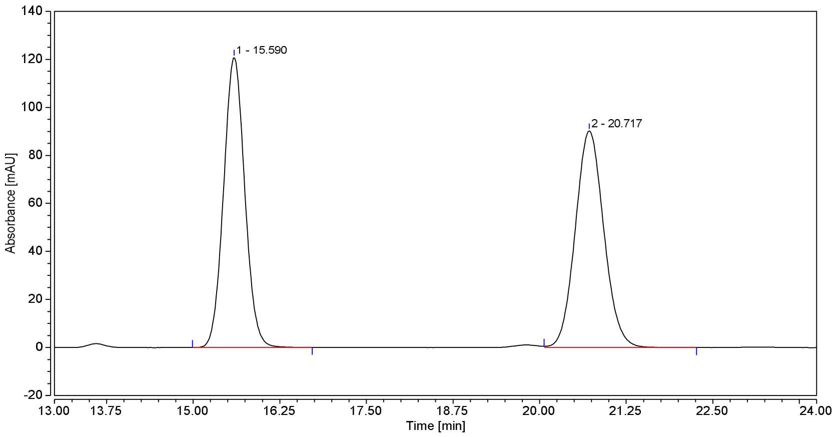

No.	Peak Name	Retention Time	Area	Height	Relative Area
		min	mAU*min	mAU	%
1		15.590	42.921	120.812	49.88
2		20.717	43.132	90.228	50.12
**Total:**			**86.053**	**211.039**	**100.00**

Asymmetric

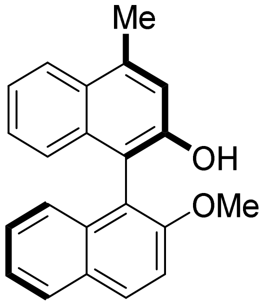

26

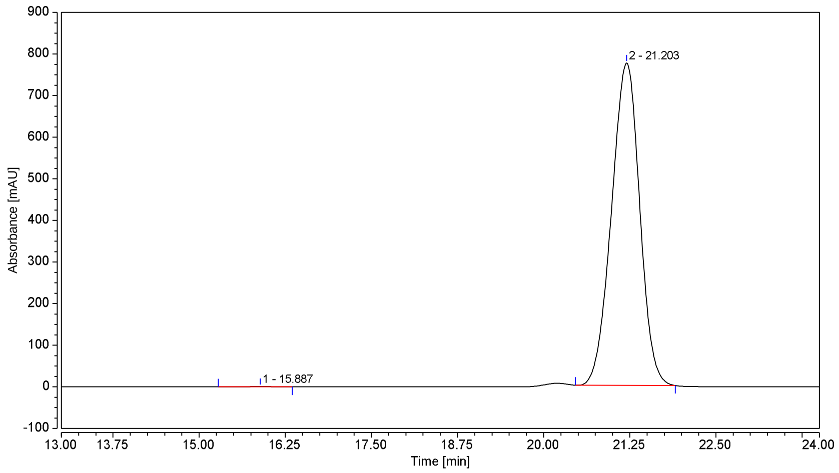

No.	Peak Name	Retention Time	Area	Height	Relative Area
		min	mAU*min	mAU	%
1		15.887	0.408	1.133	0.11
2		21.203	366.901	774.809	99.89
**Total:**			**367.309**	**775.942**	**100.00**

Chiral HPLC: (Chiralpak IC, 2% ^{i}PrOH, 98% hexane, 1.0 mL min^{-1}, λ = 291 nm) τ_R (major) = 15.6 min, τ_R (minor) = 24.7.

Racemic

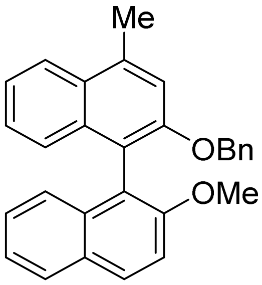

Bn-26

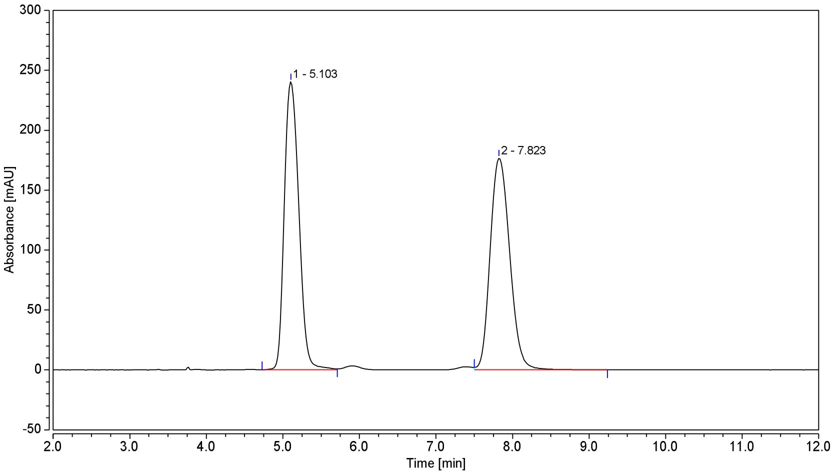

No.	Peak Name	Retention Time	Area	Height	Relative Area
		min	mAU*min	mAU	%
1		5.103	52.787	240.479	50.31
2		7.823	52.132	176.684	49.69
**Total:**			**104.919**	**417.164**	**100.00**

Asymmetric

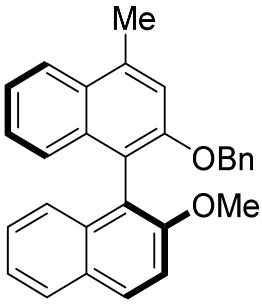

Bn-26

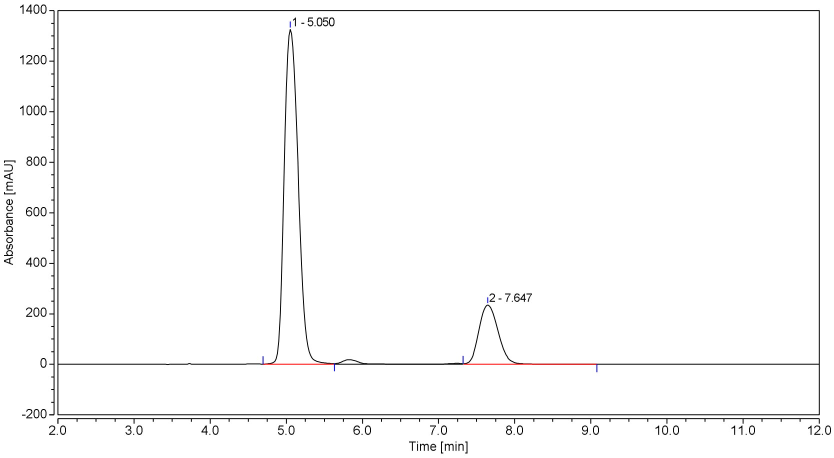

No.	Peak Name	Retention Time	Area	Height	Relative Area
		min	mAU*min	mAU	%
1		5.050	283.626	1325.859	80.55
2		7.647	68.469	234.308	19.45
**Total:**			**352.095**	**1560.167**	**100.00**

Chiral HPLC: (Chiralpak ADH, 10 % ^{i}PrOH, 90 % hexane, 1.0 mL min^{-1}, λ = 295 nm) τ_R (major) = 26.2 min, τ_R (minor) = 18.8 min.

Racemic

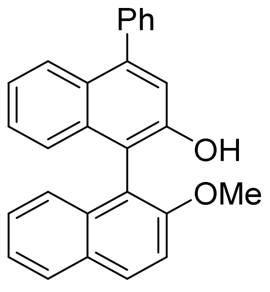

27

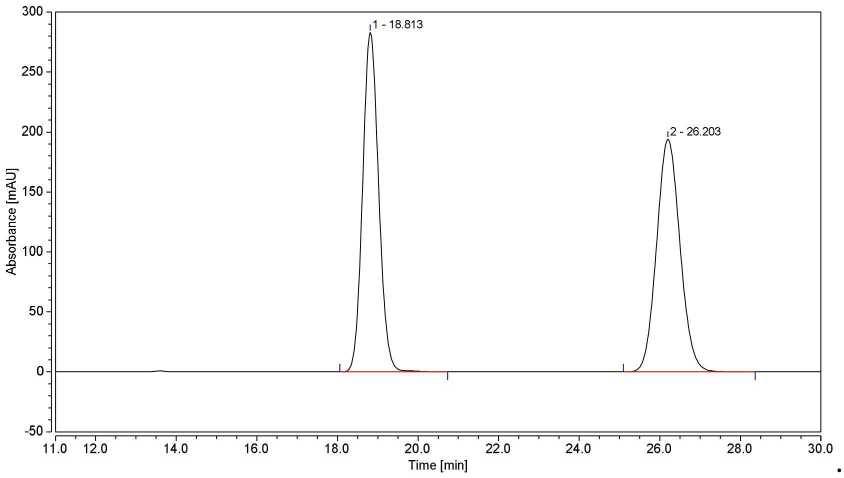

No.	Peak Name	Retention Time	Area	Height	Relative Area
		min	mAU*min	mAU	%
1		18.813	130.803	282.953	50.03
2		26.203	130.657	193.909	49.97
**Total:**			261.460	476.862	100.00

Asymmetric

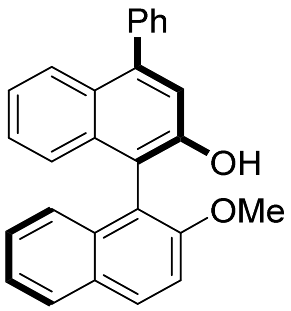

27

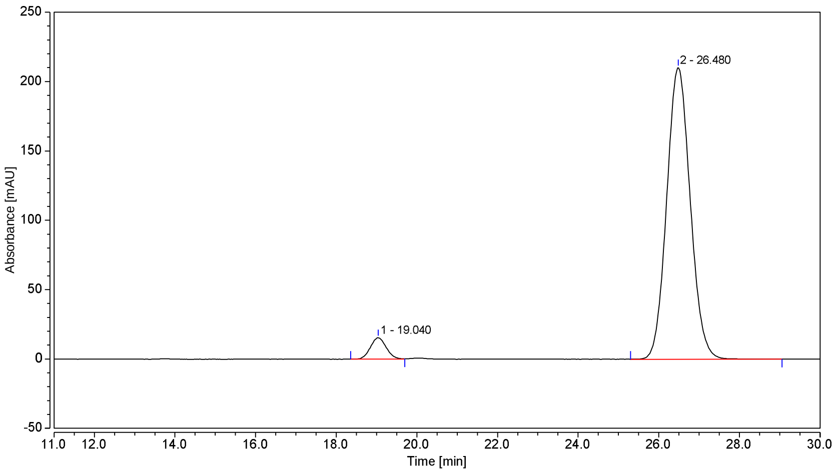

No.	Peak Name	Retention Time	Area	Height	Relative Area
		min	mAU*min	mAU	%
1		19.040	7.102	15.479	4.76
2		26.480	142.192	210.317	95.24
**Total:**			**149.294**	**225.795**	**100.00**

Chiral HPLC: (Chiralpak ADH, 2% ^{i}PrOH, 98% hexane, 1.0 mL min^{-1}, λ = 297 nm) τ_R (major) = 24.5 min, τ_R (minor) = 14.8 min.

<u>Racemic</u>

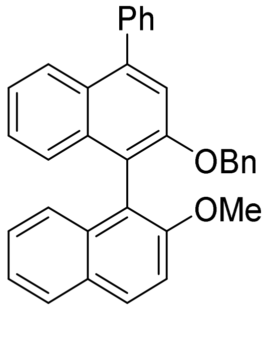

Bn-27

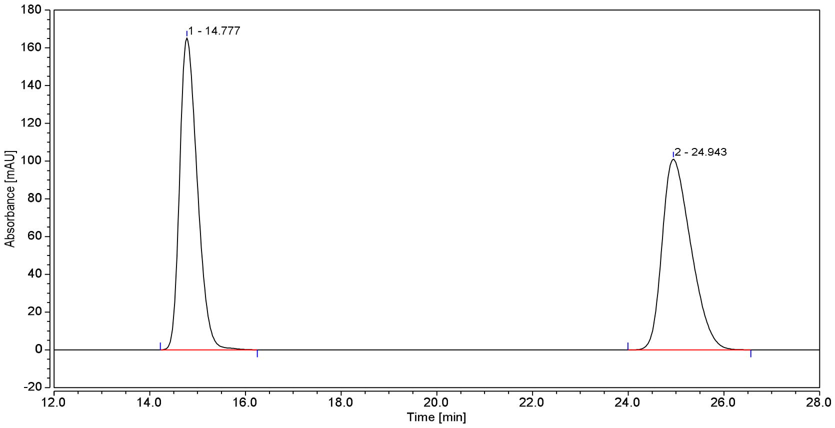

No.	Peak Name	Retention Time	Area	Height	Relative Area
		min	mAU*min	mAU	%
1		14.777	70.775	165.263	50.10
2		24.943	70.501	101.085	49.90
**Total:**			**141.276**	**266.348**	**100.00**

<u>Asymmetric</u>

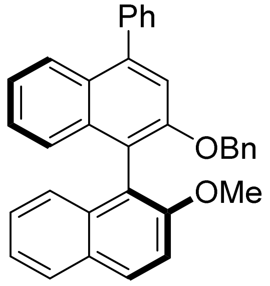

Bn-27

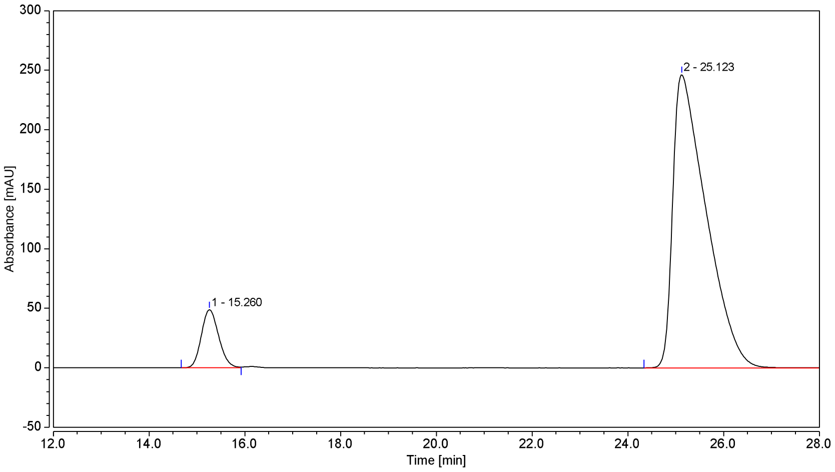

No.	Peak Name	Retention Time	Area	Height	Relative Area
		min	mAU*min	mAU	%
1		15.260	20.614	48.811	9.38
2		25.123	199.080	246.262	90.62
**Total:**			**219.694**	**295.073**	**100.00**

Chiral HPLC: (Chiralpak ADH, 10% ^{i}PrOH, 90% hexane, 1.0 mL min^{-1}, λ = 293 nm) τ_R (major) = 13.2 min, τ_R (minor) = 23.0 min.

Racemic

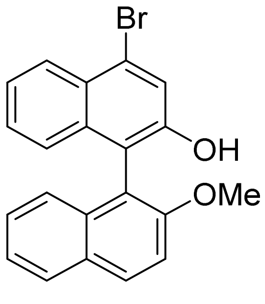

28

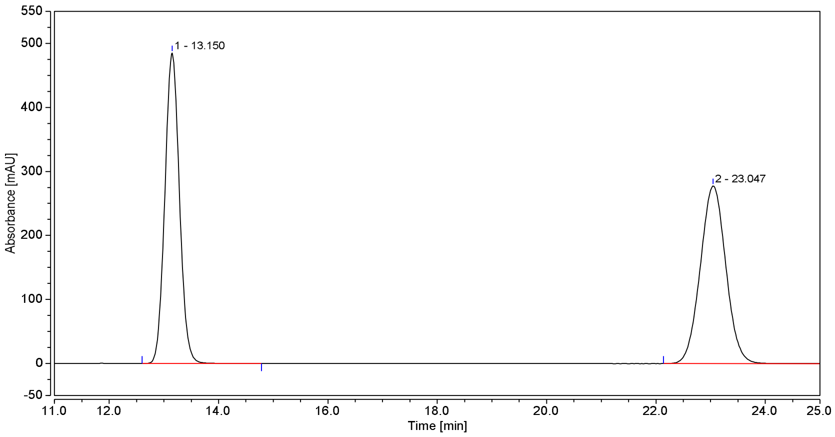

No.	Peak Name	Retention Time	Area	Height	Relative Area
		min	mAU*min	mAU	%
1		13.150	150.027	484.912	49.93
2		23.047	150.418	277.508	50.07
**Total:**			**300.445**	**762.420**	**100.00**

Asymmetric

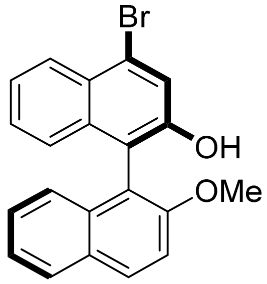

28

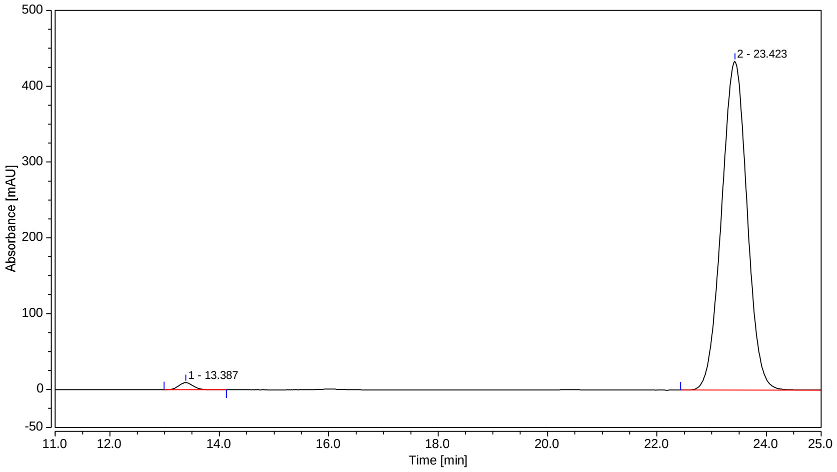

No.	Peak Name	Retention Time	Area	Height	Relative Area
		min	mAU*min	mAU	%
1		13.387	2.927	9.496	1.23
2		23.423	234.693	433.790	98.77
**Total:**			**237.620**	**443.285**	**100.00**

Chiral HPLC: (Chiralpak IC, 1% ^{i}PrOH, 99% hexane, 1.0 mL min^{-1}, λ = 232 nm) τ_R (major) = 15.0 min, τ_R (minor) = 18.0 min.

Racemic.

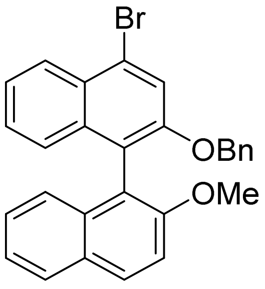

Bn-28

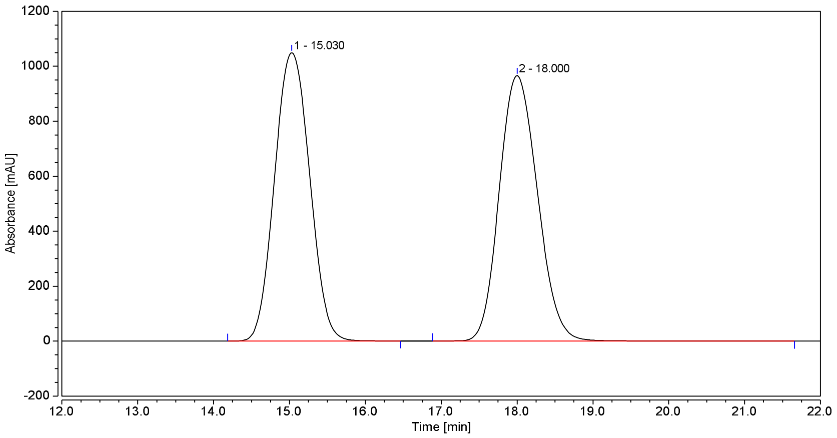

No.	Peak Name	Retention Time	Area	Height	Relative Area
		min	mAU*min	mAU	%
1		15.03	564.591	1049.961	49.96
2		18.00	565.473	966.211	50.04
**Total:**			**1130.065**	**2016.171**	**100.00**

Asymmetric

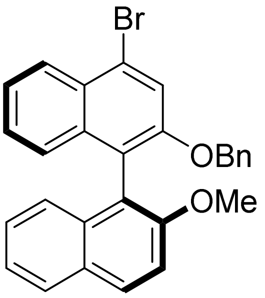

Bn-28

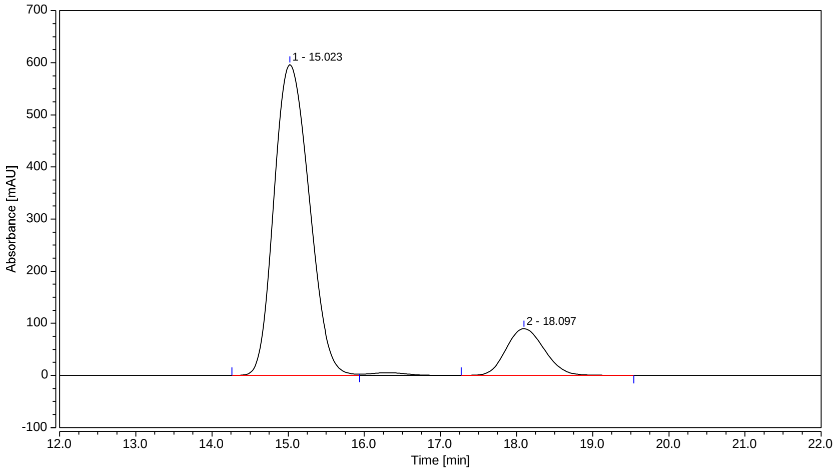

No.	Peak Name	Retention Time	Area	Height	Relative Area
		min	mAU*min	mAU	%
1		15.023	319.318	596.470	86.01
2		18.097	51.944	89.643	13.99
**Total:**			**371.262**	**686.113**	**100.00**

Chiral HPLC: (Chiralpak ADH, 15% ^{i}PrOH, 85% hexane, 1.0 mL min^{-1}, λ = 231 nm) τ_R (minor) = 8.7 min, τ_R (major) = 12.7 min.

Racemic

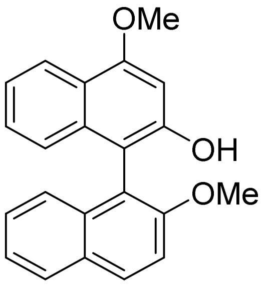

29

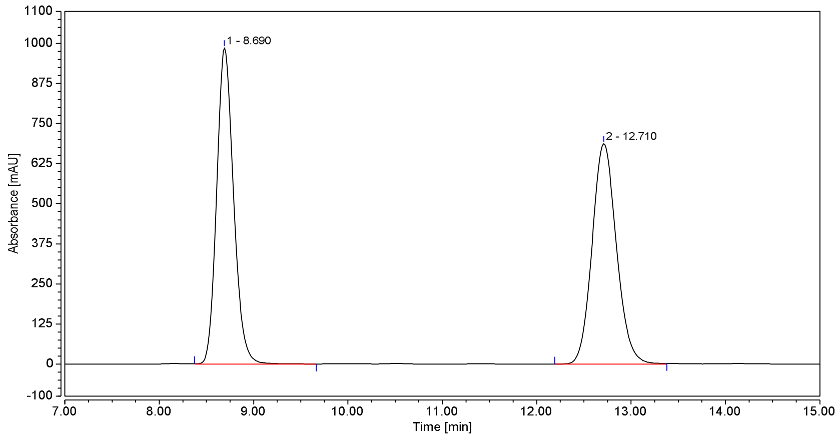

No.	Peak Name	Retention Time	Area	Height	Relative Area
		min	mAU*min	mAU	%
1		8.690	205.251	986.114	49.98
2		12.710	205.385	687.335	50.02
**Total:**			**410.636**	**1673.448**	**100.00**

Asymmetric

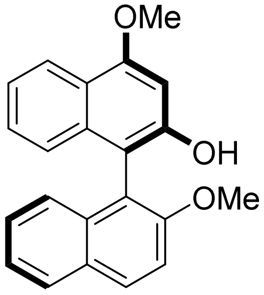

29

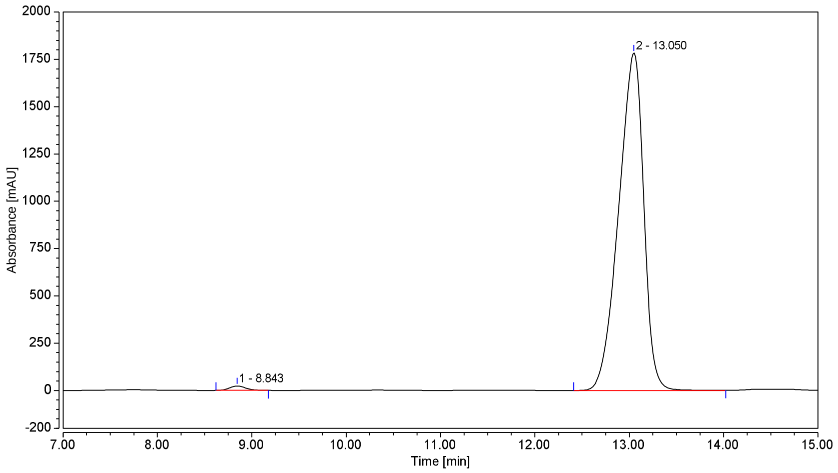

No.	Peak Name	Retention Time	Area	Height	Relative Area
		min	mAU*min	mAU	%
1		8.843	4.719	23.320	0.85
2		13.050	548.083	1783.682	99.15
**Total:**			**552.802**	**1807.002**	**100.00**

Chiral HPLC: (Chiralpak IC, 3% ^{i}PrOH, 97% hexane, 1.0 mL min^{-1}, λ = 292 nm) τ_R (major) = 6.1 min, τ_R (minor) = 8.2 min.

Racemic.

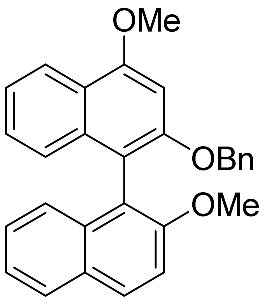

Bn-29

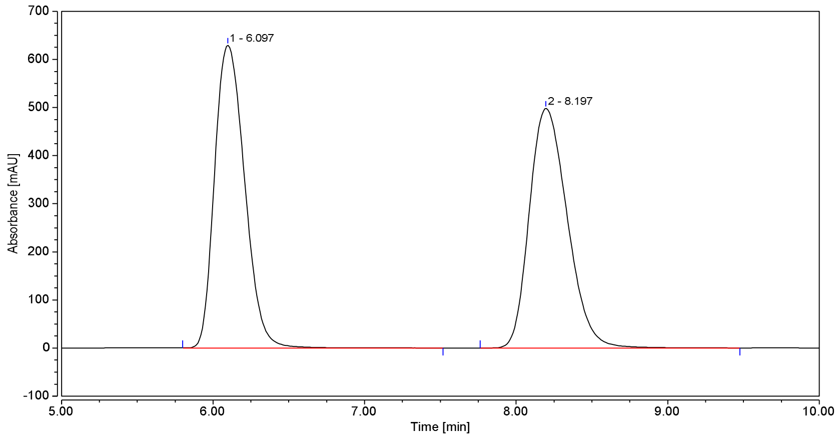

No.	Peak Name	Retention Time	Area	Height	Relative Area
		min	mAU*min	mAU	%
1		6.097	144.632	629.007	49.99
2		8.197	144.708	497.911	50.01
**Total:**			**289.339**	**1126.917**	**100.00**

Asymmetric

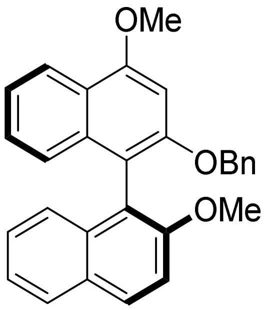

Bn-29

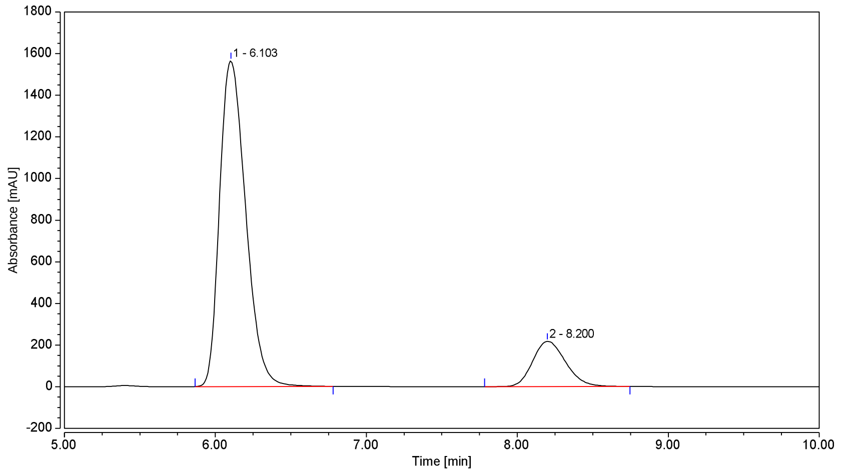

No.	Peak Name	Retention Time	Area	Height	Relative Area
		min	mAU*min	mAU	%
1		6.103	312.866	1564.775	85.03
2		8.200	55.087	217.401	14.97
**Total:**			**367.953**	**1782.176**	**100.00**

Chiral HPLC: (Chiralpak ADH, 5% ^{i}PrOH, 95% hexane, 1.00 mL min^{-1}, λ = 290 nm) τ_R (major) = 26.7 min, τ_R (minor) = 21.7 min

Racemic

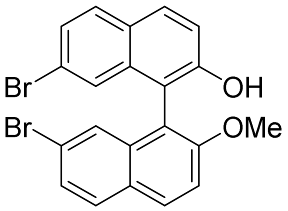

30

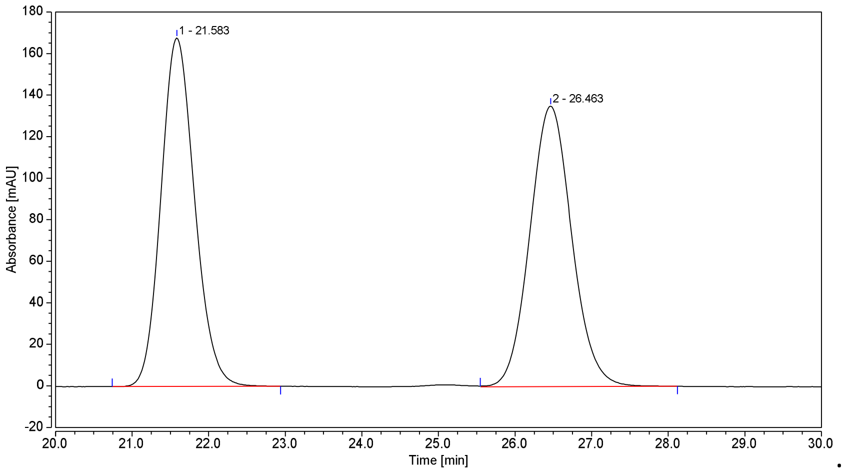

No.	Peak Name	Retention Time	Area	Height	Relative Area
		min	mAU*min	mAU	%
1		21.583	85.348	167.794	49.86
2		26.463	85.823	134.988	50.14
**Total:**			171.172	302.782	100.00

Asymmetric

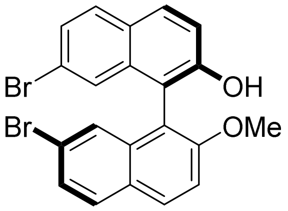

30

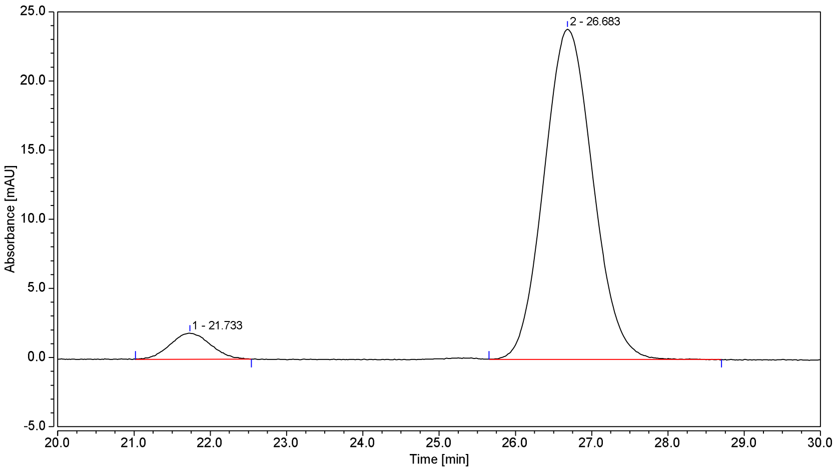

No.	Peak Name	Retention Time	Area	Height	Relative Area
		min	mAU*min	mAU	%
1		21.733	1.167	1.883	6.18
2		26.683	17.708	23.878	93.82
**Total:**			**18.875**	**25.760**	**100.00**

Chiral HPLC: (Chiralpak ADH, 5% ^{i}PrOH, 95% hexane, 1.00 mL min^{-1}, λ = 290nm, 15 μL injection) τ_R (major) = 7.6 min, τ_R (minor) = 6.6 min

<u>**Racemic**</u>

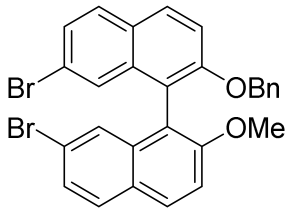

Bn-30

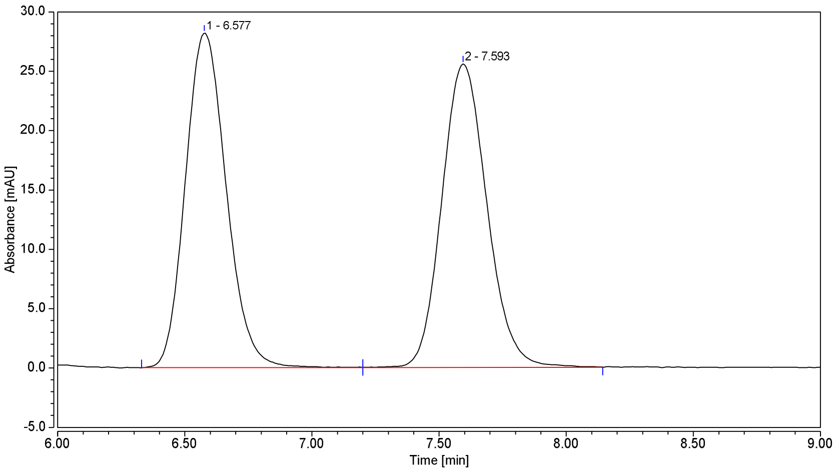

No.	Peak Name	Retention Time	Area	Height	Relative Area
		min	mAU*min	mAU	%
1		6.577	5.250	28.193	49.97
2		7.593	5.257	25.556	50.03
**Total:**			**10.507**	**53.749**	**100.00**

<u>**Asymmetric**</u>

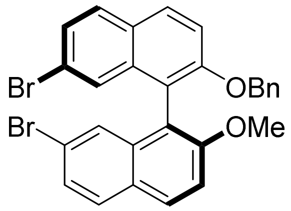

Bn-30

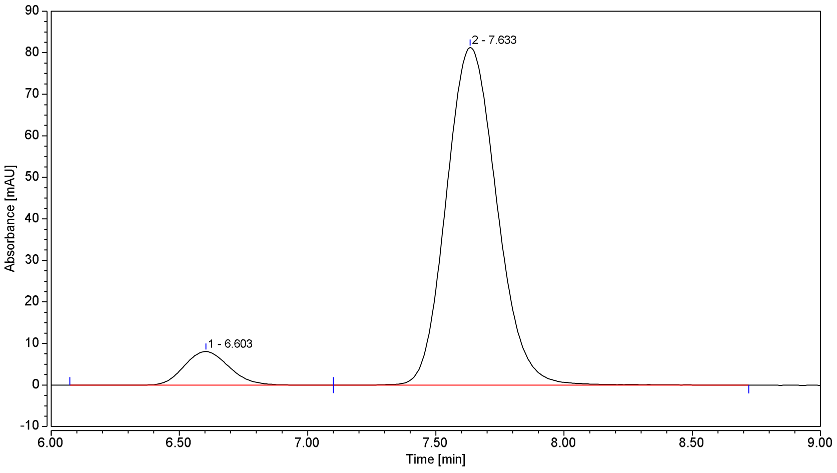

No.	Peak Name	Retention Time	Area	Height	Relative Area
		min	mAU*min	mAU	%
1		6.603	1.632	8.073	8.08
2		7.633	18.574	81.266	91.92
**Total:**			**20.206**	**89.339**	**100.00**

Chiral HPLC: (Chiralpak ADH, 5% ^{i}PrOH, 95% hexane, 1.00 mL min^{-1}, λ = 290 nm) τ_R (major) = 47.8 min, τ_R (minor) = 40.7 min

Racemic

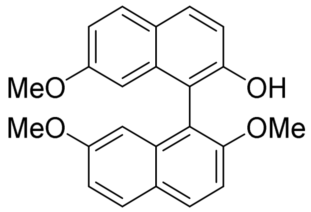

31

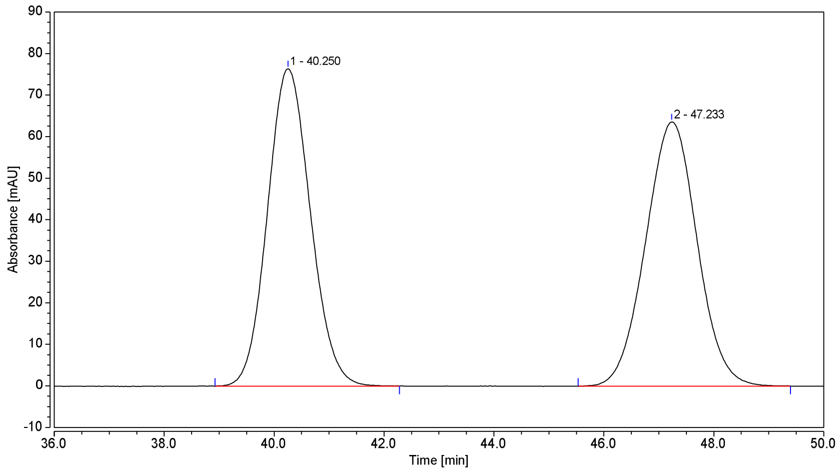

No.	Peak Name	Retention Time	Area	Height	Relative Area
		min	mAU*min	mAU	%
1		40.250	69.130	76.397	49.94
2		47.233	69.285	63.653	50.06
**Total:**			**138.414**	**140.050**	**100.00**

Asymmetric

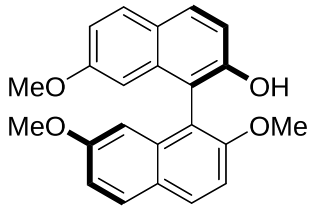

31

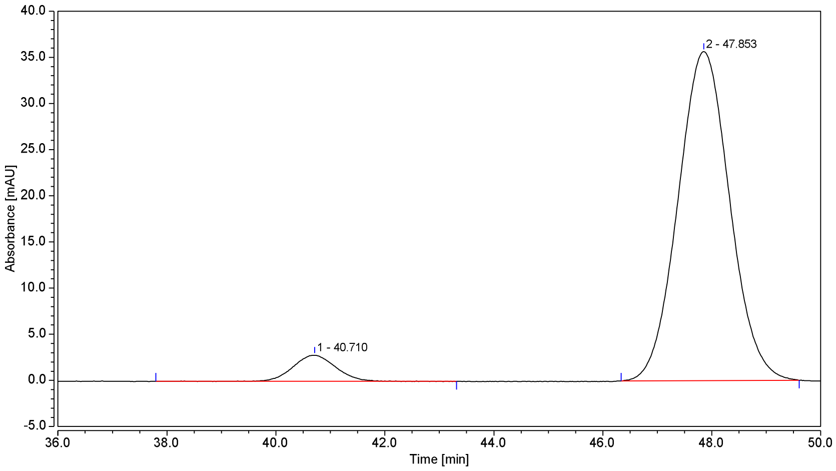

No.	Peak Name	Retention Time	Area	Height	Relative Area
		min	mAU*min	mAU	%
1		40.710	2.527	2.827	6.07
2		47.853	39.143	35.658	93.93
**Total:**			**41.670**	**38.485**	**100.00**

Chiral HPLC: (Chiralpak ADH, 5% ^{i}PrOH, 95% hexane, 1.00 mL min^{-1}, λ = 290 nm) τ_R (major) = 27.4 min, τ_R (minor) = 11.6 min

Racemic

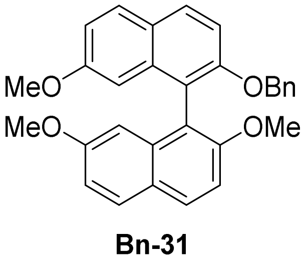

Bn-31

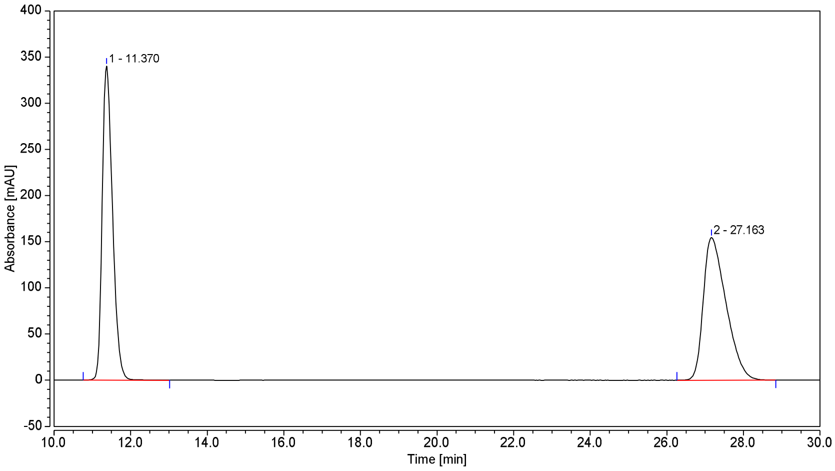

No.	Peak Name	Retention Time	Area	Height	Relative Area
		min	mAU*min	mAU	%
1		11.370	106.766	340.371	50.11
2		27.163	106.286	154.589	49.89
**Total:**			**213.053**	**494.960**	**100.00**

Asymmetric

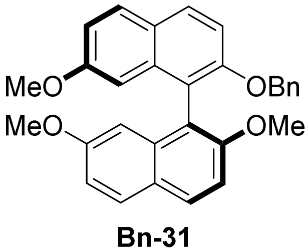

Bn-31

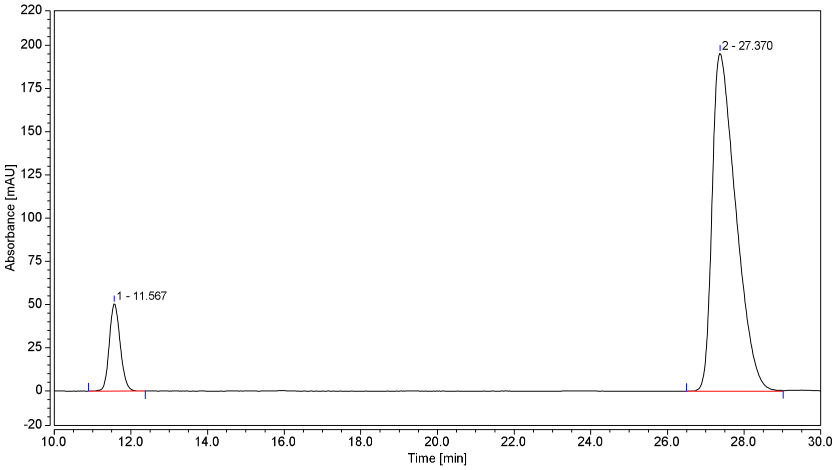

No.	Peak Name	Retention Time	Area	Height	Relative Area
		min	mAU*min	mAU	%
1		11.567	16.670	50.640	10.64
2		27.370	140.042	195.476	89.36
**Total:**			**156.712**	**246.116**	**100.00**

Chiral HPLC: (Chiralpak ADH, 5% ^{i}PrOH, 95% hexane, 1.00 mL min^{-1}, λ = 290 nm) τ_R (major) = 19.5 min, τ_R (minor) = 22.1 min

Racemic

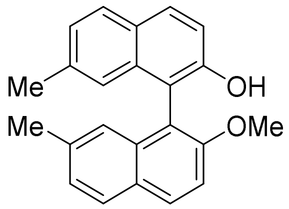

32

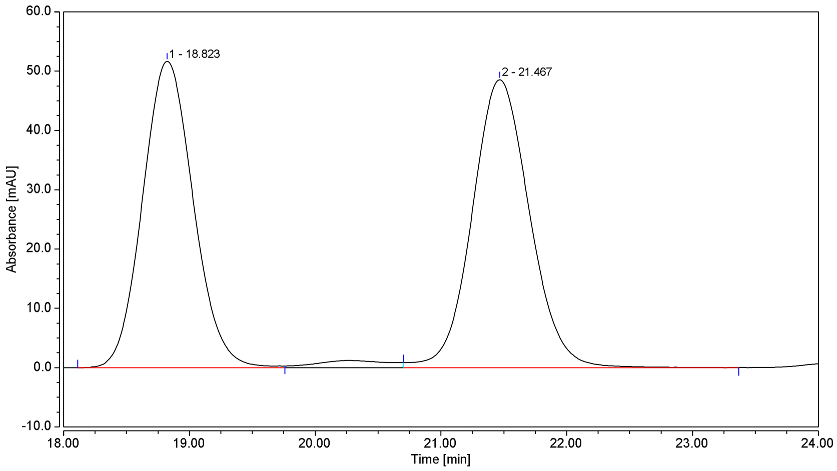

No.	Peak Name	Retention Time	Area	Height	Relative Area
		min	mAU*min	mAU	%
1		18.823	24.144	51.676	48.46
2		21.467	25.683	48.561	51.54
**Total:**			**49.827**	**100.237**	**100.00**

Asymmetric

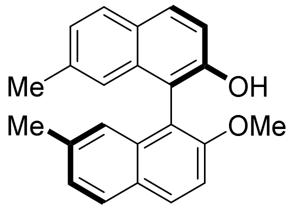

32

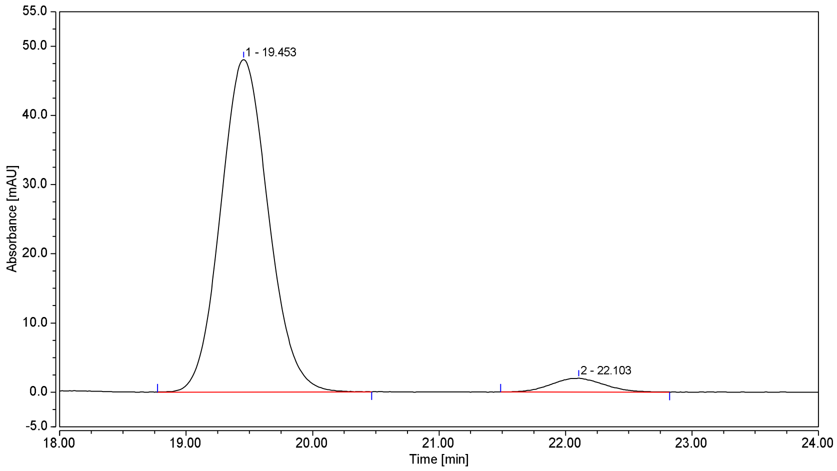

No.	Peak Name	Retention Time	Area	Height	Relative Area
		min	mAU*min	mAU	%
1		19.453	21.026	48.052	95.59
2		22.103	0.970	2.003	4.41
**Total:**			**21.996**	**50.055**	**100.00**

Chiral HPLC: (Chiralpak ADH, 5% ^{i}PrOH, 95% hexane, 1.00 mL min^{-1}, λ = 290 nm) τ_R (major) = 8.4 min, τ_R (minor) = 5.5 min

Racemic

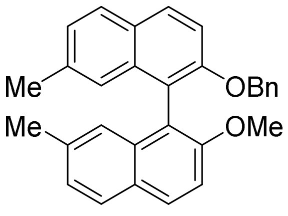

Bn-32

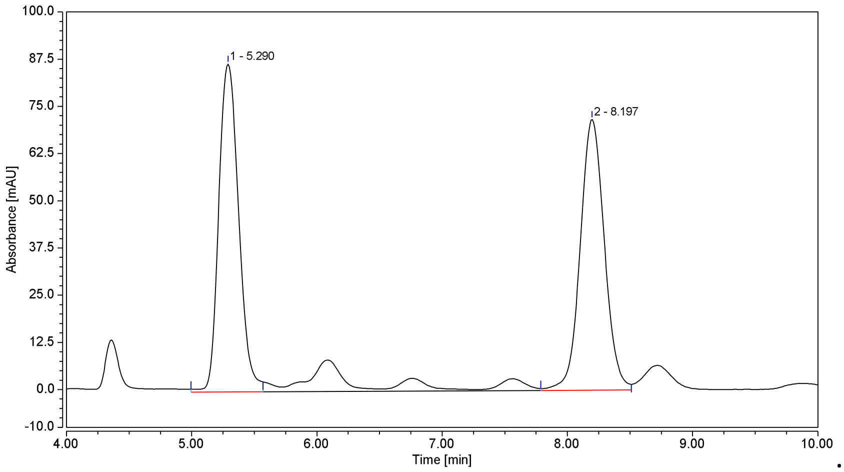

No.	Peak Name	Retention Time	Area	Height	Relative Area
		min	mAU*min	mAU	%
1		5.290	16.034	86.890	50.01
2		8.197	16.025	71.741	49.99
**Total:**			**32.060**	**158.631**	**100.00**

Asymmetric

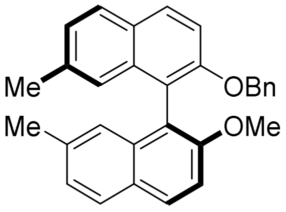

Bn-32

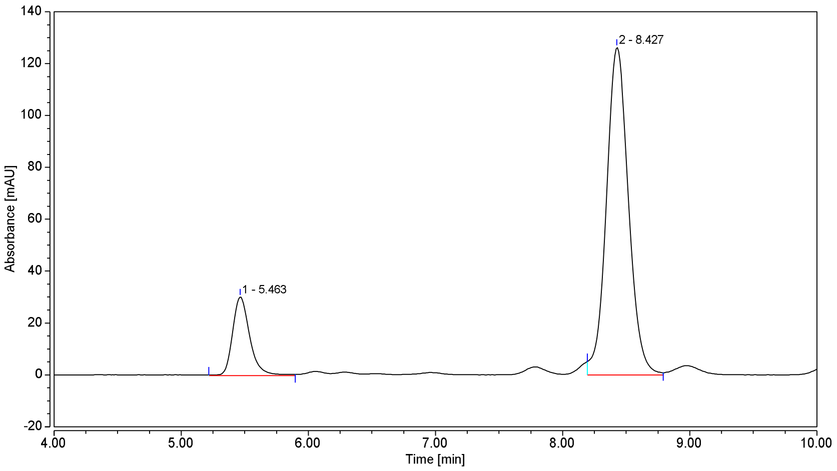

No.	Peak Name	Retention Time	Area	Height	Relative Area
		min	mAU*min	mAU	%
1		5.463	4.831	30.360	15.96
2		8.427	25.435	126.259	84.04
**Total:**			**30.266**	**156.619**	**100.00**

Chiral HPLC: (Chiralpak ADH, 7.5% ^{i}PrOH, 92.5% hexane, 1.00 mL min^{-1}, λ = 290 nm) τ_R (major) = 25.4 min, τ_R (minor) = 20.2 min

Racemic

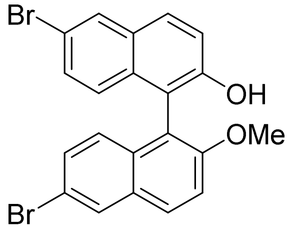

33

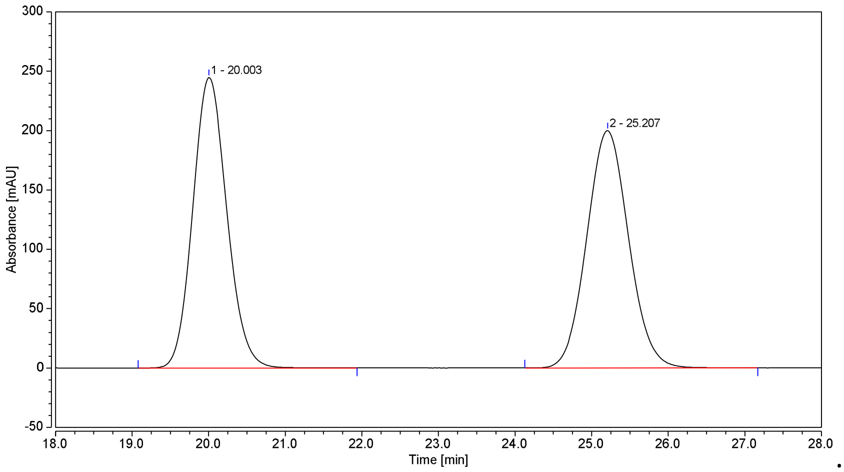

No.	Peak Name	Retention Time	Area	Height	Relative Area
		min	mAU*min	mAU	%
1		20.003	126.546	244.803	49.99
2		25.207	126.597	199.952	50.01
**Total:**			**253.144**	**444.755**	**100.00**

Asymmetric

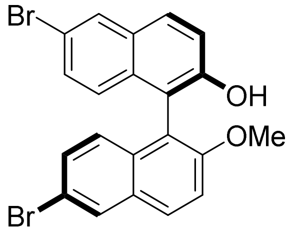

33

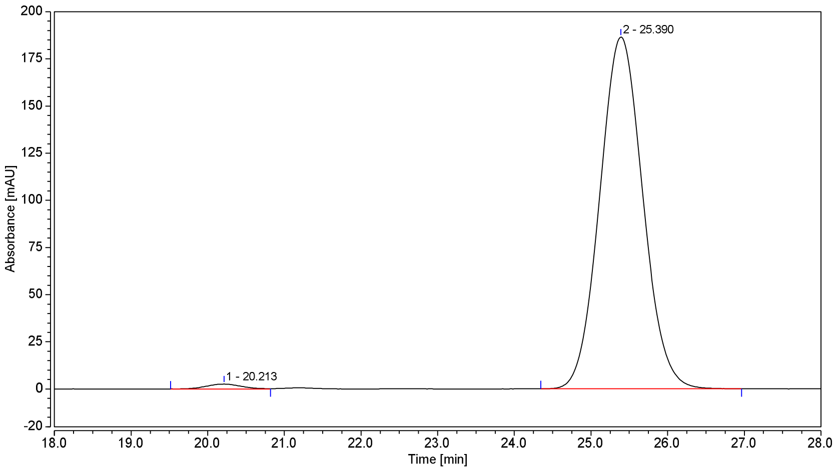

No.	Peak Name	Retention Time	Area	Height	Relative Area
		min	mAU*min	mAU	%
1		20.213	1.379	2.636	1.12
2		25.390	121.390	186.520	98.88
**Total:**			**122.769**	**189.156**	**100.00**

Chiral HPLC: (Chiralpak ADH, 7.5% ^{i}PrOH, 92.5% hexane, 1.00 mL min^{-1}, λ = 290 nm) τ_R (major) = 13.3 min, τ_R (minor) = 10.8 min

Racemic

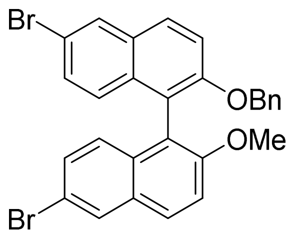

Bn-33

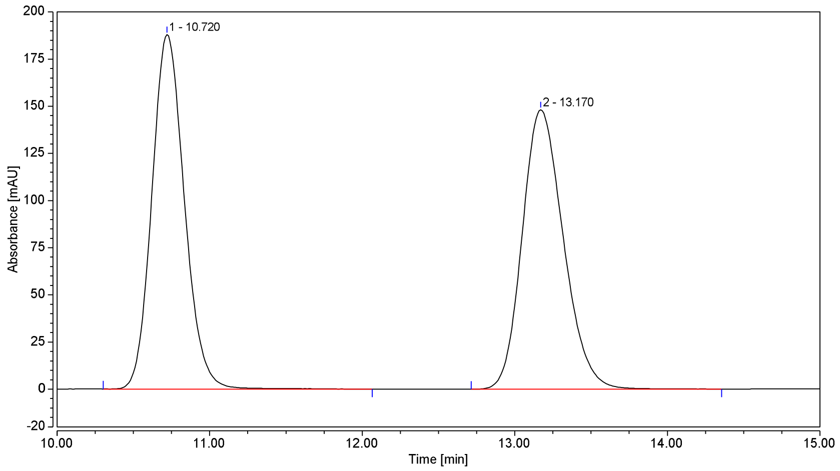

No.	Peak Name	Retention Time	Area	Height	Relative Area
		min	mAU*min	mAU	%
1		10.720	46.477	187.937	50.03
2		13.170	46.425	148.219	49.97
**Total:**			**92.902**	**336.156**	**100.00**

Asymmetric

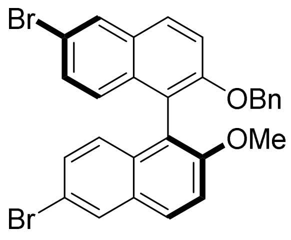

Bn-33

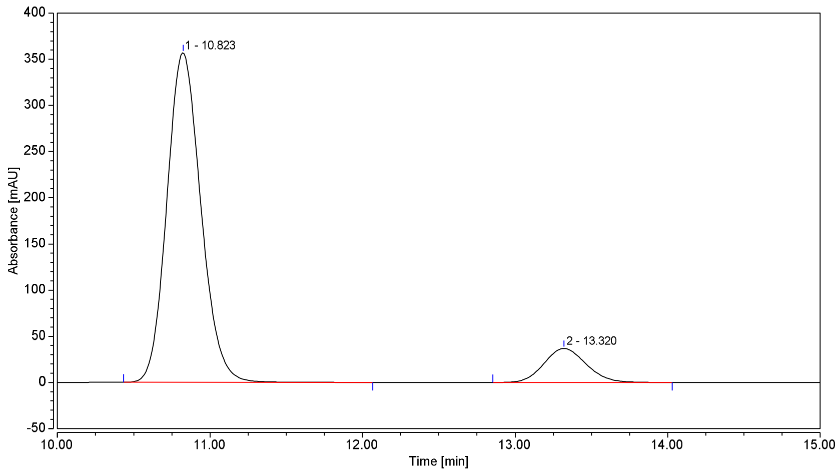

No.	Peak Name	Retention Time	Area	Height	Relative Area
		min	mAU*min	mAU	%
1		10.823	92.028	356.972	88.64
2		13.320	11.789	36.737	11.36
**Total:**			**103.816**	**393.709**	**100.00**

Chiral HPLC: (Chiralpak ADH, 12.5% ^{i}PrOH, 87.5% hexane, 1.00 mL min^{-1}, λ = 290 nm) τ_R (major) = 25.6 min, τ_R (minor) = 17.7 min

Racemic

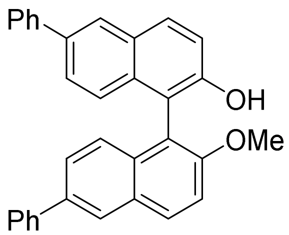

34

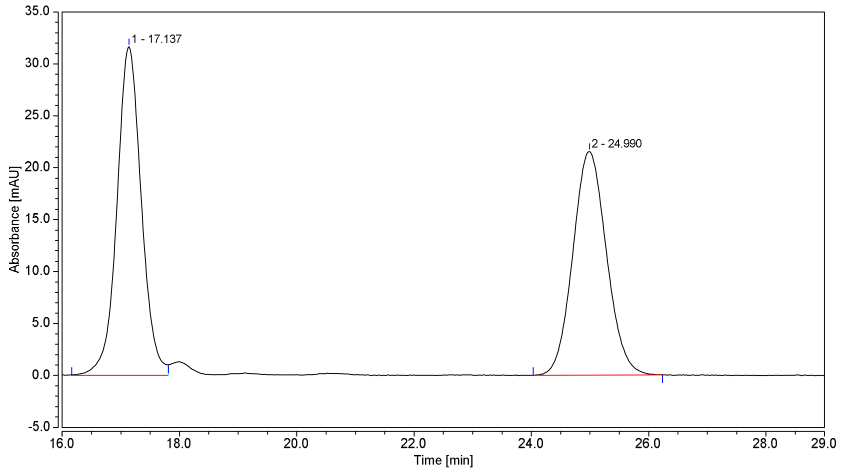

No.	Peak Name	Retention Time	Area	Height	Relative Area
		min	mAU*min	mAU	%
1		17.137	15.226	31.639	51.50
2		24.990	14.338	21.545	48.50
**Total:**			**29.565**	**53.183**	**100.00**

Asymmetric

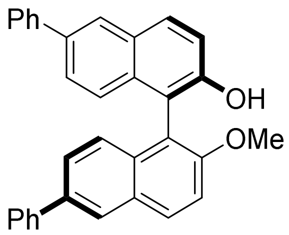

34

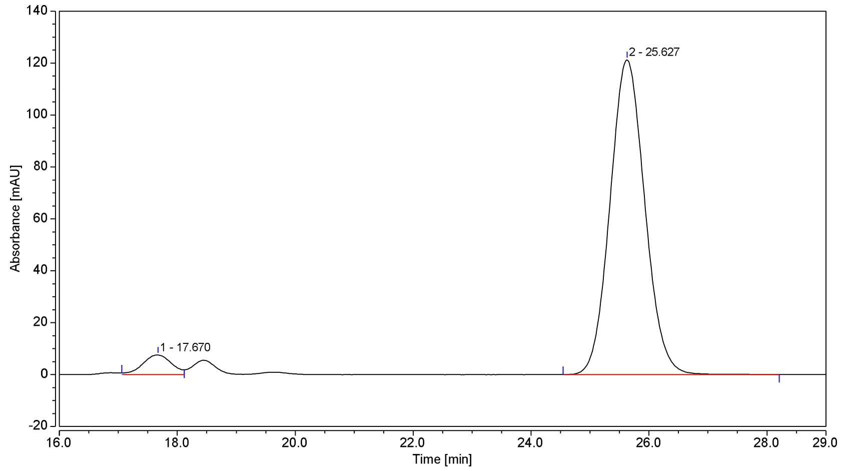

No.	Peak Name	Retention Time	Area	Height	Relative Area
		min	mAU*min	mAU	%
1		17.670	4.308	7.605	4.95
2		25.627	82.806	121.263	95.05
**Total:**			**87.114**	**128.868**	**100.00**

Chiral HPLC: (Chiralpak ADH, 12.5% ^{i}PrOH, 87.5% hexane, 1.00 mL min^{-1}, λ = 290 nm) τ_R (major) = 12.3 min, τ_R (minor) = 19.5 min

Racemic

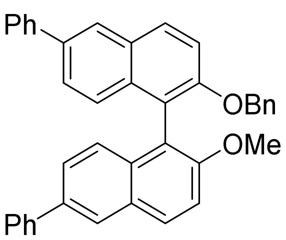

Bn-34

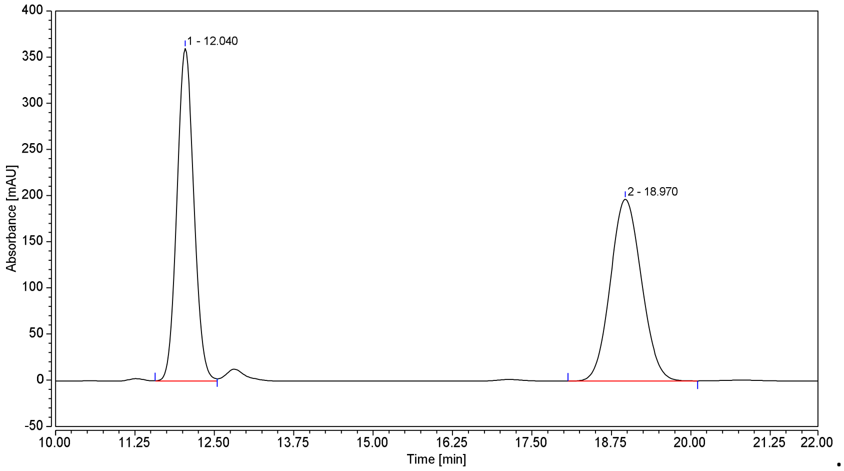

No.	Peak Name	Retention Time	Area	Height	Relative Area
		min	mAU*min	mAU	%
1		12.040	112.038	360.412	50.04
2		18.970	111.864	196.950	49.96
**Total:**			**223.902**	**557.362**	**100.00**

Asymmetric

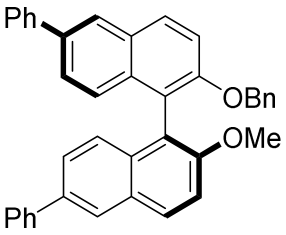

Bn-34

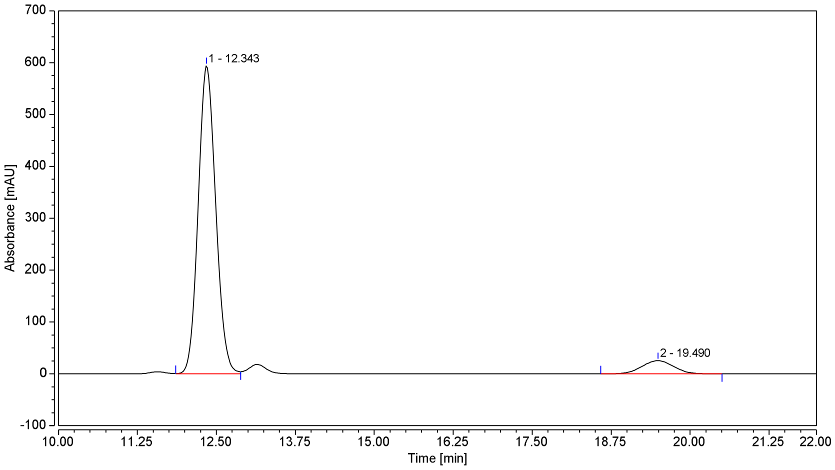

No.	Peak Name	Retention Time	Area	Height	Relative Area
		min	mAU*min	mAU	%
1		12.343	191.597	593.832	92.78
2		19.490	14.906	25.527	7.22
**Total:**			**206.504**	**619.359**	**100.00**

Chiral HPLC: (Chiralpak ADH, 12.5% ^{i}PrOH, 87.5% hexane, 1.00 mL min^{-1}, λ = 290 nm) τ_R (major) = 41.2 min, τ_R (minor) = 24.3 min

Racemic

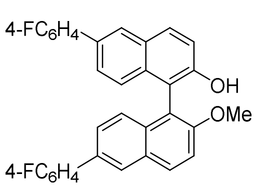

35

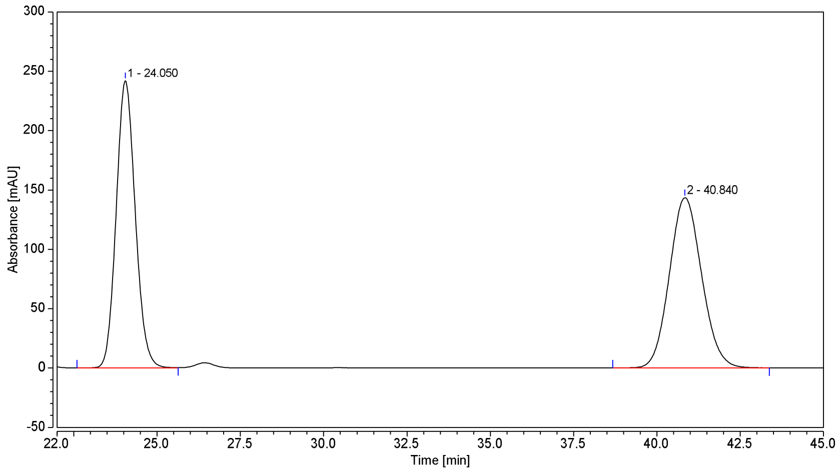

No.	Peak Name	Retention Time	Area	Height	Relative Area
		min	mAU*min	mAU	%
1		24.050	160.639	242.200	49.94
2		40.840	161.038	143.426	50.06
**Total:**			**321.677**	**385.626**	**100.00**

Asymmetric

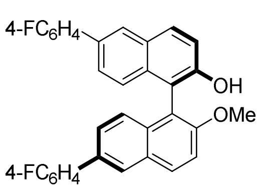

35

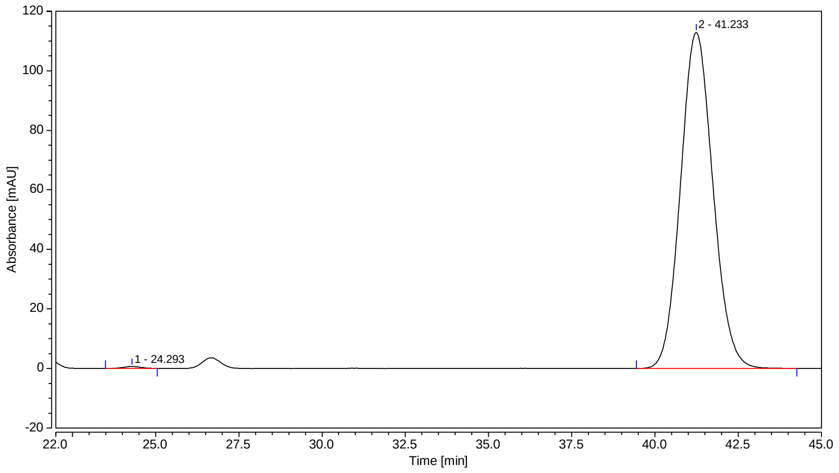

No.	Peak Name	Retention Time	Area	Height	Relative Area
		min	mAU*min	mAU	%
1		24.293	0.435	0.673	0.34
2		41.233	127.384	112.925	99.66
**Total:**			**127.819**	**113.598**	**100.00**

Chiral HPLC: (Chiralpak ADH, 12.5% ^{i}PrOH, 87.5% hexane, 1.00 mL min^{-1}, λ = 290 nm) τ_R (major) = 16.6 min, τ_R (minor) = 26.6 min

Racemic

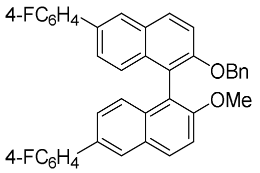

Bn-35

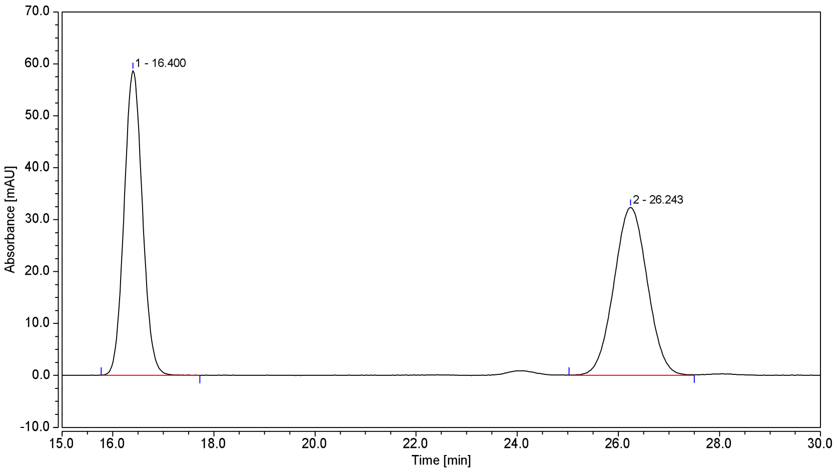

No.	Peak Name	Retention Time	Area	Height	Relative Area
		min	mAU*min	mAU	%
1		16.400	24.662	58.657	49.86
2		26.243	24.799	32.337	50.14
**Total:**			49.460	90.994	100.00

Asymmetric

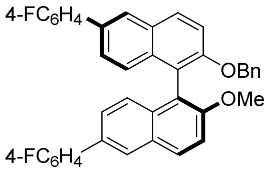

Bn-35

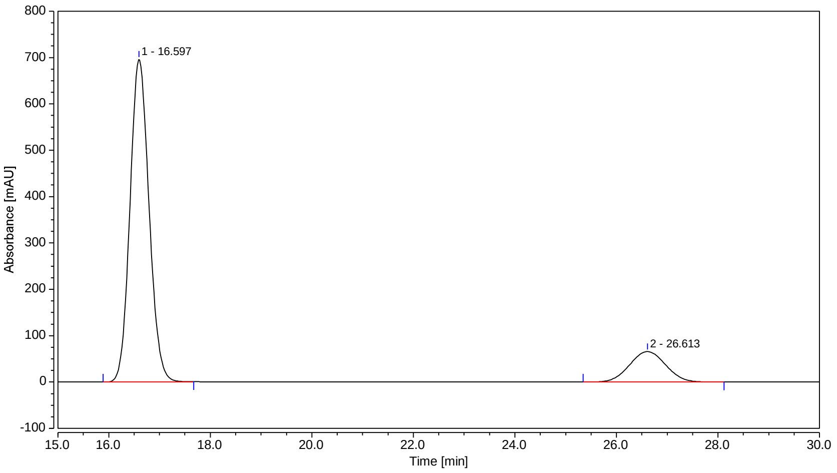

No.	Peak Name	Retention Time	Area	Height	Relative Area
		min	mAU*min	mAU	%
1		16.597	302.471	696.897	84.77
2		26.613	54.325	65.720	15.23
**Total:**			**356.796**	**762.617**	**100.00**

Chiral HPLC: (Chiralpak ADH, 50% ^{i}PrOH, 50% hexane, 1.00 mL min^{-1}, λ = 290 nm) τ_R (major) = 27.8 min, τ_R (minor) = 17.7 min

Racemic

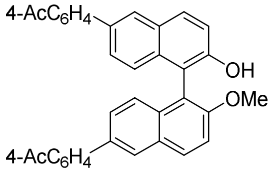

36

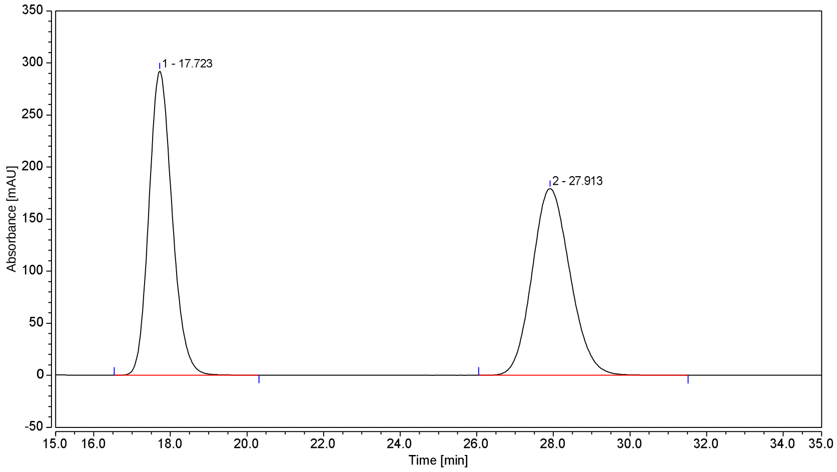

No.	Peak Name	Retention Time	Area	Height	Relative Area
		min	mAU*min	mAU	%
1		17.723	205.884	292.089	49.99
2		27.913	205.966	179.368	50.01
**Total:**			**411.850**	**471.457**	**100.00**

Asymmetric

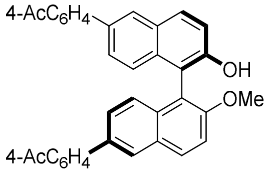

36

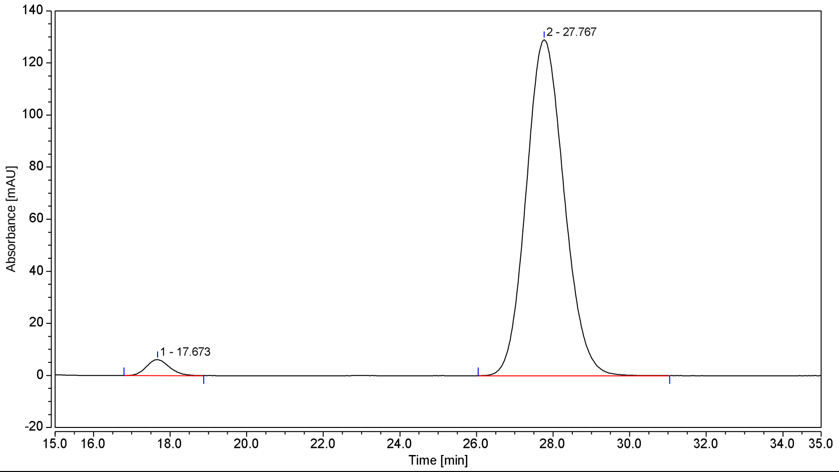

No.	Peak Name	Retention Time	Area	Height	Relative Area
		min	mAU*min	mAU	%
1		17.673	4.365	6.183	2.86
2		27.767	148.109	129.083	97.14
Total:			**152.475**	**135.266**	**100.00**

Chiral HPLC: (Chiralpak ADH, 50% ^{i}PrOH, 50% hexane, 1.00 mL min^{-1}, λ = 290 nm) τ_R (major) = 37.6 min, τ_R (minor) = 42.0 min

Racemic

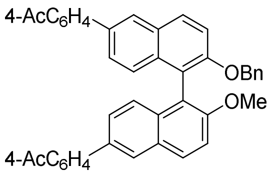

Bn-36

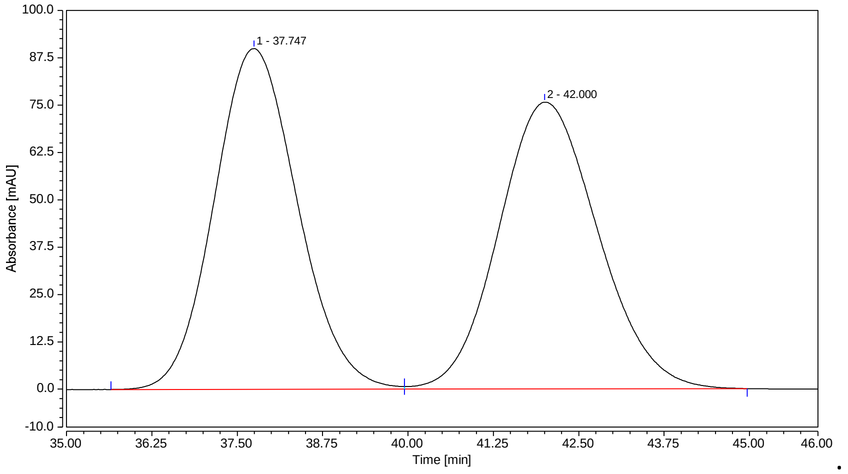

No.	Peak Name	Retention Time	Area	Height	Relative Area
		min	mAU*min	mAU	%
1		37.747	127.339	89.946	49.97
2		42.000	127.500	75.692	50.03
Total:			**254.840**	**165.639**	**100.00**

Asymmetric

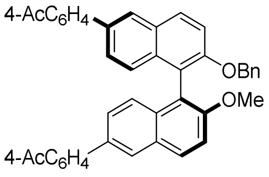

Bn-36

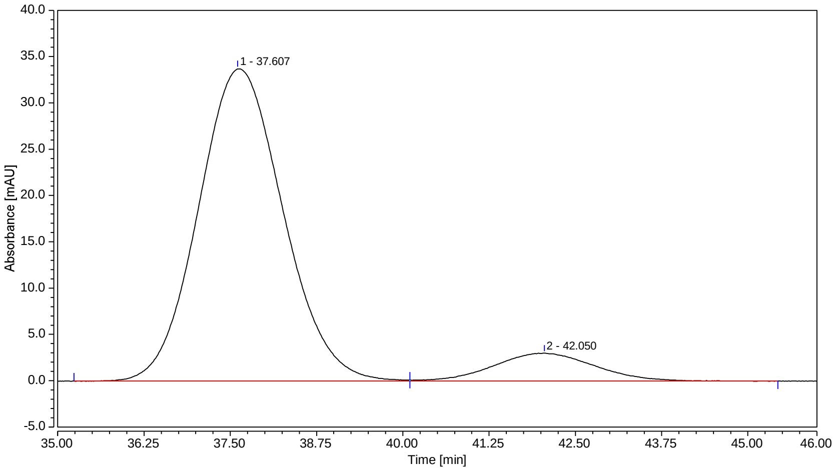

No.	Peak Name	Retention Time	Area	Height	Relative Area
		min	mAU*min	mAU	%
1		37.607	47.923	33.724	90.42
2		42.050	5.078	3.014	9.58
**Total:**			**53.001**	**36.738**	**100.00**

Chiral HPLC: (Chiralpak ADH, 12.5% ^{i}PrOH, 87.5% hexane, 1.00 mL min^{-1}, λ = 290 nm) τ_R (major) = 16.6 min, τ_R (minor) = 12.4 min

Racemic

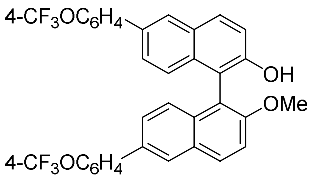

37

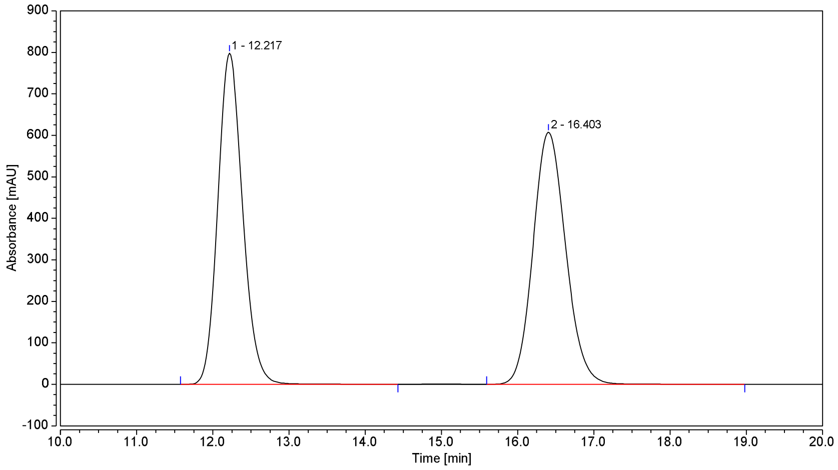

No.	Peak Name	Retention Time	Area	Height	Relative Area
		min	mAU*min	mAU	%
1		12.217	301.003	797.855	49.97
2		16.403	301.365	607.620	50.03
**Total:**			**602.368**	**1405.475**	**100.00**

Asymmetric

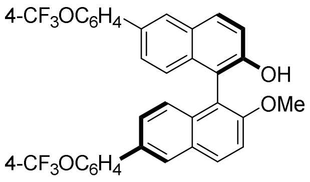

37

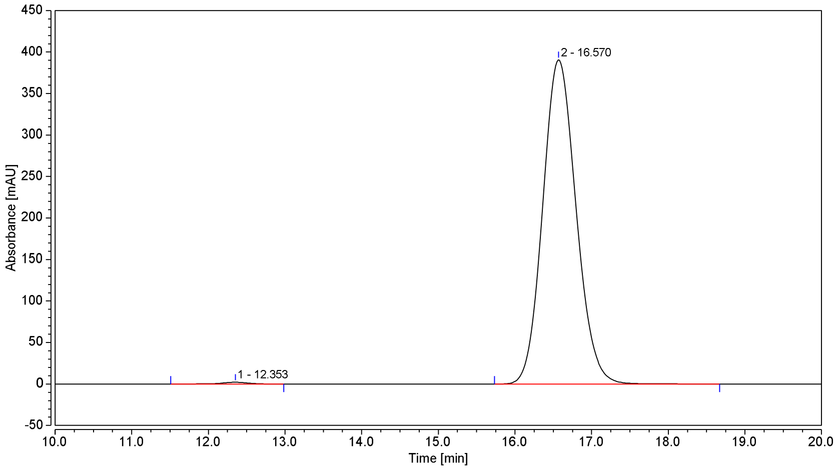

No.	Peak Name	Retention Time	Area	Height	Relative Area
		min	mAU*min	mAU	%
1		12.353	0.943	2.195	0.48
2		16.570	196.598	390.760	99.52
**Total:**			**197.541**	**392.955**	**100.00**

Chiral HPLC: (Chiralpak IC, 1% ^{i}PrOH, 99% hexane, 1.00 mL min^{-1}, λ = 290 nm) τ_R (major) = 6.9 min, τ_R (minor) = 9.6 min

Racemic

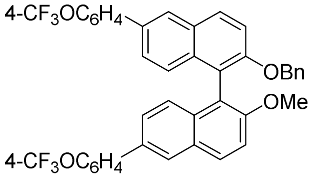

Bn-37

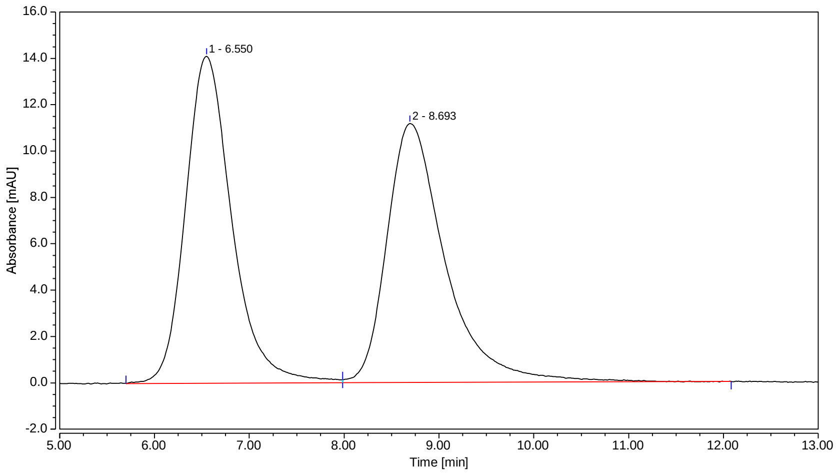

No.	Peak Name	Retention Time	Area	Height	Relative Area
		min	mAU*min	mAU	%
1		6.550	8.010	14.108	50.14
2		8.693	7.964	11.173	49.86
**Total:**			**15.974**	**25.281**	**100.00**

Asymmetric

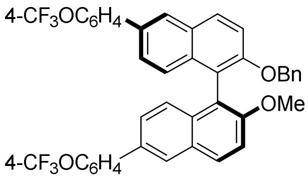

Bn-37

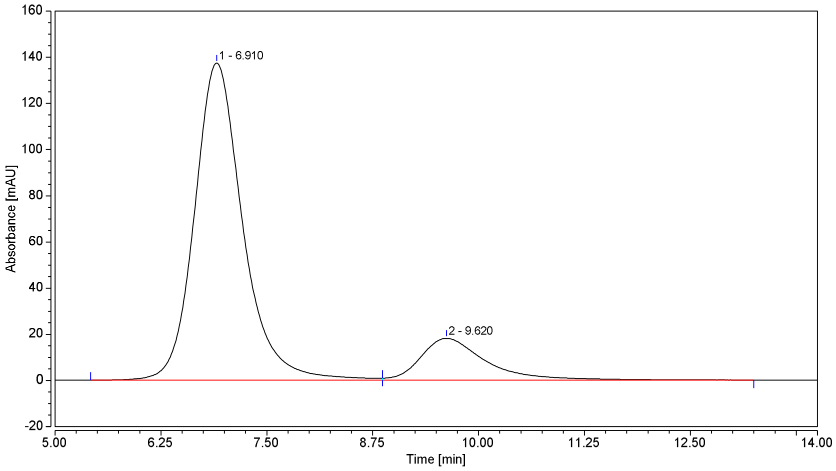

No.	Peak Name	Retention Time	Area	Height	Relative Area
		min	mAU*min	mAU	%
1		6.910	90.822	137.452	84.75
2		9.620	16.346	18.123	15.25
**Total:**			**107.168**	**155.575**	**100.00**

Chiral HPLC: (Chiralpak IC, 2% ^{i}PrOH, 98% hexane, 1.00 mL min^{-1}, λ = 290 nm) τ_R (major) = 11.8 min, τ_R (minor) = 23.1 min

Racemic

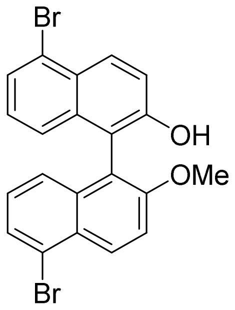

38

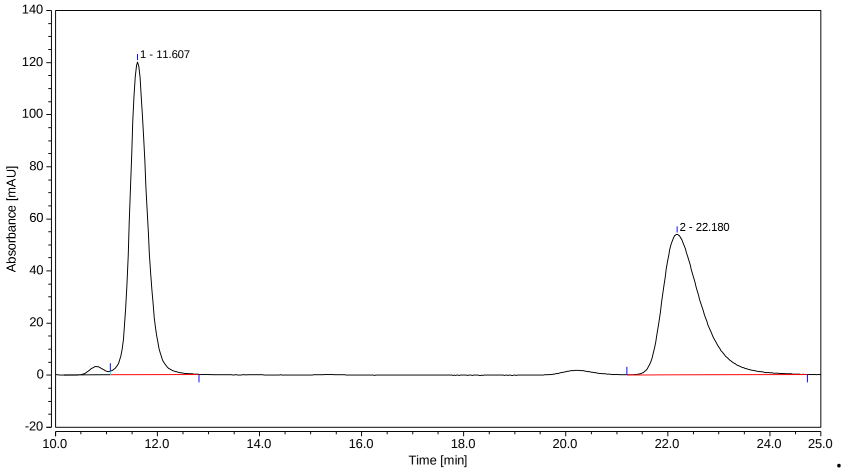

No.	Peak Name	Retention Time	Area	Height	Relative Area
		min	mAU*min	mAU	%
1		11.607	47.416	120.001	50.12
2		22.180	47.196	53.912	49.88
**Total:**			**94.612**	**173.913**	**100.00**

Asymmetric

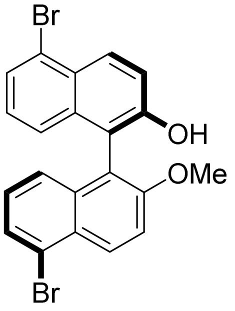

38

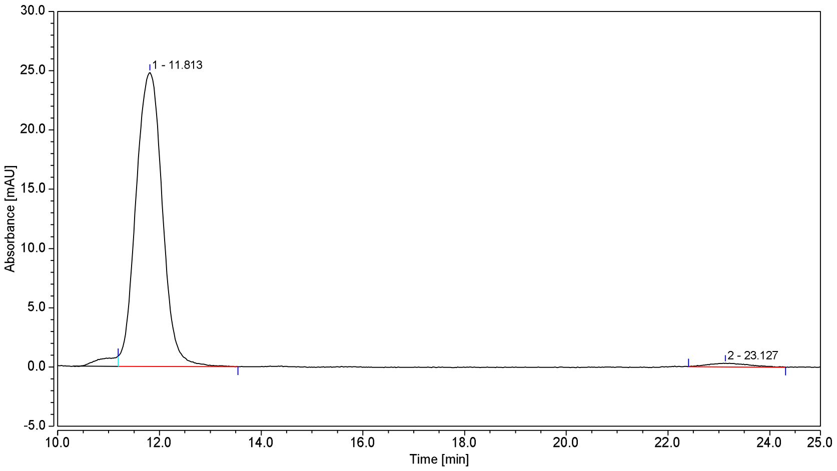

No.	Peak Name	Retention Time	Area	Height	Relative Area
		min	mAU*min	mAU	%
1		11.813	14.869	24.797	98.05
2		23.127	0.296	0.315	1.95
**Total:**			**15.165**	**25.112**	**100.00**

Chiral HPLC: (Chiralpak IC, 2% ^{i}PrOH, 98% hexane, 1.00 mL min^{-1}, λ = 290 nm) τ_R (major) = 5.2 min, τ_R (minor) = 6.9 min

Racemic

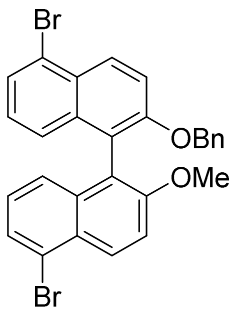

Bn-38

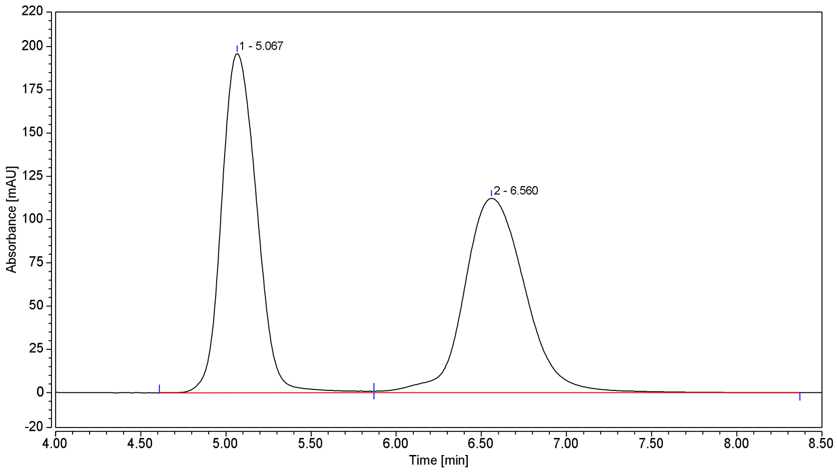

No.	Peak Name	Retention Time	Area	Height	Relative Area
		min	mAU*min	mAU	%
1		5.067	46.703	195.952	50.09
2		6.560	46.529	112.317	49.91
**Total:**			93.233	308.269	100.00

Asymmetric

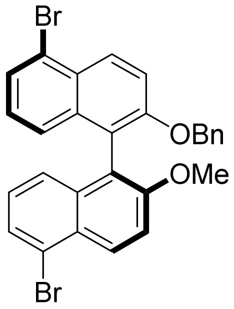

Bn-38

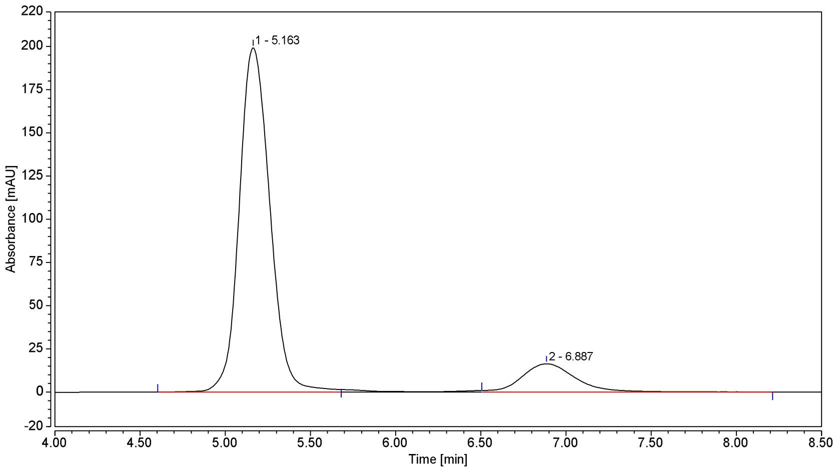

No.	Peak Name	Retention Time	Area	Height	Relative Area
		min	mAU*min	mAU	%
1		5.163	41.437	199.111	87.44
2		6.887	5.953	16.313	12.56
**Total:**			**47.390**	**215.424**	**100.00**

Chiral HPLC: (Chiralpak ADH, 12.5% ^{i}PrOH, 87.5% hexane, 1.00 mL min^{-1}, λ = 290 nm) τ_R (major) = 29.1 min, τ_R (minor) = 9.7 min

Racemic

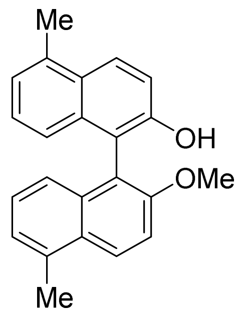

39

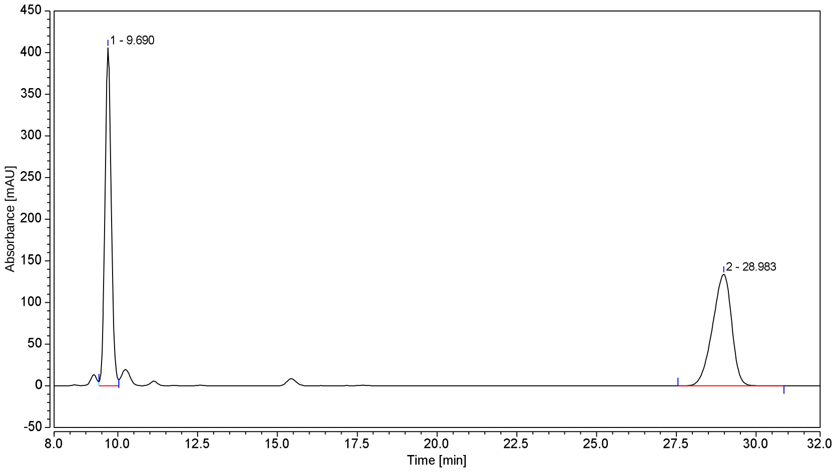

No.	Peak Name	Retention Time	Area	Height	Relative Area
		min	mAU*min	mAU	%
1		9.690	92.702	405.688	49.89
2		28.983	93.096	133.845	50.11
**Total:**			**185.798**	**539.533**	**100.00**

Asymmetric

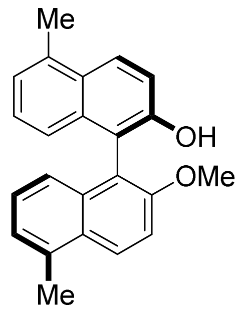

39

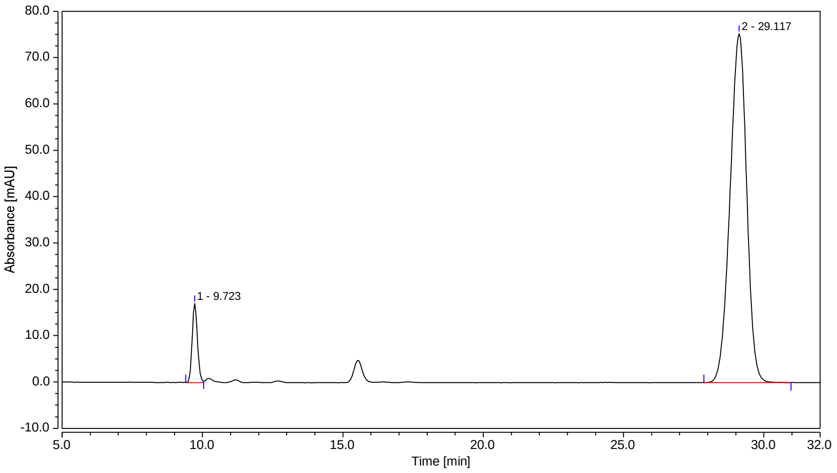

No.	Peak Name	Retention Time	Area	Height	Relative Area
		min	mAU*min	mAU	%
1		9.723	3.752	17.032	6.75
2		29.117	51.815	75.284	93.25
**Total:**			**55.567**	**92.315**	**100.00**

Chiral HPLC: (Chiralpak ADH, 12.5% ^{i}PrOH, 87.5% hexane, 1.00 mL min^{-1}, λ = 290 nm) τ_R (major) = 8.9 min, τ_R (minor) = 8.2 min

Racemic

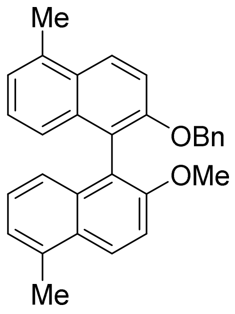

Bn-39

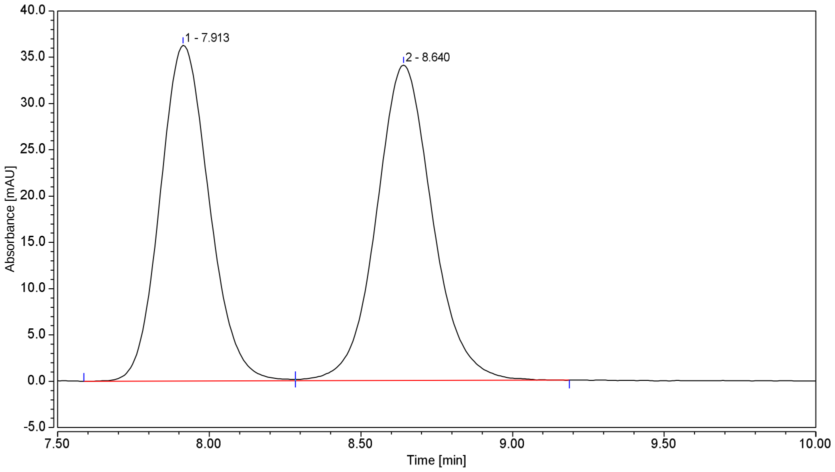

No.	Peak Name	Retention Time	Area	Height	Relative Area
		min	mAU*min	mAU	%
1		7.913	6.794	36.266	48.21
2		8.640	7.297	34.084	51.79
**Total:**			**14.091**	**70.350**	**100.00**

Asymmetric

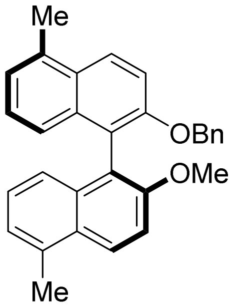

Bn-39

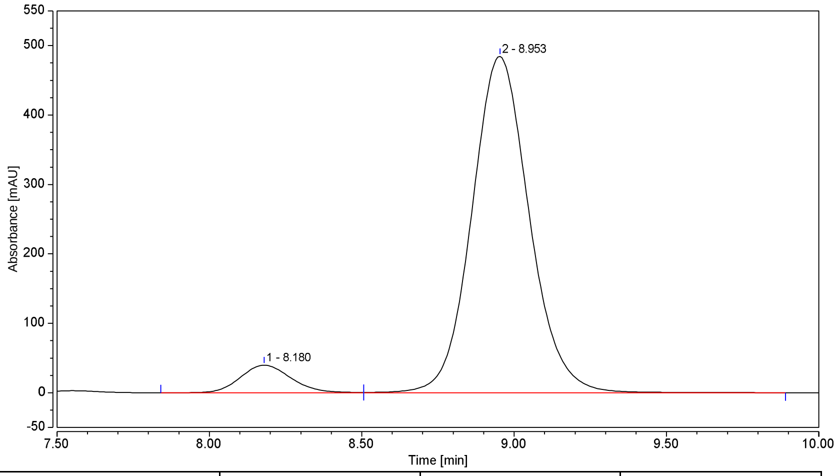

No.	Peak Name	Retention Time	Area	Height	Relative Area
		min	mAU*min	mAU	%
1		8.180	7.654	39.810	6.75
2		8.953	105.665	484.432	93.25
**Total:**			**113.319**	**524.242**	**100.00**

Chiral HPLC: (Chiralpak IC, 2% ^{i}PrOH, 98% hexane, 1.00 mL min^{-1}, λ = 290 nm) τ_R (major) = 8.0 min, τ_R (minor) = 14.9 min

Racemic

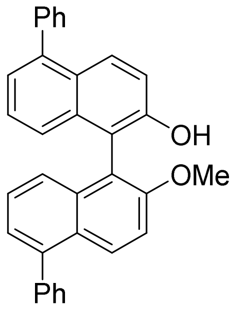

40

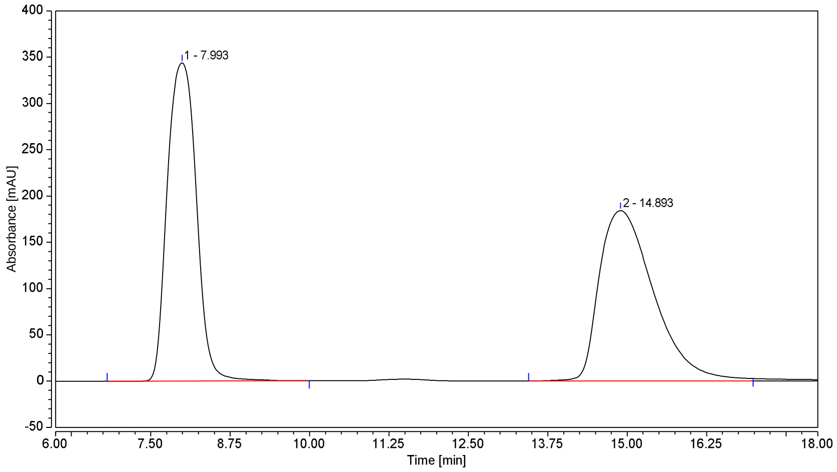

No.	Peak Name	Retention Time	Area	Height	Relative Area
		min	mAU*min	mAU	%
1		7.993	182.144	343.636	49.88
2		14.893	183.044	184.034	50.12
**Total:**			**365.188**	**527.670**	**100.00**

Asymmetric

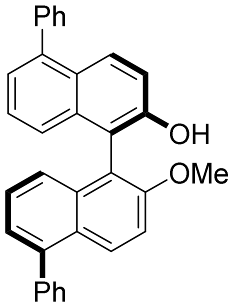

40

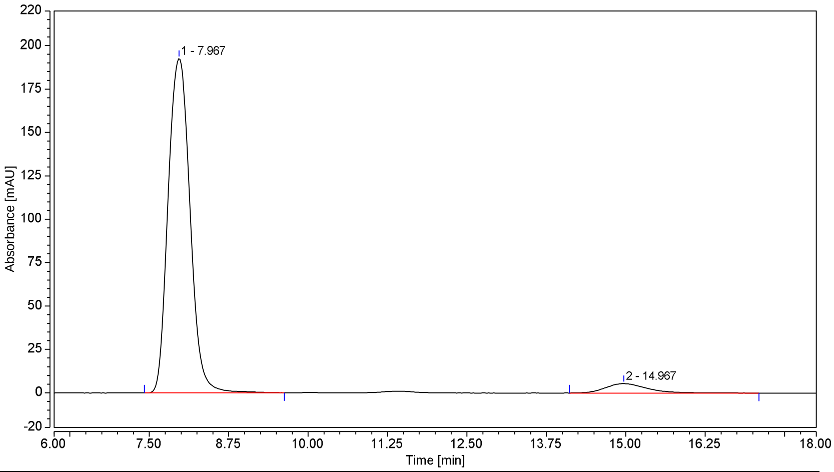

No.	Peak Name	Retention Time	Area	Height	Relative Area
		min	mAU*min	mAU	%
1		7.967	77.306	192.685	94.85
2		14.967	4.200	5.412	5.15
**Total:**			**81.506**	**198.096**	**100.00**

Chiral HPLC: (Chiralpak IC, 2% ^{i}PrOH, 99% hexane, 1.00 mL min^{-1}, λ = 290 nm) τ_R (major) = 5.0 min, τ_R (minor) = 6.2 min

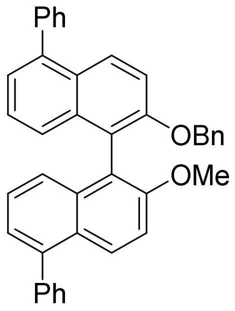

Bn-40

Racemic

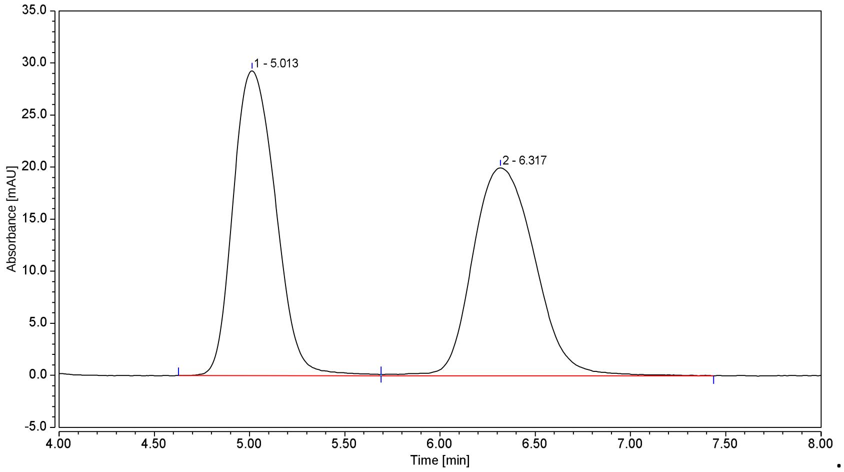

No.	Peak Name	Retention Time	Area	Height	Relative Area
		min	mAU*min	mAU	%
1		5.013	7.563	29.270	50.09
2		6.317	7.537	19.964	49.91
**Total:**			**15.101**	**49.234**	**100.00**

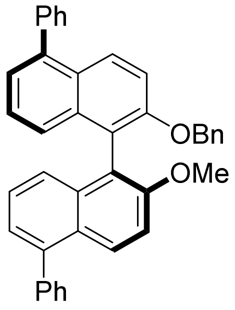

Bn-40

Asymmetric

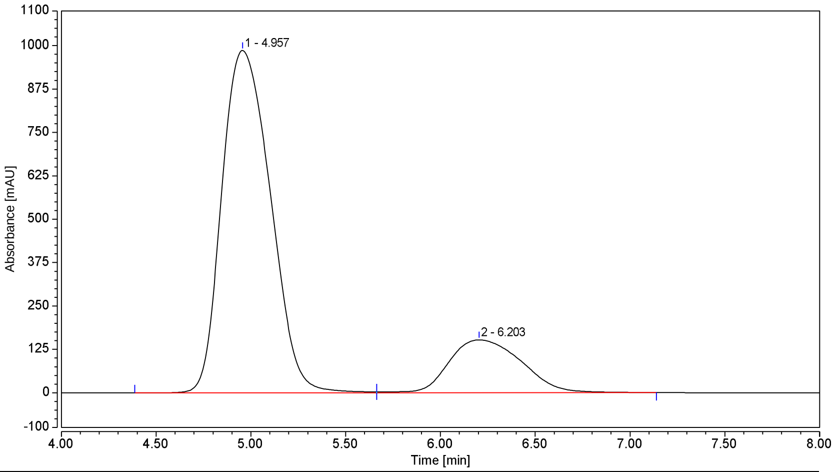

No.	Peak Name	Retention Time	Area	Height	Relative Area
		min	mAU*min	mAU	%
1		4.957	293.416	986.273	81.31
2		6.203	67.455	152.329	18.69
**Total:**			**360.872**	**1138.602**	**100.00**

Chiral HPLC: (Chiralpak ADH, 10% ^{i}PrOH, 90% hexane, 1.0 mL min^{-1}, λ = 293 nm) τ_R (major) = 15.3 min, τ_R (minor) = 31.2 min.

Racemic

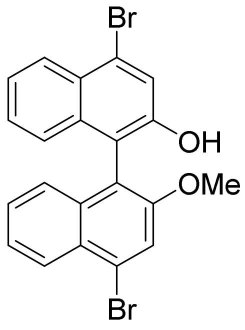

41

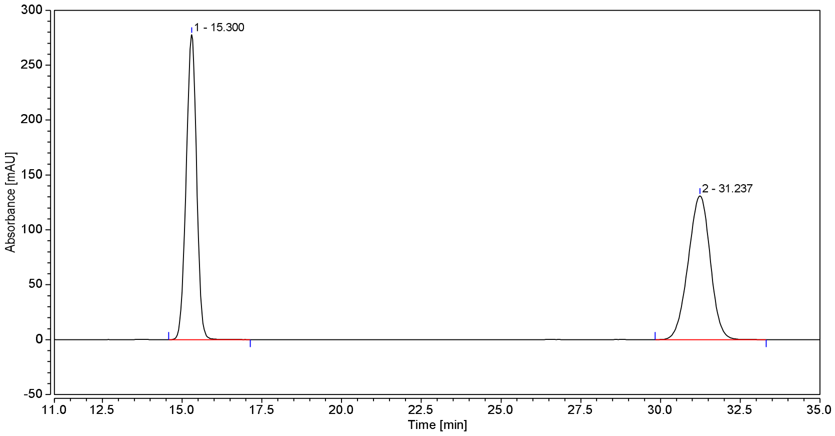

No.	Peak Name	Retention Time	Area	Height	Relative Area
		min	mAU*min	mAU	%
1		15.300	104.995	277.734	50.00
2		31.237	105.008	131.069	50.00
**Total:**			**210.003**	**408.803**	**100.00**

Asymmetric

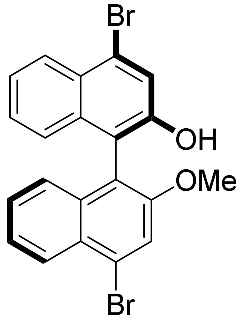

41

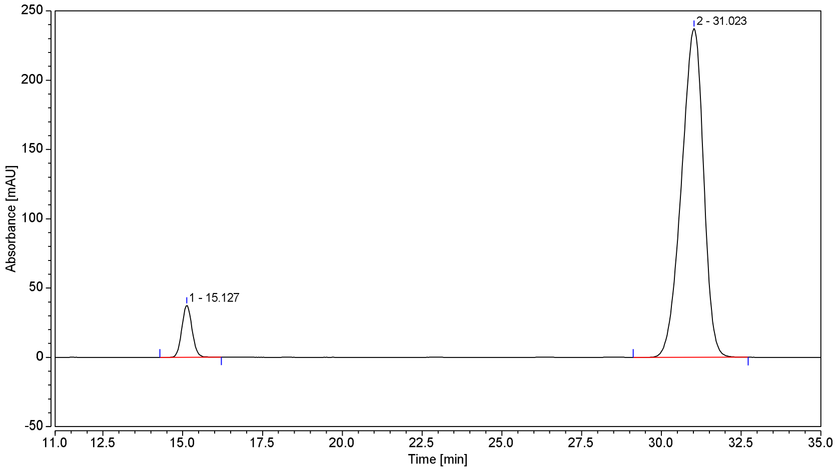

No.	Peak Name	Retention Time	Area	Height	Relative Area
		min	mAU*min	mAU	%
1		15.127	13.901	37.364	6.82
2		31.023	190.039	237.212	93.18
**Total:**			**203.940**	**274.577**	**100.00**

Chiral HPLC: (Chiralpak ADH, 1% ^{i}PrOH, 99% hexane, 1.0 mL min^{-1}, λ = 295 nm) τ_R (major) = 16.2 min, τ_R (minor) = 11.1 min.

Racemic.

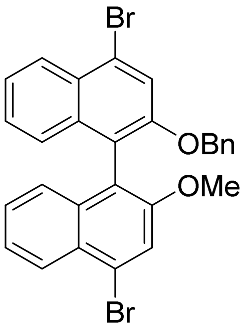

Bn-41

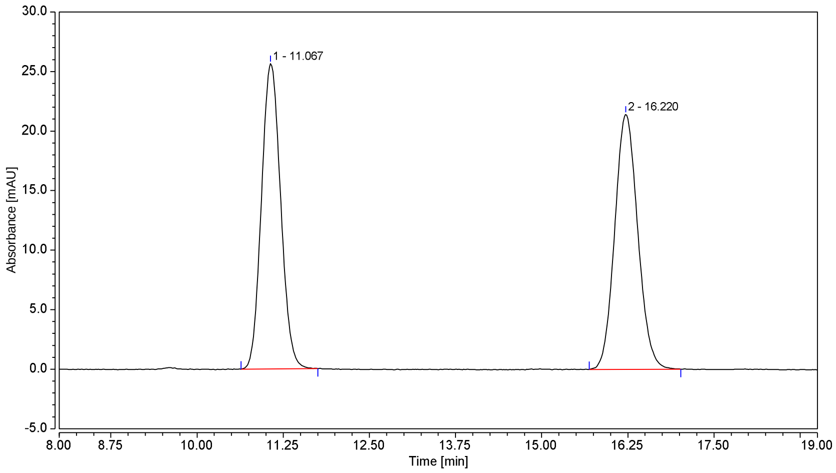

No.	Peak Name	Retention Time	Area	Height	Relative Area
		min	mAU*min	mAU	%
1		11.067	8.283	25.645	49.91
2		16.220	8.313	21.432	50.09
**Total:**			**16.596**	**47.077**	**100.00**

Asymmetric

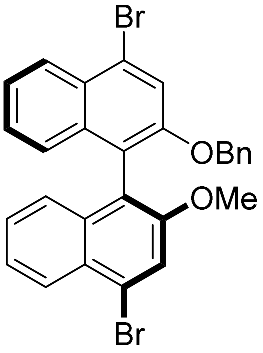

Bn-41

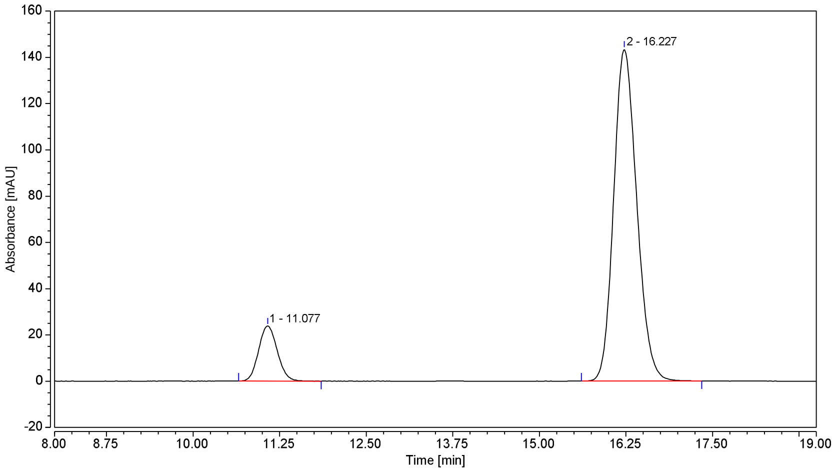

No.	Peak Name	Retention Time	Area	Height	Relative Area
		min	mAU*min	mAU	%
1		11.077	7.479	23.833	11.90
2		16.227	55.370	143.458	88.10
**Total:**			**62.850**	**167.290**	**100.00**

Chiral HPLC: (Chiralpak ADH, 20% ^{i}PrOH, 80% hexane, 1.0 mL min^{-1}, λ = 300 nm) τ_R (major) = 20.8 min, τ_R (minor) = 36.0 min.

Racemic

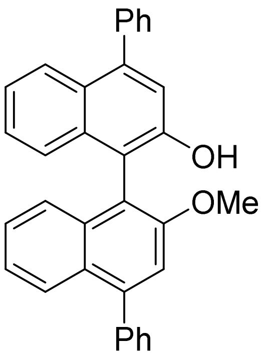

42

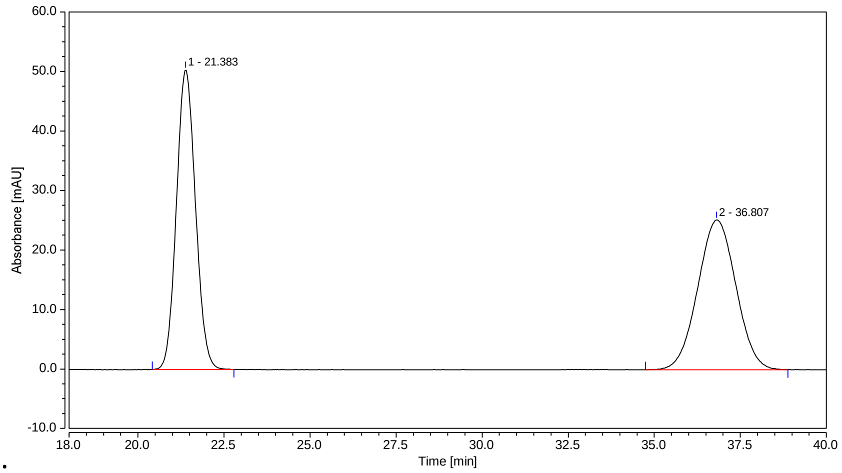

No.	Peak Name	Retention Time	Area	Height	Relative Area
		min	mAU*min	mAU	%
1		21.383	32.337	50.408	49.95
2		36.807	32.404	25.195	50.05
**Total:**			**64.742**	**75.602**	**100.00**

Asymmetric

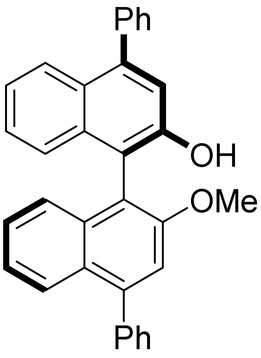

42

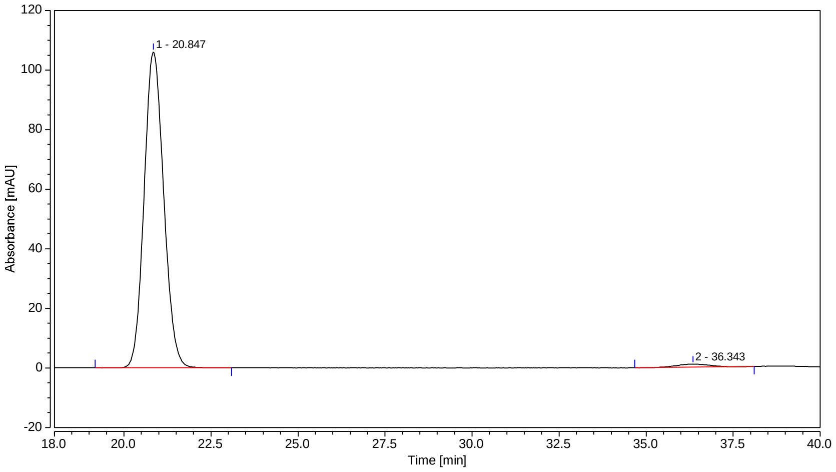

No.	Peak Name	Retention Time	Area	Height	Relative Area
		min	mAU*min	mAU	%
1		20.847	69.276	106.129	98.50
2		36.343	1.055	1.005	1.50
**Total:**			**70.331**	**107.134**	**100.00**

Chiral HPLC: (Chiralpak ODH, 2% ^{i}PrOH, 98% hexane, 1.0 mL min^{-1}, λ = 295 nm) τ_R (major) = 10.7 min, τ_R (minor) = 9.0 min.

Racemic.

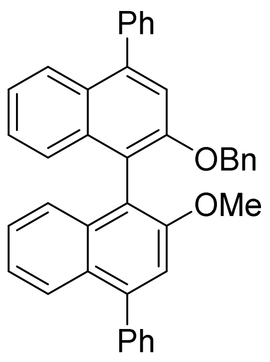

Bn-42

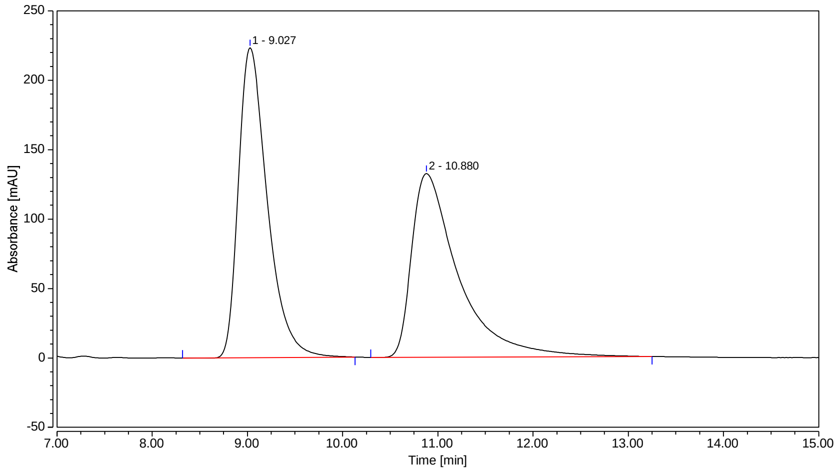

No.	Peak Name	Retention Time	Area	Height	Relative Area
		min	mAU*min	mAU	%
1		9.027	78.521	223.291	51.04
2		10.880	75.320	132.306	48.96
**Total:**			**153.841**	**355.597**	**100.00**

Asymmetric

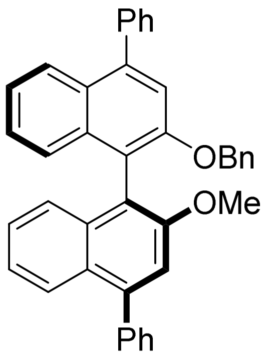

Bn-42

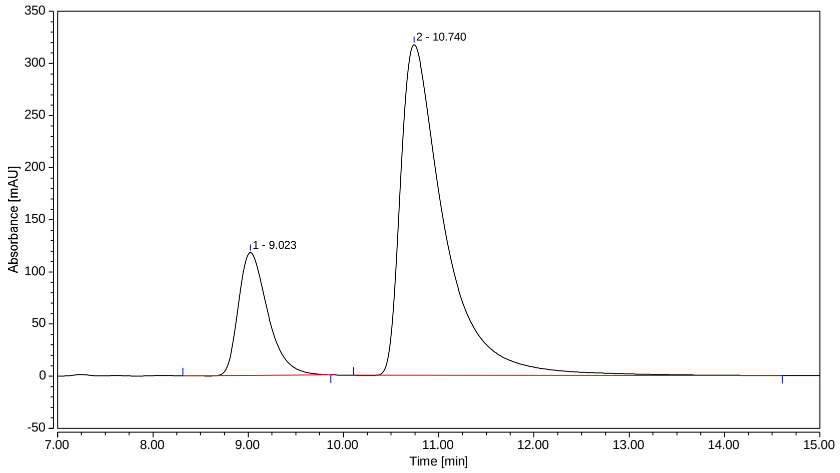

No.	Peak Name	Retention Time	Area	Height	Relative Area
		min	mAU*min	mAU	%
1		9.023	41.910	117.882	19.81
2		10.740	169.701	317.325	80.19
**Total:**			**211.611**	**435.206**	**100.00**

Chiral HPLC: (Chiralpak ADH, 10% ^{i}PrOH, 90% hexane, 1.0 mL min^{-1}, λ = 293 nm) τ_R (major) = 20.2 min, τ_R (minor) = 23.5 min.

<u>Racemic</u>

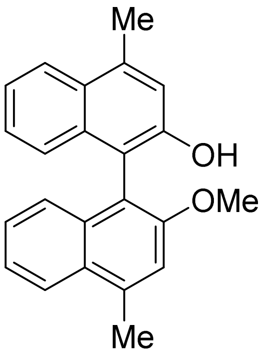

43

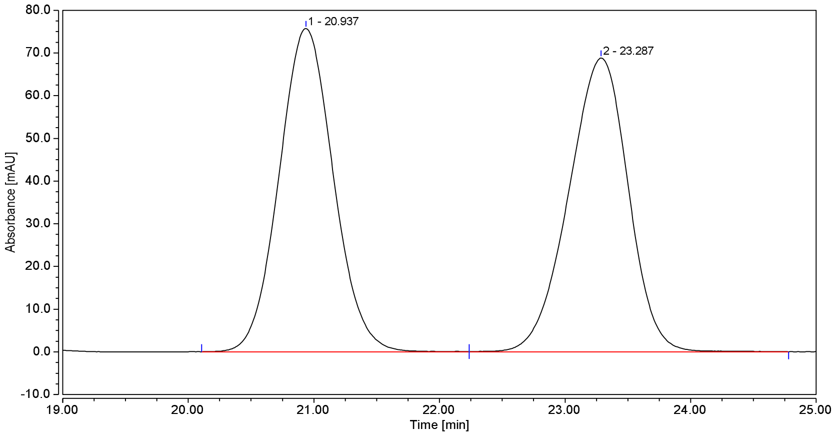

No.	Peak Name	Retention Time	Area	Height	Relative Area
		min	mAU*min	mAU	%
1		20.937	37.991	75.721	50.14
2		23.287	37.785	68.805	49.86
**Total:**			**75.776**	**144.526**	**100.00**

<u>Asymmetric</u>

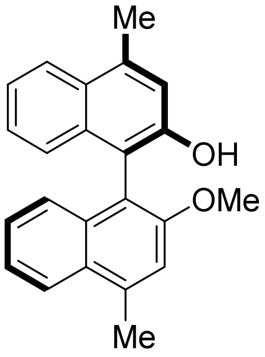

43

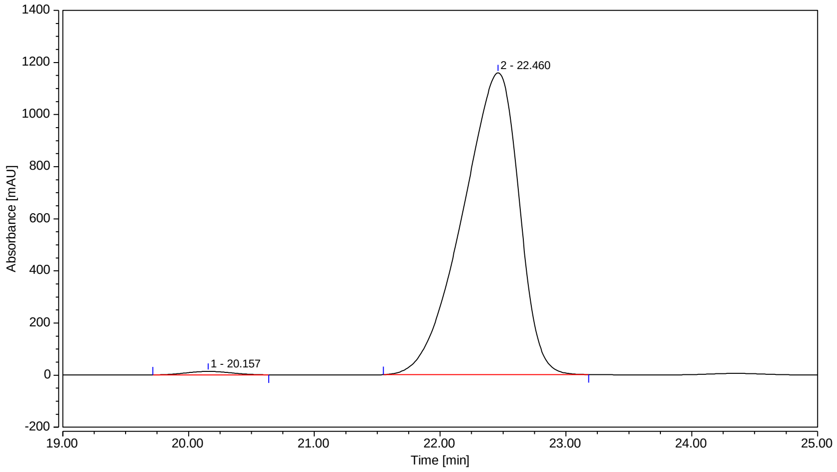

No.	Peak Name	Retention Time	Area	Height	Relative Area
		min	mAU*min	mAU	%
1		20.157	5.697	13.510	0.94
2		22.460	601.624	1158.301	99.06
**Total:**			**607.322**	**1171.811**	**100.00**

Chiral HPLC: (Chiralpak ADH, 3% ^{i}PrOH, 99% hexane, 1.0 mL min^{-1}, λ = 250 nm) τ_R (major) = 19.7 min, τ_R (minor) = 12.0 min.

Racemic.

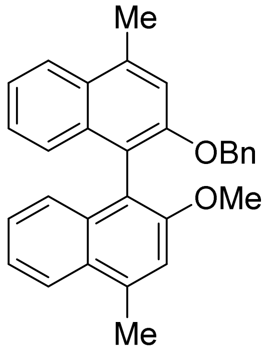

Bn-43

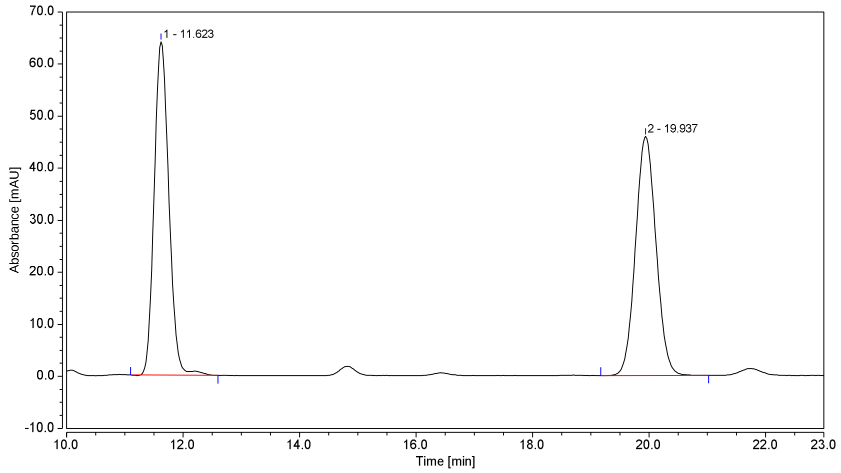

No.	Peak Name	Retention Time	Area	Height	Relative Area
		min	mAU*min	mAU	%
1		11.623	18.740	64.096	50.04
2		19.937	18.707	45.935	49.96
**Total:**			**37.448**	**110.031**	**100.00**

Asymmetric

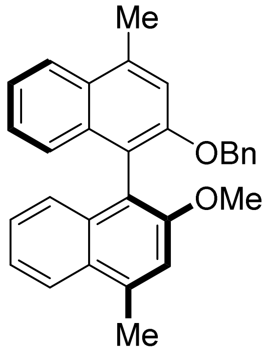

Bn-43

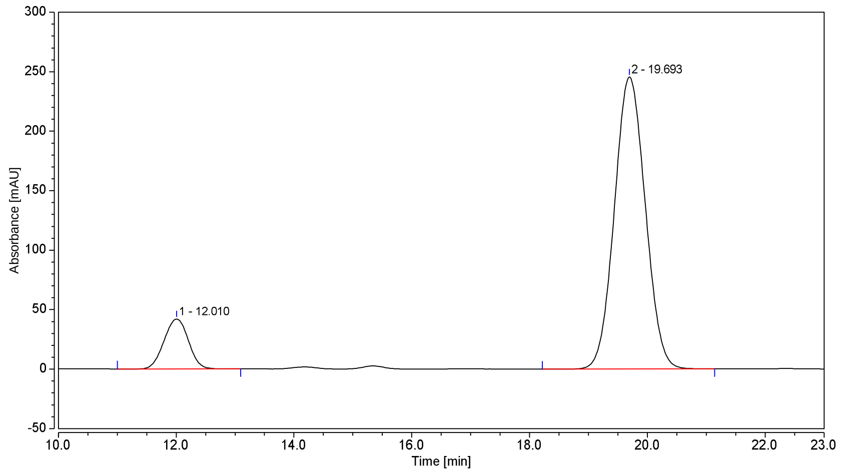

No.	Peak Name	Retention Time	Area	Height	Relative Area
		min	mAU*min	mAU	%
1		12.010	20.000	41.920	11.83
2		19.693	149.017	245.426	88.17
**Total:**			**169.017**	**287.346**	**100.00**

Chiral HPLC: (Chiralpak ADH, 7.5% ^{i}PrOH, 92.5% hexane, 1.00 mL min^{-1}, λ = 290 nm) τ_R (major) = 25.4 min, τ_R (minor) = 20.2 min

Racemic

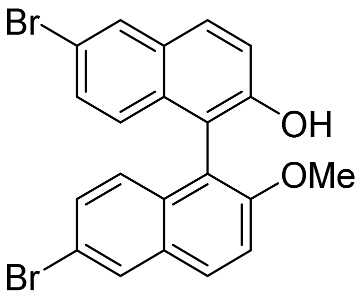

33

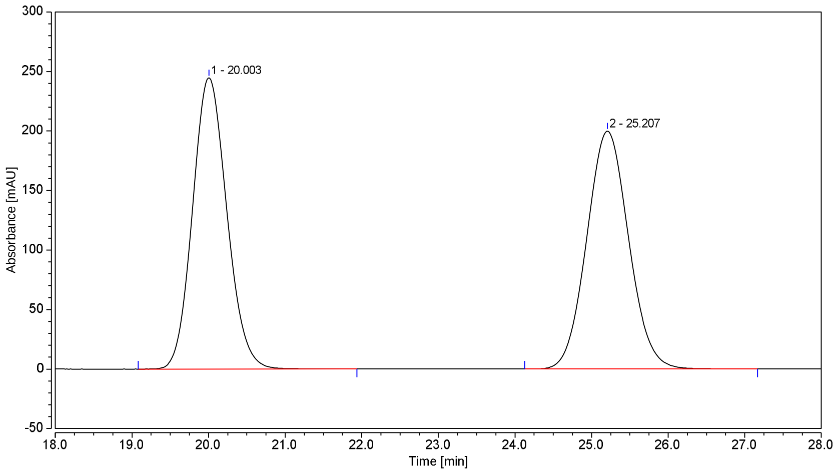

No.	Peak Name	Retention Time	Area	Height	Relative Area
		min	mAU*min	mAU	%
1		20.003	126.546	244.803	49.99
2		25.207	126.597	199.952	50.01
**Total:**			**253.144**	**444.755**	**100.00**

Asymmetric

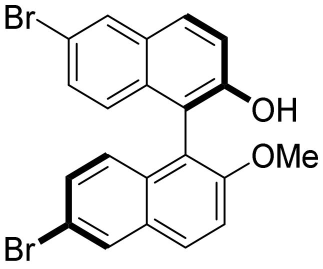

33 (10 g scale)

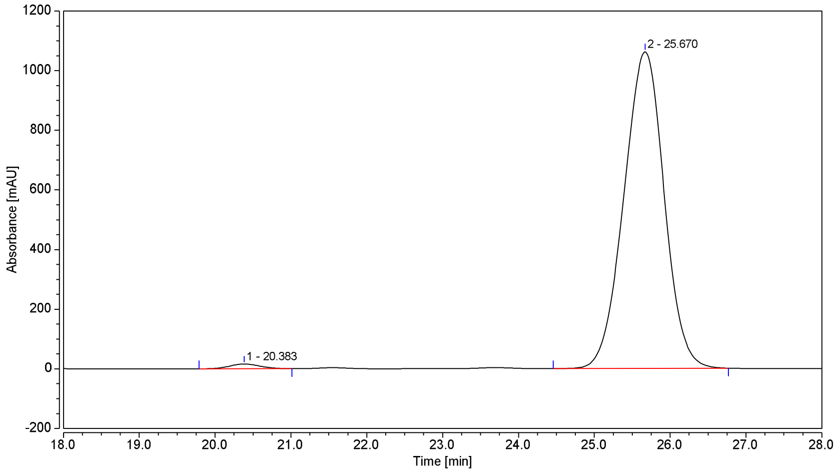

No.	Peak Name	Retention Time	Area	Height	Relative Area
		min	mAU*min	mAU	%
1		20.383	7.395	15.728	1.12
2		25.670	653.285	1062.077	98.88
**Total:**			**660.680**	**1077.805**	**100.00**

Chiral HPLC: (Chiralpak ADH, 7.5% ^{i}PrOH, 92.5% hexane, 1.00 mL min^{-1}, λ = 290 nm) τ_R (major) = 13.3 min, τ_R (minor) = 10.8 min

Racemic

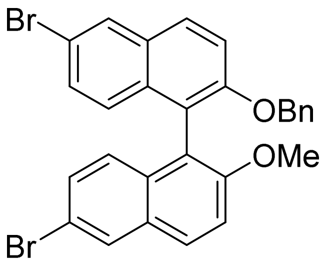

33

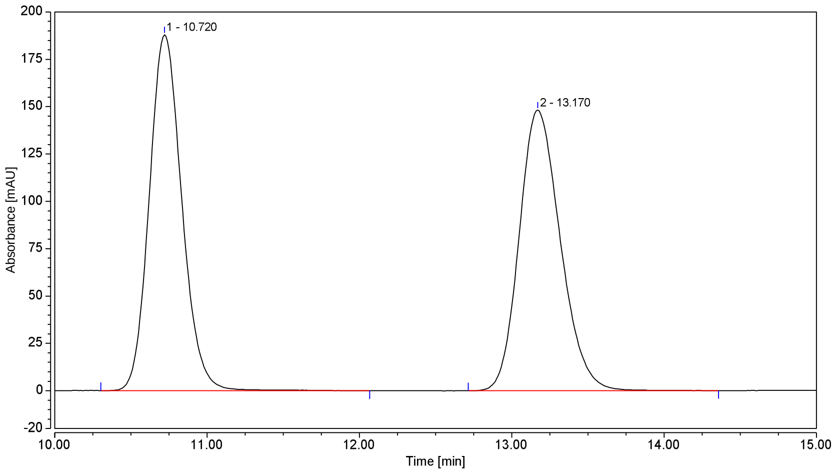

No.	Peak Name	Retention Time	Area	Height	Relative Area
		min	mAU*min	mAU	%
1		10.720	46.477	187.937	50.03
2		13.170	46.425	148.219	49.97
**Total:**			**92.902**	**336.156**	**100.00**

Asymmetric

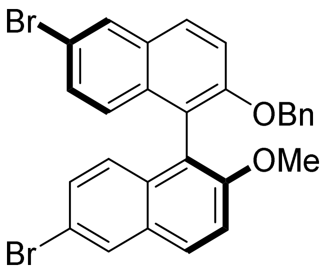

Bn-33 (10 g scale)

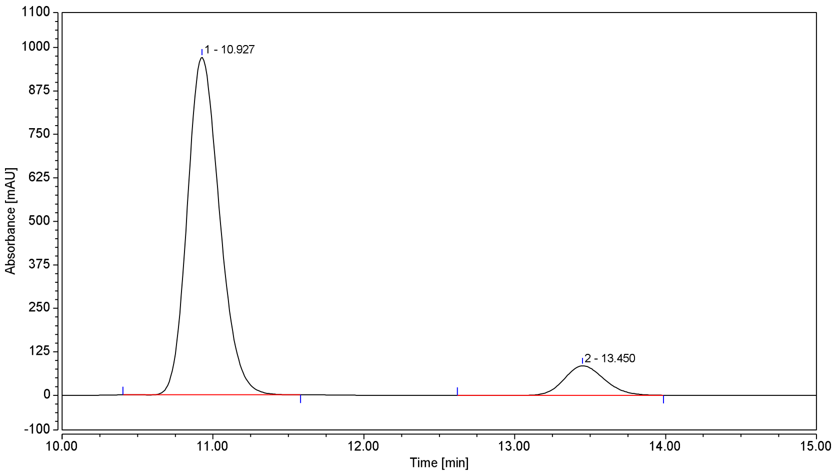

No.	Peak Name	Retention Time	Area	Height	Relative Area
		min	mAU*min	mAU	%
1		10.927	240.504	970.447	90.22
2		13.450	26.069	84.658	9.78
**Total:**			**266.573**	**1055.105**	**100.00**
